# Total Synthesis and Structural Studies of Zwitterionic *Bacteroides fragilis* Polysaccharide A1 Fragments

**DOI:** 10.1021/jacs.3c03976

**Published:** 2023-06-13

**Authors:** Zhen Wang, Ana Poveda, Qingju Zhang, Luca Unione, Herman S. Overkleeft, Gijsbert A. van der Marel, Jiménez-Barbero Jesús, Jeroen D. C. Codée

**Affiliations:** † Leiden Institute of Chemistry, Leiden University, Einsteinweg 55, 2333 CC Leiden, The Netherlands; ‡ CIC bioGUNE, Basque Research & Technology Alliance (BRTA), Bizkaia Technology Park, Building 800, 48162 Derio, Bizkaia, Spain; § National Research Centre for Carbohydrate Synthesis, Jiangxi Normal University, 99 Ziyang Avenue, Nanchang 330022, China; ∥ Ikerbasque, Basque Foundation for Science, Maria Diaz de Haro 3, 48013 Bilbao, Bizkaia, Spain; ⊥ Department of Organic Chemistry II, Faculty of Science and Technology, University of the Basque Country, EHU-UPV, 48940 Leioa, Spain; # Centro de Investigación Biomédica En Red de Enfermedades Respiratorias (CIBERES), 28029 Madrid, Spain

## Abstract

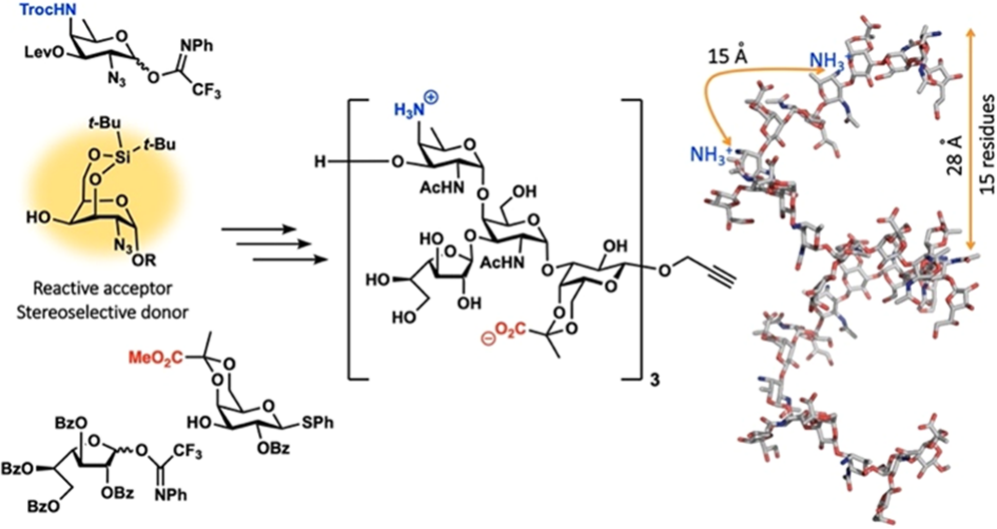

Zwitterionic polysaccharides (ZPSs) are exceptional carbohydrates, carrying both positively charged amine groups and negatively charged carboxylates, that can be loaded onto MHC-II molecules to activate T cells. It remains enigmatic, however, how these polysaccharides bind to these receptors, and to understand the structural features responsible for this “peptide-like” behavior, well-defined ZPS fragments are required in sufficient quantity and quality. We here present the first total synthesis of *Bacteroides fragilis* PS A1 fragments encompassing up to 12 monosaccharides, representing three repeating units. Key to our successful syntheses has been the incorporation of a C-3,C-6-silylidene-bridged “ring-inverted” galactosamine building block that was designed to act as an apt nucleophile as well as a stereoselective glycosyl donor. Our stereoselective synthesis route is further characterized by a unique protecting group strategy, built on base-labile protecting groups, which has allowed the incorporation of an orthogonal alkyne functionalization handle. Detailed structural studies have revealed that the assembled oligosaccharides take up a bent structure, which translates into a left-handed helix for larger PS A1 polysaccharides, presenting the key positively charged amino groups to the outside of the helix. The availability of the fragments and the insight into their secondary structure will enable detailed interaction studies with binding proteins to unravel the mode of action of these unique oligosaccharides at the atomic level.

## Introduction

**Figure 1:**
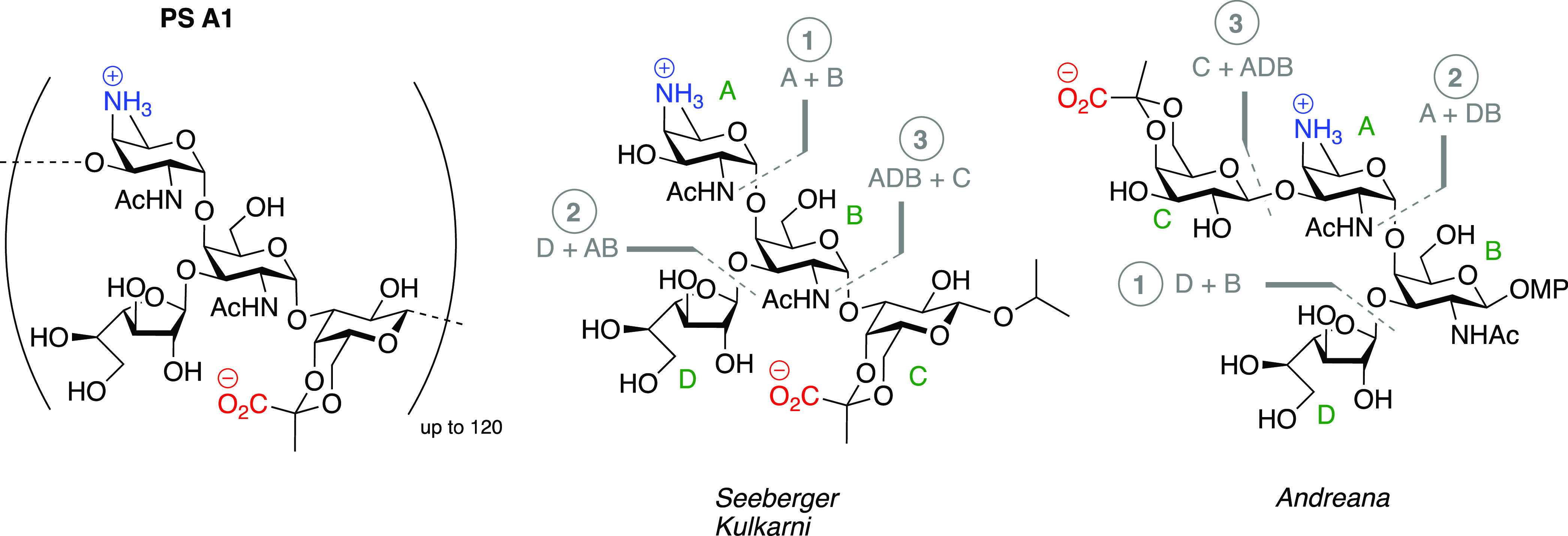
Structure of the zwitterionic polysaccharide PS A1 and the tetrasaccharides synthesized to date.

Zwitterionic polysaccharides (ZPSs) present a unique class of carbohydrates from both a structural and biological perspective.^1^ These bacterial polysaccharides are characterized by the presence of positively and negatively charged groups on the carbohydrate backbone, and this not only differentiates them from other carbohydrates; it also bestows the ZPSs with distinctive biological activity.^2−4^ It is the only class of carbohydrates that elicits a T-cell-dependent immune response, a mode of action normally restricted to peptides.^5,6^ In addition, it has been reported that ZPSs can stimulate the innate immune system through interaction with Toll-like receptor 2 (TLR2), thereby linking innate and adaptive immune responses.^7^ The availability of polymeric ZPSs from biological sources has led to global insight into their mode of action.^8−12^ Knowledge on how and why they interact with their binding partners on the molecular level, however, is lacking. Therefore, the availability of well-defined, polymer fragments would be of great value. PS A1 (Figure 1) is one of the best-studied ZPSs, and it is a capsular polysaccharide (CPS) of *Bacteroides fragilis*, which causes intraabdominal abscesses and sepsis in humans.^13,14^ It is composed of tetrasaccharide repeating units (RUs), built up from a β-d-galactofuranose, an *N*-acetyl-α-d-galactosamine, a negatively charged pyruvate-functionalized β-d-galactopyranose, and the rare 2-acetamido-4-amino-2,4,6-trideoxy-α-d-galactose (AAT).^15^ It has been shown, through chemical modification of PS A1 polymers, that the positively charged amino groups and negatively charged carboxylates are required for the unique immunomodulatory activity of the polysaccharide.^2,3,16^ Furthermore, it has been proposed that the PS A1 polysaccharide can take up a secondary structure to properly position the positively and negatively charged groups to interact with MHC-II molecules to present the ZPS to their T-cell receptors. Structural studies on the ZPSs PS A2 (a polysaccharide built up from very different monosaccharides) and the *Streptococcus pneumonia* serotype 1 polysaccharide (Sp1) have revealed these ZPSs to adopt a right-handed helical structure.^17−19^ We have shown, through the generation of synthetic fragments, that an Sp1 oligosaccharide encompassing three repeating units (*i.e.*, a nonasaccharide) can complete a full helical turn, emulating the secondary structure of the polymer.^18^ Detailed structural studies on PS A1 have not been reported.

To unravel the mode of action of PS A1 oligosaccharides at the molecular level, several efforts have been undertaken to synthesize these complex targets.^20^ The PS A1 structure represents multiple challenging structural features, including the presence of the rare AAT residues and the *cis*-linkages through which two of the four constituting monomers are connected. Three groups have previously succeeded in synthesizing the PS A1 repeating unit. Pragani and Seeberger described the synthesis of the PS A1 tetrasaccharide repeating unit having an *iso*-propyl cap at the reducing end.^21,22^ They first evaluated a glycosylation strategy in which an AAT donor (monosaccharide A) was condensed with a DBC trisaccharide, but in line with our prior studies,^23^ they found this glycosylation to be nonproductive because of significant steric hindrance. Switching to a [3 + 1] glycosylation strategy using an ADB trisaccharide donor and a pyruvate galactose monosaccharide (C) acceptor allowed them to complete the assembly of the tetrasaccharide (see Figure 1). Later, Kulkarni and co-workers followed the same strategy with a slightly different protecting group scheme to generate an identical tetrasaccharide.^24^ In contrast to Seeberger’s *de novo* approach to access the required AAT building block from l-threonine, Kulkarni and co-workers developed an efficient one-pot double nucleophilic substitution on a d-rhamnose building block. Andreana and co-workers assembled a *p*-methoxyphenol-capped PS A1 tetrasaccharide, representing a frame-shifted repeating unit (CADB). They built this tetrasaccharide using a [1 + 3] strategy, coupling a pyruvate galactosyl donor (C) to an ADB trisaccharide.^25^ None of these strategies allowed for the elongation to generate larger oligosaccharides.

We here reported on the development of a synthetic strategy to generate larger PS A1 fragments, and we describe the synthesis of PS A1 oligosaccharides up to the dodecasaccharide level (three RUs). We have generated fragments with and without the galactofuranose branches to probe the influence of these side chains on the structure of the oligomers. Our synthetic strategy hinges on the use of a conformationally “inverted” and restricted galactosamine building block that we show to serve well as both a donor and acceptor building block. By inverting the ring conformation of the galactosamine, the axial C-4-OH, which is a very difficult alcohol to glycosylate (vide supra), is placed in an equatorial orientation to improve its reactivity.^26^ At the same time, the 3,6-silylidene ketal shields the top face of the building block, which allows for highly stereoselective glycosylation reactions when these building blocks are used as donor glycoside.^27^ While it is commonplace in contemporary oligosaccharide synthesis to use benzyl-type protecting groups for permanent protection of the growing oligosaccharide chain, our developed protecting group strategy builds on the use of base-labile permanent protecting groups, which has allowed the incorporation of an alkyne spacer at the reducing end, that can be used for future conjugation purposes through a copper-catalyzed alkyne–azide click (CuAAC) reaction.^28^ The synthetic structures thus prepared enabled us to conduct a detailed study of their 3D structure in solution by using a combination of NMR spectroscopy and molecular dynamics (MD) techniques. They adopt a well-defined bent structure, which translates to a left-handed helical structure for longer PS A1 polysaccharides, with the galactofuranose appendages being solvent-exposed, positioning the negatively charged carboxylates parallel and the positively charged amines perpendicular to the helix axis. The availability of well-defined oligomers and insight into their 3D structure now pave the way to unravel the atomic details of their binding to MHC-II molecules and T-cell receptors as well as other immune receptors, such as TLR2 and antibodies.

## Results and Discussion

### Targets and Strategy

**Scheme 1:**
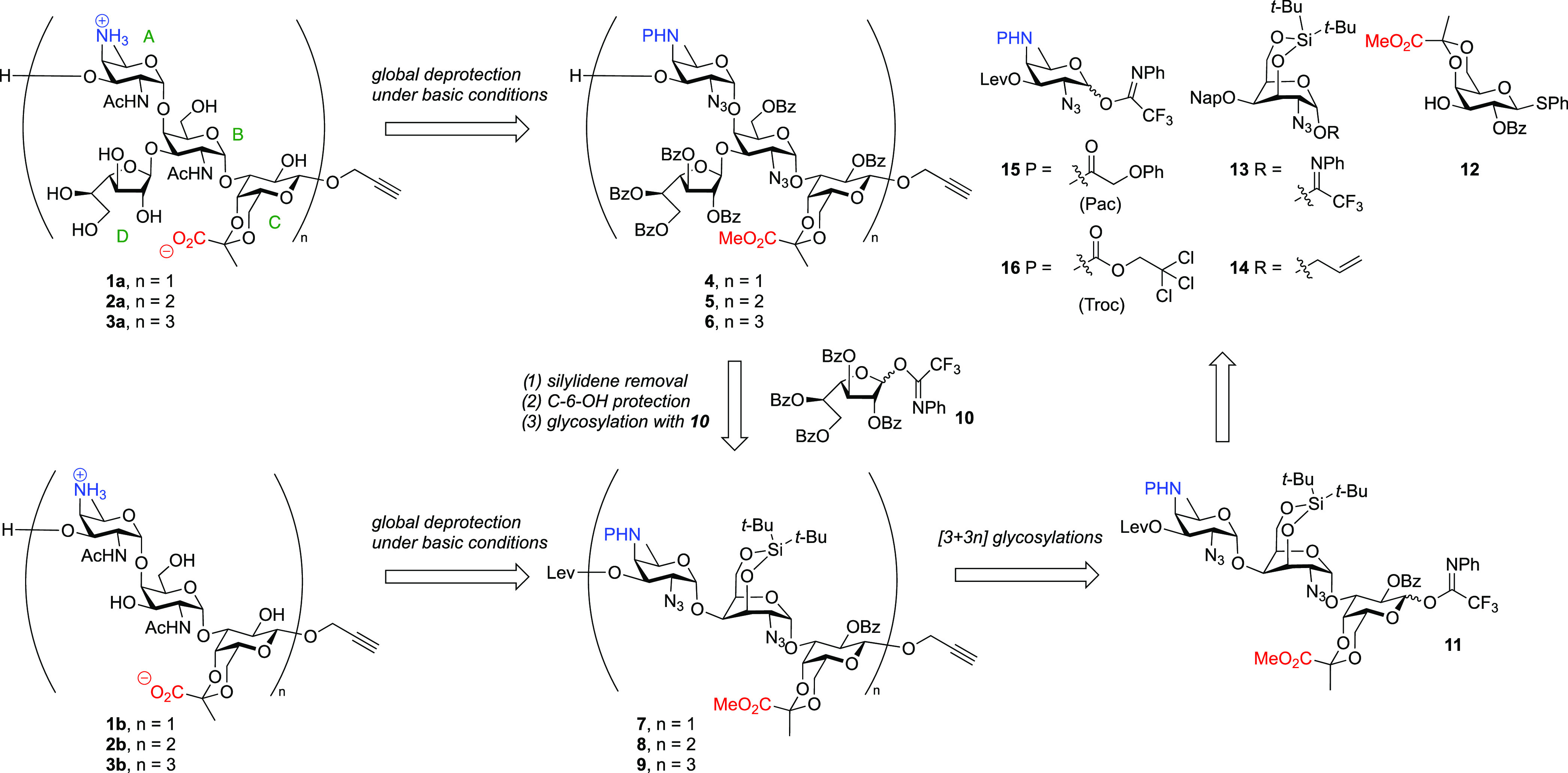
Retrosynthetic Analysis for the Assembly of Target Oligosaccharides **1a**–**3a** and **1b**–**3b**

**Scheme 2:**
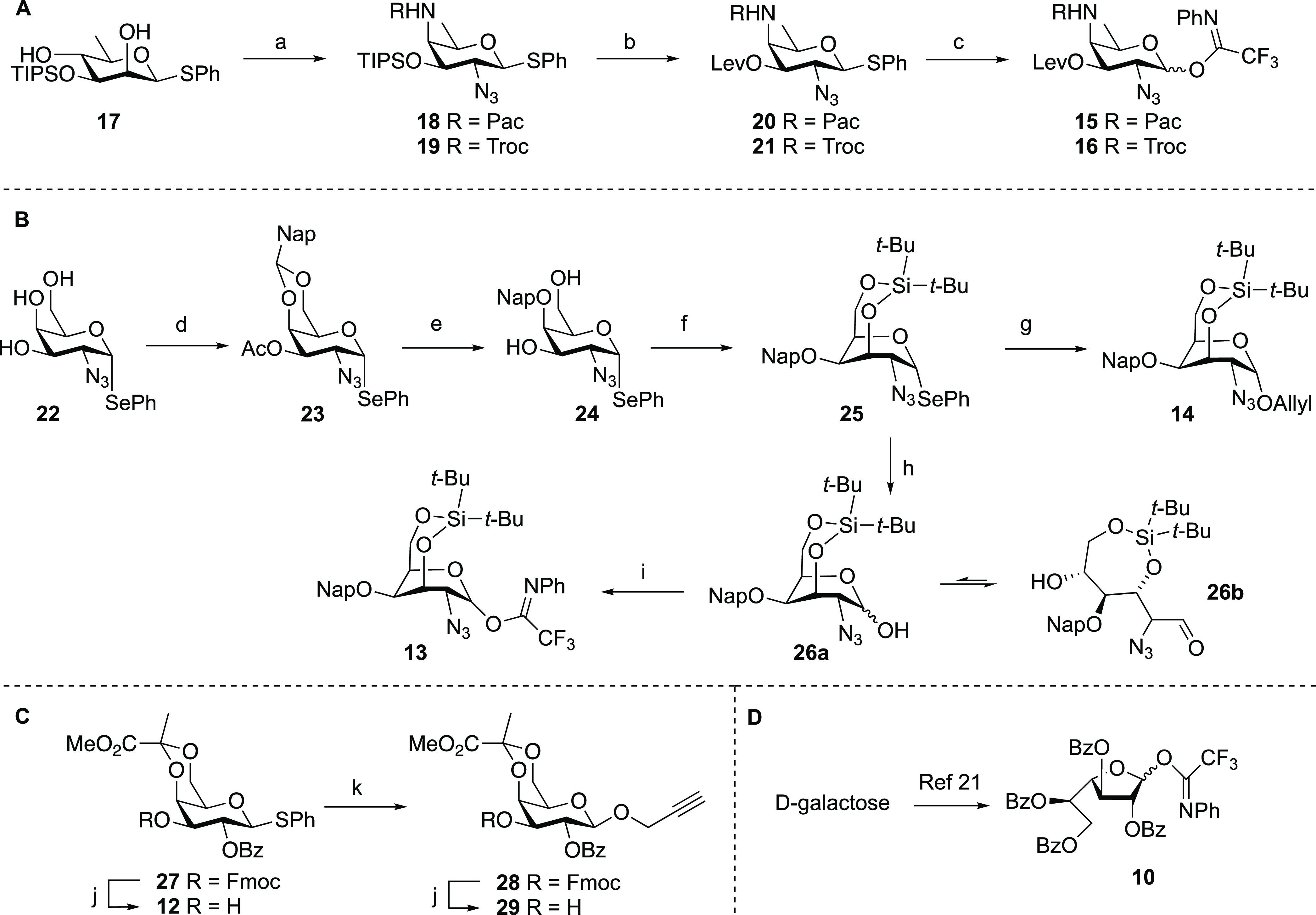
Synthesis of the AAT and Galactosamine Building Blocks (A) Synthesis of the AAT building blocks. (B) Synthesis of the 3,4-silylidene donor and acceptor synthons. (C) Synthesis of pyruvate galactose building blocks. Reagents and conditions: (a) (i) Tf_2_O, Py, DMAP, DCM, −10 to 10 °C; (ii) TBAN_3_, MeCN, −30 to −20 °C; (iii) 7N NH_3_ in MeOH; (iv) PacCl or TrocCl NaHCO_3_, THF, H_2_O, 0 °C to rt; (v) TBAF, AcOH, THF, five steps, **18**, 53%; **19**, 32%. (b) LevOH, EDCI, DMAP, DCM, **20**, quant.; **21**, 95%. (c) (i) NIS, TFA, DCM, 0; (ii) *N*-phenyltrifluoroacetimidoyl chloride, Cs_2_CO_3_, acetone, **15**, 64%; **16**, 82%; (d) (i) 2-(Dimethoxymethyl)naphthalene, CSA, MeCN; (ii) Ac_2_O, Py, quant. (e) (i) BH_3_/THF, Bu_2_BOTf, DCM, 84%; (ii) NaOMe, MeOH, DCM, 99%; (f) (*t*-Bu)_2_Si(OTf)_2_, 2,6-lutidine, 4 Å MS, 100 °C, 67%; (g) (i) AllylOH, NIS, TfOH, 4 Å MS, 0 °C, 90%; (ii) DDQ, DCM, water, 86%; (h) NIS, acetone, water, 0 °C, 98%; (i) *N*-phenyltrifluoroacetimidoyl chloride, Cs_2_CO_3_, acetone, 75%; (j) Et_3_N, DCM, **12**, 83%; **29**, 75%; (k) Ph_2_SO, TTBP, Tf_2_O, Propynyl alcohol, −60 to −40 °C, 80%; PacCl = phenoxyacetyl chloride, DCM = dichloromethane, DMAP = 4-dimethylaminopyridine, EDCI = 1-(3-dimethylaminopropyl)-3-ethylcarbodiimide, NIS = *N*-iodosuccinimide, Py = pyridine, TBAF = tetrabutylammonium fluoride, TBAN_3_ = tetrabutylammonium azide, Tf = trifluoromethanesulfonyl, TFA = trifluoroacetic acid, TIPS = triisopropylsilyl. DDQ = 2,3-dichloro-5,6-dicyano-*p*-benzoquinone, Ph_2_SO = diphenylsulfoxide, TTBP = 2,4,6-Tri-*tert*-butylpyrimidine, LevOH = levulinic acid, CSA = camphorsulfonic acid.

Our target oligosaccharides are depicted in Scheme 1, and the set of molecules encompasses PS A1 fragments ranging from one to three repeating units (fragments **1a**–**3a**) as well as structures lacking the galactofuranosyl appendages (fragments **1b**–**3b**). We and Seeberger and co-workers previously found that the C-4-OH of the galactosazide moiety represents a challenging nucleophile to glycosylate (vide supra), and we therefore opted for the use of a galactosazide building block having an “inverted” ring conformation. We reasoned that locking this building block in a ^1^C_4_-type chair conformation would turn the relatively unreactive axial hydroxyl^29−31^ into an equatorially disposed nucleophile that would be more reactive. In addition, we projected that tethering the C-3-OH and C-6-OH with a di-*tert*-butyl silylidene group would shield the top face of the building block, thus directing glycosylation reactions of these donors to the α-face to forge the challenging 1,2-*cis*-galactosamine linkages. The use of the 3,6-*O*-di-*tert*-butyl silylidene-functionalized building blocks in our synthetic plan is retrosynthetically depicted in Scheme 2. We aimed to assemble the target fragments **1a**–**3a** from their fully protected precursors **4**–**6**, and we reasoned that we could use base-labile protecting groups for all hydroxyl functionalities, the pyruvate acid, and the AAT amino group. Oligosaccharides **4**–**6** and **1b**–**3b**, lacking the galactofuranosyl residues, can be obtained from the protected oligosaccharide backbones **7**–**9**, which are to be obtained using trisaccharide building block **11**. The trisaccharide building blocks can be stereoselectively linked, building on the anchimeric assistance of the benzoyl group at the C-2 of the pyruvalated galactose in **11**. The trisaccharide building blocks will be built from monosaccharides **12**–**16.** Our global deprotection strategy necessitates the use of a base-labile protecting group on the AAT C-4-nitrogen, and we initially investigated the use of a phenoxyacetyl (Pac) group. Because we found that this group was too stable at the oligosaccharide stage (vide infra), a trichloroethoxycarbonyl (Troc) was employed in the final, successful assemblies. Azide groups were used to serve as precursors for the acetamide functionalities in the galactosamine and AAT building blocks (**13**, **14** and **15**, **16**, respectively) to serve as nonparticipating groups in the construction of the *cis*-glycosidic linkages.

### First-Generation Assembly

The assembly of the required building blocks is depicted in Scheme 2. The AAT building blocks **15** and **16** were assembled using Kulkarni’s strategy^32^ starting from 3-*O*-tri-*iso*-propylsilyl-protected β-rhamnose building block **16** as recently reported (Scheme 2A).^19^ Triflation of both the C-2 and C-4 hydroxy groups was followed by inversion of the C-2-triflate with an azide and subsequent substitution of the somewhat less reactive C-4-triflate with ammonia to give the AAT core structure. The so-introduced C-4-amine was protected with either a Pac group or masked as a Troc carbonate, after which the silyl ether was removed and a levulinoyl ester was installed to give AAT building blocks **20** and **21**. The thioglycosides were next transformed into the corresponding *N*-phenyl trifluoroacetimidate donors **15** and **16**, respectively.

The key silylidene-protected galactosazide building blocks were assembled as shown in Scheme 2B. Starting from known galactosazide **22**, we first installed a naphthylidene acetal on the C-4 and C-6 hydroxy groups and masked the remaining alcohol as an acetyl ester. Reductive opening of the naphthylidene acetal using borane in combination with dibutylboron triflate gave the C-4-*O*-naphthylmethyl ether, after which saponification of the acetyl ester delivered the 3,6-diol. Installation of the bridging silylidene required strenuous conditions and was effected using di(*tert*-butyl)silyl bistriflate and 2,6-lutidine at elevated temperature. The target silylidene-protected galactosazide was obtained in a 67% yield alongside several partially silylated side products. These side products could be treated with TBAF and AcOH to return the starting diol (24% recovery; see the Supporting Information). The silylidene-bridged selenodonor was next transformed into allyl galactoside **14** in a 90% yield by condensation with allyl alcohol under the agency of *N*-iodosuccinimide and triflic acid. Notably, this condensation proceeded completely stereoselectively to provide the *cis*-linked product, indicating that the 3,6-silylidene bridge effectively shields the top face of the galactosazide donor, in line with the results described by Bols and co-workers for 3,6-silylidene galactosyl donors.^27^ In parallel, we generated imidate donor **13** from its selenophenyl precursor. To this end, we hydrolyzed the selenoacetal to provide lactol **26a**. This lactol spontaneously ring-opened to provide the corresponding aldehyde **26b**. Gratifyingly, the treatment of **26b** with Cs_2_CO_3_ and *N*-phenyl trifluoroacetamidoyl chloride uneventfully provided the target imidate donor **13** in a 75% yield.

The required pyruvate galactose building blocks were obtained from thioglycoside **27**, which was previously reported by Seeberger and co-workers.^21^ Removal of the 9-fluorenylmethyl carbonate delivered building block **12**, while condensation of **27** with propargyl alcohol using the benzene diphenylsulfoxide-triflic anhydride (Tf_2_O) couple^33,34^ and liberation of the C-3-OH using triethylamine gave **29**. Galactofuranose building block **10** was generated as previously described.^21^

**Scheme 3:**
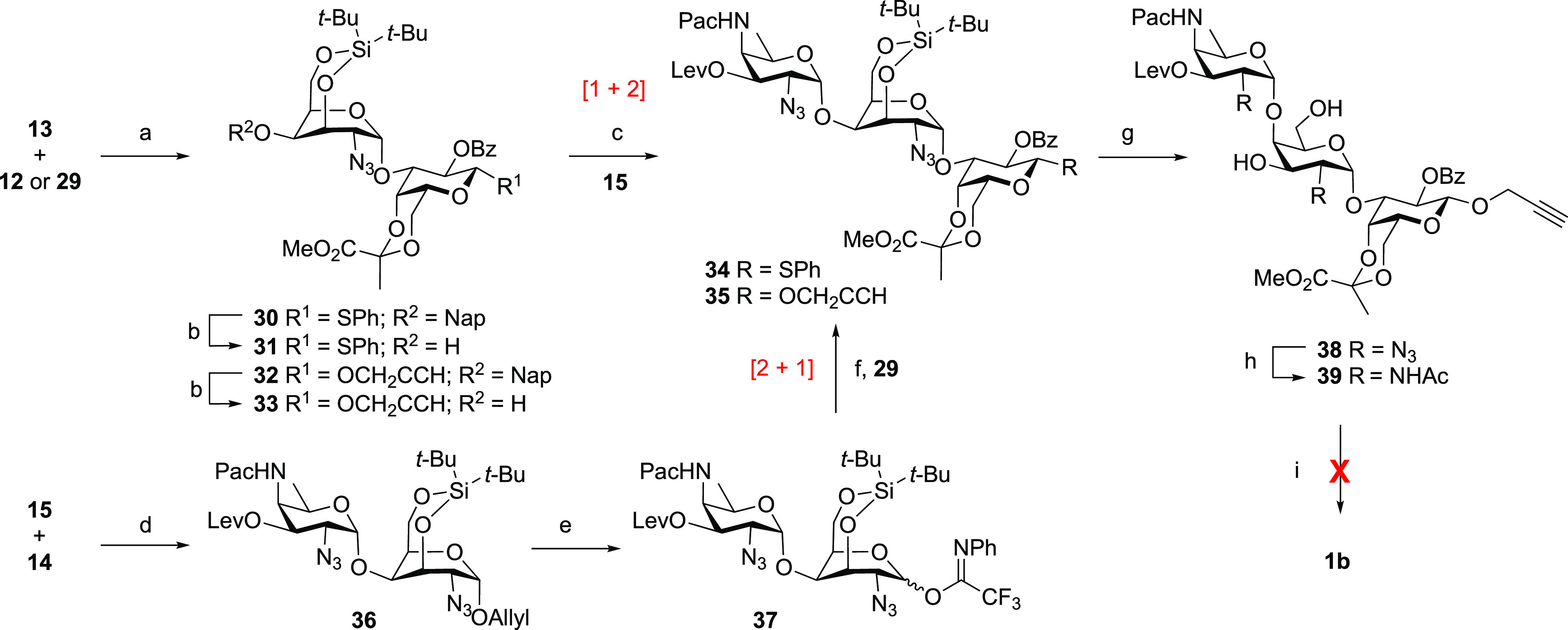
Initial Attempt at the Assembly of the PS A1 Oligosaccharides Reagents and conditions: (a) TfOH, DCM, 0 °C, 4 Å MS, **30**, 75%; **32**, 78%; (b) DDQ, DCM, water, **31**, 94%; **33**, 89%; (c) TBSOTf, DCM, 0 °C, 4 Å MS, **34**, 34%; **35**, 21%; (d) TfOH, DCM, 0 °C, 4 Å MS, 95%; (e) (i) (Ir(COD)(Ph_2_MeP)_2_·PF_6_), H_2_, THF, then NIS, water, quant.; (ii) *N*-phenyltrifluoroacetimidoyl chloride, Cs_2_CO_3_, acetone, 94%; (f) TBSOTf, DCM, 4 Å MS, **35**, 72%; (g) TBAF, AcOH, THF, 99%; (h) (i) 1,3-propanedithiol, Py, Et_3_N, water; (ii) Ac_2_O, THF, water, NaHCO_3_, 54%; (i) NaOH, water or NH_3_-H_2_O. TBSOTf = *tert*-butyldimethylsilyl trifluoromethanesulfonate.

With the building blocks in hand, we set out to assemble the set of target PS A1 oligomers. As depicted in Scheme 3, we first explored the glycosylation of silylidene donor **13** with galactosyl acceptors **12** and **29**. The chemoselective glycosylation between imidate donor **13** and thioglycoside acceptor **12** proceeded in a completely stereoselective manner to provide disaccharide **30** in a 75% yield. The condensation of **13** with propargyl galactoside acceptor **29** proceeded in a similar fashion to give the propargyl disaccharide **32** in a 78% yield. Removal of the naphthylmethyl ethers in **30** and **32** then delivered disaccharide acceptors **31** and **33**, respectively. Unfortunately, the glycosylation of thiophenyl disaccharide **31** and AAT donor **15** led to a complex mixture, and despite significant optimization attempts, the target trisaccharide **34** could not be obtained in more than 34% yield. Although the desired product was formed in a stereoselective manner, TLC-MS and NMR indicated the formation of several side products as a result of aglycon transfer and donor hydrolysis events. We therefore switched to the use of propargyl acceptor **33**, but this did not lead to an improved outcome. The use of other AAT donor types (including the thioglycoside, sulfoxide, lactol, and propargylbenzoate; see the Supporting Information) was to no avail. As the [1 + 2] glycosylation reaction sequence proved unproductive, we next explored a [2 + 1] approach and generated allyl disaccharide **36** from AAT donor **15** and silylidene acceptor **14**. This glycosylation, catalyzed by triflic acid (TfOH), proceeded uneventfully to provide the desired disaccharide **36** in a 95% yield as a single anomer. This latter glycosylation shows that the equatorial C-4-OH in **14** is an apt nucleophile. The anomeric allyl group was removed by isomerization to the corresponding enol ether and treatment with NIS. The disaccharide was isolated as a mixture of the lactol and ring-opened aldehyde in a ± 4:6 ratio. Installation of the *N*-phenyl trifluoroacetimidate functionality delivered disaccharide donor **37** in a 94% yield over the two steps. In the ensuing [2 + 1] glycosylation, the disaccharide donor **37** and propargyl galactoside **29** were stereoselectively united to give the trisaccharide **35** in a 72% yield.^35^ With this building block in hand, we decided to explore the planned basic deprotection chemistry. First, the silylidene ketal was removed to provide diol **38** in near-quantitative yield. The azide to acetamide transformation was affected by the subsequent treatment of **38** with 1,3-propanedithiol and acetic anhydride to deliver trisaccharide **39** in a 54% yield. Unfortunately, basic deprotection of this trisaccharide proved challenging. Especially, the phenoxyacetyl group was difficult to selectively remove, even though a reaction on a model AAT monosaccharide had shown that this group could be readily cleaved using basic hydrolysis conditions. The harsh reaction conditions required to remove the Pac group from **39** led to substantial acetamide cleavage, and we therefore had to abandon the Pac-based synthesis route.

### Second-Generation Synthesis

**Scheme 4:**
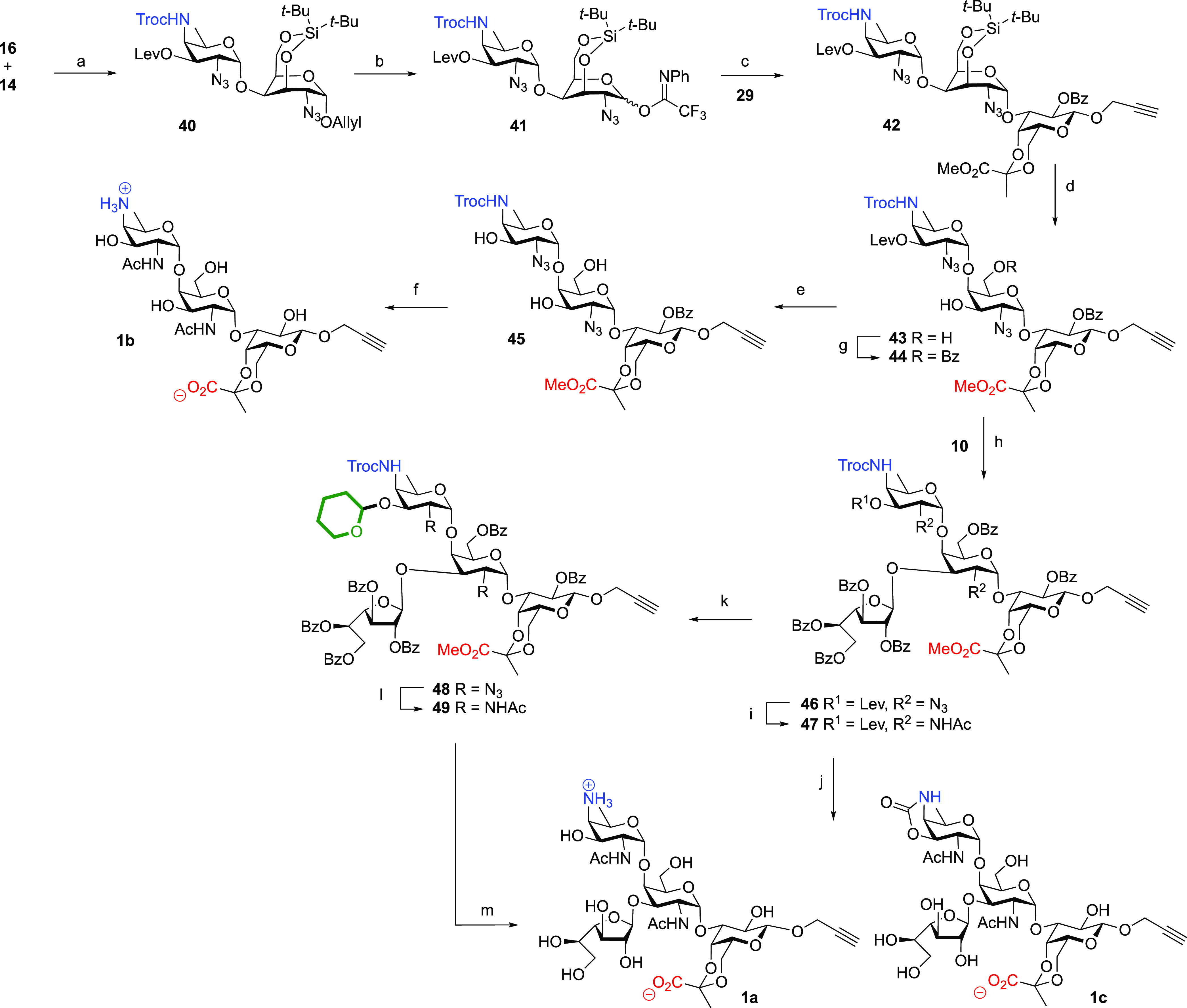
Successful Synthesis of Trisaccharide **1b** and Tetrasaccharide **1a** Reagents and conditions: (a) TfOH, DCM, 4 Å MS, 0 °C, 89%; (b) (i) (Ir(COD)(Ph_2_MeP)_2_·PF_6_), H_2_, THF, then NIS, water, 97%; (ii) *N*-phenyltrifluoroacetimidoyl chloride, Cs_2_CO_3_, acetone, 82%; (c) TBSOTf, DCM, 4Å MS, 84%; (d) HF/Py, THF, Py, 97%; (e) N_2_H_4_·AcOH, AcOH, Py, 0 °C, 78%; (f) (i) AcSH, Py, 73%; (ii) NaOH, dioxane, THF, water, 57%; (g) BzOBt, Et_3_N, DCM, 91%; (h) TBSOTf, DCM, 4 Å MS, 73%; (i) (i) N_2_H_4_·AcOH, AcOH, Py, 0 °C, 80%; (ii) AcSH, Py, 40%; (j) NaOH, dioxane, THF, H_2_O, **1a**, 23%; **1c**, 53%; (k) (i) N_2_H_4_·AcOH, AcOH, Py, 0 °C, 80%; (ii) 3,4-dihydropyran, PPTS, DCM, 76%; (l) (i) PPh_3_, THF, Py, water; (ii) Ac_2_O, THF, water, NaHCO_3_, 83%; (m) (i) LiOH, dioxane, THF, water, then 1 M HCl, AcOH, 50 °C, 89%. BzOBt = benzoyl hydroxybenzotriazole, AcSH = thioacetic acid, PPTS = pyridinium *p*-toluenesulfonate.

Taking lessons from our first assembly approach, we then moved to the use of the Troc-protected AAT building block. Although Troc-groups are generally removed by treatment with zinc, there is also precedent for the removal of these carbamates under basic conditions.^36,37^ The assembly of the set of target PS A1 oligomers using the Troc-based approach is depicted in Scheme 4 and started with the union of Troc-protected AAT donor and silylidene galactosazide acceptor **14** to deliver disaccharide **40** in an 89% yield as a single anomer. Transformation of **40** into imidate donor **41** was achieved as described above to give the dimer donor in a 79% yield over two steps. Extension of this dimer with propargyl galactoside **29** proceeded with complete stereoselectivity under the agency of TBSOTf to give trisaccharide **42** in an 84% yield. Deprotection of this trisaccharide was then affected by the removal of the silylidene ketal and levulinoyl ester. Next, the azides were transformed into the corresponding acetamides using thioacetic acid to set the stage for the crucial global deprotection event. Gratifyingly, the treatment of the so-formed trisaccharide with NaOH effectively unmasked the pyruvate carboxylate, the galactosyl C-2-OH, and the AAT C-4-amine to deliver the first trisaccharide target **1b** in a 57% yield. It was observed that a minor amount of the AAT cyclic 3,4-carbonate was formed, a side reaction that has often been observed during deprotection of carbamate-protected AAT synthons.^21,25,38^

With adequate deprotection conditions established, we next set out to introduce the galactofuranosyl branch on the trisaccharide backbone. To this end, we regioselectively masked the primary alcohol in **43** with a benzoate using benzoyl hydroxybenzotriazole (BzOBt) as a mild and selective benzoylating agent.^39^ Furanosylation of the remaining C-3-OH with building block **10** and TBSOTf as an activator then delivered fully protected tetrasaccharide **46** in 73%. The deprotection of this tetrasaccharide was done in a similar manner as described for the deprotection of trisaccharide **43**. Thus, the removal of the AAT C-4-*O*-levulinoyl ester was followed by the introduction of the acetamides to give **47**. Unfortunately, global deprotection of this tetrasaccharide using NaOH provided the target tetrasaccharide in only a 23% yield, with cyclic carbamate **1c** being formed in a 53% yield. We therefore decided to block the AAT C-3-hydroxyl before global basic deprotection. We initially tried to introduce a silyl ether at this position, but the hydroxyl group proved to be reluctant to silylation even under forceful silylation conditions (TBSOTf, DiPEA). We next explored the use of a tetrahydropyranyl group for protection. Introduction of this group can be achieved under relatively mild conditions through the generation of a reactive tetrahydropyranosyl oxocarbenium ion and indeed the AAT C-3-OH released from the tetrasaccharide **46** could be effectively protected by treatment with 3,4-dihydropyran and pyridinium *para*-toluenesulfonate (PPTS) to give **48**. Next, the azides were reduced under basic Staudinger conditions after which the liberated amines were acetylated to provide tetrasaccharide **49** in an 83% yield. This set the stage for the global basic deprotection, which now proceeded uneventfully to liberate all functional groups except the AAT C-3-OH, which was finally deprotected by treatment with aqueous acetic acid to give tetrasaccharide **1a** in 57% over the last 5 steps.

**Scheme 5:**
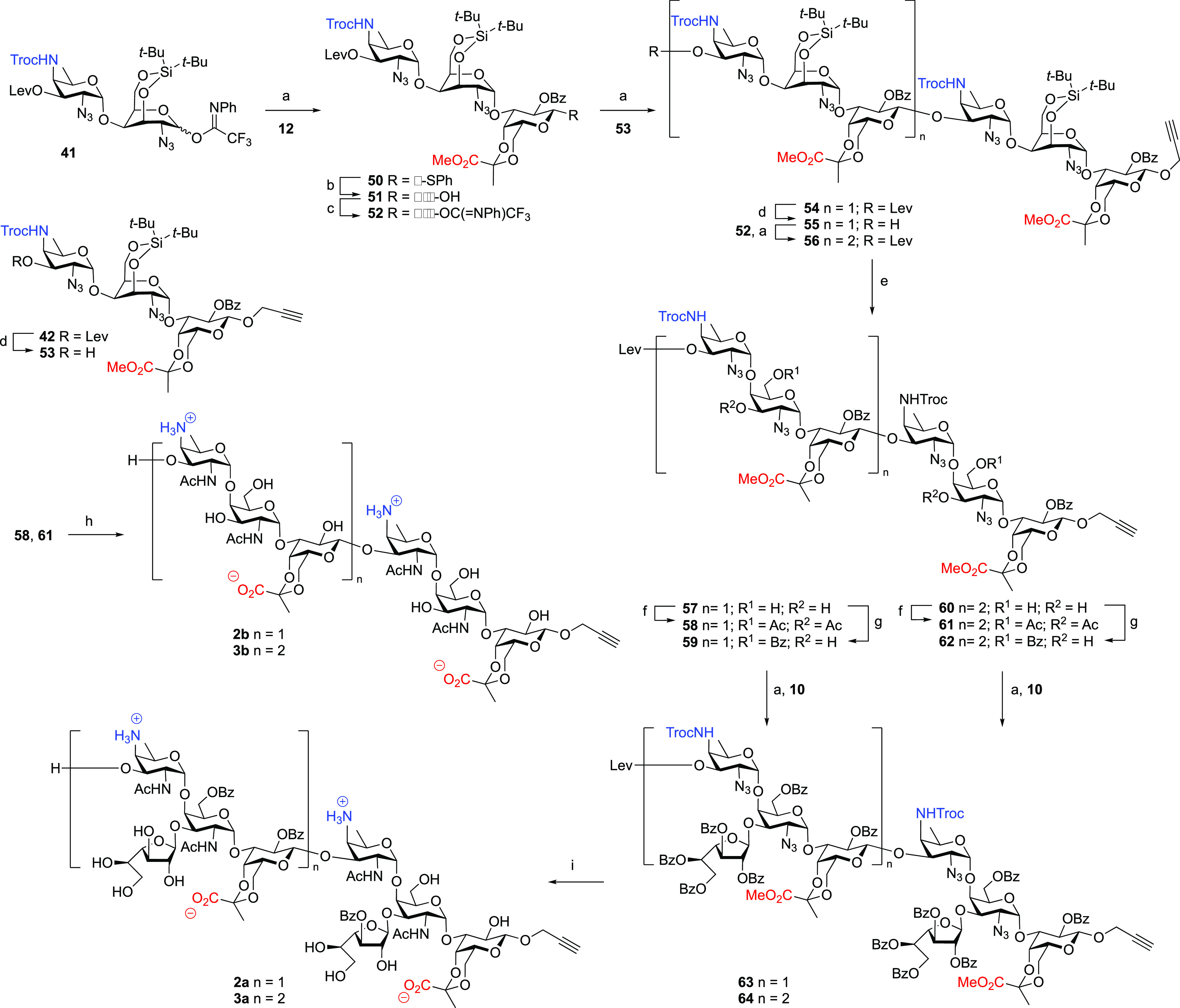
Assembly of Hexamer **2b**, Octamer **2a**, Nonamer **3b**, and Dodecamer **3a** Reagents and conditions: (a) TBSOTf, DCM, 0 °C, 4 Å MS, **50**, 82%; **54**, 79%; **56**, 86%; **63**, 72%; **64**, 85%; (b) NIS, TFA, DCM, 0 °C, quant.; (c) *N*-phenyltrifluoroacetimidoyl chloride, Cs_2_CO_3_, acetone, quant.; (d) N_2_H_4_·AcOH, AcOH, Py, 0 °C, **53**, 97%; **55**, 96%; (e) HF·Py, THF, Py, 0 °C, **57**, 92%; (f) (i) Ac_2_O, Py; (ii) N_2_H_4_·AcOH, AcOH, Py, 0 °C, **58**, 88%; **61**, 85% (3 steps); (g) BzOBt, Et_3_N, DCM, **59**, 69%; **62**, 84% (2 steps); (h) (i) 3,4-dihydropyran, PPTS, DCM; (ii) PPh_3_, THF, Py, water, 70 °C; (iii) Ac_2_O, THF, water, NaHCO_3_; (iv) LiOH, dioxane, THF, water; (v) 1 M HCl, AcOH, 50 °C, **2b**, 57%; **3b**, 49%; (i) (i) N_2_H_4_·AcOH, AcOH, Py, 0 °C; (ii) 3,4-dihydropyran, PPTS, DCM; (iii) PPh_3_, THF, Py, water, 70 °C; (iv) Ac_2_O, THF, water, NaHCO_3_; (v) LiOH, dioxane, THF, water; (vi) 1 M HCl, AcOH, 50 °C, **2a**, 65%; **3a**, 50%.

With methods established to effectively generate the key trisaccharide building block and deprotect the final compounds, we set out to assemble the larger target structures as depicted in Scheme 5. First, the trisaccharide donor and acceptor building blocks were generated. For the assembly of the former, disaccharide **41** was coupled with thioglycoside **12** to give trisaccharide **50** in a fully stereoselective manner in an 82% yield. The thiophenol group was then exchanged for an imidate by hydrolysis of the thioacetal using NIS/TFA,^40^ and the reaction of the liberated lactol **51** with the imidoyl chloride gave trisaccharide donor **52**. The trisaccharide acceptor **53** was generated by delevulinoylation of **42** using hydrazine acetate. The crucial [3 + 3] glycosylation was achieved by the activation of donor **52** with TBSOTf to stereoselectively provide hexasaccharide **54** in a 79% yield. Delevulinoylation then provided the hexasaccharide acceptor **55**, which in the ensuing [3 + 6] glycosylation with another copy of **52** provided nonasaccharide **56** in an 86% yield. In line with the deprotection and functionalization chemistry described above, the silylidene ketals of the hexa- and nonasaccharides were removed after which the primary alcohols were regioselectively benzoylated (**57** to **59** and **60** to **62**). Besides, the liberated alcohols in **57** and **60** were acetylated, after which removal of the levulinoyl esters of the AAT sugar provided **58** from **57** and **61** from **60**, respectively. The protected hexa- and nonamers **58** and **61** were brought to an end by installing the THP ethers, reduction of the azides using basic Staudinger conditions, acetylation of the so-liberated amines, and finally global basic deprotection and mild acidic THP cleavage. This sequence of reactions provided the hexasaccharide **2b** and nonasaccharide **3b** in 57 and 49% yield respectively from **58** and **61**. To deliver the PS A1 octa- and dodecasaccharides, hexasaccharide diol **59** and nonasaccharide triol **62** were glycosylated with an excess of galactofuranosyl donor **10** (three equivalents per hydroxy group) to stereoselectively give the target octasaccharide **62** and dodecasaccharide **63** in 72 and 85% yields, respectively. Following the now well-established deprotection sequence (Lev removal, THP installation, azide to acetamide transformation, basic deprotection, and AcOH mediated THP removal) completed the total synthesis of the final two target compounds **2a** and **3a** in 65 and 50% over six steps, respectively. With the chemistry developed, we were able to generate more than 50 mg of dodecasaccharide **3a**, showing the applicability of the established methodology.

### Structural Studies

Having the synthetic oligomers available, we set out to investigate their secondary structure using a combination of NMR and MD protocols. All ^1^H and ^13^C NMR resonances were assigned through standard TOCSY, NOESY, and HSQC experiments. The analysis of the intraresidues NOE and *J*-couplings established that the three pyranosides within the RUs adopt a ^4^C_1_ conformation, independent of the presence of the Gal*f*-appendages or the length of the oligosaccharide. A high degree of flexibility was observed for the Gal*f*-residues. Furanose rings can adopt a variety of envelope (E) and twist (T) conformations of similar energy that interconvert through a process known as pseudorotation.^41^ The dynamic structure of the furanose ring makes its conformational analysis challenging. Yet, the 1D NMR spectrum of **1a** shows well-separated resonances, making the analysis of the ^3^J_H,H_-coupling constants straightforward (Figure S2). In parallel, theoretical ^3^*J*_H,H_-coupling constants for the Gal*f*-β-OMe, as a model, were calculated using Altona equations^42^ with MSPIN^43^ for an ensemble of conformations along the pseudorotational itinerary. The experimentally determined coupling constants ^3^*J*_H1,H2_, ^3^*J*_H2,H3_, and ^3^*J*_H3,H4_ were used to define the solution-state ring conformations. Comparison of calculated- and NMR-derived ^3^*J*_HH_-coupling constants showed that in **1a** the puckering of the five-membered ring can be described by an equilibrium ensemble in the region defined by ^4^T_3_, ^4^T_0_, and ^4^E conformers (see the Supporting Information, Table S1 and Figure S3). In all of these three conformers, the ethylene glycol substituent at C-4 is in a favorable pseudoequatorial orientation. The experimentally determined large ^3^*J*_H3,H4_ and the small ^3^*J*_H2,H3_ and ^3^*J*_H1,H2_ represent equilibrium values among these three discrete conformations. The puckering analysis of the Gal*f* rings in compounds **2a** and **3a** indicated the same behavior as found for **1a**.

**Figure 2:**
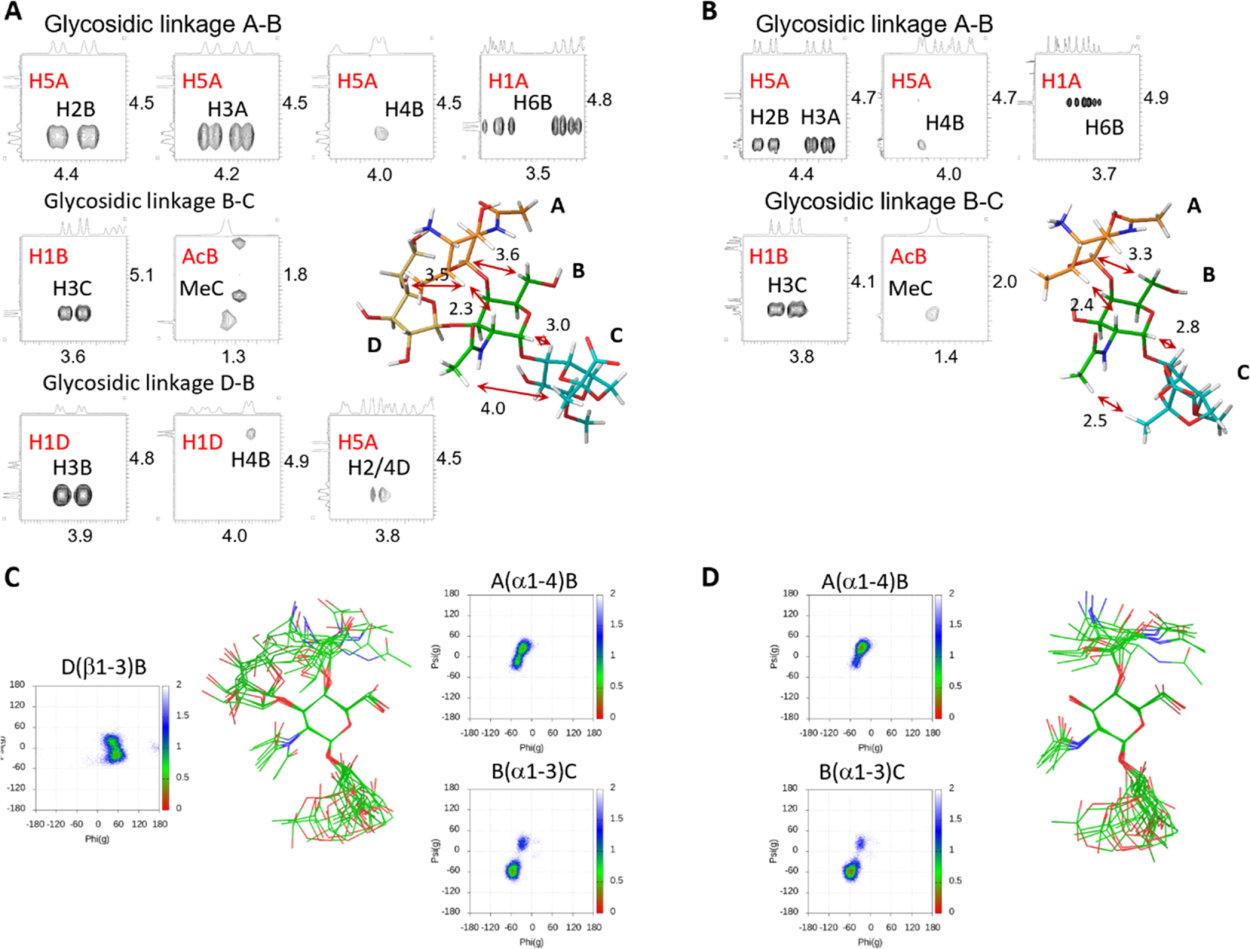
Analysis of the torsion angles around the glycosidic linkages in **1a** and **1b**. (A, B) 2D sections of the NOE correlations defining the main conformation around the glycosidic linkages of **1a** (A) and **1b** (B). (C, D) Eight snapshot superimpositions and Φ/Ψ maps of the conformations explored along the MD simulation for compounds **1a** (C) and **1b** (D).

Next, the conformation around the glycosidic linkages for compound **1a** was scrutinized. The NOESY spectra showed key inter-residue cross-peaks, which allowed us to unequivocally define the relative orientation of adjacent monosaccharides. The NOEs observed for the H5A-H2B, H5A-4B, and H1A-H6B proton pairs are indicative of a conformational equilibrium between the *exo-syn-*Φ/*syn*(+)-Ψ and *exo-syn-*Φ/*syn*(−)-Ψ conformations around the A–B glycosidic linkage (see Figure 2A). Comparison of the relative intensity of the inter- and intra-residue NOEs indicated that the *exo-syn-*Φ/*syn*(+)-Ψ geometry is the major conformation. Fittingly, MD simulations also predicted the *syn*(+)-Ψ as the most populated conformer, although transitions between *syn*(−)-Ψ and *syn*(+)-Ψ conformers were observed along the simulation (Figure 2C). In comparison, the B–C linkage was more restricted. Both the NOE-estimated distances and the MD simulation indicated the *exo-syn-*Φ/*syn*(−)*-*Ψ as the most representative conformation. The key NOEs between the H1B-H3C and methyl protons of the B2-acetamide group and those of the pyruvate moiety of unit C unequivocally defined the torsion angle (Figure 2A). Inspection of the 3D model structures revealed that for the B–C torsion angle, the *exo-syn-*Φ/*syn*(+)*-*Ψ conformation is prevented due to a steric clash between the hydroxymethylene group of the GalNAc (residue B) and the pyruvate moiety of C. Regarding the Gal*f-*GalNAc glycosidic linkage, the H1D-H3B, H1D-H4B, and the H5A-H2/4D proton pairs indicated an equilibrium between the *exo-syn-*Φ/*syn*(+)-Ψ and *exo-syn-*Φ/*syn*(−)-Ψ conformations around the B–D glycosidic linkage (Figure 2A,C).

A similar protocol was applied to **1b**, which does not carry the Gal*f*-residue linked at position 3 of the GalNAc residue B. The analysis of the key inter-residues NOEs and their relative intensity demonstrated that the presence of the galactofuranoside moiety does not perturb the overall 3D structure (Figure 2B). In fact, the H5A-H2B and H5A-H4B NOEs, in the presence or absence of the Gal*f*, were found to be very similar. The MD simulation further supported this result (Figures 2D and S7). From these studies, we concluded that the Gal*f* ring does not influence the conformational distribution nor the dynamics of the short oligosaccharides.

**Figure 3:**
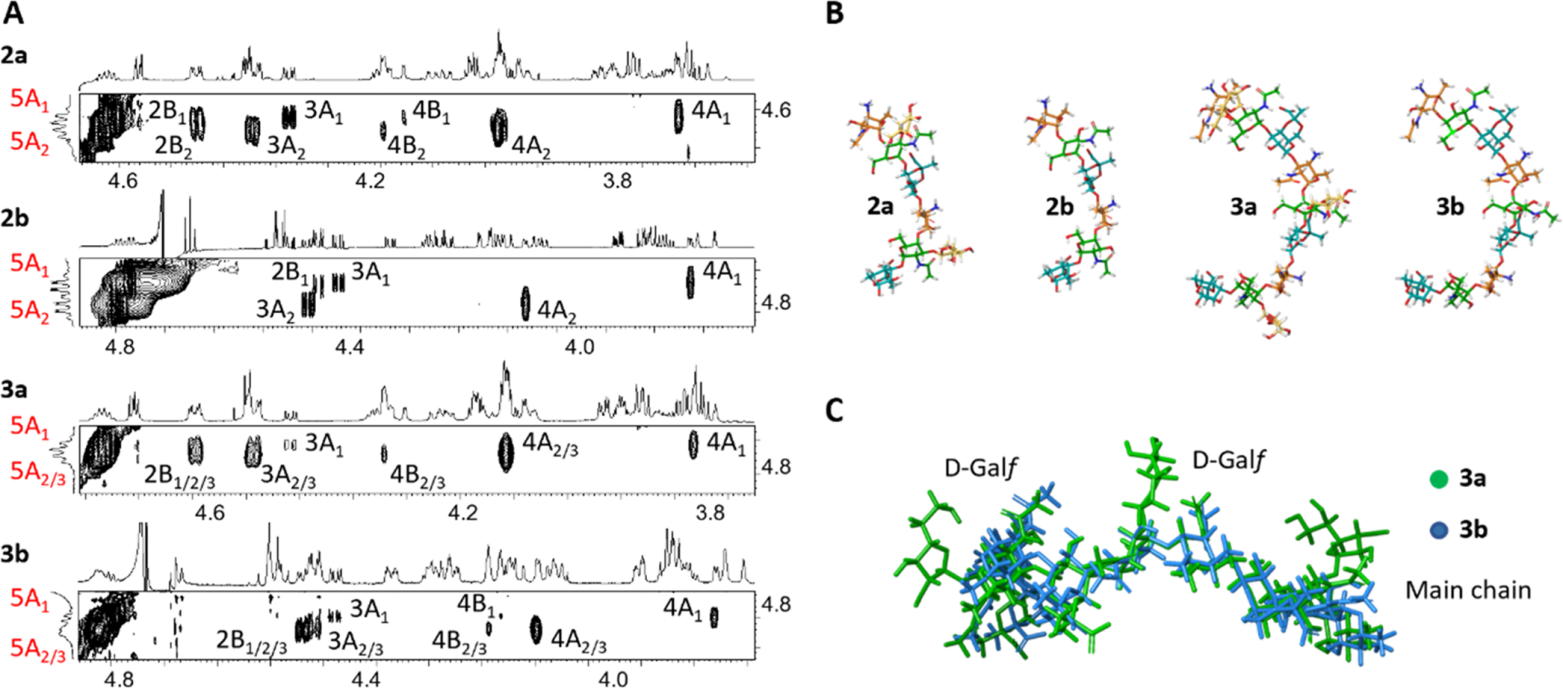
Conformational analysis of the longer oligosaccharides **2** and **3**. (A) Strips of two-dimensional NOESY spectra of molecules **2a/b**–**3a/b** taken at a frequency of H5A. (B) Main conformation of molecules **2a/b** and **3a/b** as defined by NOE analysis. (C) Superposition of the 3D structures of **3a** and **3b**, showing the orientation of the d-Gal*f* rings in **3a** (in green).

We next extended the structural analysis to the larger molecules. NOESY experiments, combined with MD simulations, indicated fairly similar conformational features for the larger oligosaccharides **2a/b** and **3a/b** as found for the shortest analogues (Figure 3). Briefly, inter-residue NOEs defined *exo-syn-*Φ/*syn*(+)-Ψ and *exo-syn-*Φ/*syn*(−)-Ψ conformations around the A–B, and the *exo-syn-*Φ/*syn*(−)-Ψ conformation around the B–C bonds and the new C*_x_*-A_(*x*+1)_ glycosidic linkages (see Figure 3A for excerpts of the spectra). As in the monomeric RU, the B–D glycosidic linkage is defined by an equilibrium between the *exo-syn-*Φ/*syn*(+)-Ψ and *exo-syn-*Φ/*syn*(−)-Ψ conformations. Overall, this results in the Gal*f*-residues pointing in the same direction, outwards with respect to the oligosaccharide main chain (Figure 3C). The addition of repeating units does not alter the flexibility around the glycosidic linkages (Figure S8).

**Figure 4:**
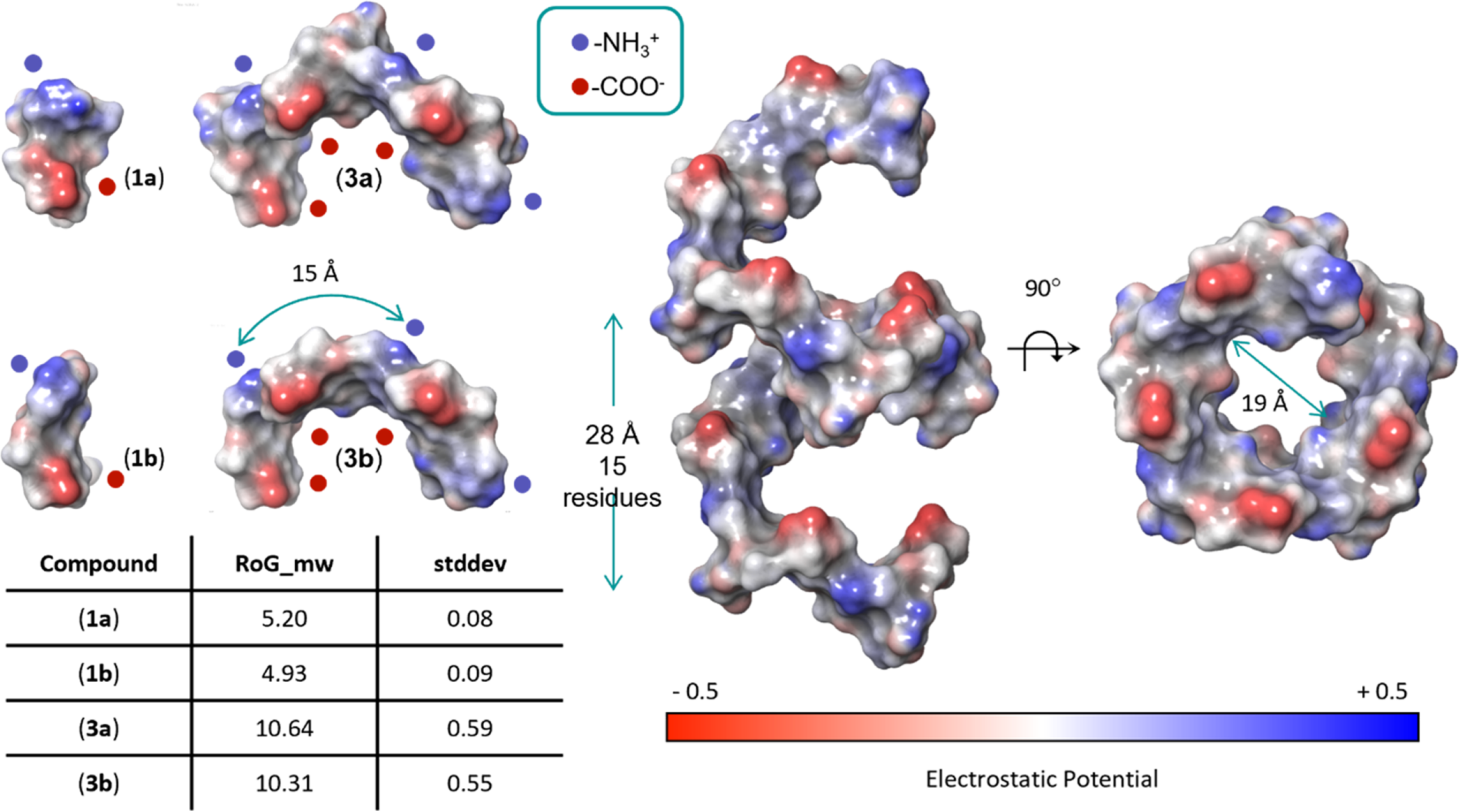
Surface molecular models of **1a/b** and **3a/b** PS A1 oligosaccharide compounds and of a modeled oligosaccharide made of 9 RUs (a 27-monomer long backbone carrying 9 Gal*f*-residues). The table reports the average RoG values and relative standard deviation for each compound.

Notably, when we examined the radius of gyration (RoG), defined as the root-mean-square distance of the collection of atoms from their common center of gravity, to establish the overall extension of the molecules, it became apparent that the PS A1 oligosaccharides do not extend in a linear manner. Instead, the longer oligosaccharides adopt a bent structure and the RoG of the nonasaccharide **3a** is only twice that calculated for trisaccharide **1a**. Interestingly, along the arch of the longer oligomers, the positively charged amine groups point outwards, while the negatively charged pyruvate carboxylates point inward (Figure 4).

The major conformers determined for **3a/b** suggest that longer oligo- and polysaccharides can adopt a well-organized structure, and to predict the 3D structure of larger fragments we modeled an oligosaccharide made of 9 RUs (see Figure 4). This molecule adopts a left-handed helical structure, with 15 residues per turn (5 RUs, ca. 28 Å) and a cavity having a diameter of ca. 19 Å. Along the helix, the negatively charged groups are positioned parallel to the axis of the helix, while the positively charged amines are placed perpendicular to the axis. The distance between adjacent positive charges is ca. 15 Å, while that in between the negatively charged groups is slightly shorter (ca. 14 Å). The Gal*f*-residues are all solvent-exposed. Important differences become apparent when this PS A1 structure is compared to the helices of the zwitterionic Sp1 and PS A2 polysaccharides. While the latter two ZPSs adopt a right-handed helix, the helix formed by PS A1 is left-handed. In addition, the radius of the PS A1 helix is significantly larger than that of the other two zwitterionic polysaccharides, requiring 15 monosaccharides to complete a turn, while only 8 monosaccharides are required to complete a turn in the Sp1 and PS A2 helices. It has been postulated that the spatial arrangement of the positively charged groups along the Sp1 and PS A2 helices enables the unique interaction of these polysaccharides with T cells. Fittingly, and in spite of the significantly different secondary structure of the PS A1 relative to PS A2 and Sp1 polysaccharides, the distance in between the adjacent amines is ca. 15 Å in all three cases. Furthermore, in all three ZPSs, the positively charged groups point outward. These similar patterns suggest that different 3D structures can be accepted to elicit a T-cell response as long as the spatial arrangement of the positive groups is preserved. The synthetic oligosaccharides described herein—alongside the helical Sp1 oligomers that we previously generated—will be valuable tools to further unravel the mode of interaction of these saccharides and their designated binding partners at the atomic level.

## Conclusions

In conclusion, we have here described the first total synthesis of PS A1 oligosaccharides comprising up to three repeating units. Key to the successful syntheses was the use of a C-3,C-6-bridged silylidene-protected galactosamine building block, having an “inverted” conformation that places the C-4-OH in an equatorial orientation, turning it into an apt nucleophile. In addition, the silylidene bridge effectively shields the top face of the galactosamine building block, thereby enabling highly diastereoselective glycosylation reactions when using the synthon as a glycosyl donor. To further streamline the syntheses, we developed a protecting group strategy that hinges on the use of base-labile protecting groups, which has allowed us to install an alkyne linker at the reducing end of the oligomers. This functionality can be readily exploited to functionalize the oligosaccharides with, for example, a fluorophore or photoaffinity probe or attach them to a carrier protein or antigenic peptide to create innovative vaccine modalities. The linker can also be used to immobilize the oligomers to microarray or surface plasmon resonance chip surfaces to enable biophysical interaction studies. Our structural studies have shown the PS A1 to adopt a left-handed helical structure, which differs significantly from the secondary structures adopted by other zwitterionic polysaccharides. It does, however, place the positively charged amino groups at the periphery of the helix with a mutual distance of 15 Å, a key structural feature that is encountered both in the Sp1 and in the PS A2 helices. It will be exciting to see how the PS A1 structure interacts with binding proteins, and the structural studies presented here will present an ideal stepping stone to embark on these studies, revealing the molecular basis of the unique immunomodulatory behavior of these unique structures.

## Supporting Information Available

Experimental procedures, characterization data, NMR spectra, and structural characterization data (PDF)
