# Supplementary material for: Total Synthesis and Structural Studies of Zwitterionic Bacteroides fragilis Polysaccharide A1 Fragments
Source: J Am Chem Soc. 2023 Jun 13;145(25):14052–63. doi: 10.1021/jacs.3c03976 (PMC10311536; doi:10.1021/jacs.3c03976)

Supporting Information

Total synthesis and structural studies of zwitterionic *Bacteroides fragilis* polysaccharide A1 fragments

Zhen Wang,[1,3] Ana Poveda,[2] Qingju Zhang,[1,3] Luca Unione,[2,4] Herman S. Overkleeft,[1] Gijsbert A. van der Marel,[1] Jesús Jiménez-Barbero,[2,4,5,6] Jeroen D. C. Codée*,[1]

[1] Leiden Institute of Chemistry, Leiden University, Einsteinweg 55, 2333 CC Leiden, The Netherlands

[2] CICbioGUNE, Basque Research & Technology Alliance (BRTA), Bizkaia Technology Park, Building 800, 48162 Derio, Bizkaia, Spain

[3] National Research Centre for Carbohydrate Synthesis, Jiangxi Normal University, 99 Ziyang Avenue, Nanchang, 330022 China

[4] Ikerbasque, Basque Foundation for Science, Maria Diaz de Haro 3, 48013 Bilbao, Bizkaia, Spain

[5] Department of Organic Chemistry, II Faculty of Science and Technology University of the Basque Country, EHU-UPV, 48940, Leioa, Spain

[6] Centro de Investigación Biomédica En Red de Enfermedades Respiratorias (CIBERES), 28029, Madrid, Spain

Corresponding author email: jcodee@chem.leidenuniv.nl

General experimental procedures

All reagents were of commercial grade and used as received. All moisture sensitive reactions were performed under an argon or nitrogen atmosphere, at ambient temperature, unless stated otherwise. DCM used in the glycosylation reactions was dried with flamed 4Å or 5Å molecular sieves before being used. Reactions were monitored by TLC analysis with detection by UV (Merck, silica gel 60, F245) with detection by UV absorption (254 nm) and where applicable by spraying with 20% sulfuric acid in EtOH or with a solution of $(NH_4)_6Mo_7O_{24}·4H_2O$ (25 g/L) and $(NH_4)_4Ce(SO_4)_4·2H_2O$ (10 g/L) in 10% sulfuric acid (aq.) followed by charring at ~150 °C. Column chromatography was performed on silica gel (40-63μm). ^{1}H and ^{13}C spectra were recorded on a Bruker AV 400 or Bruker AV 500 or Bruker AV 600 and Bruker AV 850. Chemical shifts (δ) are given in ppm relative to tetramethylsilane as internal standard (^{1}H NMR in $CDCl_3$) or the residual signal of the deuterated solvent. Coupling constants (*J*) are given in Hz. All ^{13}C spectra are proton decoupled. NMR peak assignments were made using COSY and HSQC experiments, where applicable Clean TOCSY, HMBC and GATED experiments were used to further elucidate the structure. The anomeric product ratios were analyzed through integration of proton NMR signals. High-resolution mass (HRMS) was performed on a Thermo Finnigan LTQ Orbitrap mass spectrometer equipped with an electrospray ion source in positive ion mode (source voltage 3.5 kV, sheath gas flow 10, capillary temperature 275 °C) resolution R = 60.000 at m/z 400 (mass range of 150–4000) and dioctylphthalate(m/z = 391.28428) as lock mass, or on a Waters Spynat G2-Si(OTF) equipped with an electrospray ion source in positive mode (source voltage 3.5 kV) and LeuEnk (m/z = 556.2771). Optical rotation measurements ($[\alpha]_D^{20}$) were performed on an Anton Paar Modular Circular Polarimeter MCP 100/150 with a concentration of 10 mg/mL (c 1), unless stated otherwise.

Experimental Procedures and Characterization Data of Products

Phenyl 2-azido-6-deoxy-4-*N*-phenoxyacetimide-1-thio-β-D-galactopyranoside (18)

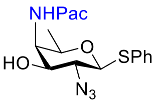

Phenyl 6-deoxy-3-*O*-triisopropylsilyl-1-thio-β-D-mannopyranoside **17**[1] (7.9 g, 19.2 mmol, 1.0 eq) was dissolved in DCM (170 ml) and pyridine (20 mL, 250 mmol, 13.0 eq), DMAP (2.35 g, 19.2 mmol, 1.0 eq), and then Tf_2O (19.3 mL, 115.2 mmol, 6.0 eq) was added to the reaction mixture at -10 ℃, and slowly warm up to 10 ℃ in 2 h. After TLC showed complete consumption of the starting material, the reaction mixture was diluted with DCM and washed with 1M HCl solution and saturated aqueous sodium bicarbonate. The organic layer was dried over Na_2SO_4 and concentrated *in vacuo*. The residue was dissolved in dry CH_3CN (250 mL), a $TBAN_3$ (5.56 g, 19.6 mmol, 1.02 eq) solution in

CH_3CN (25 mL) was slowly added to the reaction mixture at -30 °C and stirred for a day. The reaction was warmed slowly to -20°C and stirred for 2 additional days. After TLC showed complete consumption of the starting material, 7N NH_3 in methanol (40 mL) was added at -20°C. The reaction was slowly warmed to 5 °C and stirred 3 days. After TLC showed complete consumption of the starting material, the mixture was concentrated *in vacuo*. The residue was dissolved in THF (190 mL) and water (95 mL), and then sodium bicarbonate (6.5 g, 76.8 mmol, 4.0 eq) was added and cooled to 0 °C. After phenoxyacetyl chloride (PacCl) (5.3 mL, 38.4 mmol, 2.0 eq) was added the mixture was stirred overnight at room temperature. After TLC showed complete consumption of the starting material, the reaction was quenched with saturated aqueous sodium bicarbonate and diluted with EtOAc. The solution was washed with water (2x) and brine. The aqueous layer was extracted with EtOAc (3x), dried with $MgSO_4$, filtered, and concentrated *in vacuo*. The crude was dissolved in THF (190 mL) and AcOH (2.2 mL, 38.4 mmol, 2 eq). Then 1M TBAF in THF (39 mL, 39 mmol, 2 eq) was added at 0 °C. The reaction mixture was stirred overnight. After TLC showed complete consumption of the starting material, the reaction was quenched with saturated aqueous ammonium chloride and diluted with EtOAc. The solution was washed with water (2x) and brine. The aqueous layer was extracted with EtOAc (3x), dried with $MgSO_4$, filtered, and concentrated *in vacuo*. The compound was purified by flash chromatography (PE/EA/DCM 5:1:1 - 2:1:1) to yield compound **18** (4.2 g, 10.2 mmol, 53%). ^{1}H NMR (400 MHz, Chloroform-*d*) δ 7.62 – 7.52 (m, 2H), 7.41 – 7.27 (m, 5H), 7.12 – 7.05 (m, 1H), 6.99 – 6.92 (m, 2H), 6.69 (d, *J* = 8.7 Hz, 1H, NH), 4.64 – 4.49 (m, 2H, Pac), 4.38 (d, *J* = 10.2 Hz, 1H, H-1), 4.32 – 4.24 (m, 1H, H-4), 3.84 – 3.72 (m, 2H, H-3, H-5), 3.03 (t, *J* = 9.9 Hz, 1H, H-2), 1.16 (d, *J* = 6.4 Hz, 3H, H-6). ^{13}C NMR (101 MHz, $CDCl_3$) δ 171.14 (Pac), 157.03 (Pac), 133.54, 131.25, 130.03, 129.23, 128.65, 122.67, 115.06, 86.43 (C-1), 74.91 (C-3), 73.64 (C-5), 67.45 (Pac), 62.59 (C-2), 53.32 (C-4), 17.11 (C-6). HR-MS: Calculated for $C_{20}H_{22}N_4O_4S$ [M+H$^+$]: 415.1435, found: 415.1432. $[\alpha]^{20}_D$ = + 5.6° (c = 1, $CHCl_3$). TLC: Rf = 0.5 (PE/EA = 1/1, v/v).

Phenyl 2-azido-6-deoxy-3-*O*-levulinoyl-4-*N*-phenoxyacetimide-1-thio-β-D-galactopyranoside (20)

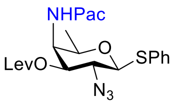

Compound **18** (4.22 g, 10.18 mmol, 1.0 eq) was co-evaporated with anhydrous toluene three times and dissolved in DCM (100 mL). At 0 °C, levulinic acid (3.3 g, 28.4 mmol, 2.8 eq), 1-ethyl-3-(3-dimethylaminopropyl) carbodiimide (EDCI) (3.15 g, 20.4 mmol, 2.0 eq) and 4-dimethylaminopyridine (DMAP) (250 mg, 2 mmol, 0.2 eq) were added. The reaction was stirred overnight. After TLC showed complete

consumption of the starting material, the reaction was diluted with DCM and washed with saturated aqueous sodium bicarbonate and brine. The organic phase was dried with $MgSO_4$, filtered, and concentrated *in vacuo*. The compound was purified by flash chromatography (PE/EA/DCM 7:1:1 – 2:1:1) to yield compound **20** (5.2 g, 10.2 mmol, quant.). ^{1}H NMR (400 MHz, Chloroform-*d*) δ 7.61 – 7.52 (m, 2H), 7.42 – 7.29 (m, 5H), 7.12 – 7.05 (m, 1H), 6.99 – 6.93 (m, 2H), 6.53 (d, *J* = 9.5 Hz, 1H, NH), 4.78 (dd, *J* = 10.2, 3.9 Hz, 1H, H-3), 4.64 – 4.48 (m, 2H), 4.47 – 4.37 (m, 2H, H-4, H-1), 3.86 – 3.76 (m, 1H, H-5), 3.04 (t, *J* = 10.2 Hz, 1H, H-2), 2.90 – 2.45 (m, 4H, Lev), 2.17 (s, 3H, Lev), 1.14 (d, *J* = 6.4 Hz, 3H, H-6). ^{13}C NMR (101 MHz, $CDCl_3$) δ 206.33 (Lev), 171.92 (Lev), 169.19 (Pac), 157.01 (Pac), 133.46, 131.02, 129.98, 129.24, 128.73, 122.59, 114.93, 86.55 (C-1), 74.90 (C-3), 73.58 (C-5), 67.42 (Pac), 59.57 (C-2), 49.86 (C-4), 37.82 (Lev), 29.80 (Lev), 27.86 (Lev), 16.85 (C-6). HR-MS: Calculated for $C_{25}H_{28}N_4O_6S$ [M+Na$^+$]: 535.1622, found: 535.1635. $[\alpha]^{20}_D$ = + 3.5° (c = 1, $CHCl_3$). TLC: Rf = 0.5 (PE/EA = 1/1, v/v).

2-*N*-azido-6-deoxy-3-*O*-levulinoyl-4-*N*-phenoxyacetimide-α/β-D-galactopyranoside (15a)

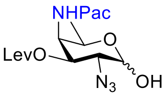

Compound **20** (0.949 g, 1.85 mmol, 1 eq) was dissolved in DCM (10 mL) and cooled to 0 °C. NIS (0.626 g, 2.78 mmol, 1.5 eq) and TFA (0.17 mL, 2.22 mmol, 1.2 eq) were added and the solution was stirred for 1 hour. NIS (0.2 g, 0.925 mmol, 0.5 eq) and TFA (0.01 mL, 1.3 mmol, 0.7 eq) were added and the solution stirred for a another hour. The reaction was quenched with triethyl amine and aqueous sodium thiosulphate. The solution was diluted with DCM and washed with brine (3×). The organic phase was dried with $MgSO_4$, filtered, and concentrated *in vacuo*. The compound was purified by flash chromatography (PE/DCM/EA 3:1:1 - 1:1:1) to yield the titled compound **15a** (0.61 g, 1.46 mmol, 79%). ^{1}H NMR (400 MHz, Chloroform-*d*) δ 7.45 – 7.22 (m, 2H, arom), 7.09 – 6.89 (m, 3H, arom), 6.88 – 6.69 (m, 1H, NH), 6.45 – 5.46 (m, 1H, OH), 5.38 – 5.23 (m, 1H), 4.82 – 3.69 (m, 5H), 3.31 – 3.20 (m, 1H), 2.92 – 2.43 (m, 4H, Lev) , 2.17 (d, *J* = 1.5 Hz, 3H, Lev), 1.09 (d, *J* = 6.3 Hz, 3H, H-6). ^{13}C NMR (101 MHz, $CDCl_3$) δ 207.15, 207.13, 171.95, 169.83, 169.77, 156.78, 156.72, 129.66, 129.60, 129.56, 129.53, 122.23, 122.15, 114.57, 114.48, 114.40, 96.04, 91.60, 72.75, 70.09, 68.59, 66.92, 63.36, 61.97, 58.03, 50.65, 49.83, 37.58, 37.55, 29.50, 27.70, 27.63, 16.26, 16.14. HR-MS: Calculated for $C_{19}H_{24}N_4O_7$ [M+Na$^+$]: 443.1537, found: 443.1538. TLC: Rf = 0.4 (PE/Acetone = 2/1, v/v).

***N*-phenyl-trifluoroacetimidoyl 2-*N*-azido-3-*O*-levulinoyl-5-methyl-4-*N*-phenoxyacetimide-α/β-D-galactopyranoside (15)**

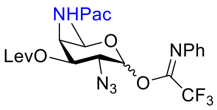

Compound **15a** (0.568 g, 1.35 mmol, 1.0 eq) was dissolved in acetone (13.5 mL) and cooled to 0 °C. *N*-phenyl trifluoroacetimidoyl chloride (0.41 g, 1.98 mmol, 1.47 eq) and cesium carbonate (0.527 g, 1.62 mmol, 1.2 eq) were added. The solution was allowed to warm to RT and stirred for overnight. The reaction was quenched with triethyl amine and concentrated *in vacuo*. The compound was purified by flash chromatography (PE/EA 5:1 - 1:1) to yield compound **15** (0.646 g, 1.09 mmol, 81%). ^{1}H NMR (500 MHz, Acetone-d_6) δ 7.42 – 7.30 (m, 4H), 7.28 – 7.12 (m, 2H), 7.06 – 6.98 (m, 3H), 6.95 – 6.87 (m, 2H), 5.03 – 4.86 (m), 4.76 – 4.60 (m), 4.52 – 4.39 (m), 4.22 – 3.96 (m), 3.87 – 3.71 (m), 2.91 – 2.39 (m, 4H), 2.12 (s, 3H), 1.17 – 1.09 (m, 3H). ^{13}C NMR (126 MHz, Acetone) δ 204.33 (Lev), 171.90 (Lev), 169.56 (Pac), 158.47 (Pac), 143.84, 130.21, 130.19, 129.49, 125.15, 122.24, 122.21, 119.70, 115.38, 115.32, 96.67, 73.06, 71.06, 67.57, 60.86, 50.14, 37.88, 29.31, 28.34, 16.22 (C-6). HR-MS: Calculated for $C_{27}H_{28}F_3N_5O_7$ [M+Na$^+$]: 614.1833, found: 614.1830. TLC: Rf = 0.2 (PE/Acetone = 5/1, v/v).

Phenyl 2-azido-6-deoxy-4-*N*-2,2,2-trichloroethyl-3-*O*-triisopropylsilyl-1-thio-β-D-galactopyranoside (19a)

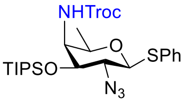

Phenyl 6-deoxy-3-*O*-triisopropylsilyl-1-thio-β-D-mannopyranoside **17** (11.4 g, 27.7 mmol, 1.0 eq) was dissolved in DCM (280 ml) and pyridine (30 mL, 370 mmol, 13.0 eq), and Tf_2O (29.0 mL, 166 mmol, 6.0 eq) were added to the reaction mixture at -10 °C, and the mixture was slowly warmed up to 10 °C in 2 h. After TLC showed complete consumption of the starting material, the reaction mixture was diluted with DCM and washed with 1M HCl solution and saturated aqueous sodium bicarbonate. The organic layer was dried over Na_2SO_4 and concentrated *in vacuo*. The residue was dissolved in dry CH_3CN (300 mL), a $TBAN_3$ (7.5 g, 26.4 mmol, 0.95 eq) solution in CH_3CN (70 mL) was slowly added to the reaction mixture at -30 °C and the mixture was stirred one day. The reaction was warmed slowly to -20°C and stirred for 2 additional days. After TLC showed complete consumption of the starting material, 7N NH_3 in methanol (50 mL) was added in -20°C. The reaction was slowly warmed to 5 °C and stirred 3 days. After TLC showed complete consumption of the starting material, the mixture was concentrated *in vacuo*. After through a flash chromatography, the residue was dissolved in THF (75 mL), and then sodium bicarbonate (2.0 g, 23.8 mmol, 3.0 eq) was added and cooled to 0 °C. After 2,2,2-trichloroethoxycarbonyl chloride (TrocCl) (2.3 mL, 16.7 mmol, 2.0 eq) added, the mixture was stirred for overnight at room temperature. After TLC showed complete consumption of the starting material, the reaction was quenched with saturated aqueous sodium bicarbonate and diluted with EtOAc.

The solution was washed with water (2x) and brine. The aqueous layer was extracted with EtOAc (3x), dried with $MgSO_4$, filtered, and concentrated *in vacuo*. The compound was purified by flash chromatography (PE/EA 50:1 - 30:1) to yield compound **19a** (5.7 g, 9.3 mmol, 33%). ^{1}H NMR (500 MHz, $CDCl_3$) δ 7.64 – 7.55 (m, 2H), 7.42 – 7.32 (m, 3H), 4.96 – 4.87 (m, 2H, NH, Troc), 4.54 – 4.42 (m, 2H, Troc, H-1), 3.99 – 3.92 (m, 1H, H-4), 3.78 (dd, J = 9.5, 4.3 Hz, 1H, H-3), 3.66 – 3.58 (m, 1H, H-5), 3.11 (t, J = 9.8 Hz, 1H, H-2), 1.27 (d, J = 6.3 Hz, 3H, H-6), 1.20 – 1.10 (m, 3H, TIPS), 1.10 – 1.01 (m, 18H, TIPS).^{13}C NMR (126 MHz, $CDCl_3$) δ 155.12 (Troc), 133.63, 133.43, 131.80, 129.33, 128.79, 95.59 (Troc), 87.36 (C-1), 74.78 (Troc), 74.45 (C-5), 73.74 (C-3), 64.52 (C-2), 56.07 (C-4), 18.09 (TIPS), 17.23 (C-6), 12.82 (TIPS). HR-MS: Calculated for $C_{24}H_{37}Cl_3N_4O_4SSi$ [M+H$^+$]: 611.1443, found: 611.1441. $[\alpha]^{20}_D$ = + 2.2° (c = 1, $CHCl_3$). TLC: Rf = 0.5 (PE/EA = 20/1, v/v).

Phenyl 2-azido-6-deoxy-4-*N*-2,2,2-trichloroethyl-1-thio-β-D-galactopyranoside (19)

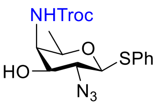

Compound **19a** (4.9 g, 8.0 mmol, 1.0 eq) was dissolved in THF (80 mL) and pyridine (40 mL), then cooled to 0 °C and hydrogen fluoride (HF)/pyridine (70%) (10 mL, 385 mmol, 48 eq) was added dropwise. The solution was stirred for 11 days. After TLC showed complete consumption of the starting material, the reaction was quenched with saturated aqueous sodium bicarbonate slowly and diluted with EtOAc. The solution was washed with water (2x) and brine. The aqueous layer was extracted with EtOAc (3x), dried with $MgSO_4$, filtered, and concentrated *in vacuo*. The compound was purified by flash chromatography (Pentane/EA 4:1 - 3:1) to yield compound **19** (3.54 g, 7.77 mmol, 97%). ^{1}H NMR (400 MHz, $CDCl_3$) δ 7.64 – 7.51 (m, 2H), 7.43 – 7.30 (m, 3H), 5.22 (d, J = 9.6 Hz, 1H, NH), 4.88 (d, J = 12.0 Hz, 1H, Troc), 4.59 (d, J = 11.9 Hz, 1H, Troc), 4.44 (d, J = 10.2 Hz, 1H, H-1), 4.05 – 3.94 (m, 1H, H-4), 3.81 – 3.63 (m, 2H, H-5, H-3), 3.42 (d, J = 4.3 Hz, 1H, 3-OH), 3.22 (t, J = 9.9 Hz, 1H, H-2), 1.27 (d, J = 6.4 Hz, 3H, H-6). ^{13}C NMR (101 MHz, $CDCl_3$) δ 156.13 (Troc), 133.51, 131.39, 129.30, 128.87, 95.37 (Troc), 86.67 (C-1), 74.89 (Troc), 73.83, 73.79 (C-3, C-5), 62.78 (C-2), 55.22 (C-4), 17.07 (C-6). HR-MS: Calculated for $C_{15}H_{17}Cl_3N_4O_4S$ [M+Na]$^+$: 476.9928, found: 476.9930. $[\alpha]^{20}_D$ = - 22° (c = 1, $CHCl_3$). TLC: Rf = 0.2 (Pentane/EA = 4/1, v/v)

Phenyl 2-azido-6-deoxy-3-*O*-levulinoyl-4-*N*-2,2,2-trichloroethyl-1-thio-β-D-galactopyranoside (21)

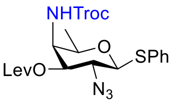

Compound **19** (3.67 g, 8.05 mmol, 1.0 eq) was co-evaporated with anhydrous toluene three times and dissolved in DCM (80 mL). At 0 °C, levulinic acid (2.5 g, 21.5 mmol, 2.7 eq), 1-ethyl-3-(3-dimethylaminopropyl) carbodiimide (EDCI)

(2.4 g, 15.5 mmol, 2.0 eq) and 4-dimethylaminopyridine (DMAP) (940 mg, 8.05 mmol, 1.0 eq) were added. The reaction was stirred overnight. The reaction was diluted with DCM and washed with saturated aqueous sodium bicarbonate and brine. The organic phase was dried with $MgSO_4$, filtered, and concentrated *in vacuo*. The compound was purified by flash chromatography (Pentane/EA 5:1 – 3:1) to yield compound **21** (4.22 g, 7.62 mmol, 95%). ^{1}H NMR (500 MHz, $CDCl_3$) δ 7.63 – 7.55 (m, 2H), 7.42 – 7.35 (m, 3H), 5.07 (d, *J* = 9.7 Hz, 1H, NH), 4.80 (dd, *J* = 10.2, 3.8 Hz, 1H, H-3), 4.72 (s, 2H, Troc), 4.48 (d, *J* = 10.2 Hz, 1H, H-1), 4.16 – 4.09 (m, 1H, H-4), 3.82 – 3.75 (m, 1H, H-5), 3.38 (t, *J* = 10.2 Hz, 1H, H-2), 2.85 – 2.45 (m, 4H, Lev), 2.17 (s, 3H, Lev), 1.26 (d, *J* = 6.5 Hz, 3H, H-6). ^{13}C NMR (126 MHz, $CDCl_3$) δ 206.27 (Lev), 171.97 (Lev), 154.92 (Troc), 133.83, 130.95, 129.34, 129.10, 95.55 (Troc), 86.58 (C-1), 74.81 (C-3), 74.64 (Troc), 73.53 (C-5), 59.63 (C-2), 52.39 (C-4), 37.84 (Lev), 29.83 (Lev), 27.92 (Lev), 16.94 (C-6). HR-MS: Calculated for $C_{20}H_{23}Cl_3N_4O_6S$ [M+H$^+$]: 553.0477, found: 553.0472. $[\alpha]^{20}_D$ = - 27° (c = 1, $CHCl_3$). TLC: Rf = 0.3 (Pentane/EA = 4/1, v/v).

2-azido-6-deoxy-3-*O*-levulinoyl-4-*N*-2,2,2-trichloroethyl-α/β-D-galactopyranoside (16a)

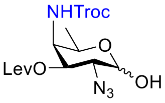

Compound **21** (4.22 g, 7.62 mmol, 1.0 eq) was dissolved in DCM (80 mL) and reduced to 0 °C. NIS (2.6 g, 11.6 mmol, 1.5 eq) and TFA (0.7 mL, 9.1 mmol, 1.2 eq) were added and the solution stirred for 3 hours. The reaction was quenched with triethyl amine and sodium thiosulphate. The solution was diluted with DCM and washed with brine (3×). The organic phase was dried with MgSO4, filtered, and concentrated in vacuo. The compound was purified by flash chromatography (Pentane/ EA 3:1 - 3:2) to yield the titled compound **16a** (3.2 g, 6.93 mmol, 90%). ^{1}H NMR (500 MHz, $CDCl_3$) δ 6.20 – 5.45 (m, 1H, NH), 5.39 – 5.02 (m, 1H), 4.82 – 4.46 (m, 3H), 4.28 – 4.09 (m, 1H, H-4), 3.89 – 3.77 (m, 1H, H-5), 3.68 – 3.52 (m, 1H, H-2), 2.94 – 2.47 (m, 4H, Lev), 2.25 – 2.13 (m, 3H, Lev), 1.32 – 1.16 (m, 3H, H-6). ^{13}C NMR (126 MHz, $CDCl_3$) δ 207.40, 207.22, 172.31, 172.16, 155.38, 155.32, 96.61 (C-1), 95.73 (Troc), 92.15 (C-1), 74.70 (Troc), 73.18 (C-3), 70.48 (C-3), 69.57, 64.39, 62.05, 58.30, 53.38, 52.50, 38.00, 37.96, 29.89, 29.82, 28.08, 28.05, 16.59, 16.49. HR-MS: Calculated for $C_{14}H_{19}Cl_3N_4O_7$ [M+Na$^+$]: 483.0212, found: 483.0211. TLC: Rf = 0.2 (Pentane/EA = 2/1, v/v).

***N*-phenyl-trifluoroacetimidoyl 2-*N*-azido-6-deoxy-3-*O*-levulinoyl-4-*N*-2,2,2-trichloroethyl-α/β-D-galactopyranoside** (**16**)

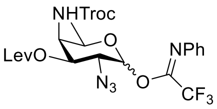

Compound **16a** (3.18 g, 6.89 mmol, 1.0 eq) was dissolved in acetone (70 mL) and cooled to 0 °C. *N*-phenyl trifluoroacetimidoyl chloride (2.14 g, 10.31 mmol, 1.5 eq) and cesium carbonate (2.7 g, 8.29 mmol, 1.2 eq) were

added. The solution was allowed to warm to RT and stirred overnight. The reaction was quenched with triethyl amine and concentrated *in vacuo*. The compound was purified by flash chromatography (Pentane/EA 6:1 - 4:1) to yield compound **16** (3.98 g, 6.29 mmol, 91%). ^{1}H NMR (500 MHz, $CDCl_3$) δ 7.37 – 7.28 (m, 2H), 7.18 – 7.09 (m, 1H), 6.90 – 6.80 (m, 2H), 5.89 – 5.23 (m, 2H), 5.14 – 4.59 (m, 3H), 4.41 – 4.10 (m, 1H), 3.89 – 3.68 (m, 2H), 2.94 – 2.45 (m, 4H), 2.23 – 2.14 (m, 3H), 1.31 – 1.19 (m, 3H). ^{13}C NMR (126 MHz, $CDCl_3$) δ 206.29, 172.00, 155.12, 142.98, 128.97, 128.90, 124.82, 119.27, 95.80, 95.54, 74.79, 74.77, 73.07, 70.76, 67.50, 60.12, 57.11, 52.82, 52.31, 37.91, 37.89, 29.88, 29.38, 27.98, 27.91, 16.42. HR-MS: Calculated for $C_{22}H_{23}Cl_3F_3N_5O_7$ [M+Na$^+$]: 654.0507, found: 654.0503. TLC: Rf = 0.2 (Pentane/EA = 5/1, v/v).

Phenyl 3-*O*-acetyl-2-azido-2-deoxy-4,6-*O*-(2-methylnaphthylidene)-1-seleno-α-D-galactopyranoside (23)

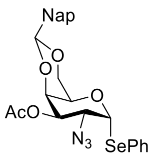

Phenyl 2-azido-1-seleno-β-D-galactopyranoside[2] **22** (9.3 g, 27.1 mmol, 1.0 eq) was dissolved in acetonitrile (270 mL). Freshly prepared[3] 2-naphthaldehyde dimethyl acetal (9.1 g, 44.8 mmol, 1.65 eq) and camphorsufonic acid (CSA) (314 mg, 1.35 mmol, 0.05 eq) were added to the solution and stirred at 50 °C, at 260 mbar for 1 hour, while additional MeCN was added once the solvent had evaporated. After analysis by TLC showed complete consumption of the starting material, the reaction was quenched with triethylamine, co-evaporated with toluene and concentrated *in vacuo*. The crude was dissolved in the pyridine (54 mL) and put in an ice bath, after which Ac_2O (54 mL) was added. The reaction was stirred overnight. After analysis by TLC showed complete consumption of the starting material, the reaction was concentrated *in vacuo*. The compound was purified by flash chromatography (Pentane/EA 20:1 - 10:1) to yield compound **23** (14.2 g, 27.1 mmol, quant.). ^{1}H NMR (400 MHz, $CDCl_3$) δ 7.98 – 7.92 (m, 1H), 7.89 – 7.76 (m, 3H), 7.62 – 7.50 (m, 3H), 7.50 – 7.42 (m, 2H), 7.31 – 7.22 (m, 3H), 6.09 (d, J = 5.2 Hz, 1H, H-1), 5.65 (s, 1H), 5.09 (dd, J = 10.8, 3.4 Hz, 1H, H-3), 4.58 – 4.49 (m, 2H, H-2, H-4), 4.18 – 4.09 (m, 2H, H-5, H-6), 4.03 (dd, J = 12.8, 2.0 Hz, 1H, H-6), 2.15 (s, 3H, OAc). ^{13}C NMR (101 MHz, $CDCl_3$) δ 170.35, 134.72, 134.00, 133.71, 132.84, 129.24, 128.39, 128.31, 128.13, 127.86, 127.72, 126.51, 126.23, 125.64, 123.74, 101.04, 84.94 (C-1), 73.05 (C-4), 72.40 (C-3), 69.02 (C-6), 64.80 (C-5), 58.36 (C-2), 20.98 (Ac). HR-MS: Calculated for $C_{25}H_{23}N_3O_5Se$ [M+H]$^+$: 526.0876, found: 526.0876. $[\alpha]^{20}_D$ = + 214.0° (c = 1, $CHCl_3$). TLC: Rf = 0.2 (Pentane/EA = 9/1, v/v).

Phenyl 3-*O*-acetyl-2-azido-2-deoxy-4-*O*-(2-methylnaphthyl)-1-seleno-α-D-galactopyranoside (24a)

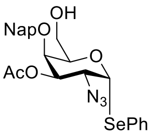

A solution of 1M BH_3 in THF (43 mL, 43 mmol, 2.5 eq) was added to compound **23** (9.1 g, 17.3 mmol, 1.0 eq) in DCM (170 mL) at 0 °C and the solution was stirred for 5 minutes. A solution of 1M Bu_2BOTf in THF (19 mL, 19 mmol, 1.1 eq) was then added to the clear solution slowly. After 2 hours at 0 °C, TLC showed that the starting material had disappeared. The reaction was quenched with triethylamine, followed by careful addition of methanol until the evolution of H_2 had ceased. The reaction mixture was coevaporated with methanol three times. The compound was purified by flash chromatography (Pentane/EA 4:1 - 3:1) to yield compound **24a** (7.7 g, 14.6 mmol, 84%).^{1}H NMR (400 MHz, $CDCl_3$) δ 7.89 – 7.77 (m, 3H), 7.75 – 7.69 (m, 1H), 7.62 – 7.53 (m, 2H), 7.52 – 7.40 (m, 3H), 7.32 – 7.21 (m, 3H), 6.00 (d, *J* = 5.4 Hz, 1H, H-1), 5.08 (dd, *J* = 10.8, 2.9 Hz, 1H, H-3), 4.82 (d, *J* = 11.6 Hz, 1H, Nap), 4.68 (d, *J* = 11.6 Hz, 1H, Nap), 4.47 (dd, *J* = 10.8, 5.4 Hz, 1H, H-2), 4.33 – 4.23 (m, 1H, H-5), 4.17 – 4.07 (m, 1H, H-4), 3.69 (dd, *J* = 11.3, 7.1 Hz, 1H, H-6), 3.51 (dd, *J* = 11.3, 5.1 Hz, 1H, H-6), 2.05 (s, 3H, Ac). ^{13}C NMR (101 MHz, $CDCl_3$) δ 170.23 (Ac), 135.13, 134.75, 133.20, 133.17, 129.29, 128.56, 128.23, 128.01, 127.81, 127.18, 126.51, 126.40, 126.04, 84.11 (C-1), 75.49 (Nap), 74.30 (C-3), 74.29 (C-4), 73.23 (C-5), 61.66 (C-6), 59.35 (C-2), 20.94 (Ac). HR-MS: Calculated for $C_{25}H_{25}N_3O_5Se$ $[M+NH_4]^+$: 545.1298, found: 545.1296. $[\alpha]^{20}_D$ = + 248.8° (c = 1, $CHCl_3$). TLC: Rf = 0.3 (Pentane/EA = 3/1, v/v).

Phenyl 2-azido-2-deoxy-4-*O*-(2-methylnaphthyl)-1-seleno-α-D-galactopyranoside (24)

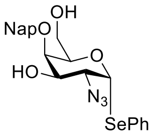

Compound **24a** (1.65 g. 3.13 mmol, 1.0 eq) was dissolved in methanol (20 mL) and DCM (10 mL). Sodium methoxide (25 wt. % in methanol) (0.5 mL, 2.2 mmol, 0.7 eq) was added. The reaction was stirred for 2 hours. After analysis by TLC showed complete consumption of the starting material, the reaction was quenched with amberlite H^+, filtered and concentrated *in vacuo*. The compound was purified by flash chromatography (Pentane/EA 4:1 - 2:1) to yield compound **24** (1.5 g, 3.1 mmol, 99%). ^{1}H NMR (400 MHz, CD_3COCD_3) δ 7.94 – 7.83 (m, 4H), 7.69 – 7.55 (m, 3H), 7.54 – 7.44 (m, 2H), 7.35 – 7.24 (m, 3H), 6.04 (d, *J* = 5.2 Hz, 1H, H-1), 5.23 – 5.11 (m, 1H, Nap), 4.96 – 4.84 (m, 2H, Nap, 3-OH), 4.39 – 4.25 (m, 2H, H-2, H-5), 4.17 – 4.10 (m, 1H, H-4), 4.03 – 3.92 (m, 1H, H-3), 3.90 – 3.71 (m, 2H, 6-OH, H-6), 3.68 – 3.57 (m, 1H, H-6). ^{13}C NMR (101 MHz, $CDCl_3$) δ 137.51, 135.38, 134.18, 133.83, 129.90, 129.86, 128.69, 128.50, 128.42, 128.38, 127.13, 127.01, 126.90, 126.66, 86.79 (C-1), 77.73 (C-4), 76.08 (Nap), 74.90 (C-5), 73.16 (C-3), 63.39 (C-2), 61.35 (C-6). HR-MS: Calculated for $C_{23}H_{23}N_3O_4Se$ $[M+NH_4]^+$: 503.1192, found: 503.1189. $[\alpha]^{20}_D$ = + 226.2° (c = 1, $CHCl_3$). TLC: Rf = 0.2 (Pentane/EA = 3/1, v/v).

Phenyl 2-azido-2-deoxy-3,6-*O*-di-*tert*-butylsilylidene-4-*O*-(2-methylnaphthyl)-1-seleno-α-D-galactopyranoside (25)

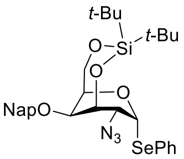

Compound **24** (10.2 g, 21.1 mmol, 1.0 eq) was dissolved in 211 mL dry 2,6-lutidine. To the solution was added di-*tert*-butylsilyl bistriflate (8.2 mL, 25.2 mmol, 1.2 eq) and 4Å molecular sieves, and then the mixture was heated to 100 °C for 3 h. After analysis by TLC showed complete consumption of the starting material, the reaction was cooled to rt, and diluted by EtOAc, and washed 3 times with 1 M HCl solution, one time with sodium bicarbonate solution, and one time with brine. The organic layer was dried with MgSO4, filtered, and evaporated *in vacuo*. The crude product was purified by flash column chromatography (Pentane/EA 80:1 - 50:1) to yield compound **25** (8.8 g, 14.0 mmol, 67%) and several partially silylated side products which could be treated with 1M TBAF (70 mmol) and AcOH (4.0 mL) in THF (70 mL) at 50 °C to recover the starting material **24** (2.43 g, 5.02 mmol, 24%). ^{1}H NMR (500 MHz, $CDCl_3$) δ 7.87 – 7.79 (m, 3H), 7.79 – 7.75 (m, 1H), 7.61 – 7.53 (m, 2H), 7.52 – 7.41 (m, 3H), 7.29 – 7.21 (m, 3H), 6.31 (d, *J* = 3.4 Hz, 1H, H-1), 4.86 (d, *J* = 12.1 Hz, 1H, Nap), 4.71 (d, *J* = 12.1 Hz, 1H, Nap), 4.64 (t, *J* = 2.9 Hz, 1H, H-3), 4.43 (dd, *J* = 13.2, 1.7 Hz, 1H, H-6), 4.24 (dd, *J* = 6.8, 3.0 Hz, 1H, H-4), 4.21 – 4.13 (m, 2H, H-2, H-6), 4.03 – 3.95 (m, 1H, H-5), 1.07 (s, 9H, *t*-Bu), 0.88 (s, 9H, *t*-Bu). ^{13}C NMR (126 MHz, $CDCl_3$) δ 134.46, 133.28, 133.22, 129.59, 129.15, 128.58, 128.07, 127.84, 127.66, 127.45, 126.43, 126.33, 125.99, 79.24 (C-1), 76.14 (C-5), 73.15 (C-4), 72.18 (C-3), 71.45 (Nap), 66.71 (C-2), 65.95 (C-6), 29.27 (*t*-Bu), 28.11 (*t*-Bu), 23.19 (*t*-Bu), 21.03 (*t*-Bu). HR-MS: Calculated for $C_{31}H_{39}N_3O_4SeSi$ $[M+NH_4]^+$: 643.2213, found: 643.2212. $[\alpha]^{20}_D$ = -32.8° (c = 1, $CHCl_3$). TLC: Rf = 0.4 (Pentane/EA = 40/1, v/v).

2-azido-2-((4R,5S,6R)-2,2-di-*tert*-butyl-6-hydroxy-5-(2-methylnaphthyl)-1,3,2-dioxasilepan-4-yl)acetaldehyde (26b)

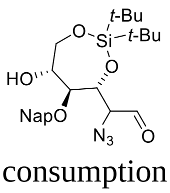

Compound **25** (274 mg, 0.44 mmol, 1.0 eq) was dissolved in acetone/water (4.5/0.5 mL) and cooled to 0 °C. NIS (148 mg, 0.66 mmol, 1.5 eq) was added and the solution stirred for 2 hours. After analysis by TLC showed complete consumption of the starting material, the reaction was quenched sodium thiosulphate. The solution was diluted with DCM and washed with brine (3×). The organic phase was dried with $MgSO_4$, filtered, and concentrated in vacuo. The compound was purified by flash chromatography (Pentane/ EA 10:1 – 8:1) to yield the titled compound **26b** (208 mg, 0.43 mmol, 98%). ^{1}H NMR (500 MHz, $CDCl_3$) δ 9.77 (s, 1H, CHO), 7.89 – 7.73 (m, 4H), 7.54 – 7.44 (m, 2H), 7.40 (dd, *J* = 8.4, 1.7 Hz, 1H), 5.03 – 4.95 (m, 1H, Nap), 4.92 (d, *J* = 11.2 Hz,

1H, Nap), 4.71 (dd, J = 9.1, 1.9 Hz, 1H, H-3), 4.08 (d, J = 1.9 Hz, 1H, H-2), 4.07 – 3.95 (m, 2H, H-6), 3.81 – 3.71 (m, 1H, H-5), 3.56 (t, J = 9.0 Hz, 1H, H-4), 2.55 (d, J = 2.6 Hz, 1H, 5-OH), 1.06 (s, 9H, *t*-Bu), 0.99 (s, 9H, *t*-Bu). ^{13}C NMR (126 MHz, $CDCl_3$) δ 196.33 (CHO), 134.83, 133.42, 133.25, 128.85, 128.08, 127.88, 126.87, 126.57, 126.46, 125.64, 84.65 (C-1), 76.33 (Nap), 75.91 (C-5), 74.06 (C-3), 68.85 (C-2), 65.90 (C-6), 28.02 (*t*-Bu), 27.91 (*t*-Bu), 21.62 (*t*-Bu), 21.59 (*t*-Bu). HR-MS: Calculated for $C_{25}H_{35}N_3O_5Si$ [M+Na$^+$]: 508.2238, found: 508.2234. $[\alpha]^{20}_D$ = - 2.0º (c = 1, $CHCl_3$). TLC: Rf = 0.15 (Pentane/EA = 9/1, v/v).

_N_-phenyl-trifluoroacetimidoyl 2-azido-2-deoxy-3,6-_O_-di-_tert_-butylsilylidene-4-_O_-(2-methylnaphthyl)-α/β-D-galactopyranoside (13)

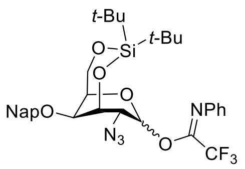

Compound **26b** (507 mg, 1.05 mmol, 1.0 eq) was dissolved in acetone (10.0 mL) and cooled to 0 °C. *N*-phenyl trifluoroacetimidoyl chloride (325 mg, 1.6 mmol, 1.5 eq) and cesium carbonate (409 mg, 1.2 mmol, 1.2 eq) were added. The solution was allowed to warm to RT and stirred for overnight. After analysis by TLC showed complete consumption of the starting material, the reaction was quenched with triethyl amine, filtered, and concentrated *in vacuo*. The compound was purified by flash chromatography (Pentane/Et_2O 100:1 - 50:1) to yield compound **13** (514 mg, 0.78 mmol, 75%). ^{1}H NMR (400 MHz, Acetone) δ 7.99 – 7.81 (m, 4H), 7.59 – 7.45 (m, 3H), 7.37 – 7.22 (m, 2H), 7.11 – 6.94 (m, 1H), 6.86 (d, J = 7.8 Hz, 2H), 6.19 – 5.46 (m, 1H), 5.04 – 4.89 (m, 1H), 4.87 – 4.69 (m, 1H), 4.67 – 4.53 (m, 1H), 4.50 – 3.81 (m, 5H), 1.09 – 0.92 (m, 18H). ^{13}C NMR (101 MHz, Acetone) δ 135.95, 135.47, 134.21, 134.10, 130.76, 129.82, 129.71, 129.64, 129.06, 129.03, 128.77, 128.56, 128.53, 128.40, 127.91, 127.73, 127.22, 127.09, 127.06, 126.96, 126.89, 126.77, 125.30, 120.01, 76.97, 74.87, 74.36, 73.08, 72.50 (Nap), 72.32, 70.21, 67.72, 64.48 (C-6), 64.13, 28.95, 28.82, 28.55, 26.57, 22.67, 21.97. HR-MS: Calculated for $C_{33}H_{39}F_3N_4O_5Si$ [M-[O(C=NPh)CF_3]+OH+NH_4]$^+$: 503.2684, found: 503.2679. TLC: Rf = 0.6 (Pentane/Et_2O = 30/1, v/v).

Allyl 2-azido-2-deoxy-3,6-_O_-di-_tert_-butylsilylidene-4-_O_-(2-methylnaphthyl)-α-D-galactopyranoside (14a)

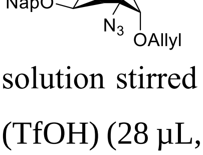

Donor **25** (780 mg, 1.25 mmol, 1.0 eq) was co-evaporated with toluene (3×) and placed under a nitrogen atmosphere. Dry DCM (13.0 mL), allyl alcohol (254 µL, 3.8 mmol, 3.0 eq) and 4Å molecular sieves were added and the solution stirred for 30 minutes at rt, and then cooled to 0 °C. Trifluoromethanesulfonic acid (TfOH) (28 µL, 0.31 mmol, 0.2 eq) and NIS (561 mg, 2.5 mmol, 2.0 eq) were added and stirred 2 hours. After analysis by TLC showed complete consumption of the starting material, the

reaction was quenched with $Na_2S_2O_3$ and $NaHCO_3$ solution, washed with water (2×) and brine (3×). The organic phase was dried with $MgSO_4$, filtered, and concentrated *in vacuo*. The compound was purified by flash chromatography (Pentane/EA 60:1 - 40:1) to yield compound **14a** (594 mg, 1.13 mmol, 90%). ^{1}H NMR (500 MHz, $CDCl_3$) δ 7.86 – 7.76 (m, 3H), 7.76 – 7.70 (m, 1H), 7.52 – 7.39 (m, 3H), 5.88 – 5.74 (m, 1H, Allyl), 5.39 (d, *J* = 4.5 Hz, 1H, H-1), 5.23 – 5.16 (m, 1H, Allyl), 5.11 – 5.05 (m, 1H, Allyl), 4.88 – 4.82 (m, 1H, Nap), 4.66 – 4.58 (m, 1H, Nap), 4.48 (t, *J* = 2.4 Hz, 1H, H-3), 4.40 (dd, *J* = 12.8, 1.6 Hz, 1H, H-6), 4.31 – 4.21 (m, 2H, H-4, Allyl), 4.12 (dd, *J* = 12.7, 2.5 Hz, 1H, H-6), 4.07 – 4.00 (m, 1H, Allyl), 3.96 – 3.89 (m, 1H, H-5), 3.87 (dd, *J* = 4.5, 1.9 Hz, 1H, H-2), 1.06 – 0.97 (m, 18H, *t*-Bu). ^{13}C NMR (126 MHz, $CDCl_3$) δ 134.81, 133.45 (Allyl), 133.28, 133.19, 128.40, 128.06, 127.79, 127.18, 126.29, 126.18, 125.94, 117.43 (Allyl), 94.78 (C-1), 74.13 (C-4), 73.59 (C-5), 72.04 (C-3), 71.61 (Nap), 69.48 (Allyl), 65.00 (C-6), 63.98 (C-2), 28.98 (*t*-Bu), 28.31 (*t*-Bu), 22.80 (*t*-Bu), 21.19 (*t*-Bu). HR-MS: Calculated for $C_{28}H_{39}N_3O_5Si$ $[M+NH_4]^+$: 543.2997, found: 543.2997. $[\alpha]^{20}_D$ = + 35.8° (c = 1, $CHCl_3$). TLC: Rf = 0.5 (Pentane/EA = 40/1, v/v).

Allyl 2-azido-2-deoxy-3,6-*O*-di-*tert*-butylsilylidene-α-D-galactopyranoside (14)

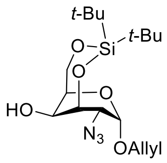

Coupound **14a** (668 mg, 1.31 mmol, 1.0 eq) was dissolved in DCM (13 mL) and water (1.3 mL). After cooling to 0 °C, 2,3-dichloro-5,6-dicyano-*p*-benzoquinone (DDQ) (891 mg, 3.9 mmol, 3.0 eq) was added. The reaction was stirred at RT overnight. After analysis by TLC showed complete consumption of the starting material, quenched by saturated aqueous sodium thiosulphate, extracted with DCM, and washed with water and brine. The organic layer was dried with anhydrous $MgSO_4$, filtered, and concentrated *in vacuo*, and the product purified by column chromatography (PE/EA 30:1 – 15/1) to yield **14** (433 mg, 1.12 mmol, 86%). ^{1}H NMR (400 MHz, $CDCl_3$) δ 6.03 – 5.82 (m, 1H, Allyl), 5.41 – 5.30 (m, 1H, Allyl), 5.29 – 5.18 (m, 2H, H-1, Allyl), 4.58 – 4.47 (m, 1H, H-4), 4.39 – 4.25 (m, 3H, H-6, Allyl, H-3), 4.20 (dd, *J* = 13.1, 1.7 Hz, 1H, H-6), 4.14 – 4.02 (m, 2H, Allyl, H-5), 3.89 (dd, *J* = 5.5, 1.5 Hz, 1H, H-2), 2.63 (d, *J* = 8.8 Hz, 1H, 4-OH), 1.07 – 0.99 (m, 18H, *t*-Bu). ^{13}C NMR (101 MHz, $CDCl_3$) δ 133.39 (CH-Allyl), 117.92 (CH_2-Allyl), 94.44 (C-1), 76.32 (C-3), 73.49 (C-5), 69.75 (Allyl), 68.41 (C-4), 65.62 (C-6), 64.13 (C-2), 28.64 (*t*-Bu), 28.49 (*t*-Bu), 22.21 (*t*-Bu), 21.51 (*t*-Bu). $[\alpha]^{20}_D$ = + 105.9° (c = 1, $CHCl_3$). TLC: Rf = 0.6 (Pentane/EA = 9/1, v/v).

Phenyl 2-*O*-benzoyl-4,6-di-*O*-[1-(*R*)-(methoxycarbonyl)-ethyldiene]-thio-β-D-galactopyranoside (12)

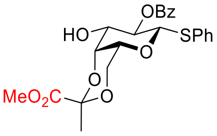

Phenyl 2-*O*-benzoyl-3-*O*-fluorenylmethyloxycarbonyl-4,6-di-*O*-[1-(*R*)-(methoxycarbonyl)-ethyldiene]-thio-β-D-galactopyranoside **27**[4] (4.93 g, 7,23 mmol, 1.0 eq) was dissolved in DCM (73 mL) and triethyl amine (60 mL, 434 mmol, 60 eq) was added and the solution was stirred for 4.5 hours. The solution was co-evaporated with toluene three times and concentrated *in vacuo*. The compound was purified by flash chromatography (PE/EA 3:1 - 1:1) to yield compound **12** (2.82 g, 6.03 mmol, 83%). ^{1}H NMR (400 MHz, Chloroform-*d*) δ 8.11 – 8.05 (m, 2H), 7.64 – 7.42 (m, 5H), 7.32 – 7.23 (m, 3H), 5.24 (t, *J* = 9.7 Hz, 1H, H-2), 4.78 (d, *J* = 9.8 Hz, 1H, H-1), 4.23 – 4.20 (m, 1H, H-4), 4.20 – 4.14 (m, 1H, H-6), 4.04 – 3.97 (m, 1H, H-6), 3.86 – 3.77 (m, 4H, OMe, H-3), 3.54 – 3.48 (m, 1H, H-5), 2.66 (d, *J* = 10.7 Hz, 1H, OH), 1.57 (s, 3H, Me,). ^{13}C NMR (101 MHz, $CDCl_3$) δ 170.21 (CO_2Me), 166.13 (Bz), 133.76, 133.36, 131.67, 130.07, 129.96, 128.87, 128.52, 128.39, 98.75 (C_{quat}), 85.37 (C-1), 72.80 (C-3), 71.46 (C-4), 70.59 (C-2), 69.24 (C-5), 65.42 (C-6), 52.91 (OMe), 25.84 (Me). HR-MS: Calculated for $C_{23}H_{24}O_8S$ [M+Na$^+$]: 483.1084, found: 483.1082. $[\alpha]^{20}_D$ = - 32.2° (c = 1, $CHCl_3$). TLC: Rf = 0.4 (Pentane/EA = 1/1, v/v).

Propynyl 2-*O*-benzoyl-3-*O*-fluorenylmethyloxycarbonyl-4,6-di-*O*-[1-(*R*)-(methoxycarbonyl)-ethyldiene]-β-D-galactopyranoside (28)

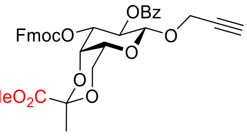

Phenyl 2-*O*-benzoyl-3-*O*-fluorenylmethyloxycarbonyl-4,6-di-*O*-[1-(*R*)-(methoxycarbonyl)-ethyldiene]-thio-β-D-galactopyranoside **27** (1.08 g, 1.59 mmol, 1.0 eq), diphenyl sulfoxide (0.41 g, 2.02 mmol, 1.27 eq) and 2,4,6-tri-*tert*-butylpyrimidine (TTBP) (0.99 g, 3.99 mmol, 2.5 eq) were added to a flask and co-evaporated with toluene (3×) under an argon atmosphere. Dry DCM (36 mL) and molecular sieves (3Å) were added, and the solution cooled to -60 °C. Triflic anhydride (Tf_2O) (0.35 mL, 2.06 mmol, 1.30 eq) was added and the solution stirred for thirty minutes. Propynyl alcohol (0.27 mL, 4.76 mmol, 3 eq) was added and the solution allowed to warm to -40 °C and stirred overnight. The reaction was quenched with sodium bicarbonate, diluted with ethyl acetate, and washed with water (1×) and brine (3×). The compound was dried with $MgSO_4$, filtered, and concentrated *in vacuo*. The column was purified by flash chromatography (PE/EA 5:1 - 1:1) to yield compound **28** (0.80 g, 1.27 mmol, 80%). ^{1}H NMR (500 MHz, Acetone-d_6) δ 8.11 – 8.02 (m, 2H), 7.85 – 7.78 (m, 2H), 7.69 – 7.61 (m, 1H), 7.59 – 7.48 (m, 4H), 7.42 – 7.32 (m, 2H), 7.29 – 7.22 (m, 1H), 7.19 – 7.13 (m, 1H), 5.66 – 5.58 (m, 1H, H-2), 5.17 – 5.11 (m, 1H, H-3), 5.11 – 5.05 (m, 1H, H-1), 4.56 (d, *J* = 3.7 Hz, 1H, H-4), 4.47 – 4.35 (m, 3H), 4.33 – 4.21 (m, 2H), 4.16 – 4.08 (m, 1H, H-6), 4.07 – 4.01 (m, 1H, H-6), 3.87 – 3.82 (m, 1H, H-5), 3.65 (s, 3H, OMe), 2.99 – 2.94 (m, 1H), 1.53 (s, 3H, Me). ^{13}C NMR (126 MHz,

Acetone) δ 168.82, 163.86, 153.01, 142.53, 142.36, 140.15, 132.36, 128.91, 128.66, 127.58, 126.83, 126.80, 126.19, 126.12, 124.16, 124.08, 119.01, 97.55, 97.33 (C-1), 77.82, 74.79, 74.38 (C-3), 68.84 (Fmoc CH_2), 68.01, 67.94, 64.42 (C-5), 63.82 (C-6), 54.33, 50.85 (OMe), 45.38 (Fmoc), 24.25 (Me). HR-MS: Calculated for $C_{35}H_{32}O_{11}$ [$M+NH_4^+$]: 646.2283, found: 646.2280. $[\alpha]^{20}_{D}$ = + 4.4° (c = 1, $CHCl_3$). TLC: Rf = 0.6 (Pentane/EA = 3/2, v/v).

Propynyl 2-*O*-benzoyl-4,6-di-*O*-[1-(*R*)-(methoxycarbonyl)-ethyldiene]-β-D-galactopyranoside (29)

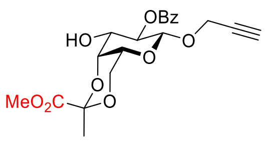

Compound **28** (0.797 g, 1.27 mmol, 1.0 eq) was dissolved in DCM (12 mL) and triethyl amine (9.0 mL, 69 mmol, 55 eq) was added and the solution stirred overnight. After TLC showed complete consumption of the starting material, the reaction was quenched co-evaporated with toluene and concentrated *in vacuo*. The compound was purified by column chromatography (PE/EA 3:1 - 3:2) to yield compound **29** (0.385 g, 0.94 mmol, 75%). ^{1}H NMR (400 MHz, Acetone-d_6) δ 8.13 – 8.03 (m, 2H), 7.69 – 7.57 (m, 1H), 7.57 – 7.44 (m, 2H), 5.34 (dd, J = 9.9, 8.1 Hz, 1H, H-2), 4.88 (d, J = 8.0 Hz, 1H, H-1), 4.37 (d, J = 2.4 Hz, 2H, CH_2), 4.26 (dd, J = 3.7, 1.2 Hz, 1H, H-4), 4.21 (d, J = 8.4 Hz, 1H, OH), 4.09 (dd, J = 12.9, 1.9 Hz, 1H, H-6), 4.05 – 3.94 (m, 2H, H-3, H-6), 3.78 (s, 3H, OMe), 3.68 (t, J = 1.6 Hz, 1H, H-5), 2.93 (t, J = 2.4 Hz, 1H, Alkyn), 1.52 (s, 3H, Me). ^{13}C NMR (101 MHz, Acetone-d_6) δ 170.93 (CO_2Me), 166.10 (Bz), 133.80, 131.32, 130.30, 129.27, 129.22, 99.35 (C-1), 99.22 (C_{quart}), 79.83 (Alkyn), 76.36 (Alkyn), 73.07 (C-2), 72.45 (C-4), 71.47 (C-3), 66.65 (C-5), 65.73 (C-6), 55.87 (alkyn), 52.71 (OMe), 26.12(Me). HR-MS: Calculated for $C_{20}H_{22}O_9$ [$M+Na^+$]: 429.1156, found: 429.1154. $[\alpha]^{20}_{D}$ = - 29.3° (c = 1, $CHCl_3$). TLC: Rf = 0.2 (Pentane/EA = 1/1, v/v).

Phenyl 3-*O*-(2-azido-2-deoxy-3,6-*O*-di-*tert*-butylsilylidene-4-*O*-(2-methylnaphthyl)-α-D-galactopyranoside)-2-*O*-benzoyl-4,6-di-*O*-[1-(*R*)-(methoxycarbonyl)-ethyldiene]-thio-β-D-galactopyranoside (30)

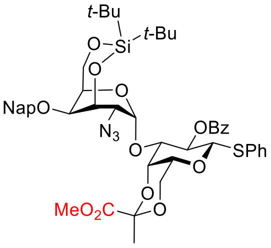

Donor **13** (723 mg, 1.1 mmol, 1.1 eq) and acceptor **12** (460 mg, 1.0 mmol, 1.0 eq) were co-evaporated with toluene (3×) and placed under a nitrogen atmosphere. Dry DCM (10.0 mL) and 4Å molecular sieves were added and the solution stirred for 30 minutes at rt, and then cooled to 0 °C. TBSOTf (50 µL, 0.22 mmol, 0.2 eq) was added and stirred 1 hour. After analysis by TLC showed complete consumption of the starting material, the reaction was quenched with $NaHCO_3$ solution, washed with water (2×) and brine (3×). The

organic phase was dried with $MgSO_4$, filtered, and concentrated *in vacuo*. The compound was purified by flash chromatography (Pentane/EA 8:1 – Pentane/EA/DCM 8:1:1) to yield compound **30** (694 mg, 0.75 mmol, 75%). ^{1}H NMR (500 MHz, Chloroform-*d*) δ 8.12 – 8.01 (m, 2H), 7.87 – 7.76 (m, 2H), 7.74 (d, *J* = 8.4 Hz, 1H), 7.60 – 7.53 (m, 2H), 7.52 – 7.45 (m, 2H), 7.45 – 7.39 (m, 1H), 7.34 – 7.21 (m, 6H), 7.06 (dd, *J* = 8.4, 1.7 Hz, 1H), 5.67 (t, *J* = 9.8 Hz, 1H), 5.49 (d, *J* = 6.3 Hz, 1H), 4.86 (d, *J* = 10.0 Hz, 1H), 4.51 (dd, *J* = 3.5, 1.2 Hz, 1H), 4.37 (d, *J* = 11.5 Hz, 1H), 4.27 – 4.16 (m, 3H), 4.15 – 4.03 (m, 3H), 4.03 – 3.91 (m, 3H), 3.91 – 3.84 (m, 1H), 3.81 (s, 3H), 3.53 – 3.46 (m, 1H), 1.58 (s, 3H), 0.97 (s, 9H), 0.84 (s, 9H). ^{13}C NMR (126 MHz, $CDCl_3$) δ 170.42, 164.69, 134.84, 133.45, 133.24, 133.12, 132.86, 132.29, 129.65, 129.62, 128.84, 128.60, 128.14, 127.96, 127.70, 127.66, 126.13, 126.04, 125.85, 125.59, 98.79, 91.53, 86.17, 75.85, 75.56, 72.13, 71.94, 71.78, 69.02, 68.58, 66.71, 65.60, 63.70, 63.63, 52.68, 28.39, 28.26, 25.51, 22.02, 21.31. TLC: Rf = 0.15 (Pentane/EA = 8/1, v/v).

Phenyl 3-*O*-(2-azido-2-deoxy-3,6-*O*-di-*tert*-butylsilylidene-α-D-galactopyranoside)-2-*O*-benzoyl-4,6-di-*O*-[1-(*R*)-(methoxycarbonyl)-ethyldiene]-thio-β-D-galactopyranoside (31)

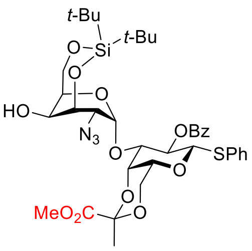

Coupound **30** (669 mg, 0.72 mmol, 1.0 eq) was dissolved in DCM (8.0 mL) and water (0.8 mL). After cooling to 0 °C, 2,3-dichloro-5,6-dicyano-*p*-benzoquinone (DDQ) (327 mg, 1.44 mmol, 2.0 eq) was added. The reaction was stirred at RT overnight. After analysis by TLC showed complete consumption of the starting material, quenched by saturated aqueous sodium thiosulphate, extracted with DCM, and washed with water and brine. The organic layer was dried with anhydrous $MgSO_4$, filtered, and concentrated *in vacuo*, and the product purified by column chromatography (PE/EA/DCM 8:1:1 – 5:1:1) to yield **31** (535 mg, 0.68 mmol, 94%). ^{1}H NMR (400 MHz, Chloroform-*d*) δ 8.09 – 8.01 (m, 2H), 7.64 – 7.57 (m, 1H), 7.57 – 7.51 (m, 2H), 7.51 – 7.43 (m, 2H), 7.32 – 7.22 (m, 3H), 5.59 (t, *J* = 9.8 Hz, 1H), 5.37 (d, *J* = 6.3 Hz, 1H), 4.83 (d, *J* = 10.0 Hz, 1H), 4.53 – 4.43 (m, 1H), 4.23 – 4.09 (m, 2H), 4.06 – 3.84 (m, 7H), 3.79 (s, 3H), 3.51 – 3.45 (m, 1H), 1.84 (d, *J* = 8.2 Hz, 1H), 1.55 (s, 3H), 0.98 – 0.89 (m, 18H). ^{13}C NMR (101 MHz, $CDCl_3$) δ 170.44, 164.83, 133.48, 133.23, 132.26, 129.83, 129.68, 128.84, 128.66, 128.15, 98.74, 91.60, 86.06, 75.99, 72.60, 69.02, 68.56, 67.77, 66.90, 65.60, 64.50, 63.80, 52.69, 28.46, 28.24, 25.51, 21.88, 21.45. TLC: Rf = 0.30 (Pentane/Acetone = 7/1, v/v).

Propynyl 3-*O*-(2-azido-2-deoxy-3,6-*O*-di-*tert*-butylsilylidene-4-*O*-(2-methylnaphthyl)-α-D-galactopyranoside)-2-*O*-benzoyl-4,6-di-*O*-[1-(*R*)-(methoxycarbonyl)-ethyldiene]-β-D-galactopyranoside (32)

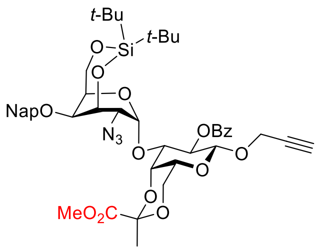

Donor **13** (513 mg, 0.78 mmol, 1.2 eq) and acceptor **29** (265 mg, 0.65 mmol, 1.0 eq) were co-evaporated with toluene (3×) and placed under a nitrogen atmosphere. Dry DCM (8.0 mL) and 4Å molecular sieves were added and the solution stirred for 30 minutes at rt, and then cooled to 0 °C. Trifluoromethanesulfonic acid (TfOH) (12 µL, 0.13 mmol, 0.2 eq) was added and stirred 1 hour. After analysis by TLC showed complete consumption of the starting material, the reaction was quenched with $NaHCO_3$ solution, washed with water (2×) and brine (3×). The organic phase was dried with $MgSO_4$, filtered, and concentrated *in vacuo*. The compound was purified by flash chromatography (Pentane/EA 10:1 - 3:1) to yield compound **32** (447 mg, 0.51 mmol, 78%). ^{1}H NMR (500 MHz, $CDCl_3$) δ 8.10 – 8.00 (m, 2H), 7.85 – 7.74 (m, 2H), 7.71 (d, *J* = 8.5 Hz, 1H), 7.51 – 7.43 (m, 2H), 7.43 – 7.38 (m, 1H), 7.29 – 7.19 (m, 4H), 7.04 (dd, *J* = 8.4, 1.7 Hz, 1H), 5.67 (dd, *J* = 10.0, 8.1 Hz, 1H, H-2a), 5.48 (d, *J* = 6.3 Hz, 1H, H-1b), 4.90 (d, *J* = 8.1 Hz, 1H, H-1a), 4.51 – 4.41 (m, 2H, H-4a, Propynyl), 4.39 – 4.30 (m, 2H, Propynyl, Nap), 4.25 – 4.06 (m, 6H, H-5b, H-6b, H-6a, H-3a, H-3b, H-4b), 4.05 – 3.96 (m, 2H, H-6b, H-6a), 3.95 – 3.90 (m, 1H, H-2b), 3.91 – 3.84 (m, 1H, Nap), 3.80 (s, 3H, COOMe), 3.52 – 3.42 (m, 1H, H-5a), 2.35 (t, *J* = 2.4 Hz, 1H, Propynyl), 1.61 (s, 3H, Me), 0.96 (s, 9H, *t*-Bu), 0.83 (s, 9H, *t*-Bu). ^{13}C NMR (126 MHz, $CDCl_3$) δ 170.42 (COOMe), 164.92 (Bz), 134.82, 133.32, 133.12, 132.87, 129.77, 129.63, 128.48, 127.97, 127.68, 126.20, 126.03, 125.85, 125.63, 98.96 (C_{quart}), 98.31 (C-1a), 91.66 (C-1b), 78.66 (Propynyl), 75.87 (C-4b), 75.25 (Propynyl), 74.44 (C-3a), 72.23 (Nap), 72.05 (C-3b), 71.75 (C-5b), 69.88 (C-2a), 66.61 (C-4a), 65.82 (C-5a), 65.33 (C-6a), 63.73 (C-6b), 63.64 (C-2b), 55.62 (Propynyl), 52.71 (COOMe), 28.39 (*t*-Bu), 28.26 (*t*-Bu), 25.57 (Me), 22.03 (*t*-Bu), 21.33 (*t*-Bu). HR-MS: Calculated for $C_{45}H_{55}N_3O_{13}Si$ $[M+Na]^+$: 896.3396, found: 896.3396. $[\alpha]^{20}_D$ = + 69.4° (c = 1, $CHCl_3$). TLC: Rf = 0.3 (Pentane/EA = 3/1, v/v).

Propynyl 3-*O*-(2-azido-2-deoxy-3,6-*O*-di-*tert*-butylsilylidene-α-D-galactopyranoside)-2-*O*-benzoyl-4,6-di-*O*-[1-(*R*)-(methoxycarbonyl)-ethyldiene]-β-D-galactopyranoside (33)

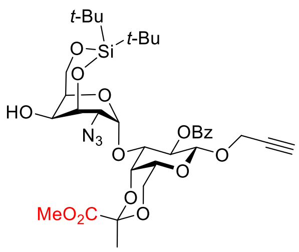

Coupound **32** (45 mg, 0.05 mmol, 1.0 eq) was dissolved in DCM (2.0 mL) and water (0.2 mL). After cooling to 0 °C, 2,3-dichloro-5,6-dicyano-*p*-benzoquinone (DDQ) (35 mg, 0.15 mmol, 3.0 eq) was added. The reaction was stirred at RT overnight. After analysis by TLC showed complete consumption of the starting material, quenched by saturated aqueous sodium thiosulphate, extracted with DCM, and washed with water and brine. The organic layer was dried with anhydrous $MgSO_4$, filtered, and concentrated

in vacuo, and the product purified by column chromatography (PE/EA 4:1 – 3:1) to yield **33** (33.7 mg, 0.046 mmol, 89%). ^{1}H NMR (500 MHz, $CDCl_3$) δ 8.10 – 8.00 (m, 2H), 7.62 – 7.54 (m, 1H), 7.45 (t, *J* = 7.8 Hz, 2H), 5.61 (dd, *J* = 10.0, 8.1 Hz, 1H), 5.41 (d, *J* = 6.4 Hz, 1H), 4.89 (d, *J* = 8.1 Hz, 1H), 4.49 – 4.40 (m, 2H), 4.36 (dd, *J* = 16.1, 2.5 Hz, 1H), 4.24 – 4.12 (m, 2H), 4.08 – 3.91 (m, 6H), 3.91 – 3.85 (m, 1H), 3.81 (s, 3H), 3.52 – 3.44 (m, 1H), 2.38 (t, *J* = 2.4 Hz, 1H), 2.06 (s, 1H), 1.60 (s, 3H), 1.01 – 0.90 (m, 19H). ^{13}C NMR (126 MHz, $CDCl_3$) δ 170.39, 165.04, 133.29, 129.89, 129.61, 128.48, 98.85, 98.25, 91.65, 78.60, 75.98, 75.30, 74.77, 72.59, 69.84, 67.67, 66.77, 65.75, 65.28, 64.43, 63.72, 55.57, 52.67, 28.40, 28.23, 25.52, 21.86, 21.39. HR-MS: Calculated for $C_{34}H_{47}N_3O_{13}Si$ [M+NH$_4$]$^+$: 751.3216, found: 751.3210. $[\alpha]^{20}_{D}$ = + 78.2° (c = 1, $CHCl_3$). TLC: Rf = 0.30 (Pentane/Acetone = 3/1, v/v).

Phenyl 3-*O*-(2-azido-4-*O*-(2-azido-3-*O*-levulinoyl-6-deoxy-4-*N*-phenoxyacetimide-α-D-galactopyranoside)-3,6-*O*-di-*tert*-butylsilylidene-α-D-galactopyranoside)-2-*O*-benzoyl-4,6-di-*O*-[1-(*R*)-(methoxycarbonyl)-ethyldiene]-thio-β-D-galactopyranoside (34)

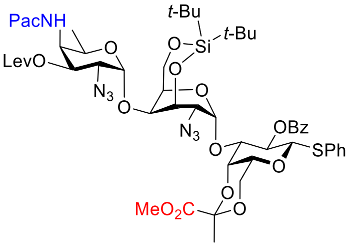

Donor **15** (218 mg, 0.37 mmol, 2.0 eq) and acceptor **31** (147 mg, 0.187 mmol, 1.0 eq) were co-evaporated with toluene (3×) and placed under a nitrogen atmosphere. Dry DCM (1.0 mL) and 5Å molecular sieves were added and the solution stirred for 30 minutes, then cooled to 0 °C. TBSOTf (9.0 μL, 0.04 mmol, 0.1 eq) was added to the reaction mixture. The solution was stirred for 3 hours. After analysis by TLC showed complete consumption of the starting material, the reaction was quenched with saturated aqueous $NaHCO_3$, washed with water (2×) and brine (3×). The organic phase was dried with $MgSO_4$, filtered, and concentrated *in vacuo*. The compound was purified by flash chromatography (Pentane/Acetone 4:1 - 3:1) to yield compound **34** (75.7 mg, 0.064 mmol, 34%). ^{1}H NMR (400 MHz, Chloroform-*d*) δ 8.13 – 8.06 (m, 2H), 7.57 – 7.48 (m, 3H), 7.47 – 7.39 (m, 4H), 7.32 – 7.25 (m, 3H), 7.18 – 7.11 (m, 1H), 7.10 – 7.05 (m, 2H), 6.53 (d, *J* = 9.4 Hz, 1H), 5.62 (t, *J* = 9.8 Hz, 1H), 5.40 (d, *J* = 6.5 Hz, 1H), 4.98 (dd, *J* = 11.2, 3.9 Hz, 1H), 4.82 (d, *J* = 10.0 Hz, 1H), 4.68 – 4.53 (m, 2H), 4.47 (dd, *J* = 3.5, 1.1 Hz, 1H), 4.42 – 4.34 (m, 1H), 4.24 – 4.02 (m, 6H), 3.98 – 3.73 (m, 8H), 3.54 – 3.46 (m, 1H), 3.42 (d, *J* = 4.2 Hz, 1H), 2.87 – 2.76 (m, 2H), 2.68 – 2.47 (m, 2H), 2.19 (s, 3H), 1.03 (d, *J* = 6.6 Hz, 3H), 0.98 – 0.94 (m, 18H). ^{13}C NMR (101 MHz, $CDCl_3$) δ 206.51, 171.70, 170.43, 169.36, 164.34, 157.43, 133.43, 133.26, 132.22, 130.24, 130.10, 129.79, 128.88, 128.60, 128.22, 122.85, 115.04, 98.82, 98.72, 91.64, 86.20, 75.64, 75.34, 74.92, 70.80, 70.69, 69.03, 68.57, 68.03, 66.59, 65.60, 64.57, 63.91, 62.89,

58.14, 52.76, 50.38, 37.98, 29.90, 28.38, 28.15, 27.88, 25.51, 21.99, 21.31, 16.28. TLC: Rf = 0.4 (Pentane/Acetone = 3/1, v/v).

Table 1. Optimization of the **[1+2]** glycosylation condition

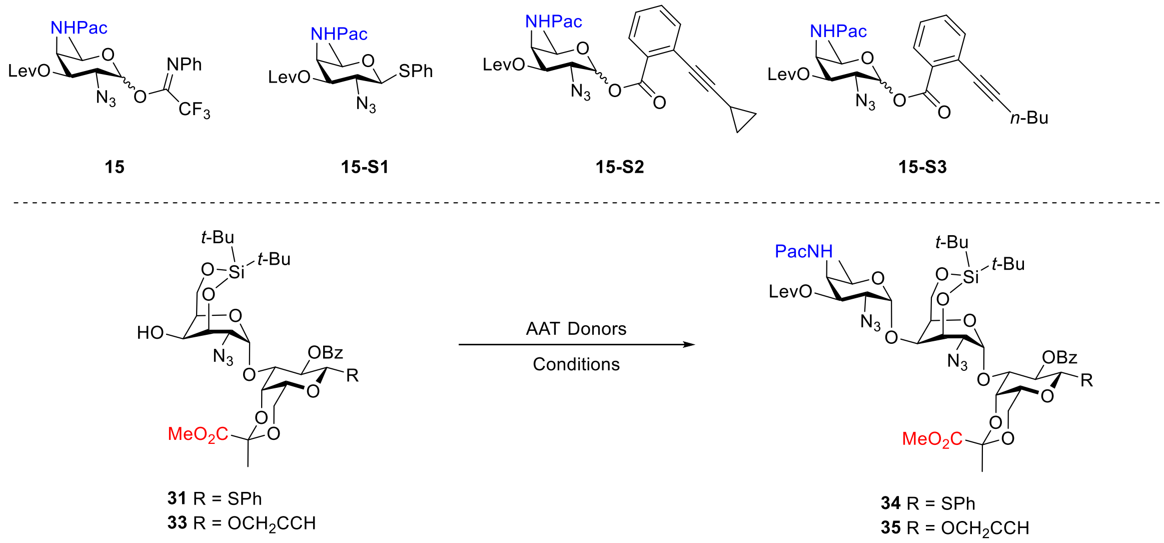

Donor	Acceptor	Conditions	Yield
**15**	**31**	TBSOTf (0.1 eq), 0 °C, DCM	16%
**15**	**31**	TBSOTf (0.1 eq), -78 °C, DCM	NR
**15**	**31**	TBSOTf (1.0 eq), MPF, -78 °C to 0 °C, DCM	<10%
**15**	**31**	TBSOTf (0.1 eq), 0 °C, 0.2M, DCM	34%
**15**	**33**	TBSOTf (0.4 eq), 0 °C, DCM	20%
**15**	**33**	TfOH (0.4 eq), 0 °C, DCM	21%
**15**	**33**	TfOH (1.0 eq), TTBP (2.0 eq), DCM	<10%
**15-S1**	**33**	DMTST (6.0 eq), TTBP, DCM	<10%
**15-S1**	**33**	MeOTf (6.0 eq), rt, DCM	15%
**15-S1**	**33**	MeOTf (6.0 eq), DCE, 60 °C	Decomposed
**15-S2**	**33**	NIS (2.5 eq), TMSOTf (0.2 eq), DCM	16%
**15-S3**	**33**	PPh_3AuNTf_2(0.1 eq), -78 °C to rt, DCM	12%
**15-S3**	**33**	PPh_3AuNTf_2(1.0 eq), rt, DCM	16%

Allyl 2-azido-2-deoxy-4-*O*-(2-azido-3-*O*-levulinoyl-6-deoxy-4-*N*-phenoxyacetimide-α-D-galactopyranoside)-3,6-*O*-di-*tert*-butylsilylidene-α-D-galactopyranoside (36)

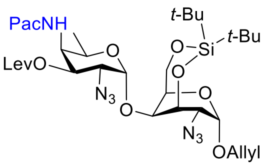

Donor **15** (574 mg, 0.97 mmol, 1.7 eq) and acceptor **14** (214 mg, 0.55 mmol, 1.0 eq) were co-evaporated with toluene (3×) and placed under a nitrogen atmosphere. Dry DCM (6.0 mL) and 4Å molecular sieves were added and the solution stirred for 30 minutes before being cooled to 0 °C. Trifluoromethanesulfonic acid (TfOH) (20 µL, 0.23 mmol, 0.4 eq) was added to the reaction mixture. The solution was stirred for 8h. After analysis by TLC showed complete consumption of the starting material, the reaction was quenched with $NaHCO_3$ solution, washed with water (2×) and brine (3×). The organic phase was dried with $MgSO_4$, filtered, and concentrated *in vacuo*. The compound was purified by flash chromatography (Pentane/Acetone 8:1 - 5:1) to yield compound **36** (416 mg, 0.53 mmol, 95%). ^{1}H NMR (400 MHz, $CDCl_3$) δ 7.40 – 7.31 (m, 2H), 7.10 – 7.03 (m, 1H), 7.00 – 6.92 (m, 2H), 6.64 (d, *J* = 9.5 Hz, 1H), 6.03 – 5.83 (m, 1H, CH-Allyl), 5.40 (d, *J* = 4.8 Hz, 1H, H-1b), 5.39 – 5.30 (m, 1H, Allyl), 5.28 – 5.13 (m, 2H, Allyl, H-3c), 4.83 (d, *J* = 4.2 Hz, 1H, H-1c), 4.68 – 4.53 (m, 2H, Pac), 4.53 – 4.28 (m, 5H, H-4c, H-4b, H-5c, H-6b, Allyl), 4.24 – 4.06 (m, 3H, H-3b, H-6b, Allyl), 4.04 – 3.94 (m, 1H, H-5b), 3.86 (dd, *J* = 4.9, 1.7 Hz, 1H, H-2b), 3.34 (dd, *J* = 11.2, 4.1 Hz, 1H, H-2c), 2.95 – 2.47 (m, 4H, Lev), 2.20 (s, 3H, Lev), 1.12 (d, *J* = 6.6 Hz, 3H, H-6c), 1.08 (s, 9H, *t*-Bu), 1.00 (s, 9H, *t*-Bu). ^{13}C NMR (101 MHz, $CDCl_3$) δ 206.42 (Lev), 171.79 (Lev), 169.31 (Pac), 157.04 (Pac), 133.41, 130.06, 122.63, 117.93, 114.77, 99.40 (C-1c), 94.80 (C-1b), 75.91 (C-4b), 74.91 (C-3b), 72.21 (C-5b), 70.93 (C-3c), 69.83 (Allyl), 67.37 (Pac), 65.08 (C-5c), 64.29 (C-2b), 64.15 (C-6b), 58.02 (C-2c), 50.47 (C-4c), 38.00 (Lev), 29.90 (Lev), 28.56 (*t*-Bu), 28.44 (*t*-Bu), 27.91 (Lev), 22.36 (*t*-Bu), 21.19 (*t*-Bu), 16.36 (C-6c). HR-MS: Calculated for $C_{36}H_{53}N_7O_{11}Si$ $[M+NH_4]^+$: 805.3911, found: 805.3906. $[\alpha]^{20}_D$ = + 99.4° (c = 1, $CHCl_3$). TLC: Rf = 0.5 (Pentane/Acetone = 4/1, v/v).

2-azido-2-deoxy-4-*O*-(2-azido-3-*O*-levulinoyl-6-deoxy-4-*N*-phenoxyacetimide-α-D-galactopyranoside)-3,6-*O*-di-*tert*-butylsilylidene-α/β-D-galactopyranoside (37a)

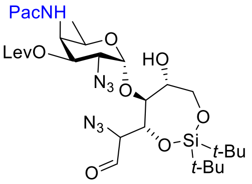

Compound **36** (416 mg, 0.53 mmol, 1.0 eq) was dissolved in freshly distilled THF (6 ml). The mixture was degassed and placed under an argon atmosphere. (1,5-Cyclooctadiene) (pyridine)-(tricyclohexylphosphine)-iridium(I) hexafluorophosphate ($Ir(COD)(Ph_2MeP)_2{\cdot}PF_6$) (23 mg, 0.03 mmol, 0.05 eq) was added and the reaction mixture was degassed and then purged with H_2 for 30 seconds. The reaction mixture was stirred for 1 hour

under an argon atmosphere. After analysis by TLC showed complete consumption of the starting material, the mixture was diluted with THF and *N*-iodosuccinimide (NIS) (180 mg, 0.8 mmol, 1.5 eq) and water were added, and the solution stirred for 2 hours at room temperature. After analysis by TLC showed complete consumption of the starting material, EtOAc was added, and the organic layer was washed two times with saturated aqueous sodium thiosulphate and brine. The organic layer was dried over $MgSO_4$ and concentrated *in vacuo*. Column chromatography (Pentane/Acetone 4:1 - 2:1) yielded **37a** (395 mg, 0.53 mmol, quant.). ^1H NMR (400 MHz, $CDCl_3$) δ 9.82 (s, 1H), 7.41 – 7.30 (m, 2H), 7.12 – 7.03 (m, 1H), 7.01 – 6.90 (m, 2H), 6.61 (d, *J* = 9.1 Hz, 1H), 5.25 – 5.09 (m, 2H), 4.73 – 4.66 (m, 1H), 4.66 – 4.47 (m, 4H), 4.23 – 4.13 (m, 1H), 4.01 – 3.72 (m, 3H), 3.66 (t, *J* = 8.9 Hz, 1H), 3.56 – 3.35 (m, 2H), 2.98 – 2.47 (m, 4H), 2.21 (s, 3H), 1.10 – 0.98 (m, 21H). ^{13}C NMR (126 MHz, $CDCl_3$) δ 211.00, 206.51, 196.43, 177.58, 172.14, 169.58, 169.53, 156.98, 130.13, 130.11, 122.75, 114.75, 114.73, 99.86, 86.87, 76.13, 73.28, 71.96, 68.76, 67.31, 65.82, 65.49, 58.78, 53.90, 50.38, 37.90, 37.88, 29.90, 29.80, 29.70, 29.37, 28.09, 27.97, 27.90, 27.87, 27.79, 21.59, 16.41. HR-MS: Calculated for $C_{33}H_{49}N_7O_{11}Si$ $[M+NH_4]^+$: 765.3598, found: 765.3592. TLC: Rf = 0.25 (Pentane/Acetone = 4/1, v/v).

***N*-phenyl-trifluoroacetimidoyl 2-azido-2-deoxy-4-*O*-(2-azido-3-*O*-levulinoyl-6-deoxy-4-*N*-phenoxyacetimide-α-D-galactopyranoside)-3,6-*O*-di-*tert*-butylsilylidene-α/β-D-galactopyranoside (37)**

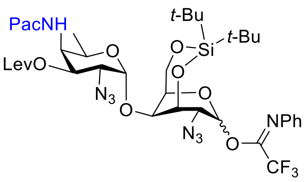

Compound **37a** (395 mg, 0.53 mmol, 1.0 eq) was dissolved in acetone (6.0 mL) and reduced to 0 °C. *N*-phenyl trifluoroacetimidoyl chloride (164 mg, 0.79 mmol, 1.5 eq) and cesium carbonate (206 mg, 0.63 mmol, 1.2 eq) were added. The solution was allowed to warm to RT and stirred overnight. After analysis by TLC showed complete consumption of the starting material, the reaction was quenched with triethyl amine, filtered, and concentrated *in vacuo*. The compound was purified by flash chromatography (Pentane/Acetone 6:1 - 5:1) to yield compound **37** (455 mg, 0.50 mmol, 94%). ^1H NMR (500 MHz, Acetone) δ 7.43 – 7.28 (m, 4H), 7.20 – 7.07 (m, 2H), 7.06 – 6.95 (m, 3H), 6.95 – 6.83 (m, 2H), 5.45 – 5.03 (m, 2H), 4.74 – 4.24 (m, 8H), 4.24 – 3.77 (m, 3H), 2.95 – 2.36 (m, 4H), 2.14 – 2.09 (m, 3H), 1.18 – 0.98 (m, 21H). ^{13}C NMR (126 MHz, Acetone) δ 206.13, 172.39, 172.17, 172.15, 169.88, 169.81, 169.77, 158.67, 158.65, 144.46, 139.26, 130.47, 130.44, 129.72, 125.33, 125.25, 125.17, 122.53, 122.51, 122.46, 120.04, 119.98, 118.31, 115.64, 115.62, 115.60, 101.31, 99.72, 99.44, 87.97, 77.60, 76.86, 75.91, 75.72, 74.74, 74.54, 73.78, 73.57, 71.80, 71.47, 71.39, 68.09, 67.93,

66.70, 66.51, 66.13, 66.04, 64.63, 64.61, 64.01, 59.99, 59.04, 58.94, 51.15, 51.12, 51.10, 51.06, 38.20, 38.15, 29.58, 29.08, 29.00, 28.80, 28.70, 28.67, 28.65, 28.16, 28.00, 22.74, 22.53, 22.34, 22.08, 21.96, 21.86, 16.56, 16.55, 16.24. HR-MS: Calculated for $C_{41}H_{53}F_3N_8O_{11}Si$ $[M+NH_4]^+$: 936.3893, found: 936.3886. TLC: Rf = 0.7 (Pentane/Acetone = 3/1, v/v).

Propynyl 3-*O*-(2-azido-4-*O*-(2-azido-3-*O*-levulinoyl-6-deoxy-4-*N*-phenoxyacetimide-α-D-galactopyranoside)-3,6-*O*-di-*tert*-butylsilylidene-α-D-galactopyranoside)-2-*O*-benzoyl-4,6-di-*O*-[1-(*R*)-(methoxycarbonyl)-ethyldiene]-β-D-galactopyranoside (35)

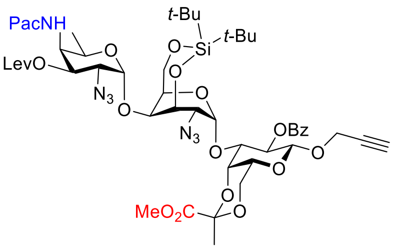

Donor **37** (73.3 mg, 0.08 mmol, 1.2 eq) and acceptor **29** (27 mg, 0.066 mmol, 1.0 eq) were co-evaporated with toluene (3×) and placed under a nitrogen atmosphere. Dry DCM (2.0 mL) and 4Å molecular sieves were added and the solution stirred for 30 minutes, then cooled to 0 °C. TBSOTf (2.0 μL, 0.008 mmol, 0.1 eq) was added to the reaction. The solution was stirred for 3 hours. After analysis by TLC showed complete consumption of the starting material, the reaction was quenched with saturated aqueous $NaHCO_3$, washed with water (2×) and brine (3×). The organic phase was dried with $MgSO_4$, filtered, and concentrated *in vacuo*. The compound was purified by flash chromatography (Pentane/Acetone 3:1 - 2:1) to yield compound **35** (54 mg, 0.048 mmol, 72%). ^{1}H NMR (500 MHz, $CDCl_3$) δ 8.14 – 8.05 (m, 2H), 7.53 – 7.46 (m, 1H), 7.46 – 7.37 (m, 4H), 7.18 – 7.11 (m, 1H), 7.09 – 7.02 (m, 2H), 6.52 (d, J = 9.3 Hz, 1H, NH), 5.65 (dd, J = 10.0, 8.1 Hz, 1H, H-2a), 5.41 (d, J = 6.4 Hz, 1H, H-1b), 4.98 (dd, J = 11.2, 3.9 Hz, 1H, H-3c), 4.88 (d, J = 8.1 Hz, 1H, H-1a), 4.60 (q, J = 15.4 Hz, 2H, Pac), 4.50 – 4.42 (m, 2H, H-4a, CH_2-propynyl), 4.41 – 4.32 (m, 2H, H-4c, CH_2-propynyl), 4.23 – 4.05 (m, 5H, H-6a, H-6b, H-5c, H-4b, H-3a), 4.02 – 3.89 (m, 3H, H-6a, H-3b, H-6b), 3.89 – 3.84 (m, 2H, H-2b, H-5b), 3.81 (s, 3H, COOMe), 3.52 – 3.46 (m, 1H, H-5a), 3.44 (d, J = 4.2 Hz, 1H, H-1c), 2.93 – 2.46 (m, 5H, Lev, H-2c), 2.35 (t, J = 2.4 Hz, 1H, CH-propynyl), 2.19 (s, 3H, Lev), 1.62 (s, 3H, Me), 1.03 (d, J = 6.6 Hz, 3H, H-6c), 1.00 – 0.91 (m, 18H, *t*-Bu). ^{13}C NMR (126 MHz, $CDCl_3$) δ 206.49 (Lev), 171.72 (Lev), 170.44 (COOMe), 169.36 (Pac), 164.60 (Bz), 157.44 (Pac), 133.31, 130.25, 130.23, 130.10, 129.99, 128.49, 122.86, 115.04, 99.02 (C_{quart}), 98.62 (C-1c, J_{CH} = 172 Hz), 98.33 (C-1a, J_{CH} = 160 Hz), 91.85 (C-1b, J_{CH} = 171 Hz), 78.66 (propynyl), 75.26 (propynyl), 75.18 (C-4b), 74.97 (C-3b), 74.50 (C-3a), 70.86 (C-3c), 70.70 (C-5b), 69.86 (C-2a), 68.04 (Pac), 66.52 (C-4a), 65.87 (C-5a), 65.35 (C-6a), 64.61 (C-5c), 63.99 (C-2b), 62.96 (C-6b), 58.20 (C-2c), 55.68 (propynyl), 52.80 (COOMe), 50.42 (C-4c), 38.01 (Lev), 29.92 (Lev), 28.42 (*t*-Bu), 28.18 (*t*-Bu), 27.91 (Lev), 25.59 (Me), 22.01 (*t*-Bu), 21.35 (*t*-Bu), 16.30 (C-6c). HR-MS: Calculated

for $C_{53}H_{69}N_7O_{19}Si$ $[M+Na]^+$: 1158.4310, found: 1158.4300. $[\alpha]^{20}_D$ = + 82.0º (c = 1, $CHCl_3$). TLC: Rf = 0.2 (Pentane/Acetone = 3/1, v/v).

Propynyl 3-*O*-(2-azido-4-*O*-(2-azido-3-*O*-levulinoyl-6-deoxy-4-*N*-phenoxyacetimide-α-D-galactopyranoside)-α-D-galactopyranoside)-2-*O*-benzoyl-4,6-di-*O*-[1-(*R*)-(methoxycarbonyl)-ethyldiene]-β-D-galactopyranoside (38)

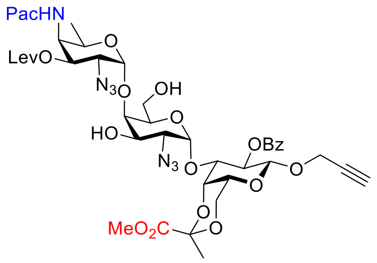

Compound **35** (22.6 mg, 0.02 mmol, 1.0 eq) was dissolved in THF (2.0 mL) and AcOH (12 µL, 0.2 mmol, 10.0 eq). Then 1M TBAF in THF (0.2 mL, 0.2 mmol, 10.0 eq) was added at 0 °C. The reaction mixture was stirred at RT overnight. After TLC showed complete consumption of the starting material, the reaction was quenched with saturated aqueous ammonium chloride and diluted with EtOAc. The solution was washed with water (2x) and brine. The aqueous layer was extracted with EtOAc (3x), dried with $MgSO_4$, filtered, and concentrated *in vacuo*. The compound was purified by flash chromatography (Pentane/Acetone 3:2 – 1:1) to yield compound **38** (19.7 mg, 0.02 mmol, 99%). ^{1}H NMR (500 MHz, $CDCl_3$) δ 8.15 – 8.05 (m, 2H), 7.64 – 7.56 (m, 1H), 7.52 – 7.44 (m, 2H), 7.38 – 7.30 (m, 2H), 7.08 – 7.01 (m, 1H), 6.97 – 6.90 (m, 2H), 6.58 (d, *J* = 9.2 Hz, 1H, NH), 5.57 (dd, *J* = 10.0, 8.1 Hz, 1H, H-2a), 5.19 (d, *J* = 3.5 Hz, 1H, H-1b), 5.14 (dd, *J* = 11.1, 3.7 Hz, 1H, H-3c), 4.88 (d, *J* = 8.1 Hz, 1H, H-1a), 4.80 (d, *J* = 4.1 Hz, 1H, H-1c), 4.66 – 4.32 (m, 7H, Pac, H-5c, H-4c, propynyl, H-4a), 4.17 (dd, *J* = 12.9, 1.6 Hz, 1H, H-6a), 4.08 – 3.96 (m, 2H, H-6a, H-3a), 3.96 – 3.88 (m, 1H, H-3b), 3.84 (s, 3H, COOMe), 3.77 – 3.71 (m, 1H, H-4b), 3.68 (t, *J* = 7.0 Hz, 1H, H-5b), 3.56 (t, *J* = 9.6 Hz, 1H, H-6b), 3.51 – 3.42 (m, 2H, H-6b, H-5a), 3.30 (dd, *J* = 11.1, 3.9 Hz, 1H, H-2c), 3.22 – 3.07 (m, 2H, H-2b), 2.93 – 2.46 (m, 4H, Lev), 2.36 (t, *J* = 2.4 Hz, 1H, CH-propynyl), 2.20 (s, 3H, Lev), 1.65 (s, 3H, Me), 1.04 (d, *J* = 6.3 Hz, 3H, H-6c). ^{13}C NMR (126 MHz, $CDCl_3$) δ 206.54 (Lev), 172.18 (Lev), 170.48 (COOMe), 169.46 (Bz), 165.12 (Pac), 157.00 (Pac), 133.51, 130.12, 129.93, 129.86, 128.67, 122.73, 114.73, 99.11 (C-1c, J_{CH} = 171 Hz), 99.01 (C_{quart}), 98.26 (C-1a, J_{CH} = 160 Hz), 95.16 (C-1b, J_{CH} = 173 Hz), 79.50 (C-4b), 78.70 (propynyl), 75.28 (propynyl), 74.44 (C-3a), 71.28 (C-3c), 70.72 (C-5b), 69.91 (C-2a), 67.36 (Pac), 67.15 (C-3b), 67.03 (C-4a), 65.92 (C-5a), 65.75 (C-5c), 65.34 (C-6a), 60.35 (C-2b), 60.15 (C-6b), 58.51 (C-2c), 55.66 (propynyl), 52.91 (COOMe), 50.43 (C-4c), 37.91 (Lev), 29.95 (Lev), 28.01 (Lev), 25.70 (Me), 16.42 (C-6c). HR-MS: Calculated for $C_{45}H_{53}N_7O_{19}$ $[M+NH_4]^+$: 1013.3734, found: 1013.3735. $[\alpha]^{20}_D$ = + 105.5º (c = 1, $CHCl_3$). TLC: Rf = 0.2 (Pentane/Acetone = 3/2, v/v).

Propynyl 3-*O*-(2-acetamido-2-deoxy-4-*O*-(2-acetamido-2-deoxy-3-*O*-levulinoyl-6-deoxy-4-*N*-phenoxyacetimide-α-D-galactopyranoside)-α-D-galactopyranoside)-2-*O*-benzoyl-4,6-di-*O*-[1-(*R*)-(methoxycarbonyl)-ethyldiene]-β-D-galactopyranoside (39)

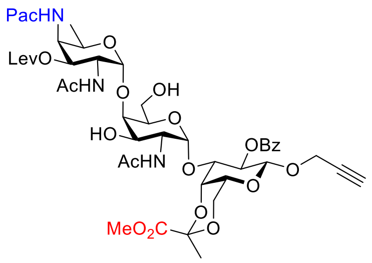

Compound **38** (134 mg, 0.135 mmol, 1.0 eq) was dissolved in pyridine (6.0 mL) and water (2.0 mL at rt. Then Et_3N (1.13 mL, 8.1 mmol, 60 eq) and 1,3-propanedithiol (0.81 mL, 8.1 mmol, 60 eq) were added under nitrogen in the dark. The resulting mixture was stirred overnight at room temperature. After TLC showed complete consumption of the starting material, the reaction mixture was concentrated and co-evaporated by toluene. The residue was dissolved in THF (3 ml) and water (1.5 mL), then sodium bicarbonate (230 mg, 2.74 mmol, 20 eq) and acetic anhydride (250 µL, 2.74 mmol, 20 eq) were added and stirred for overnight. After TLC showed complete consumption of the starting material, the reaction mixture was diluted with EtOAc and then washed with saturated aqueous sodium bicarbonate and brine. The aqueous layer was extracted with EtOAc (3x), dried with $MgSO_4$, filtered, and concentrated *in vacuo*. The compound was purified by flash chromatography (DCM/Acetone 1:1 – DCM/Acetone/MeOH 10:10:0.5) to yield compound **39** (75.4 mg, 0.073 mmol, 54%). ^{1}H NMR (500 MHz, $CDCl_3$) δ 8.12 – 7.99 (m, 2H), 7.66 – 7.57 (m, 1H), 7.53 – 7.45 (m, 2H), 7.36 – 7.28 (m, 2H), 7.06 – 6.98 (m, 1H), 6.98 – 6.92 (m, 2H), 6.88 (d, *J* = 8.9 Hz, 1H), 6.81 (d, *J* = 9.3 Hz, 1H), 6.19 (d, *J* = 8.9 Hz, 1H), 5.62 – 5.48 (m, 1H), 5.12 – 4.97 (m, 2H), 4.96 – 4.83 (m, 2H), 4.64 – 4.25 (m, 9H), 4.23 – 4.12 (m, 1H), 4.07 – 3.94 (m, 2H), 3.83 (s, 3H), 3.74 – 3.65 (m, 1H), 3.64 – 3.45 (m, 5H), 3.38 – 3.28 (m, 1H), 2.82 – 2.71 (m, 1H), 2.70 – 2.50 (m, 2H), 2.49 – 2.37 (m, 2H), 2.16 (s, 3H), 2.01 (s, 3H), 1.88 (s, 3H), 1.64 (s, 3H), 1.12 (d, *J* = 6.5 Hz, 3H). ^{13}C NMR (126 MHz, $CDCl_3$) δ 206.97, 172.67, 171.45, 170.66, 170.19, 169.36, 165.03, 157.10, 133.61, 129.82, 129.72, 129.63, 128.64, 122.29, 114.88, 100.42, 98.86, 97.96, 94.47, 81.21, 78.45, 75.32, 73.75, 69.95, 69.85, 69.49, 67.50, 66.98, 66.61, 65.62, 65.24, 61.18, 55.67, 52.97, 50.58, 50.00, 47.69, 37.80, 29.73, 28.21, 25.88, 23.28, 22.77, 16.29. HR-MS: Calculated for $C_{49}H_{61}N_3O_{21}$ $[M+NH_4]^+$: 1045.4136, found: 1045.4132. $[\alpha]^{20}_D$ = + 41.0° (c = 0.1, $CHCl_3$). TLC: Rf = 0.2 (DCM/Acetone = 1/1, v/v).

Allyl 2-azido-2-deoxy-4-*O*-(2-azido-3-*O*-levulinoyl-6-deoxy-4-*N*-2,2,2-trichloroethyl-α-D-galactopyranoside)-3,6-*O*-di-*tert*-butylsilylidene-α-D-galactopyranoside (40)

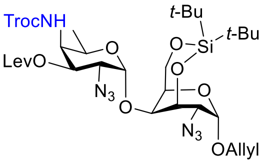

Donor **16** (687 mg, 1.09 mmol, 1.5 eq) and acceptor **14** (279 mg, 0.72 mmol, 1.0 eq) were co-evaporated with toluene (3×) and placed under a nitrogen atmosphere. Dry DCM (8.0 mL) and 4Å molecular sieves were added and the solution stirred for 30 minutes before being cooled to 0 °C. Trifluoromethanesulfonic acid (TfOH) (13 µL, 0.15 mmol, 0.2 eq) was added to the reaction. The solution was stirred for 8h. After analysis by TLC showed complete consumption of the starting material, the reaction was quenched with triethylamine (150 µL, 1.08 mmol, 1.5 eq) and concentrated *in vacuo*. The compound was purified by flash chromatography (Pentane/EA 7:1 - 5:1) to yield compound **40** (534 mg, 0.64 mmol, 89%).^{1}H NMR (400 MHz, $CDCl_3$) δ 6.02 – 5.83 (m, 1H, CH-Allyl), 5.42 (d, *J* = 4.8 Hz, 1H, H-1b), 5.39 – 5.32 (m, 1H, Allyl), 5.31 – 5.16 (m, 3H, Allyl, NH, H-3c), 4.91 (d, *J* = 4.1 Hz, 1H, H-1c), 4.83 – 4.70 (m, 2H, Troc), 4.53 – 4.43 (m, 2H, H-4b, H-6b), 4.42 – 4.30 (m, 2H, H-5c, Allyl), 4.26 – 4.08 (m, 4H, H-4c, H-3b, Allyl, H-6b), 4.05 – 3.98 (m, 1H, H-5b), 3.87 (dd, *J* = 4.8, 1.7 Hz, 1H, H-2b), 3.71 (dd, *J* = 11.2, 4.1 Hz, 1H, H-2c), 2.90 – 2.47 (m, 4H, Lev), 2.19 (s, 3H, Lev), 1.25 (d, *J* = 6.5 Hz, 3H, H-6c), 1.09 (s, 9H, *t*-Bu), 1.01 (s, 9H, *t*-Bu). ^{13}C NMR (101 MHz, $CDCl_3$) δ 206.32 (Lev), 171.81 (Lev), 155.11 (Troc), 133.42 (CH-Allyl), 117.86 (Allyl), 99.49 (C-1c, J_{CH} = 171 Hz), 95.61 (Troc), 94.84 (C-1b, J_{CH} = 168 Hz), 76.03 (C-4b), 74.95 (C-3b), 74.73 (Troc), 72.26 (C-5b), 70.81 (C-3c), 69.79 (Allyl), 65.28 (C-5c), 64.36 (C-2b), 64.20 (C-6b), 58.08 (C-2c), 53.11 (C-4c), 37.97 (Lev), 29.91 (Lev), 28.62 (*t*-Bu), 28.47 (*t*-Bu), 27.92 (Lev), 22.39 (*t*-Bu), 21.22 (*t*-Bu), 16.42 (C-6c). HR-MS: Calculated for $C_{31}H_{48}Cl_3N_7O_{11}Si$ $[M+Na]^+$: 850.2139, found: 850.2137. $[\alpha]^{20}_D$ = + 121.7° (c = 1, $CHCl_3$). TLC: Rf = 0.5 (Pentane/EA = 4/1, v/v).

(2R,3R,4S,5S,6R)-3-azido-2-(((4R,5S,6R)-4-(1-azido-2-oxoethyl)-2,2-di-tert-butyl-6-hydroxy-1,3,2-dioxasilepan-5-yl)oxy)-6-methyl-5-(((2,2,2-trichloroethoxy)carbonyl)amino)tetrahydro-2H-pyran-4-yl 4-oxopentanoate (41a)

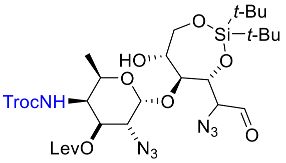

Compound **40** (2.14 mg, 2.59 mmol, 1.0 eq) was dissolved in freshly distilled THF (25 mL). The mixture was degassed and placed under an argon atmosphere. (1,5-Cyclooctadiene) (pyridine)-(tricyclohexylphosphine)-iridium(I) hexafluorophosphate ($Ir(COD)(Ph_2MeP)_2{\cdot}PF_6$) (110 mg, 0.13 mmol, 0.05 eq) was added and the reaction mixture was degassed and then purged with H_2 for 30 seconds. The reaction mixture was stirred for 2 hours under an argon atmosphere. After analysis by TLC showed complete consumption of the starting material, the mixture was diluted with THF, water (5 mL) and *N*-iodosuccinimide (NIS) (874 mg, 3.88 mmol, 1.5 eq) were added, and the solution stirred for 2 hours at room

temperature. After analysis by TLC showed complete consumption of the starting material, EtOAc was added and the organic layer was washed two times with saturated aqueous sodium thiosulphate and brine. The organic layer was dried over $MgSO_4$ and concentrated *in vacuo*. Column chromatography (Pentane/Acetone 6:1 - 4:1) yielded **41a** (1.99 g, 2.52 mmol, 97%). ^{1}H NMR (500 MHz, $CDCl_3$) δ 9.83 (s, 1H, CHO), 5.41 – 5.25 (m, 2H, NH, H-1c), 5.19 (dd, *J* = 11.1, 3.7 Hz, 1H, H-3c), 4.82 – 4.66 (m, 3H, Troc, H-3b), 4.58 (d, *J* = 2.5 Hz, 1H, 5b-OH), 4.28 – 4.15 (m, 2H, H-4c, H-5c), 4.04 – 3.87 (m, 4H, H-2b, H-6b, H-2c), 3.86 – 3.79 (m, 1H, H-5b), 3.70 (t, *J* = 8.9 Hz, 1H, H-4b), 2.93 – 2.44 (m, 4H, Lev), 2.19 (s, 3H, Lev), 1.19 (d, *J* = 6.4 Hz, 3H, H-6c), 1.05 – 0.95 (m, 18H, *t*-Bu). ^{13}C NMR (126 MHz, $CDCl_3$) δ 206.32 (Lev), 196.57 (CHO), 172.14 (Lev), 155.19 (Troc), 99.86 (C-1c, J_{CH} = 173l Hz), 95.58 (Troc), 86.82 (C-4b), 76.16 (C-5b), 74.76 (Troc), 73.33 (C-3b),, 71.91 (C-3c), 68.92 (C-2b), 65.95 (C-5c), 65.56 (C-6b), 58.97 (C-2c), 52.96 (C-4c), 37.86 (Lev), 29.89 (Lev), 28.10 (*t*-Bu), 27.98 (Lev), 27.82 (*t*-Bu), 21.62 (*t*-Bu), 16.50 (C-6c). HR-MS: Calculated for $C_{28}H_{44}Cl_3N_7O_{11}Si$ $[M+Na]^+$: 810.1826, found: 810.1819. $[\alpha]^{20}_{D}$ = + 52.1° (c = 1, $CHCl_3$). TLC: Rf = 0.5 (Pentane/Acetone = 4/1, v/v).

***N*-phenyl-trifluoroacetimidoyl 2-azido-2-deoxy-4-*O*-(2-azido-3-*O*-levulinoyl-6-deoxy-4-*N*-2,2,2-trichloroethyl-α-D-galactopyranoside)-3,6-*O*-di-*tert*-butylsilylidene-α/β-D-galactopyranoside (41)**

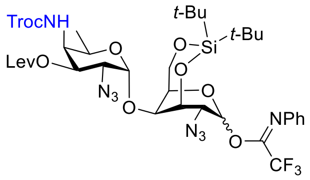

Compound **41a** (336 mg, 0.43 mmol, 1.0 eq) was dissolved in acetone (5.0 mL) and cooled to 0 °C. *N*-phenyl trifluoroacetimidoyl chloride (132 mg, 0.64 mmol, 1.5 eq) and cesium carbonate (170 mg, 0.52 mmol, 1.2 eq) were added. The solution was allowed to warm to RT and stirred overnight. After analysis by TLC showed complete consumption of the starting material, the reaction was quenched with triethyl amine, filtered, and concentrated *in vacuo*. The compound was purified by flash chromatography (Pentane/EA 6:1 - 4:1) to yield compound **41** (336 mg, 0.35 mmol, 82%).^{1}H NMR (500 MHz, Acetone) δ 7.41 – 7.32 (m, 2H), 7.19 – 7.11 (m, 1H), 7.04 (d, *J* = 10.0 Hz, 1H, NH), 6.90 (d, *J* = 7.7 Hz, 2H), 5.92 (s, 1H, H-1b), 5.35 – 5.21 (m, 1H, H-3c), 5.14 (s, 1H, H-1c), 4.91 – 4.76 (m, 2H, Troc), 4.63 – 4.07 (m, 9H, H-6b, H-5c, H-4c, H-2c), 2.89 – 2.68 (m, 2H, Lev), 2.64 – 2.43 (m, 2H, Lev), 2.13 (s, 3H, Lev), 1.23 (d, *J* = 6.4 Hz, 3H, H-6c), 1.13 – 1.01 (m, 18H, *t*-Bu). ^{13}C NMR (126 MHz, Acetone) δ 206.15 (Lev), 172.15 (Lev), 156.47 (Troc), 144.28 (C=N), 129.74, 125.34, 120.03, 99.84, 99.55 (C-1c), 97.01 (Troc), 96.77 (C-1b), 77.66 (C-4b), 75.97 (C-5b), 75.78, 74.96 (Troc), 74.79 (C-3b), 71.32, 71.22 (C-3c), 68.14 (C-2b), 66.33, 66.25 (C-5c), 64.06 (C-6b), 59.08,

58.98 (C-2c), 54.08 (C-4c), 53.98, 38.13, 29.60, 29.11, 29.02, 28.82, 28.71, 28.64, 22.55, 21.98, 16.65. HR-MS: Calculated for $C_{36}H_{48}Cl_3F_3N_8O_{11}Si$ $[M+NH_4]^+$: 976.2568, found: 976.2564. TLC: Rf = 0.5 (Pentane/Acetone = 5/1, v/v).

Propynyl 3-*O*-(2-azido-4-*O*-(2-azido-3-*O*-levulinoyl-6-deoxy-4-*N*-2,2,2-trichloroethyl-α-D-galactopyranoside)-3,6-*O*-di-*tert*-butylsilylidene-α-D-galactopyranoside)-2-*O*-benzoyl-4,6-di-*O*-[1-(*R*)-(methoxycarbonyl)-ethyldiene]-β-D-galactopyranoside (42)

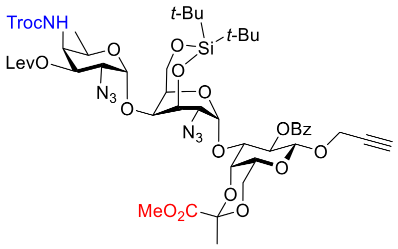

Donor **41** (913 mg, 0.95 mmol, 1.0 eq) and acceptor **29** (630 mg, 1.55 mmol, 1.63 eq) were co-evaporated with toluene (3×) and placed under a nitrogen atmosphere. Dry DCM (10.0 mL) and 4Å molecular sieves were added and the solution stirred for 30 minutes, then cooled to 0 °C. TBSOTf (22.0 µL, 0.096 mmol, 0.1 eq) was added to the reaction mixture and the solution was stirred for 2 hours. After analysis by TLC showed complete consumption of the starting material, the reaction was quenched with triethylamine and concentrated *in vacuo*. The compound was purified by flash chromatography (Pentane/EA 4:1 - 2:1) to yield compound **42** (939 g, 0.8 mmol, 84%). ^{1}H NMR (500 MHz, $CDCl_3$) δ 8.18 – 8.10 (m, 2H), 7.78 – 7.70 (m, 1H), 7.61 – 7.52 (m, 2H), 5.67 (dd, *J* = 10.0, 8.1 Hz, 1H, H-2a), 5.49 (d, *J* = 6.2 Hz, 1H, H-1b), 4.94 – 4.83 (m, 3H, H-3c, H-1a, Troc), 4.81 (d, *J* = 9.3 Hz, 1H, NH), 4.74 (d, *J* = 12.0 Hz, 1H, Troc), 4.50 – 4.41 (m, 2H, H-4a, propynyl), 4.39 – 4.31 (m, 2H, H-5b, propynyl), 4.27 (d, *J* = 12.6 Hz, 1H, H-6b), 4.17 (dd, *J* = 12.9, 1.6 Hz, 1H, H-6a), 4.11 – 3.91 (m, 7H, H-3a, H-5c, H-4c, H-3b, H-6b, H-6a, H-4b), 3.87 (d, *J* = 6.2 Hz, 1H, H-2b), 3.82 (s, 3H, COOMe), 3.76 (d, *J* = 4.3 Hz, 1H, H-1c), 3.51 – 3.45 (m, 1H, H-5a), 2.96 (dd, *J* = 11.1, 4.2 Hz, 1H, H-2c), 2.85 – 2.66 (m, 2H, Lev), 2.64 – 2.44 (m, 2H, Lev), 2.34 (t, *J* = 2.4 Hz, 1H, CH-propynyl), 2.18 (s, 3H, Lev), 1.62 (s, 3H, Me), 1.08 (d, *J* = 6.5 Hz, 3H, H-6c), 1.01 – 0.92 (m, 18H, *t*-Bu). ^{13}C NMR (126 MHz, $CDCl_3$) δ 206.33 (Lev), 171.77 (Lev), 170.48 (COOMe), 164.60 (Bz), 154.88 (Troc), 133.35, 130.50, 128.42, 99.07 (C_{quart}), 98.35 (C-1a, J_{CH} = 160 Hz), 98.07 (C-1c, J_{CH} = 172 Hz), 95.78 (Troc), 91.52 (C-1b, J_{CH} = 173 Hz), 78.69 (propynyl), 75.25 (propynyl), 74.79 (C-3b), 74.77 (Troc), 74.51 (C-5b), 74.21 (C-3a), 71.02 (C-3c), 70.78 (C-4b), 69.94 (C-2a), 66.43 (C-4a), 65.85 (C-5a), 65.37 (C-6a), 64.76 (C-5c), 64.19 (C-2b), 63.08 (C-6b), 57.88 (C-2c), 55.66 (propynyl), 52.94 (C-4c), 52.81 (COOMe), 37.94 (Lev), 29.89 (Lev), 28.40 (*t*-Bu), 28.18 (*t*-Bu), 27.90 (Lev), 25.63 (Me), 22.02 (*t*-Bu), 21.32 (*t*-Bu), 16.31 (C-6c). HR-MS: Calculated for $C_{48}H_{64}Cl_3N_7O_{19}Si$ $[M+NH_4]^+$: 1193.3430, found: 1193.3430. $[\alpha]^{20}_D$ = + 127.4° (c = 1, $CHCl_3$). TLC: Rf = 0.3 (Pentane/EA = 2/1, v/v).

Propynyl 3-*O*-(2-azido-4-*O*-(2-azido-3-*O*-levulinoyl-6-deoxy-4-*N*-2,2,2-trichloroethyl-α-D-galactopyranoside)-α-D-galactopyranoside)-2-*O*-benzoyl-4,6-di-*O*-[1-(*R*)-(methoxycarbonyl)-ethyldiene]-β-D-galactopyranoside (43)

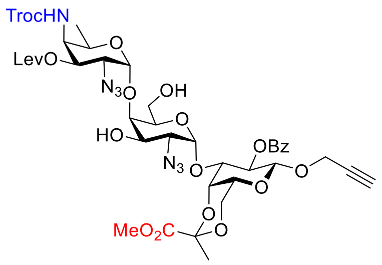

Compound **42** (169 mg, 0.144 mmol, 1.0 eq) was dissolved in THF (1.0 mL) and pyridine (1.0 mL), then cooled to 0 °C. Hydrogen fluoride (HF)/pyridine (70%) (0.1 mL) was added dropwise. The solution was stirred for 3 hours. After TLC showed complete consumption of the starting material, the reaction was quenched with saturated aqueous sodium bicarbonate slowly and diluted with EtOAc. The solution was washed with water (2x) and brine. The aqueous layer was extracted with EtOAc (3x), dried with $MgSO_4$, filtered, and concentrated *in vacuo*. The compound was purified by flash chromatography (Pentane/Acetone 2:1 – 3:2) to yield compound **43** (145 mg, 0.14 mmol, 97%). ^{1}H NMR (400 MHz, $CDCl_3$) δ 8.14 – 8.03 (m, 2H), 7.65 – 7.55 (m, 1H), 7.47 (t, *J* = 7.8 Hz, 2H), 5.57 (dd, *J* = 10.0, 8.1 Hz, 1H, H-2a), 5.49 (d, *J* = 9.4 Hz, 1H, NH), 5.25 – 5.13 (m, 2H, H-1b, H-3c), 4.92 – 4.85 (m, 2H, H-1c, H-1a), 4.79 (d, *J* = 12.1 Hz, 1H, Troc), 4.70 (d, *J* = 12.2 Hz, 1H, Troc), 4.61 – 4.51 (m, 1H, H-5c), 4.49 – 4.31 (m, 3H, propynyl, H-4a), 4.25 – 4.11 (m, 2H, H-4c, H-6a), 4.09 – 3.98 (m, 2H, H-6a, H-3a), 3.97 – 3.90 (m, 1H, H-3b), 3.84 (s, 3H, COOMe), 3.80 – 3.74 (m, 1H, H-4b), 3.72 – 3.64 (m, 2H, H-2c, H-5b), 3.64 – 3.54 (m, 1H, H-6b), 3.54 – 3.44 (m, 2H, H-6b, H-5a), 3.31 – 3.18 (m, 2H, 3b-OH, H-2b), 2.91 – 2.43 (m, 4H, Lev), 2.37 (t, *J* = 2.4 Hz, 1H, CH-propynyl), 2.19 (s, 3H, Lev), 1.99 – 1.92 (m, 1H, 6b-OH), 1.66 (s, 3H, Me), 1.15 (d, *J* = 6.5 Hz, 3H, H-6c). ^{13}C NMR (101 MHz, $CDCl_3$) δ 206.62 (Lev), 172.16 (Lev), 170.45 (COOMe), 165.21 (Bz), 155.23 (Troc), 133.53, 129.86, 129.82, 128.67, 99.03 (C-1c, J_{CH} = 171 Hz), 98.99 (C_{quart}), 98.24 (C-1a, J_{CH} = 160 Hz), 95.60 (Troc), 95.08 (C-1b, J_{CH} = 171 Hz), 78.94 (C-4b), 78.64 (propynyl), 75.33 (propynyl), 74.66 (Troc), 74.37 (C-3a), 70.99 (C-3c), 70.73 (C-5b), 69.94 (C-2a), 67.06 (C-3b), 67.02 (C-4a), 65.87 (C-5a), 65.71 (C-5c), 65.31 (C-6a), 60.20 (C-6b), 60.16 (C-2b), 58.37 (C-2c), 55.69 (propynyl), 52.99 (C-4c), 52.91 (COOMe), 37.83 (Lev), 29.89 (Lev), 27.96 (Lev), 25.67 (Me), 16.42 (C-6c). HR-MS: Calculated for $C_{40}H_{48}Cl_3N_7O_{19}$ $[M+NH_4]^+$: 1053.2409, found: 1053.2403. $[\alpha]^{20}_D$ = + 128.7° (c = 1, $CHCl_3$). TLC: Rf = 0.15 (Pentane/Acetone = 2/1, v/v).

Propynyl 3-*O*-(2-azido-4-*O*-(2-azido-6-deoxy-4-*N*-2,2,2-trichloroethyl-α-D-galactopyranoside)-α-D-galactopyranoside)-2-*O*-benzoyl-4,6-di-*O*-[1-(*R*)-(methoxycarbonyl)-ethyldiene]-β-D-galactopyranoside (45)

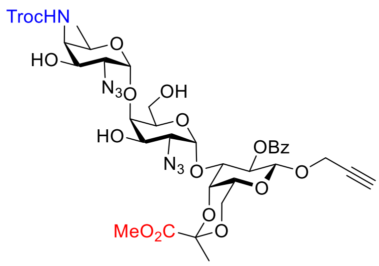

Compound **43** (96 mg, 0.093 mmol, 1.0 eq) was dissolved in pyridine (1.0 mL) and acetic acid (1.0 mL). After cooling to 0 °C, hydrazine acetate (N_2H_4 • AcOH) (26 mg, 0.28 mmol, 3.0 eq) was added. After stirring 5 hours at RT, TLC showed complete consumption of the starting material and the reaction was quenched by acetone. The solution was diluted with EtOAc and then washed with water (2x) and brine. The aqueous layer was extracted with DCM (3x), dried with $MgSO_4$, filtered, and concentrated *in vacuo*. The compound was purified by flash chromatography (PE/Acetone 2:1 – 3:2) to yield compound **45** (67.7 mg, 0.072 mmol, 78%). ^{1}H NMR (400 MHz, $CDCl_3$) δ 8.12 – 8.03 (m, 2H), 7.65 – 7.55 (m, 1H), 7.48 (t, *J* = 7.6 Hz, 2H), 5.69 – 5.51 (m, 2H, NH, H-2a), 5.24 (d, *J* = 3.4 Hz, 1H, H-1b), 4.93 – 4.79 (m, 3H, H-1a, Troc, H-1c), 4.65 (d, *J* = 12.1 Hz, 1H, Troc), 4.51 – 4.31 (m, 4H, propynyl, H-5c, H-4a), 4.23 – 4.11 (m, 2H, H-6a, H-3c), 4.09 – 3.89 (m, 4H, H-6a, H-3a, H-4c, H-3b), 3.83 (s, 3H, COOMe), 3.78 – 3.73 (m, 1H, H-4b), 3.72 – 3.65 (m, 1H, H-5b), 3.64 – 3.40 (m, 6H, H-6b, H-2c, 3b-OH, H-5a), 3.19 (dd, *J* = 11.1, 3.4 Hz, 1H, H-2b), 2.45 (s, 1H), 2.38 (t, *J* = 2.4 Hz, 1H, CH-propynyl), 1.66 (s, 3H, COOMe), 1.17 (d, *J* = 6.4 Hz, 3H, H-6c). ^{13}C NMR (101 MHz, $CDCl_3$) δ 170.55 (COOMe), 165.29 (Bz), 156.50 (Troc), 133.57, 129.80, 128.67, 99.33 (C-1c, J_{CH} = 170 Hz), 98.95 (C_{quart}), 98.22 (C-1a, J_{CH} = 159 Hz), 95.40 (Troc), 95.11 (C-1b, J_{CH} = 173 Hz), 79.87 (C-4b), 78.63 (propynyl), 75.39 (propynyl), 74.88 (Troc), 74.33 (C-3a), 70.65 (C-5b), 69.97 (C-2a), 69.18 (C-3c), 67.08 (C-3b), 66.93 (C-5c), 66.24 (C-4a), 65.84 (C-5a), 65.34 (C-6a), 61.19 (C-2c), 60.51 (C-6b), 60.24 (C-2b), 56.09 (C-4c), 55.71 (propynyl), 52.89 (COOMe), 25.65 (Me), 16.56 (C-6c). HR-MS: Calculated for $C_{35}H_{42}Cl_3N_7O_{17}$ $[M+NH_4]^+$: 955.2041, found: 955.2036. $[\alpha]^{20}_D$ = + 178.0° (c = 0.1, $CHCl_3$). TLC: Rf = 0.3 (PE/Acetone = 3/2, v/v).

Propynyl 3-*O*-(2-acetamido-2-deoxy-4-*O*-(2-acetamido-2-deoxy-6-deoxy-4-*N*-2,2,2-trichloroethyl-α-D-galactopyranoside)-α-D-galactopyranoside)-2-*O*-benzoyl-4,6-di-*O*-[1-(*R*)-(methoxycarbonyl)-ethyldiene]-β-D-galactopyranoside (1ba)

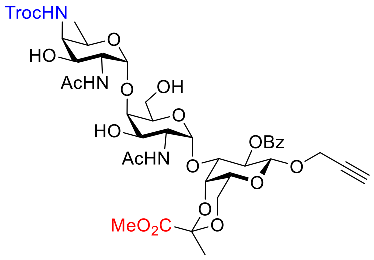

Compound **45** (12.4 mg, 0.013 mmol, 1.0 eq) was dissolved in pyridine (0.5 mL) and thioacetic acid (0.5 mL). The resulting mixture was stirred overnight at room temperature. After TLC showed complete consumption of the starting material, the reaction mixture was concentrated and co-evaporated by toluene *in vacuo*. The compound was purified by flash chromatography (DCM/MeOH 40:1 –

10:1) to yield compound **1ba** (9.3 mg, 0.01 mmol, 73%). ^{1}H NMR (500 MHz, $CDCl_3$) δ 8.12 – 8.00 (m, 2H), 7.67 – 7.59 (m, 1H), 7.49 (t, J = 7.8 Hz, 2H), 6.19 (d, J = 9.2 Hz, 1H, 2c-NHAc), 5.79 (d, J = 9.8 Hz, 1H, NH), 5.54 (dd, J = 10.0, 8.1 Hz, 1H, H-2a), 5.09 – 5.03 (m, 1H, H-1c), 4.94 – 4.85 (m, 2H, Troc, H-1a), 4.81 (d, J = 3.9 Hz, 1H, H-1b), 4.59 (d, J = 12.1 Hz, 1H, Troc), 4.52 – 4.42 (m, 2H, H-5c, propynyl), 4.42 – 4.28 (m, 3H, propynyl, H-4a, H-2c), 4.21 – 4.03 (m, 4H, H-6a, H-4c, H-2b), 4.02 – 3.90 (m, 3H, H-3a, H-6a, H-3b), 3.89 – 3.82 (m, 1H), 3.79 (s, 3H, COOMe), 3.63 (d, J = 2.6 Hz, 1H, H-4b), 3.61 – 3.43 (m, 5H, H-3c, H-5b, H-6b, H-5a), 3.37 – 3.28 (m, 1H, H-6b), 2.39 (t, J = 2.4 Hz, 1H, CH-propynyl), 2.01 (s, 3H, NHAc), 1.91 (s, 3H, NHAc), 1.64 (s, 3H, COOMe), 1.26 – 1.23 (m, 3H, H-6c). ^{13}C NMR (126 MHz, $CDCl_3$) δ 172.69 (NHAc), 171.63 (NHAc), 170.41 (COOMe), 165.19 (Bz), 156.26 (Troc), 133.76, 129.92, 129.81, 128.82, 124.91, 100.22 (C-1b), 99.00 (C_{quart}), 98.13 (C-1a), 95.70 (Troc), 94.78 (C-1c), 80.73 (C-4b), 78.63 (propynyl), 75.43 (propynyl), 74.80 (Troc), 74.07 (C-3a), 70.02 (C-3b), 69.83 (C-5b), 69.67 (C-3c), 69.40 (C-2a), 67.35 (C-5c), 67.16 (C-4a), 65.80 (C-5a), 65.44 (C-6a), 60.99 (C-6b), 56.16 (C-4c), 55.84 (propynyl), 53.08 (COOMe), 51.02 (C-2b), 50.10 (C-2c), 26.05 (Me), 23.46 (NHAc), 22.84 (NHAc), 16.59 (C-6c). HR-MS: Calculated for $C_{39}H_{50}Cl_3N_3O_{19}$ $[M+H]^+$: 970.2177, found: 970.2169. $[\alpha]^{20}_{D}$ = + 78.0° (c = 0.1, $CHCl_3$). TLC: Rf = 0.2 (DCM/MeOH = 40/1, v/v).

Propynyl 3-*O*-(2-acetamido-2-deoxy-4-*O*-(2-acetamido-2-deoxy-4-amino-6-deoxy-α-D-galactopyranoside)-α-D-galactopyranoside)-4,6-di-*O*-[1-(*R*)-(carboxyl)-ethyldiene]-β-D-galactopyranoside (1b)

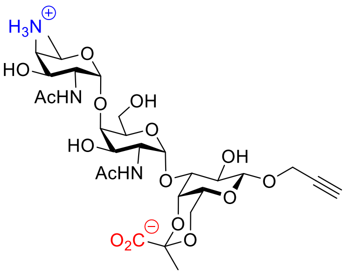

Partially protected trimer **1ba** (14 mg, 14.4 μmol, 1.0 eq) was dissolved in dioxane (1.5 mL), THF (1.5 mL), water (1 mL) and 1M NaOH (1 mL). The mixture was stirred at RT overnight. After analysis by TLC and LC-MS showed complete consumption of the starting material and intermediates, the reaction was quenched with acetic acid and then quenched the excess acid using ammonia solution. The solvent was removed through co-evaporation with toluene *in vacuo*. The compound was purified by gel filtration (HW-40, 0.15M NH_4HCO_3 in H_2O) with a Shimadzu RID-10A refractive index detector and lyophilized to yield compound **1b** (5.6 mg, 8.26 μmol, 57%). ^{1}H NMR (500 MHz, D_2O) δ 5.32 (d, J = 3.7 Hz, 1H, H-1b), 4.93 (d, J = 4.0 Hz, 1H, H-1c), 4.77 – 4.76 (m, 1H, H-5c), 4.67 (d, J = 7.8 Hz, 1H, H-1a), 4.52 – 4.42 (m, 3H, propynyl, H-4a), 4.42 – 4.32 (m, 2H, H-2b, H-3c), 4.19 – 4.10 (m, 2H, H-5b, H-3b), 4.06 – 3.92 (m, 4H, H-4b, H-2c, H-6a), 3.79 (dd, J = 9.9, 3.7 Hz, 1H, H-3a), 3.75 – 3.64 (m, 3H, H-2a, H-6b), 3.64 – 3.61 (m, 1H, H-4c),

3.60 – 3.56 (m, 1H, H-5a), 2.88 (t, J = 2.4 Hz, 1H, CH-propynyl), 2.07 (s, 3H, NHAc), 2.04 (s, 3H, NHAc), 1.44 (s, 3H, Me), 1.21 (d, J = 6.7 Hz, 3H, H-6c). ^{13}C NMR (126 MHz, D_2O) δ 175.17 (COOH), 174.85 (2b-NHAc), 174.66 (2c-NHAc), 100.99 (C_{quart}), 100.32 (C-1a), 98.14 (C-1c), 93.82 (C-1b), 78.69 (propynyl), 77.55 (C-4b), 76.37 (propynyl), 74.95 (C-3a), 71.78 (C-5b), 68.66 (C-2a), 67.24 (C-4a), 67.15 (C-3b), 66.22 (C-5a), 64.91 (C-6a), 63.91 (C-3c), 63.22 (C-5c), 60.15 (C-6b), 56.32 (propynyl), 55.37 (C-4c), 49.74 (C-2c), 49.46 (C-2b), 25.08 (Me), 22.27 (NHAc), 21.90 (NHAc), 15.49 (C-6c). HR-MS: Calculated for $C_{28}H_{43}N_3O_{16}$ $[M+H]^+$: 678.2716, found: 678.2711.

Propynyl 3-*O*-(2-azido-4-*O*-(2-azido-3-*O*-levulinoyl-6-deoxy-4-*N*-2,2,2-trichloroethyl-α-D-galactopyranoside)-6-*O*-benzoyl-α-D-galactopyranoside)-2-*O*-benzoyl-4,6-di-*O*-[1-(*R*)-(methoxycarbonyl)-ethyldiene]-β-D-galactopyranoside (44)

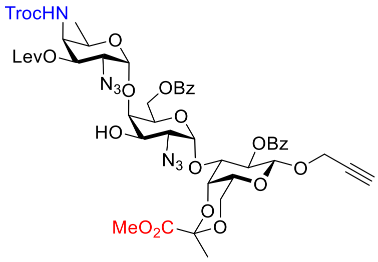

Compound **43** (167 mg, 0.161 mmol, 1.0 eq) was dissolved in DCM (4.0 mL). Et_3N (135 μL, 0.97 mmol, 6.0 eq) and BzOBt (193 mg, 0.81 mmol, 5.0 eq) were added and the mixture was stirred at RT overnight. The reaction was quenched by MeOH and concentrated *in vacuo*. The compound was purified by flash chromatography (Pentane/Acetone 3:1 - 3:2) to yield compound **44** (167 mg, 0.147 mmol, 91%). ^{1}H NMR (500 MHz, $CDCl_3$) δ 8.21 – 7.81 (m, 4H), 7.72 – 7.40 (m, 3H), 7.38 – 7.07 (m, 3H), 5.69 – 5.50 (m, 1H, H-2a), 5.45 – 5.13 (m, 3H, H-1b, NH, H-3c), 4.93 – 4.56 (m, 5H, H-1c, H-1a, Troc, H-5c), 4.52 – 4.28 (m, 5H, H-4a, H-6b, propynyl), 4.27 – 4.12 (m, 2H, H-4c, H-6a), 4.11 – 3.77 (m, 8H, H-3a, H-6a, H-5b, H-3b, COOMe, H-4b), 3.60 (dd, J = 11.1, 4.0 Hz, 1H, H-2c), 3.48 – 3.31 (m, 2H, H-2b, H-5a), 2.91 – 2.45 (m, 4H, Lev), 2.30 (t, J = 2.4 Hz, 1H, CH-propynyl), 2.25 – 2.12 (m, 3H, Lev), 1.65 (s, 3H, Me), 1.15 (d, J = 6.5 Hz, 3H, H-6c). ^{13}C NMR (126 MHz, $CDCl_3$) δ 206.42 (Lev), 172.13 (Lev), 170.41 (COOMe), 165.50 (6b-Bz), 165.07 (2a-Bz), 155.13 (Troc), 133.55, 133.49, 133.30, 130.20, 129.67, 129.61, 129.50, 128.65, 128.52, 128.47, 99.40 (C-1c, J = 170 Hz), 99.02 (C_{quart}), 98.36 (C-1a, J = 160 Hz), 95.56 (Troc), 94.00 (C-1b, J = 173 Hz), 78.65 (propynyl), 78.54 (C-4b), 75.24 (propynyl), 74.68 (Troc), 73.63 (C-3a), 70.89 (C-3c), 69.65 (C-2a), 68.74 (C-5b), 67.01 (C-3b), 66.41 (C-4a), 65.83 (C-5c), 65.73 (C-5a), 65.31 (C-6a), 61.92 (C-6b), 60.04 (C-2b), 58.26 (C-2c), 55.67 (propynyl), 52.99 (C-4c), 52.92 (COOMe), 37.88 (Lev), 29.87 (Lev), 28.01 (Lev), 25.64 (Me), 16.36 (C-6c). HR-MS: Calculated for $C_{47}H_{52}Cl_3N_7O_{20}$ $[M+NH_4]^+$: 1157.2671, found: 1157.2669. $[\alpha]^{20}_D$ = + 82.0° (c = 0.1, $CHCl_3$). TLC: Rf = 0.4 (Pentane/Acetone = 3/2, v/v).

Propynyl 3-*O*-(2-azido-4-*O*-(2-azido-3-*O*-levulinoyl-6-deoxy-4-*N*-2,2,2-trichloroethyl-α-D-galactopyranoside)-6-*O*-benzoyl-3-*O*-(2,3,5,6-tetra-*O*-benzoyl-β-D-galactofuranosyl)-α-D-galactopyranoside)-2-*O*-benzoyl-4,6-di-*O*-[1-(*R*)-(methoxycarbonyl)-ethyldiene]-β-D-galactopyranoside (46)

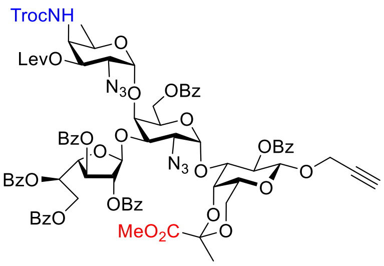

Donor **10** (160 mg, 0.21 mmol, 3.0 eq) and acceptor **44** (79.2 mg, 0.069 mmol, 1.0 eq) were co-evaporated with toluene (3×) and placed under a nitrogen atmosphere. Dry DCM (3.0 mL) and 4Å molecular sieves were added and the solution stirred for 30 minutes before being cooled to 0 °C. TBSOTf (4.0 µL, 0.017 mmol, 0.2 eq) was added to the reaction and stirred 4 hours. After analysis by TLC showed complete consumption of the starting material, the reaction was quenched with triethylamine and concentrated *in vacuo*. The compound was purified by flash chromatography (DCM/Acetone 30:1 - 100:7) to yield compound **46** (88 mg, 0.051 mmol, 73%). ^{1}H NMR (400 MHz, $CDCl_3$) δ 8.17 – 7.94 (m, 10H), 7.91 – 7.82 (m, 2H), 7.64 – 7.14 (m, 19H), 5.96 – 5.84 (m, 1H, H-5d), 5.69 – 5.57 (m, 4H, H-2d, H-2a, H-1d, H-3d), 5.45 – 5.34 (m, 1H, H-1b), 5.15 (dd, *J* = 11.2, 3.9 Hz, 1H, H-3c), 4.94 (d, *J* = 9.8 Hz, 1H, NH), 4.85 – 4.65 (m, 6H, Troc, H-1a, H-1c, H-4d, H-6d), 4.55 – 4.28 (m, 7H, H-6d, H-6b, H-6b, H-5c, H-4a, propynyl), 4.21 – 4.11 (m, 2H, H-4c, H-6a), 4.09 – 3.89 (m, 5H, H-3b, H-5b, H-6a, H-3a, H-4b), 3.83 (s, 3H, COOMe), 3.68 (dd, *J* = 10.9, 3.4 Hz, 1H, H-2b), 3.33 – 3.26 (m, 1H, H-5a), 3.11 (dd, *J* = 11.0, 3.7 Hz, 1H, H-2c), 2.89 – 2.43 (m, 4H, Lev), 2.31 (t, *J* = 2.4 Hz, 1H, CH-propynyl), 2.18 (s, 3H, Lev), 1.63 (s, 3H, Me), 1.17 (d, *J* = 6.5 Hz, 3H, H-6c). ^{13}C NMR (101 MHz, $CDCl_3$) δ 206.39 (Lev), 172.04 (Lev), 170.37 (COOMe), 166.03, 165.75, 165.66, 165.40, 165.07, 155.05 (Troc), 133.74, 133.60, 133.46, 133.45, 133.40, 133.31, 130.09, 130.00, 129.98, 129.94, 129.87, 129.83, 129.81, 129.70, 129.66, 129.62, 129.58, 129.25, 129.08, 128.84, 128.69, 128.60, 128.54, 128.49, 128.44, 107.43 (C-1d, *J* = 175 Hz), 99.33 (C-1c, *J* = 169 Hz), 99.03 (C_{quart}), 98.38 (C-1a, *J* = 160 Hz), 95.66 (Troc), 93.37 (C-1b, *J* = 174 Hz), 81.28 (C-2d), 80.69 (C-4d), 78.69 (propynyl), 78.43 (C-4b), 77.33 (C-3d), 75.20 (propynyl), 74.78 (C-3b), 74.69 (Troc), 73.13 (C-3a), 70.96 (C-3c), 70.23 (C-5d), 69.64 (C-2a), 69.39 (C-5b), 66.21 (C-4a), 65.79 (C-5a), 65.32 (C-6a), 65.23 (C-5c), 62.79 (C-6b), 62.71 (C-6d), 58.75 (C-2b, C-2c), 55.66 (propynyl), 53.10 (C-4c), 52.95 (COOMe), 37.88 (Lev), 29.91 (Lev), 27.98 (Lev), 25.64 (Me), 16.55 (C-6c). HR-MS: Calculated for $C_{81}H_{78}Cl_3N_7O_{29}$ $[M+NH_4]^+$: 1735.4248, found: 1735.4264. $[\alpha]^{20}_D$ = + 61.0° (c = 0.1, $CHCl_3$). TLC: Rf = 0.4 (DCM/Acetone = 19/1, v/v).

Propynyl 3-*O*-(2-azido-4-*O*-(2-azido-6-deoxy-4-*N*-2,2,2-trichloroethyl-α-D-galactopyranoside)-6-*O*-benzoyl-3-*O*-(2,3,5,6-tetra-*O*-benzoyl-β-D-galactofuranosyl)-α-D-galactopyranoside)-2-*O*-benzoyl-4,6-di-*O*-[1-(*R*)-(methoxycarbonyl)-ethyldiene]-β-D-galactopyranoside (47a)

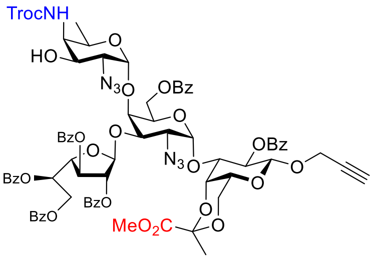

Compound **46** (80 mg, 0.047 mmol, 1.0 eq) was dissolved in pyridine (1.0 mL) and acetic acid (1.0 mL). After cooling to 0 °C, hydrazine acetate ($N_2H_4 \cdot AcOH$) (13 mg, 0.14 mmol, 3.0 eq) was added. After stirring 4 hours at RT, TLC analysis showed complete consumption of the starting material and the reaction was quenched by acetone. The solution was diluted by EtOAc and then washed with water (2x) and brine. The aqueous layer was extracted with DCM (3x), dried with $MgSO_4$, filtered, and concentrated *in vacuo*. The compound was purified by flash chromatography (DCM/Acetone 30:1 – 10:1) to yield compound **47a** (60.1 mg, 0.037 mmol, 80%). ^{1}H NMR (500 MHz, $CDCl_3$) δ 8.20 – 7.91 (m, 10H), 7.91 – 7.77 (m, 2H), 7.68 – 7.09 (m, 19H), 5.93 (s, 1H), 5.74 – 5.52 (m, 4H), 5.42 (s, 1H), 5.07 – 4.28 (m, 14H), 4.26 – 3.57 (m, 12H), 3.26 (s, 1H), 2.98 – 2.67 (m, 2H), 2.31 (t, *J* = 2.4 Hz, 1H), 1.71 – 1.55 (m, 3H), 1.37 – 1.10 (m, 3H). ^{13}C NMR (126 MHz, $CDCl_3$) δ 170.46, 166.08, 165.77, 165.72, 165.45, 165.09, 156.35, 133.81, 133.60, 133.49, 133.44, 133.32, 130.13, 130.00, 129.98, 129.90, 129.74, 129.65, 129.30, 129.08, 128.86, 128.71, 128.62, 128.55, 128.51, 128.48, 107.37, 99.14, 99.03, 98.44, 95.49, 93.26, 81.27, 80.95, 78.75, 77.49, 75.20, 74.95, 74.72, 73.06, 70.31, 69.66, 69.04, 66.11, 65.81, 65.49, 65.41, 62.90, 61.62, 58.94, 56.22, 55.68, 52.86, 25.66, 16.75. HR-MS: Calculated for $C_{76}H_{72}Cl_3N_7O_{27}$ [$M+NH_4^+$]: 1637.3880, found: 1637.3882. $[\alpha]^{20}_D$ = + 63.0° (c = 0.1, $CHCl_3$). TLC: Rf = 0.1 (DCM/Acetone = 19/1, v/v).

Propynyl 3-*O*-(2-acetamido-4-*O*-(2-acetamido-2-deoxy-6-deoxy-4-*N*-2,2,2-trichloroethyl-α-D-galactopyranoside)-6-*O*-benzoyl-3-*O*-(2,3,5,6-tetra-*O*-benzoyl-β-D-galactofuranosyl)-α-D-galactopyranoside)-2-*O*-benzoyl-4,6-di-*O*-[1-(*R*)-(methoxycarbonyl)-ethyldiene]-β-D-galactopyranoside (47)

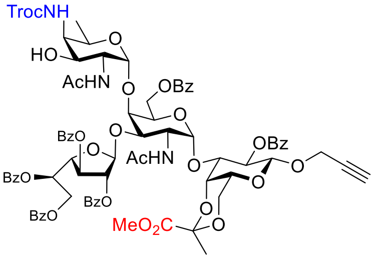

Compound **47a** (59.5 mg, 0.037 mmol, 1.0 eq) was dissolved in pyridine (0.4 mL) and thioacetic acid (0.4 mL). The resulting mixture was stirred overnight at room temperature. After TLC showed complete consumption of the starting material, the reaction mixture was concentrated and co-evaporated by toluene *in vacuo*. The compound was purified by flash chromatography (DCM/MeOH 30:1 –

20:1) to yield compound **47** (24.2 mg, 0.015 mmol, 40%). ^{1}H NMR (500 MHz, $CDCl_3$) δ 8.15 – 7.93 (m, 10H), 7.92 – 7.85 (m, 2H), 7.63 – 7.47 (m, 7H), 7.46 – 7.27 (m, 9H), 7.19 (t, *J* = 7.6 Hz, 2H), 6.47 (s, 1H), 6.08 (d, *J* = 9.8 Hz, 1H), 5.96 – 5.87 (m, 1H), 5.70 – 5.62 (m, 1H), 5.58 (dd, *J* = 10.1, 8.1 Hz, 1H), 5.48 – 5.41 (m, 1H), 5.30 – 5.24 (m, 1H), 5.17 – 5.06 (m, 2H), 4.96 (d, *J* = 3.7 Hz, 1H), 4.86 – 4.77 (m, 3H), 4.77 – 4.69 (m, 2H), 4.63 – 4.34 (m, 6H), 4.27 (d, *J* = 3.6 Hz, 1H), 4.18 – 4.07 (m, 2H), 4.04 – 3.89 (m, 4H), 3.88 – 3.80 (m, 2H), 3.80 – 3.66 (m, 5H), 3.29 – 3.22 (m, 1H), 3.00 (s, 1H), 2.35 (t, *J* = 2.4 Hz, 1H), 2.04 (s, 3H), 1.89 (s, 3H), 1.57 (s, 3H), 1.12 (d, *J* = 6.8 Hz, 3H). ^{13}C NMR (126 MHz, $CDCl_3$) δ 171.62, 170.59, 169.97, 166.02, 165.63, 165.59, 165.57, 165.35, 156.19, 133.70, 133.65, 133.45, 133.42, 133.27, 129.96, 129.90, 129.87, 129.80, 129.66, 129.61, 129.55, 129.46, 129.37, 129.19, 128.83, 128.81, 128.68, 128.57, 128.53, 128.49, 128.45, 128.42, 108.02, 98.94, 98.76, 98.13, 93.06, 81.73, 79.97, 78.63, 77.68, 76.99, 75.81, 75.20, 74.65, 72.60, 70.19, 70.08, 69.54, 68.02, 66.37, 65.68, 65.60, 65.13, 63.00, 56.19, 55.69, 53.02, 51.75, 47.86, 25.81, 23.22, 23.16, 16.68. HR-MS: Calculated for $C_{80}H_{80}Cl_3N_3O_{29}$ $[M+H]^+$: 1652.4016, found: 1652.4013. TLC: Rf = 0.2 (DCM/MeOH = 20/1, v/v).

Propynyl 3-*O*-(2-acetamido-2-deoxy-4-*O*-(2-acetamido-2-deoxy-4-amino-6-deoxy-α-D-galactopyranoside)-3-*O*-(β-D-galactofuranosyl)-α-D-galactopyranoside)-4,6-di-*O*-[1-(*R*)-(carboxyl)-ethyldiene]-β-D-galactopyranoside (1a)

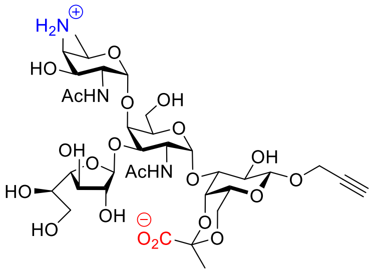

From tetramer **47**: Fully protected tetramer **47** (24 mg, 14.5 µmol, 1.0 eq) was dissolved in dioxane (2.0 mL), THF (2.0 mL), water (2.0 mL) and 1M NaOH (0.6 mL). The mixture was stirred at RT for 24 hours. After analysis by TLC and LC-MS showed complete consumption of the starting material and intermediates, the reaction was quenched with acetic acid and then quenched the excess acid using ammonia solution. The solvent was removed through co-evaporation with toluene *in vacuo*. The compound was purified by gel filtration (HW-40, 0.15M NH_4HCO_3 in H_2O) with a Shimadzu RID-10A refractive index detector and lyophilized to yield compound **1a** (2.8 mg, 3.33 µmol, 23%) and side product **1c** (6.7 mg, 7.74 µmol, 53%).

From tetramer **47a**: Compound **47a** (56.2 mg, 34.7 µmol, 1.0 eq) was dissolved in DCM (1.0 mL) and 3,4-dihydro-2H-pyran (0.1 mL). Then pyridinium *p*-toluenesulfonate (4.5 mg, 17.9 µmol, 0.5 eq) was added at RT. The mixture was stirred at RT overnight. After analysis by TLC showed complete consumption of the starting material, the reaction was quenched with triethylamine, the mixture was concentrated and purified by size exclusion chromatography

(LH-20) (DCM/MeOH 1:1). The crude (45.1 mg) was dissolved in THF (1.0 mL) and water (15 µL). Pyridine (64 µL, 0.80 mmol, 23 eq) and Ph_3P (55 mg, 0.21 mmol, 6.0 eq) were added and the reaction was allowed to stir overnight at 70 °C. After TLC showed complete consumption of the starting material, THF (1.0 ml) and water (1.0 mL) were added to the reaction. Then sodium bicarbonate (444 mg, 5.29 mmol, 152 eq) and acetic anhydride (250 µL, 2.64 mmol, 76 eq) were added and stirred overnight. After TLC showed complete consumption of the starting material, the reaction mixture was diluted with EtOAc and then washed with saturated aqueous sodium bicarbonate and brine. The aqueous layer was extracted with EtOAc (3x), dried with $MgSO_4$, filtered, and concentrated *in vacuo*. The residue was purified by size exclusion chromatography (LH-20) (DCM/MeOH 1:1). Part of the crude (20 mg) was dissolved in dioxane (1.0 mL), THF (1.0 mL), water (1.0 mL). LiOH (15 mg, 0.63 mmol, 0.2M) was added and stirred at RT. After analysis by TLC and LC-MS showed complete consumption of the starting material and intermediates, the reaction was quenched with 1M HCl to neutral pH. Acetic acid (5 mL) was added and heated to 50 °C. After analysis by LC-MS showed complete consumption of the starting material, the solvent was removed through co-evaporation with toluene *in vacuo*. The compound was purified by gel filtration (HW-40, 0.15M NH_4HCO_3 in H_2O) with a Shimadzu RID-10A refractive index detector and lyophilized to yield compound **1a** (8.6 mg, 10.2 µmol, 57% over five steps). ^{1}H NMR (500 MHz, D_2O) δ 5.33 (d, *J* = 3.7 Hz, 1H, H-1b), 5.06 (d, *J* = 3.5 Hz, 1H, H-1d), 5.02 (d, *J* = 3.8 Hz, 1H, H-1c), 4.76 – 4.70 (m, 1H, H-5c), 4.68 (d, *J* = 7.8 Hz, 1H, H-1a), 4.58 (dd, *J* = 11.2, 3.6 Hz, 1H, H-2b), 4.53 – 4.43 (m, 3H, propynyl, H-4a), 4.39 (dd, *J* = 11.5, 4.5 Hz, 1H, H-3c), 4.23 – 4.15 (m, 2H, H-5b, H-4b), 4.11 (dd, *J* = 11.2, 2.5 Hz, 1H, H-3b), 4.08 – 3.91 (m, 6H, H-4d, H-6a, H-3d, H-2d, H-2c), 3.84 – 3.70 (m, 4H, H-3a, H-5d, H-6b, H-2a), 3.69 – 3.56 (m, 5H, H-6b, H-4c, H-6d, H-5a), 2.89 (t, *J* = 2.4 Hz, 1H, CH-propynyl), 2.09 – 2.00 (m, 6H, NHAc), 1.44 (s, 3H, Me), 1.30 (d, *J* = 6.7 Hz, 3H, H-6c). ^{13}C NMR (126 MHz, D_2O) δ 175.29 (COOH), 174.72 (NHAc), 174.69 (NHAc), 108.91 (C-1d), 101.12 (C_{quart}), 100.37 (C-1a), 97.75 (C-1c), 93.91 (C-1b), 81.79 (C-2d), 80.63 (C-3d), 78.73 (propynyl), 77.40 (C-4b), 76.39 (propynyl), 75.65 (C-4d), 75.30 (C-3b), 74.91 (C-3a), 72.06 (C-5b), 70.49 (C-5d), 68.65 (C-2a), 67.18 (C-4a), 66.24 (C-5a), 64.91 (C-6a), 63.69 (C-3c), 63.20 (C-5c), 62.67 (C-6d), 60.30 (C-6b), 56.35 (propynyl), 55.39 (C-4c), 50.09 (C-2c), 48.73 (C-2b), 25.10 (Me), 22.31 (NHAc), 21.91 (NHAc), 16.13 (C-6c). HR-MS: Calculated for $C_{34}H_{53}N_3O_{21}$ $[M+H]^+$: 840.3244, found: 840.3240.

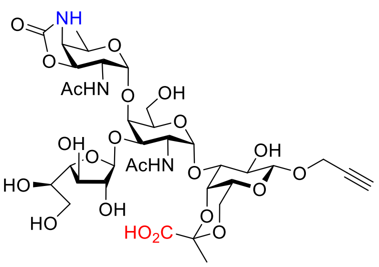

1c: ^{1}H NMR (500 MHz, D_2O) δ 5.31 (d, *J* = 3.6 Hz, 1H, H-1b), 5.04 (d, *J* = 3.1 Hz, 1H, H-1d), 5.03 (d, *J* = 3.6 Hz, 1H, H-1c), 4.97 (dd, *J* = 9.2, 7.2 Hz, 1H, H-3c), 4.68 (d, *J* = 7.8 Hz, 1H, H-1a), 4.66 – 4.60 (m, 1H, H-5c), 4.57 – 4.50 (m, 1H, H-2b), 4.50 – 4.43 (m, 3H, propynyl, H-4a), 4.24 – 4.18 (m, 2H, H-4c, H-5b), 4.16 – 4.07 (m, 3H, H-4b, H-2c, H-3b), 4.05 – 3.93 (m, 5H, H-6a, H-3d, H-2d, H-4d), 3.83 – 3.76 (m, 2H, H-3a, H-5d), 3.76 – 3.61 (m, 5H, H-2a, H-6b, H-6d), 3.61 – 3.58 (m, 1H, H-5a), 2.90 (t, *J* = 2.4 Hz, 1H, CH-propynyl), 2.10 – 2.00 (m, 6H, NHAc), 1.45 (s, 3H, Me), 1.30 (d, *J* = 6.6 Hz, 3H, H-6c). ^{13}C NMR (126 MHz, D_2O) δ 175.28 (COOH), 174.69 (NHAc), 174.62 (NHAc), 161.80 (C=O), 108.98 (C-1d), 101.11 (C_{quart}), 100.39 (C-1a), 97.75 (C-1c), 94.05 (C-1b), 81.68 (C-4d), 80.93 (C-2d), 78.74 (propynyl), 78.38 (C-4b), 76.39 (propynyl), 75.82 (C-3d), 75.24 (C-3b), 75.08 (C-5d), 74.89 (C-3c), 72.01 (C-5b), 70.30 (C-3a), 68.64 (C-2a), 67.28 (C-4a), 66.25 (C-5a), 64.91 (C-6a), 62.78 (C-6d), 62.48 (C-5c), 60.26 (C-6b), 56.36 (propynyl), 55.88 (C-4c), 50.89 (C-2c), 48.69 (C-2b), 25.10 (Me), 22.26 (NHAc), 21.89 (NHAc), 16.71 (C-6c). HR-MS: Calculated for $C_{35}H_{51}N_3O_{22}$ $[M+NH_4]^+$: 883.3302, found: 883.3296.

Phenyl 3-*O*-(2-azido-4-*O*-(2-azido-3-*O*-levulinoyl-6-deoxy-4-*N*-2,2,2-trichloroethyl-α-D-galactopyranoside)-3,6-*O*-di-*tert*-butylsilylidene-α-D-galactopyranoside)-2-*O*-benzoyl-4,6-di-*O*-[1-(*R*)-(methoxycarbonyl)-ethyldiene]-β-D-galactopyranoside (50)

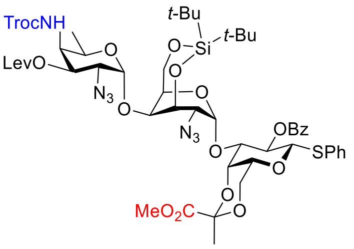

Donor **41** (1.19 g, 1.24 mmol, 1.0 eq) and acceptor **12** (1.7 g, 3.69 mmol, 3.0 eq) were co-evaporated with toluene (3×) and placed under a nitrogen atmosphere. Dry DCM (13.0 mL) and 4Å molecular sieves were added and the solution stirred for 30 minutes, then cooled to 0 °C. TBSOTf (56.0 µL, 0.24 mmol, 0.2 eq) was added to the reaction. The solution was stirred for 2 hours. After analysis by TLC showed complete consumption of the starting material, the reaction was quenched with triethylamine and concentrated *in vacuo*. The compound was purified by flash chromatography (Pentane/EA 5:1 - 3:1) to yield compound **50** (1.25 g, 1.014 mmol, 82%). ^{1}H NMR (500 MHz, $CDCl_3$) δ 8.19 – 8.09 (m, 2H), 7.80 – 7.72 (m, 1H), 7.63 – 7.55 (m, 2H), 7.55 – 7.48 (m, 2H), 7.32 – 7.27 (m, 3H), 5.63 (t, *J* = 9.7 Hz, 1H, H-2a), 5.47 (d, *J* = 6.3 Hz, 1H, H-1b), 4.90 (dd, *J* = 11.1, 3.6 Hz, 1H, H-3c), 4.87 – 4.78 (m, 3H, H-1a, Troc, NH), 4.75 (d, *J* = 12.0 Hz, 1H, Troc), 4.51 – 4.46 (m, 1H, H-4a), 4.29 (dd, *J* = 7.7, 2.5 Hz, 1H, H-4b), 4.26 – 4.17 (m, 2H, H-6a, H-6b), 4.11 – 3.99 (m, 3H, H-3a, H-5c, H-4c), 3.97 – 3.91 (m, 3H, H-6a, H-6b, H-3b), 3.90

– 3.83 (m, 2H, H-5b, H-2b), 3.80 (s, 3H, COOMe), 3.76 (d, *J* = 4.3 Hz, 1H, H-1c), 3.52 – 3.47 (m, 1H, H-5a), 3.00 (dd, *J* = 11.1, 4.2 Hz, 1H, H-2c), 2.86 – 2.67 (m, 2H, Lev), 2.64 – 2.44 (m, 2H, Lev), 2.18 (s, 3H, Lev), 1.56 (s, 3H, Me), 1.08 (d, *J* = 6.5 Hz, 3H, H-6c), 0.98 – 0.95 (m, 18H, *t*-Bu). ^{13}C NMR (126 MHz, $CDCl_3$) δ 206.36 (Lev), 171.77 (Lev), 170.47 (COOMe), 164.38 (Bz), 154.86 (Troc), 133.49, 133.32, 132.22, 130.48, 130.24, 128.90, 128.55, 128.26, 98.88 (C_{quart}), 98.18 (C-1c, *J* = 172 Hz), 95.75 (Troc), 91.44 (C-1b, *J* = 172 Hz), 86.37 (C-1a, *J* = 154 Hz), 75.45 (C-3a), 74.76 (C-4b), 74.74 (Troc), 74.67 (C-3b), 70.99 (C-3c), 70.77 (C-5b), 69.01 (C-5a), 68.69 (C-2a), 66.55 (C-4a), 65.63 (C-6a), 64.73 (C-5c), 64.11 (C-2b), 63.00 (C-6b), 57.83 (C-2c), 52.89 (C-4c), 52.80 (COOMe), 37.92 (Lev), 29.90 (Lev), 28.37 (*t*-Bu), 28.15 (*t*-Bu), 27.87 (Lev), 25.57 (Me), 22.00 (*t*-Bu), 21.29 (*t*-Bu), 16.31 (C-6c). HR-MS: Calculated for $C_{51}H_{66}Cl_3N_7O_{18}SSi$ $[M+NH_4]^+$: 1247.3358, found: 1247.3354. $[\alpha]^{20}_D$ = + 118.8° (c = 1, $CHCl_3$). TLC: Rf = 0.25 (Pentane/EA = 4/1, v/v).

3-*O*-(2-azido-4-*O*-(2-azido-3-*O*-levulinoyl-6-deoxy-4-*N*-2,2,2-trichloroethyl-α-D-galactopyranoside)-3,6-*O*-di-*tert*-butylsilylidene-α-D-galactopyranoside)-2-*O*-benzoyl-4,6-di-*O*-[1-(*R*)-(methoxycarbonyl)-ethyldiene]-α/β-D-galactopyranoside (51)

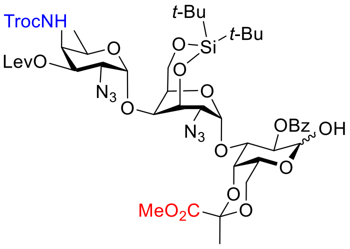

Compound **50** (1.25 g, 1.02 mmol, 1.0 eq) was dissolved in DCM (15 mL) and colled to 0 °C. NIS (343 mg, 1.52 mmol, 1.5 eq) and TFA (94 µL, 1.22 mmol, 1.2 eq) were added and the solution stirred for 2 hours. The reaction was quenched with triethyl amine and aqueous sodium thiosulphate. The solution was diluted with DCM and washed with brine (3×). The organic phase was dried with MgSO4, filtered, and concentrated *in vacuo*. The compound was purified by flash chromatography (Pentane/ EA 2:1 - 1:1) to yield the titled compound **51** (1.16 g, 1.02 mmol, quant.). ^{1}H NMR (500 MHz, $CDCl_3$) δ 8.16 – 8.06 (m, 2H), 7.80 – 7.70 (m, 1H), 7.62 – 7.52 (m, 2H), 5.73 – 5.44 (m, 2H), 4.97 – 4.88 (m, 1H), 4.84 (dd, *J* = 12.0, 5.0 Hz, 1H), 4.80 – 4.70 (m, 2H), 4.57 – 4.51 (m, 1H), 4.51 – 4.43 (m, 1H), 4.43 – 4.26 (m, 2H), 4.21 – 3.91 (m, 8H), 3.88 – 3.75 (m, 5H), 3.64 – 3.47 (m, 1H), 3.06 – 2.95 (m, 1H), 2.87 – 2.43 (m, 4H), 2.18 (s, 3H), 1.66 – 1.59 (m, 3H), 1.07 – 1.02 (m, 3H), 1.01 – 0.93 (m, 18H). ^{13}C NMR (126 MHz, $CDCl_3$) δ 206.45, 171.79, 170.53, 170.38, 166.05, 165.43, 154.87, 133.64, 133.36, 130.59, 130.38, 130.27, 130.09, 128.61, 128.59, 99.12, 99.04, 97.91, 97.87, 96.06, 95.74, 91.71, 91.30, 91.18, 74.90, 74.77, 74.32, 74.26, 73.69, 72.64, 71.02, 71.00, 70.79, 70.65, 69.82, 69.48, 66.98, 66.41, 66.03, 65.69, 65.50, 64.76, 64.68, 64.27, 64.14, 63.25, 63.11, 61.31, 57.80, 52.94, 52.90, 52.84, 37.94, 29.92, 28.43, 28.40, 28.20, 28.19, 27.88, 25.72, 25.64, 22.02, 21.35, 21.33, 16.28. HR-MS:

Calculated for $C_{45}H_{62}Cl_3N_7O_{19}Si$ [M+Na$^+$]: 1160.2828, found: 1160.2821. TLC: Rf = 0.1 (Pentane/EA = 2/1, v/v).

***N*-phenyl-trifluoroacetimidoyl 3-*O*-(2-azido-4-*O*-(2-azido-3-*O*-levulinoyl-6-deoxy-4-*N*-2,2,2-trichloroethyl-α-D-galactopyranoside)-3,6-*O*-di-*tert*-butylsilylidene-α-D-galactopyranoside)-2-*O*-benzoyl-4,6-di-*O*-[1-(*R*)-(methoxycarbonyl)-ethyldiene]-α/β-D-galactopyranoside (52)**

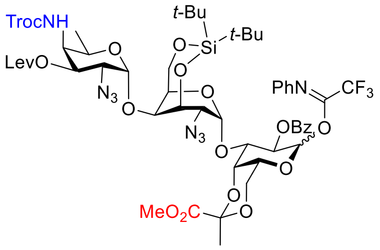

Compound **51** (135 mg, 0.118 mmol, 1.0 eq) was dissolved in acetone (3.0 mL) and reduced to 0 °C. *N*-phenyl trifluoroacetimidoyl chloride (37 mg, 0.178 mmol, 1.5 eq) and cesium carbonate (46 mg, 0.14 mmol, 1.2 eq) were added. The solution was allowed to warm to RT and stirred overnight. After analysis by TLC showed complete consumption of the starting material, the reaction was quenched with triethyl amine, filtered, and concentrated *in vacuo*. The compound was purified by flash chromatography (Pentane/EA 5:1 - 3:1) to yield compound **52** (155 mg, 0.118 mmol, quant.). ^{1}H NMR (500 MHz, Acetone) δ 8.10 (d, *J* = 7.4 Hz, 2H), 7.87 – 7.76 (m, 1H), 7.72 – 7.62 (m, 2H), 7.17 (t, *J* = 7.6 Hz, 2H), 7.09 – 6.98 (m, 1H), 6.48 (d, *J* = 9.9 Hz, 3H, NH), 5.76 (d, *J* = 10.3 Hz, 1H, H-2a), 5.65 (d, *J* = 6.3 Hz, 1H, H-1b), 5.12 (dd, *J* = 11.3, 4.2 Hz, 1H, H-3c), 4.93 – 4.75 (m, 3H, Troc, H-4a), 4.66 – 4.41 (m, 2H, H-3a, H-5b), 4.36 – 3.94 (m, 10H, H-6b, H-1c, H-5c, H-4b, H-6a, H-4c, H-5a), 3.91 (d, *J* = 6.3 Hz, 1H, H-2b), 3.86 – 3.78 (m, 3H, COOMe), 3.74 (dd, *J* = 11.3, 4.1 Hz, 1H, H-2c), 2.83 – 2.68 (m, 2H, Lev), 2.59 – 2.42 (m, 2H, Lev), 2.16 – 2.10 (m, 3H, Lev), 1.54 (s, 3H, Me), 1.09 (d, *J* = 6.4 Hz, 3H, H-6c), 1.05 – 0.94 (m, 18H, *t*-Bu). ^{13}C NMR (126 MHz, Acetone) δ 206.18, 206.01 (Lev), 172.11 (Lev), 170.79 (COOMe), 165.69 (Bz), 156.24 (Troc), 144.24, 134.65, 130.65, 130.24, 129.86, 129.53, 99.50 (C_{quart}), 99.35 (C-1c), 96.99 (Troc), 93.25 (C-1b), 76.08 (C-3b), 75.20 (C-5b), 74.98 (Troc), 71.69 (C-3a), 71.50 (C-4b), 71.36 (C-3c), 69.00 (C-2a), 68.19 (C-4a), 65.91 (C-5c), 65.57 (C-6a), 65.33 (C-5a), 64.77 (C-2b), 64.04 (C-6b), 58.73 (C-2c), 53.93 (C-4c), 52.93 (COOMe), 38.08 (Lev), 29.59 (Lev), 28.87 (*t*-Bu), 28.81 (*t*-Bu), 28.58 (Lev), 25.94 (Me), 22.49 (*t*-Bu), 21.90 (*t*-Bu), 16.46 (C-6c). HR-MS: Calculated for $C_{53}H_{66}Cl_3F_3N_8O_{19}Si$ [M+NH$_4$]$^+$: 1326.3569, found: 1326.3565. TLC: Rf = 0.8 (Pentane/EA = 2/1, v/v).

Propynyl 3-*O*-(2-azido-4-*O*-(2-azido-6-deoxy-4-*N*-2,2,2-trichloroethyl-α-D-galactopyranoside)-3,6-*O*-di-*tert*-butylsilylidene-α-D-galactopyranoside)-2-*O*-benzoyl-4,6-di-*O*-[1-(*R*)-(methoxycarbonyl)-ethyldiene]-β-D-galactopyranoside (53)

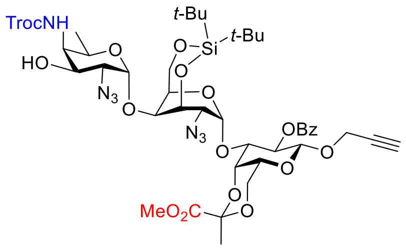

Compound **42** (175 mg, 0.149 mmol, 1.0 eq) was dissolved in pyridine (1.0 mL) and acetic acid (1.0 mL). After cooling to 0 °C, hydrazine acetate (N_2H_4 • AcOH) (41 mg, 0.45 mmol, 3.0 eq) was added. After stirring 3 hours at RT, TLC anbalysis showed complete consumption of the starting material and the reaction was quenched by acetone. The solution was diluted by EtOAc and then washed with water (2x) and brine. The aqueous layer was extracted with DCM (3x), dried with $MgSO_4$, filtered, and concentrated *in vacuo*. The compound was purified by flash chromatography (Pentane/EA 4:1 – 2:1) to yield compound **53** (156 mg, 0.144 mmol, 97%). ^1H NMR (500 MHz, $CDCl_3$) δ 8.18 – 8.09 (m, 2H), 7.76 – 7.67 (m, 1H), 7.60 – 7.51 (m, 2H), 5.67 (dd, *J* = 10.0, 8.1 Hz, 1H, H-2a), 5.50 (d, *J* = 6.3 Hz, 1H, H-1b), 4.91 – 4.81 (m, 3H, NH, Troc, H-1a), 4.78 (d, *J* = 12.0 Hz, 1H, Troc), 4.51 – 4.41 (m, 2H, H-4a, propynyl), 4.39 – 4.26 (m, 3H, propynyl, H-4b, H-6b), 4.18 (dd, *J* = 12.9, 1.6 Hz, 1H, H-6a), 4.09 (dd, *J* = 10.0, 3.7 Hz, 1H, H-3a), 4.04 – 3.91 (m, 5H, H-6b, H-6a, H-3b, H-5b, H-5c), 3.91 – 3.83 (m, 3H, H-4c, H-3c, H-2b), 3.82 (s, 3H, COOMe), 3.72 (d, *J* = 4.3 Hz, 1H, H-1c), 3.53 – 3.43 (m, 1H, H-5a), 2.74 (dd, *J* = 10.1, 4.3 Hz, 1H, H-2c), 2.70 (d, *J* = 3.7 Hz, 1H, 3c-OH), 2.34 (t, *J* = 2.4 Hz, 1H, CH-propynyl), 1.62 (s, 3H, Me), 1.09 (d, *J* = 6.5 Hz, 3H, H-6c), 1.01 – 0.92 (m, 18H, *t*-Bu). ^{13}C NMR (126 MHz, $CDCl_3$) δ 170.51 (COOMe), 164.61 (Bz), 156.33 (Troc), 133.34, 130.48, 130.44, 128.40, 99.08 (C_{quart}), 98.33 (C-1a, *J* = 159 Hz), 98.00 (C-1c, *J* = 171 Hz), 95.48 (Troc), 91.36 (C-1b, *J* = 173 Hz), 78.67 (propynyl), 75.27 (propynyl), 75.05 (Troc), 74.83 (C-3b), 74.11 (C-3a, C-4b), 70.76 (C-5b), 69.87 (C-2a), 69.82 (C-3c), 66.38 (C-4a), 65.81 (C-5a), 65.38 (C-6a), 64.81 (C-5c), 64.19 (C-2b), 63.16 (C-6b), 60.85 (C-2c), 55.97 (C-4c), 55.67 (propynyl), 52.84 (COOMe), 28.36 (t-Bu), 28.32 (t-Bu), 25.64 (Me), 22.01 (*t*-Bu), 21.34 (*t*-Bu), 16.50 (C-6c). HR-MS: Calculated for $C_{43}H_{58}Cl_3N_7O_{17}Si$ $[M+NH_4]^+$: 1095.3062, found: 1095.3051. $[\alpha]^{20}_D$ = + 113.6° (c = 1, $CHCl_3$). TLC: Rf = 0.4 (Pentane/EA = 2/1, v/v).

Hexasaccharide (54)

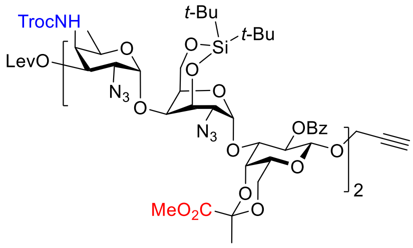

Donor **52** (216 mg, 0.165 mmol, 1.6 eq) and acceptor **53** (110 mg, 0.102 mmol, 1.0 eq) were co-evaporated with toluene (3×) and placed under a nitrogen atmosphere. Dry DCM (3.0 mL) and 4Å molecular sieves were added and the solution stirred for 30 minutes before being cooled to 0 °C. TBSOTf (3.0 µL, 0.013 mmol, 0.1 eq) was added to the reaction and stirred 4 hours. After analysis by TLC showed complete consumption of the starting material, the reaction was

quenched with triethylamine and concentrated *in vacuo*. The compound was purified by flash chromatography (Pentane/EA 3:1 - 1:1) to yield compound **54** (177 mg, 0.08 mmol, 79%).^{1}H NMR (500 MHz, $CDCl_3$) δ 8.23 – 7.94 (m, 4H), 7.83 – 7.69 (m, 1H), 7.65 – 7.46 (m, 3H), 7.46 – 7.31 (m, 2H), 5.70 – 5.53 (m, 2H), 5.53 – 5.37 (m, 2H), 5.10 (d, *J* = 12.2 Hz, 1H), 4.96 – 4.71 (m, 5H), 4.67 (d, *J* = 9.6 Hz, 1H), 4.62 – 4.50 (m, 2H), 4.49 – 3.64 (m, 34H), 3.59 (s, 1H), 3.50 – 3.31 (m, 2H), 2.99 (d, *J* = 10.9 Hz, 1H), 2.88 – 2.42 (m, 5H), 2.38 – 2.25 (m, 1H), 2.18 (s, 3H), 1.64 – 1.51 (m, 6H), 1.17 – 1.03 (m, 6H), 1.03 – 0.80 (m, 36H). ^{13}C NMR (126 MHz, $CDCl_3$) δ 206.36, 171.79, 170.64, 170.52, 164.56, 164.39, 155.03, 154.87, 133.32, 133.23, 130.39, 130.35, 130.24, 128.44, 128.40, 102.00, 99.06, 98.89, 98.32, 98.20, 97.65, 95.87, 95.80, 91.55, 91.43, 78.70, 76.05, 75.21, 74.93, 74.86, 74.81, 74.73, 74.21, 73.64, 71.02, 70.79, 70.63, 69.86, 66.42, 66.29, 65.87, 65.83, 65.79, 65.39, 64.84, 64.77, 64.10, 63.16, 63.10, 59.89, 57.88, 55.62, 54.77, 52.96, 52.81, 52.79, 37.96, 29.93, 28.62, 28.47, 28.40, 28.19, 27.90, 25.63, 25.60, 22.07, 22.04, 21.37, 21.33, 16.35, 16.30. HR-MS: Calculated for $C_{88}H_{118}Cl_6N_{14}O_{35}Si_2$ $[M+NH_4]^+$: 2214.5892, found: 2214.5902. $[\alpha]^{20}_D$ = + 148.0º (c = 0.1, $CHCl_3$). TLC: Rf = 0.25 (Pentane/EA = 2/1, v/v).

Hexasaccharide (57)

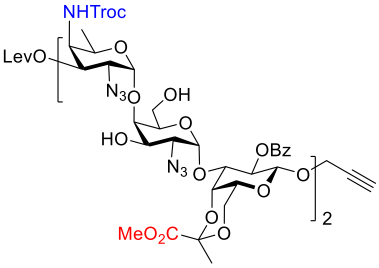

Compound **54** (42 mg, 0.019 mmol, 1.0 eq) was dissolved in THF (1.0 mL) and pyridine (1.0 mL), then cooled to 0 °C. Hydrogen fluoride (HF)/pyridine (70%) (0.1 mL) was added dropwise. The solution was stirred for 4 hours. After TLC showed complete consumption of the starting material, the reaction was quenched with saturated aqueous sodium bicarbonate slowly and diluted with EtOAc. The solution was washed with water (2x) and brine. The aqueous layer was extracted with EtOAc (3x), dried with $MgSO_4$, filtered, and concentrated *in vacuo*. The compound was purified by flash chromatography (DCM/Acetone 5:1 – 4:1) to yield compound **57** (31.6 mg, 0.016 mmol, 92%). ^{1}H NMR (500 MHz, $CDCl_3$) δ 8.14 – 8.01 (m, 4H), 7.65 – 7.55 (m, 2H), 7.52 – 7.41 (m, 4H), 5.61 – 5.50 (m, 2H), 5.44 – 5.34 (m, 1H), 5.33 – 5.26 (m, 1H), 5.25 – 5.12 (m, 3H), 4.99 (d, *J* = 12.1 Hz, 1H), 4.91 – 4.84 (m, 2H), 4.83 – 4.74 (m, 3H), 4.71 (d, *J* = 12.1 Hz, 1H), 4.55 – 4.47 (m, 2H), 4.47 – 4.33 (m, 5H), 4.32 – 4.11 (m, 5H), 4.09 – 3.77 (m, 14H), 3.73 – 3.39 (m, 10H), 3.38 – 3.28 (m, 3H), 3.23 – 3.06 (m, 3H), 2.90 – 2.44 (m, 4H), 2.36 (t, *J* = 2.4 Hz, 1H), 2.18 (d, *J* = 3.3 Hz, 3H), 1.66 (s, 3H), 1.62 (s, 3H), 1.22 – 1.12 (m, 6H). ^{13}C NMR (126 MHz, $CDCl_3$) δ 206.53, 172.19, 170.58, 165.16, 155.18, 133.67, 133.51, 129.90, 129.85, 129.69, 128.81, 128.66, 101.89, 99.15, 98.98,

98.83, 98.26, 95.70, 95.31, 94.70, 79.83, 79.33, 78.72, 75.28, 74.85, 74.75, 74.50, 73.98, 71.05, 70.77, 70.71, 70.37, 70.01, 67.17, 67.05, 66.52, 66.15, 65.91, 65.39, 64.85, 60.53, 60.40, 60.29, 60.24, 60.13, 58.50, 55.65, 53.91, 52.98, 52.89, 37.85, 29.92, 29.82, 29.39, 27.98, 25.70, 25.61, 16.45, 16.21. HR-MS: Calculated for $C_{72}H_{86}Cl_6N_{14}O_{35}$ $[M+NH_4]^+$: 1934.3849, found: 1934.3860. $[\alpha]^{20}_D$ = + 183.0° (c = 0.1, $CHCl_3$). TLC: Rf = 0.2 (DCM/Acetone = 4/1, v/v).

Hexasaccharide (58)

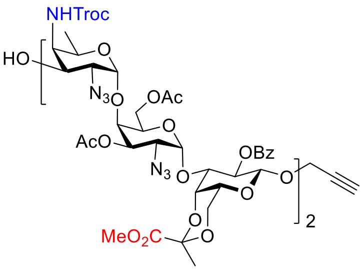

Compound **57** (20 mg, 10.4 µmol, 1.0 eq) was dissolved in the pyridine (1.0 mL) and put in an ice bath. Ac_2O (0.5 mL) was added. The reaction was stirred overnight. After analysis by TLC showed complete consumption of the starting material, the reaction was concentrated *in vacuo*. The crude was dissolved in pyridine (1.0 mL) and acetic acid (1.0 mL). After cooling to 0 °C, hydrazine acetate (N_2H_4 • AcOH) (5.0 mg, 0.54 µmol, 5.4 eq) was added. After stirring 4 hours at RT, TLC analysis showed complete consumption of the starting material, the reaction was quenched by acetone. The reaction was concentrated *in vacuo*. The compound was purified by flash chromatography (DCM/Acetone 8:1 – 6:1) to yield compound **58** (18.3 mg, 9.19 µmol, 88%). ^{1}H NMR (500 MHz, $CDCl_3$) δ 8.10 – 7.99 (m, 4H), 7.59 – 7.50 (m, 2H), 7.45 – 7.37 (m, 4H), 5.65 – 5.50 (m, 2H), 5.42 – 5.29 (m, 2H), 5.29 – 5.15 (m, 2H), 5.09 – 4.78 (m, 6H), 4.72 – 4.57 (m, 3H), 4.50 – 4.33 (m, 6H), 4.33 – 3.69 (m, 25H), 3.57 – 3.43 (m, 4H), 3.28 – 3.13 (m, 2H), 2.96 (s, 1H), 2.37 (t, J = 2.4 Hz, 1H), 2.16 – 2.04 (m, 6H), 1.96 (s, 3H), 1.86 (s, 3H), 1.70 – 1.56 (m, 6H), 1.19 – 1.08 (m, 6H). ^{13}C NMR (126 MHz, $CDCl_3$) δ 170.60, 169.92, 169.81, 169.76, 169.69, 164.97, 164.85, 156.38, 155.36, 133.75, 133.19, 129.90, 129.84, 129.80, 129.69, 128.51, 128.44, 117.64, 101.85, 99.12, 98.99, 98.76, 98.70, 98.23, 95.75, 95.37, 93.95, 78.66, 75.28, 75.15, 74.92, 74.83, 74.77, 74.11, 73.71, 73.62, 70.01, 69.67, 69.19, 69.04, 68.39, 68.28, 66.35, 66.17, 66.05, 65.88, 65.78, 65.44, 65.32, 64.87, 61.18, 61.00, 60.71, 59.53, 56.91, 56.88, 56.13, 55.66, 54.98, 52.80, 25.65, 25.58, 21.20, 21.15, 20.92, 20.76, 16.82, 16.68. HR-MS: Calculated for $C_{75}H_{88}Cl_6N_{14}O_{37}$ $[M+NH_4]^+$: 2004.3904, found: 2004.3910. $[\alpha]^{20}_D$ = + 149.0° (c = 0.1, $CHCl_3$). TLC: Rf = 0.1 (DCM/Acetone = 8/1, v/v).

Hexasaccharide (2b)

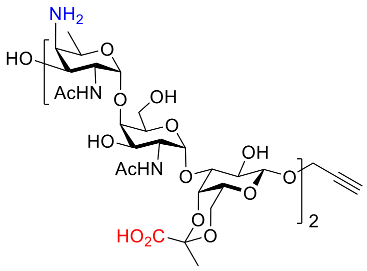

Compound **58** (18.3 mg, 9.2 μmol, 1.0 eq) was dissolved in DCM (1.0 mL) and 3,4-dihydro-2H-pyran (0.1 mL). Then pyridinium *p*-toluenesulfonate (2.4 mg, 9.2 μmol, 1.0 eq) was added at RT. The mixture was stirred at RT overnight. After analysis by TLC showed complete consumption of the starting material, the reaction was quenched with triethylamine and purified by size exclusion chromatography (LH-20) (DCM/MeOH 1:1). The crude was dissolved in THF (1.0 mL) and water (11 μL). Pyridine (50 μL, 0.62 mmol, 64 eq) and Ph_3P (40 mg, 0.15 mmol, 16 eq) were added and the reaction was allowed to stir overnight at 70 °C. After TLC showed complete consumption of the starting material, THF (1.0 ml) and water (1.0 mL) were added to the reaction. Then sodium bicarbonate (155 mg, 1.85 mmol, 200 eq) and acetic anhydride (87 μL, 0.92 mmol, 100 eq) were added and stirred for overnight. After TLC showed complete consumption of the starting material, the reaction mixture was diluted with EtOAc and then washed with saturated aqueous sodium bicarbonate and brine. The aqueous layer was extracted with EtOAc (3x), dried with $MgSO_4$, filtered, and concentrated *in vacuo*. The residue was purified by flash size exclusion (LH-20) (DCM/MeOH 1:1). The crude was dissolved in dioxane (2.0 mL), THF (2.0 mL), water (2.0 mL). LiOH (30 mg, 1.25 mmol, 0.2M) was added and stirred at RT. After analysis by TLC and LC-MS showed complete consumption of the starting material and intermediates, the reaction was quenched with 1M HCl to neutral. Acetic acid (10 mL) was added and heated to 50 °C. After analysis by LC-MS showed complete consumption of the starting material, the solvent was removed through co-evaporation with toluene *in vacuo*. The compound was purified by gel filtration (HW-40, 0.15M NH_4HCO_3 in H_2O) with a Shimadzu RID-10A refractive index detector and lyophilized to yield compound **2b** (6.8 mg, 5.23 μmol, 57% over five steps). ^{1}H NMR (500 MHz, D_2O) δ 5.39 – 5.28 (m, 2H), 5.01 – 4.92 (m, 2H), 4.81 – 4.71 (m, 2H), 4.71 – 4.62 (m, 2H), 4.55 – 4.33 (m, 8H), 4.26 (dd, *J* = 11.5, 4.1 Hz, 1H), 4.22 – 4.10 (m, 4H), 4.10 – 3.90 (m, 8H), 3.83 – 3.55 (m, 11H), 2.90 (t, *J* = 2.4 Hz, 1H), 2.14 – 2.00 (m, 12H), 1.51 – 1.39 (m, 6H), 1.28 – 1.17 (m, 6H). ^{13}C NMR (126 MHz, D_2O) δ 175.26, 174.98, 174.84, 174.71, 174.69, 104.49, 101.27, 101.10, 100.36, 98.26, 98.18, 94.09, 93.90, 78.74, 77.59, 77.46, 76.39, 75.20, 75.06, 75.00, 71.72, 68.69, 68.59, 67.33, 67.30, 67.20, 66.26, 66.08, 65.34, 64.92, 63.94, 63.26, 62.96, 60.13, 60.10, 56.34, 55.38, 54.91, 49.76, 49.52, 48.26, 25.10, 25.06, 22.30, 21.96, 21.93, 15.52. HR-MS: Calculated for $C_{53}H_{82}N_6O_{31}$ $[M+H]^+$: 1299.5097, found: 1299.5093.

Hexasaccharide (59)

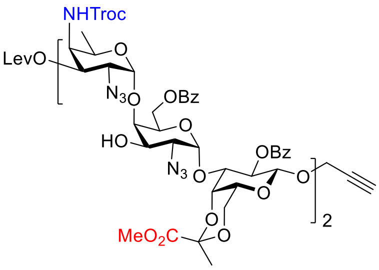

Compound **57** (165 mg, 0.086 mmol, 1.0 eq) was dissolved in DCM (2.0 mL). Et_3N (153 µL, 1.1 mmol, 12.0 eq) and PhCOOBt (219 mg, 0.92 mmol, 11 eq) were added and the mixture was stirred at RT overnight. The reaction was quenched by MeOH and concentrated *in vacuo*. The compound was purified by flash chromatography (DCM/Acetone 20:1 - 5:1) to yield compound **59** (127 mg, 0.06 mmol, 69%). ^{1}H NMR (500 MHz, $CDCl_3$) δ 8.01 – 7.94 (m, 4H), 7.93 – 7.83 (m, 4H), 7.67 – 7.55 (m, 2H), 7.50 – 7.42 (m, 4H), 7.24 – 7.13 (m, 6H), 5.61 – 5.50 (m, 2H), 5.40 – 5.27 (m, 2H), 5.23 – 5.11 (m, 3H), 5.00 (d, J = 12.1 Hz, 1H), 4.80 – 4.64 (m, 5H), 4.61 – 4.51 (m, 2H), 4.51 – 4.27 (m, 9H), 4.26 – 4.08 (m, 5H), 4.01 – 3.77 (m, 15H), 3.77 – 3.67 (m, 2H), 3.59 (dd, J = 11.2, 4.0 Hz, 1H), 3.38 – 3.24 (m, 5H), 3.18 (s, 1H), 2.97 (s, 1H), 2.84 – 2.46 (m, 4H), 2.30 (t, J = 2.4 Hz, 1H), 2.18 (s, 3H), 1.67 – 1.59 (m, 6H), 1.20 – 1.11 (m, 6H). ^{13}C NMR (126 MHz, $CDCl_3$) δ 206.42, 172.15, 170.57, 165.46, 165.42, 164.97, 164.92, 155.22, 155.10, 133.52, 133.49, 133.30, 133.28, 129.76, 129.63, 129.57, 129.53, 129.50, 129.38, 128.68, 128.65, 128.51, 128.47, 101.79, 99.45, 99.17, 98.99, 98.80, 98.34, 95.69, 95.55, 93.85, 93.77, 78.85, 78.69, 75.89, 75.21, 74.82, 74.69, 73.40, 73.37, 70.92, 69.78, 69.58, 68.86, 68.77, 67.12, 67.03, 66.17, 66.06, 65.95, 65.77, 65.42, 64.83, 62.01, 61.82, 60.16, 59.98, 58.26, 55.64, 52.95, 52.90, 52.87, 37.87, 29.89, 27.99, 25.65, 25.58, 16.38, 16.34. HR-MS: Calculated for $C_{86}H_{94}Cl_6N_{14}O_{37}$ $[M+NH_4]^+$: 2142.4374, found: 2142.4379. $[\alpha]^{20}_D$ = + 98.0º (c = 0.1, $CHCl_3$). TLC: Rf = 0.2 (DCM/Acetone = 8/1, v/v).

Octasaccharide (63)

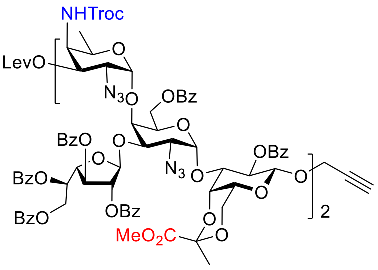

Donor **10** (155 mg, 0.2 mmol, 6.0 eq) and acceptor **59** (71.5 mg, 33.6 µmol, 1.0 eq) were co-evaporated with toluene (3×) and placed under a nitrogen atmosphere. Dry DCM (1.0 mL) and 4Å molecular sieves were added and the solution stirred for 30 minutes before being reduced to 0 °C. TBSOTf (1.0 µL, 4.35 µmol, 0.2 eq) was added to the reaction and stirred 8 hours. After analysis by TLC showed complete consumption of the starting material, the reaction was quenched with triethylamine and concentrated *in vacuo*. The compound was purified by flash chromatography (DCM/Acetone 14:1 - 10:1) to yield compound **63** (80 mg, 24.3 µmol, 72%). ^{1}H NMR (500 MHz, $CDCl_3$) δ 8.17 – 7.77 (m, 24H), 7.65 – 7.27 (m, 30H), 7.25 – 7.11 (m, 6H), 5.94 – 5.82 (m, 2H), 5.69 – 5.51 (m, 8H), 5.48 – 5.41 (m, 1H), 5.39 – 5.31 (m, 1H), 5.13 (dd, J = 11.2, 3.8

Hz, 1H), 5.04 (d, J = 12.1 Hz, 1H), 4.90 (d, J = 9.5 Hz, 1H), 4.85 – 4.65 (m, 8H), 4.65 – 4.58 (m, 2H), 4.58 – 4.30 (m, 12H), 4.29 – 4.19 (m, 3H), 4.19 – 4.08 (m, 3H), 4.04 – 3.71 (m, 17H), 3.70 – 3.57 (m, 2H), 3.42 – 3.17 (m, 2H), 3.14 – 3.05 (m, 1H), 2.87 – 2.43 (m, 4H), 2.31 (t, J = 2.4 Hz, 1H), 2.18 (s, 3H), 1.65 – 1.56 (m, 6H), 1.24 – 1.12 (m, 6H). ^{13}C NMR (126 MHz, $CDCl_3$) δ 206.41, 172.06, 170.62, 170.49, 166.08, 165.96, 165.79, 165.76, 165.73, 165.67, 165.62, 165.45, 165.40, 165.04, 164.91, 155.13, 155.08, 133.90, 133.76, 133.62, 133.56, 133.48, 133.41, 133.34, 133.26, 130.11, 130.04, 129.99, 129.96, 129.91, 129.84, 129.82, 129.72, 129.69, 129.62, 129.31, 129.12, 129.01, 128.90, 128.85, 128.77, 128.73, 128.70, 128.66, 128.63, 128.58, 128.56, 128.49, 128.47, 107.55, 101.67, 99.43, 99.02, 98.90, 98.81, 98.40, 95.85, 95.68, 93.09, 81.33, 81.19, 80.62, 78.74, 78.59, 77.60, 77.30, 75.53, 75.19, 74.98, 74.85, 74.73, 74.56, 73.11, 72.89, 71.03, 70.32, 69.85, 69.62, 66.27, 65.95, 65.86, 65.76, 65.45, 65.25, 64.87, 62.85, 62.76, 60.40, 59.06, 58.83, 58.70, 55.67, 55.10, 53.14, 52.90, 52.83, 37.91, 29.93, 29.81, 28.01, 25.64, 25.62, 16.72, 16.58. HR-MS: Calculated for $C_{154}H_{146}Cl_6N_{14}O_{55}$ $[M+2(NH_4)]^{2+}/2$: 1658.3933, found: 1658.3927. $[\alpha]^{20}_D$ = + 15.0° (c = 0.1, $CHCl_3$). TLC: Rf = 0.3 (DCM/Acetone = 10/1, v/v).

Octasaccharide (2a)

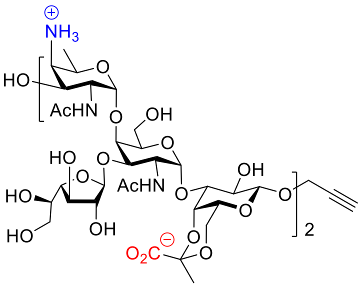

Compound **63** (40.6 mg, 12.4 µmol, 1.0 eq) was dissolved in pyridine (1.0 mL) and acetic acid (1.0 mL). After cooling to 0 °C, hydrazine acetate (N_2H_4 • AcOH) (4.0 mg, 37.2 µmol, 3.0 eq) was added. After stirring 4 hours at RT, TLC analysis showed complete consumption of the starting material, the reaction was quenched with acetone. The reaction was concentrated *in vacuo*. The residue was purified by size exclusion chromatography (LH-20) (DCM/MeOH 1:1). The crude was dissolved in DCM (1.0 mL) and 3,4-dihydro-2H-pyran (0.1 mL). Then pyridinium *p*-toluenesulfonate (3.0 mg, 11.9 µmol, 1.0 eq) was added and the mixture was stirred at RT overnight. After analysis by TLC showed complete consumption of the starting material, the reaction was quenched with triethylamine and purified by size exclusion chromatography (LH-20) (DCM/MeOH 1:1). The crude was dissolved in THF (1.0 mL) and water (15 µL). Pyridine (64 µL, 0.80 mmol, 64 eq) and Ph_3P (52 mg, 0.20 mmol, 16 eq) were added and the reaction was allowed to stir overnight at 70 °C. After TLC showed complete consumption of the starting material, THF (1.0 ml) and water (1.0 mL) were added to the reaction. Then sodium bicarbonate (210 mg, 2.5 mmol, 200 eq) and acetic anhydride (120 µL, 1.27 mmol, 100 eq) were added and stirred for overnight. After TLC showed complete consumption of the starting

material, the reaction mixture was diluted with EtOAc and then washed with saturated aqueous sodium bicarbonate and brine. The aqueous layer was extracted with EtOAc (3x), dried with $MgSO_4$, filtered, and concentrated *in vacuo*. The residue was purified by size exclusion chromatography (LH-20) (DCM/MeOH 1:1). The crude was dissolved in dioxane (2.0 mL), THF (2.0 mL), water (2.0 mL). LiOH (30 mg, 1.25 mmol, 0.2M) was added and stirred at RT. After analysis by TLC and LC-MS showed complete consumption of the starting material and intermediates, the reaction was quenched with 1M HCl to neutral pH. Acetic acid (10 mL) was added and the mixture heated to 50 °C. After analysis by LC-MS showed complete consumption of the starting material, the solvent was removed through co-evaporation with toluene *in vacuo*. The compound was purified by gel filtration (HW-40, 0.15M NH_4HCO_3 in H_2O) with a Shimadzu RID-10A refractive index detector and lyophilized to yield compound **2a** (13.1 mg, 8.07 µmol, 65% over six steps). ^{1}H NMR (500 MHz, D_2O) δ 5.36 – 5.28 (m, 2H), 5.10 – 5.01 (m, 4H), 4.76 – 4.73 (m, 2H), 4.71 – 4.66 (m, 2H), 4.61 – 4.55 (m, 2H), 4.51 – 4.35 (m, 6H), 4.27 – 4.17 (m, 5H), 4.15 – 4.08 (m, 2H), 4.08 – 3.92 (m, 12H), 3.85 – 3.56 (m, 18H), 2.90 (t, *J* = 2.4 Hz, 1H), 2.10 – 1.99 (m, 12H), 1.50 – 1.41 (m, 6H), 1.36 – 1.28 (m, 6H). ^{13}C NMR (126 MHz, D_2O) δ 175.27, 175.25, 174.98, 174.74, 174.66, 108.94, 104.44, 101.27, 101.09, 100.38, 97.93, 97.76, 94.21, 93.97, 81.83, 81.75, 80.65, 78.75, 77.49, 77.40, 76.40, 75.69, 75.62, 75.32, 74.98, 74.95, 71.98, 71.89, 70.50, 70.46, 68.65, 68.54, 67.41, 67.23, 66.25, 66.11, 65.34, 64.91, 63.69, 63.20, 62.97, 62.67, 60.26, 60.22, 56.36, 55.41, 54.89, 50.11, 48.77, 48.63, 25.10, 25.06, 22.33, 21.98, 21.92, 16.14. HR-MS: Calculated for $C_{65}H_{102}N_6O_{41}$ $[M+2H]^{2+}/2$: 812.3113, found: 812.3107.

Hexasaccharide (55)

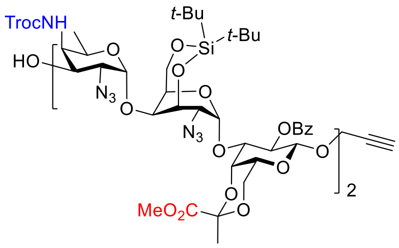

Compound **54** (262 mg, 0.119 mmol, 1.0 eq) was dissolved in pyridine (1.0 mL) and acetic acid (1.0 mL). After cooling to 0 °C, hydrazine acetate (N_2H_4 • AcOH) (33 mg, 0.36 mmol, 3.0 eq) was added. After stirring 6 hours at RT, TLC analysis showed complete consumption of the starting material, the reaction was quenched with acetone and concentrated *in vacuo*. The residue was purified by size exclusion chromatography (LH-20) (DCM/MeOH 1:1) to yield compound **55** (240 mg, 0.114 mmol, 96%). ^{1}H NMR (500 MHz, $CDCl_3$) δ 8.18 – 7.95 (m, 4H), 7.73 (t, *J* = 7.5 Hz, 1H), 7.63 – 7.48 (m, 3H), 7.44 – 7.35 (m, 2H), 5.68 – 5.56 (m, 2H), 5.52 – 5.39 (m, 2H), 5.10 (d, *J* = 12.1 Hz, 1H), 4.93 – 4.81 (m, 3H), 4.80 – 4.66 (m, 2H), 4.62 – 4.51 (m, 2H), 4.49 – 4.38 (m, 3H), 4.37 – 4.25 (m, 4H), 4.24 – 4.13 (m, 2H), 4.12 – 3.67 (m, 25H), 3.59 (d, *J* = 4.3

Hz, 1H), 3.50 – 3.33 (m, 2H), 2.91 – 2.72 (m, 2H), 2.65 (dd, *J* = 10.9, 4.2 Hz, 1H), 2.32 (t, *J* = 2.4 Hz, 1H), 2.02 (s, 1H), 1.63 – 1.56 (m, 6H), 1.11 – 1.05 (m, 6H), 0.99 – 0.92 (m, 36H). ^{13}C NMR (126 MHz, $CDCl_3$) δ 170.61, 170.47, 164.52, 164.36, 156.23, 154.99, 133.25, 133.18, 130.31, 130.29, 130.17, 128.38, 128.35, 101.94, 98.99, 98.83, 98.28, 98.10, 97.62, 95.82, 95.50, 91.39, 91.35, 78.65, 75.95, 75.22, 75.02, 74.86, 74.81, 74.27, 74.17, 74.09, 73.60, 71.12, 70.75, 70.58, 70.33, 69.79, 69.66, 66.38, 66.21, 65.79, 65.76, 65.72, 65.32, 64.82, 64.07, 64.04, 63.15, 63.09, 60.77, 59.81, 55.98, 55.59, 54.72, 52.76, 52.74, 29.76, 28.55, 28.52, 28.43, 28.37, 28.35, 28.32, 28.29, 25.58, 25.55, 22.01, 21.97, 21.31, 16.47, 16.25. HR-MS: Calculated for $C_{83}H_{112}Cl_6N_{14}O_{33}Si_2$ $[M+NH_4]^+$: 2116.5524, found: 2116.5524. $[\alpha]^{20}_D$ = + 150.0º (c = 0.1, $CHCl_3$). TLC: Rf = 0.4 (Pentane/EA = 2/1, v/v).

Nonasaccharide (56)

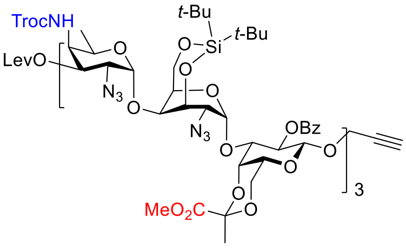

Donor **52** (120 mg, 0.091 mmol, 1.67 eq) and acceptor **55** (115 mg, 0.055 mmol, 1.0 eq) were co-evaporated with toluene (3×) and placed under a nitrogen atmosphere. Dry DCM (3.0 mL) and 4Å molecular sieves were added and the solution stirred for 30 minutes before being cooled to 0 °C. TBSOTf (3.0 µL, 0.013 mmol, 0.1 eq) was added to the reaction and stirred 5 hours. After analysis by TLC showed complete consumption of the starting material, the reaction was quenched with triethylamine and concentrated *in vacuo*. The compound was purified by flash chromatography (Pentane/EA 3:1 - 1:1) to yield compound **56** (151.7 mg, 0.047 mmol, 86%). ^{1}H NMR (400 MHz, $CDCl_3$) δ 8.20 – 7.94 (m, 6H), 7.76 (t, *J* = 7.6 Hz, 1H), 7.64 – 7.47 (m, 4H), 7.46 – 7.32 (m, 4H), 5.70 – 5.39 (m, 6H), 5.16 – 5.04 (m, 2H), 4.95 – 4.66 (m, 7H), 4.63 – 4.50 (m, 4H), 4.49 – 3.63 (m, 52H), 3.56 (d, *J* = 4.2 Hz, 2H), 3.46 (s, 1H), 3.42 – 3.30 (m, 2H), 2.99 (dd, *J* = 11.2, 4.3 Hz, 1H), 2.90 – 2.43 (m, 4H), 2.33 (t, *J* = 2.4 Hz, 1H), 2.18 (s, 3H), 1.66 – 1.52 (m, 9H), 1.14 – 1.03 (m, 9H), 1.02 – 0.88 (m, 54H). ^{13}C NMR (101 MHz, $CDCl_3$) δ 206.39, 171.75, 170.62, 170.59, 170.49, 164.54, 164.37, 155.00, 154.83, 133.24, 130.29, 130.14, 130.08, 128.39, 101.94, 99.02, 98.86, 98.82, 98.27, 98.15, 97.63, 95.85, 95.80, 91.51, 91.31, 78.64, 76.00, 75.23, 74.87, 74.81, 74.75, 74.66, 74.11, 73.85, 73.63, 70.98, 70.73, 70.57, 70.34, 69.77, 66.34, 66.23, 65.76, 65.34, 64.81, 64.72, 64.04, 63.93, 63.10, 59.77, 57.82, 55.61, 54.71, 52.90, 52.79, 52.75, 37.91, 30.40, 29.78, 28.55, 28.36, 28.14, 25.59, 25.56, 25.54, 22.79, 22.01, 21.33, 21.29, 16.31, 14.24. HR-MS: Calculated for $C_{128}H_{172}Cl_9N_{21}O_{51}Si_3$ $[M+2(NH_4)]^{2+}/2$: 1626.9346, found: 1626.9335. $[\alpha]^{20}_D$ = + 143.0º (c = 0.1, $CHCl_3$). TLC: Rf = 0.25 (Pentane/EA = 2/1, v/v).

Nonasaccharide (61)

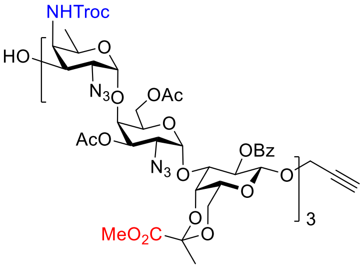

Compound **56** (76.1 mg, 0.024 mmol, 1.0 eq) was dissolved in THF (1.0 mL) and pyridine (1.0 mL), then cooled to 0 °C. Hydrogen fluoride (HF)/pyridine (70%) (0.1 mL) was added dropwise. The solution was stirred for 4 hours. After TLC showed complete consumption of the starting material, the reaction was quenched with saturated aqueous sodium bicarbonate slowly and diluted with EtOAc. The solution was washed with water (2x) and brine. The aqueous layer was extracted with EtOAc (3x), dried with $MgSO_4$, filtered, and concentrated *in vacuo*. The residue was purified by size exclusion chromatography (LH-20) (DCM/MeOH 1:1). The crude was dissolved in the pyridine (1.0 mL) and put in an ice bath. Ac_2O (0.5 mL) was added and the reaction was stirred overnight. After analysis by TLC showed complete consumption of the starting material, the mixture was concentrated *in vacuo*. The residue was purified by flash size exclusion (LH-20) (DCM/MeOH 1:1). The crude was dissolved in pyridine (1.0 mL) and acetic acid (1.0 mL). After cooling to 0 °C, hydrazine acetate (N_2H_4•AcOH) (12 mg, 0.13 mmol, 5.0 eq) was added. After stirring 4 hours at RT, TLC analysis showed complete consumption of the starting material, the reaction was quenched with acetone and concentrated *in vacuo*. The residue was purified by size exclusion chromatography (LH-20) (DCM/MeOH 1:1) to yield the compound **61** (59.3 mg, 0.02 mmol, 85% for 3steps). ^{1}H NMR (400 MHz, $CDCl_3$) δ 8.12 – 7.98 (m, 6H), 7.60 – 7.49 (m, 3H), 7.46 – 7.35 (m, 6H), 5.65 – 5.51 (m, 3H), 5.40 – 5.31 (m, 3H), 5.26 – 5.11 (m, 3H), 5.10 – 4.93 (m, 5H), 4.93 – 4.79 (m, 4H), 4.70 – 4.57 (m, 4H), 4.50 – 4.33 (m, 8H), 4.33 – 4.14 (m, 9H), 4.14 – 4.04 (m, 6H), 4.04 – 3.93 (m, 5H), 3.93 – 3.63 (m, 18H), 3.58 – 3.40 (m, 6H), 3.28 – 3.12 (m, 3H), 2.88 (s, 1H), 2.37 (t, J = 2.4 Hz, 1H), 2.15 – 2.07 (m, 9H), 1.96 (s, 3H), 1.90 (s, 3H), 1.86 (s, 3H), 1.69 – 1.58 (m, 9H), 1.20 – 1.09 (m, 9H). ^{13}C NMR (101 MHz, $CDCl_3$) δ 170.71, 170.61, 169.89, 169.82, 169.75, 169.71, 169.68, 164.96, 164.81, 156.39, 155.35, 133.19, 129.90, 129.69, 129.62, 128.44, 101.87, 99.13, 99.00, 98.76, 98.66, 98.22, 95.73, 95.36, 93.94, 93.72, 78.66, 77.36, 75.28, 75.22, 74.93, 74.83, 74.78, 74.07, 73.70, 73.38, 69.97, 69.65, 69.17, 69.03, 68.93, 68.47, 68.36, 68.27, 66.34, 66.16, 66.03, 65.86, 65.77, 65.45, 65.30, 64.90, 61.15, 61.01, 60.69, 59.48, 56.91, 56.11, 55.67, 54.96, 52.81, 52.76, 25.66, 25.58, 21.21, 21.16, 20.93, 20.88, 20.77, 16.83, 16.69. HR-MS: Calculated for $C_{111}H_{130}Cl_9N_{21}O_{55}$ $[M+2(NH_4)]^{2+}/2$: 1493.7947, found: 1493.7956. $[\alpha]^{20}_D$ = + 146.0° (c = 0.1, $CHCl_3$). TLC: Rf = 0.3 (DCM/Acetone = 6/1, v/v).

Nonasaccharide (3b)

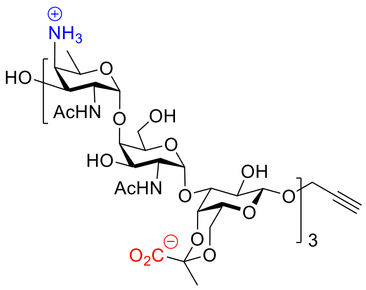

Compound **61** (33.0 mg, 11 µmol, 1.0 eq) was dissolved in DCM (1.0 mL) and 3,4-dihydro-2H-pyran (0.1 mL). Then pyridinium *p*-toluenesulfonate (2.8 mg, 11 µmol, 1.0 eq) was added and the mixture was stirred at RT overnight. After analysis by TLC showed complete consumption of the starting material, the reaction was quenched with triethylamine and purified by size exclusion chromatography (LH-20) (DCM/MeOH 1:1). The crude was dissolved in THF (1.0 mL) and water (20 µL). Pyridine (85 µL, 1.06 mmol, 96 eq) and Ph_3P (70 mg, 0.27 mmol, 24 eq) were added and the reaction was allowed to stir overnight at 70 °C. After TLC showed complete consumption of the starting material, THF (1.0 ml) and water (1.0 mL) were added to the reaction. Then sodium bicarbonate (185 mg, 2.2 mmol, 200 eq) and acetic anhydride (104 µL, 1.1 mmol, 100 eq) were added and stirred overnight. After TLC showed complete consumption of the starting material, the reaction mixture was diluted with EtOAc and then washed with saturated aqueous sodium bicarbonate and brine. The aqueous layer was extracted with EtOAc (3x), dried with $MgSO_4$, filtered, and concentrated *in vacuo*. The residue was purified by size exclusion chromatography (LH-20) (DCM/MeOH 1:1). The crude was dissolved in dioxane (2.0 mL), THF (2.0 mL), water (2.0 mL). LiOH (30 mg, 1.25 mmol, 0.2M) was added and stirred at RT. After analysis by TLC and LC-MS showed complete consumption of the starting material and intermediates, the reaction was quenched with 1M HCl to neutral pH. Acetic acid (10 mL) was added and heated to 50 °C. After analysis by LC-MS showed complete consumption of the starting material, the solvent was removed through co-evaporation with toluene *in vacuo*. The compound was purified by gel filtration (HW-40, 0.15M NH_4HCO_3 in H_2O) with a Shimadzu RID-10A refractive index detector and lyophilized to yield compound **3b** (10.4 mg, 5.41 µmol, 49% over five steps). ^{1}H NMR (500 MHz, D_2O) δ 5.37 – 5.27 (m, 3H), 5.01 – 4.91 (m, 3H), 4.70 – 4.61 (m, 3H), 4.77 – 4.75 (m, 2H), 4.53 – 4.33 (m, 11H), 4.29 – 4.22 (m, 2H), 4.21 – 4.11 (m, 6H), 4.09 – 3.90 (m, 13H), 3.83 – 3.66 (m, 12H), 3.66 – 3.56 (m, 4H), 2.90 (t, J = 2.4 Hz, 1H), 2.12 – 2.01 (m, 18H), 1.51 – 1.41 (m, 9H), 1.28 – 1.16 (m, 9H). ^{13}C NMR (126 MHz, D_2O) δ 175.25, 174.97, 174.83, 174.81, 174.72, 174.70, 174.69, 104.50, 101.27, 101.10, 100.36, 98.29, 98.26, 98.18, 94.09, 93.88, 78.74, 77.59, 77.50, 77.45, 76.39, 75.21, 75.03, 74.98, 71.72, 71.62, 68.69, 68.59, 67.33, 67.30, 67.24, 67.19, 66.26, 66.08, 65.34, 63.94, 63.26, 62.96, 60.13, 60.10, 60.06, 56.34, 55.39, 54.92, 49.76, 49.52,

48.26, 25.10, 25.06, 22.30, 21.97, 21.94, 15.51. HR-MS: Calculated for $C_{78}H_{121}N_9O_{46}$ $[M+2H]^{2+}/2$: 960.8776, found: 960.8769.

Nonasaccharide (62)

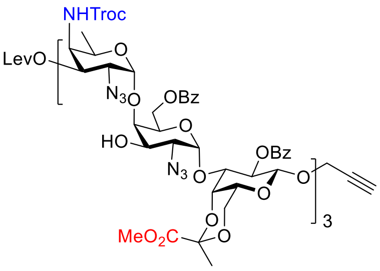

Compound **60** (245.5 mg, 76.1 μmol, 1.0 eq) was dissolved in THF (1.0 mL) and pyridine (1.0 mL), then cooled to 0 °C. Hydrogen fluoride (HF)/pyridine (70%) (0.1 mL) was added dropwise. The solution was stirred for 6 hours. After TLC showed complete consumption of the starting material, the reaction was quenched with saturated aqueous sodium bicarbonate slowly and diluted with EtOAc. The solution was washed with water (2x) and brine. The aqueous layer was extracted with EtOAc (3x), dried with $MgSO_4$, filtered, and concentrated *in vacuo*. The residue was purified by flash size exclusion (LH-20) (DCM/MeOH 1:1). The crude was dissolved in DCM (2.0 mL). Et_3N (191 μL, 1.37 mmol, 18.0 eq) and $PhCO_2Bt$ (273 mg, 1.14 mmol, 15 eq) were added and stirred at RT for overnight. The reaction was quenched by MeOH and concentrated *in vacuo*. The compound was purified by flash chromatography (DCM/Acetone 8:1 - 5:1) to yield compound **62** (199 mg, 0.064 mmol, 84%). ^{1}H NMR (500 MHz, $CDCl_3$) δ 8.03 – 7.79 (m, 12H), 7.68 – 7.56 (m, 3H), 7.53 – 7.40 (m, 6H), 7.27 – 7.10 (m, 9H), 5.61 – 5.48 (m, 3H), 5.42 – 5.24 (m, 4H), 5.24 – 5.11 (m, 4H), 5.04 – 4.94 (m, 2H), 4.83 – 4.63 (m, 6H), 4.61 – 4.08 (m, 24H), 4.01 – 3.54 (m, 27H), 3.41 – 3.08 (m, 9H), 2.98 (d, *J* = 6.8 Hz, 1H), 2.86 – 2.44 (m, 4H), 2.31 (t, *J* = 2.4 Hz, 1H), 2.14 – 2.21 (m, 3H), 1.70 – 1.56 (m, 9H), 1.20 – 1.09 (m, 9H). ^{13}C NMR (126 MHz, $CDCl_3$) δ 206.43, 172.16, 170.70, 170.58, 170.56, 165.47, 165.43, 164.99, 164.91, 155.22, 155.10, 133.51, 133.27, 129.75, 129.71, 129.63, 129.53, 129.50, 129.37, 128.68, 128.66, 128.49, 101.79, 101.71, 99.45, 99.15, 98.99, 98.79, 98.76, 98.34, 95.68, 95.54, 93.85, 93.77, 93.60, 78.74, 75.85, 75.22, 74.82, 74.70, 73.38, 73.11, 70.92, 69.79, 69.59, 68.88, 68.77, 67.09, 67.03, 66.17, 66.06, 65.95, 65.77, 65.42, 64.84, 62.00, 60.15, 59.97, 58.27, 55.65, 52.95, 52.91, 52.85, 52.81, 37.87, 29.88, 27.99, 25.65, 25.57, 16.37, 16.33. HR-MS: Calculated for $C_{125}H_{136}Cl_9N_{21}O_{54}$ $[M+2(NH_4)]^{2+}/2$: 1572.8207, found: 1572.8202. $[\alpha]^{20}_D$ = + 104.0° (c = 0.1, $CHCl_3$). TLC: Rf = 0.4 (DCM/Acetone = 6/1, v/v).

Dodecasaccharide (64)

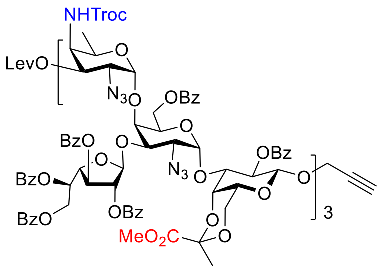

Donor **10** (164 mg, 0.214 mmol, 9.0 eq) and acceptor **62** (73.8 mg, 23.7 µmol, 1.0 eq) were co-evaporated with toluene (3×) and placed under a nitrogen atmosphere. Dry DCM (2.0 mL) and 4Å molecular sieves were added and the solution stirred for 30 minutes before being reduced to 0 °C. TBSOTf (2.0 µL, 8.7 µmol, 0.2 eq) was added to the reaction and stirred 6 hours. After analysis by TLC showed complete consumption of the starting material, the reaction was quenched with triethylamine and concentrated *in vacuo*. The compound was purified by flash chromatography (DCM/Acetone 12:1 - 10:1) to yield compound **64** (97.3 mg, 20 µmol, 85%). ^{1}H NMR (400 MHz, $CDCl_3$) δ 8.18 – 7.77 (m, 36H), 7.65 – 7.11 (m, 54H), 5.95 – 5.82 (m, 3H), 5.69 – 5.50 (m, 12H), 5.48 – 5.40 (m, 2H), 5.36 (s, 1H), 5.13 (dd, *J* = 11.2, 3.8 Hz, 1H), 5.07 – 4.99 (m, 2H), 4.93 (d, *J* = 9.4 Hz, 1H), 4.86 – 4.19 (m, 36H), 4.19 – 4.09 (m, 4H), 4.06 – 3.70 (m, 25H), 3.69 – 3.56 (m, 3H), 3.34 – 3.18 (m, 3H), 3.14 – 3.05 (m, 1H), 2.88 – 2.43 (m, 4H), 2.34 – 2.29 (m, 1H), 2.22 – 2.16 (m, 3H), 1.69 – 1.53 (m, 9H), 1.24 – 1.11 (m, 9H). ^{13}C NMR (101 MHz, $CDCl_3$) δ 206.43, 172.07, 170.70, 170.60, 170.47, 166.07, 165.98, 165.96, 165.77, 165.75, 165.72, 165.67, 165.60, 165.53, 165.45, 165.39, 165.03, 164.89, 155.11, 155.07, 133.89, 133.76, 133.48, 130.10, 130.02, 129.98, 129.94, 129.89, 129.83, 129.77, 129.71, 129.67, 129.60, 129.52, 129.28, 129.09, 128.97, 128.87, 128.82, 128.75, 128.72, 128.65, 128.63, 128.57, 128.48, 107.54, 101.63, 99.42, 99.00, 98.80, 98.77, 98.36, 95.83, 95.67, 93.27, 93.06, 81.32, 81.11, 80.95, 80.58, 78.72, 77.55, 77.36, 77.28, 75.53, 75.20, 74.98, 74.81, 74.71, 74.53, 73.08, 72.86, 71.02, 70.27, 69.81, 69.57, 66.22, 65.93, 65.74, 65.44, 65.24, 64.95, 62.77, 60.28, 59.03, 58.80, 58.67, 55.67, 55.09, 53.87, 53.12, 52.90, 52.80, 52.75, 37.89, 29.93, 29.37, 27.99, 25.63, 16.70, 16.57. HR-MS: Calculated for $C_{227}H_{214}Cl_9N_{21}O_{81}$ $[M+2(NH_4)]^{2+}/2$: 2440.5693, found: 2440.5589. $[\alpha]^{20}_{D}$ = + 77.0° (c = 0.1, $CHCl_3$). TLC: Rf = 0.3 (DCM/Acetone = 10/1, v/v).

Dodecasaccharide (3a)

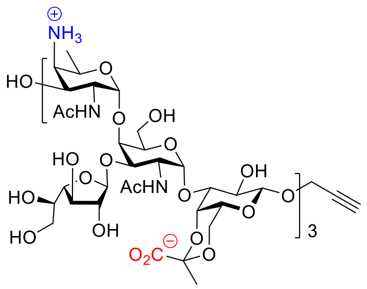

Compound **64** (97.3 mg, 20 µmol, 1.0 eq) was dissolved in pyridine (1.0 mL) and acetic acid (1.0 mL). After cooling to 0 °C, hydrazine acetate (N_2H_4•AcOH) (10.0 mg, 108.6 µmol, 5.0 eq) was added. After stirring 4 hours at RT, TLC analysis showed complete consumption of the starting material and the reaction was quenched with acetone. The reaction was concentrated *in vacuo* and tyhe residue was purified by size exclusion chromatography (LH-20) (DCM/MeOH 1:1). The product was dissolved in DCM (1.0 mL) and 3,4-dihydro-2H-pyran (0.1 mL). Then pyridinium *p*-toluenesulfonate (6.0 mg, 24.0 µmol, 1.2 eq) was added and the mixture was stirred at RT overnight. After analysis by TLC showed complete consumption of the starting material, the reaction was quenched with triethylamine and purified by size exclusion chromatography (LH-20) (DCM/MeOH 1:1). The product was dissolved in THF (1.0 mL) and water (40 µL). Pyridine (160 µL, 2.0 mmol, 100 eq) and Ph_3P (120 mg, 0.46 mmol, 23 eq) were added and the reaction was allowed to stir overnight at 70 °C. After TLC showed complete consumption of the starting material, THF (1.0 ml) and water (1.0 mL) were added to the reaction. Then sodium bicarbonate (360 mg, 4.3 mmol, 200 eq) and acetic anhydride (200 µL, 2.12 mmol, 100 eq) were added and stirred for overnight. After TLC showed complete consumption of the starting material, the reaction mixture was diluted with EtOAc and then washed with saturated aqueous sodium bicarbonate and brine. The aqueous layer was extracted with EtOAc (3x), dried with $MgSO_4$, filtered, and concentrated *in vacuo*. The residue was purified by size exclusion chromatography (LH-20) (DCM/MeOH 1:1). The crude was dissolved in dioxane (2.0 mL), THF (2.0 mL), water (2.0 mL). LiOH (30 mg, 1.25 mmol, 0.2M) was added and stirred at RT. After analysis by TLC and LC-MS showed complete consumption of the starting material and intermediates, the reaction was quenched with 1M HCl to neutral. Acetic acid (8 mL) was added and heated to 50 °C. After analysis by LC-MS showed complete consumption of the starting material, the solvent was removed through co-evaporation with toluene *in vacuo*. The compound was purified by gel filtration (HW-40, 0.15M NH_4HCO_3 in H_2O) with a Shimadzu RID-10A refractive index detector and lyophilized to yield compound **3a** (24 mg, 10 µmol, 50% over six steps). ^{1}H NMR (500 MHz, D_2O) δ 5.37 – 5.28 (m, 3H), 5.12 – 5.01 (m, 6H), 4.76 – 4.73 (m, 3H), 4.72 – 4.65 (m, 3H), 4.63 – 4.55 (m, 3H), 4.54 – 4.44 (m, 7H), 4.41 (dd, *J* = 11.4, 4.4 Hz, 1H), 4.30 – 4.17 (m, 8H), 4.16 – 3.91 (m, 21H), 3.86 – 3.54 (m, 25H), 2.93 – 2.89 (m, 1H), 2.12 – 2.01 (m, 18H), 1.52 – 1.42 (m, 9H), 1.38 – 1.28 (m,

9H). ^{13}C NMR (126 MHz, D_2O) δ 175.26, 174.98, 174.76, 174.67, 174.65, 108.96, 104.47, 101.30, 101.12, 100.41, 98.00, 97.93, 97.80, 94.27, 94.24, 93.97, 81.85, 81.77, 80.67, 78.77, 77.50, 77.38, 76.41, 75.71, 75.67, 75.34, 74.98, 72.00, 71.93, 71.82, 70.52, 70.48, 68.66, 68.55, 67.44, 67.24, 66.26, 66.12, 65.36, 64.93, 63.71, 63.21, 62.98, 62.69, 60.25, 60.24, 60.20, 56.38, 55.43, 54.91, 50.12, 48.79, 48.65, 25.12, 25.07, 22.35, 22.00, 21.95, 16.16. HR-MS: Calculated for $C_{96}H_{151}N_9O_{61}$ $[M+3H]^{3+}/3$: 802.9736, found: 802.9731.

References

1. Wang, Z.; Gimeno, A.; Lete, M. G.; Overkleeft, H. S.; van der Marel, G. A.; Chiodo, F.; Jiménez-Barbero, J.; Codée, J. D. C., Synthetic Zwitterionic Streptococcus pneumoniae Type 1 Oligosaccharides Carrying Labile O-Acetyl Esters. *Angew. Chem. Int. Ed.* **2022**, e202211940.

2. Hagen, B.; van Dijk, J. H. M.; Zhang, Q.; Overkleeft, H. S.; van der Marel, G. A.; Codée, J. D. C., Synthesis of the Staphylococcus aureus Strain M Capsular Polysaccharide Repeating Unit. *Org. Lett.* **2017**, *19*, 2514-2517.

3. Zhang, M.; Wang, Y.; Yang, Y.; Hu, X., An Alternative Approach to Direct Aldol Reaction Based on Gold-Catalyzed Methoxyl Transfer. *Adv. Synth. Catal.* **2012**, *354,* 981-985.

4. Pathan, E. K.; Ghosh, B.; Podilapu, A. R.; Kulkarni, S. S., Total Synthesis of the Repeating Unit of Bacteroides fragilis Zwitterionic Polysaccharide A1. *J. Org. Chem.* **2021**, *86*, 6090-6099.

zhen2007Biosyn.64.fid — wz499-c, column again — bbo-h1 CDCl3 /opt/topspin2.1 nmrafd 4

18

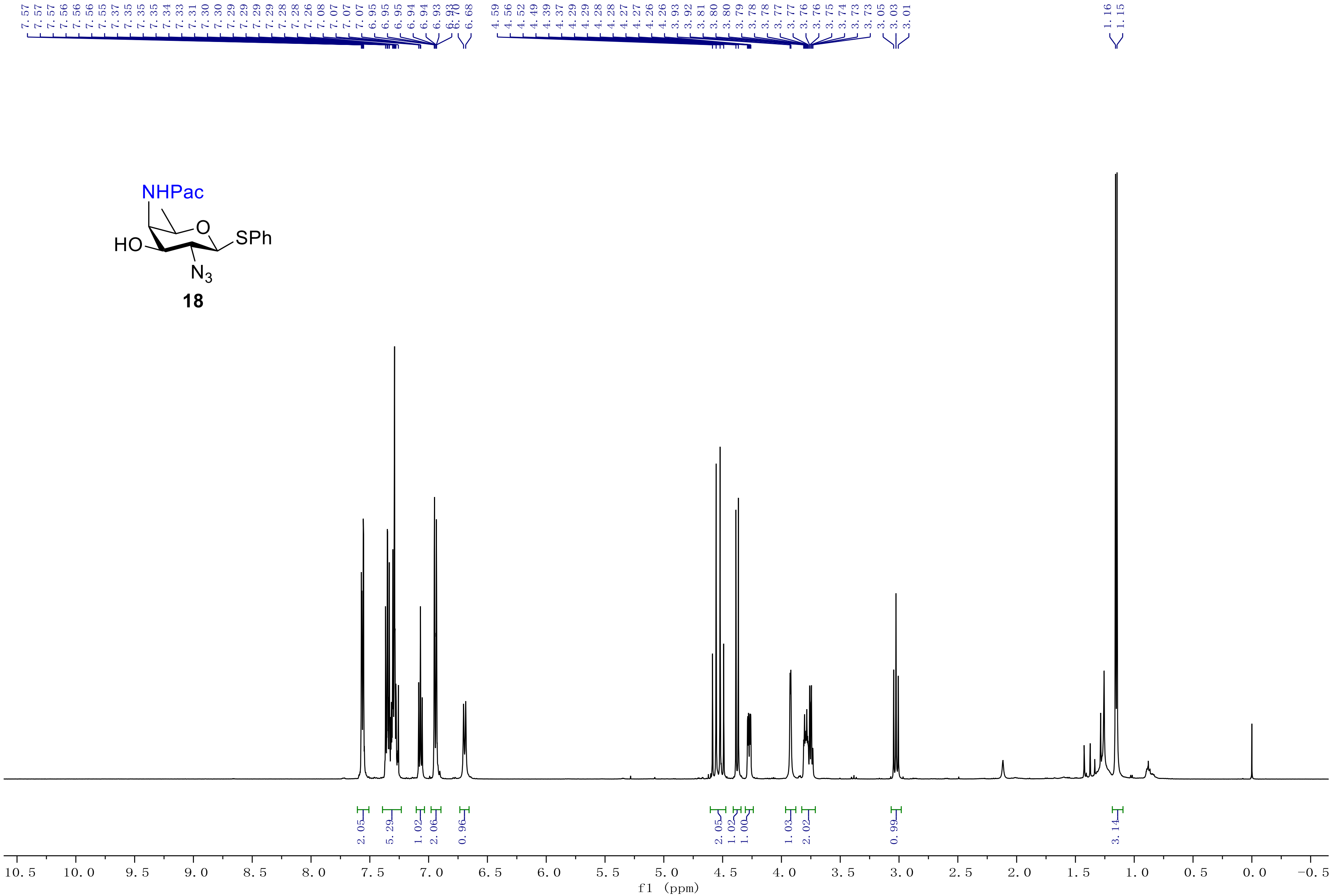

zhen2007Biosyn.67.fid — wz499-c, column again — bbo-c13-APT CDCl3 /opt/topspin2.1 nmrafd 4

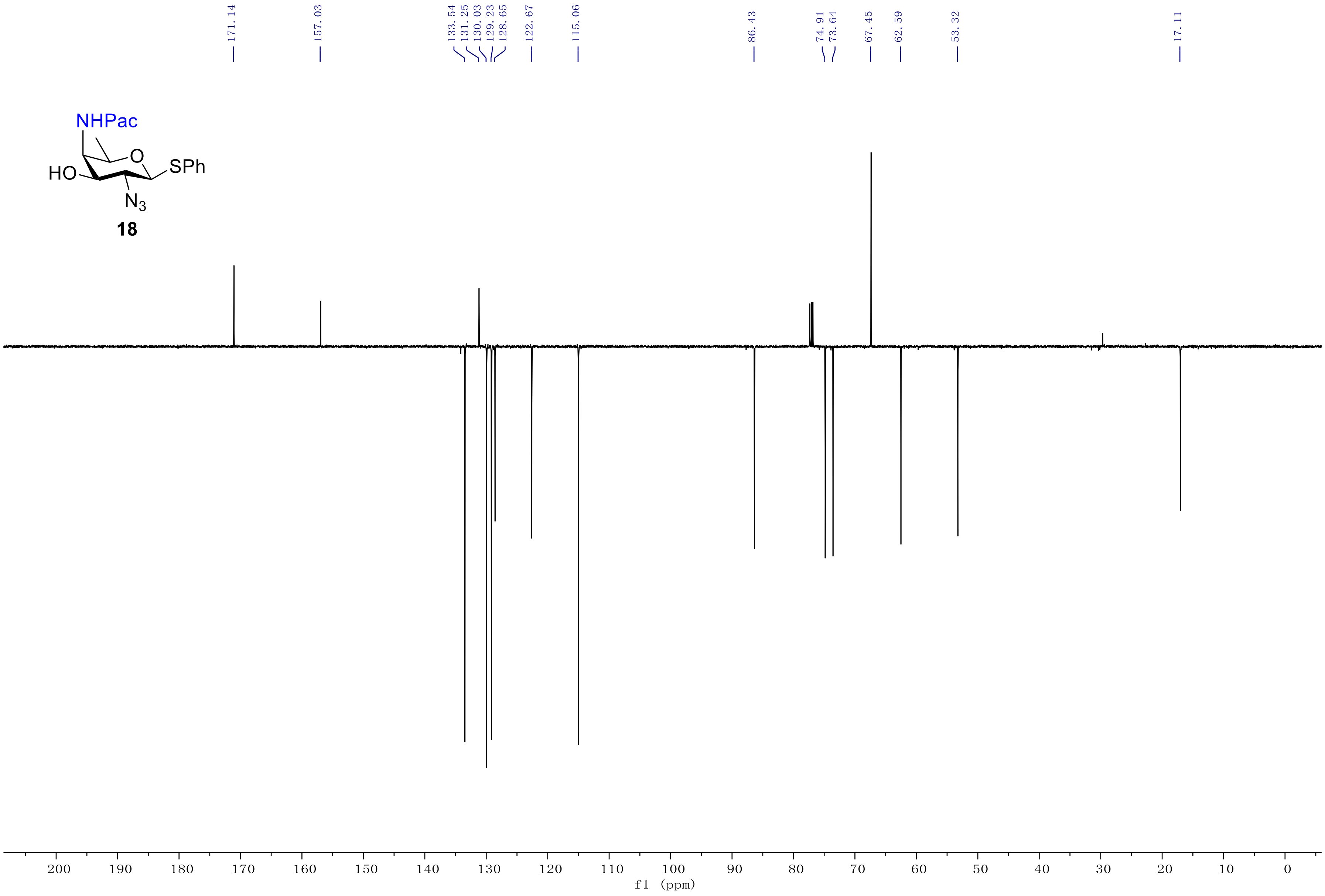

zhen2007Biosyn.65.ser — wz499-c, column again — bbo-h1-cosy CDCl3 /opt/topspin2.1 nmrafd 4

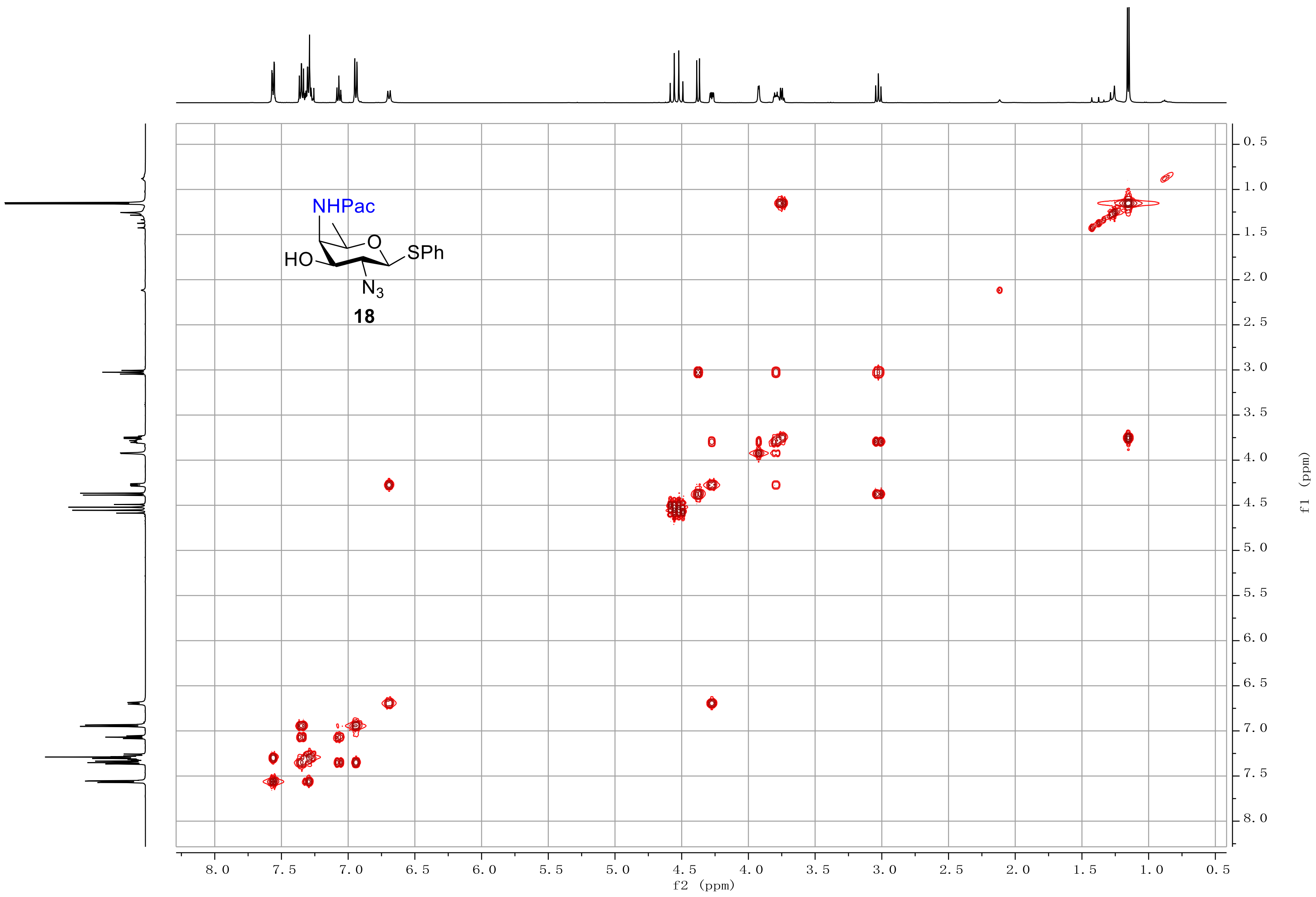

zhen2007Biosyn.66.ser — wz499-c, column again — bbo-c13-HSQC CDCl3 /opt/topspin2.1 nmrafd 4

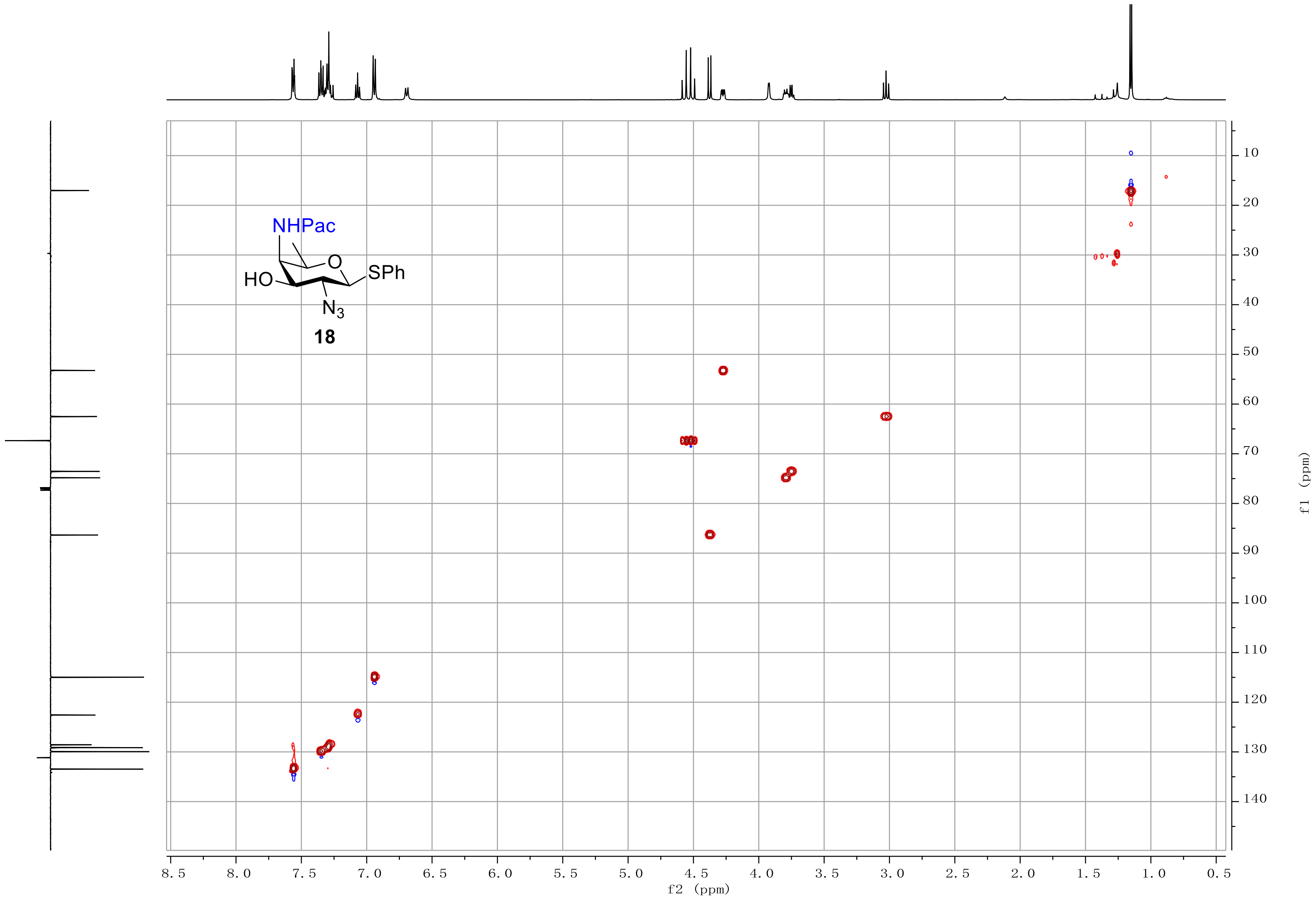

zhen2007Biosyn.68.ser — wz499-c, column again — bbo-c13-HMBC CDCl3 /opt/topspin2.1 nmrafd 4

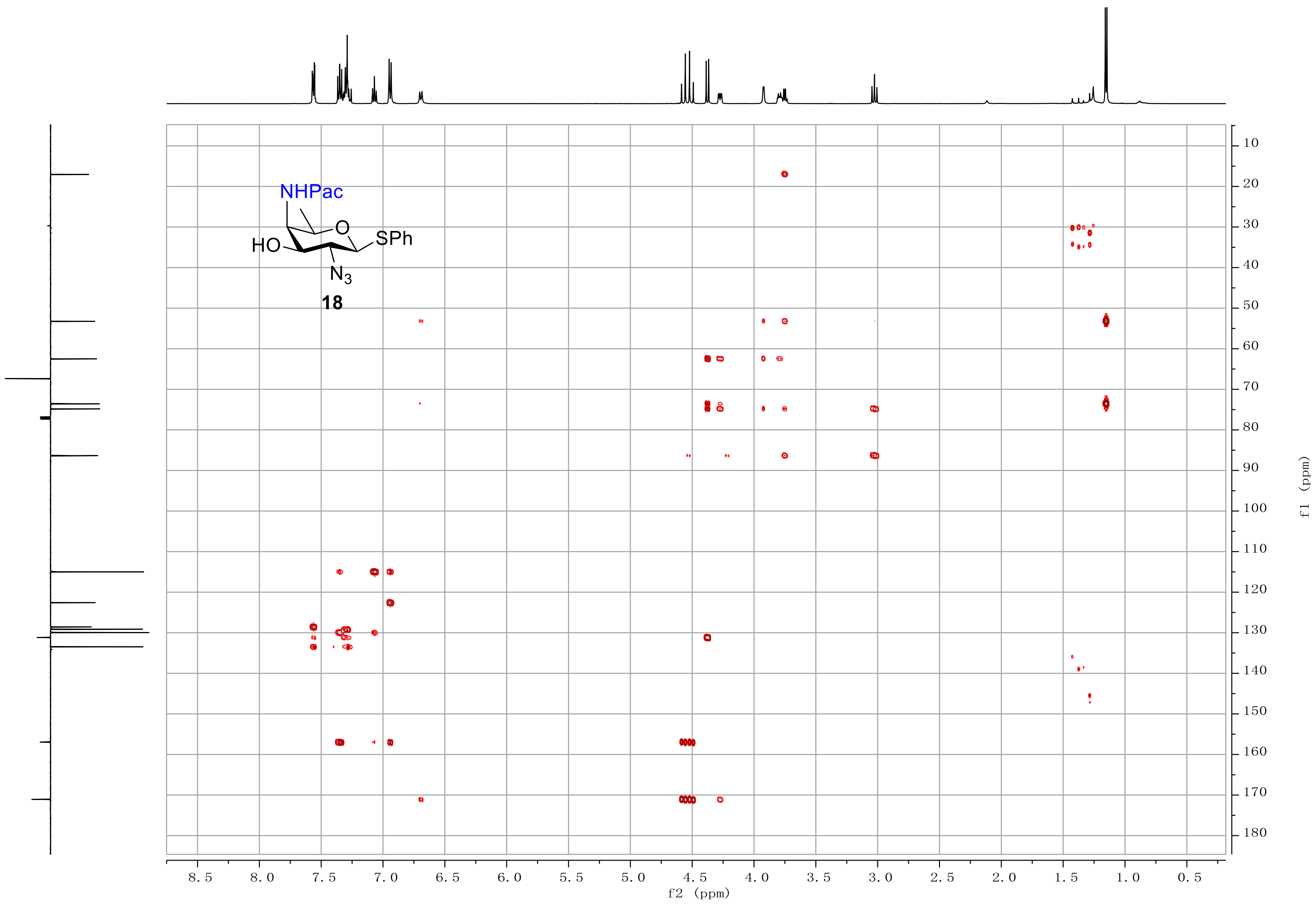

zhen2007Biosyn.36.fid — wz500-c — bbo-h1 CDCl3 /opt/topspin2.1 nmrafd 14

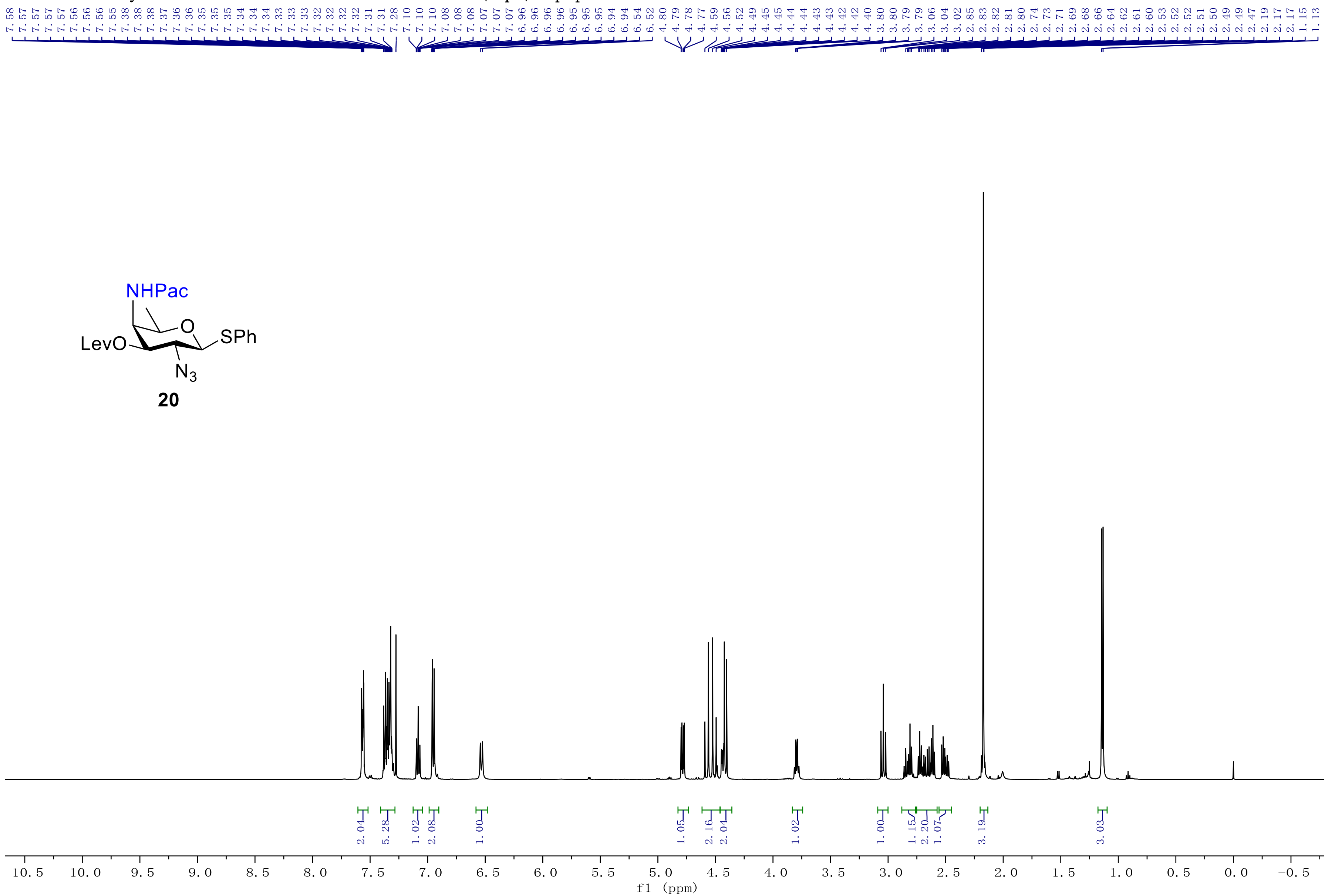

zhen2007Biosyn.39.fid — wz500-c — bbo-c13-APT CDCl3 /opt/topspin2.1 nmrafd 14

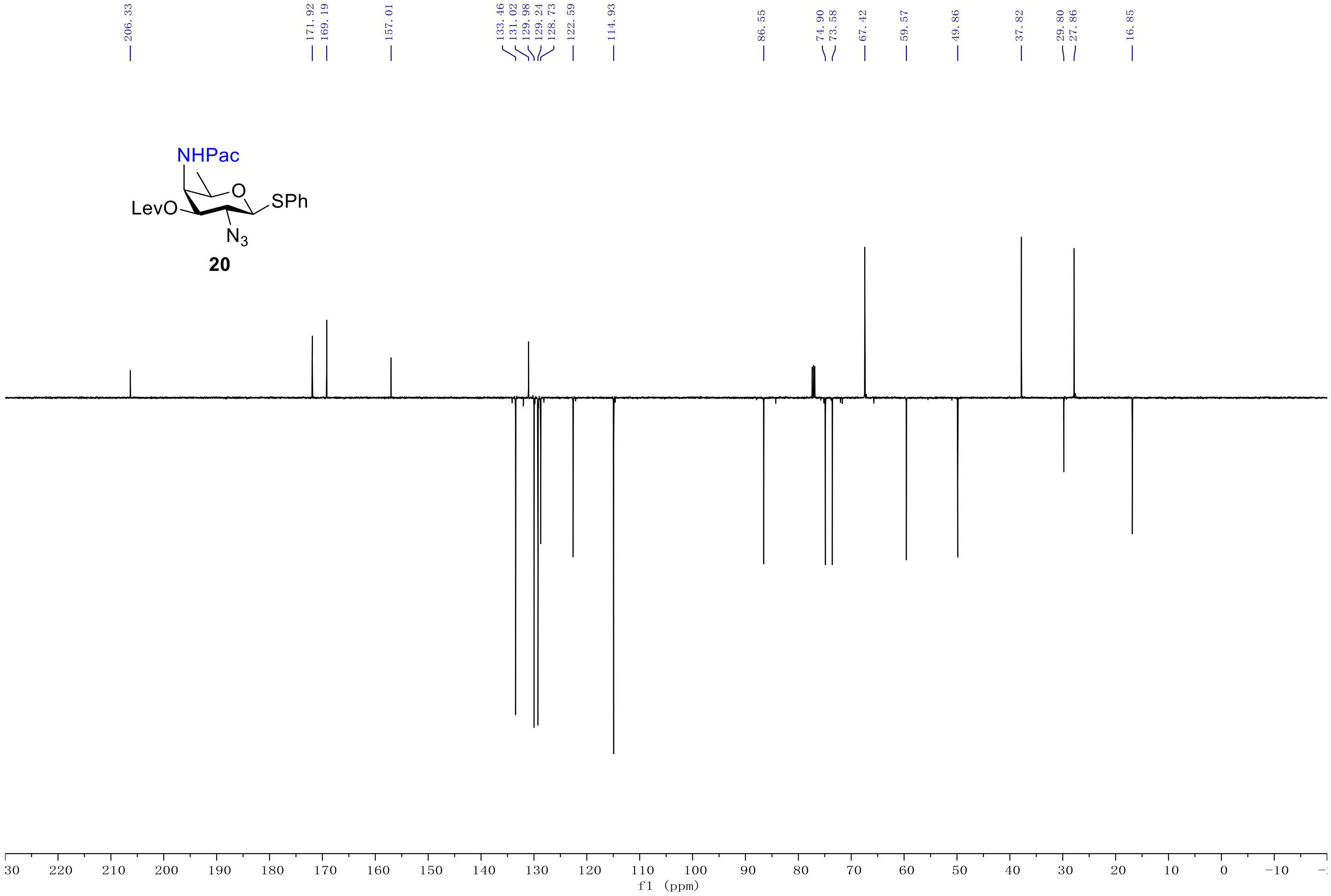

zhen2007Biosyn.37.ser — wz500-c — bbo-h1-cosy CDCl3 /opt/topspin2.1 nmrafd 14

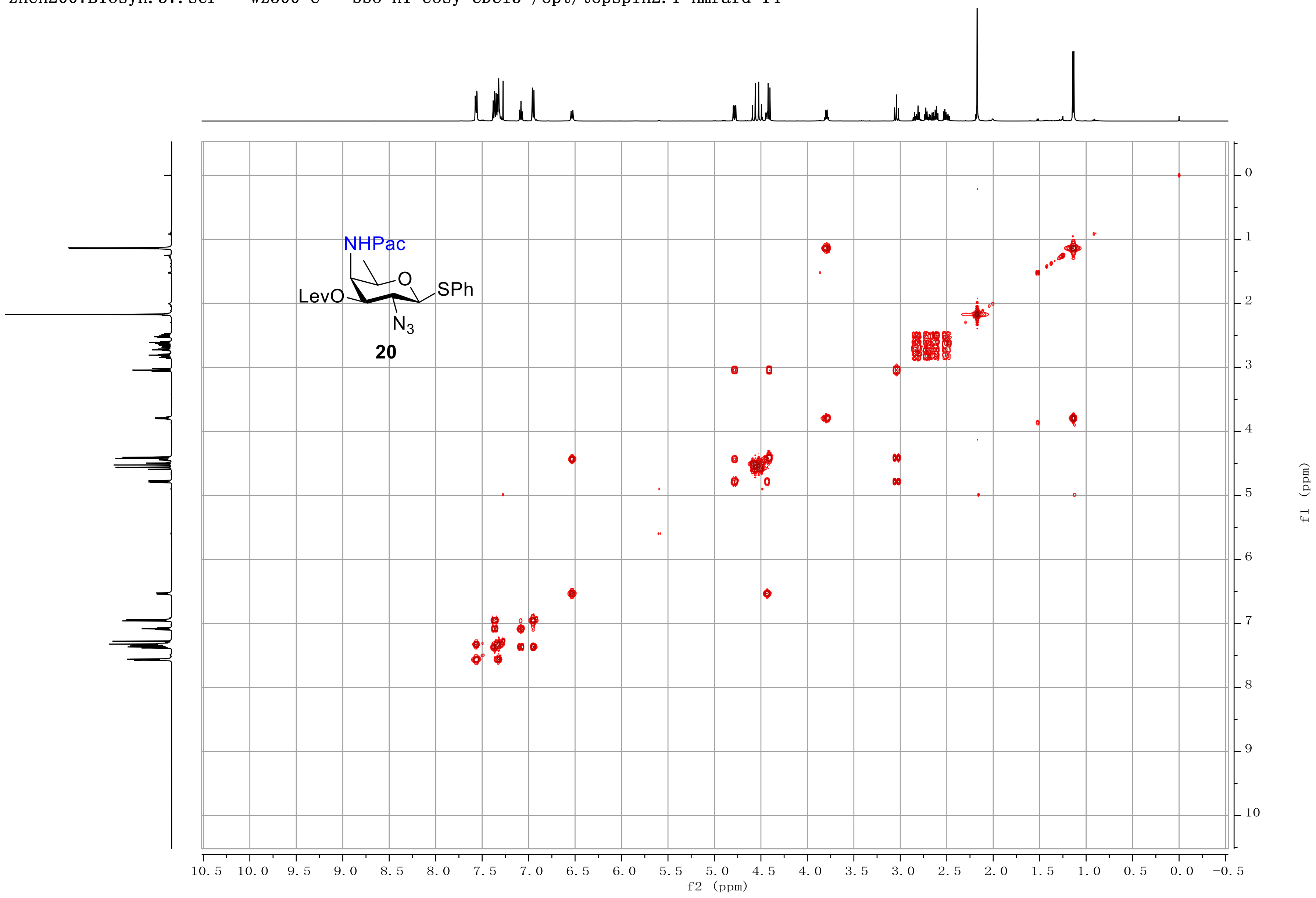

zhen2007Biosyn.38.ser — wz500-c — bbo-c13-HSQC CDCl3 /opt/topspin2.1 nmrafd 14

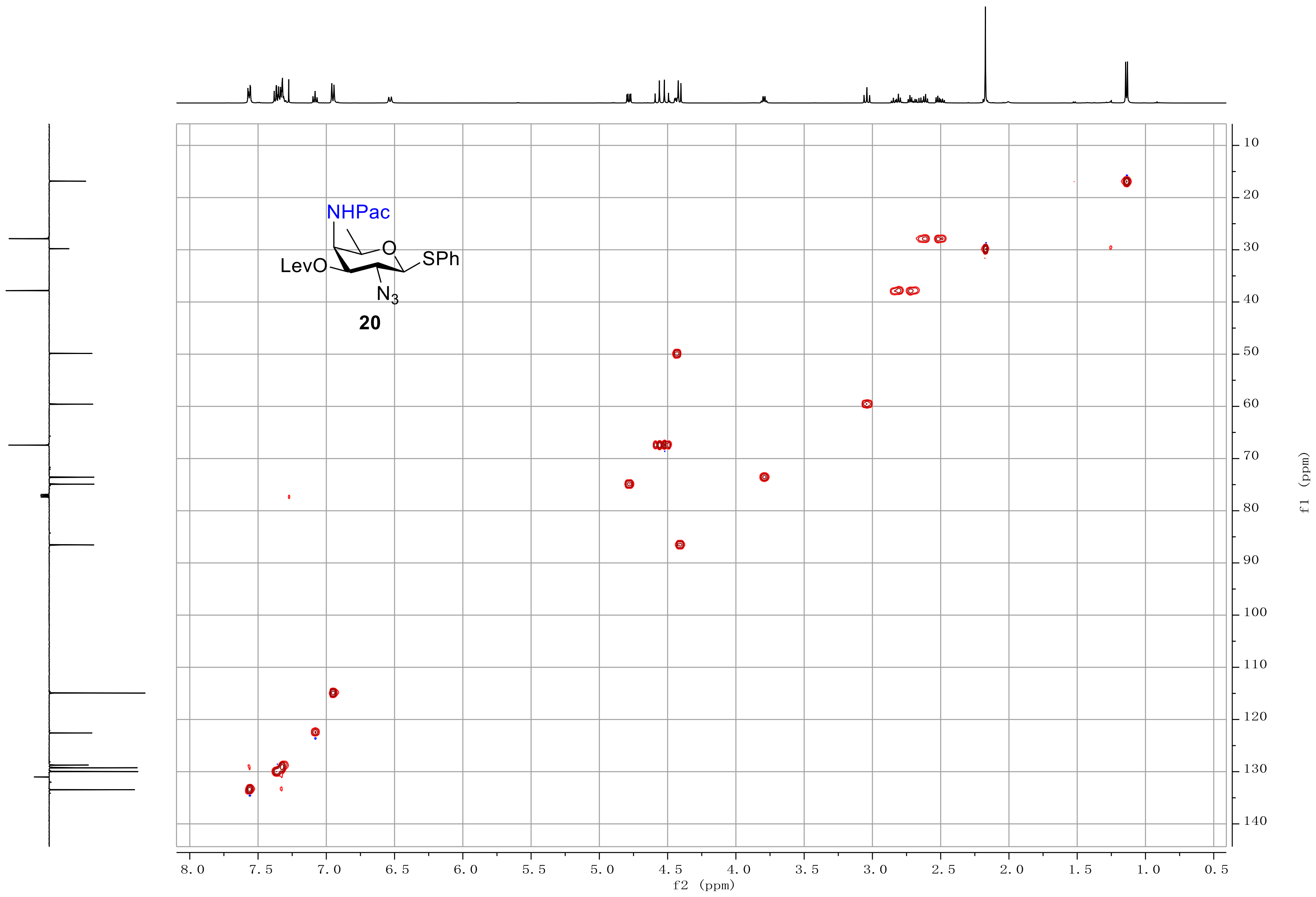

zhen2007Biosyn.40.ser — wz500-c — bbo-c13-HMBC CDCl3 /opt/topspin2.1 nmrafd 14

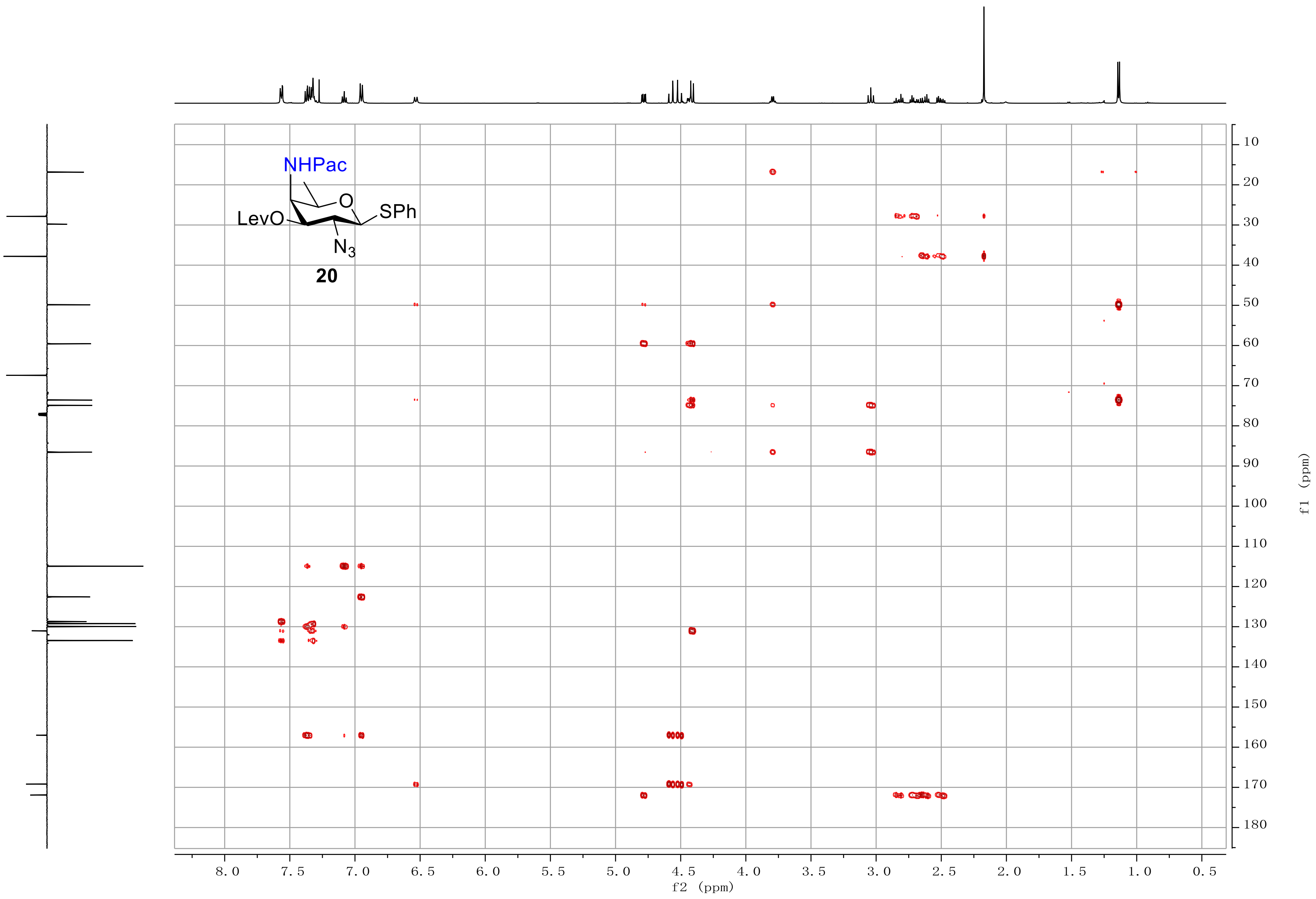

1903Connor.49.fid — CD109 — h1 CDCl3 /opt/DATA nmrafd 36

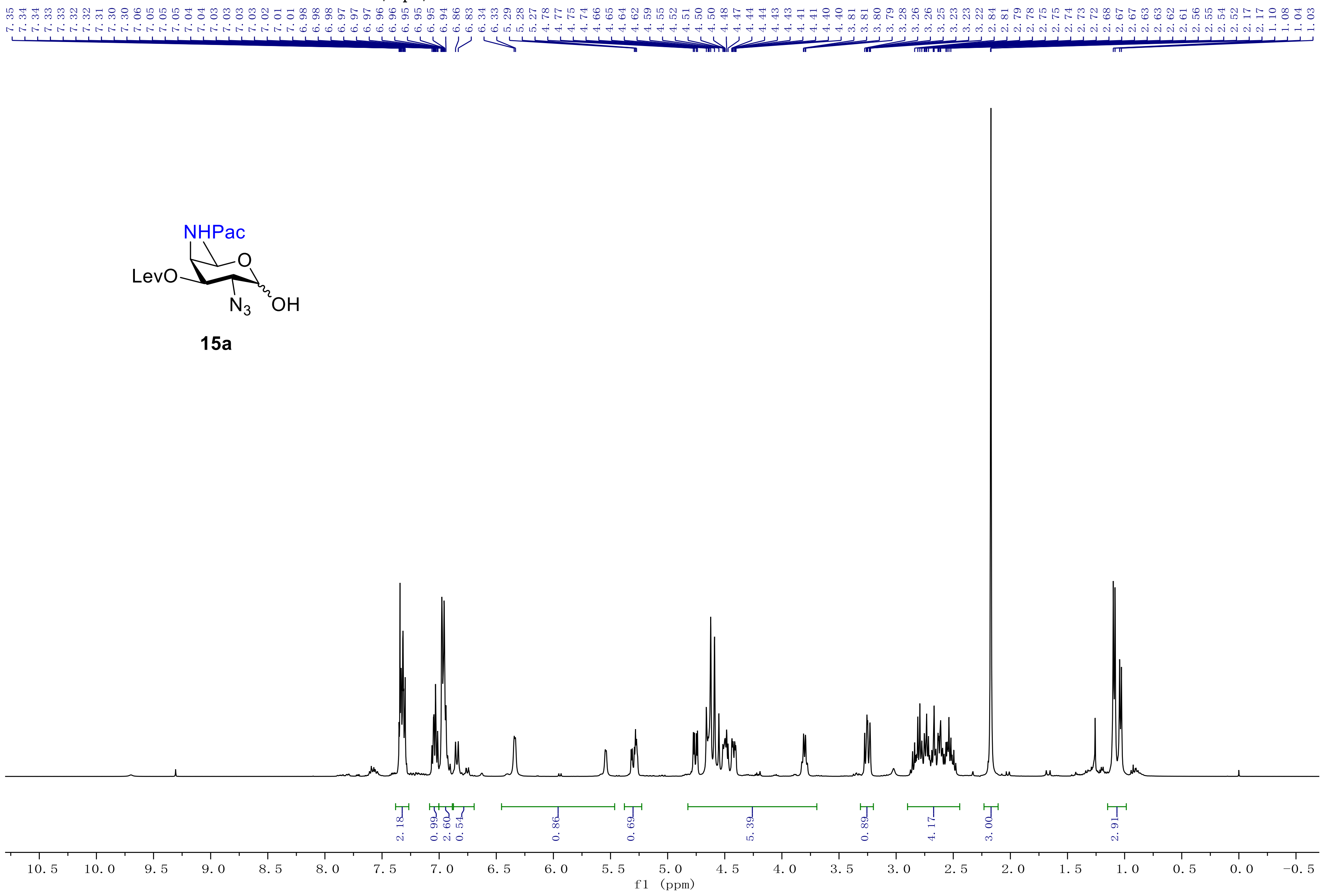

15a

1903Connor.50.fid — CD109 — C13APT CDCl3 /opt/DATA nmrafd 36

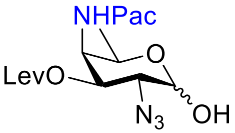

15a

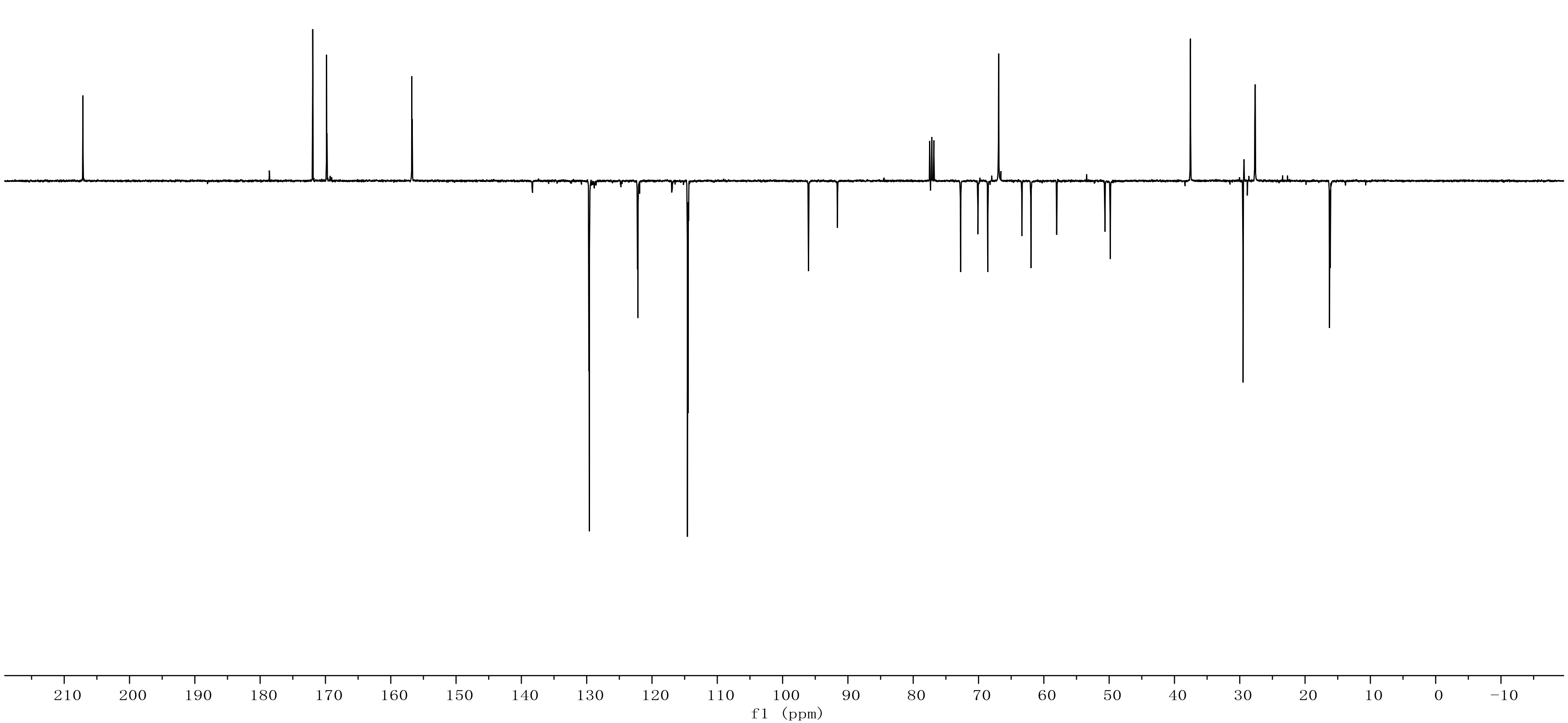

1903Connor.51.ser — CD109 — h1COSY CDCl3 /opt/DATA nmrafd 36

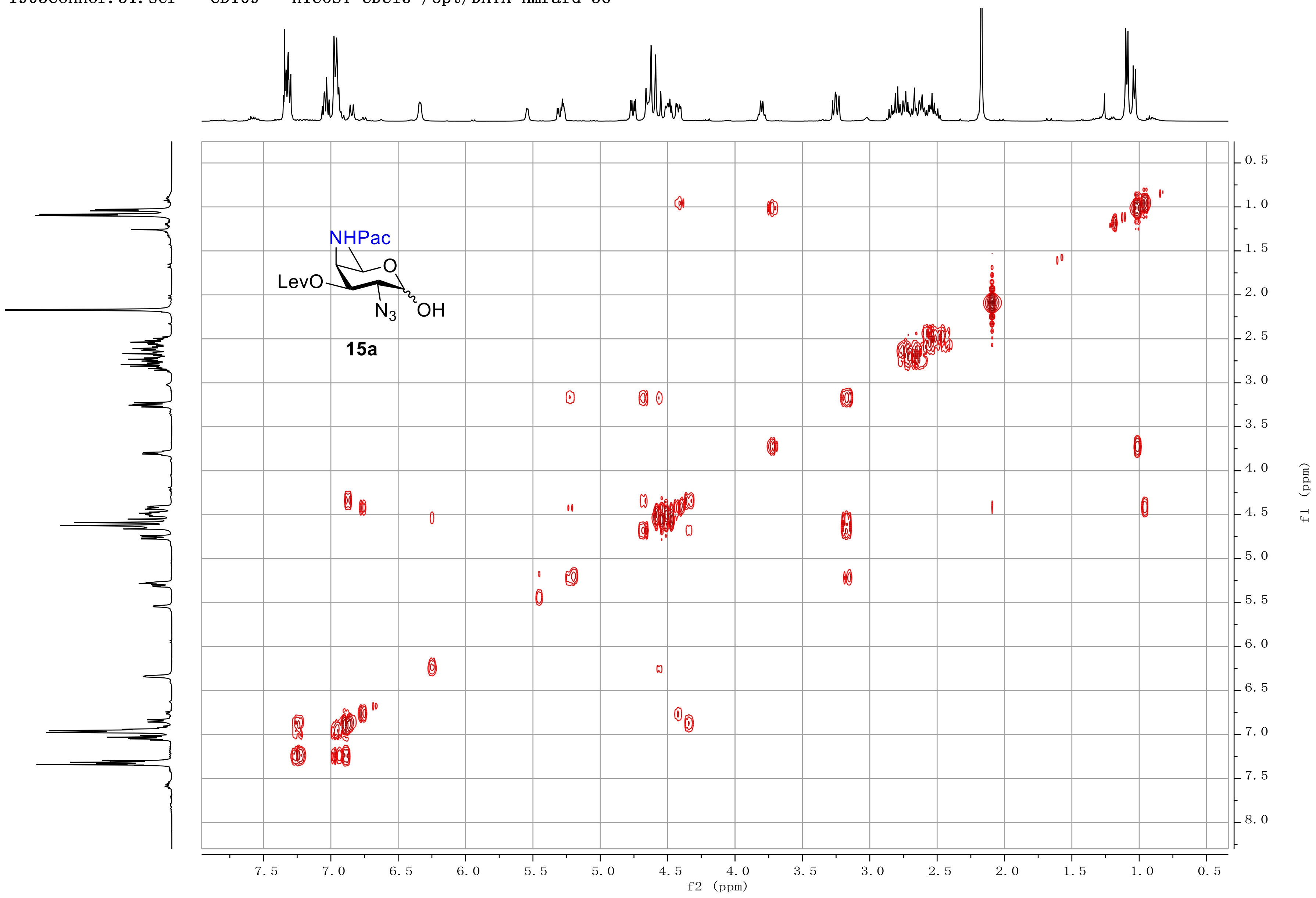

1903Connor.52.ser — CD109 — c13HSQC CDCl3 /opt/DATA nmrafd 36

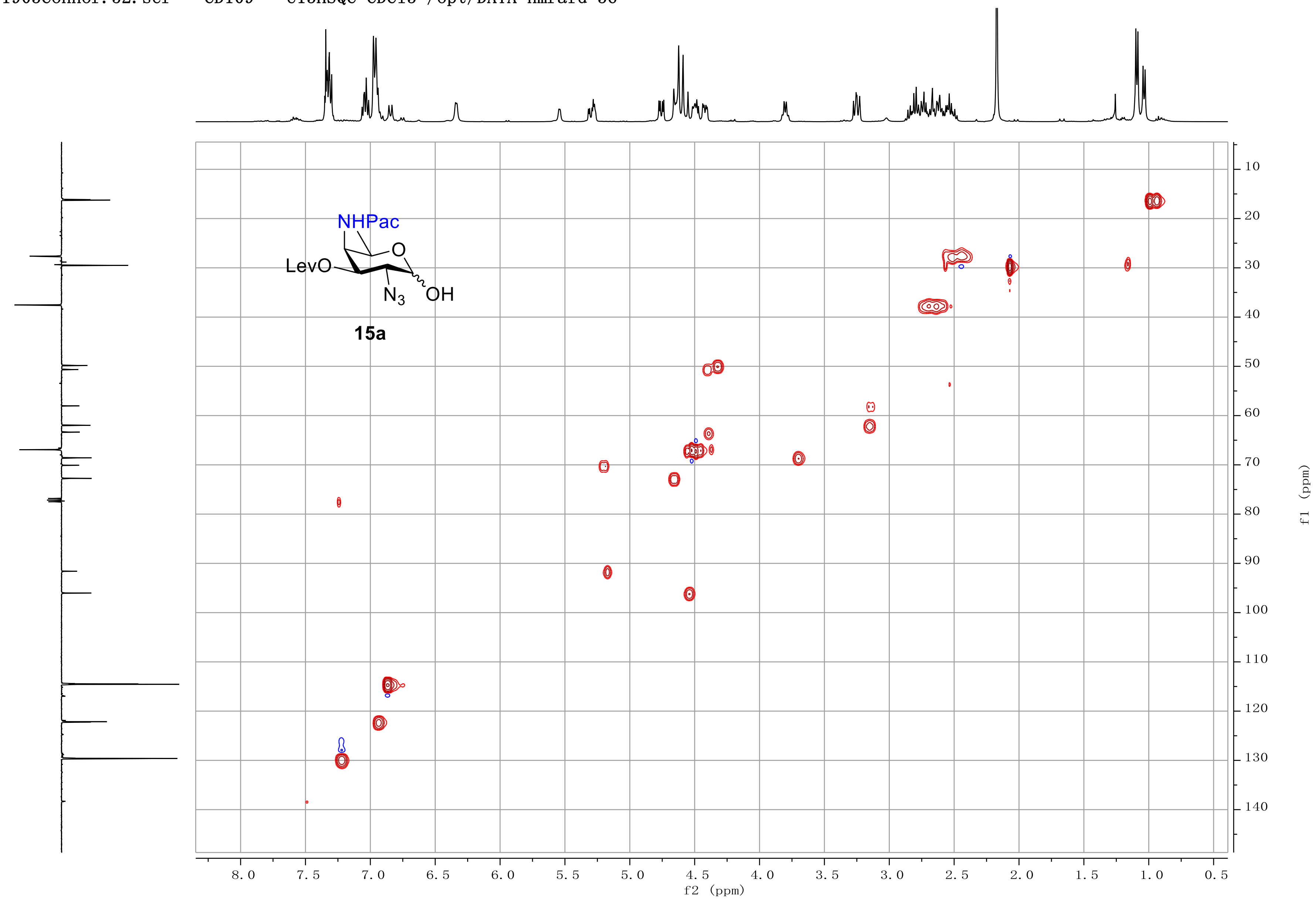

1903Connor.53.ser — CD109 — c13HMBC CDCl3 /opt/DATA nmrafd 36

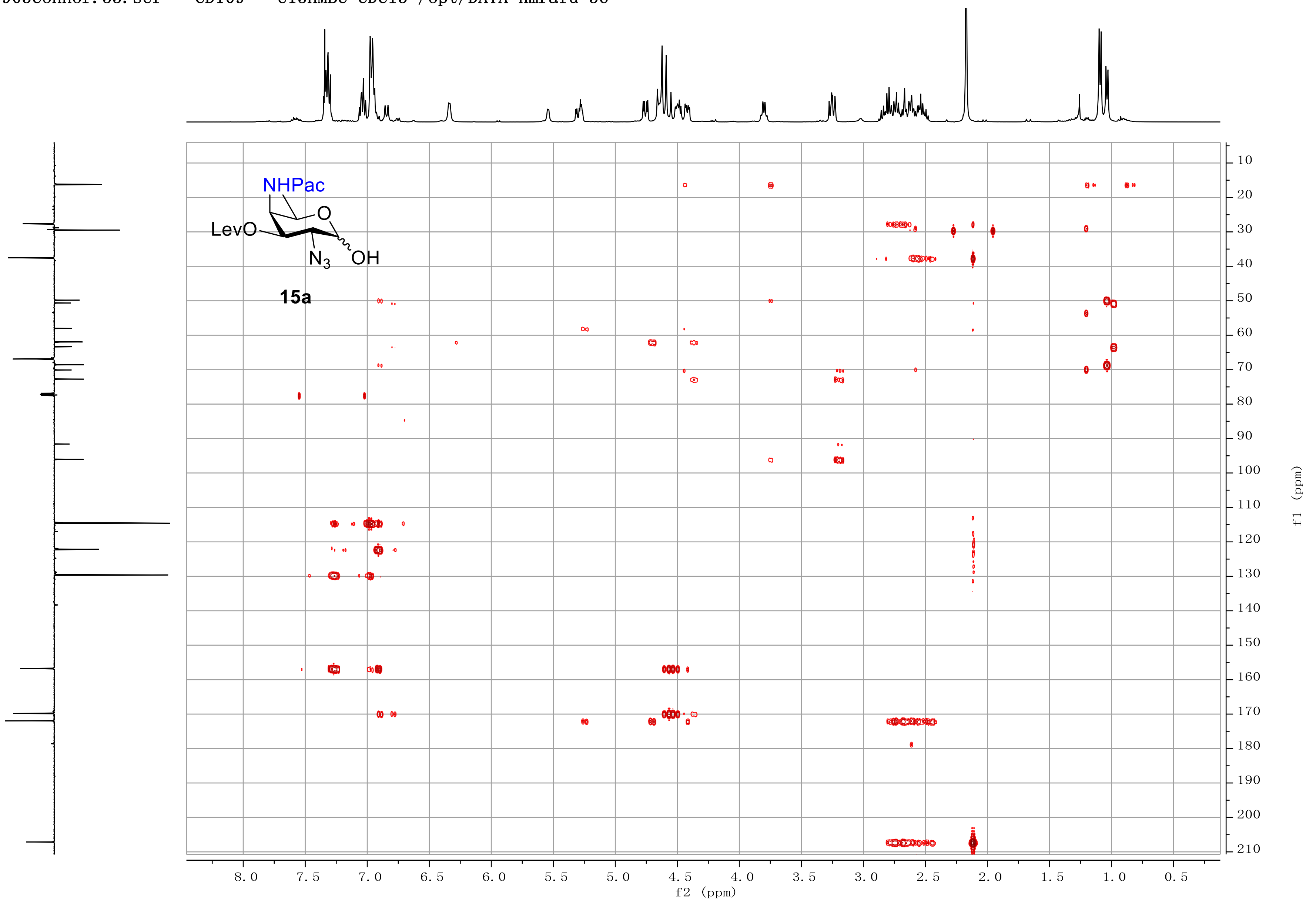

Qingju1807Biosyn.10.fid — 0735, aceton

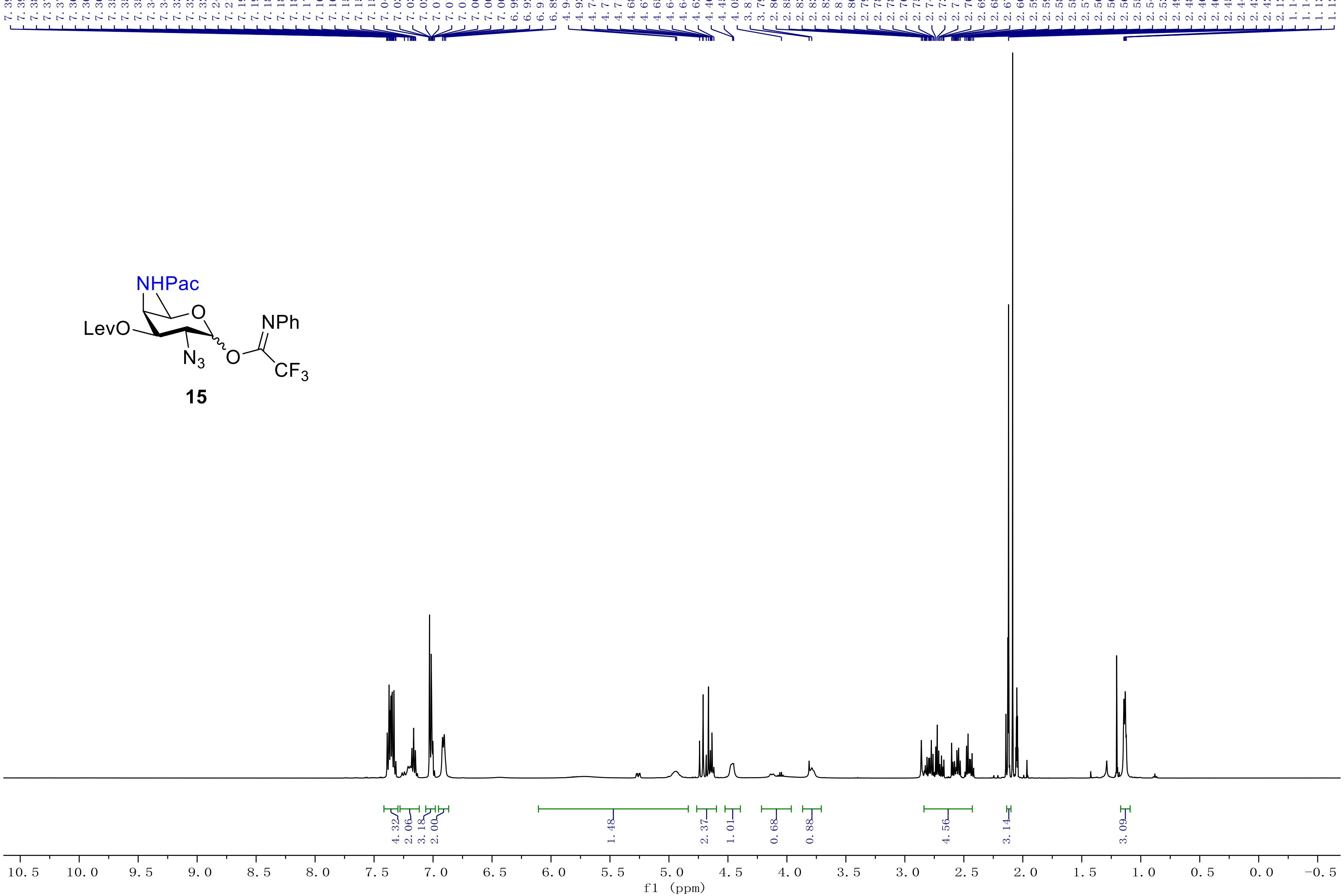

Qingju1807Biosyn.13.fid — 0735 — bbo-c13-APT Acetone /opt/topspin2.1 nmrafd 2

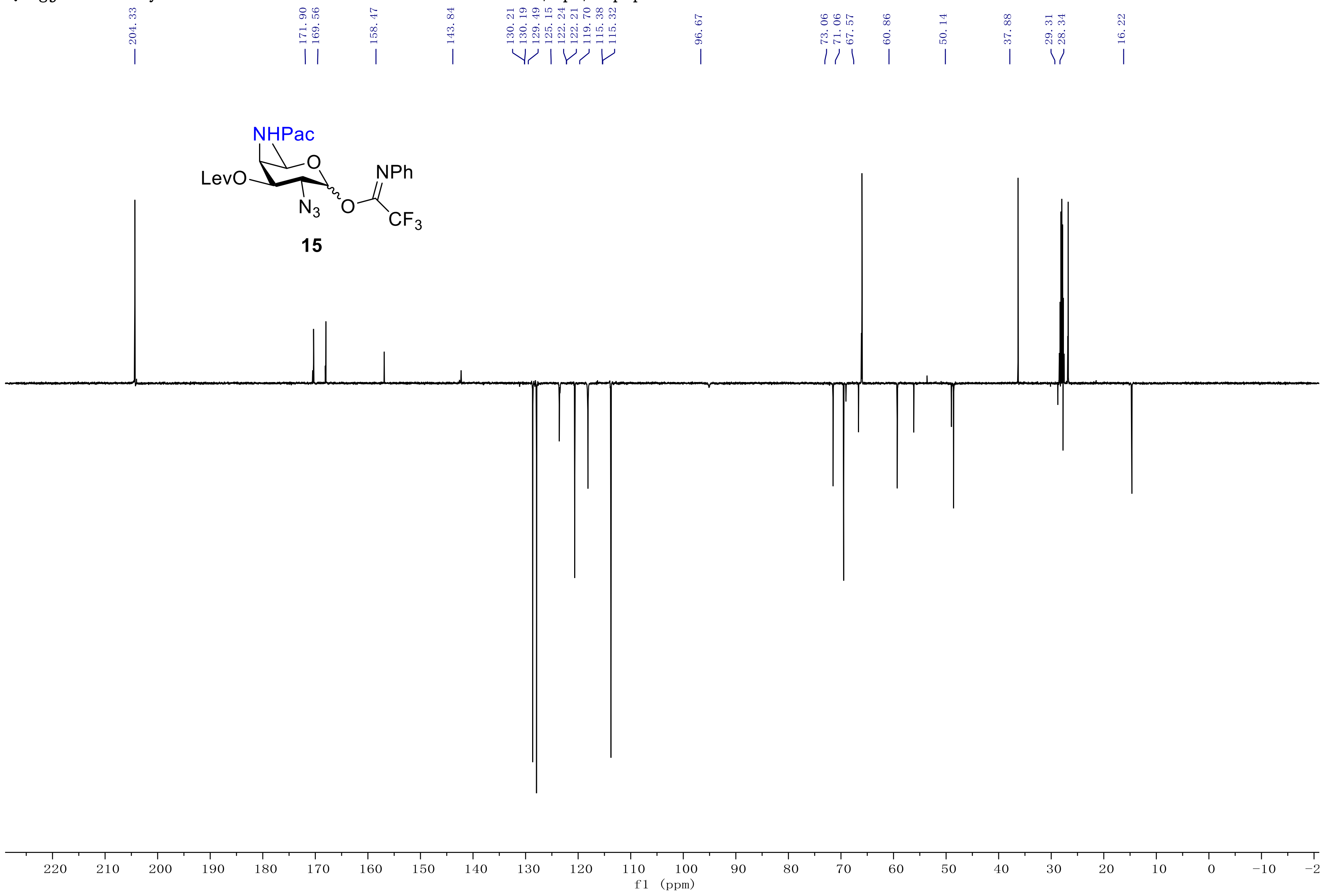

Qingju1807Biosyn.11.ser — 0735, cosy , aceton

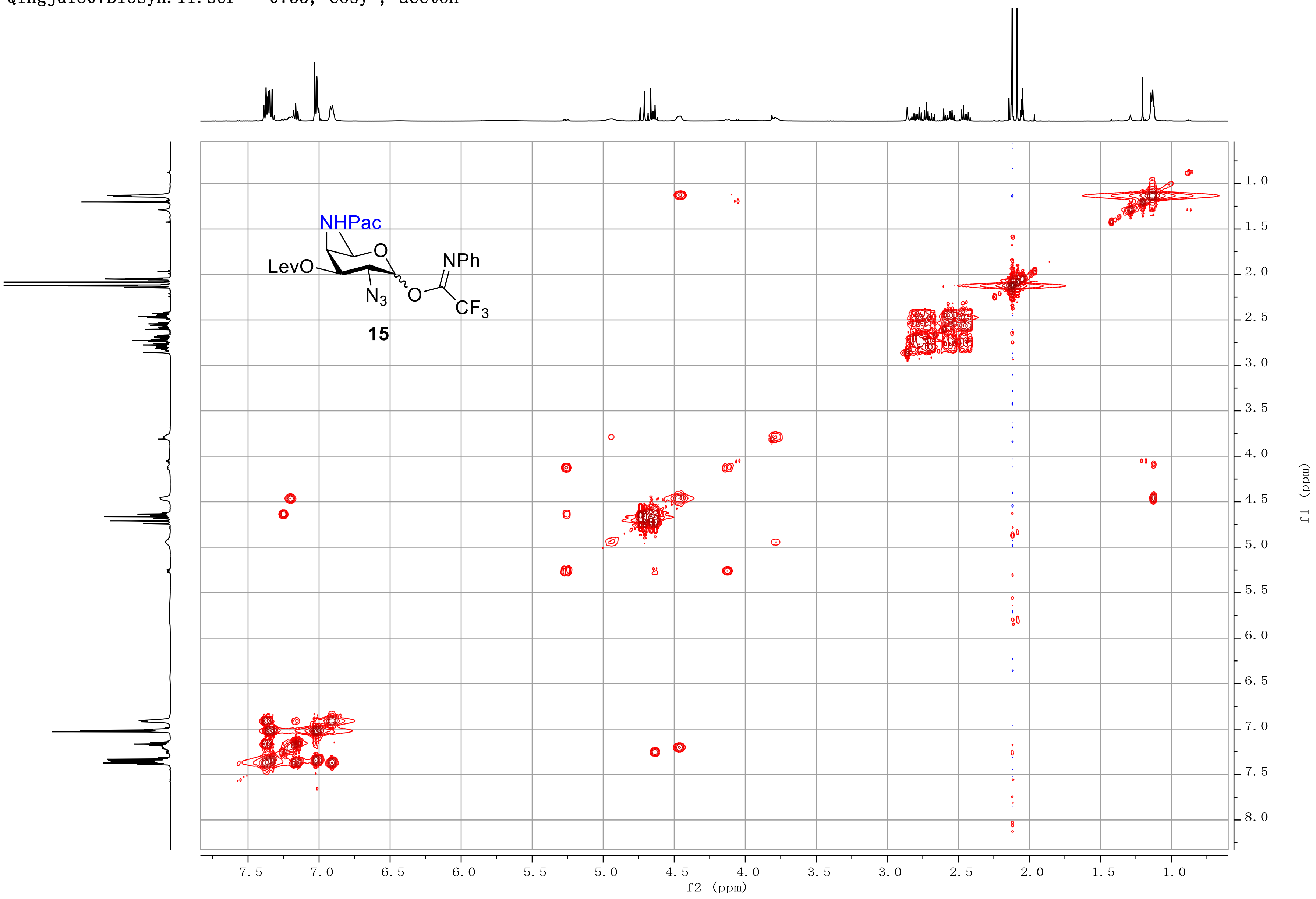

Qingju1807Biosyn.12.ser — 0735 — bbo-c13-HSQC Acetone /opt/topspin2.1 nmrafd 2

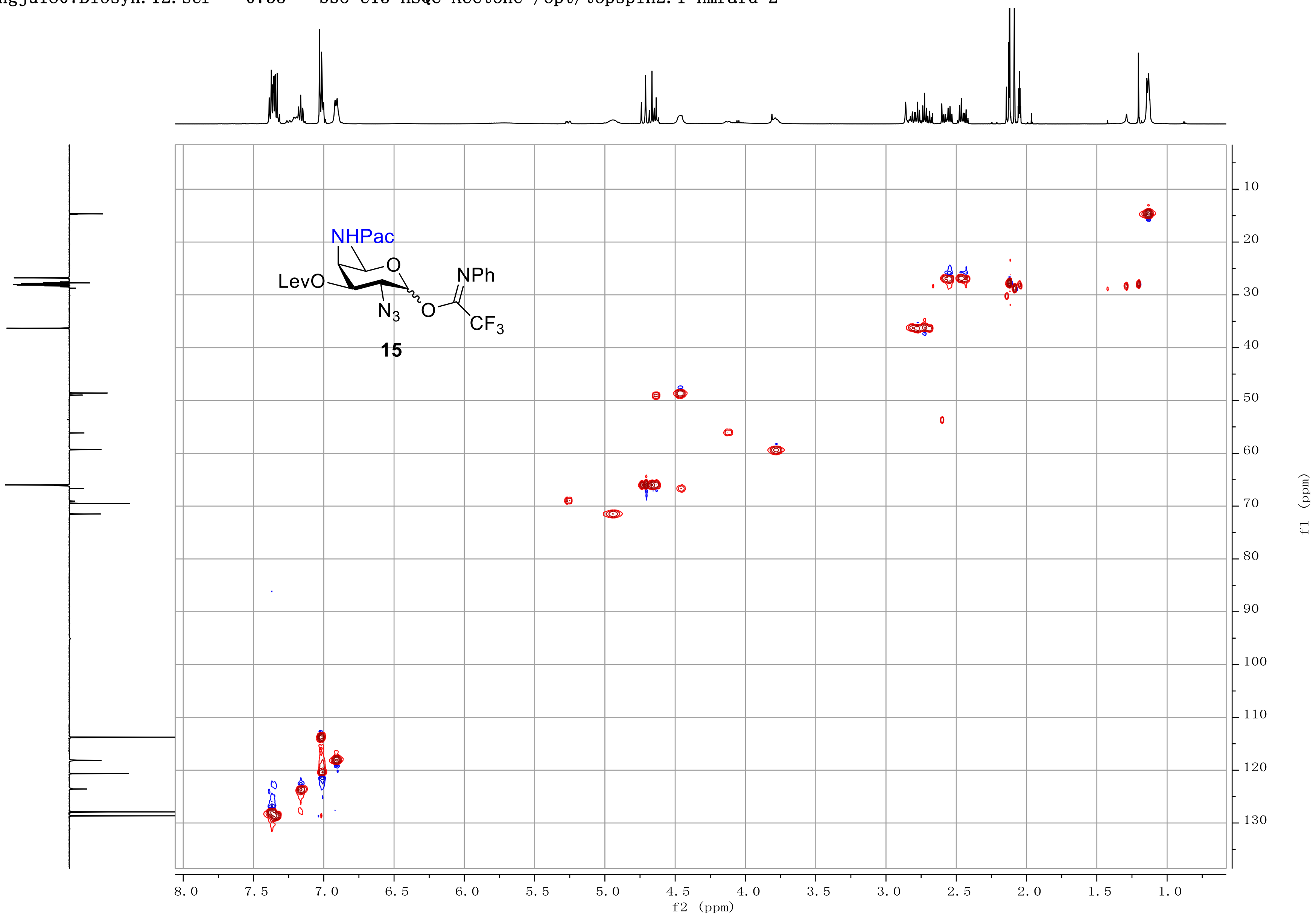

zhen2107biosyn.74.fid — wz781-A-S — bbo-h1 CDCl3 /opt/topspin2.1 nmrafd 9

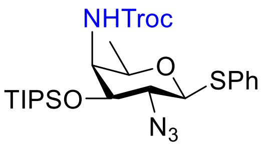

19a

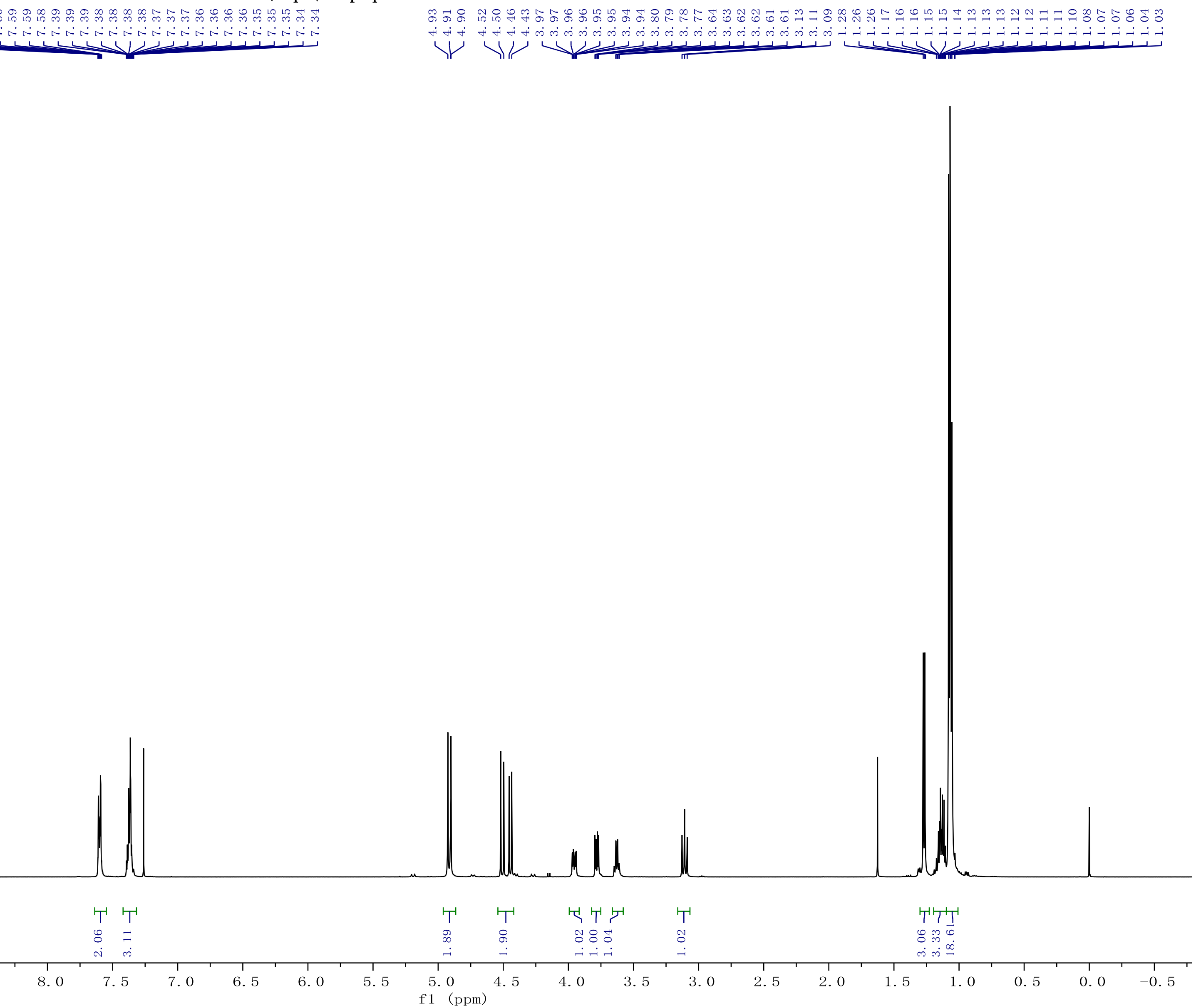

zhen2107biosyn.77.fid — wz781-A-S — bbo-c13-APT CDCl3 /opt/topspin2.1 nmrafd 9

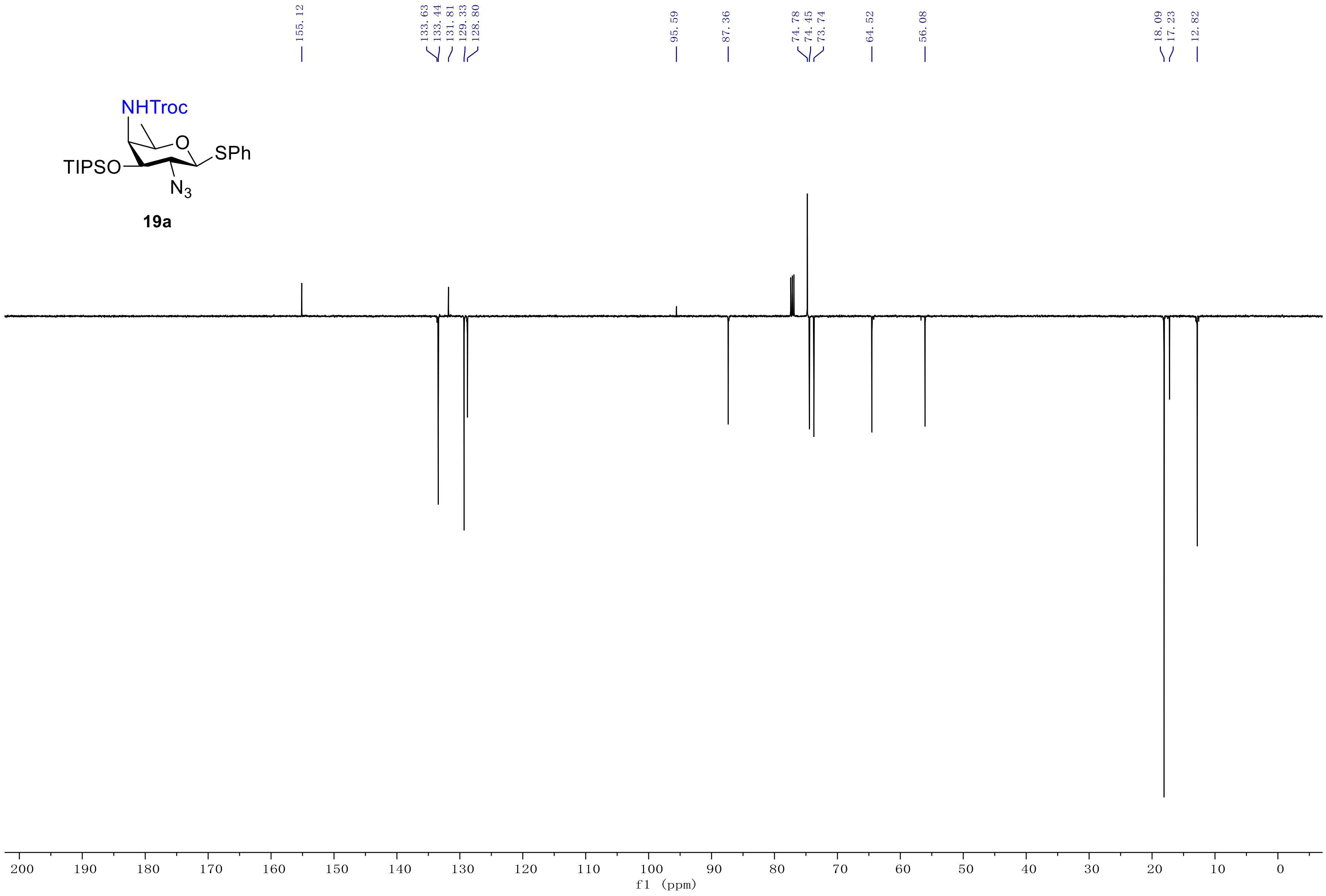

zhen2107biosyn.75.ser — wz781-A-S — bbo-h1-cosy CDCl3 /opt/topspin2.1 nmrafd 9

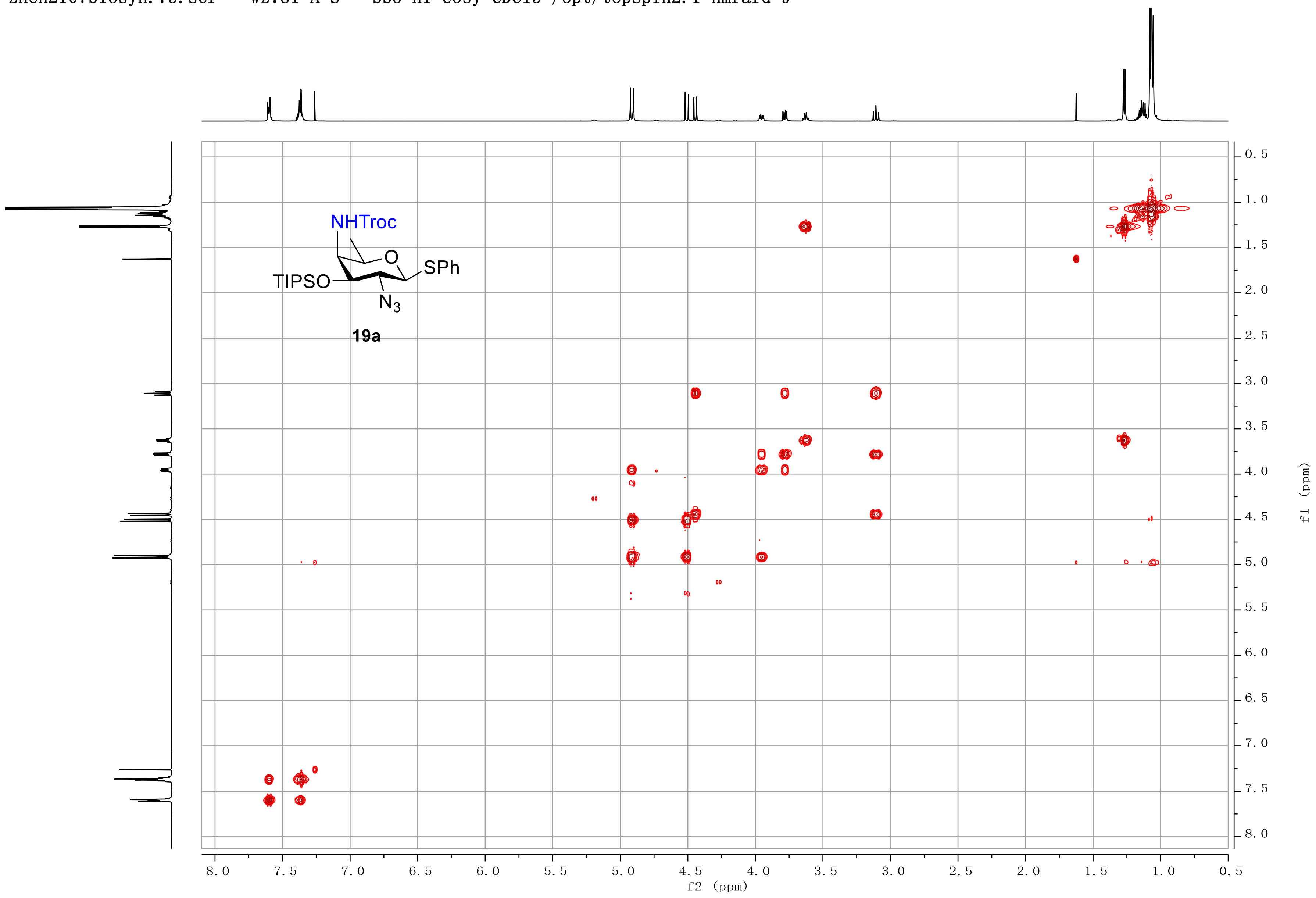

zhen2107biosyn.76.ser — wz781-A-S — bbo-c13-HSQC CDCl3 /opt/topspin2.1 nmrafd 9

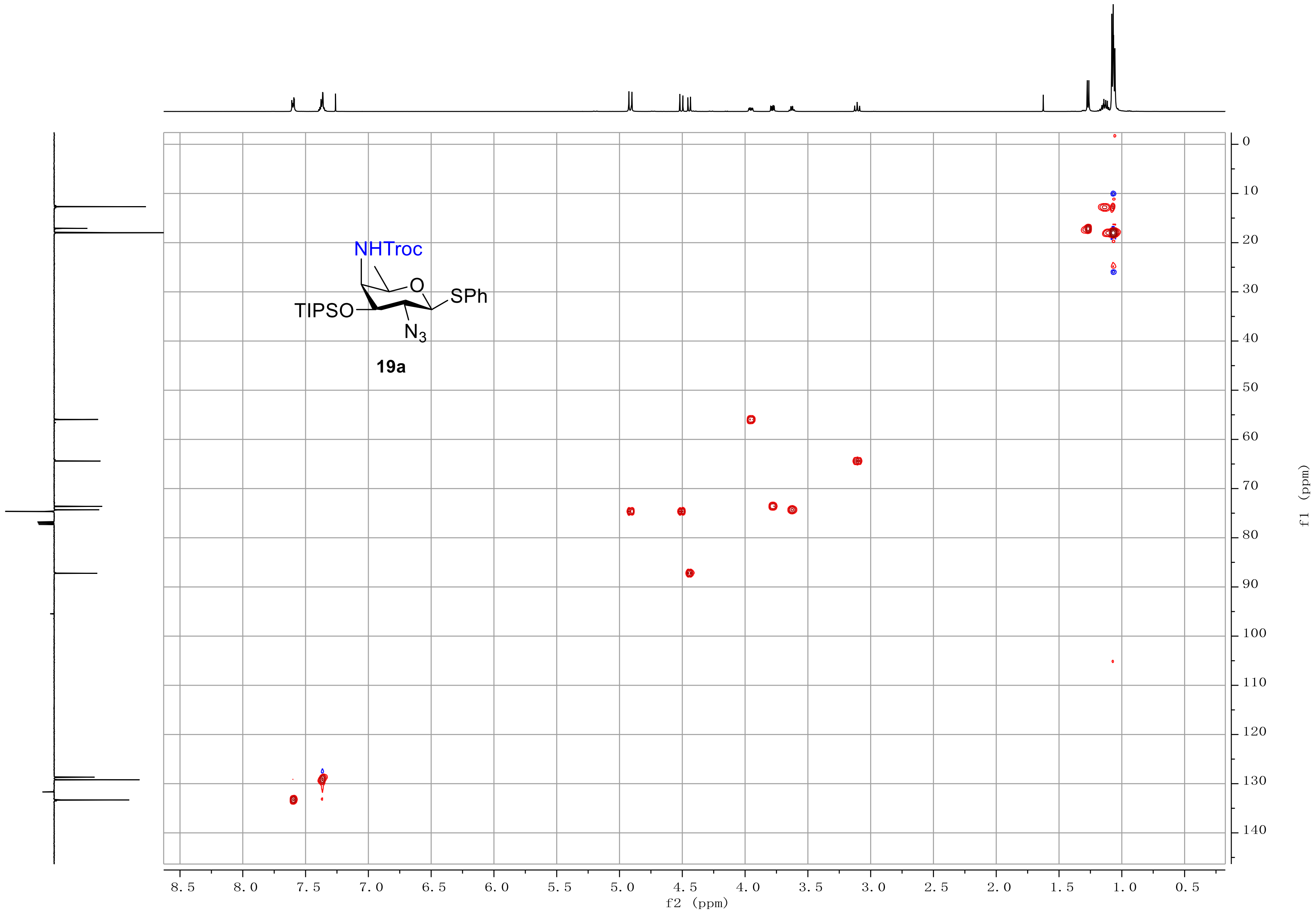

zhen2107biosyn.78.ser — wz781-A-S — bbo-c13-HMBC CDCl3 /opt/topspin2.1 nmrafd 9

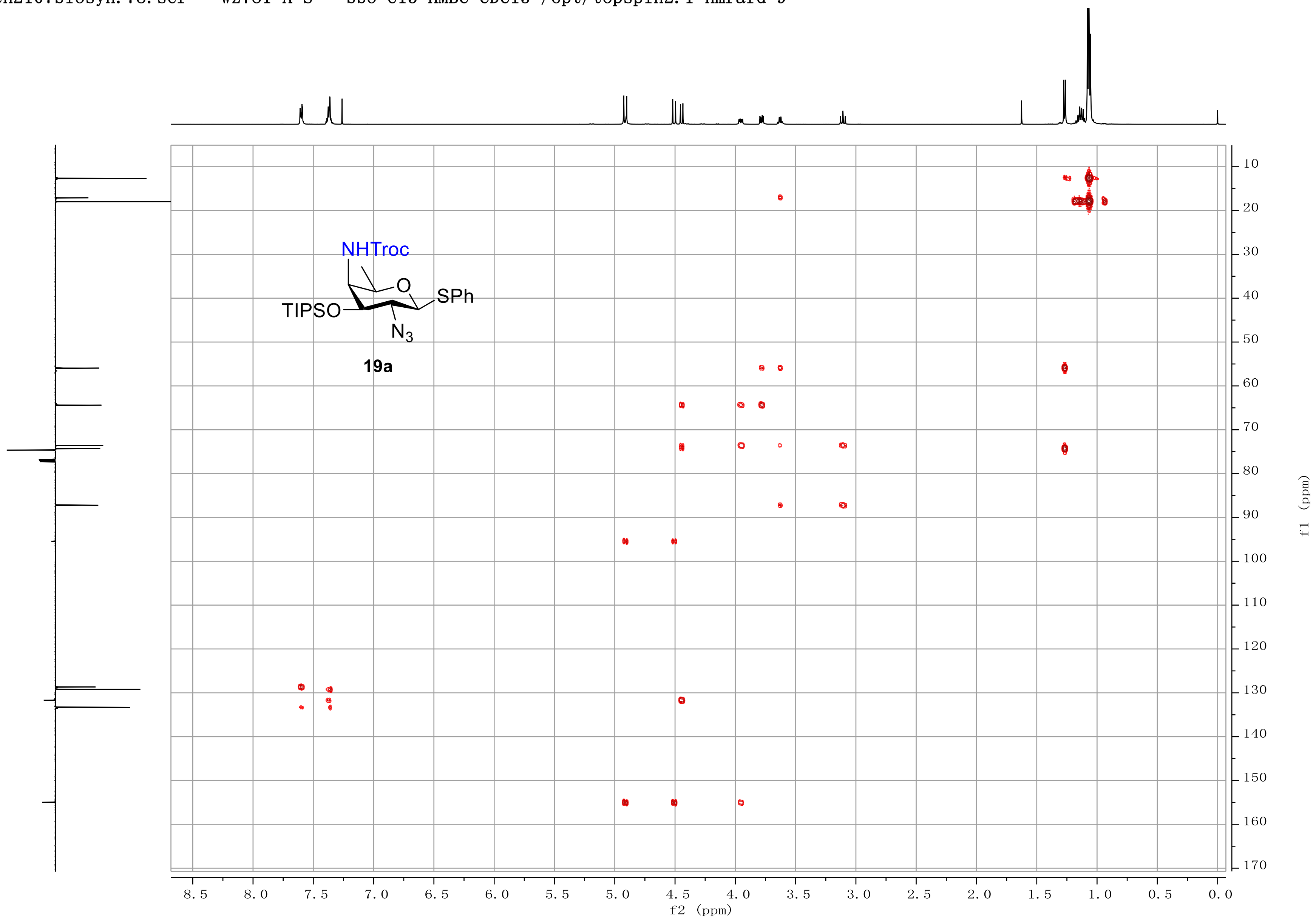

2108zhen.28.fid — wz782-D — h1 CDCl3 /opt/DATA nmrafd 7

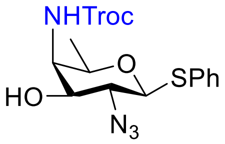

19

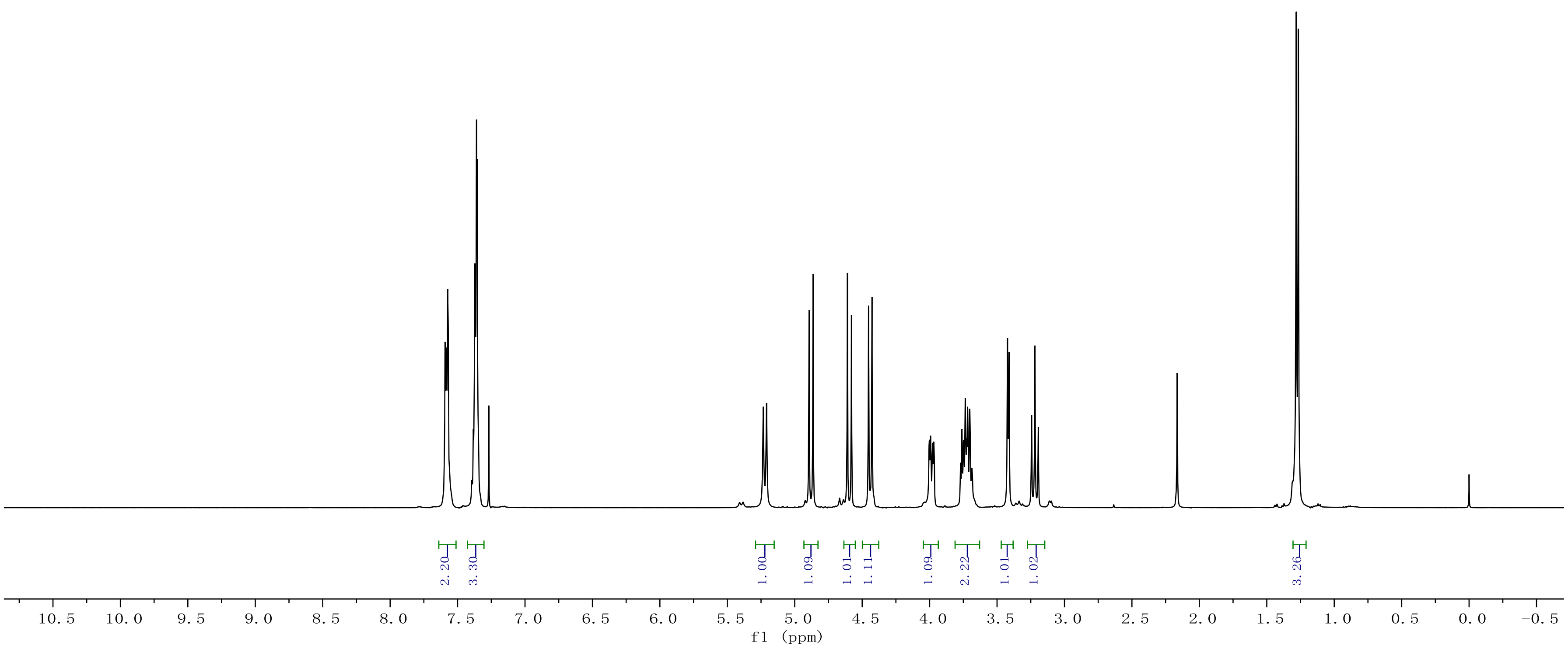

2108zhen.29.fid — wz782-D — C13APT CDCl3 /opt/DATA nmrafd 7

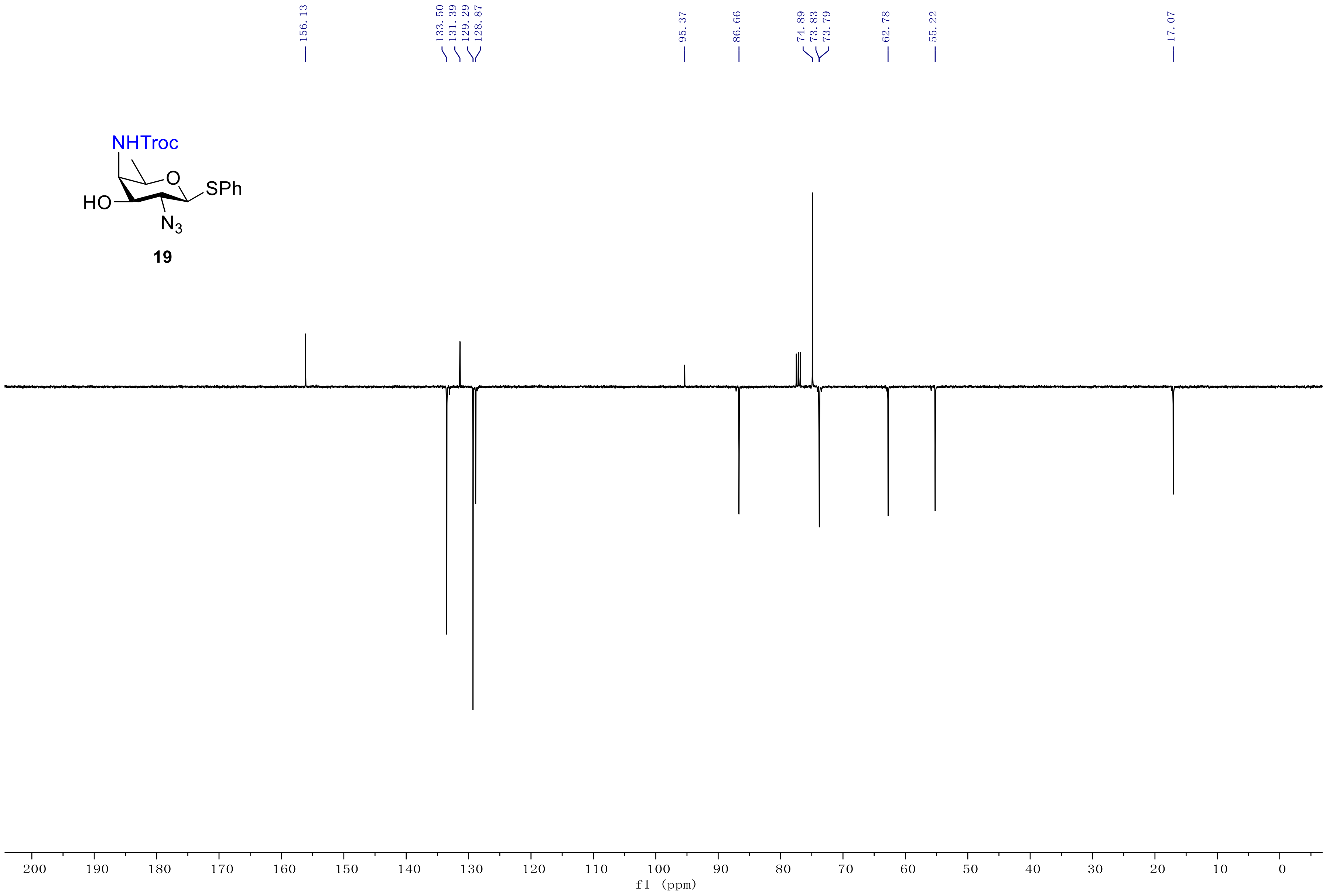

2108zhen.30.ser — wz782-D — h1COSY CDCl3 /opt/DATA nmrafd 7

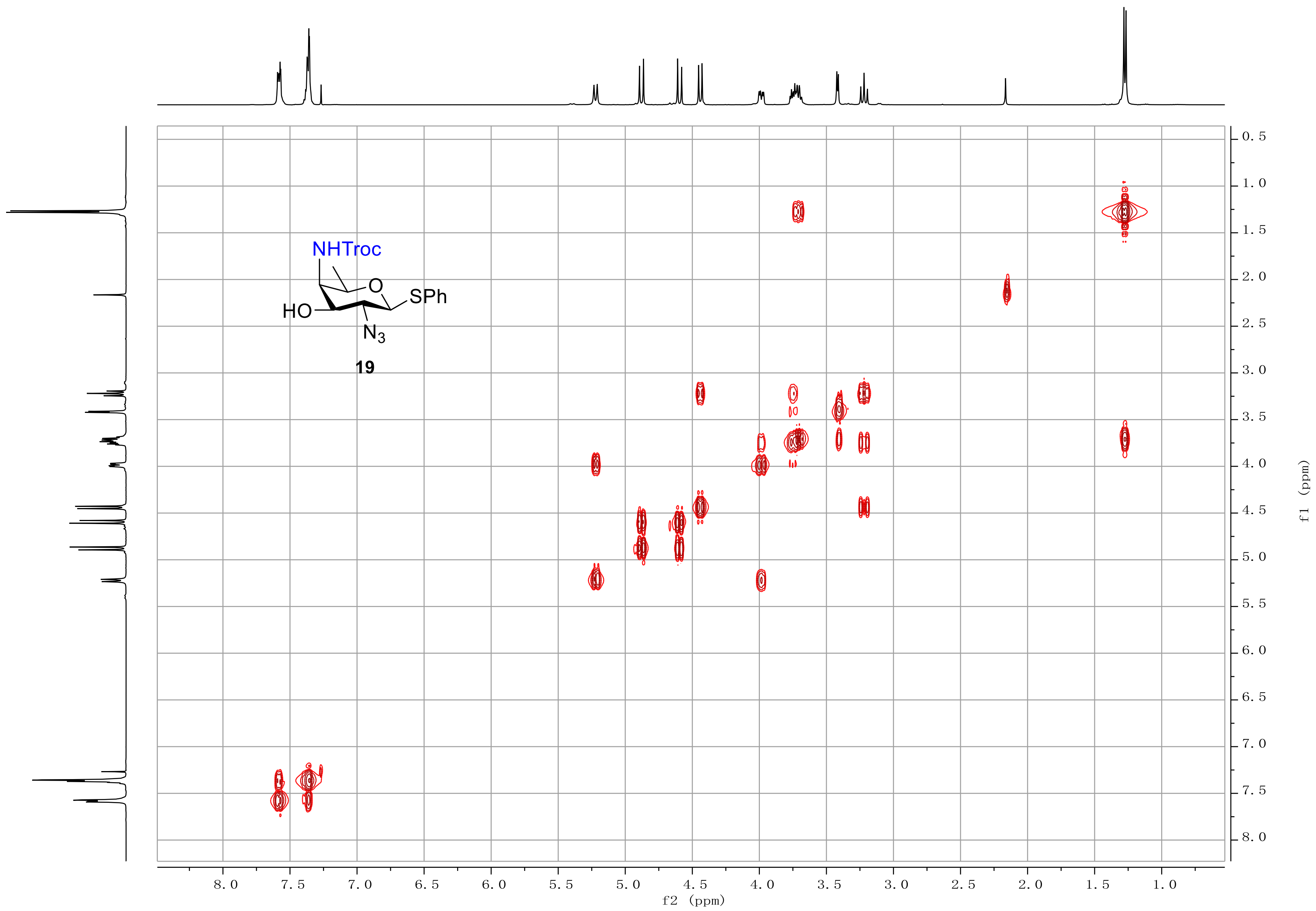

2108zhen.31.ser — wz782-D — c13HSQC CDCl3 /opt/DATA nmrafd 7

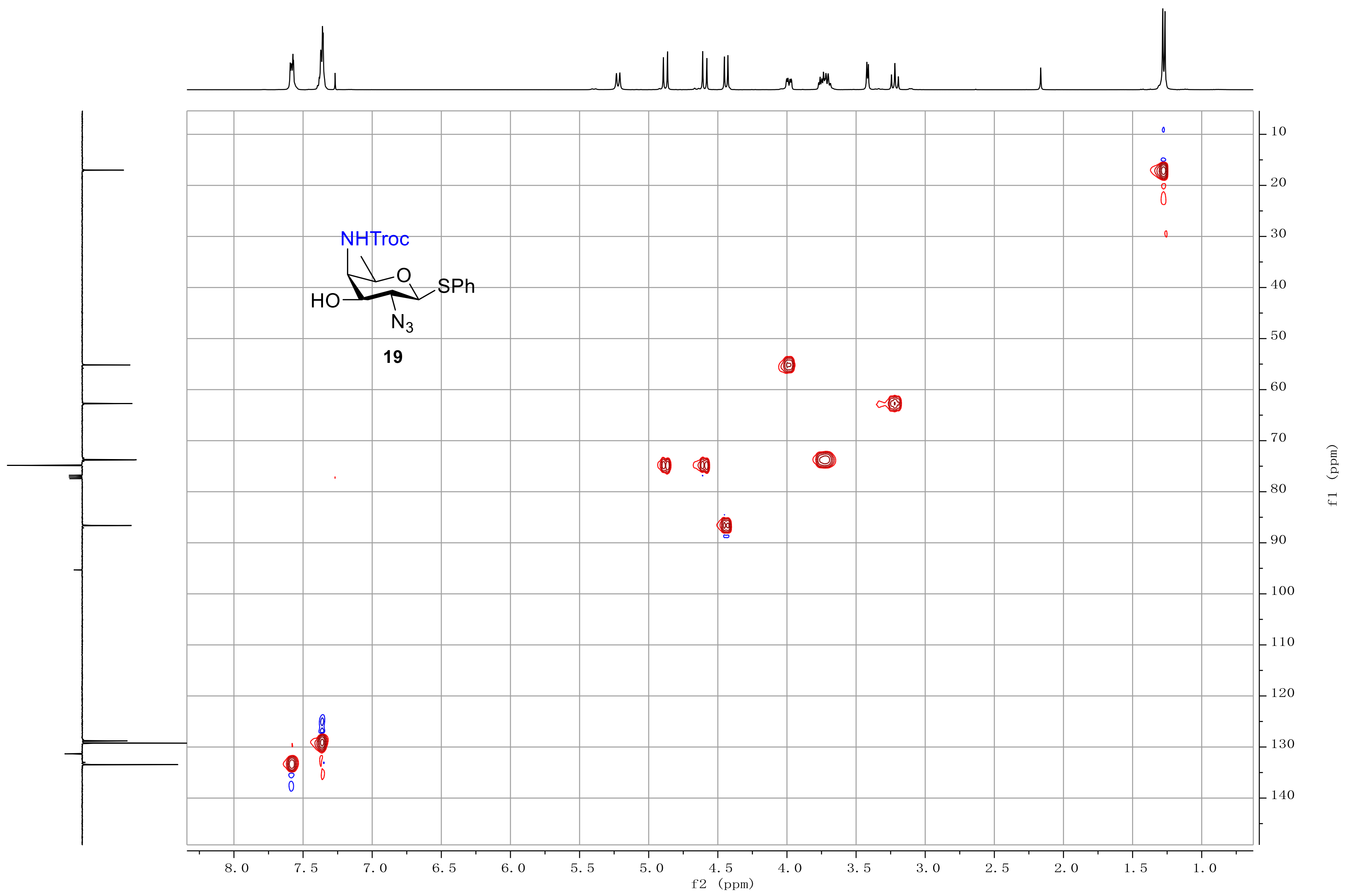

2108zhen.32.ser — wz782-D — c13HMBC CDCl3 /opt/DATA nmrafd 7

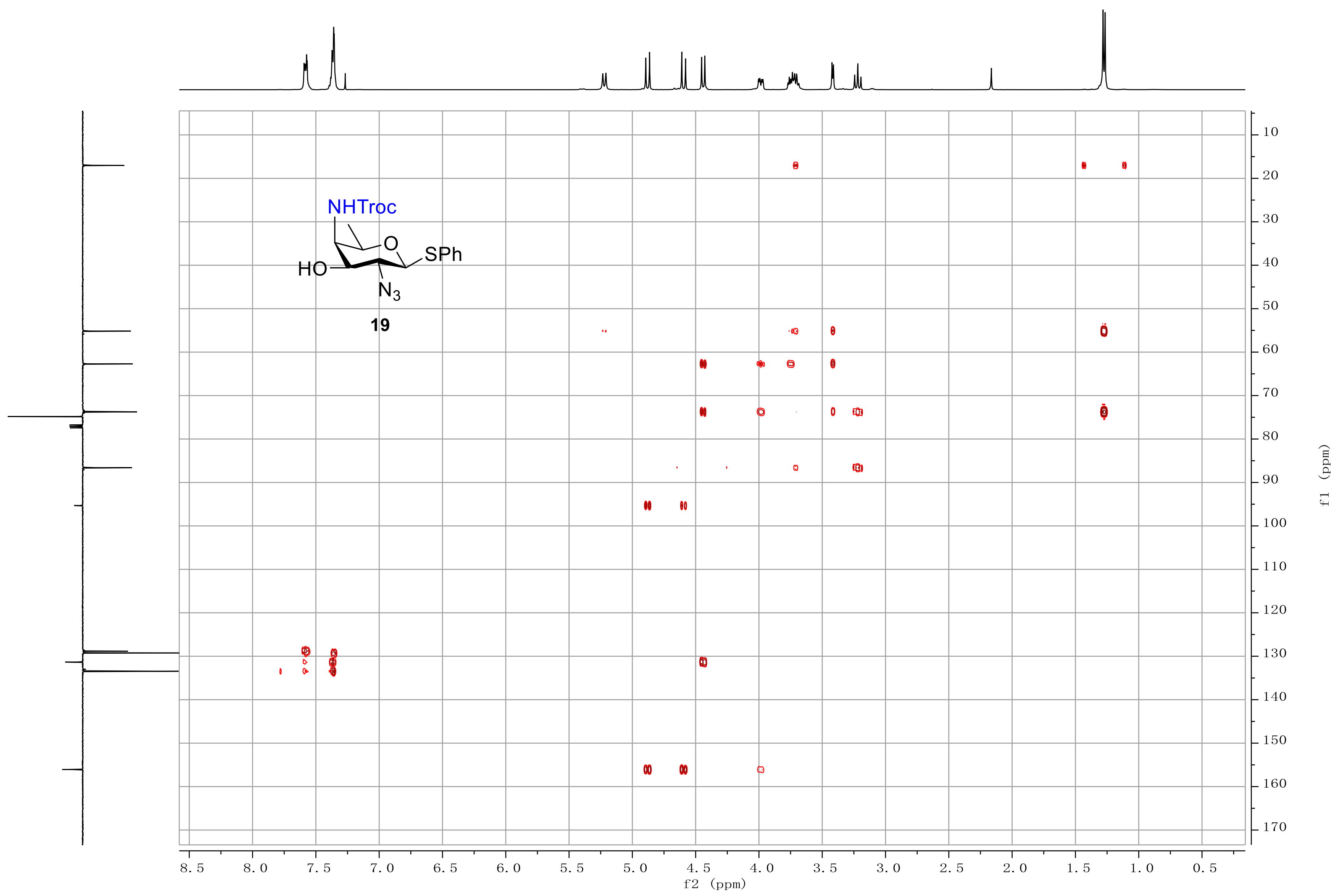

zhen2107biosyn.46.fid — wz778-1-s — bbo-h1 CDCl3 /opt/topspin2.1 nmrafd 7

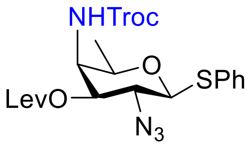

21

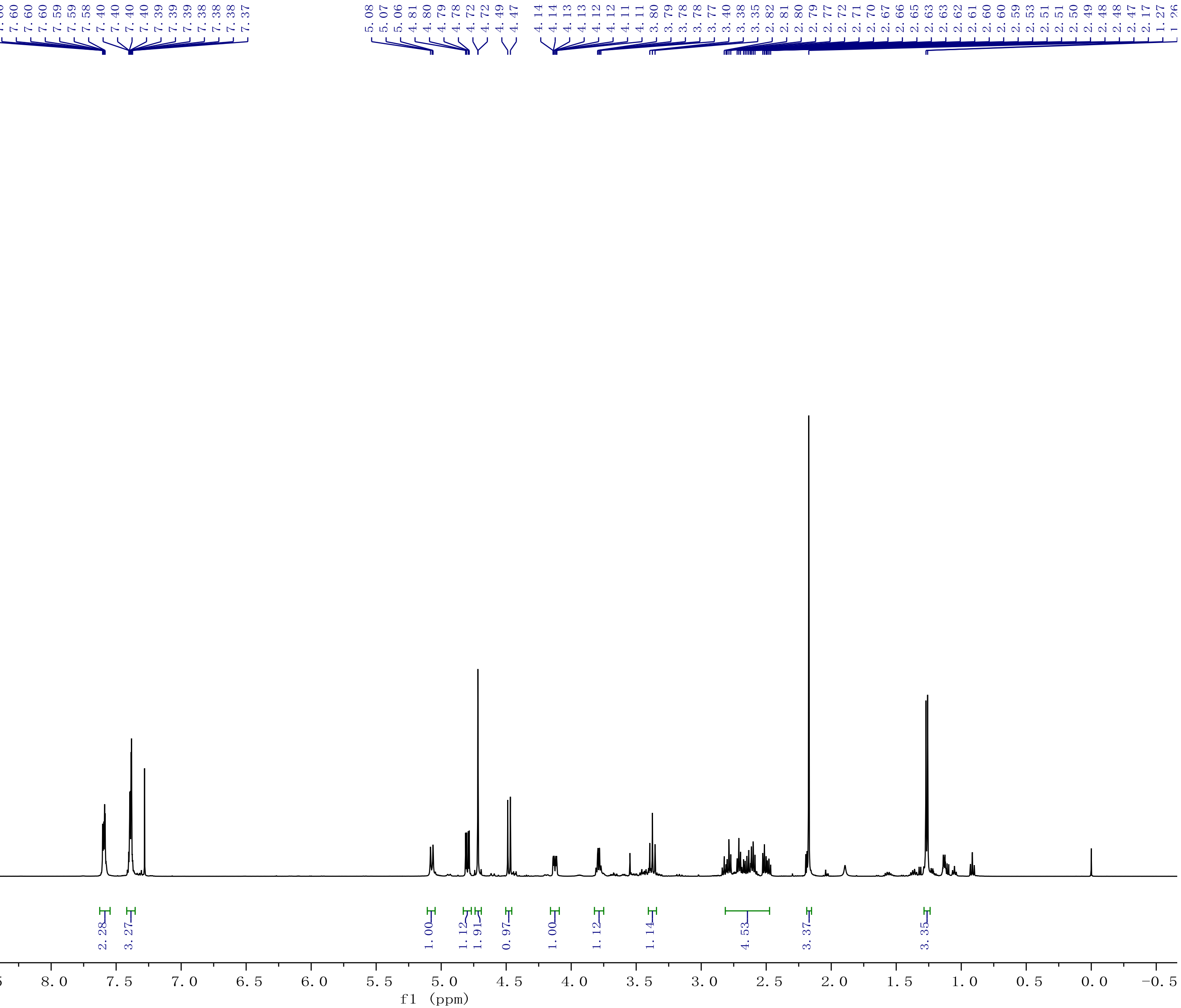

zhen2107biosyn.49.fid — wz778-1-s — bbo-c13-APT CDCl3 /opt/topspin2.1 nmrafd 7

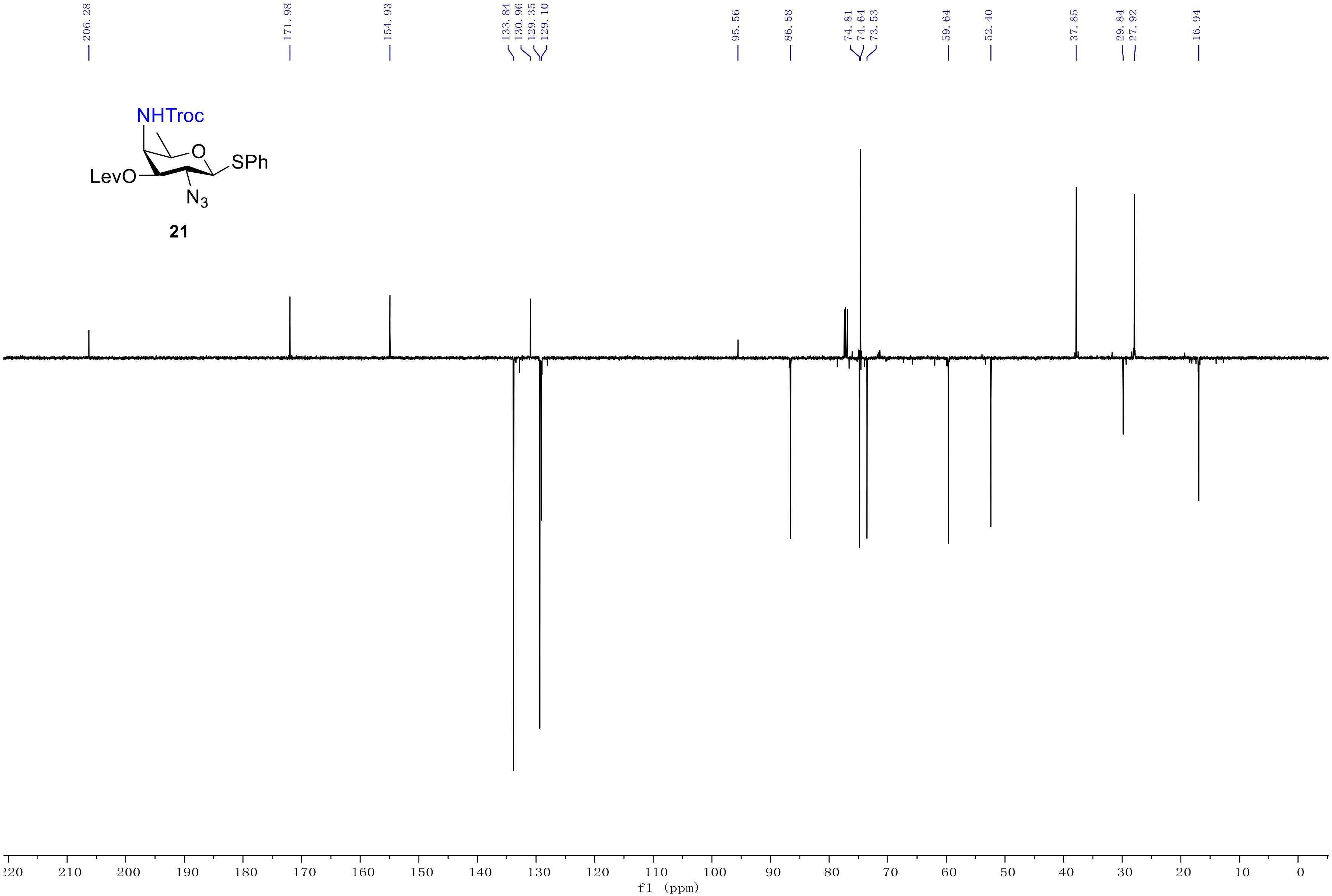

zhen2107biosyn.47.ser — wz778-1-s — bbo-h1-cosy CDCl3 /opt/topspin2.1 nmrafd 7

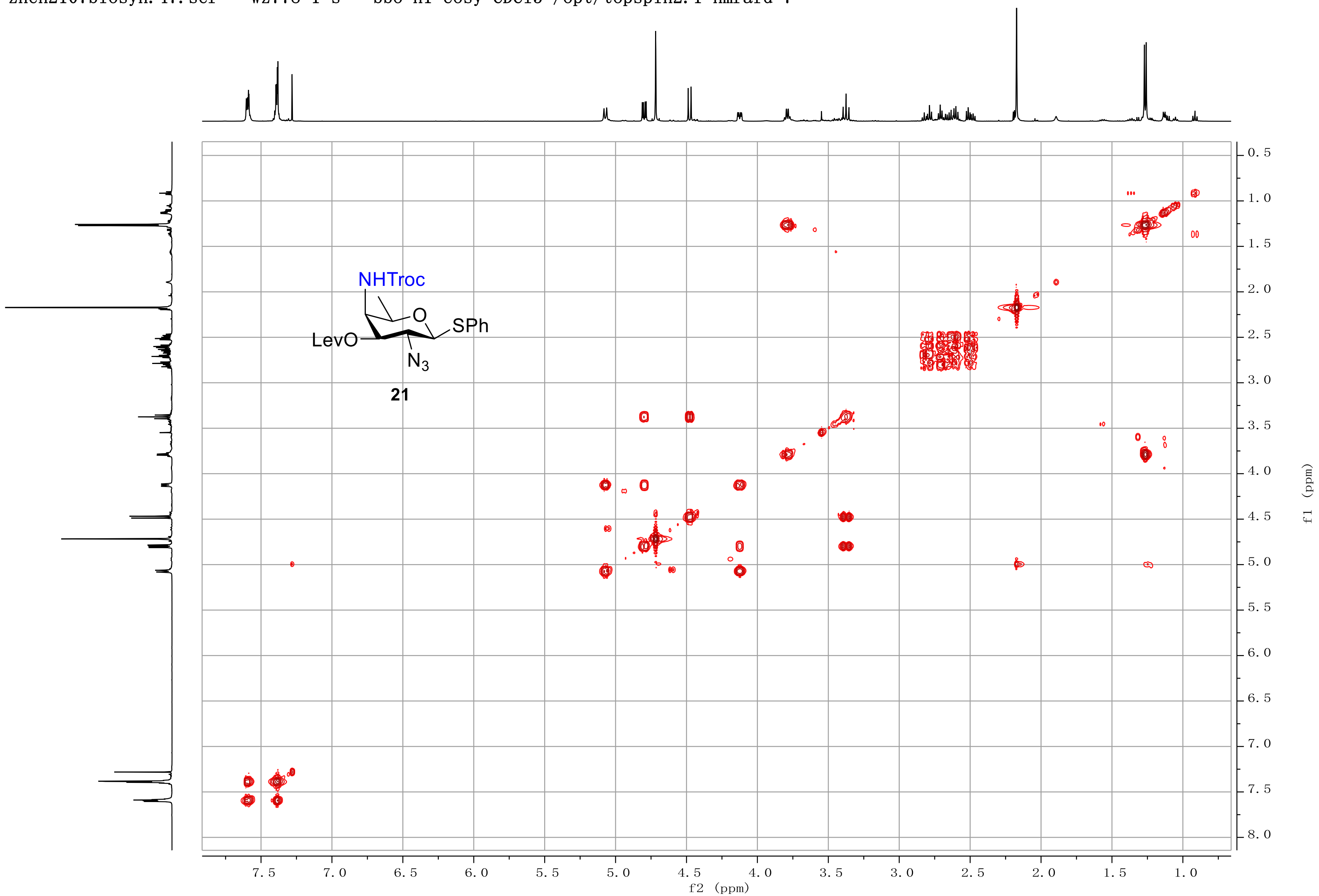

zhen2107biosyn.48.ser — wz778-1-s — bbo-c13-HSQC CDCl3 /opt/topspin2.1 nmrafd 7

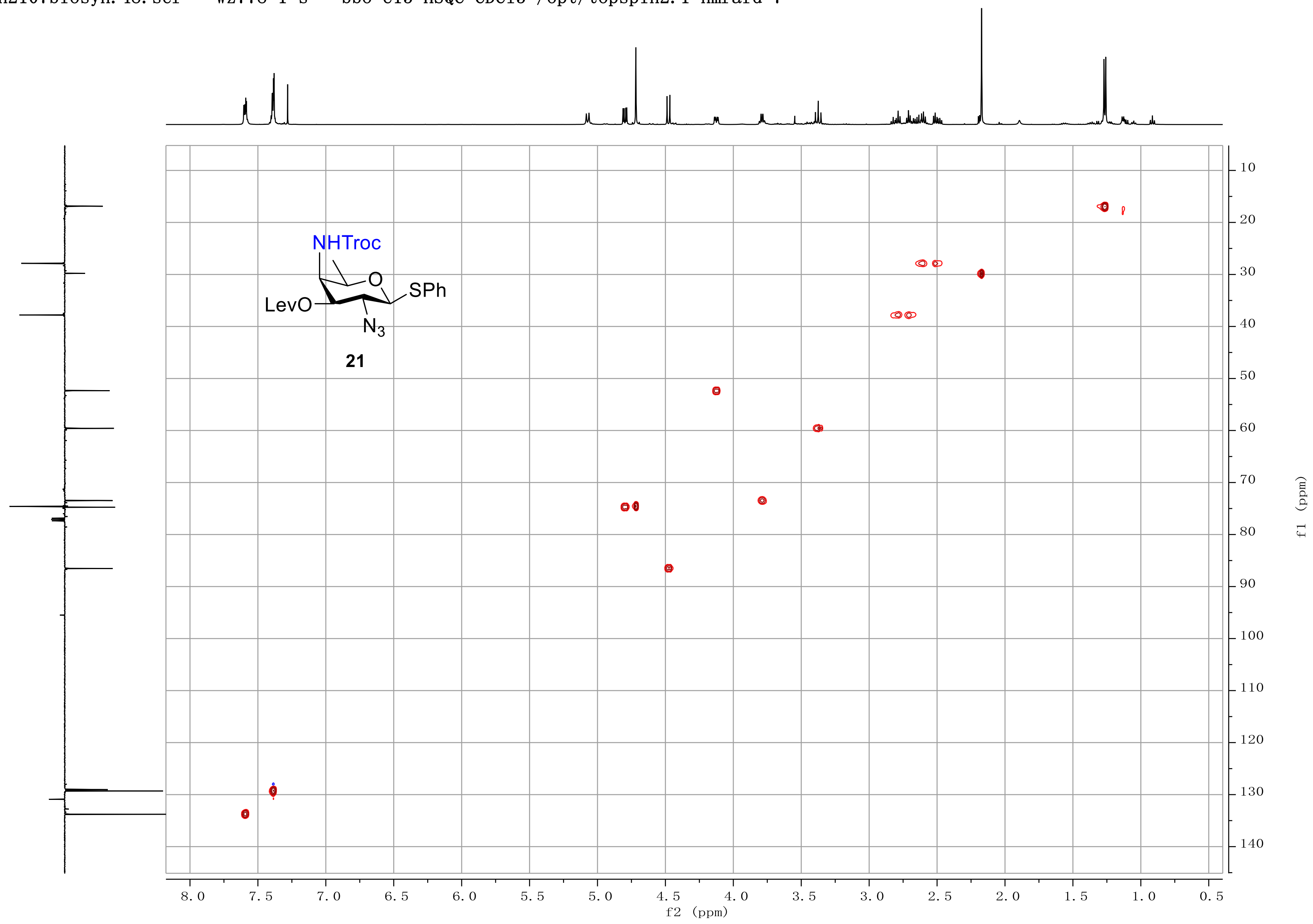

zhen2107biosyn.50.ser — wz778-1-s — bbo-c13-HMBC CDCl3 /opt/topspin2.1 nmrafd 7

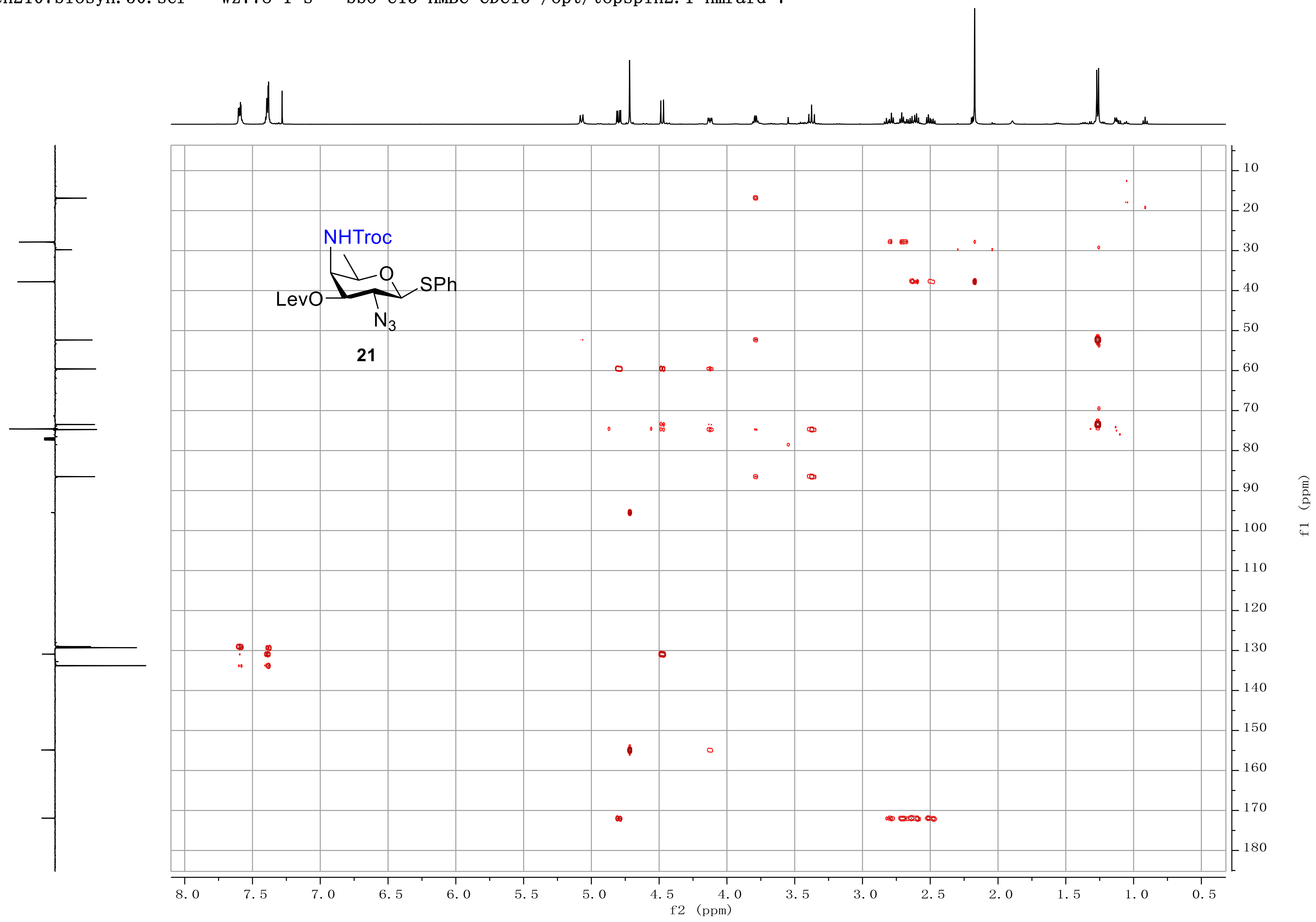

zhen2108biosyn.6.fid — wz779-B-s — bbo-h1 CDCl3 /opt/topspin2.1 nmrafd 7

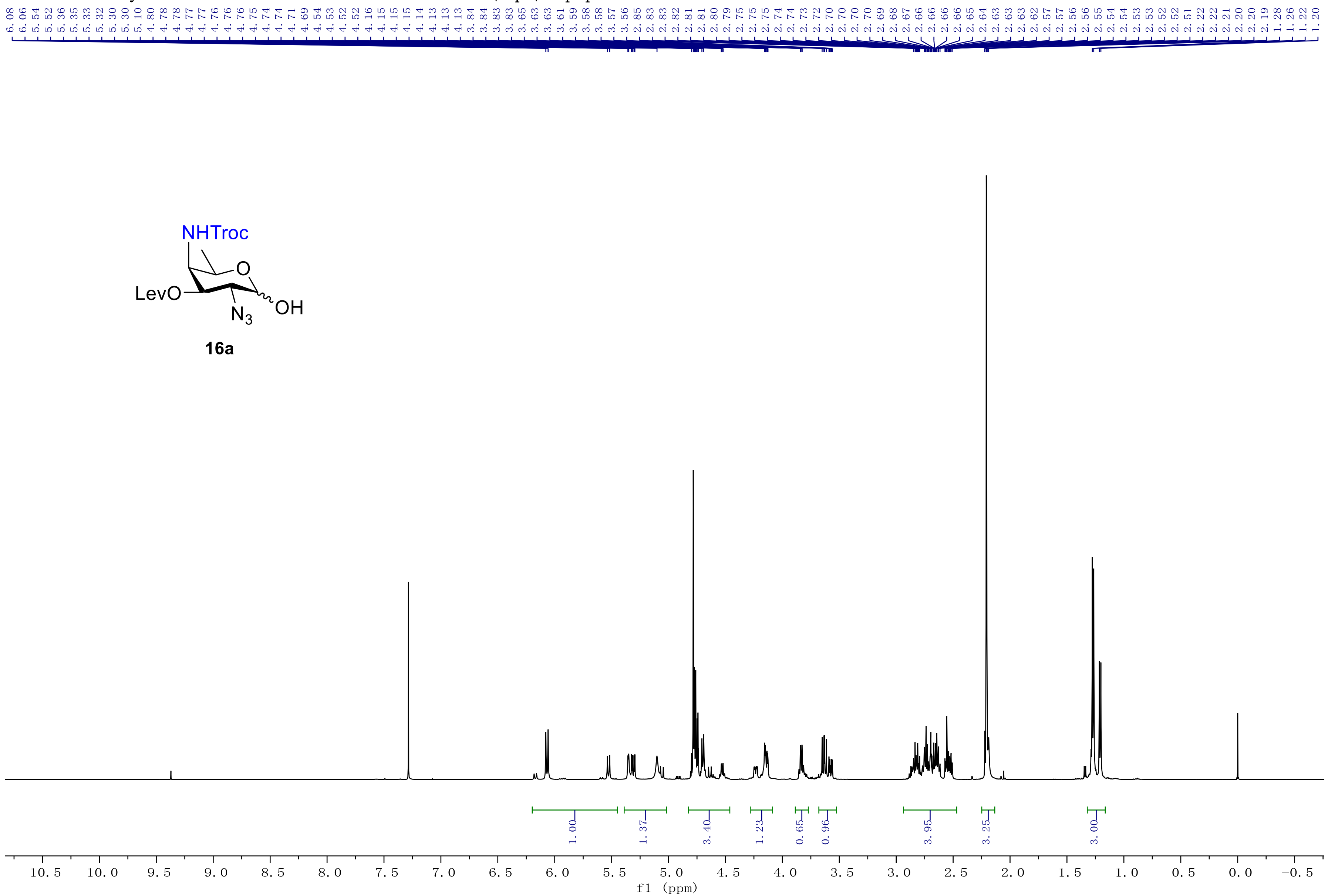

zhen2108biosyn.9.fid — wz779-B-s — bbo-c13-APT CDCl3 /opt/topspin2.1 nmrafd 7

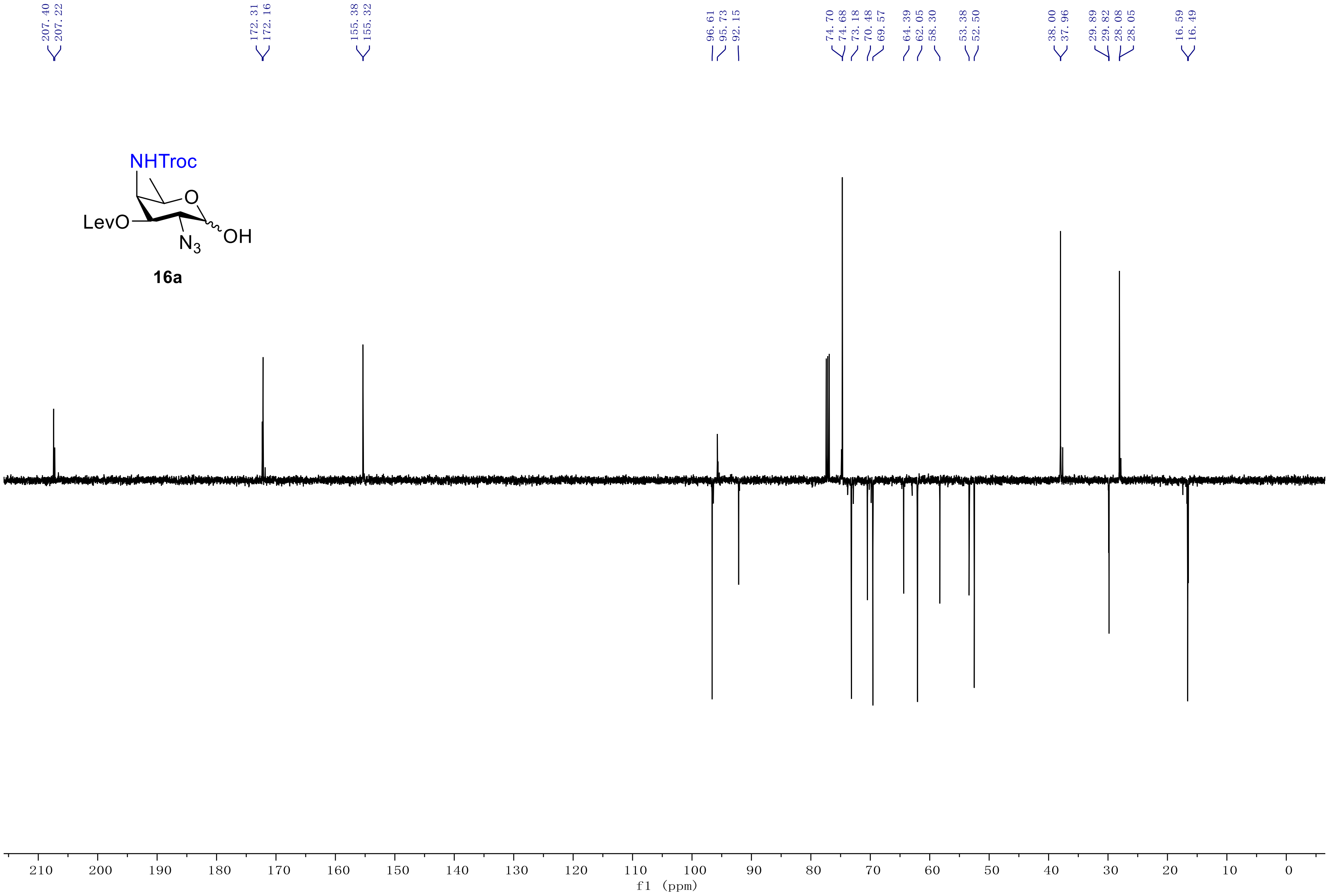

zhen2108biosyn.7.ser — wz779-B-s — bbo-h1-cosy CDCl3 /opt/topspin2.1 nmrafd 7

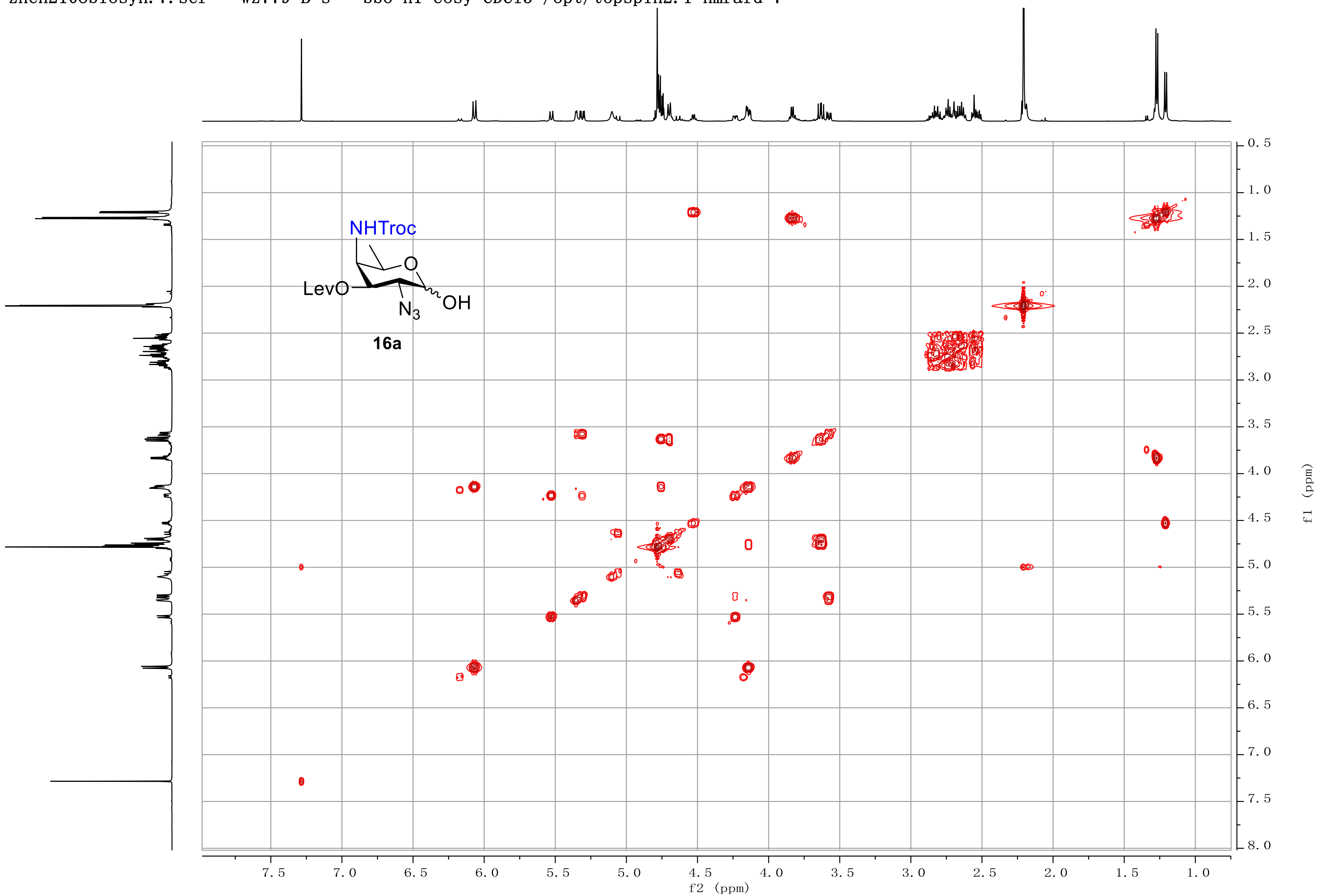

zhen2108biosyn.8.ser — wz779-B-s — bbo-c13-HSQC CDCl3 /opt/topspin2.1 nmrafd 7

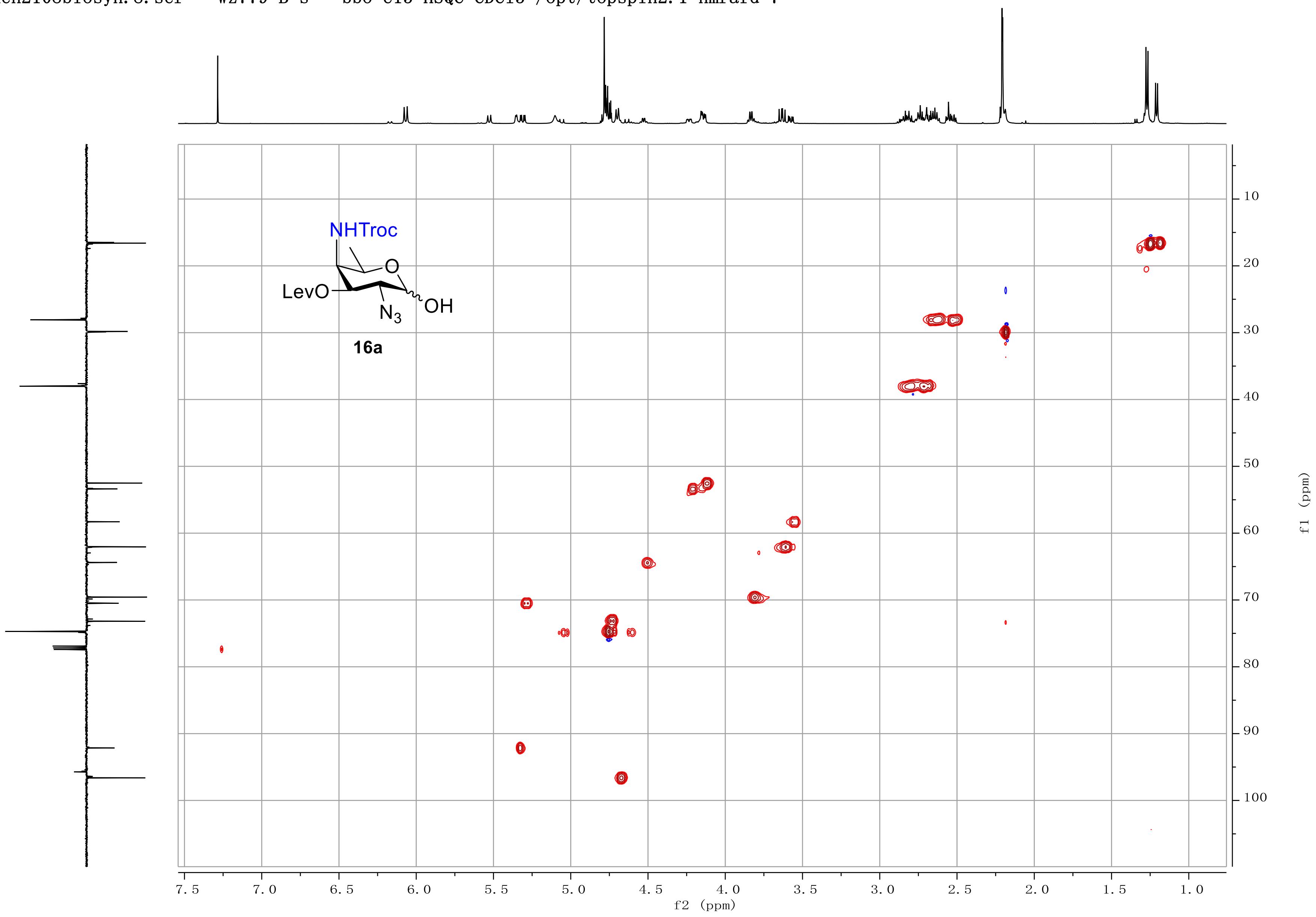

zhen2108biosyn.10.ser — wz779-B-s — bbo-c13-HMBC CDCl3 /opt/topspin2.1 nmrafd 7

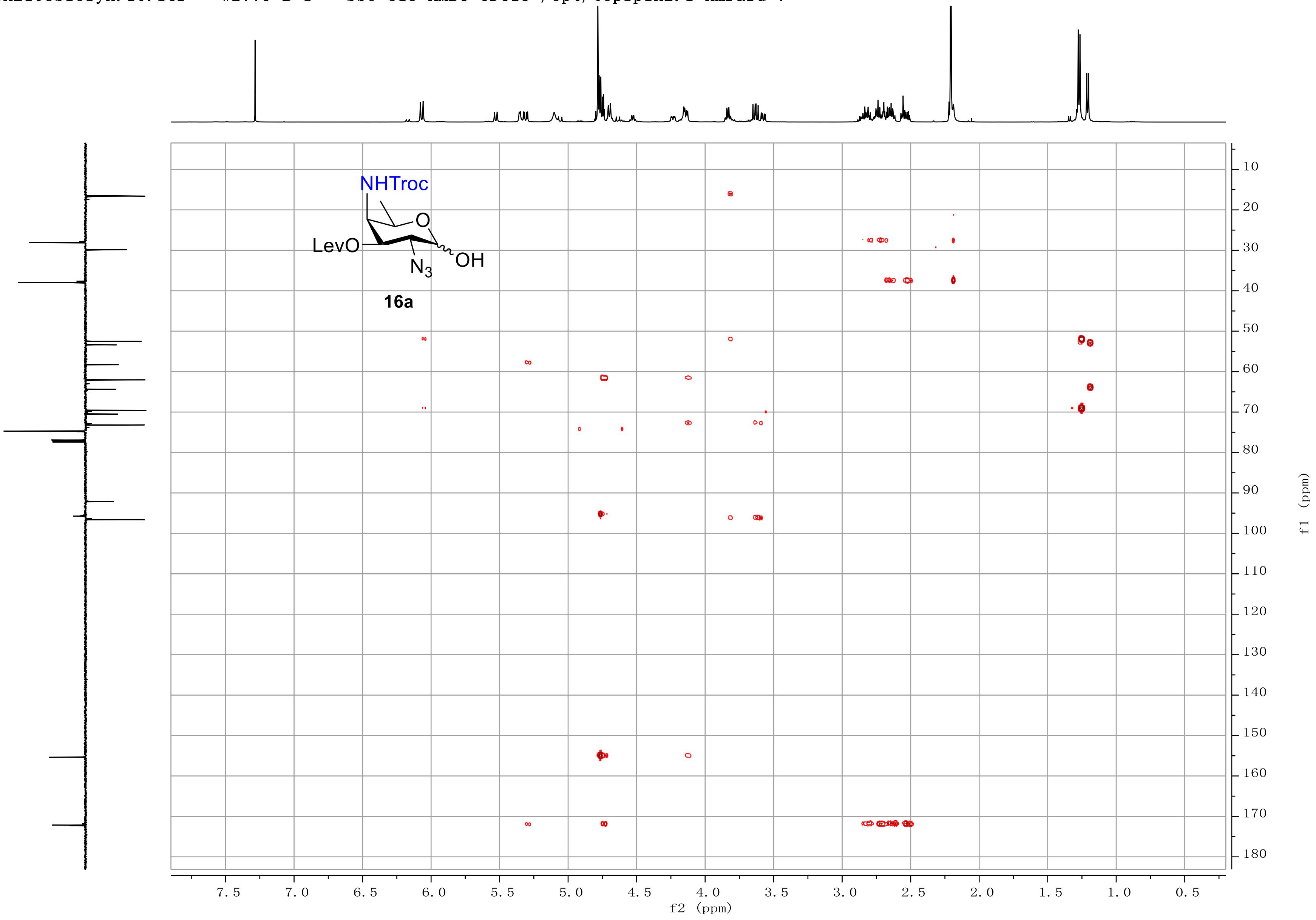

zhen2108biosyn.11.fid — wz780-A-s — bbo-h1 CDCl3 /opt/topspin2.1 nmrafd 7

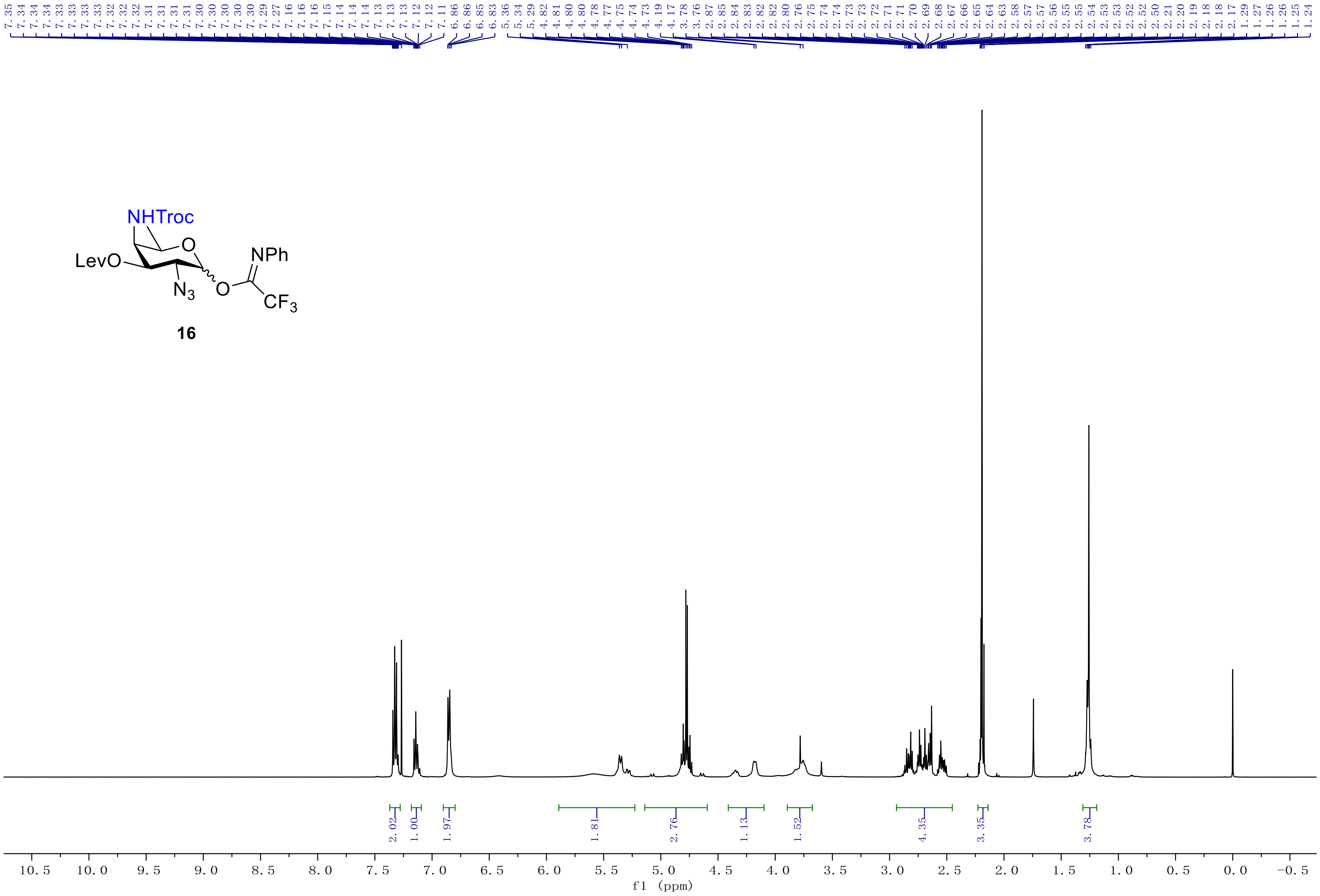

16

zhen2108biosyn.14.fid — wz780-A-s — bbo-c13-APT CDCl3 /opt/topspin2.1 nmrafd 7

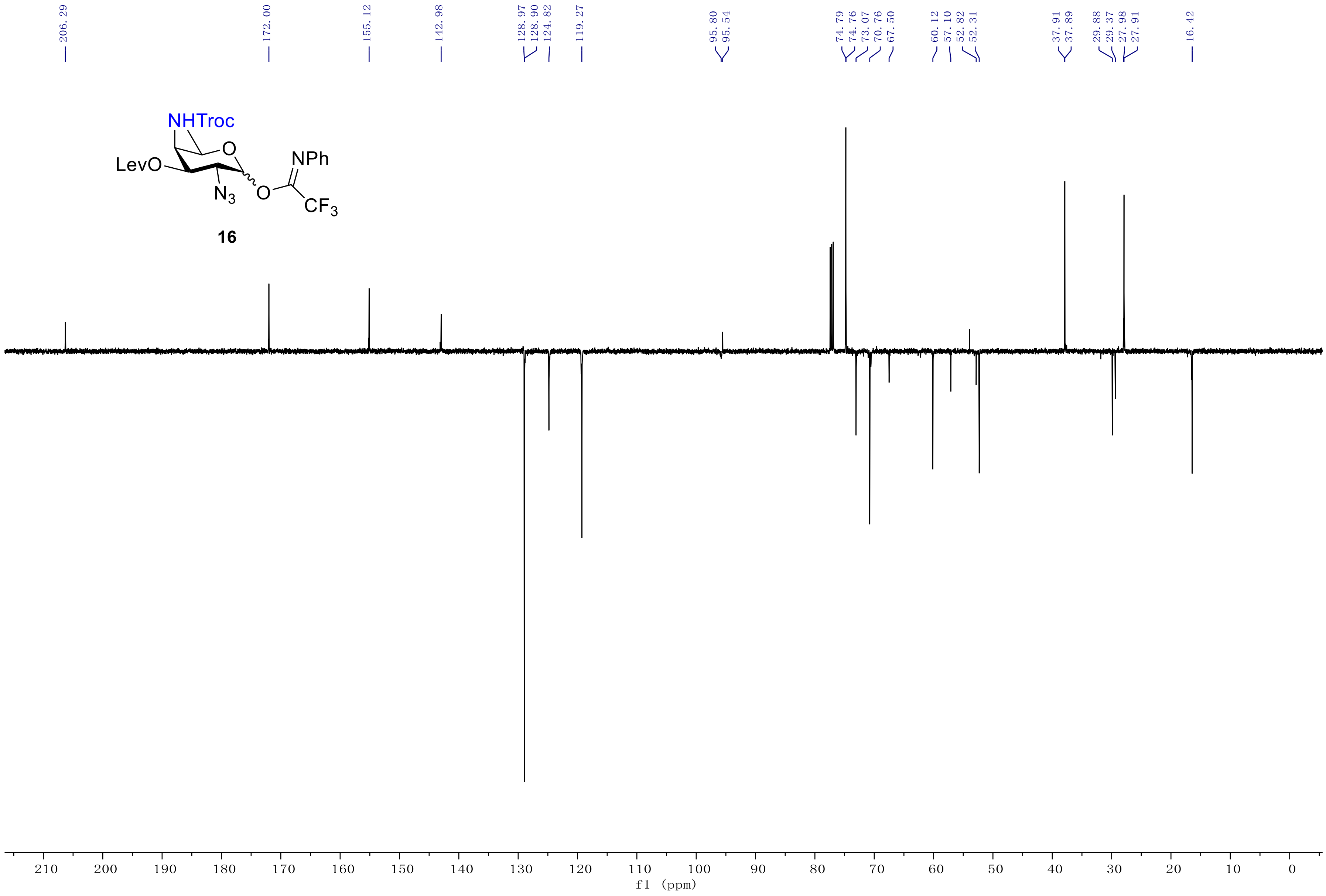

zhen2108biosyn.12.ser — wz780-A-s — bbo-h1-cosy CDCl3 /opt/topspin2.1 nmrafd 7

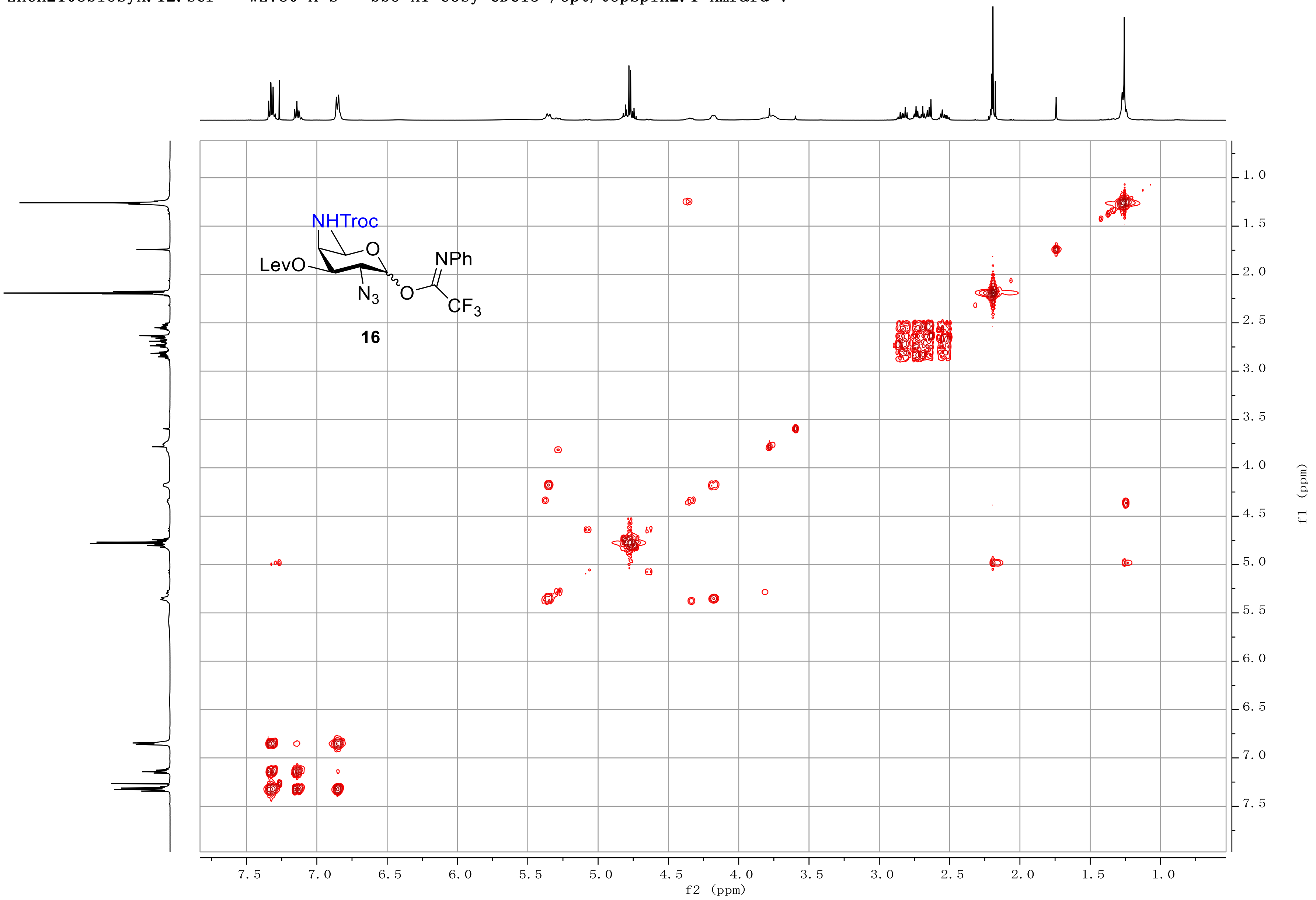

zhen2108biosyn.13.ser — wz780-A-s — bbo-c13-HSQC CDCl3 /opt/topspin2.1 nmrafd 7

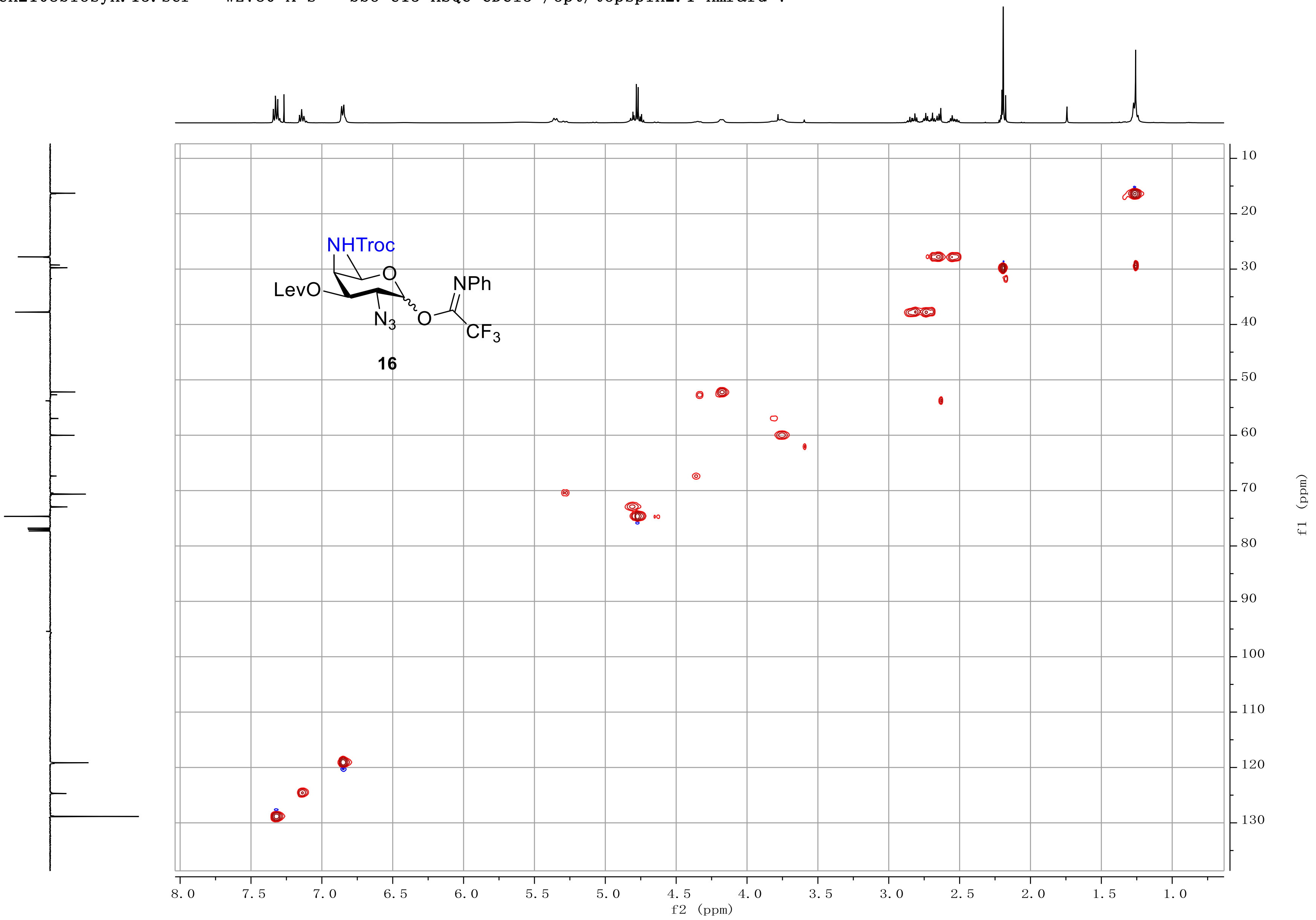

zhen2108biosyn.15.ser — wz780-A-s — bbo-c13-HMBC CDCl3 /opt/topspin2.1 nmrafd 7

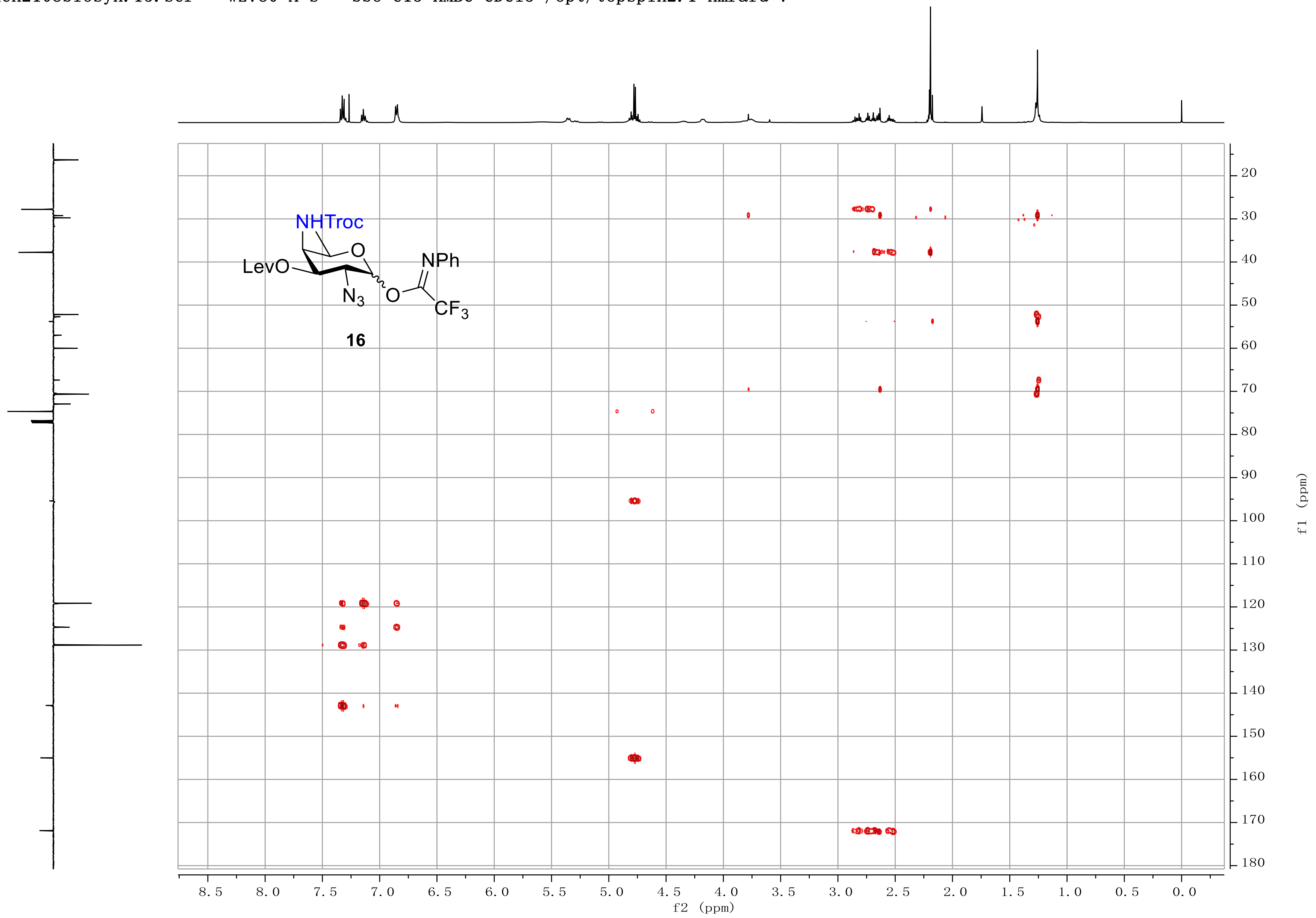

2009zhen.37.fid — wz729-1 — h1 CDCl3 /opt/DATA nmrafd 8

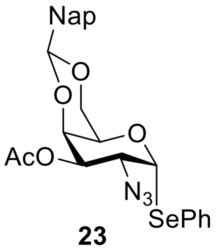

23

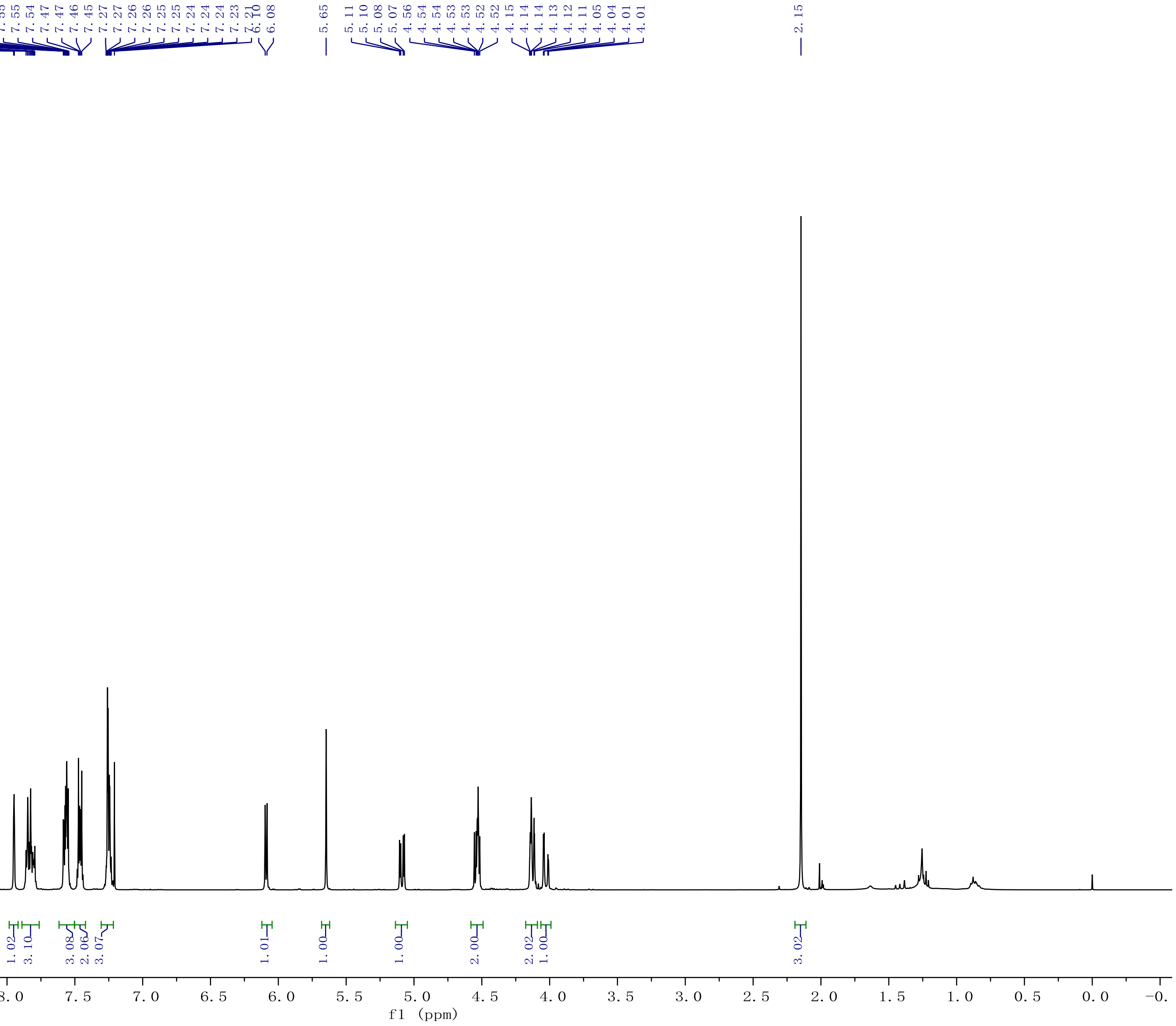

2009zhen.38.fid — wz729-1 — C13APT CDCl3 /opt/DATA nmrafd 8

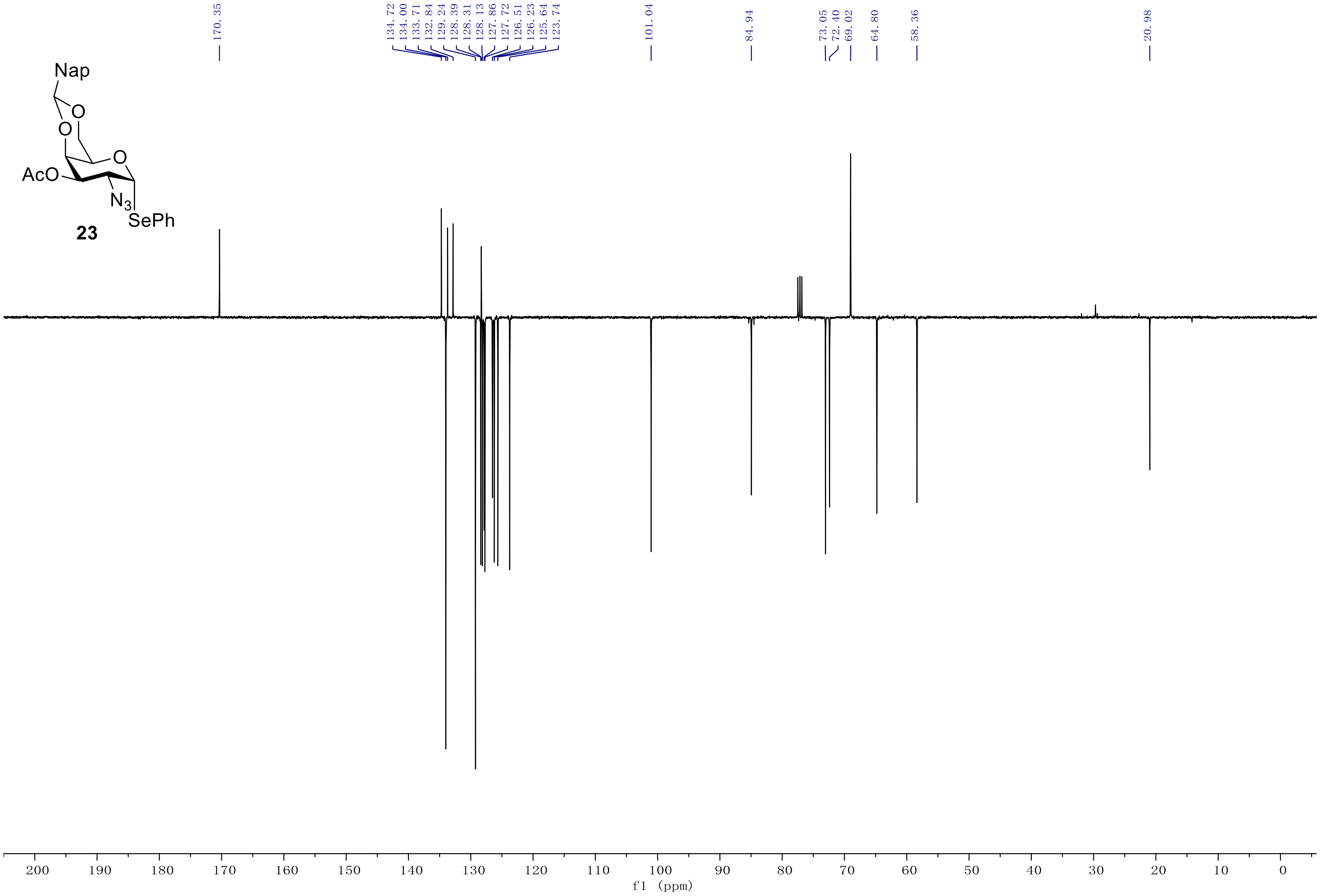

2009zhen.39.ser — wz729-1 — h1COSY CDCl3 /opt/DATA nmrafd 8

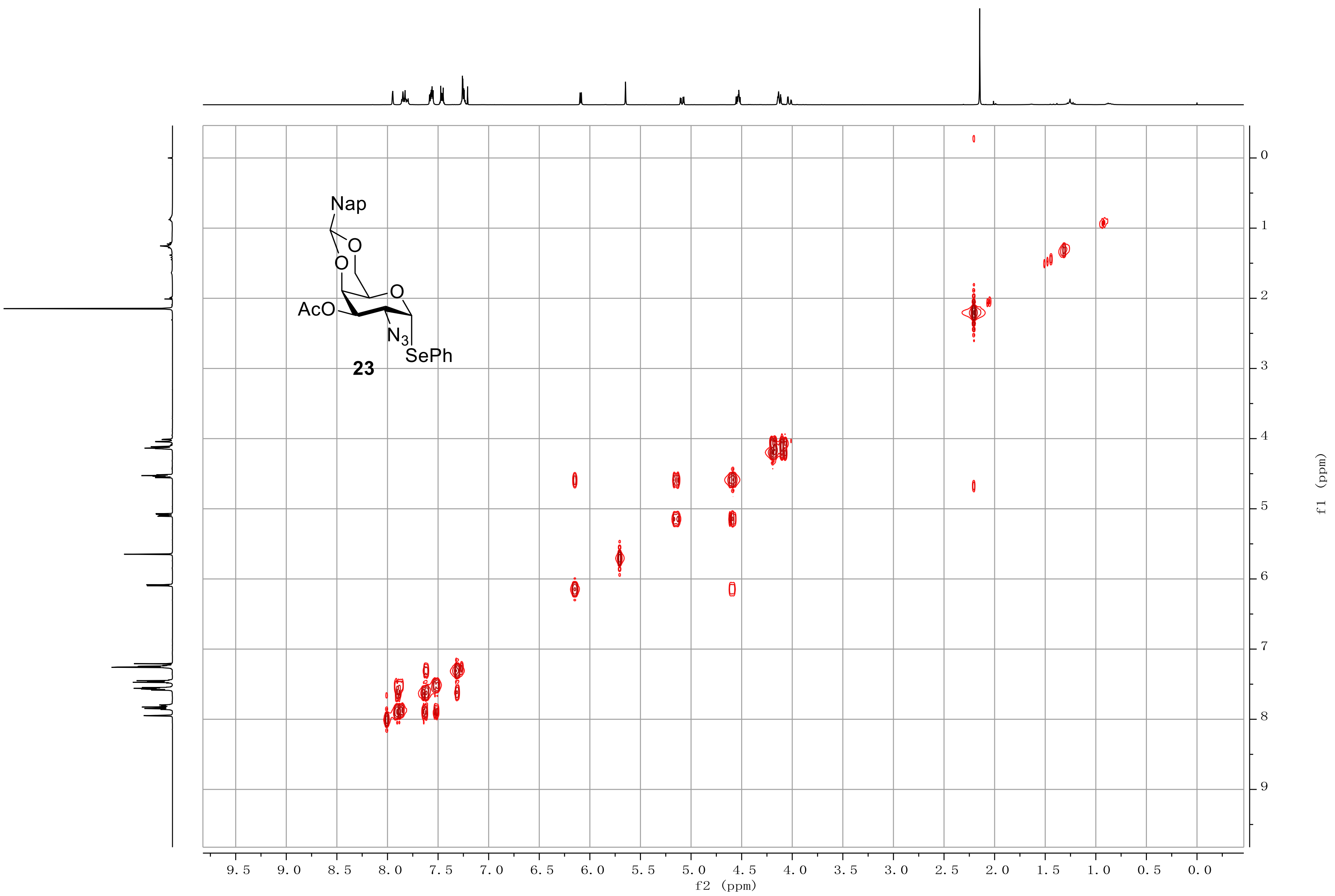

2009zhen.40.ser — wz729-1 — c13HSQC CDCl3 /opt/DATA nmrafd 8

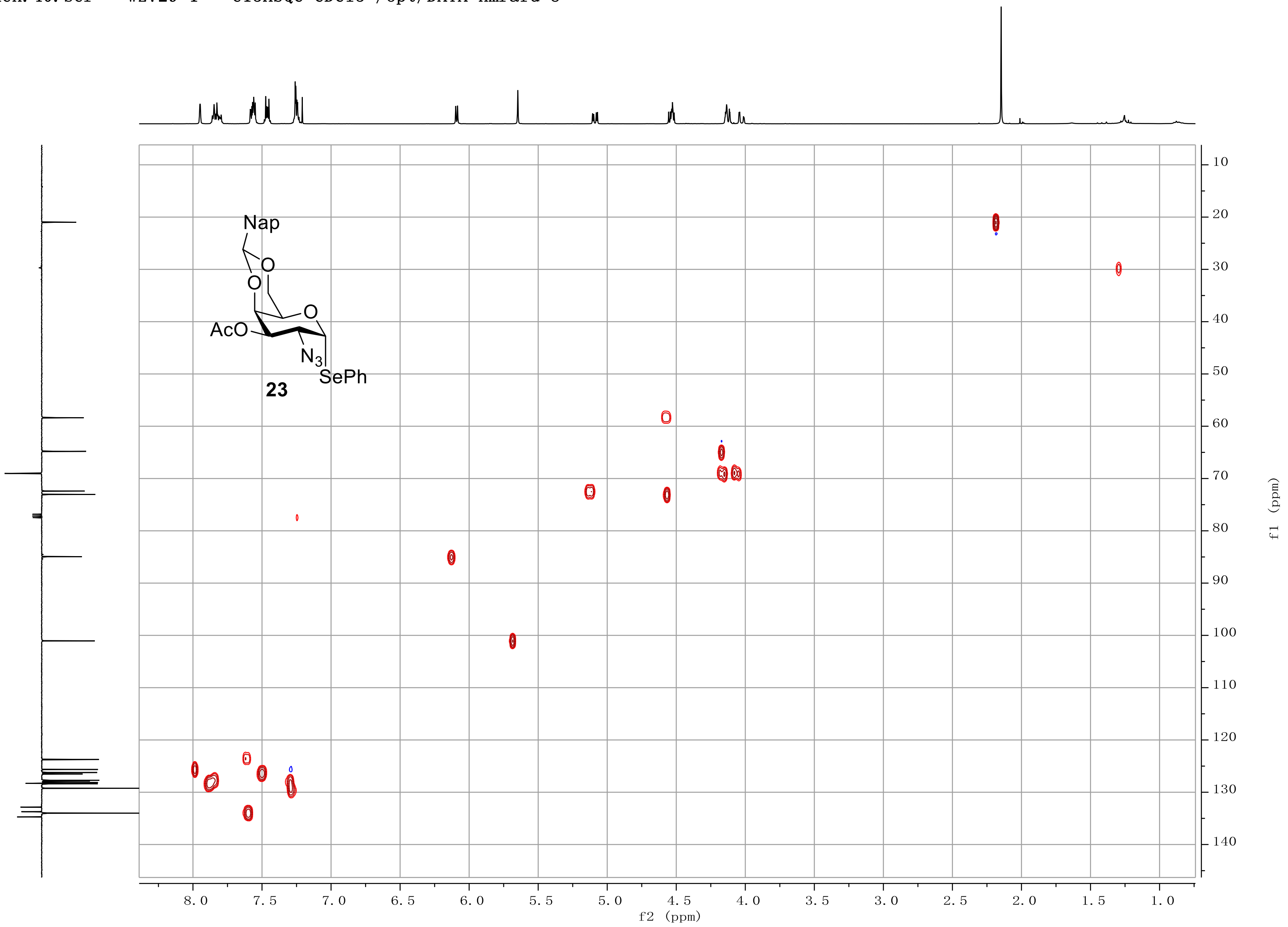

2009zhen.41.ser — wz729-1 — c13HMBC CDCl3 /opt/DATA nmrafd 8

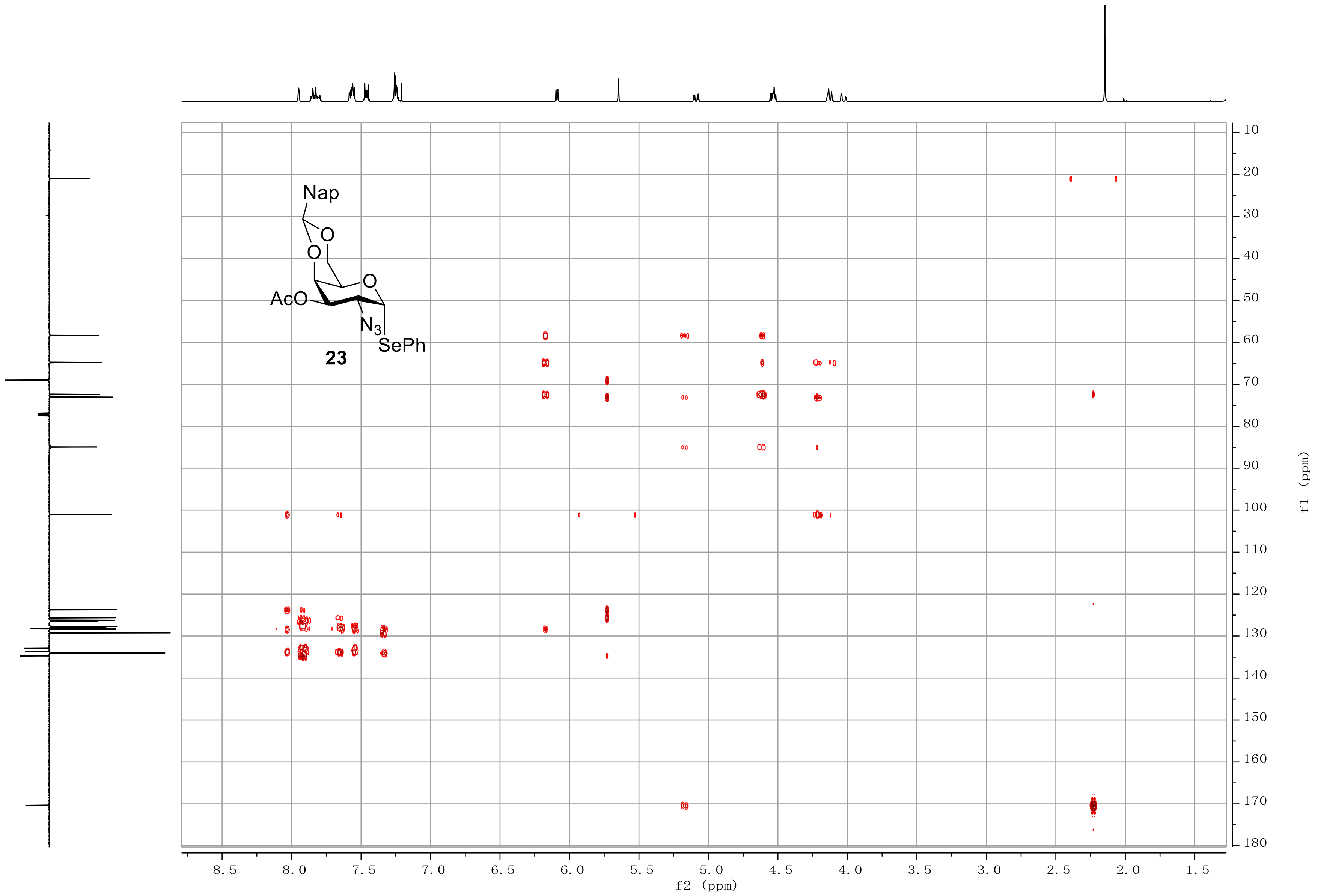

2010zhen.33.fid — wz731-a-1-1 — h1 CDCl3 /opt/DATA nmrafd 12

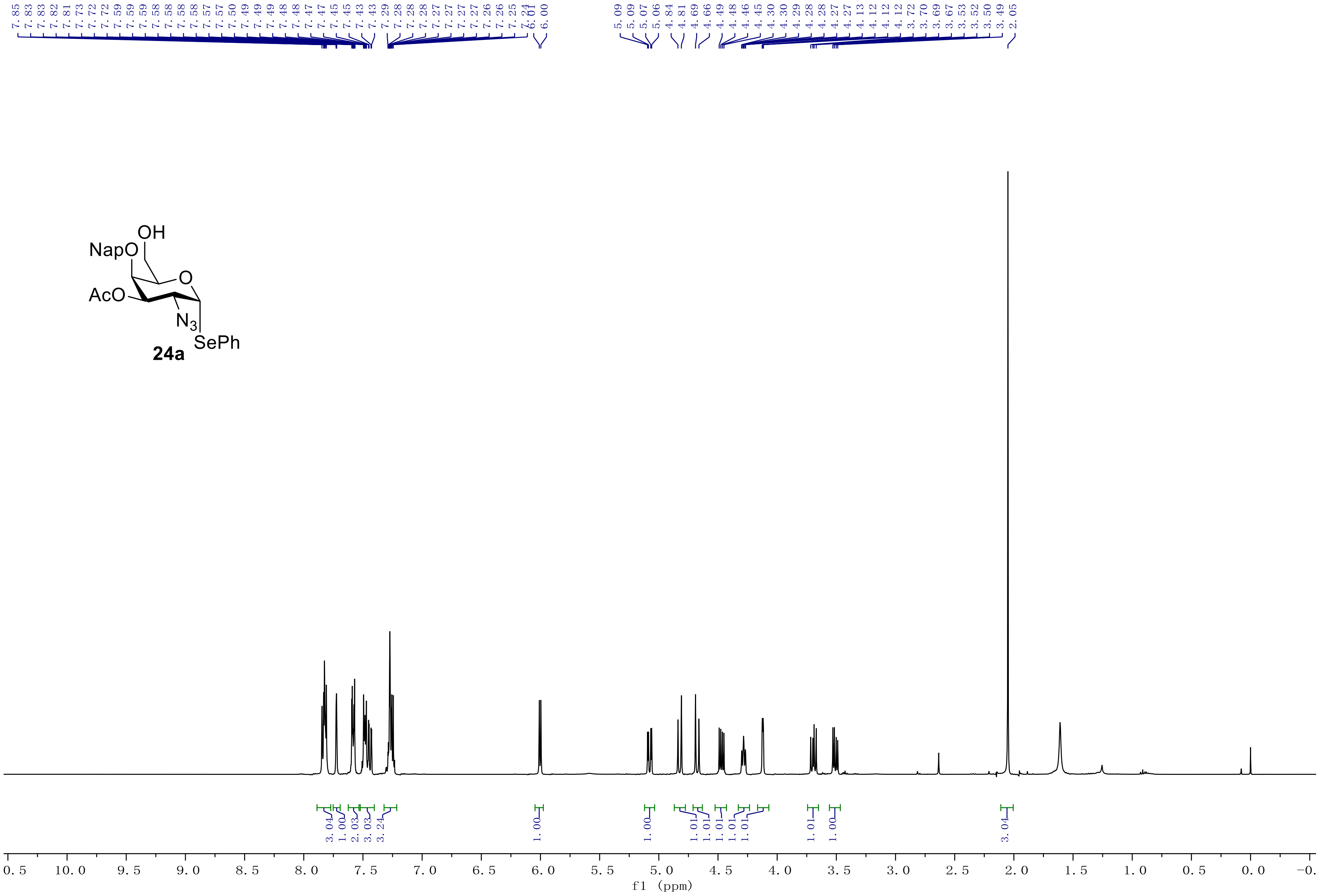

24a

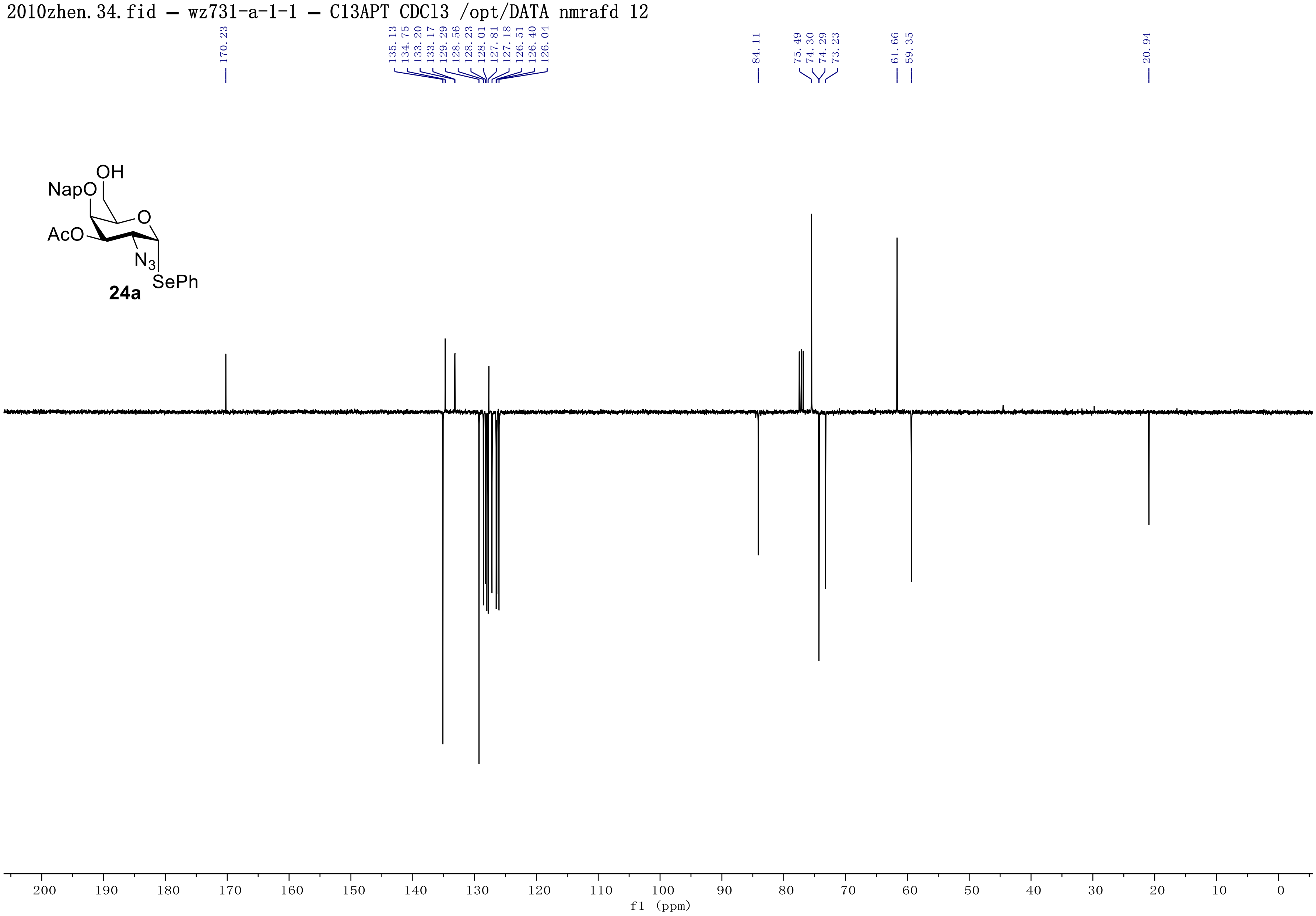
2010zhen.34.fid — wz731-a-1-1 — C13APT CDCl3 /opt/DATA nmrafd 12
170.23
135.13
134.75
133.20
133.17
129.29
128.56
128.23
128.01
127.81
127.18
126.51
126.40
126.04
84.11
75.49
74.30
74.29
73.23
61.66
59.35
20.94
OH
NapO
O
AcO
N3
SePh
24a
200 190 180 170 160 150 140 130 120 110 100 90 80 70 60 50 40 30 20 10 0
f1 (ppm)

2010zhen.35.ser — wz731-a-1-1 — h1COSY CDCl3 /opt/DATA nmrafd 12

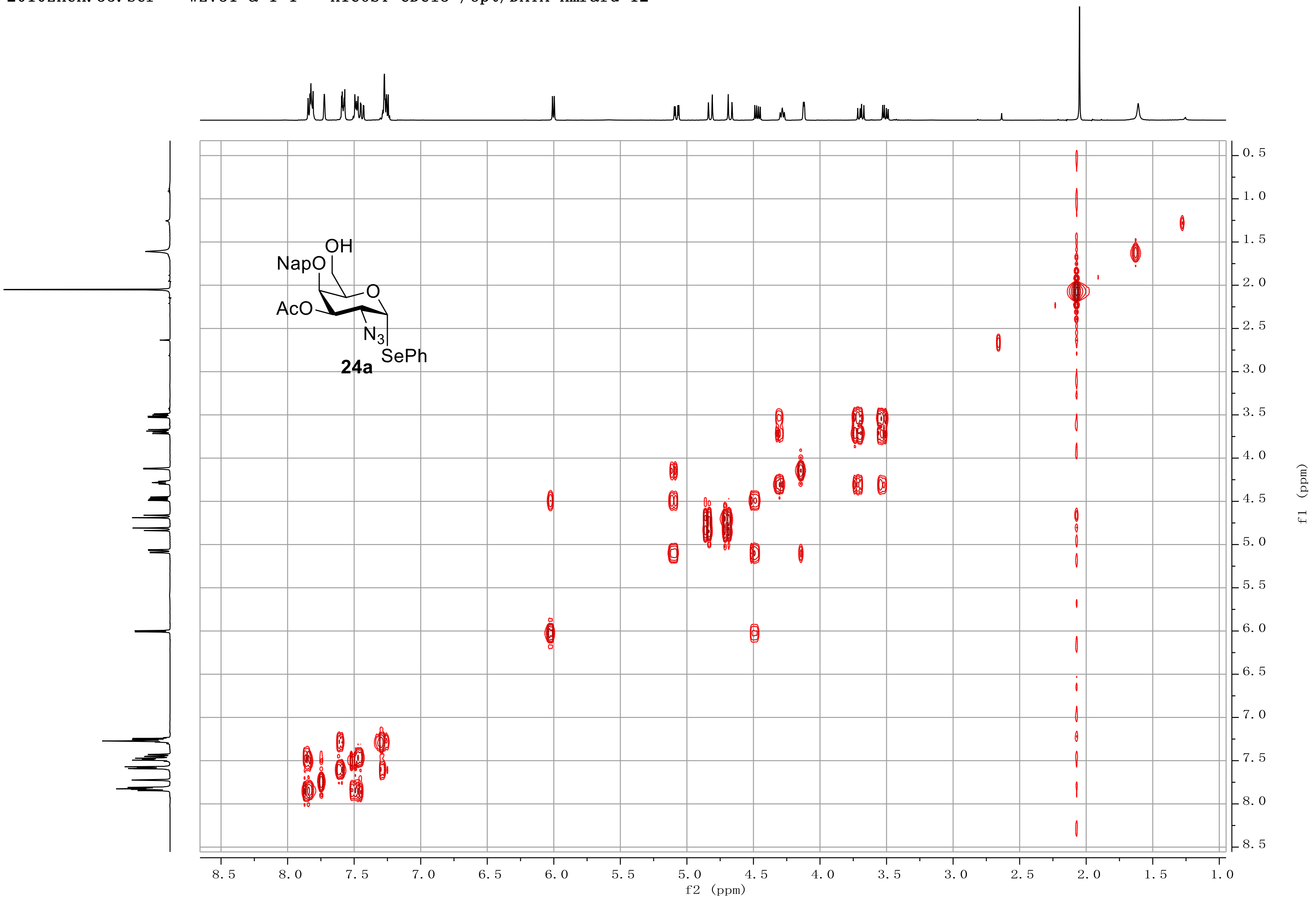

2010zhen.36.ser — wz731-a-1-1 — c13HSQC CDCl3 /opt/DATA nmrafd 12

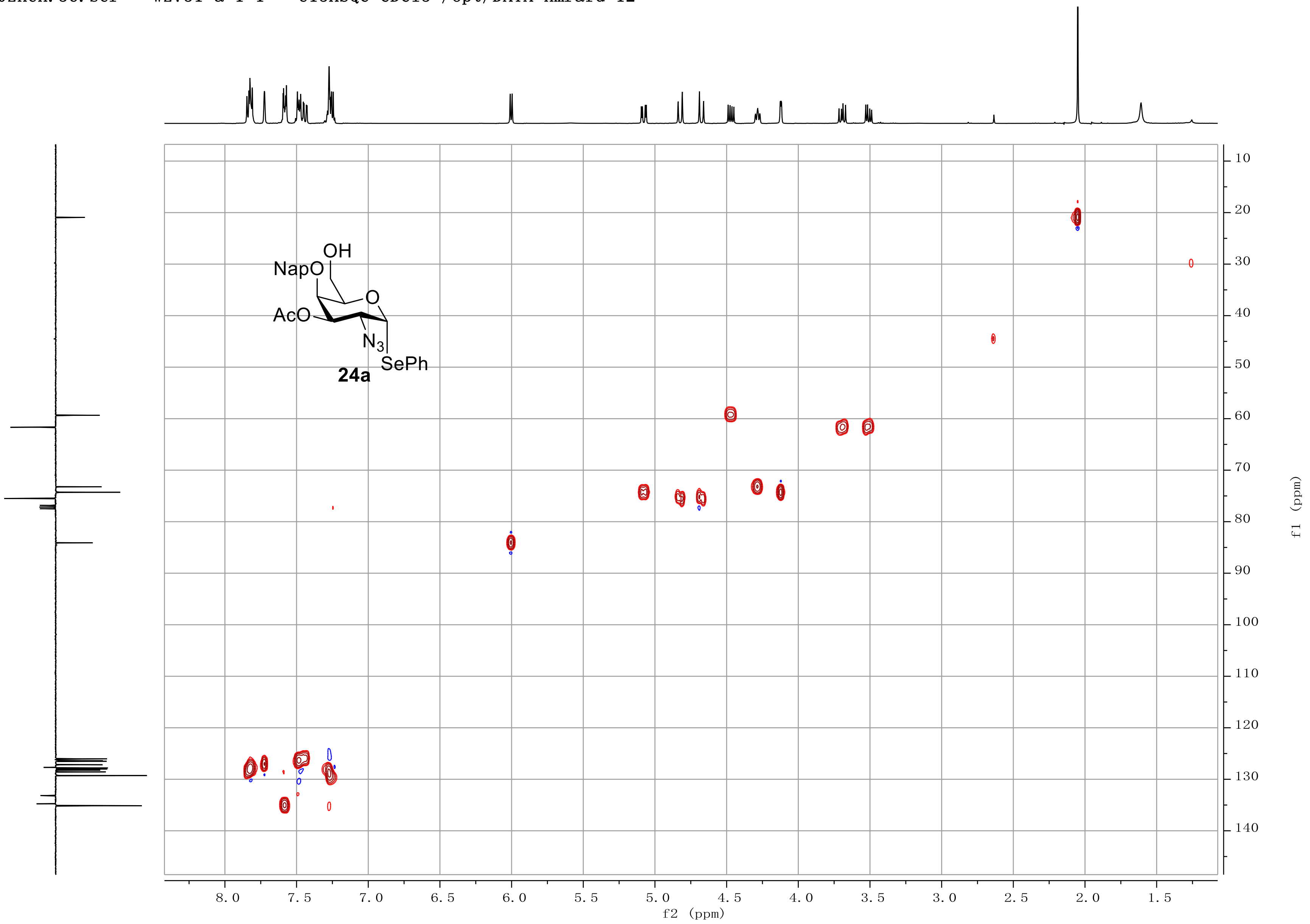

2010zhen.37.ser — wz731-a-1-1 — c13HMBC CDCl3 /opt/DATA nmrafd 12

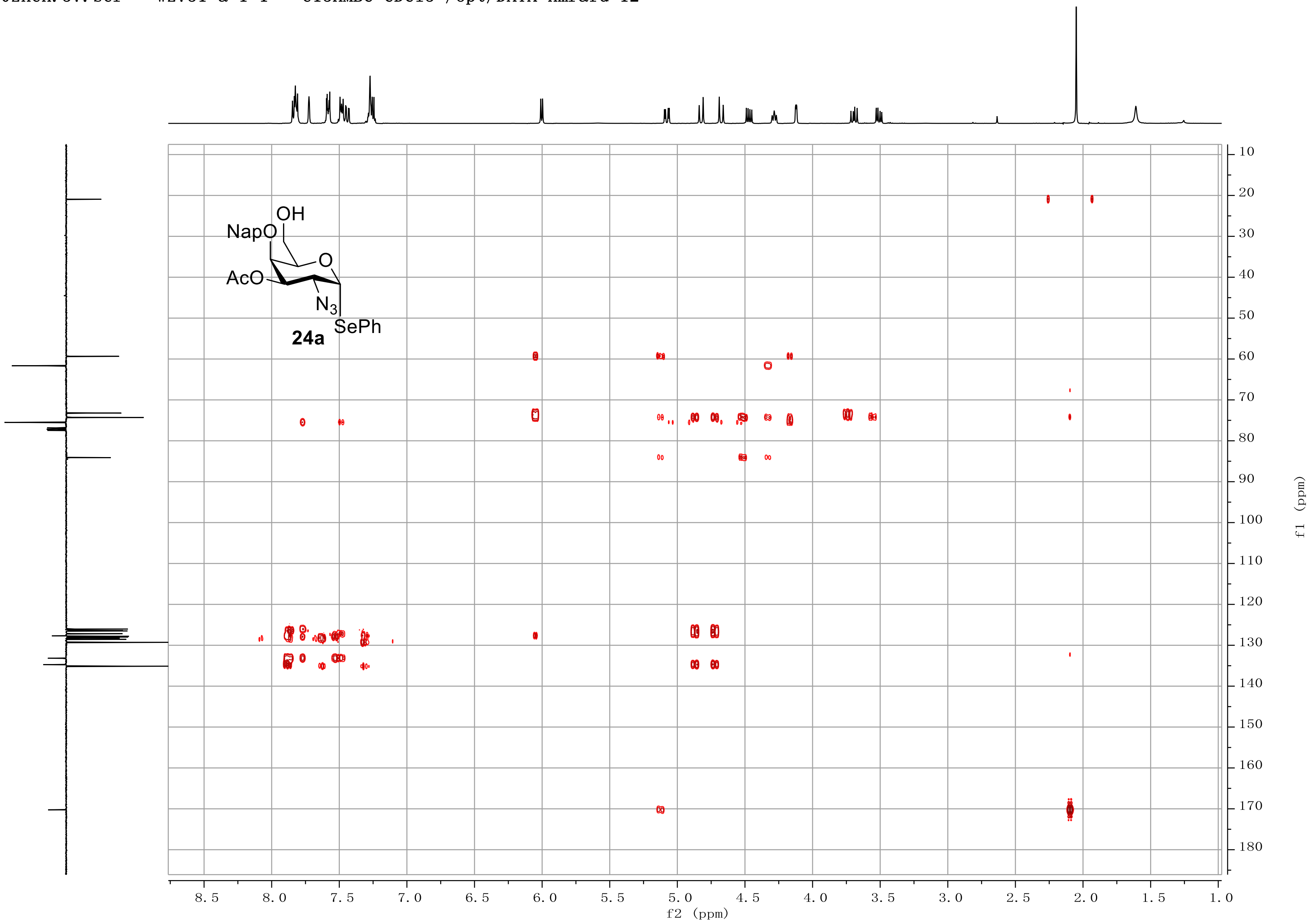

zhen2007Biosyn.6.fid — wz707-1 — bbo-h1 Acetone /opt/topspin2.1 nmrafd 10

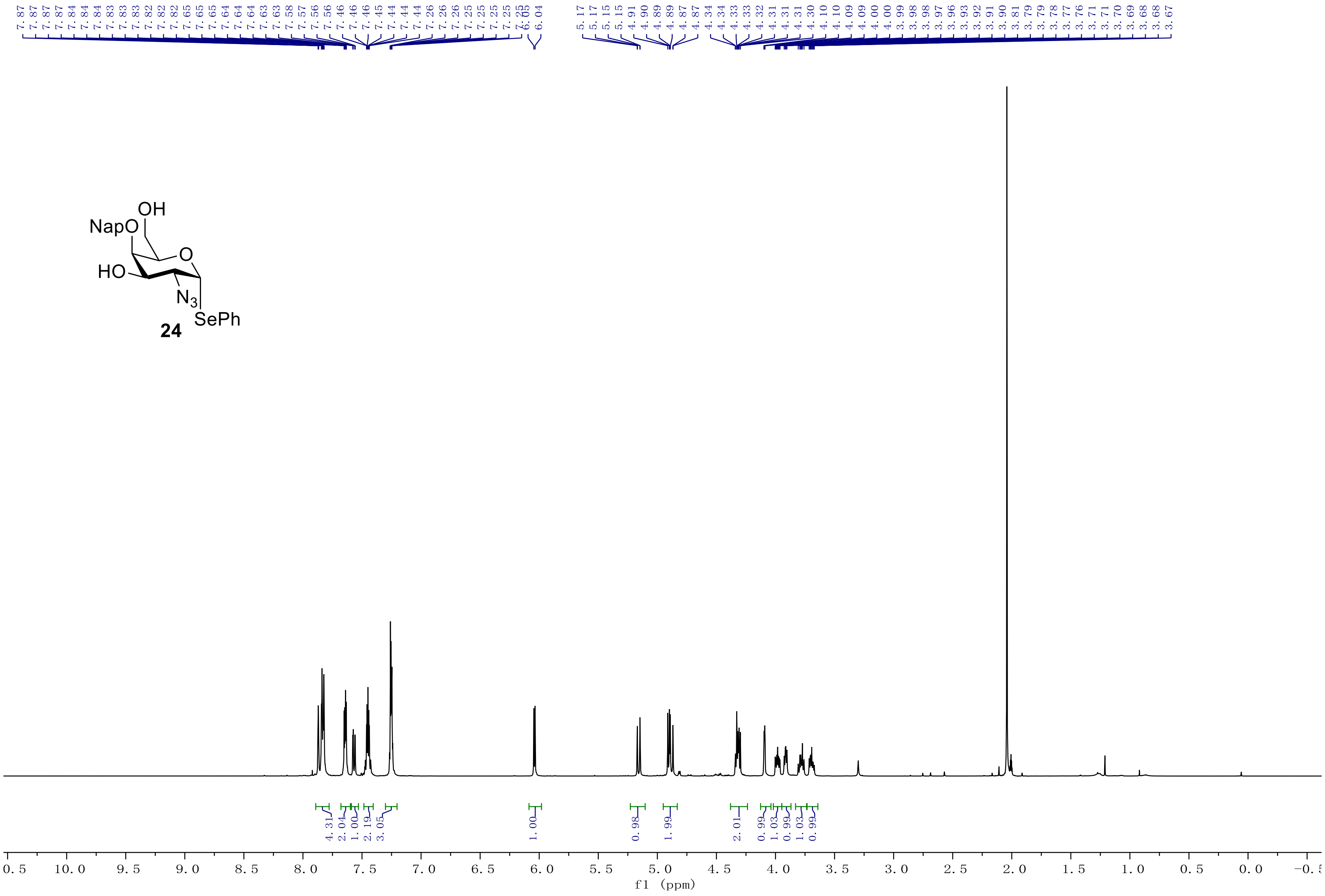

zhen2007Biosyn.9.fid — wz707-1 — bbo-c13-APT Acetone /opt/topspin2.1 nmrafd 10

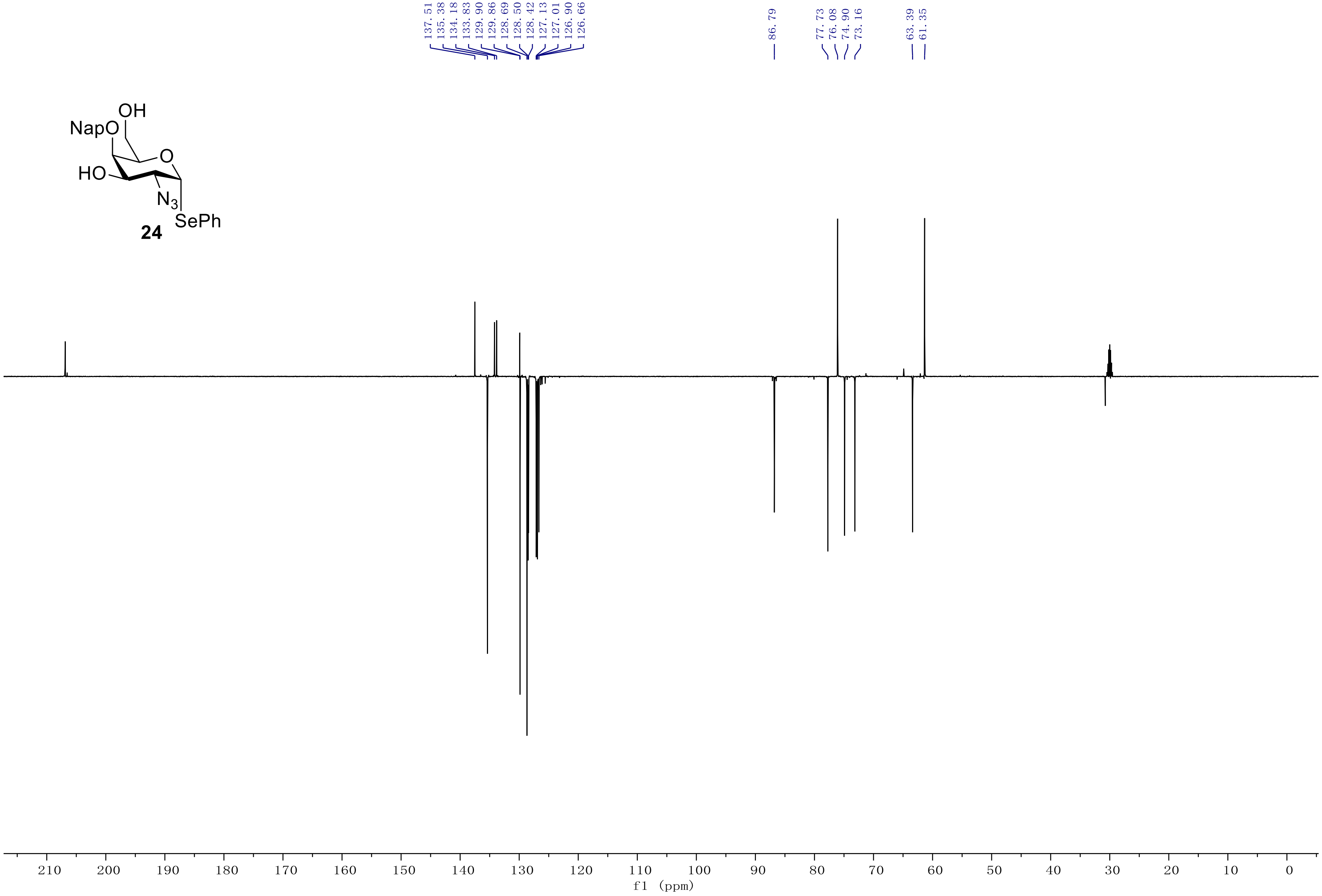

zhen2007Biosyn.7.ser — wz707-1 — bbo-h1-cosy Acetone /opt/topspin2.1 nmrafd 10

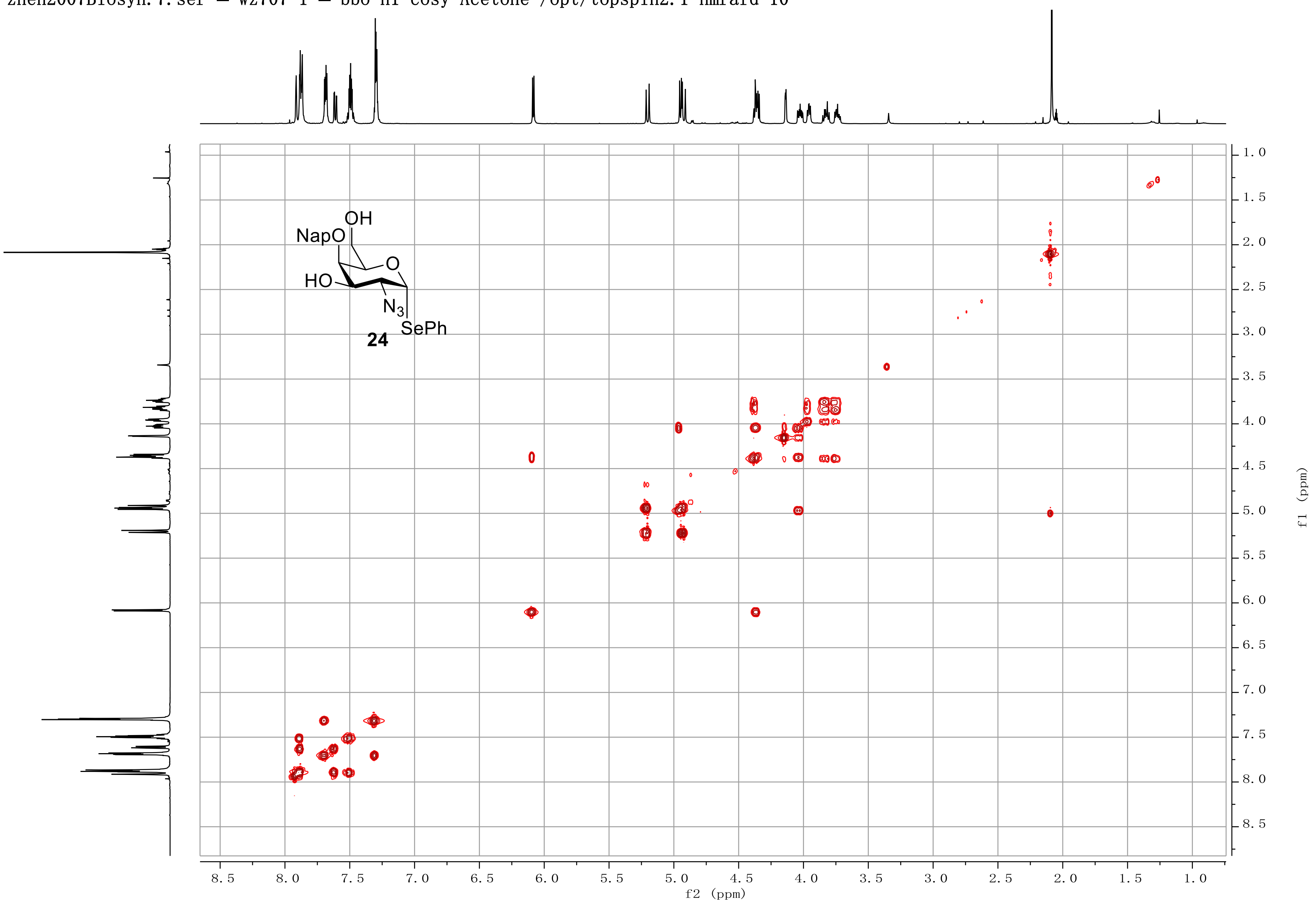

zhen2007Biosyn.8.ser — wz707-1 — bbo-c13-HSQC Acetone /opt/topspin2.1 nmrafd 10

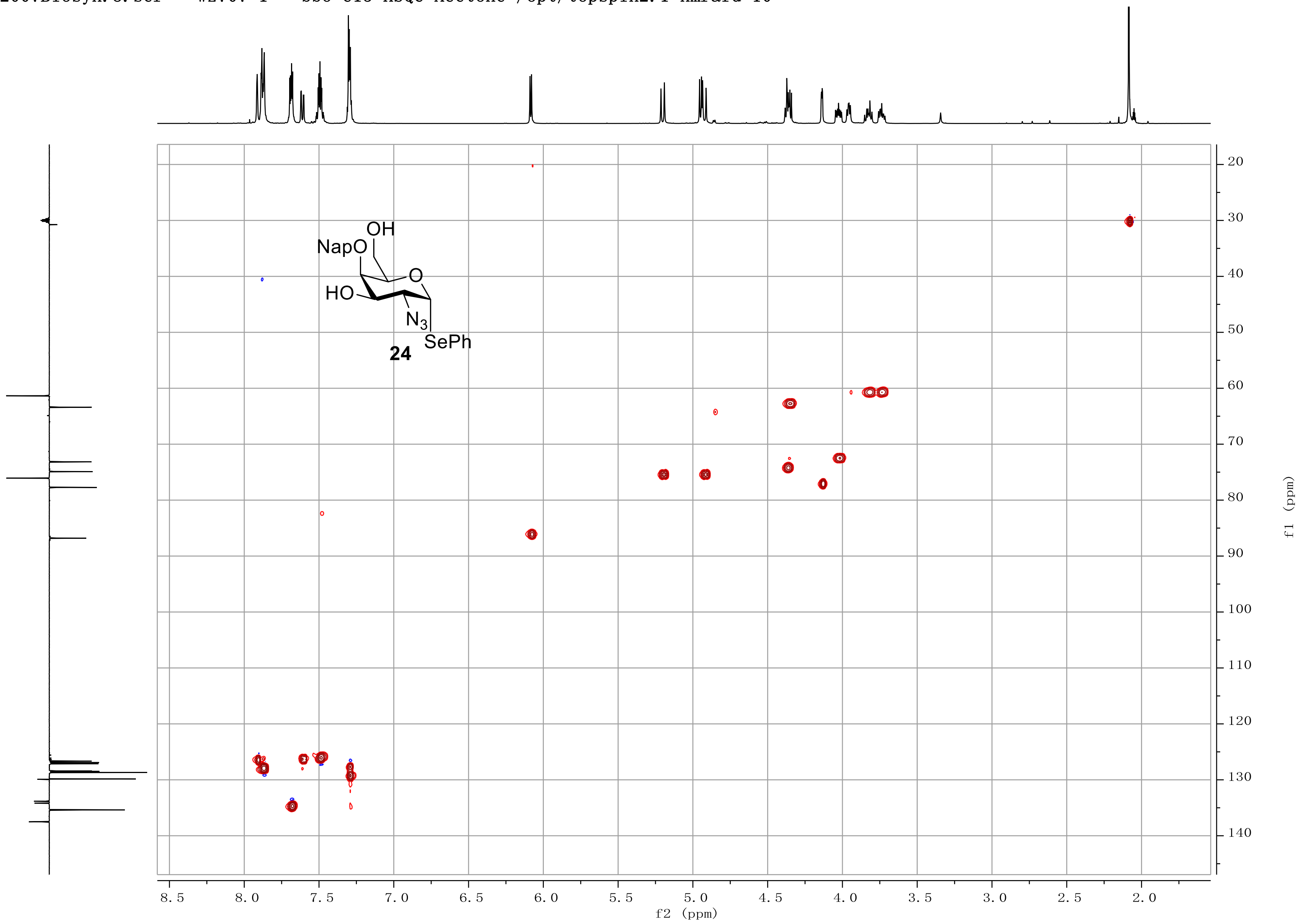

zhen2007Biosyn.10.ser — wz707-1 — bbo-c13-HMBC Acetone /opt/topspin2.1 nmrafd 10

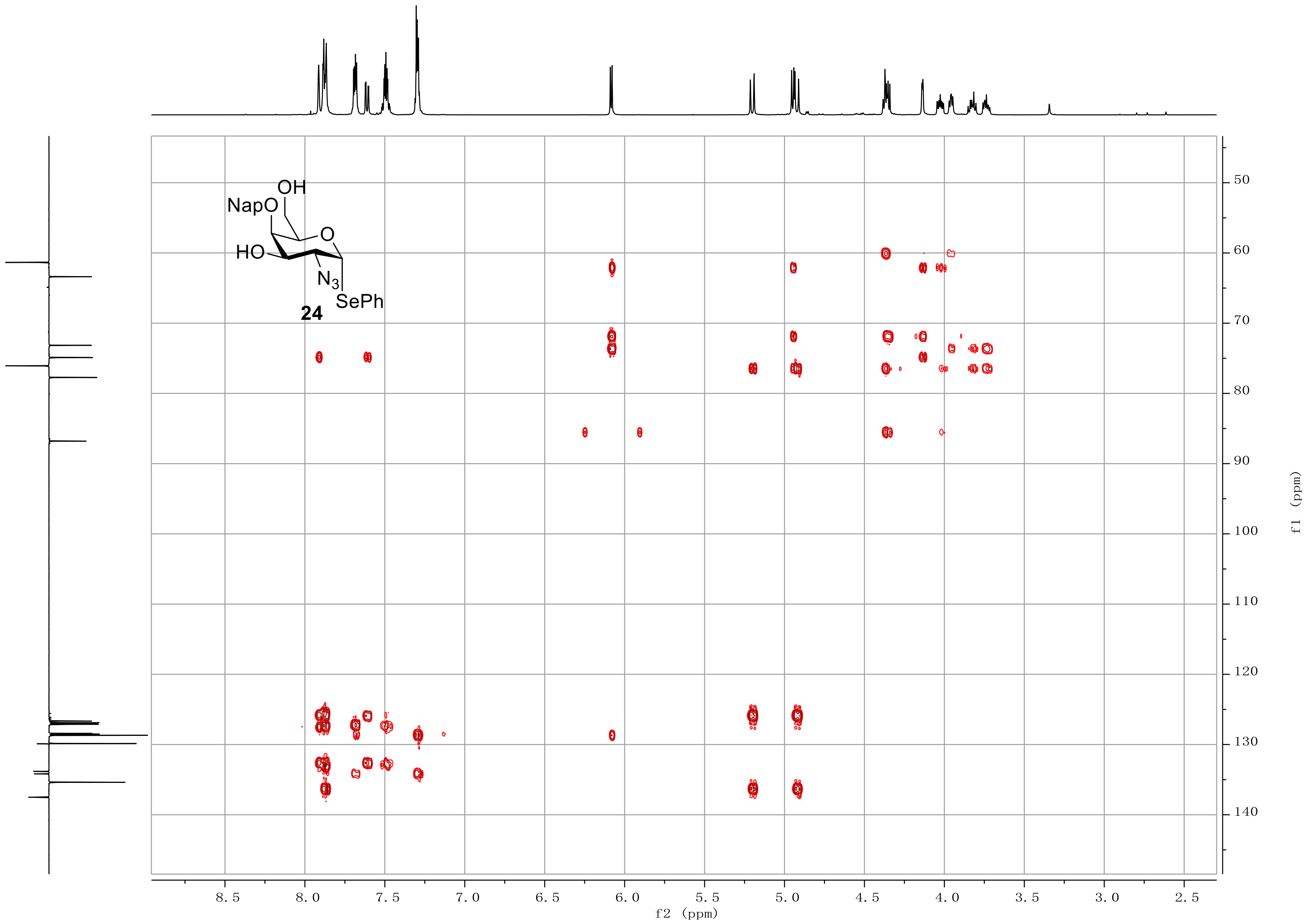

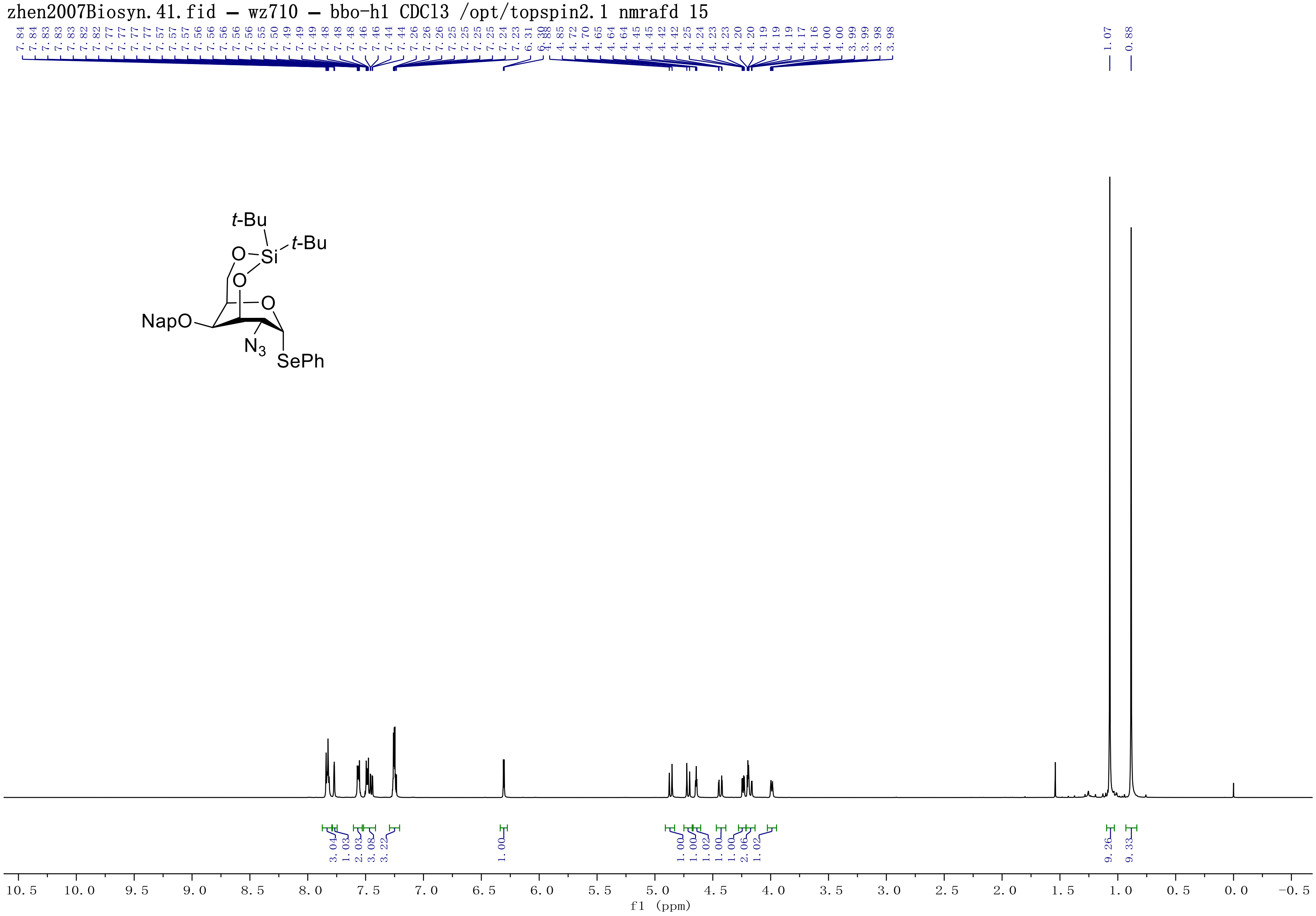
zhen2007Biosyn.41.fid — wz710 — bbo-h1 CDCl3 /opt/topspin2.1 nmrafd 15
t-Bu
O
Si
t-Bu
O
O
NapO
N3
SePh
f1 (ppm)

zhen2007Biosyn.44.fid — wz710 — bbo-c13-APT CDCl3 /opt/topspin2.1 nmrafd 15

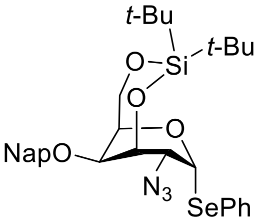

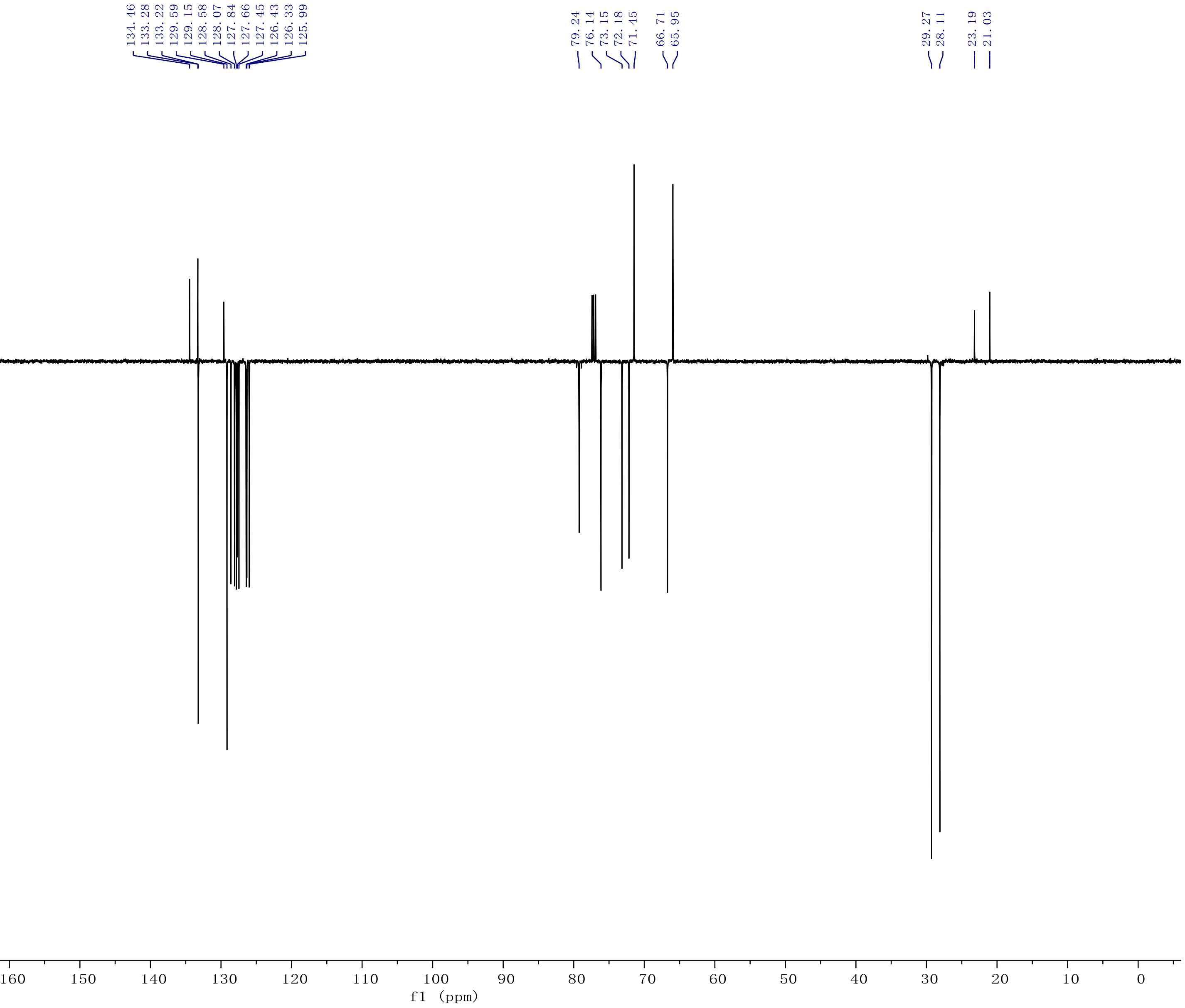

zhen2007Biosyn.42.ser — wz710 — bbo-h1-cosy CDCl3 /opt/topspin2.1 nmrafd 15

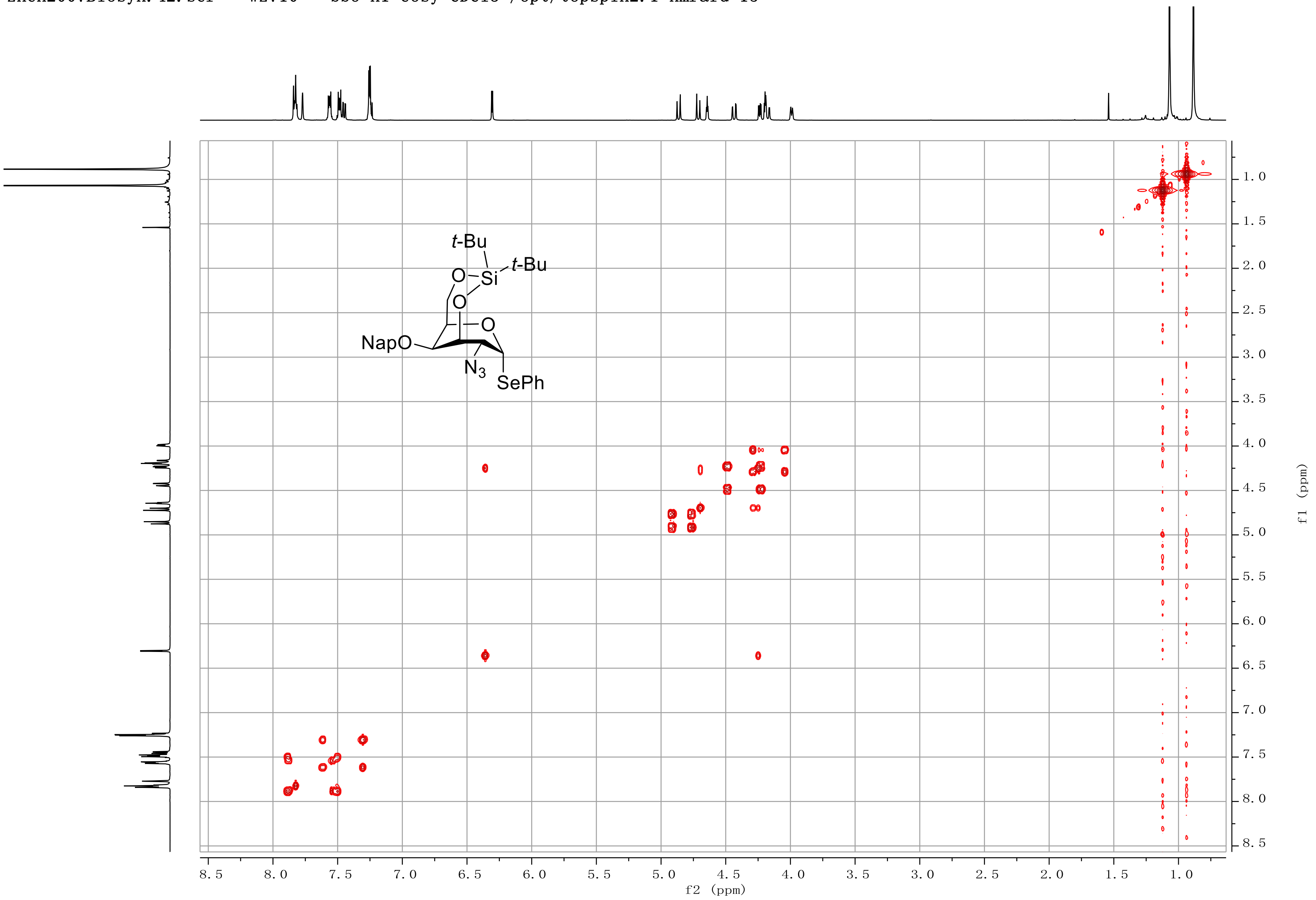

zhen2007Biosyn.43.ser — wz710 — bbo-c13-HSQC CDCl3 /opt/topspin2.1 nmrafd 15

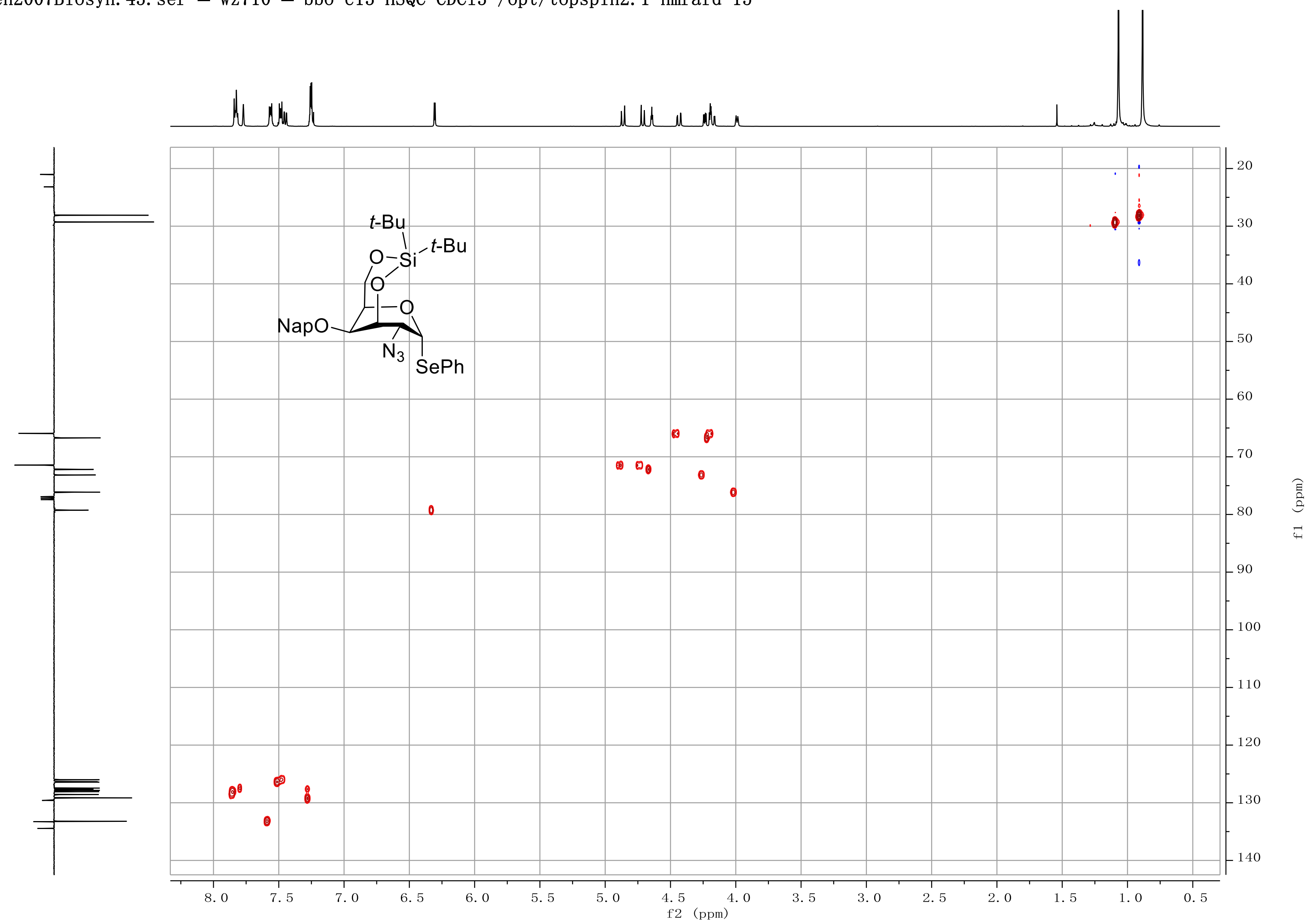

zhen2007Biosyn.45.ser — wz710 — bbo-c13-HMBC CDCl3 /opt/topspin2.1 nmrafd 15

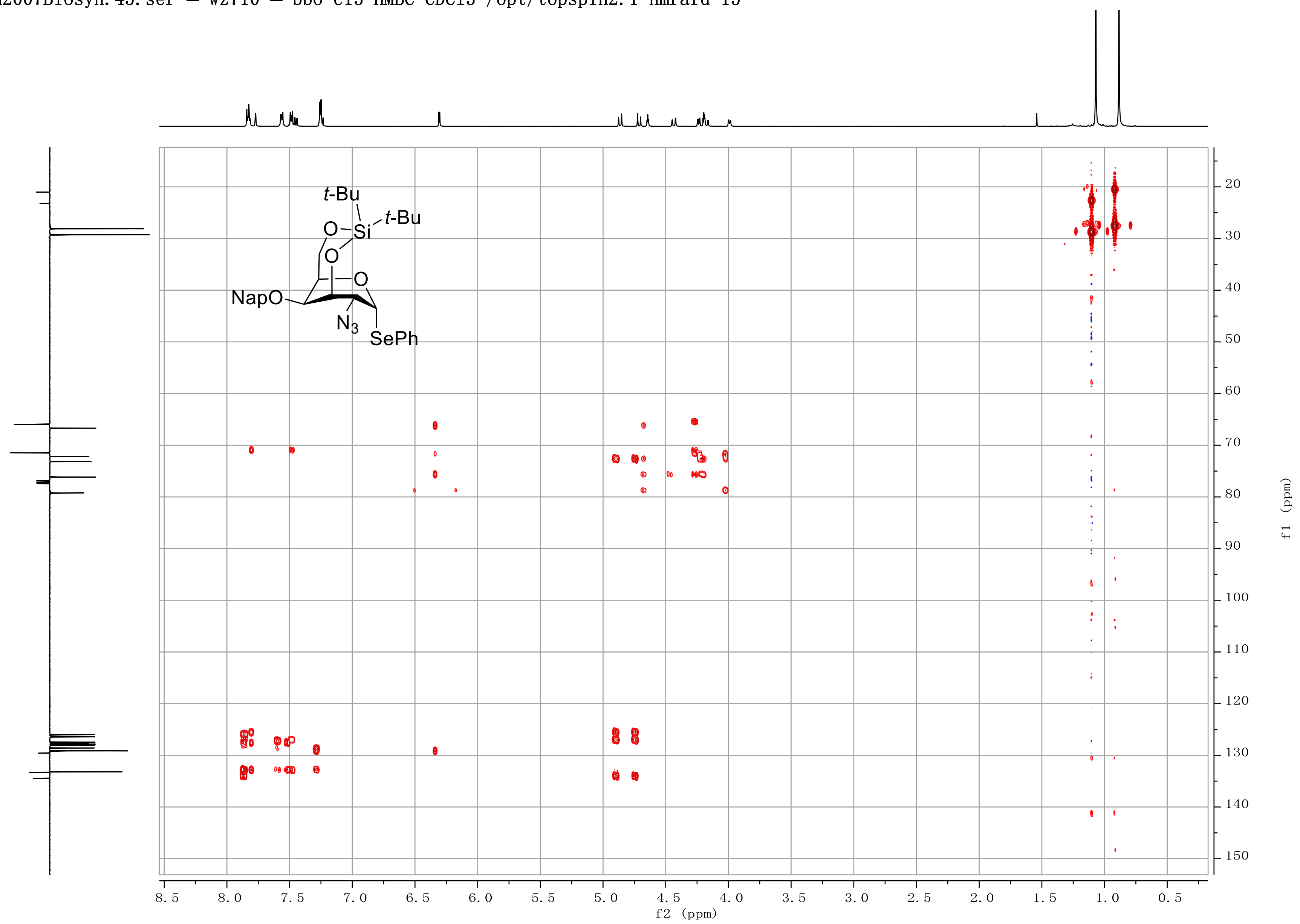

zhen2007Biosyn.69.fid — wz712 — bbo-h1 CDCl3 /opt/topspin2.1 nmrafd 5

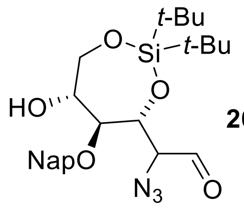

26b

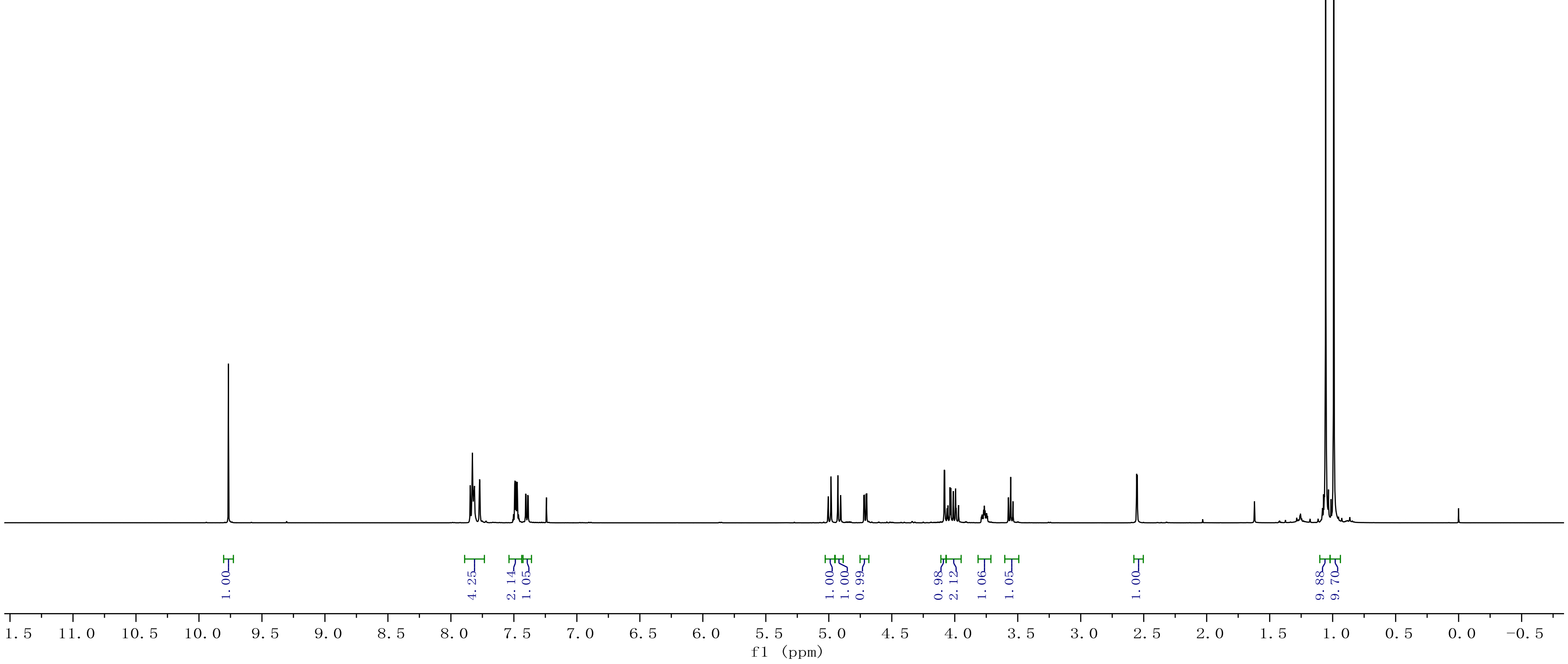

zhen2007Biosyn.72.fid — wz712 — bbo-c13-APT CDCl3 /opt/topspin2.1 nmrafd 5

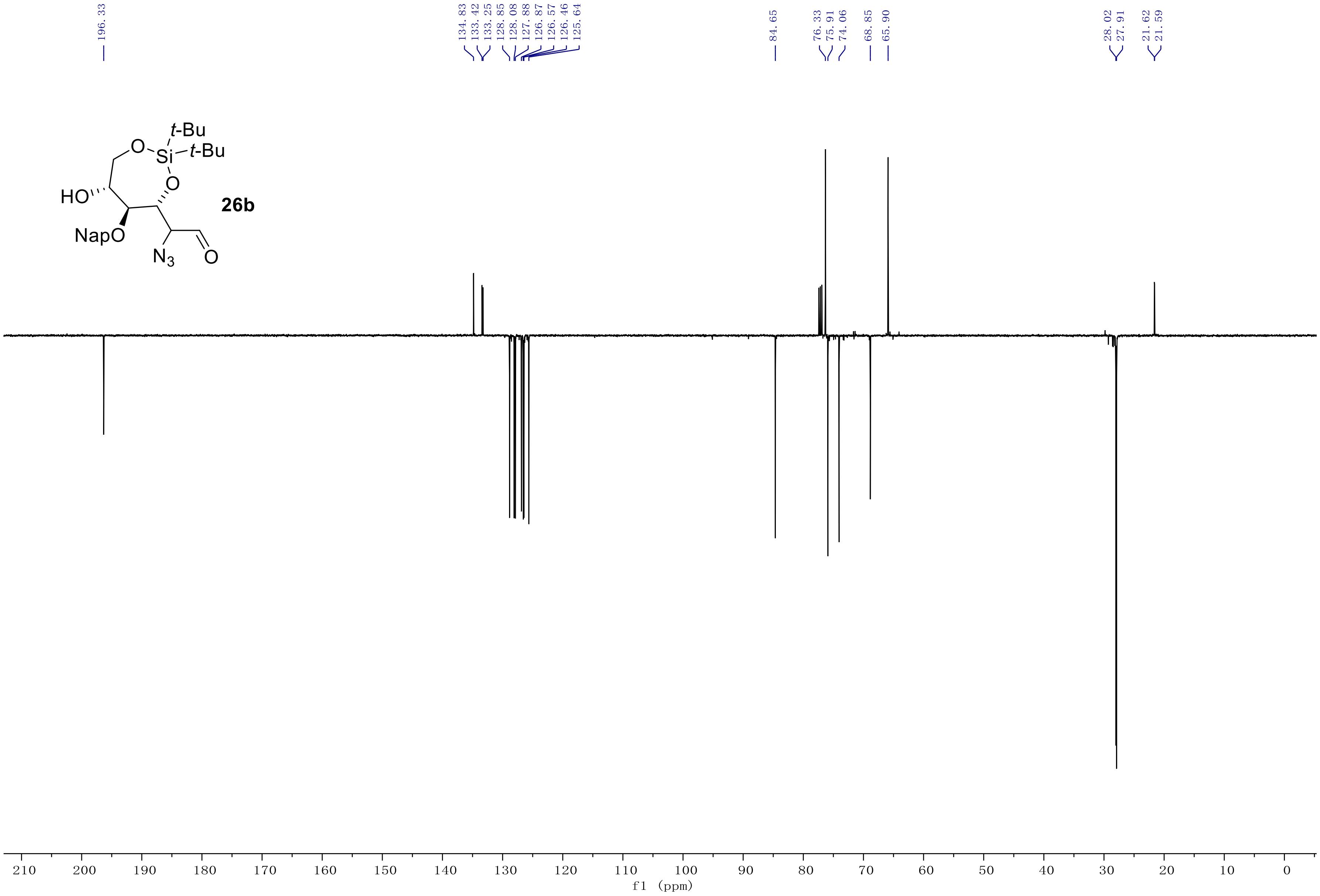

zhen2007Biosyn.70.ser — wz712 — bbo-h1-cosy CDCl3 /opt/topspin2.1 nmrafd 5

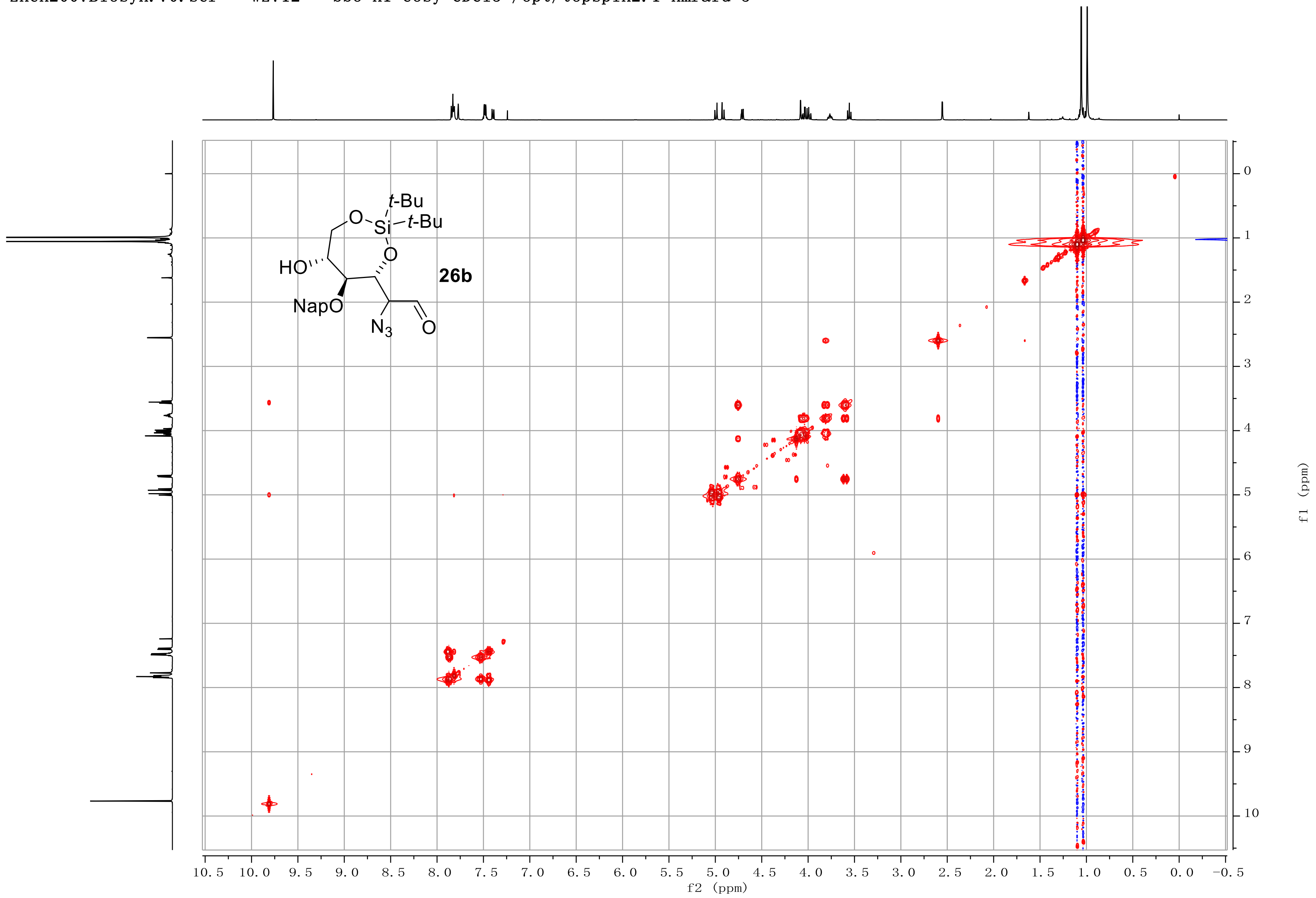

zhen2007Biosyn.74.ser — HSQC av500, bbo, 200 ppm wz712

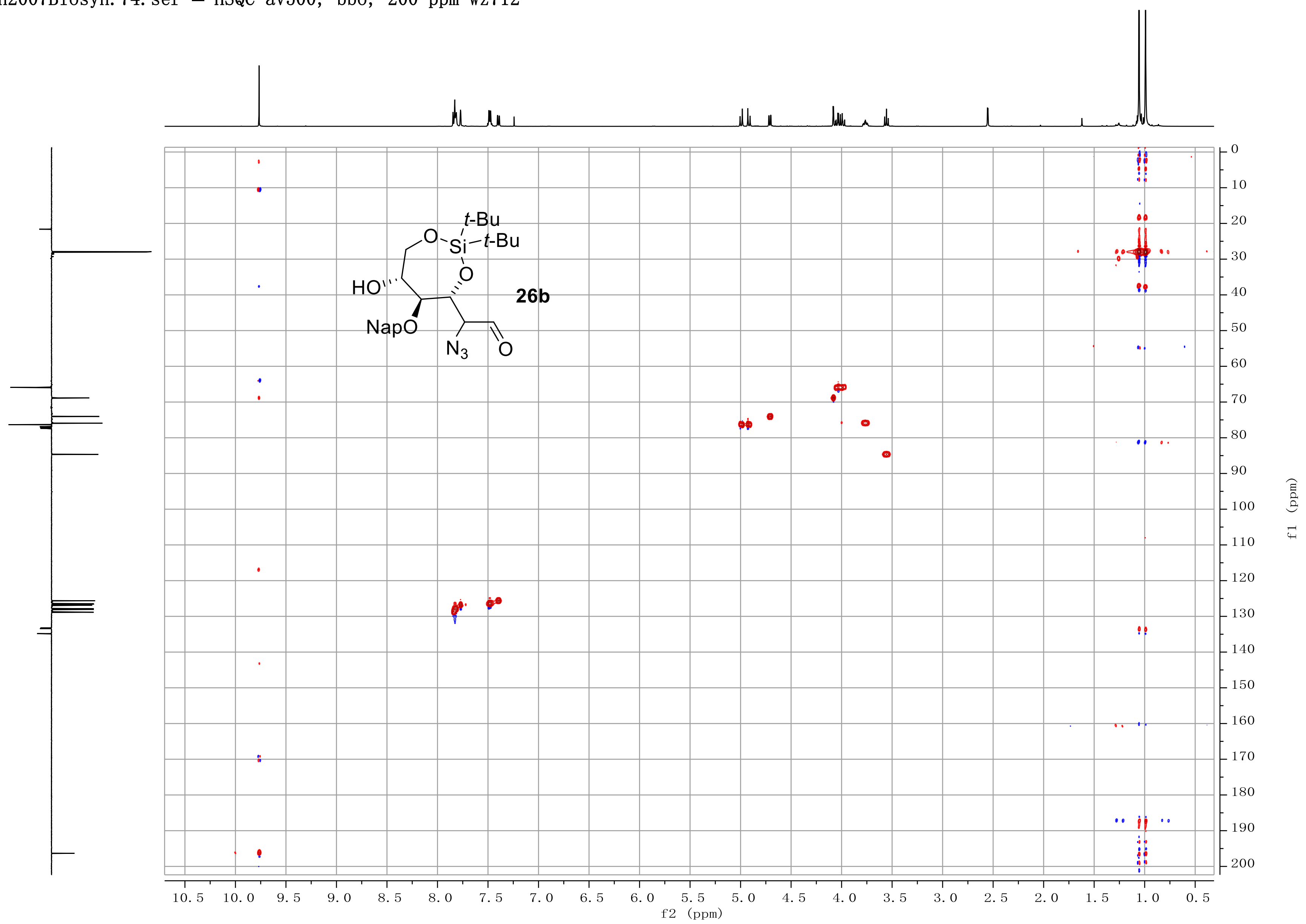

zhen2007Biosyn.73.ser — wz712 — bbo-c13-HMBC CDCl3 /opt/topspin2.1 nmrafd 5

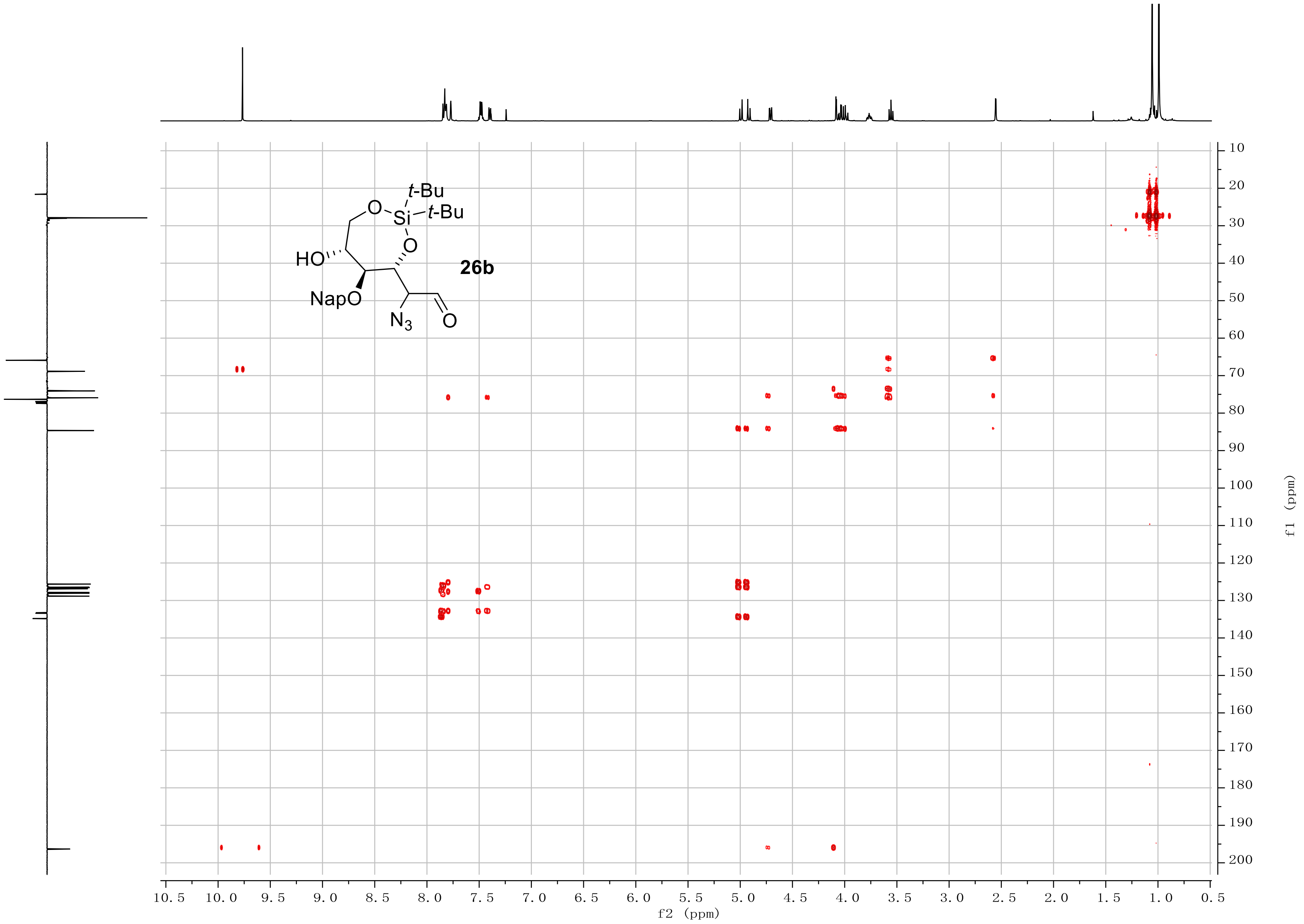

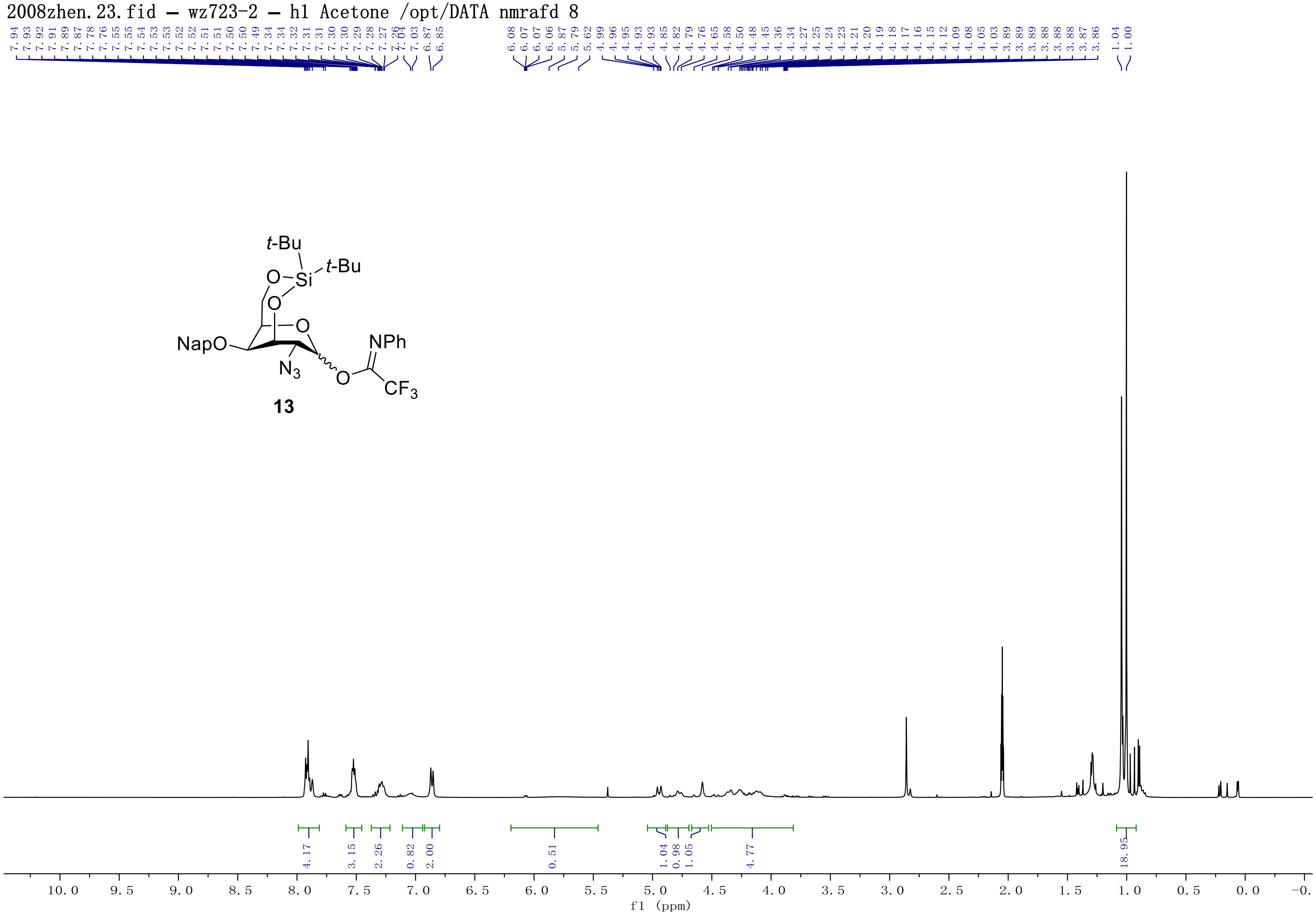

2008zhen.23.fid — wz723-2 — h1 Acetone /opt/DATA nmrafd 8
t-Bu
t-Bu
O
Si
O
O
NapO
N3
NPh
O
CF3
13
f1 (ppm)

2008zhen.24.fid — wz723-2 — C13APT Acetone /opt/DATA nmrafd 8

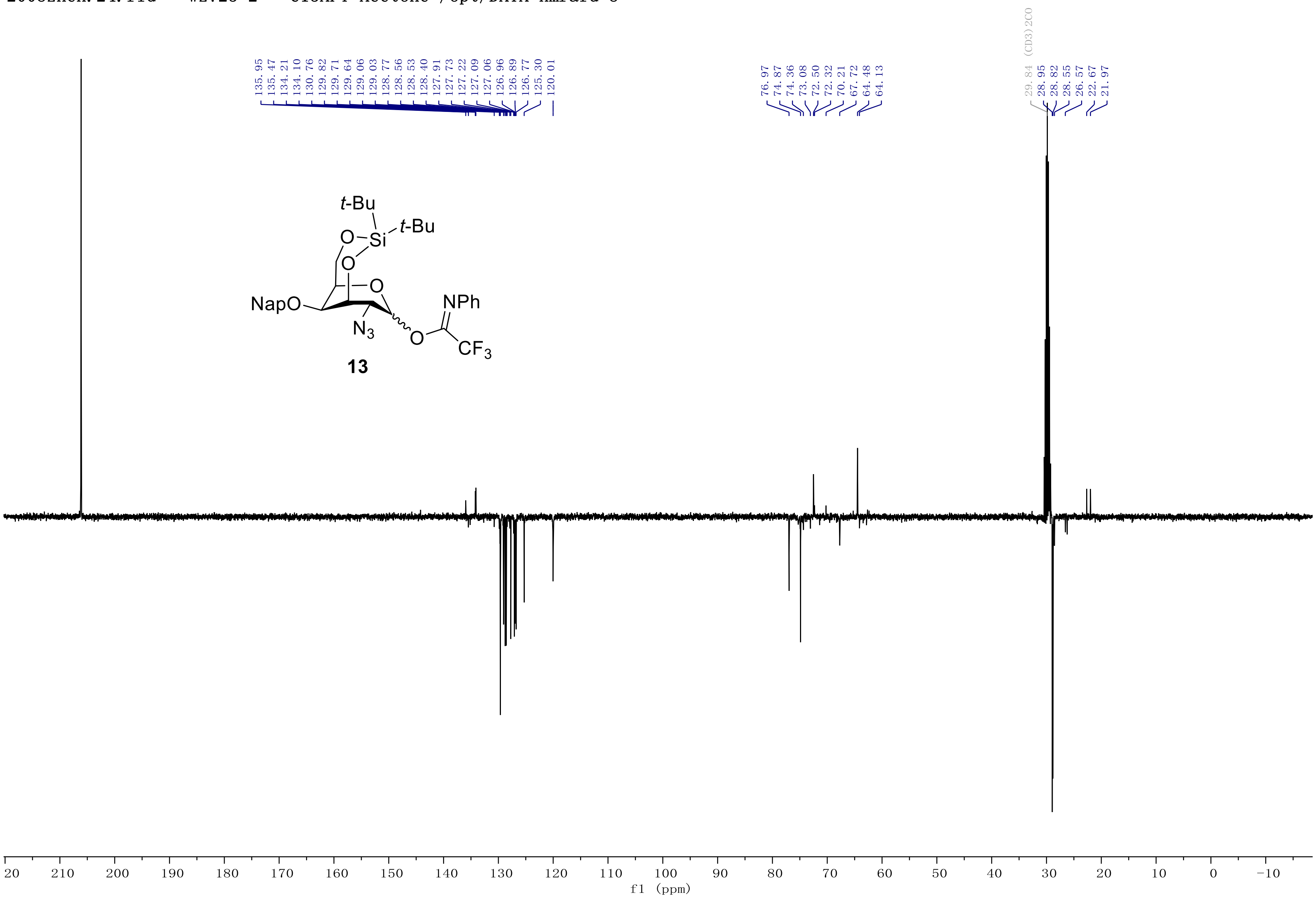

2008zhen.25.ser — wz723-2 — h1COSY Acetone /opt/DATA nmrafd 8

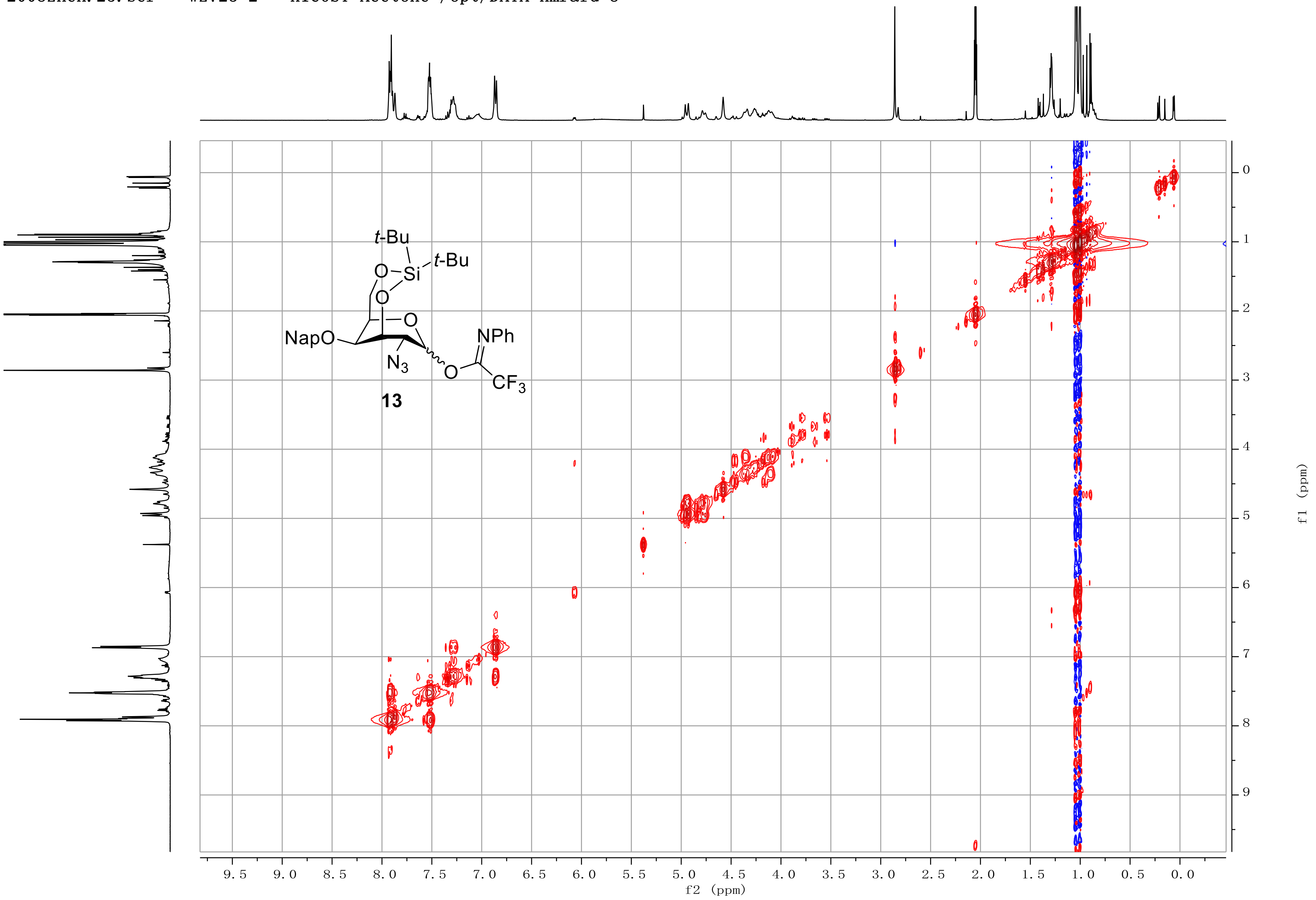

2008zhen.26.ser — wz723-2 — c13HSQC Acetone /opt/DATA nmrafd 8

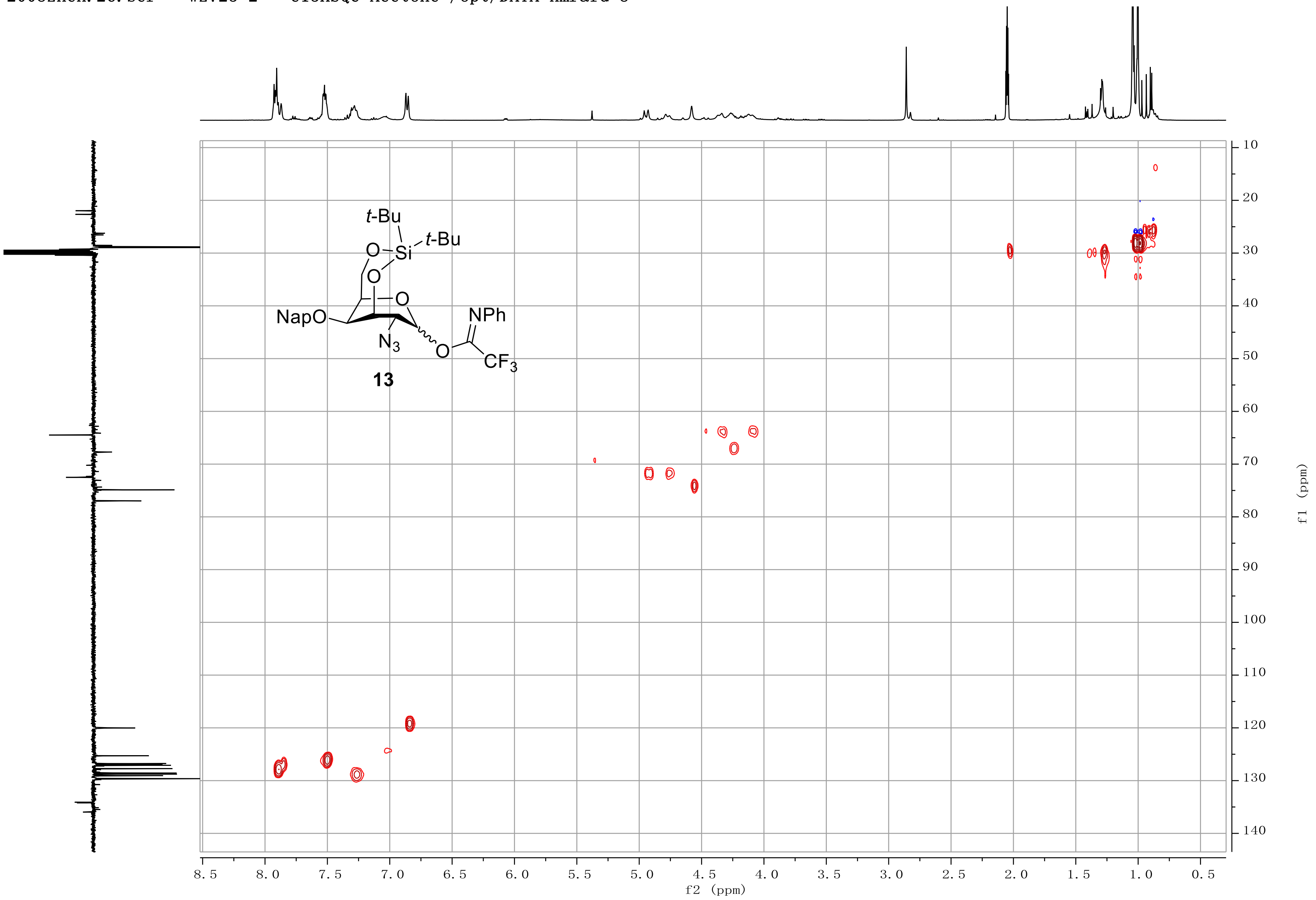

zhen2108biosyn.21.fid — wz753-C-s — bbo-h1 CDCl3 /opt/topspin2.1 nmrafd 4

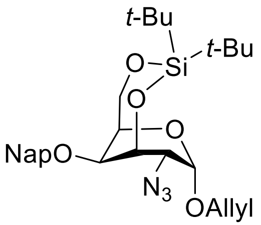

14a

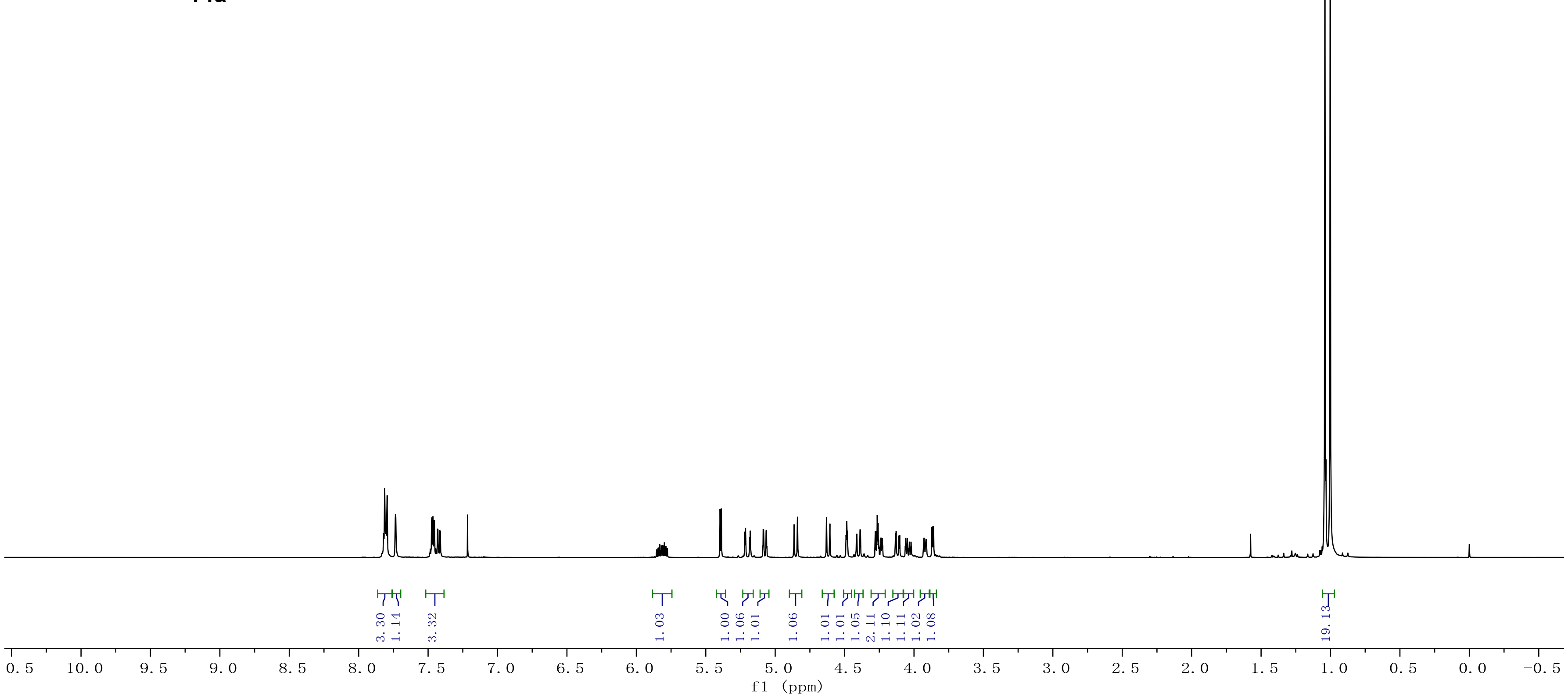

zhen2108biosyn.24.fid — wz753-C-s — bbo-c13-APT CDCl3 /opt/topspin2.1 nmrafd 4

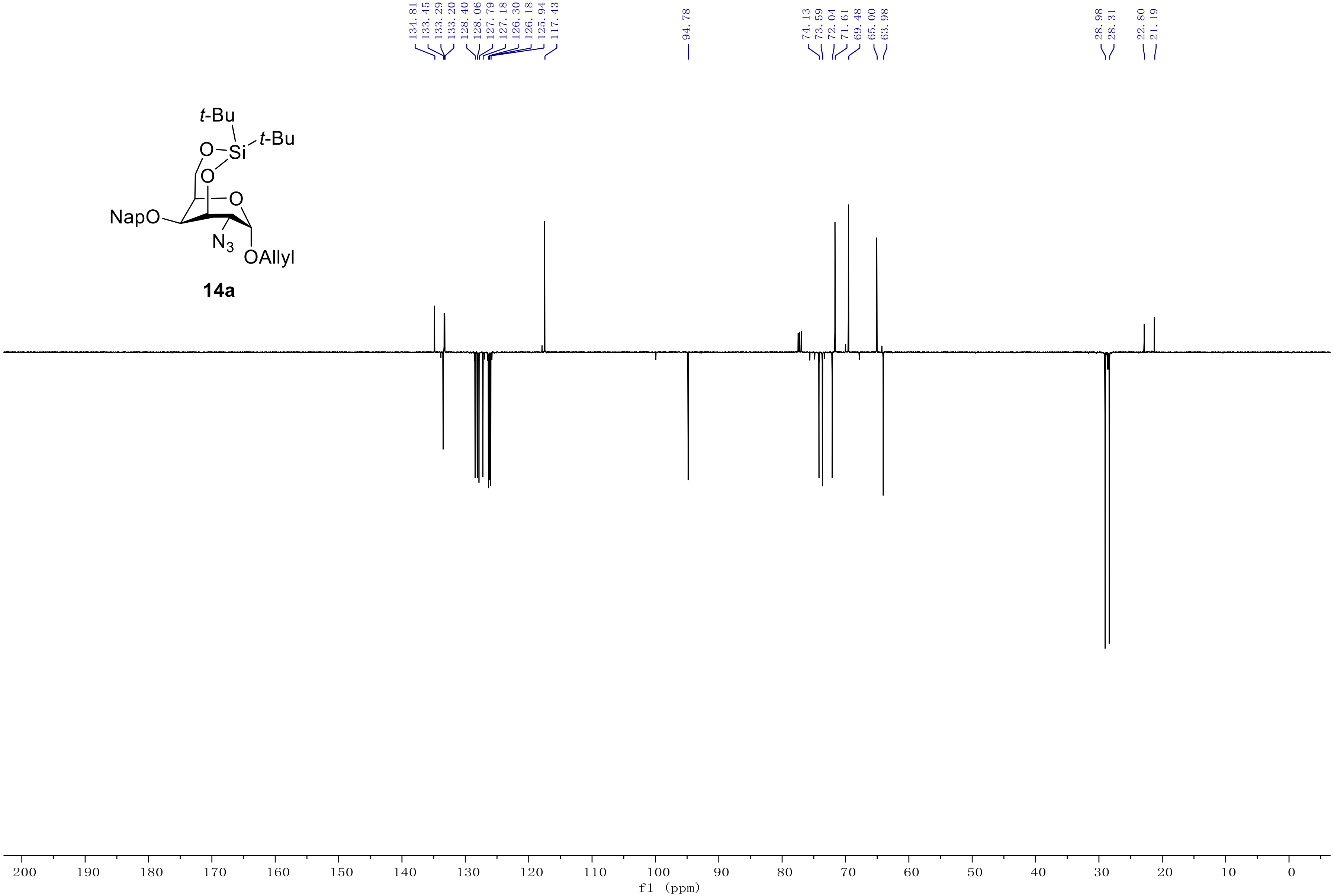

14a

zhen2108biosyn.22.ser — wz753-C-s — bbo-h1-cosy CDCl3 /opt/topspin2.1 nmrafd 4

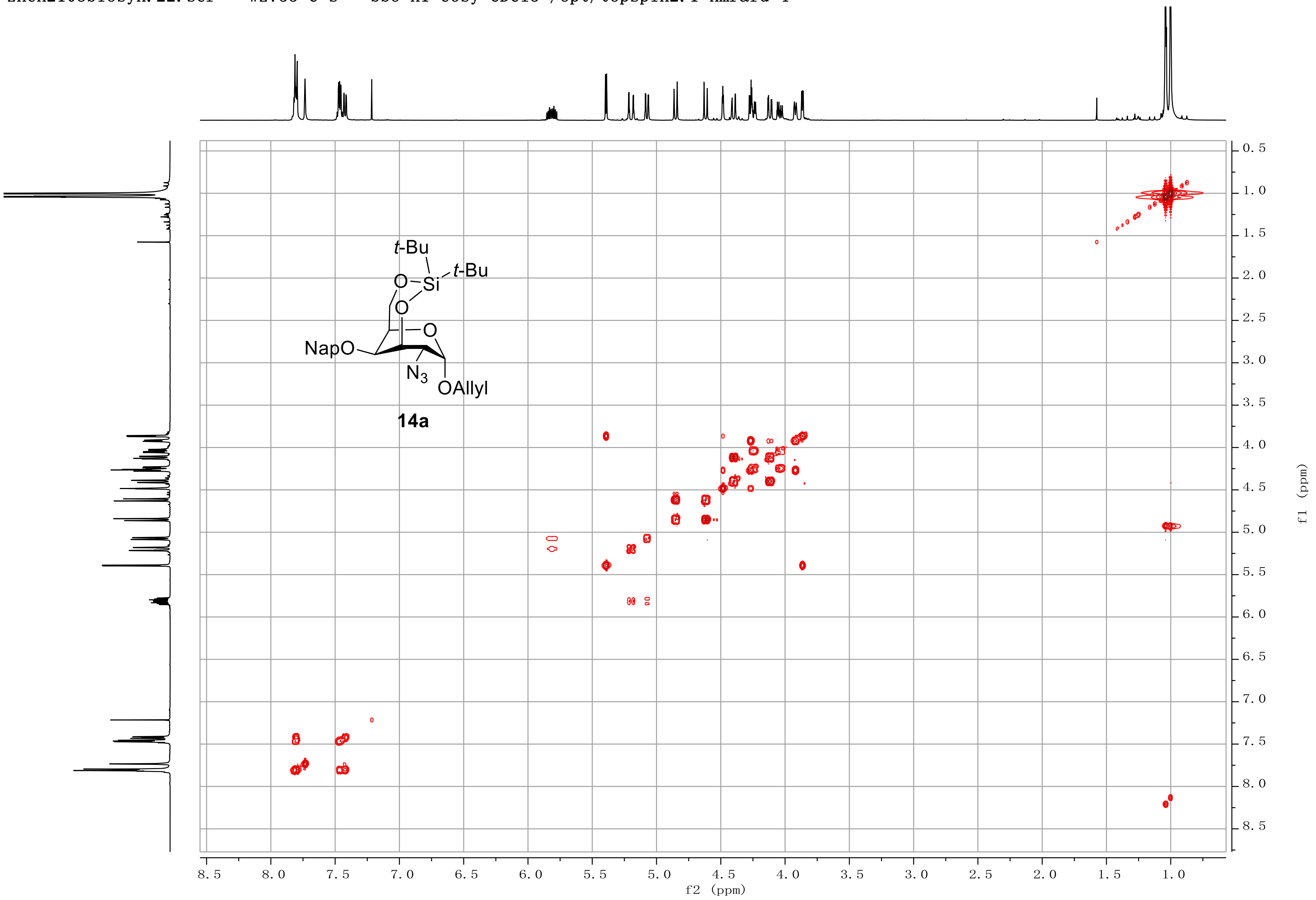

zhen2108biosyn.23.ser — wz753-C-s — bbo-c13-HSQC CDCl3 /opt/topspin2.1 nmrafd 4

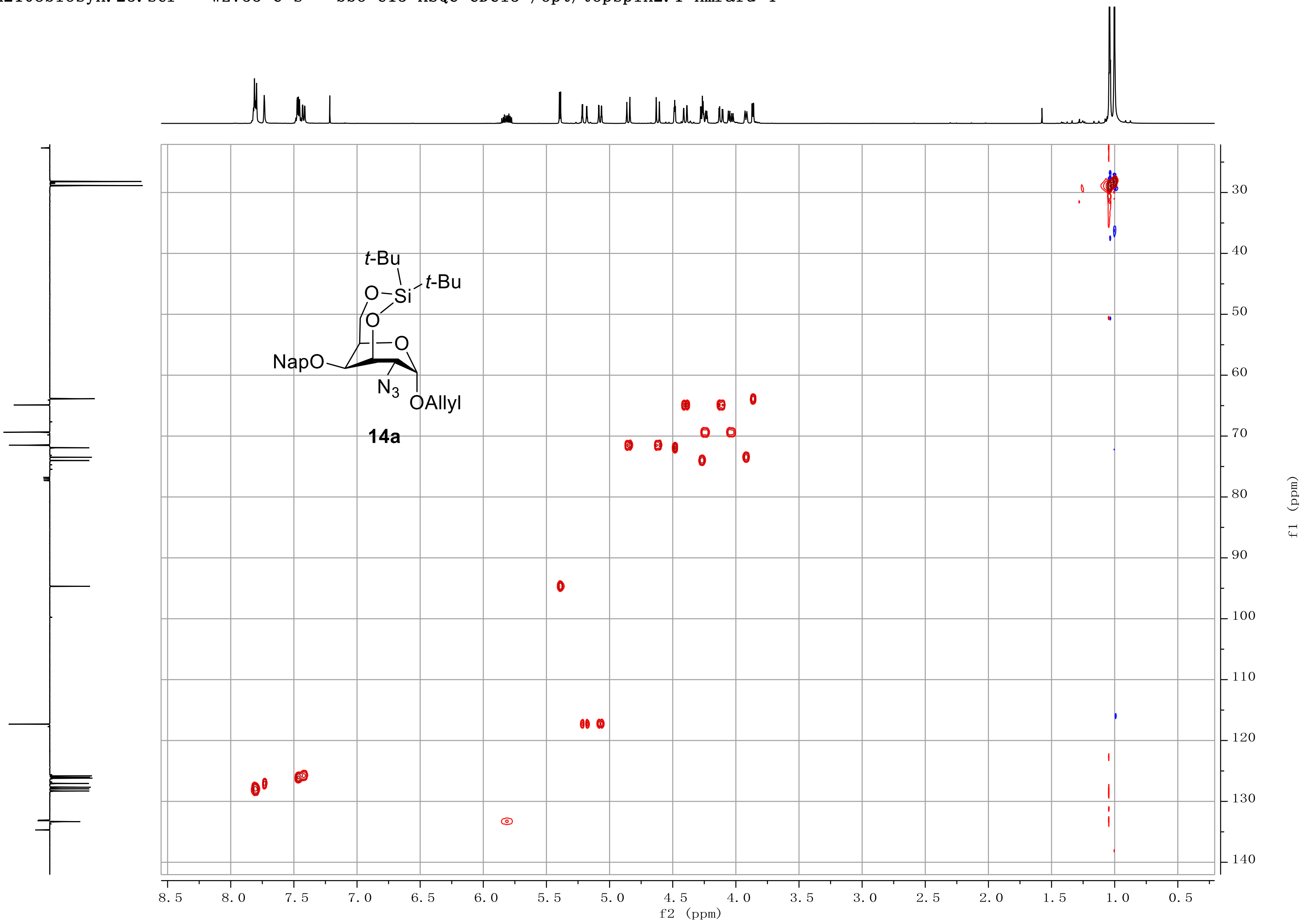

zhen2108biosyn.25.ser — wz753-C-s — bbo-c13-HMBC CDCl3 /opt/topspin2.1 nmrafd 4

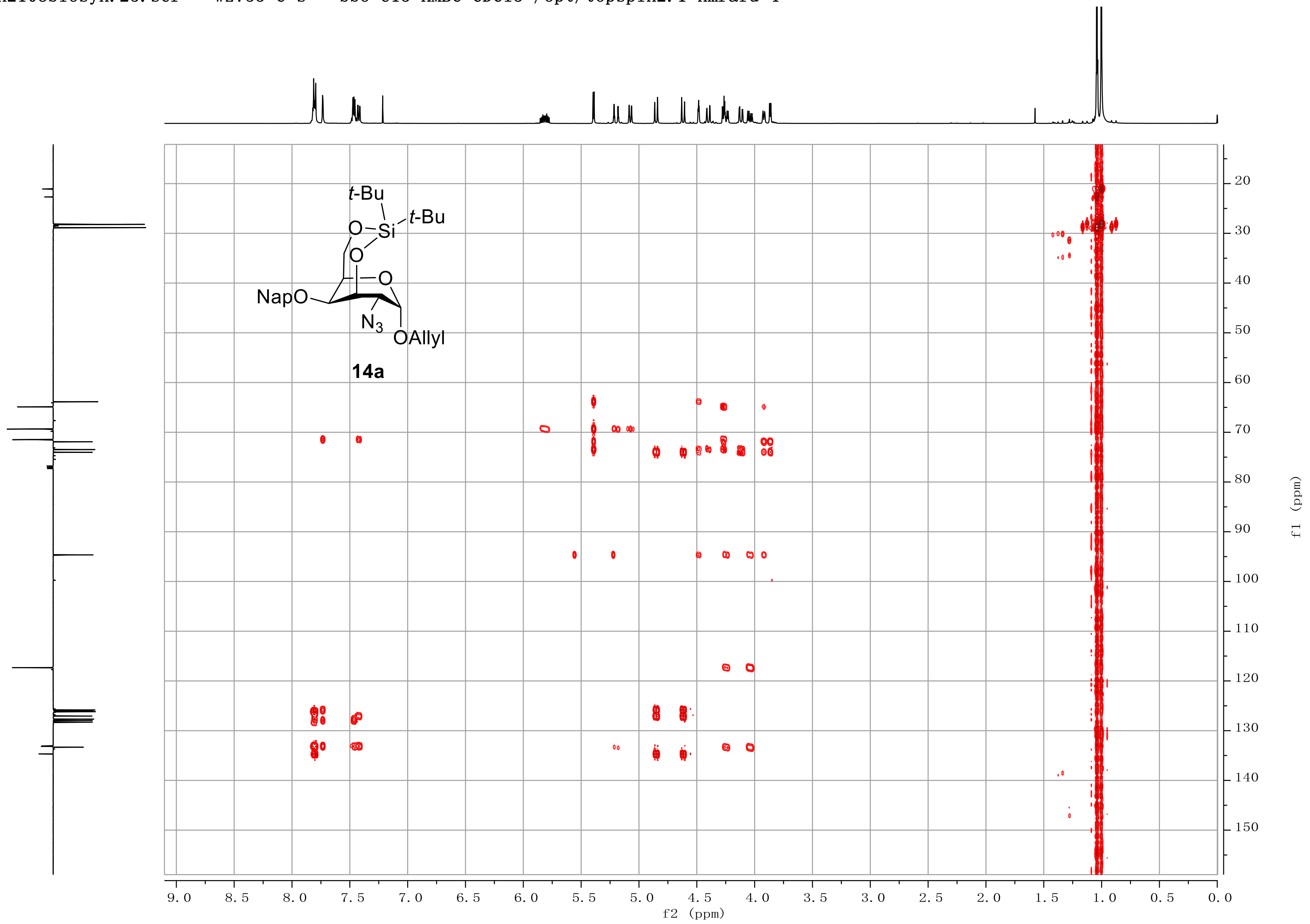

2101zhen.22.fid — wz754 — h1 CDCl3 /opt/DATA nmrafd 6

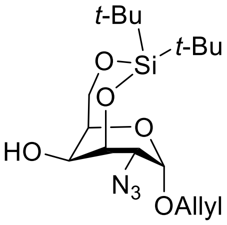

14

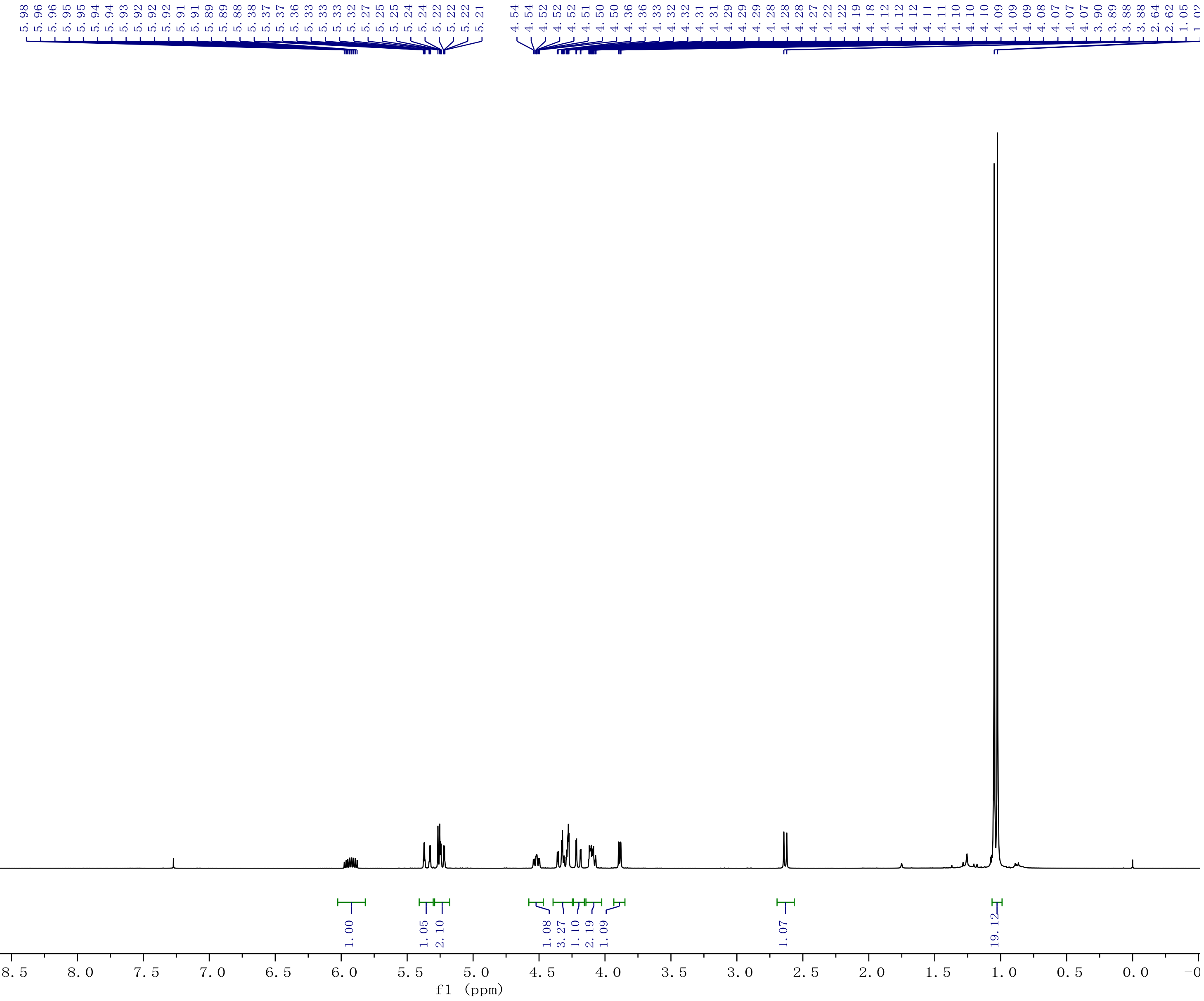

2101zhen.23.fid — wz754 — C13APT CDCl3 /opt/DATA nmrafd 6

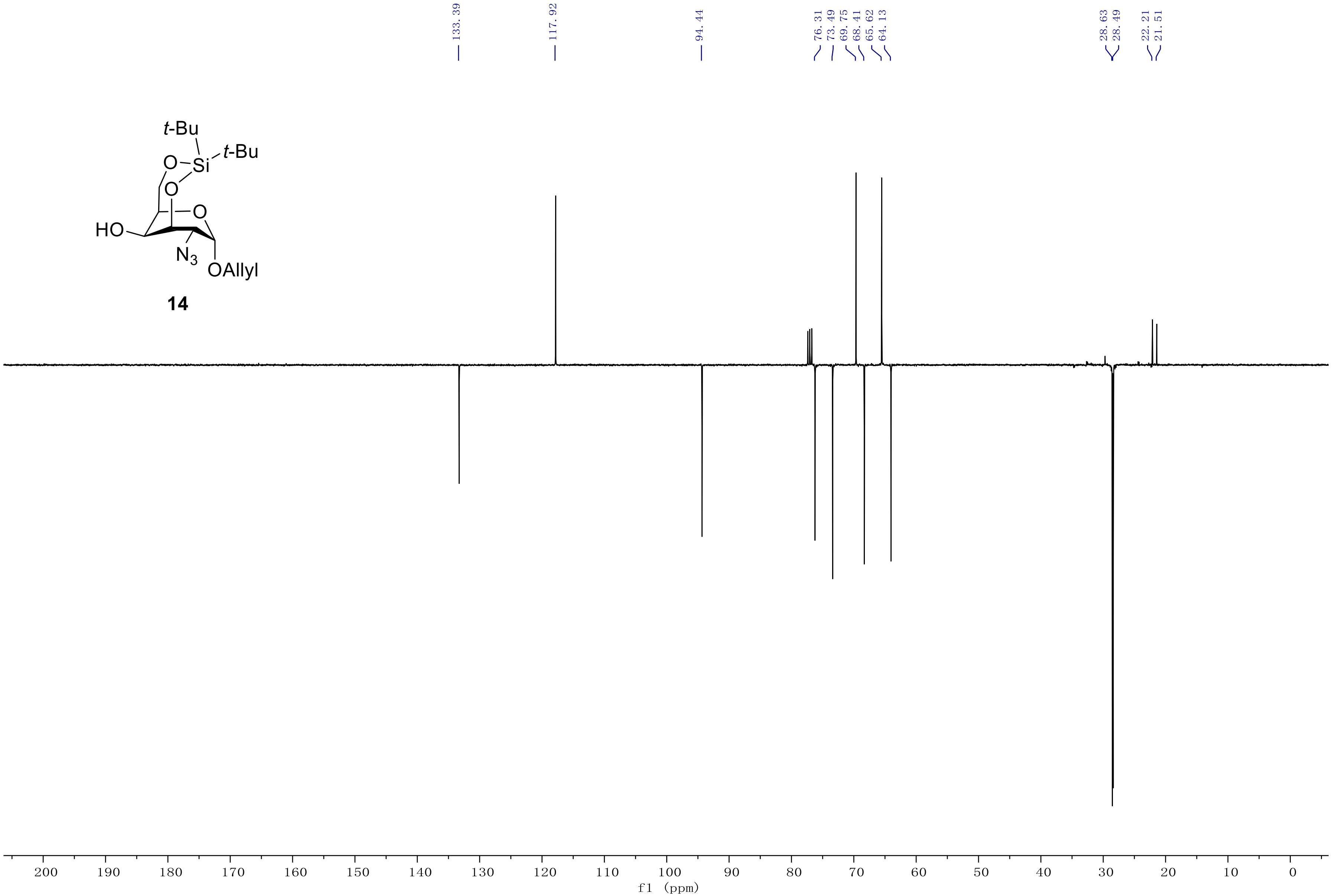

2101zhen.24.ser — wz754 — h1COSY CDCl3 /opt/DATA nmrafd 6

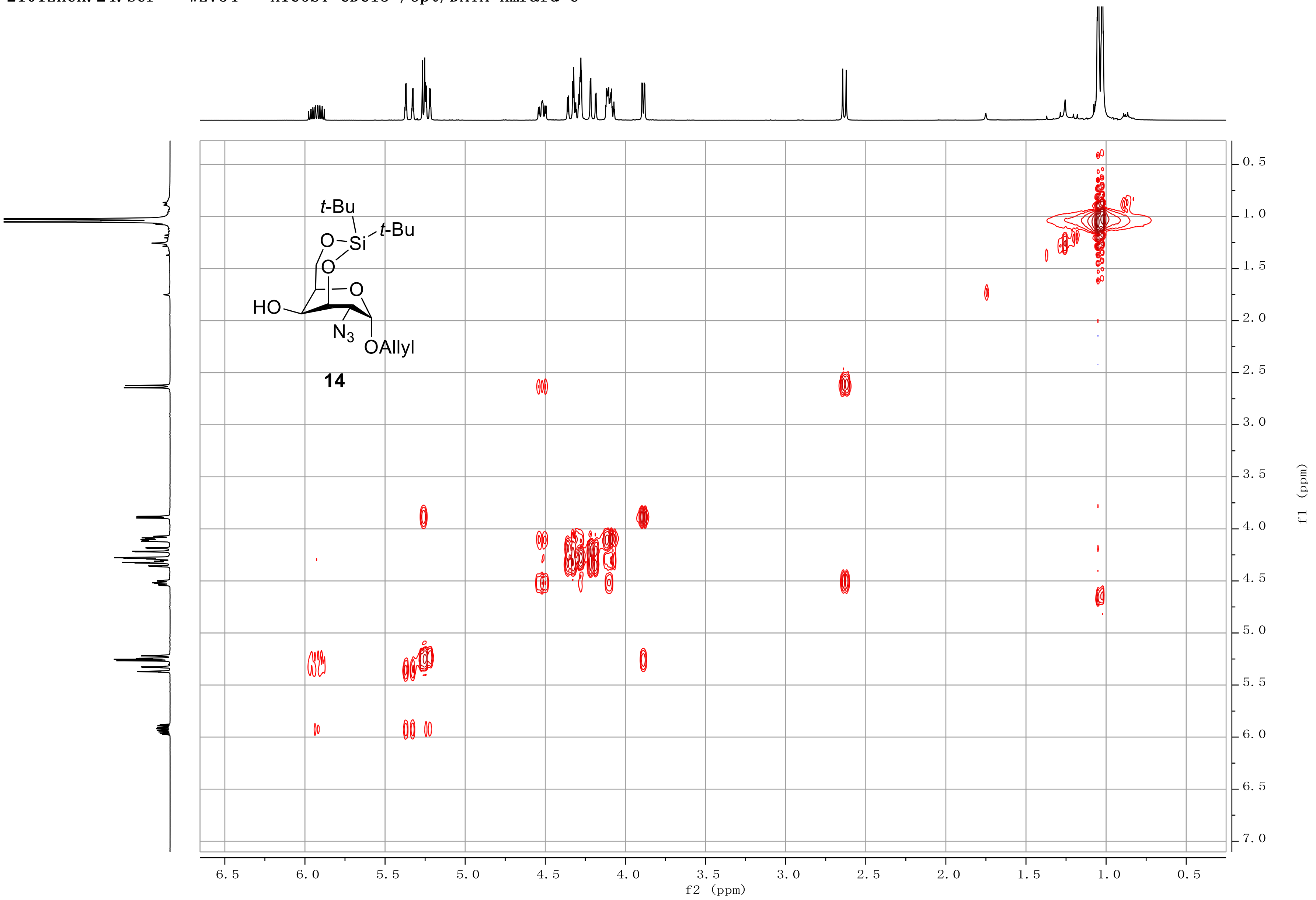

2101zhen.25.ser — wz754 — c13HSQC CDCl3 /opt/DATA nmrafd 6

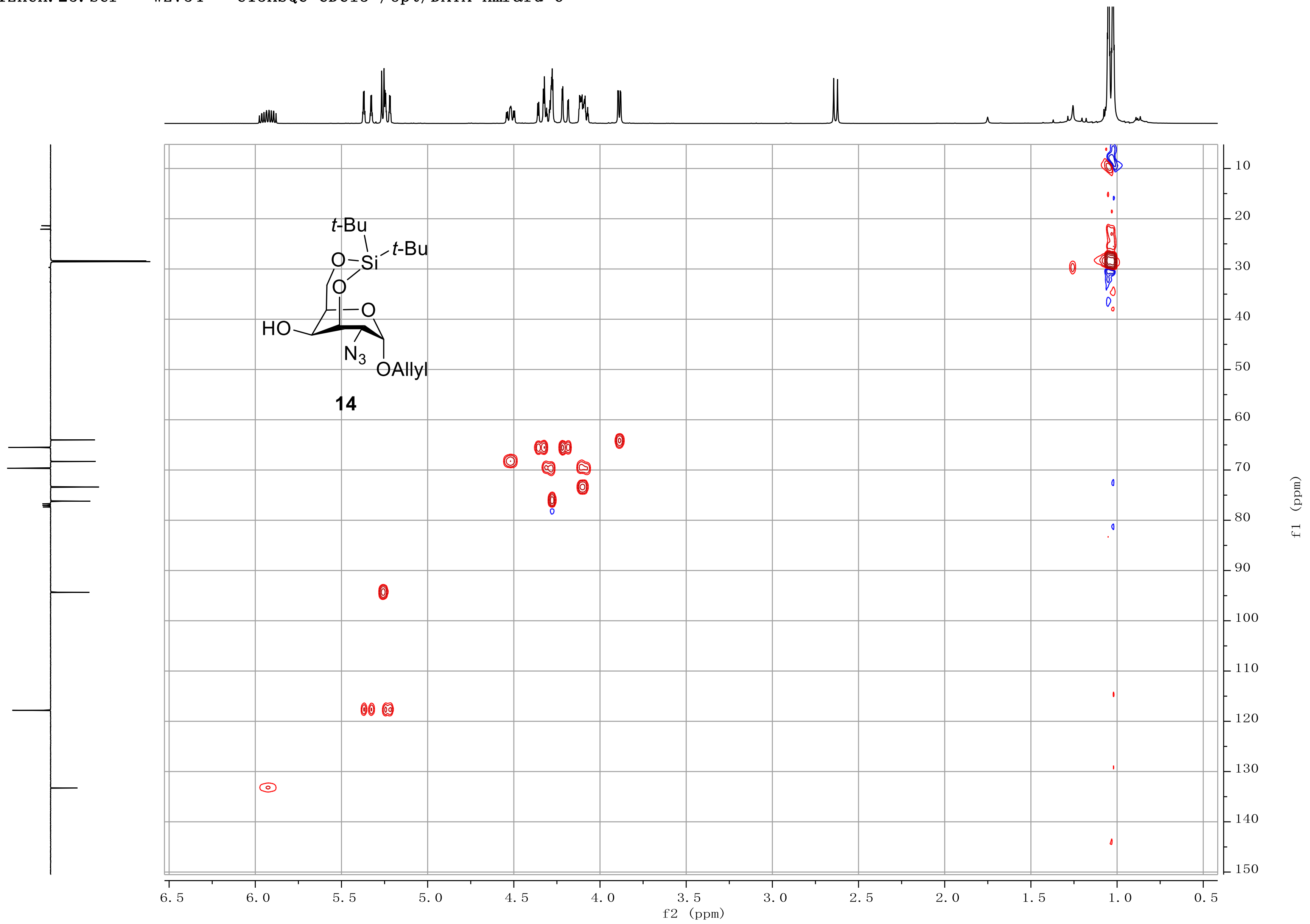

2101zhen.26.ser — wz754 — c13HMBC CDCl3 /opt/DATA nmrafd 6

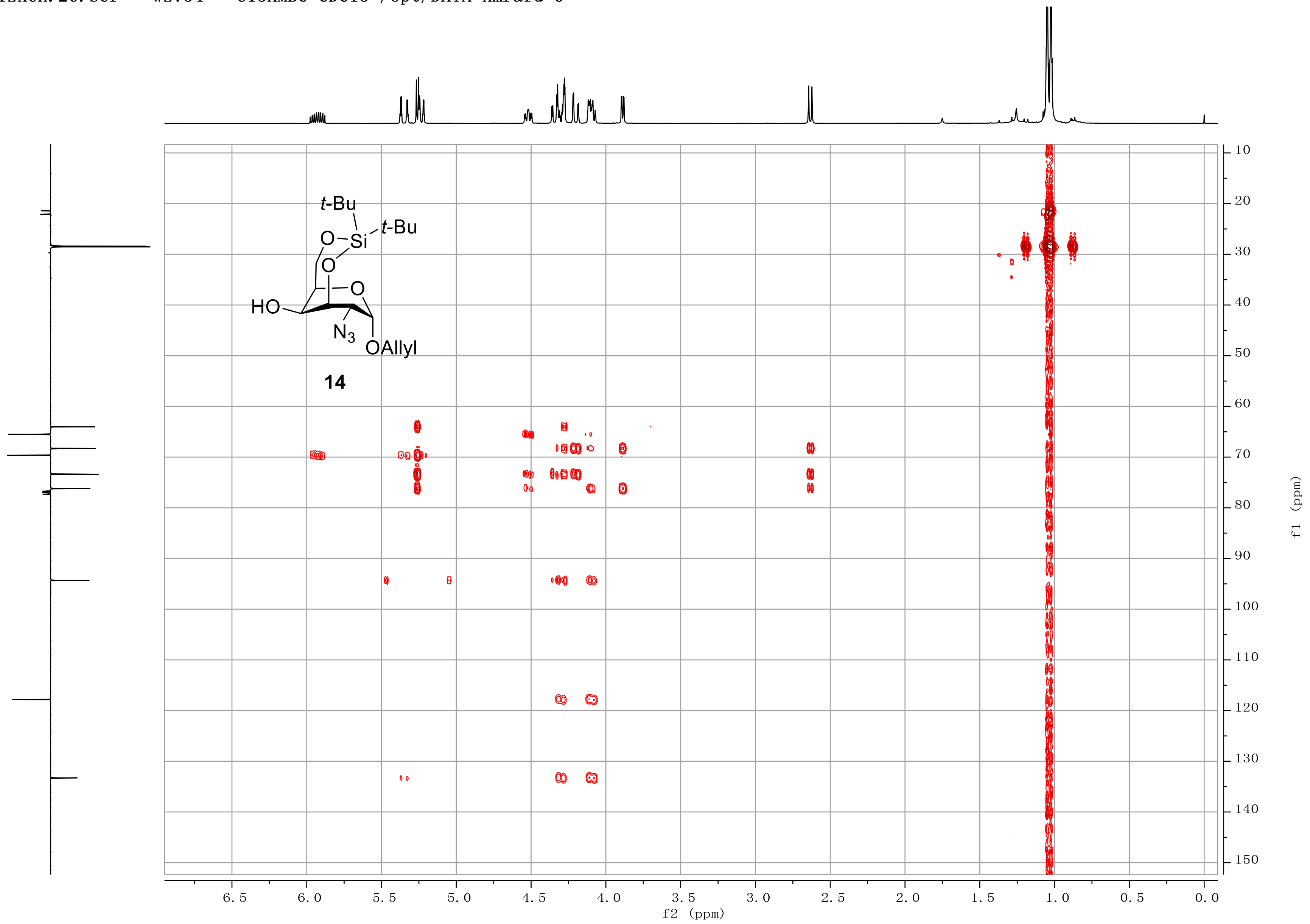

1902Connor/CD85 Proton — CD85 — h1 CDCl3 /opt/DATA nmrafd 12

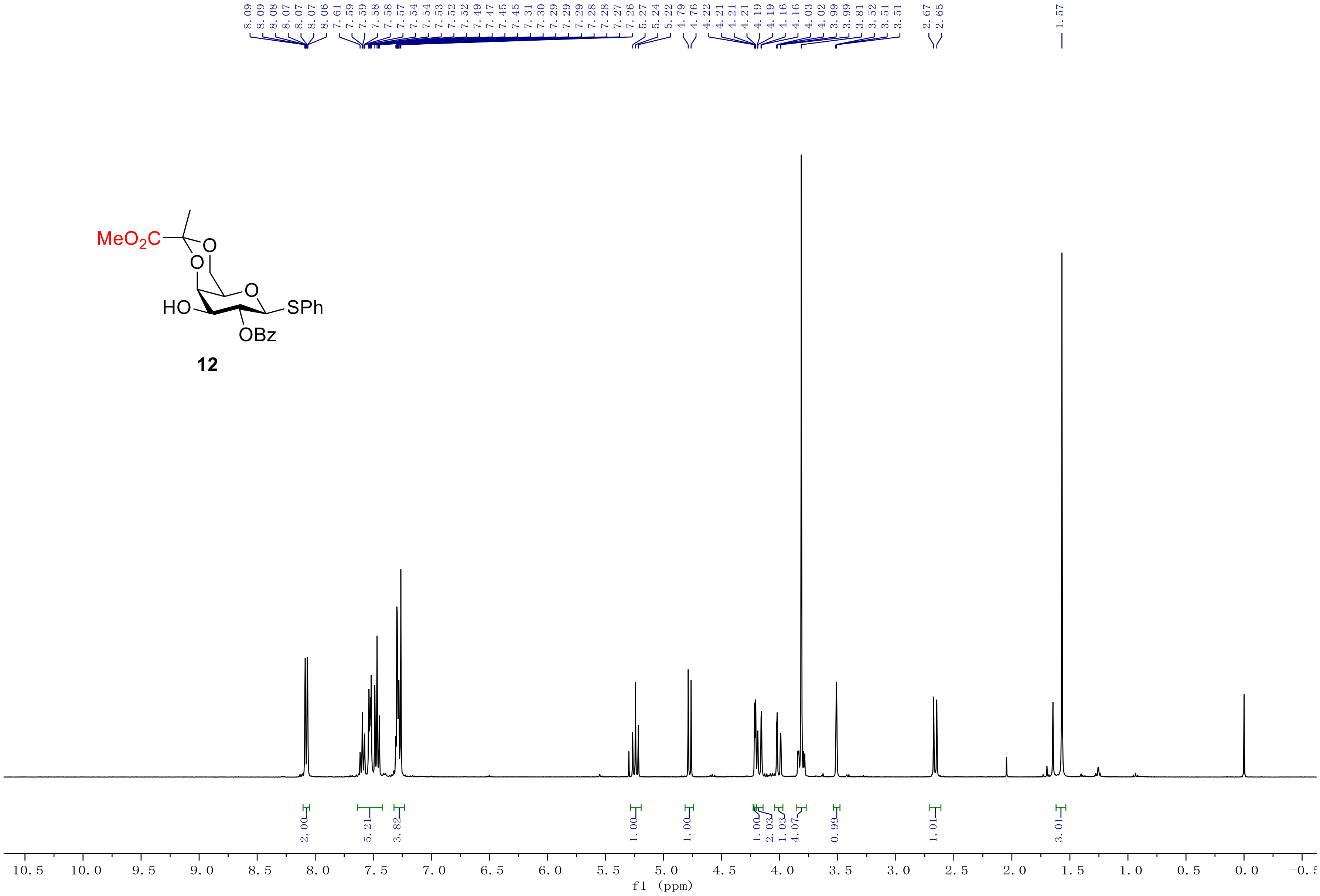

12

1902Connor/CD85 Carbon — CD85 — C13APT CDCl3 /opt/DATA nmrafd 12

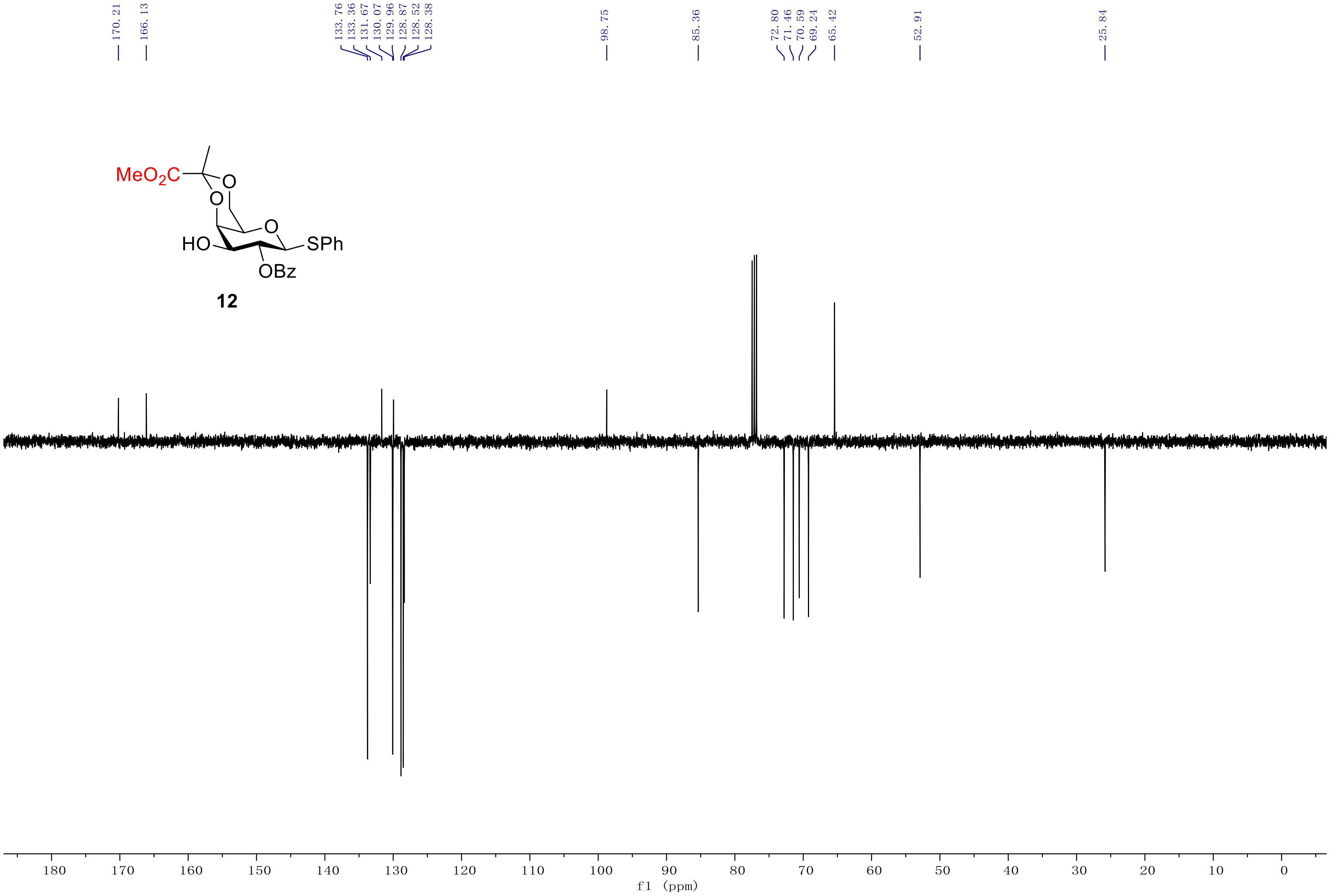

1902Connor/CD85 COSY — CD85 — h1COSY CDCl3 /opt/DATA nmrafd 12

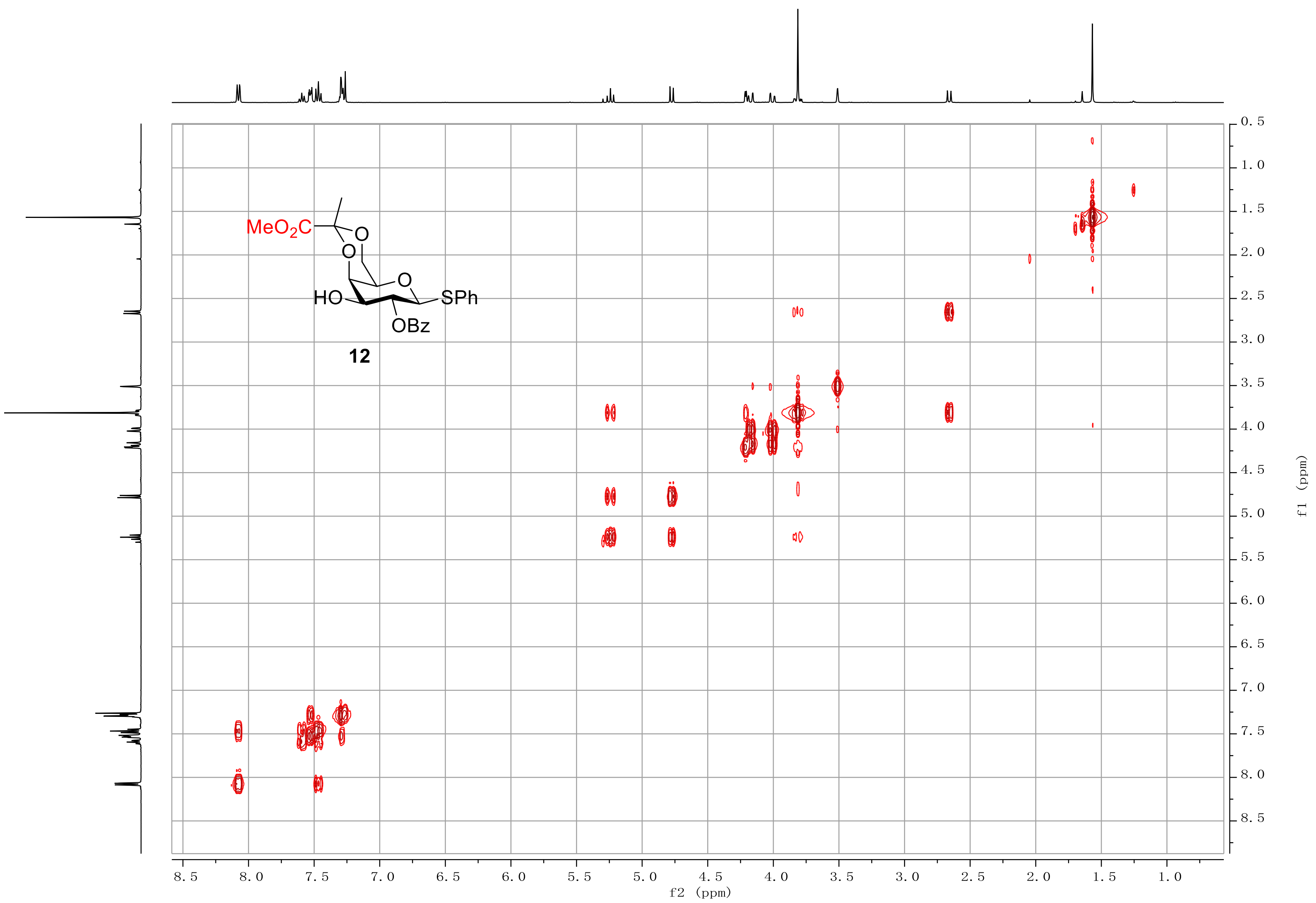

1902Connor/CD85 HSQC — CD85 — c13HSQC CDCl3 /opt/DATA nmrafd 12

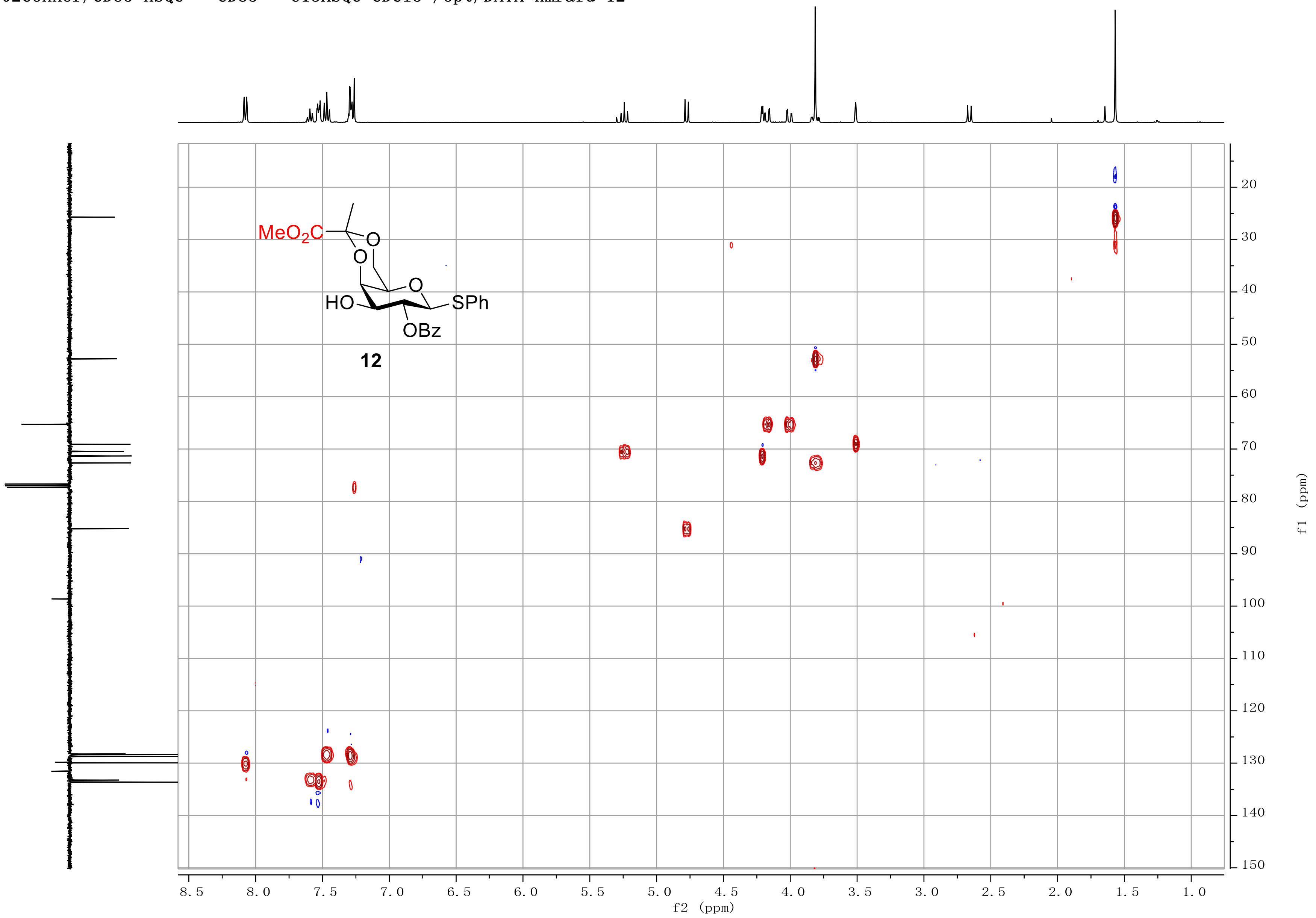

Qingju1808Biosyn.9.fid — 0744 — bbo-h1 Acetone /opt/topspin2.1 nmrafd 6

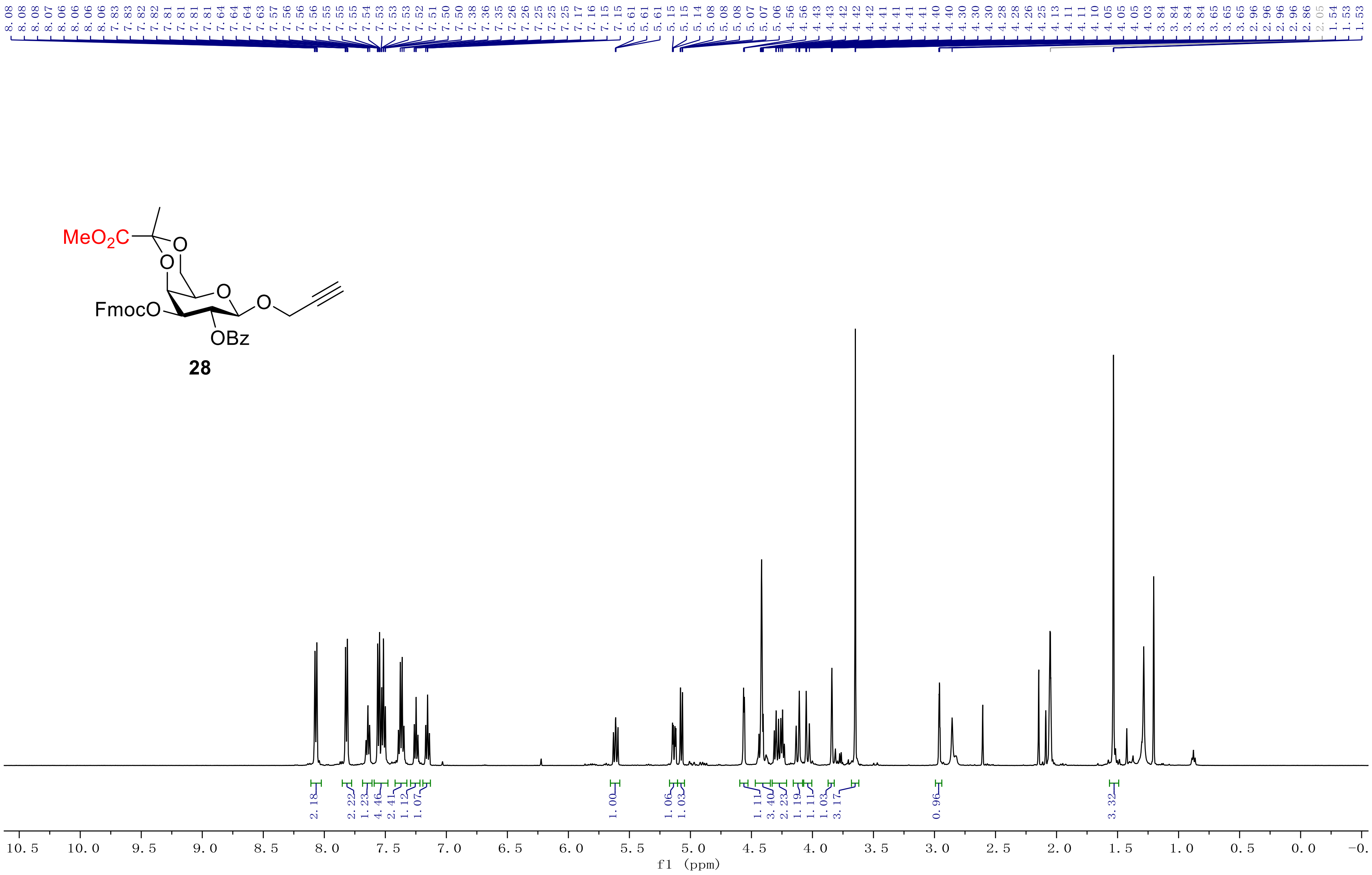

28

Qingju1808Biosyn.12.fid — 0744 — bbo-c13-APT Acetone /opt/topspin2.1 nmrafd 6

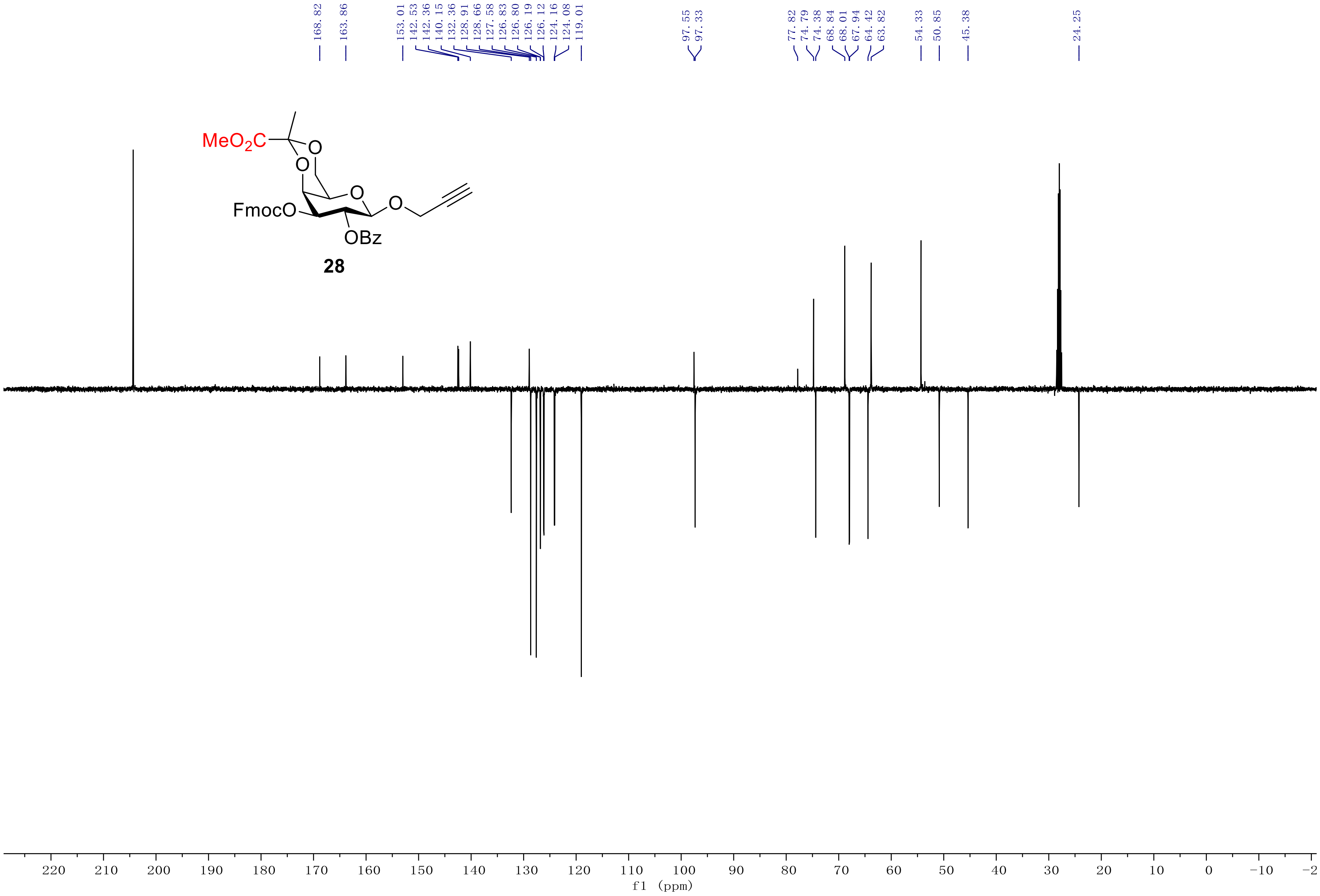

Qingju1808Biosyn.10.ser — 0744 — bbo-h1-cosy Acetone /opt/topspin2.1 nmrafd 6

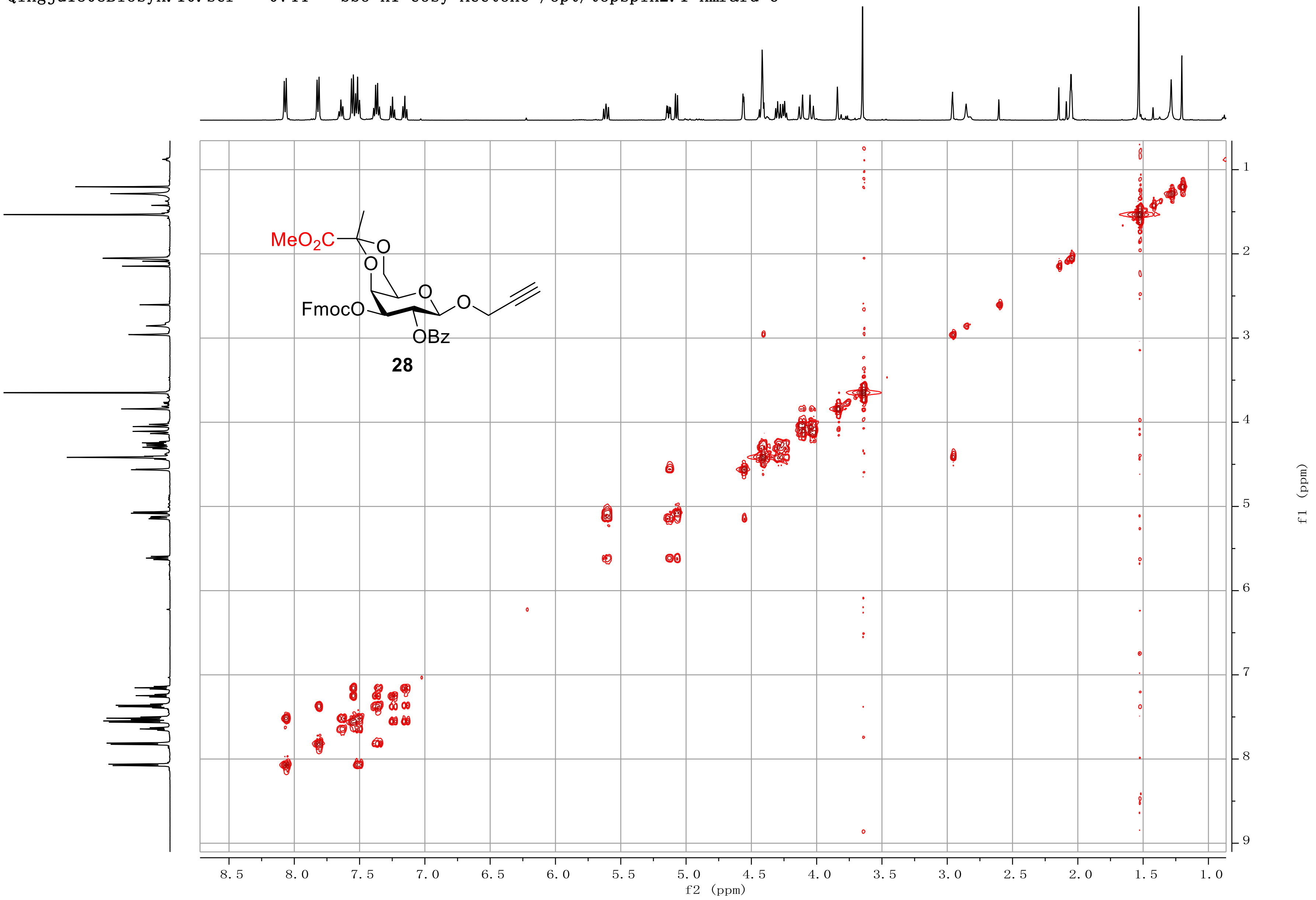

Qingju1808Biosyn.11.ser — 0744 — bbo-c13-HSQC Acetone /opt/topspin2.1 nmrafd 6

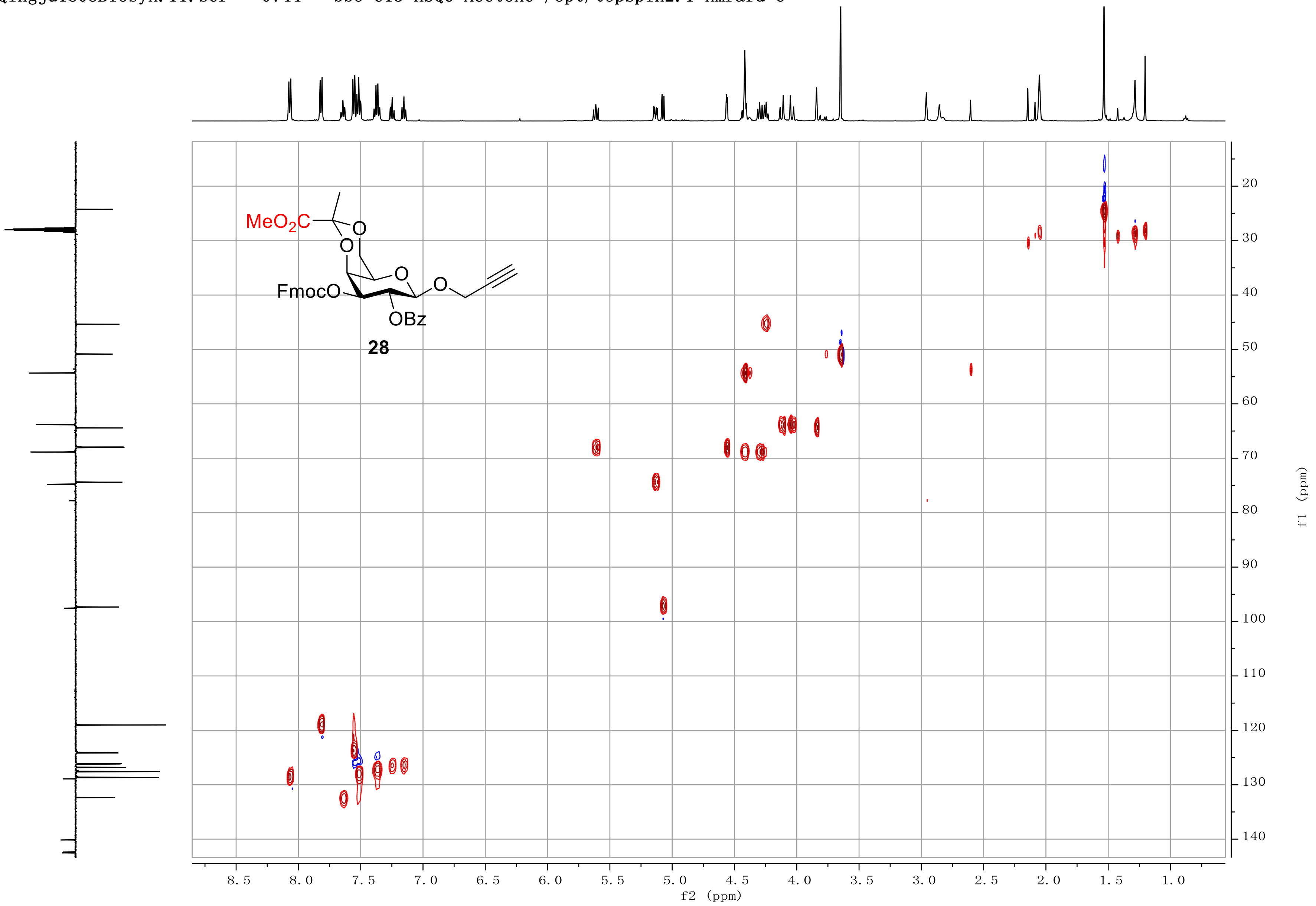

1808Qingju.14.fid — 0746 — h1 Acetone /opt/DATA nmrafd 18

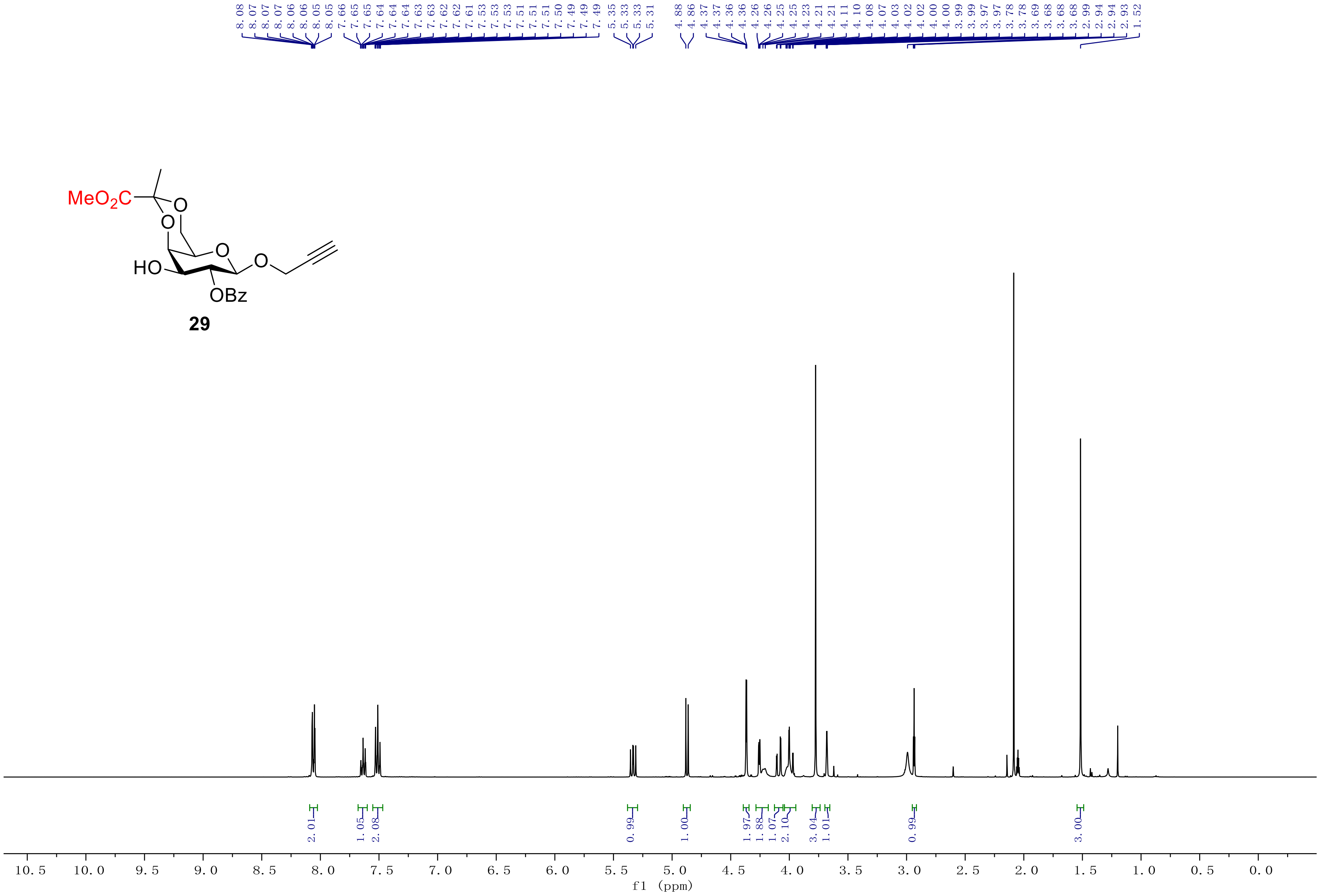

29

1808Qingju.15.fid — 0746 — C13APT Acetone /opt/DATA nmrafd 18

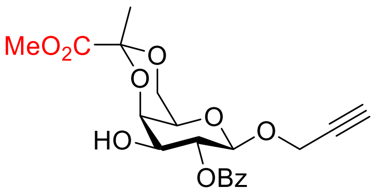

29

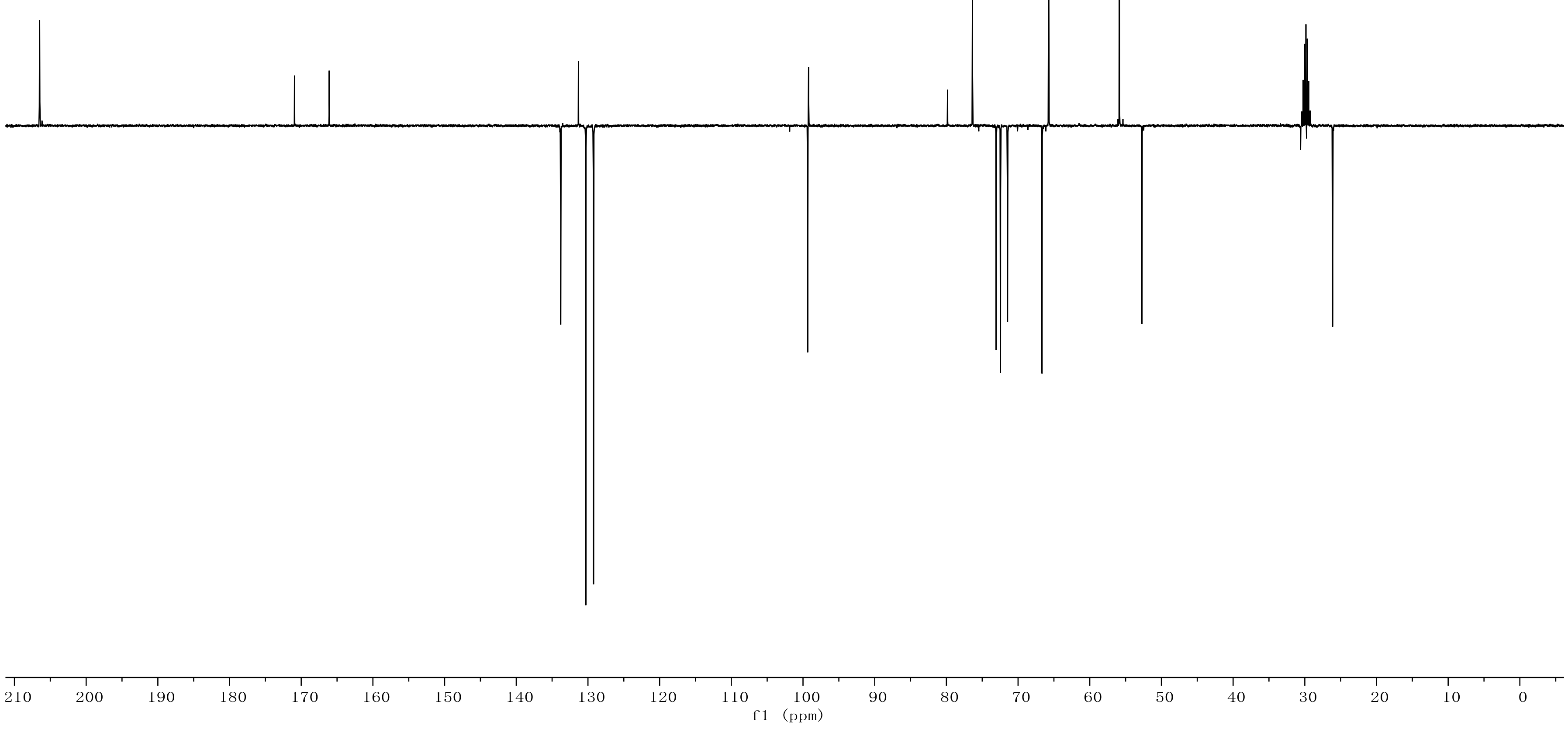

1808Qingju.16.ser — 0746 — h1COSY Acetone /opt/DATA nmrafd 18

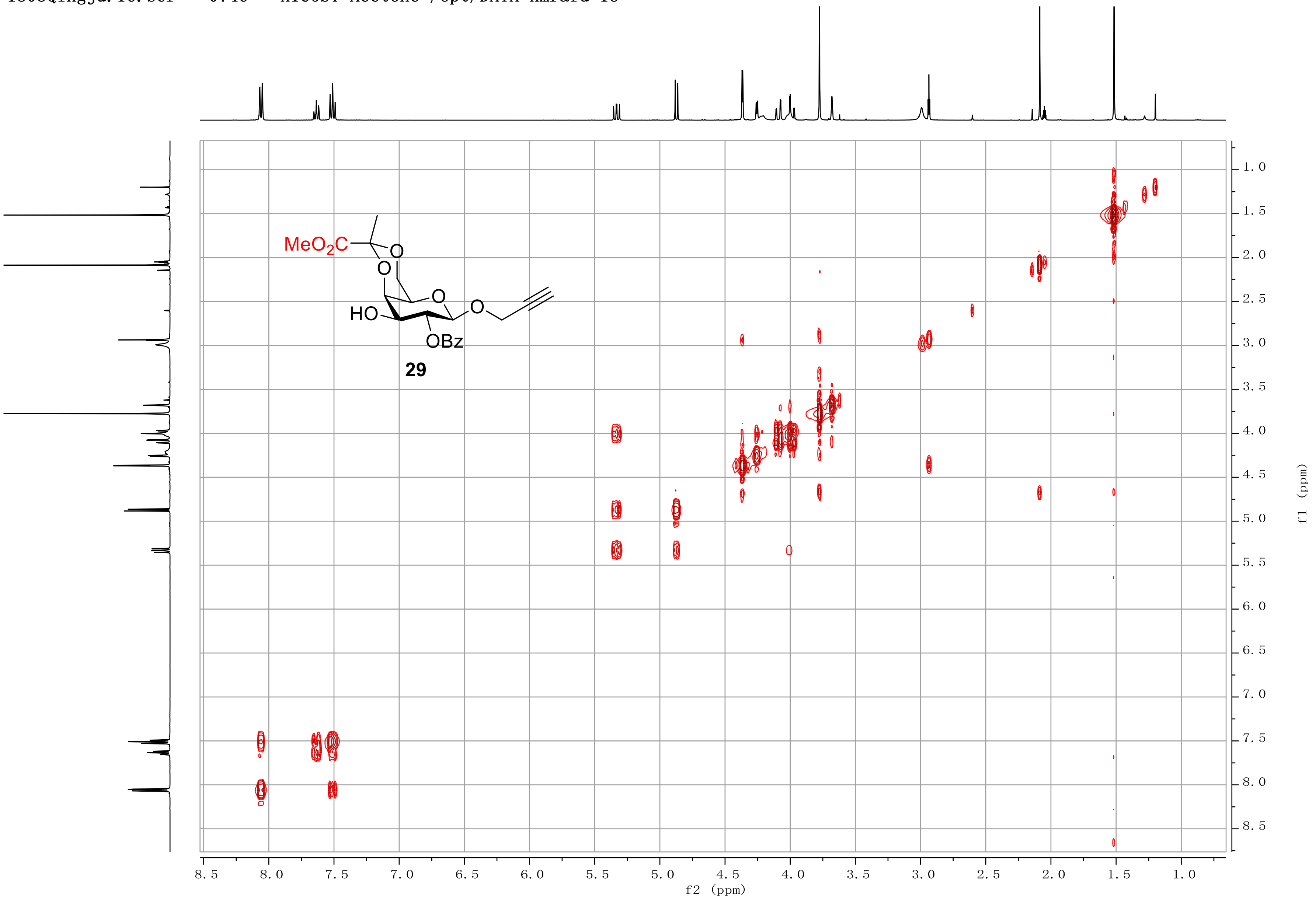

1808Qingju.17.ser — 0746 — c13HSQC Acetone /opt/DATA nmrafd 18

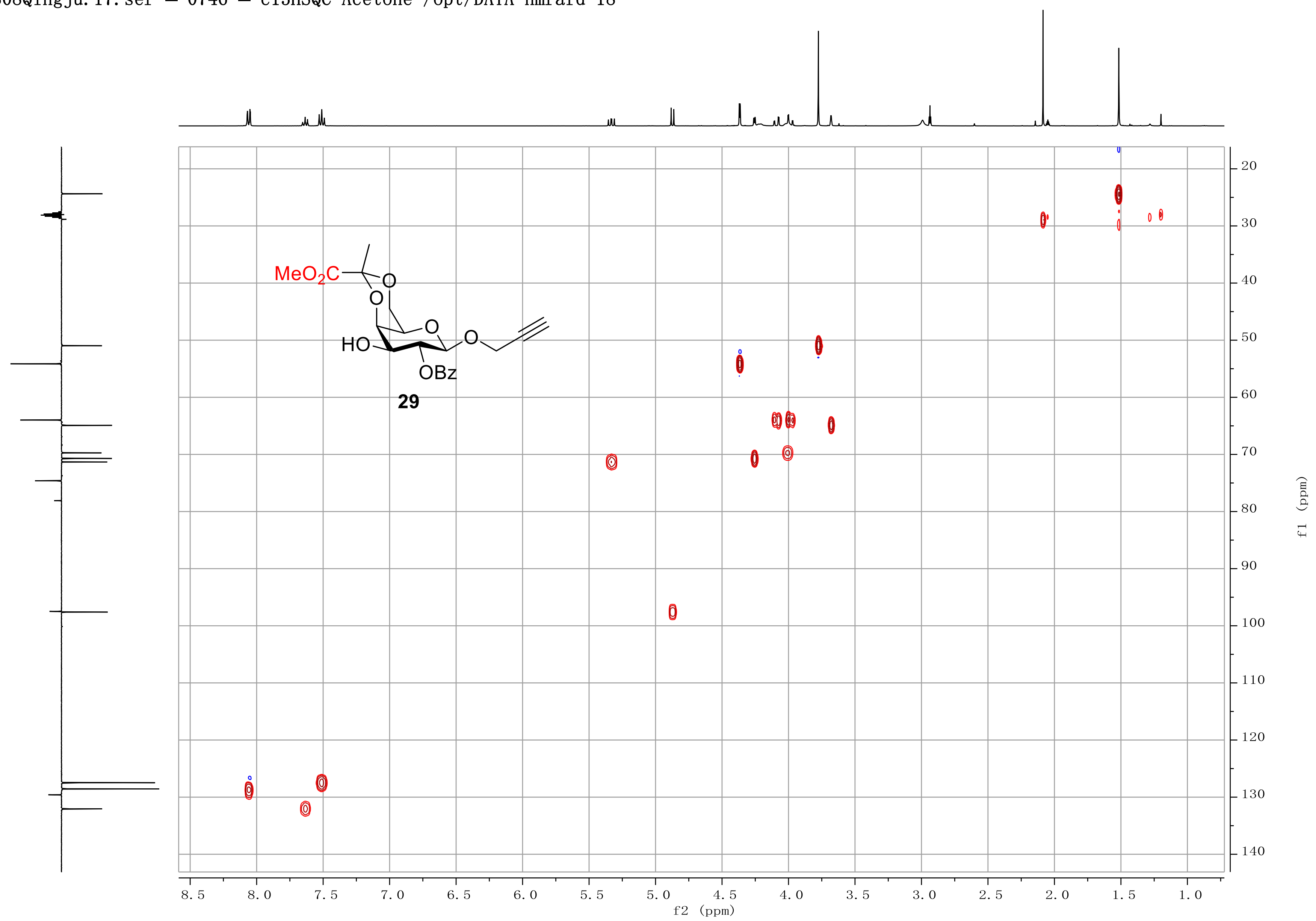

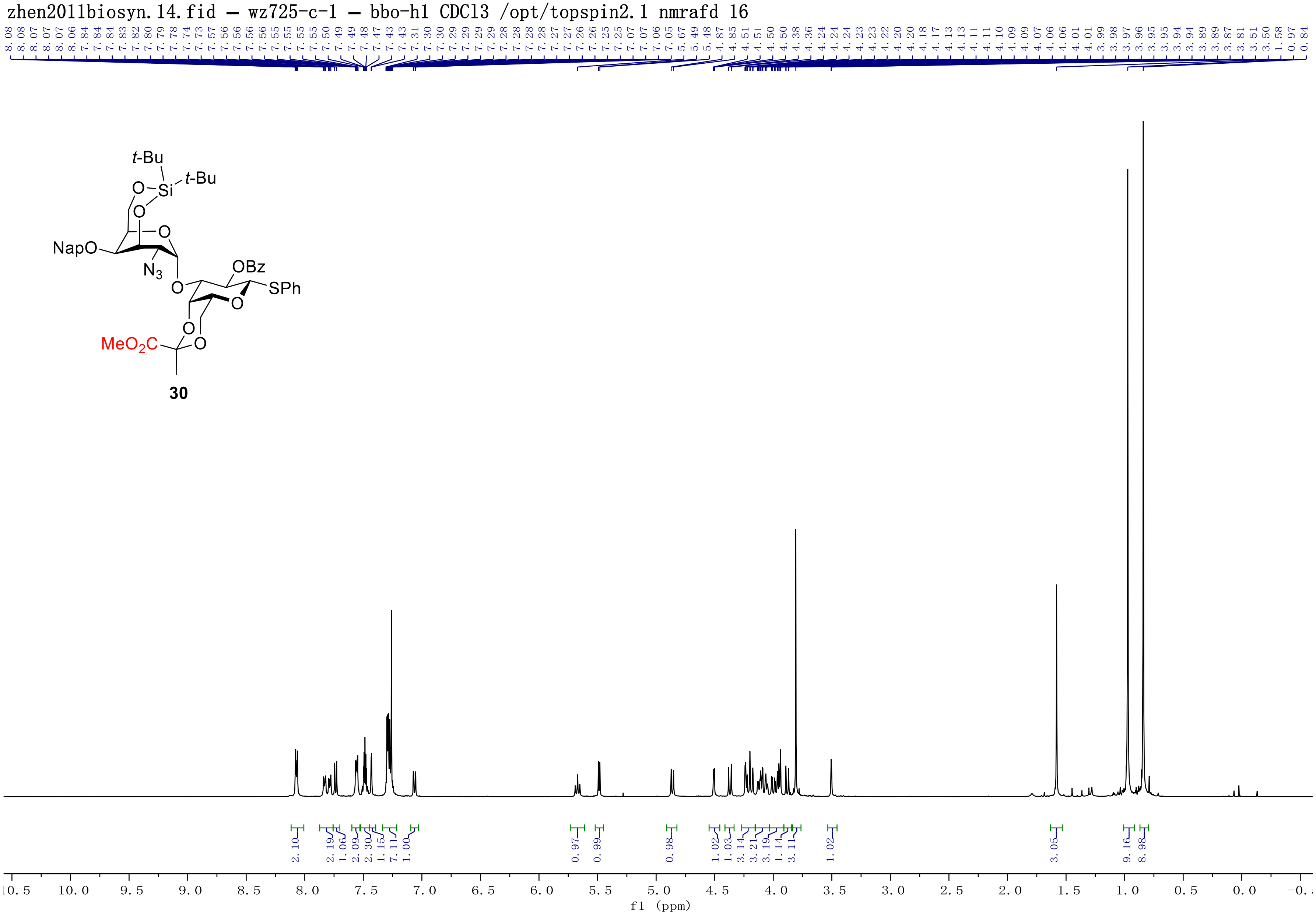
zhen2011biosyn.14.fid — wz725-c-1 — bbo-h1 CDCl3 /opt/topspin2.1 nmrafd 16
8.08 8.08 8.07 8.07 8.06 7.84 7.84 7.84 7.83 7.82 7.80 7.79 7.78 7.74 7.73 7.57 7.56 7.56 7.56 7.55 7.55 7.55 7.55 7.50 7.49 7.49 7.48 7.47 7.43 7.43 7.31 7.30 7.29 7.29 7.29 7.29 7.28 7.28 7.28 7.28 7.27 7.27 7.26 7.26 7.25 7.25 7.07 7.07 7.06 7.05 5.67 5.49 5.48 4.87 4.85 4.51 4.51 4.50 4.50 4.38 4.36 4.24 4.24 4.24 4.23 4.23 4.22 4.20 4.18 4.17 4.13 4.13 4.11 4.11 4.10 4.09 4.09 4.07 4.06 4.06 4.01 4.01 3.99 3.98 3.97 3.96 3.95 3.95 3.94 3.94 3.89 3.87 3.81 3.51 3.50 1.58 0.97 0.84
t-Bu
t-Bu
O
Si
O
O
NapO
N3
OBz
O
SPh
O
O
O
MeO2C
O
30
2.10 2.19 1.06 2.09 2.30 1.15 7.11 1.00 0.97 0.99 0.98 1.02 1.03 3.14 3.21 3.19 1.14 3.11 1.02 3.05 9.16 8.98
10.5 10.0 9.5 9.0 8.5 8.0 7.5 7.0 6.5 6.0 5.5 5.0 4.5 4.0 3.5 3.0 2.5 2.0 1.5 1.0 0.5 0.0 -0.
f1 (ppm)

zhen2011biosyn.17.fid — wz725-c-1 — bbo-c13-APT CDCl3 /opt/topspin2.1 nmrafd 16

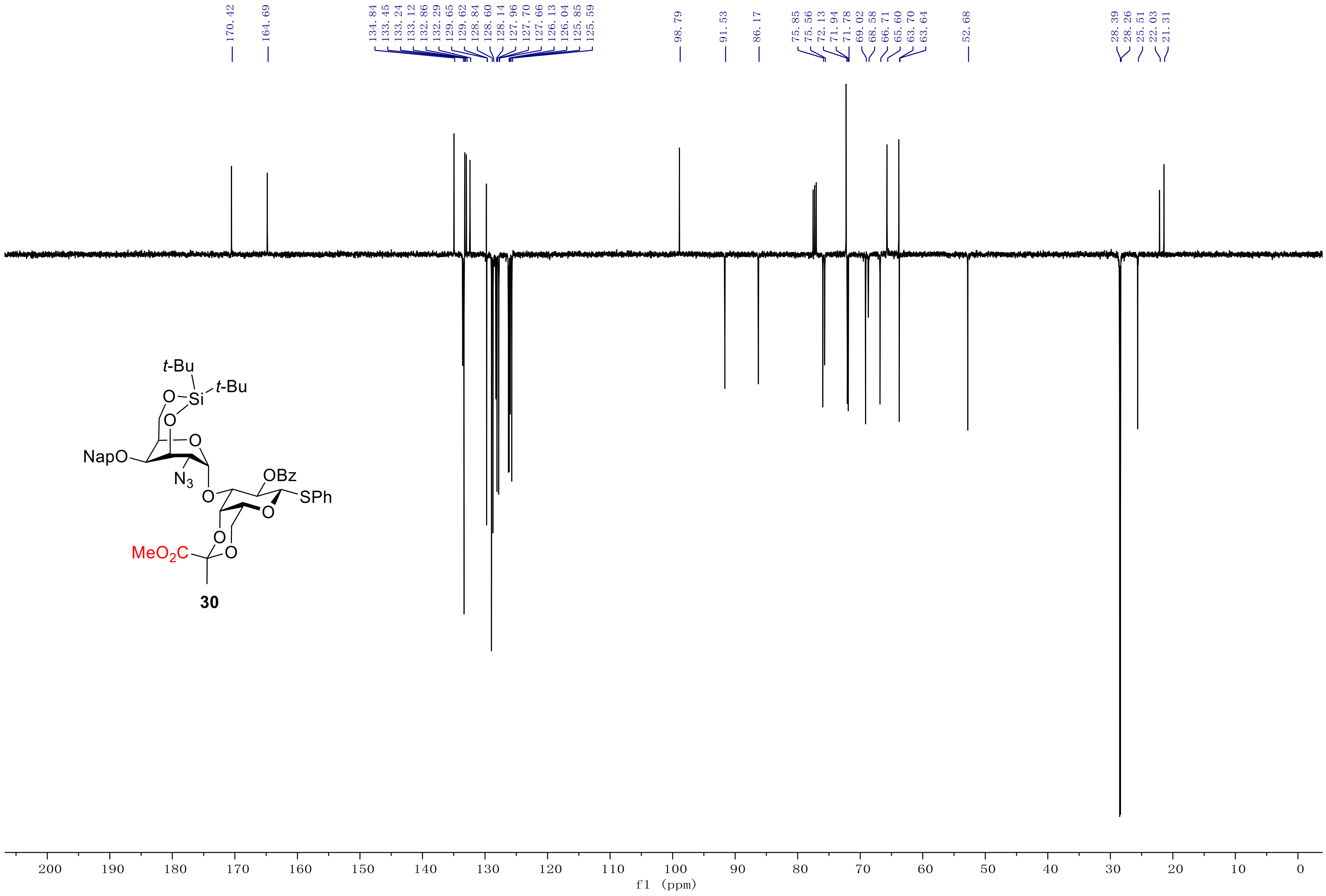

30

zhen2011biosyn.15.ser — wz725-c-1 — bbo-h1-cosy CDCl3 /opt/topspin2.1 nmrafd 16

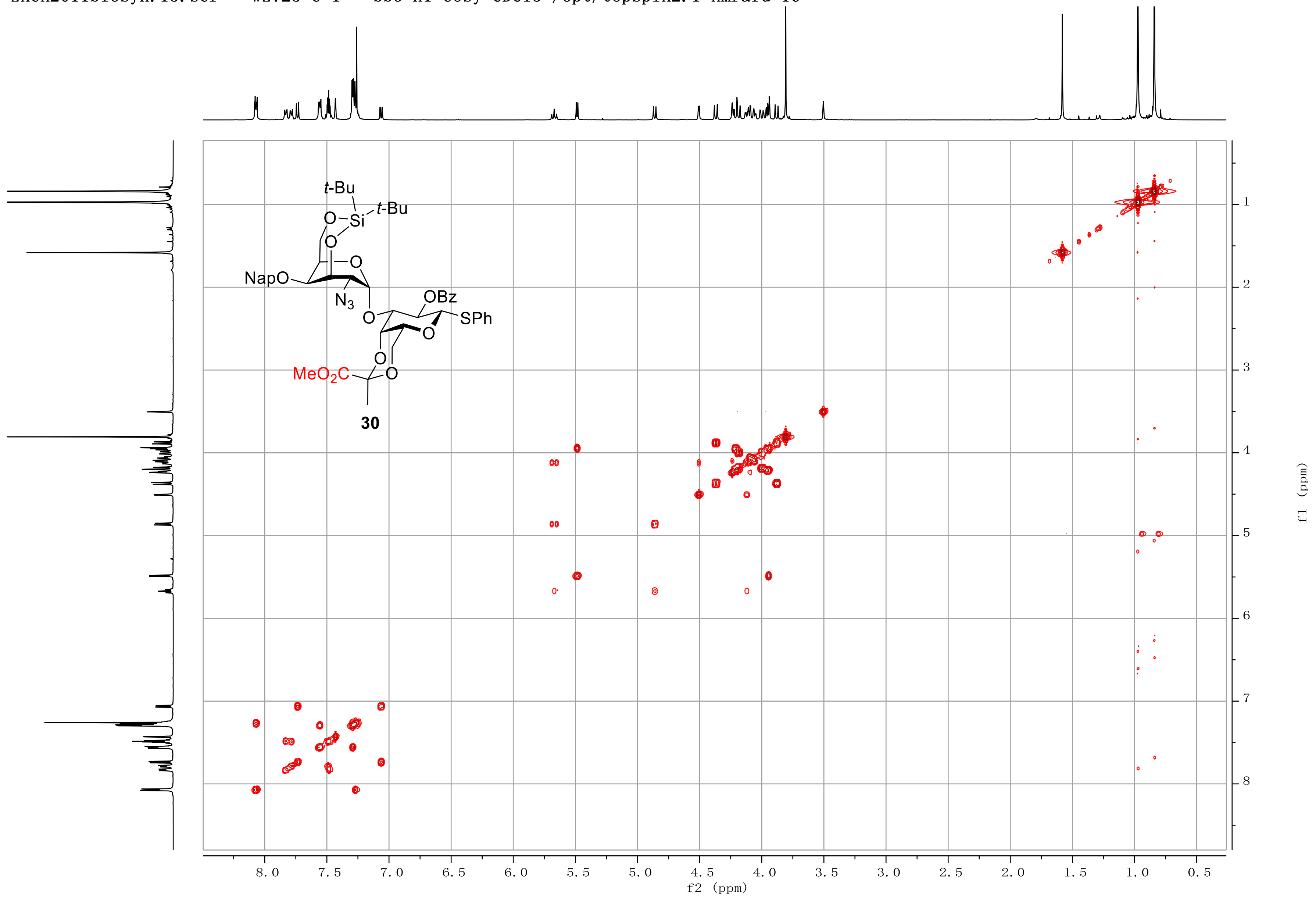

zhen2011biosyn.16.ser — wz725-c-1 — bbo-c13-HSQC CDCl3 /opt/topspin2.1 nmrafd 16

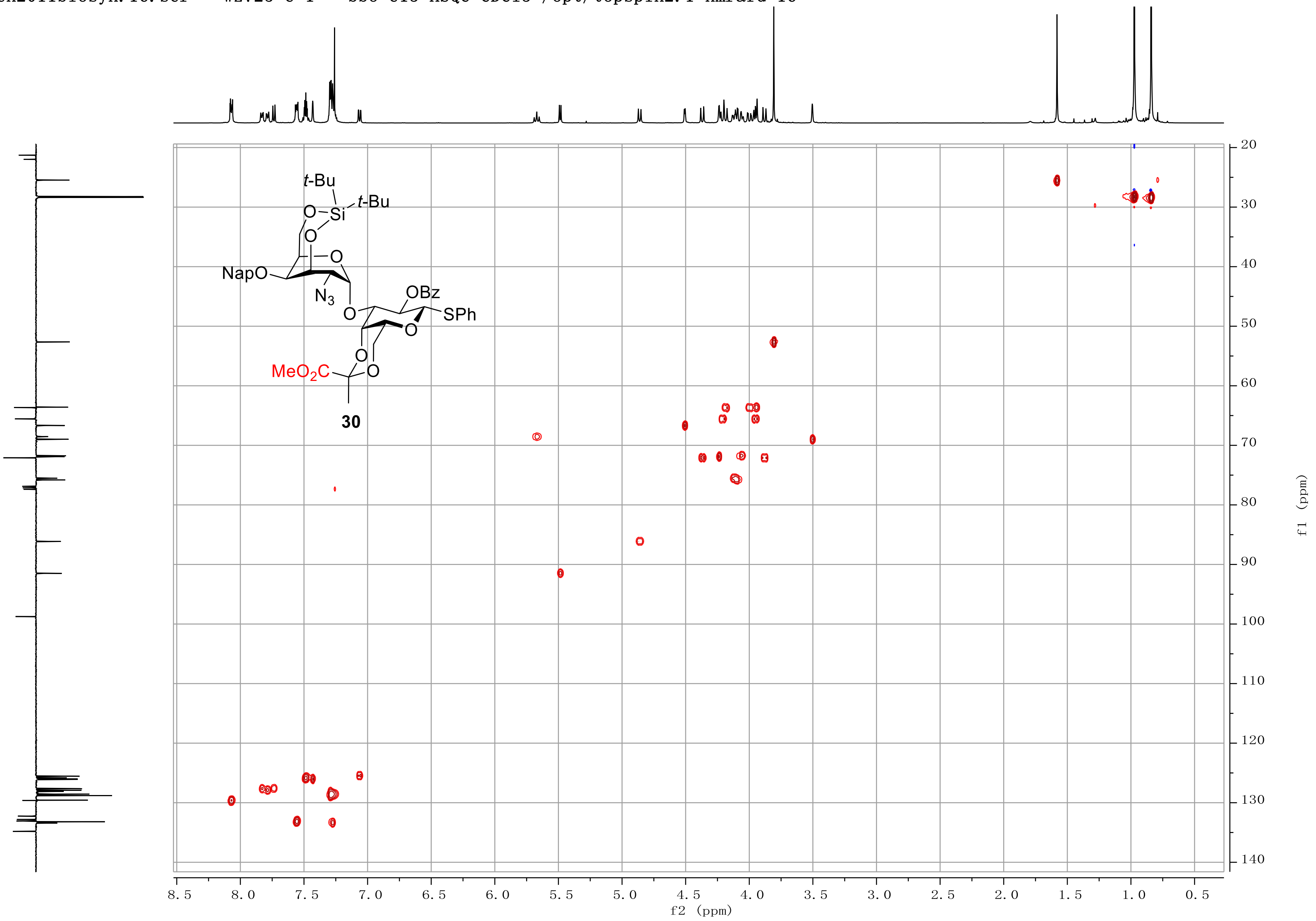

zhen2011biosyn.18.ser — wz725-c-1 — bbo-c13-HMBC CDCl3 /opt/topspin2.1 nmrafd 16

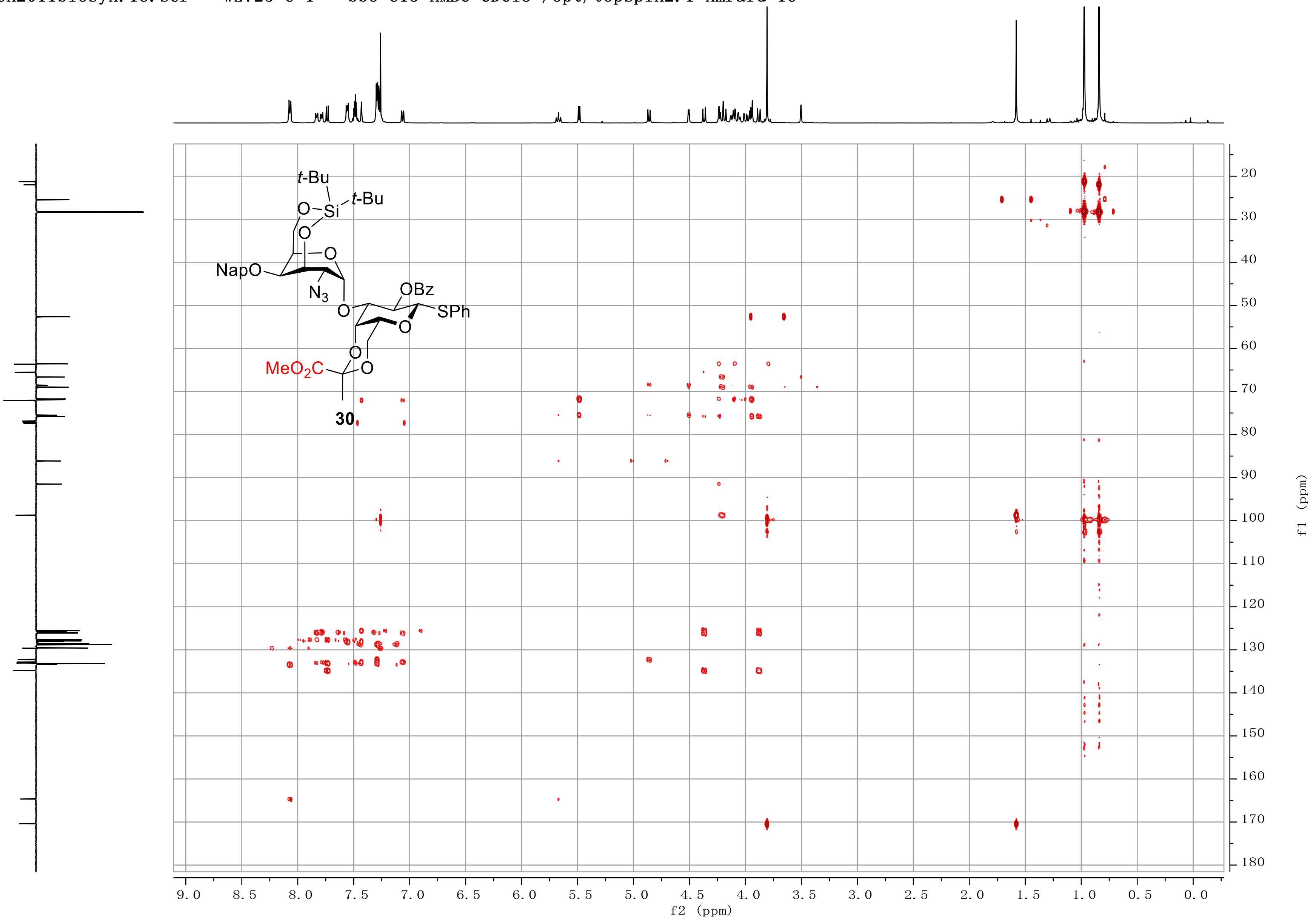

zhen2011biosyn.19.ser — wz725-c-1 — bbo-c13-hmbc-ipv-gated CDCl3 /opt/topspin2.1 nmrafd 16

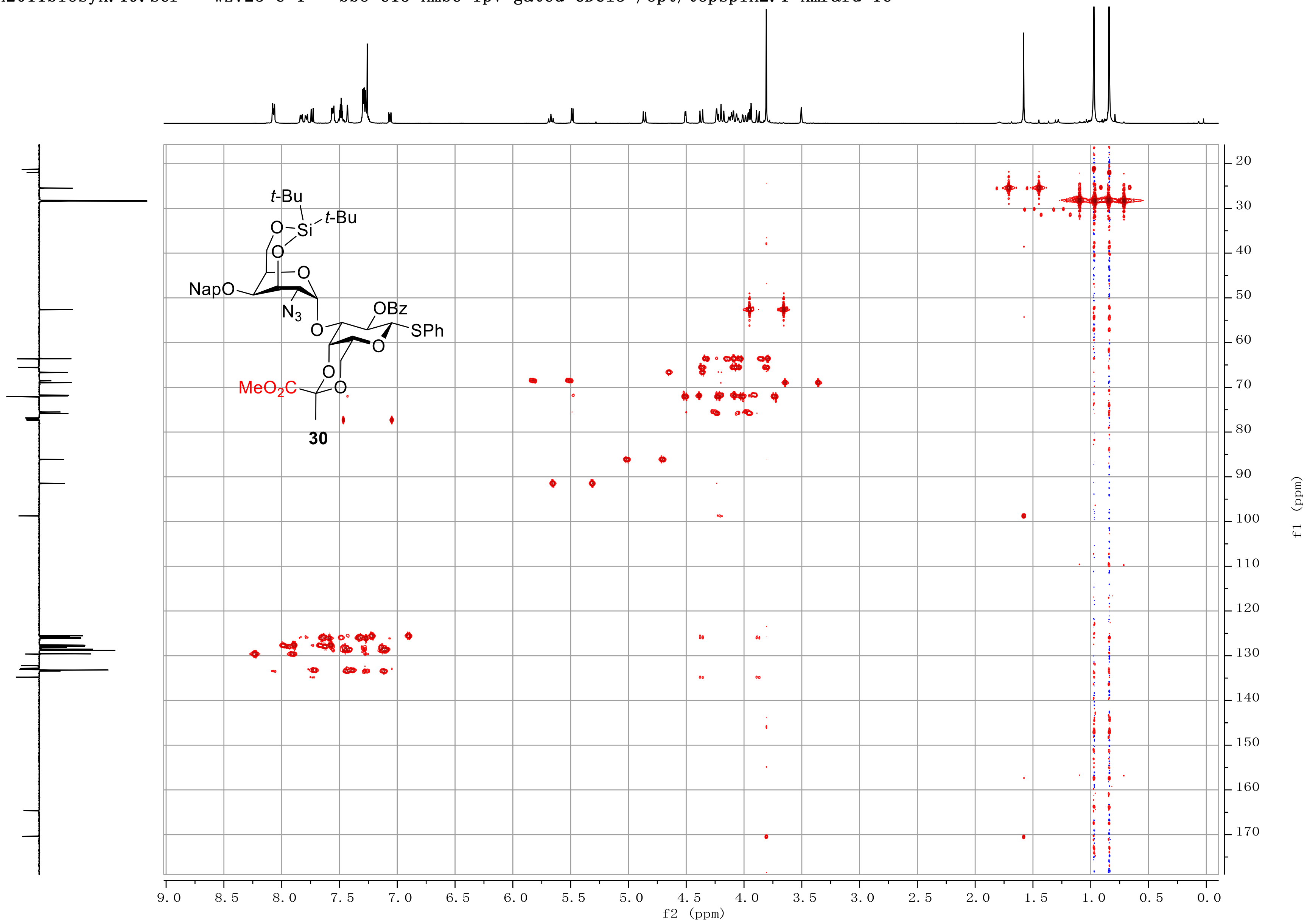

2011zhen.52.fid — wz733-c-1-1 — h1 CDCl3 /opt/DATA nmrafd 18

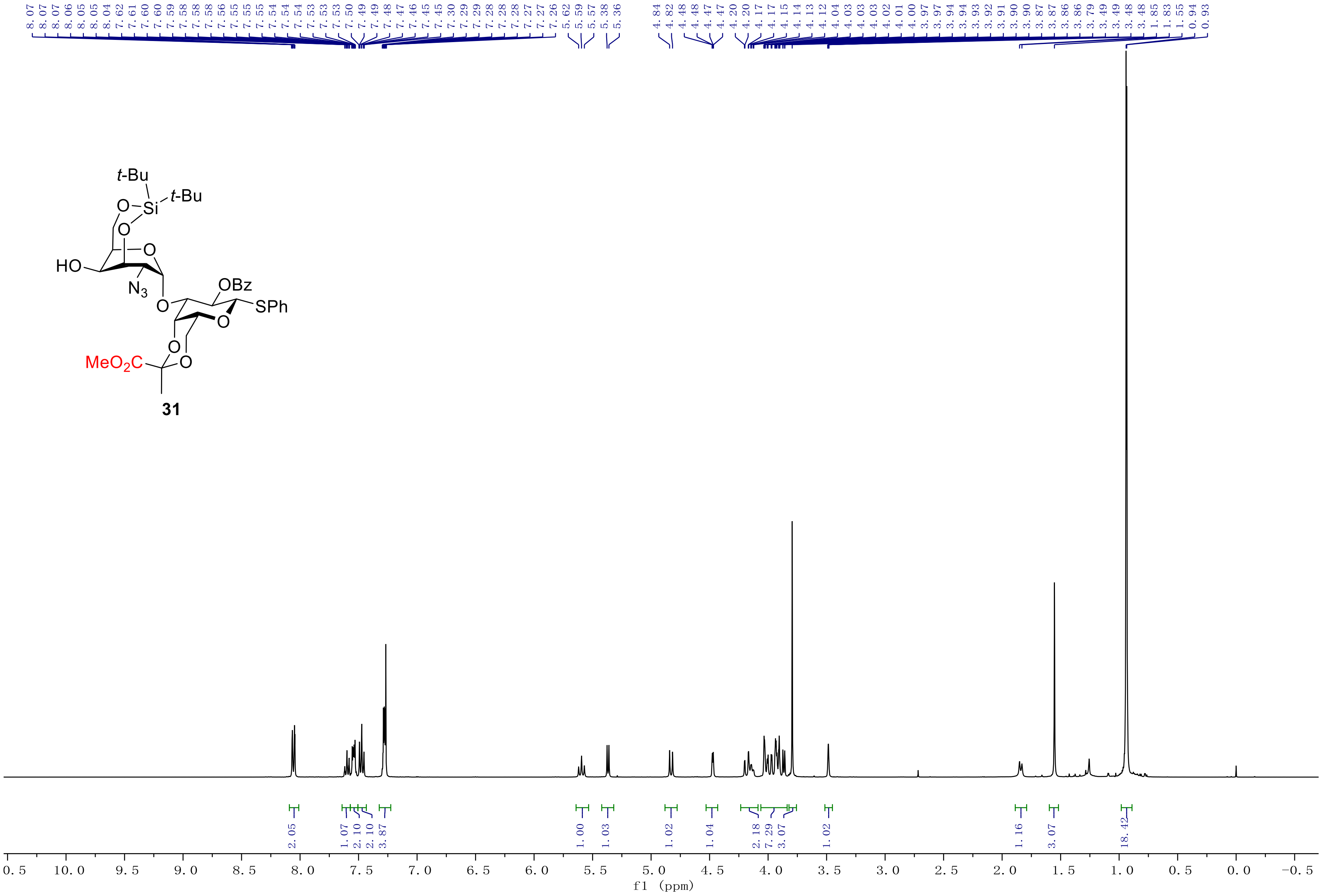

2011zhen.53.fid — wz733-c-1-1 — C13APT CDCl3 /opt/DATA nmrafd 18

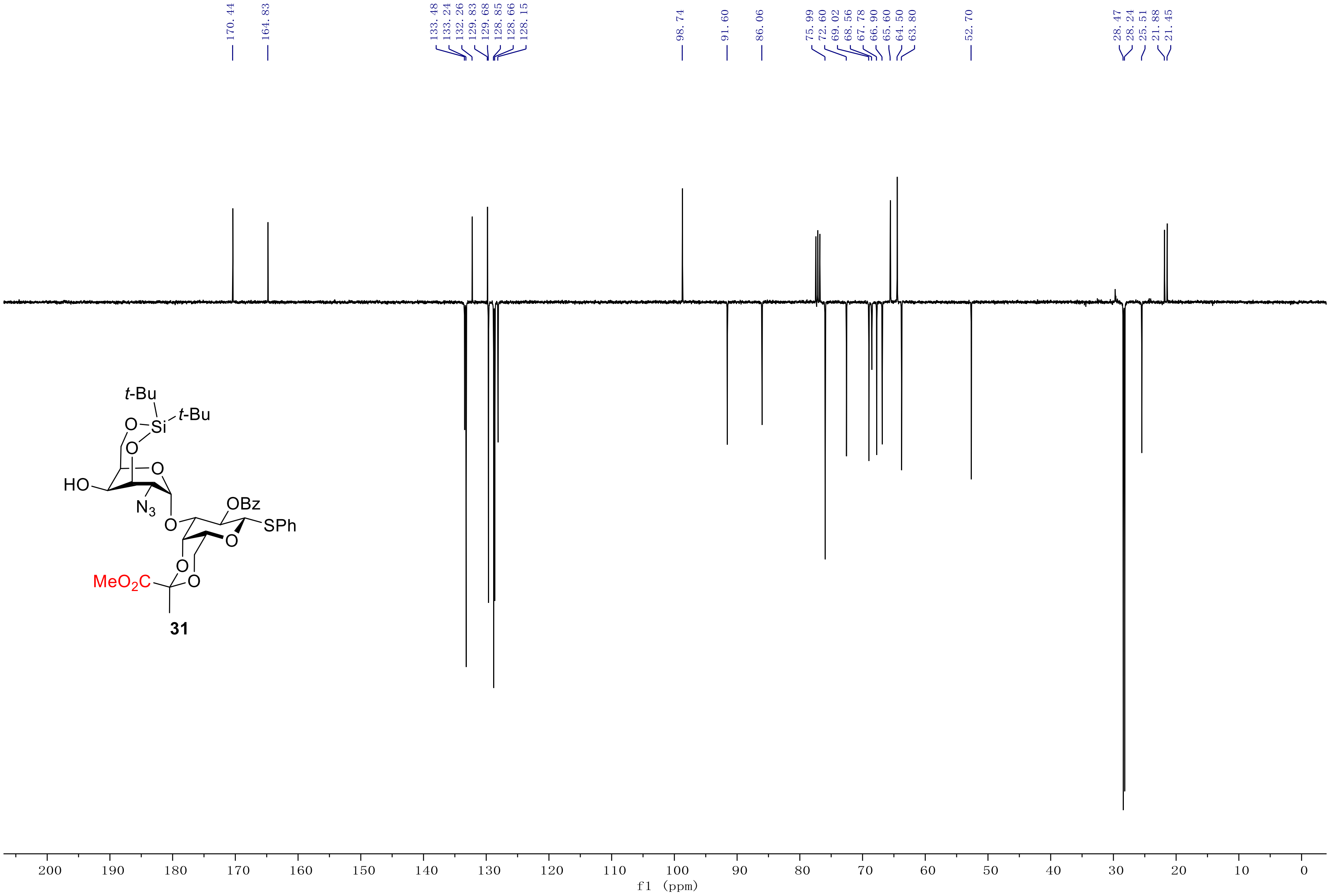

2011zhen.54.ser — wz733-c-1-1 — h1COSY CDCl3 /opt/DATA nmrafd 18

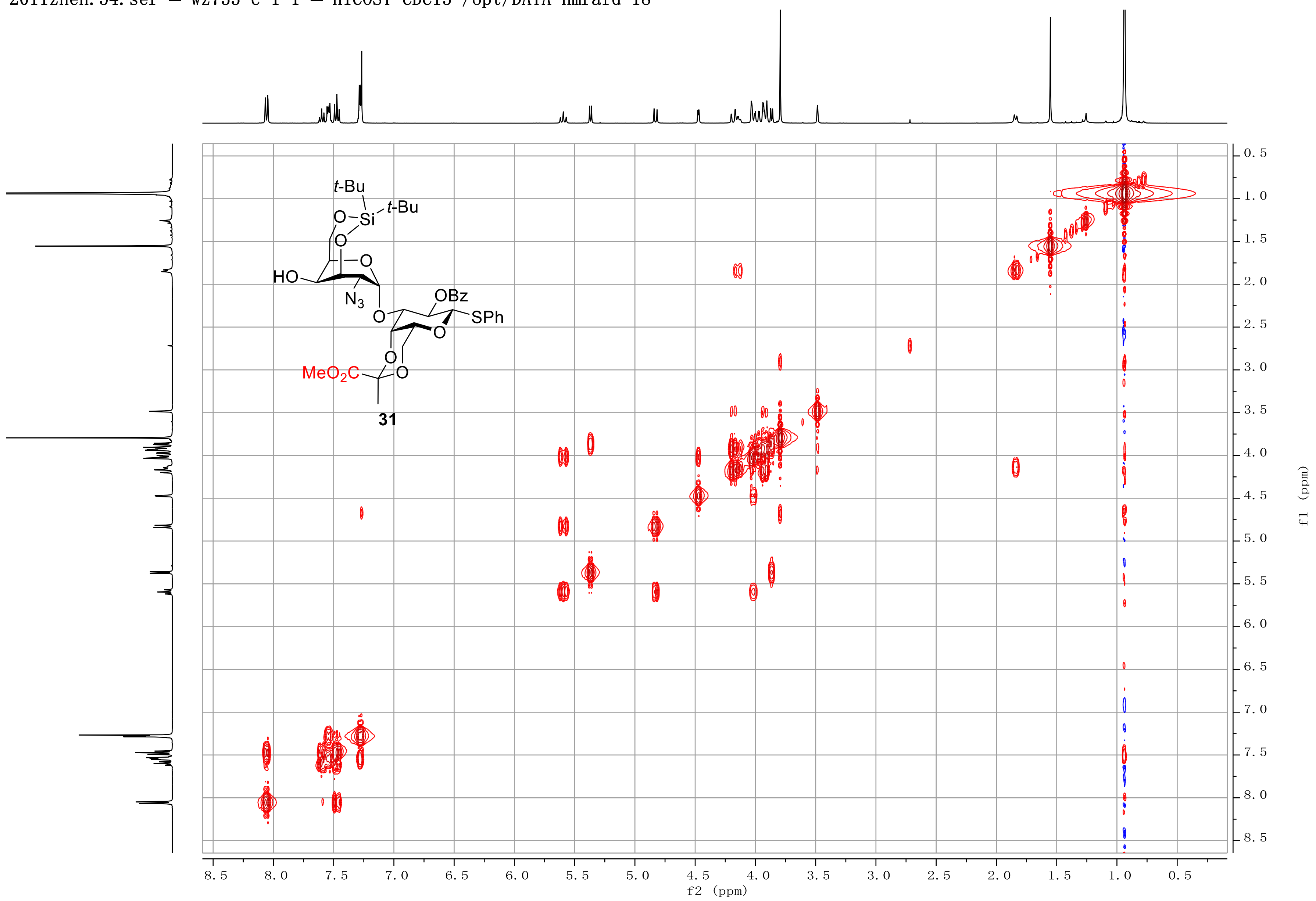

2011zhen.55.ser — wz733-c-1-1 — c13HSQC CDCl3 /opt/DATA nmrafd 18

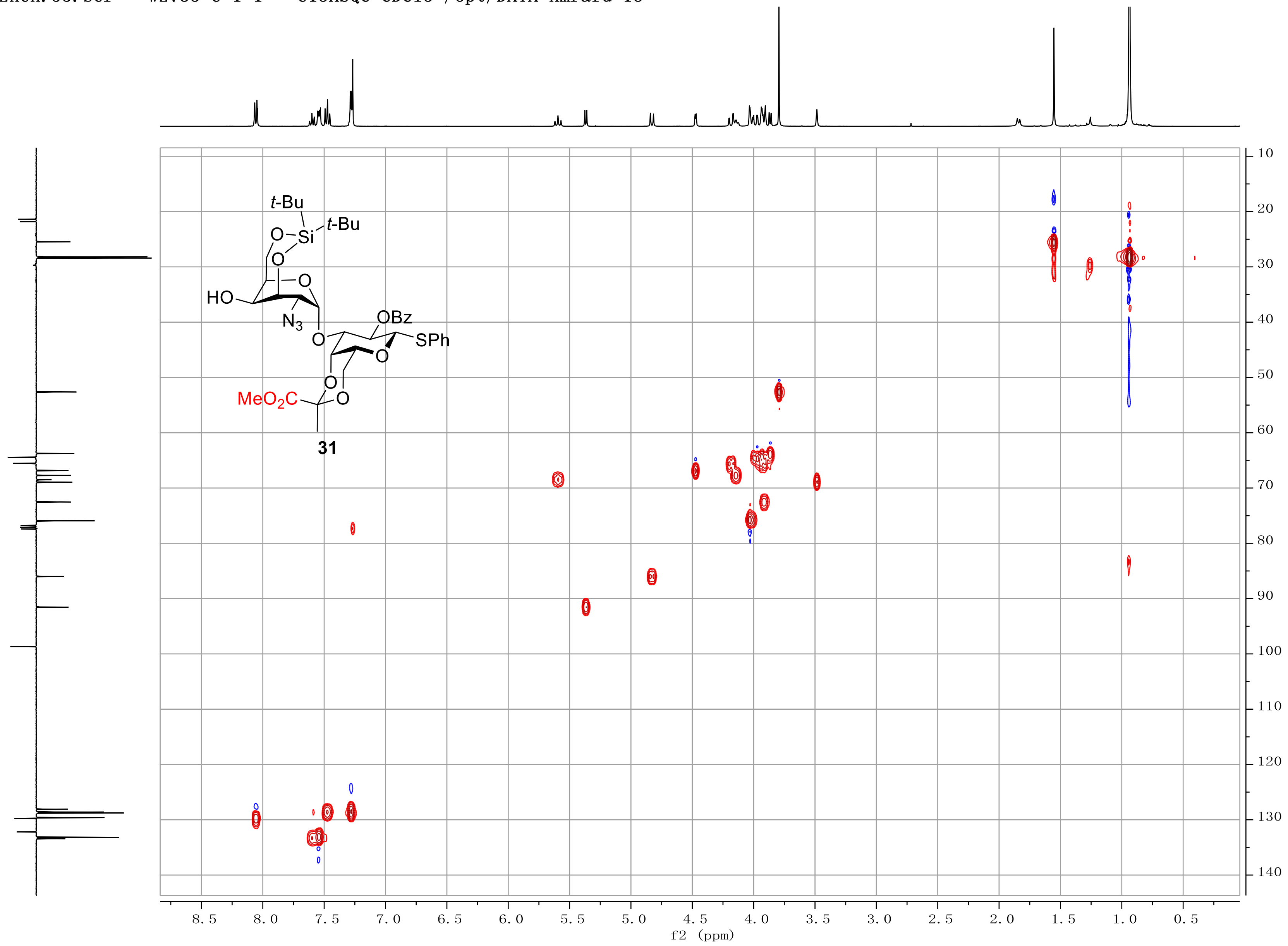

2011zhen.56.ser — wz733-c-1-1 — c13HMBC CDCl3 /opt/DATA nmrafd 18

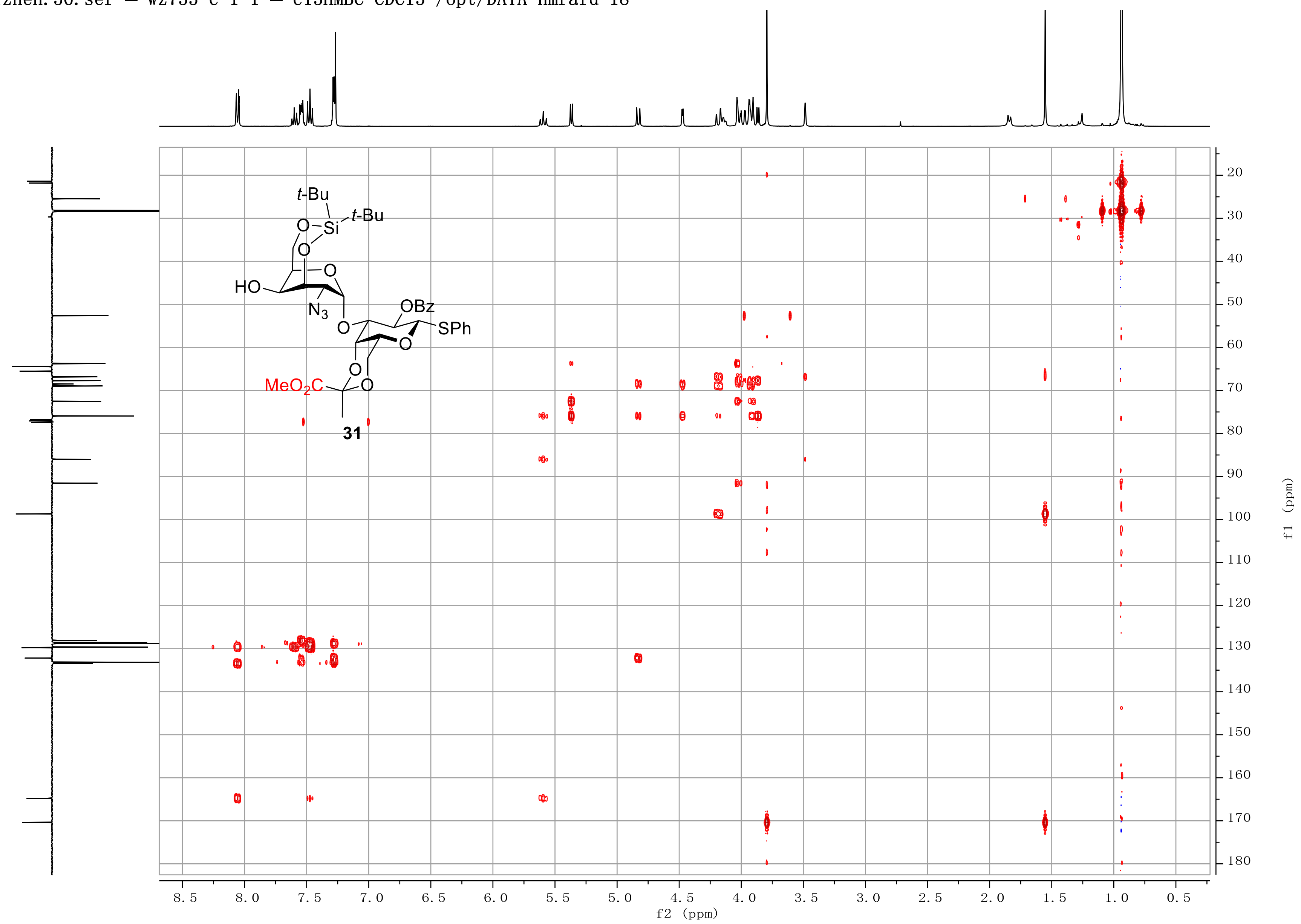

zhen2012biosyn.40.fid — wz742 — bbo-h1 CDCl3 /opt/topspin2.1 nmrafd 4

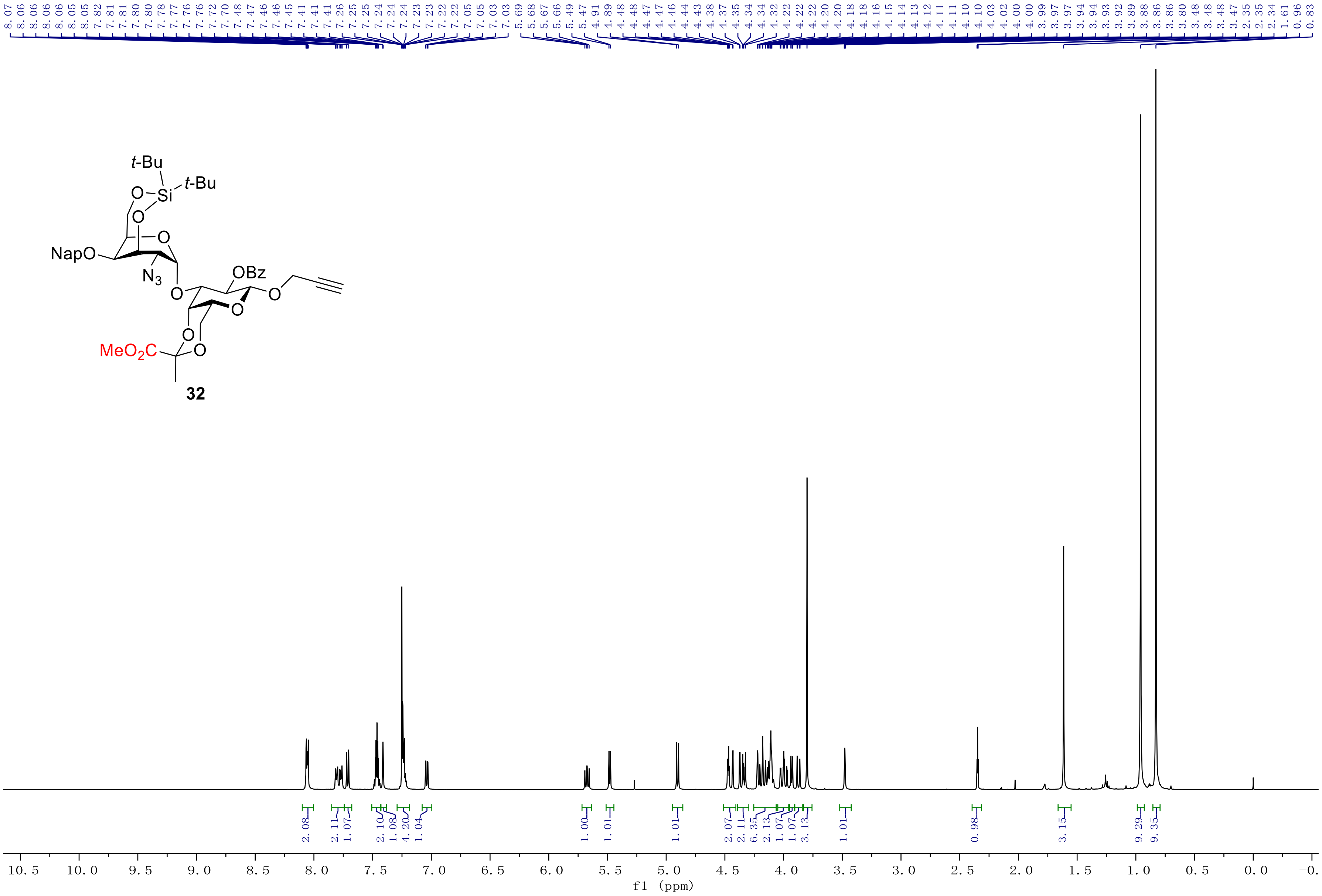

zhen2012biosyn.43.fid — wz742 — bbo-c13-APT CDCl3 /opt/topspin2.1 nmrafd 4

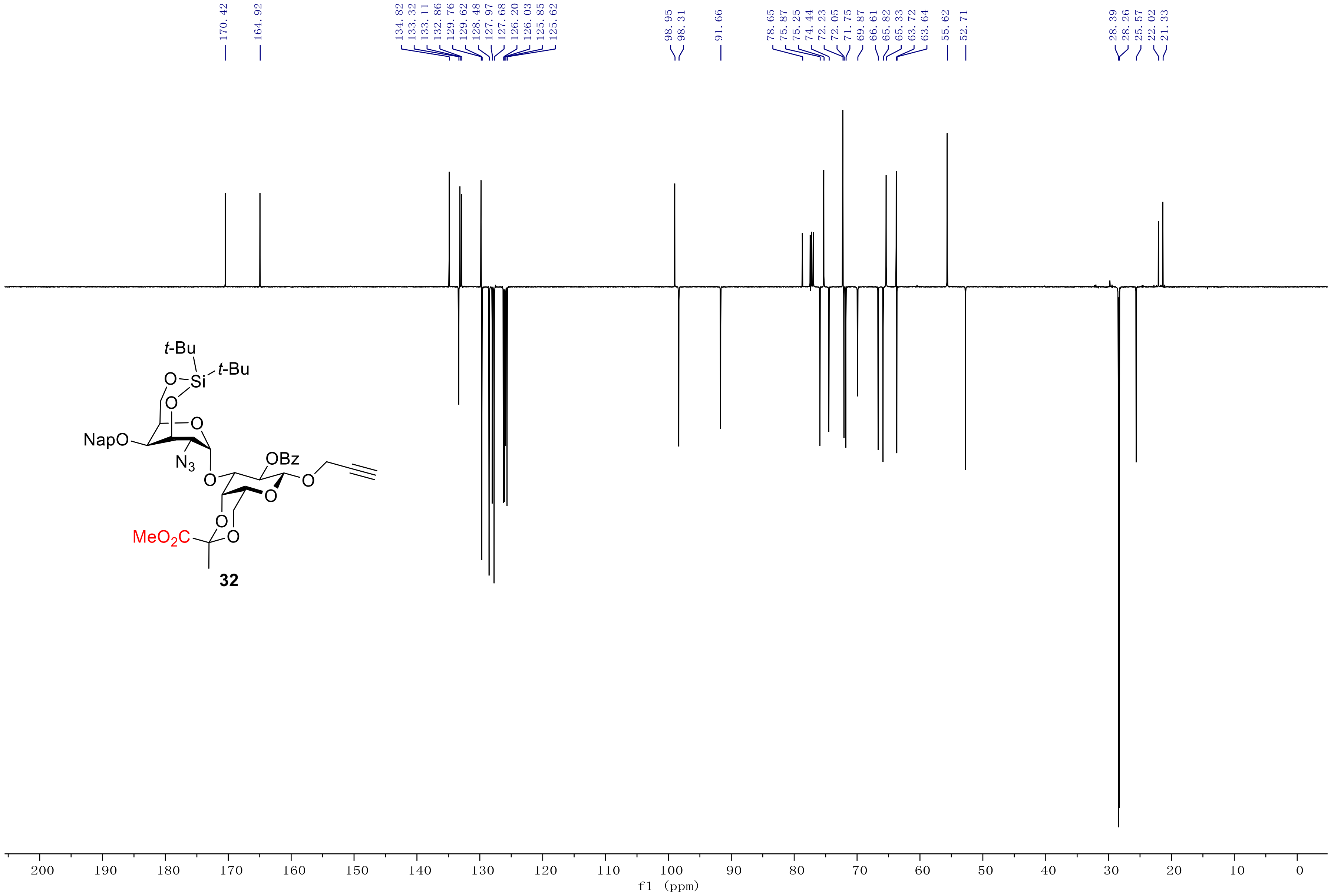

zhen2012biosyn.41.ser — wz742 — bbo-h1-cosy CDCl3 /opt/topspin2.1 nmrafd 4

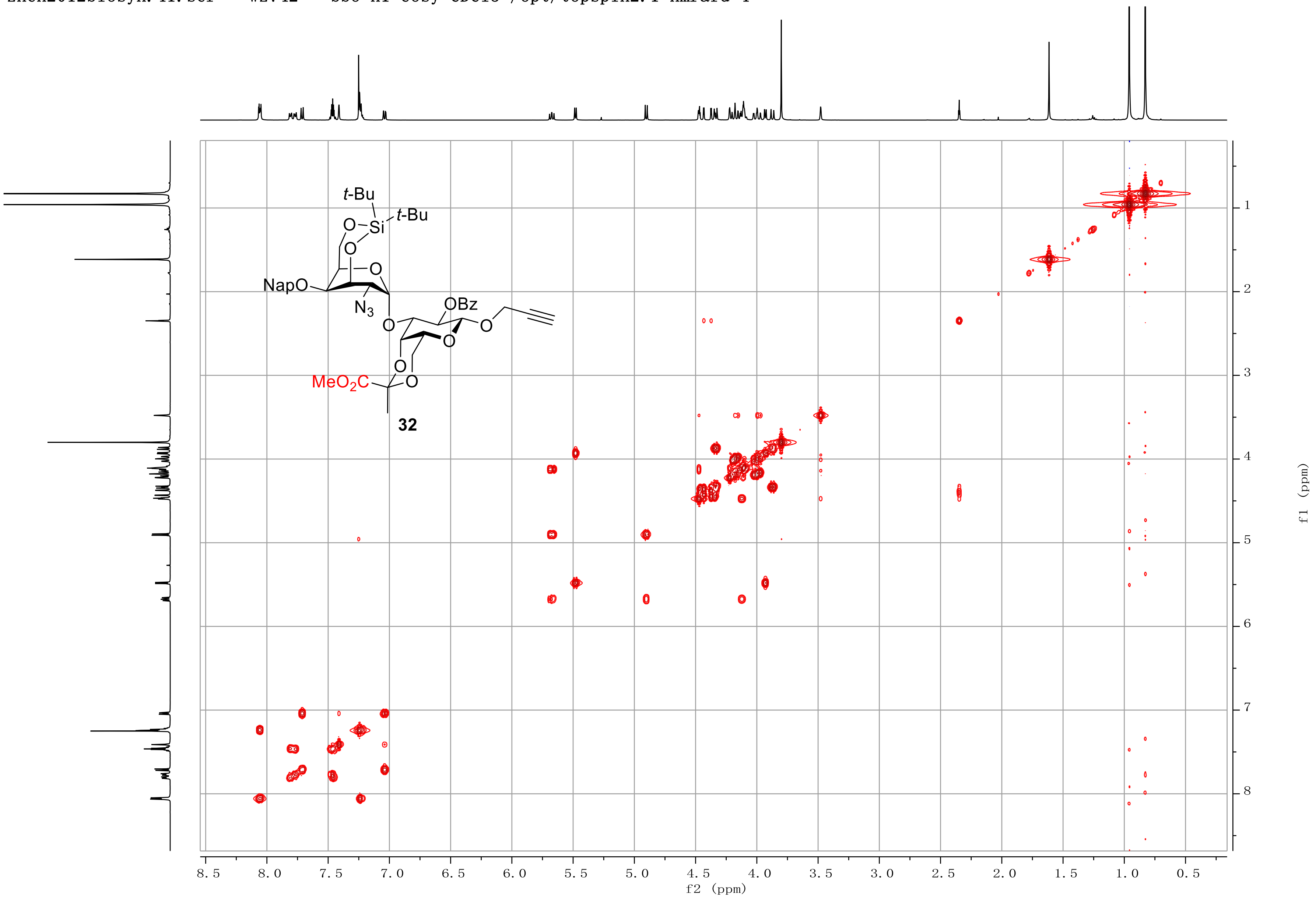

zhen2012biosyn.42.ser — wz742 — bbo-c13-HSQC CDCl3 /opt/topspin2.1 nmrafd 4

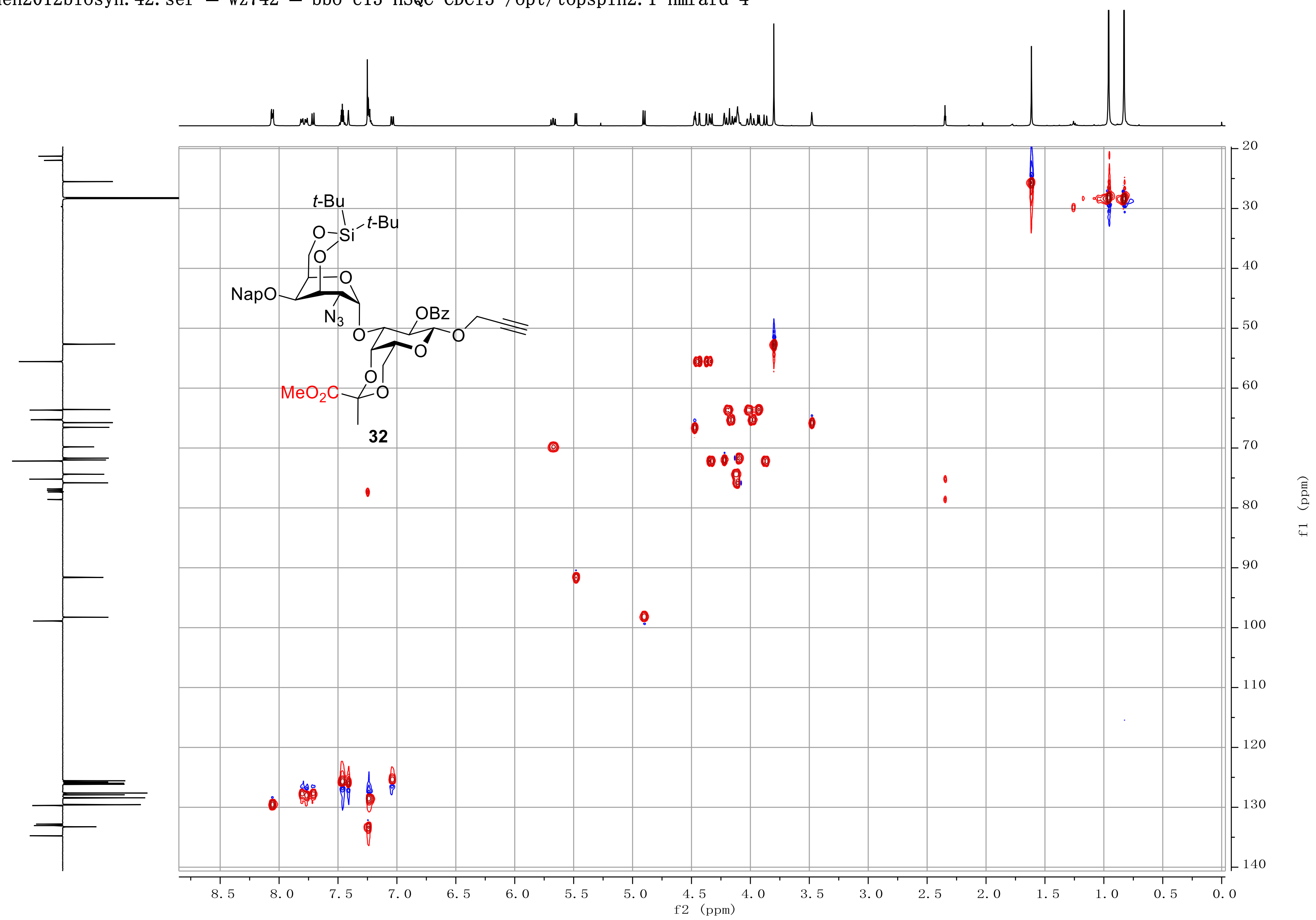

zhen2012biosyn.44.ser — wz742 — bbo-c13-HMBC CDCl3 /opt/topspin2.1 nmrafd 4

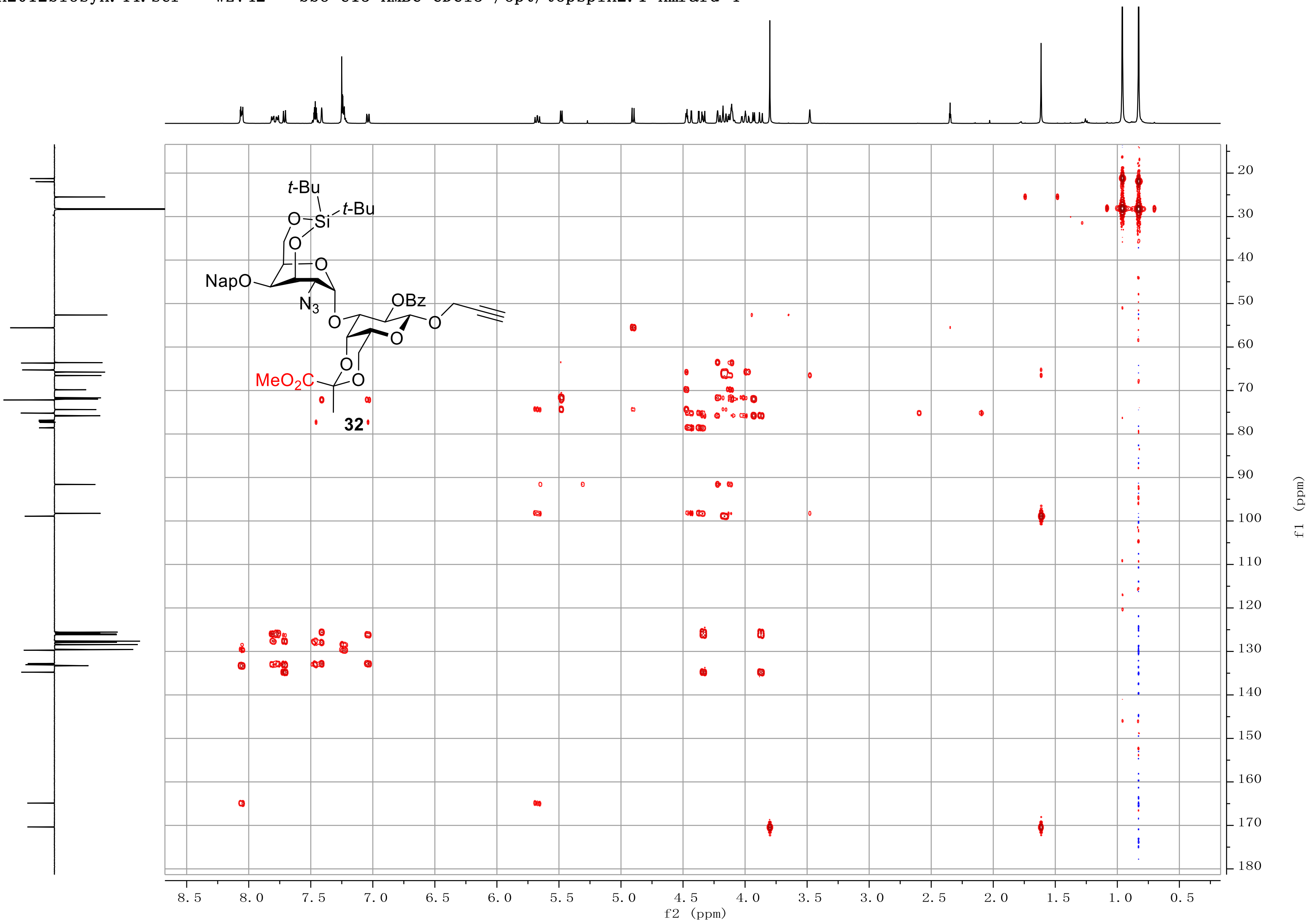

zhen2012biosyn.59.fid — wz739-B — bbo-h1 CDCl3 /opt/topspin2.1 nmrafd 11

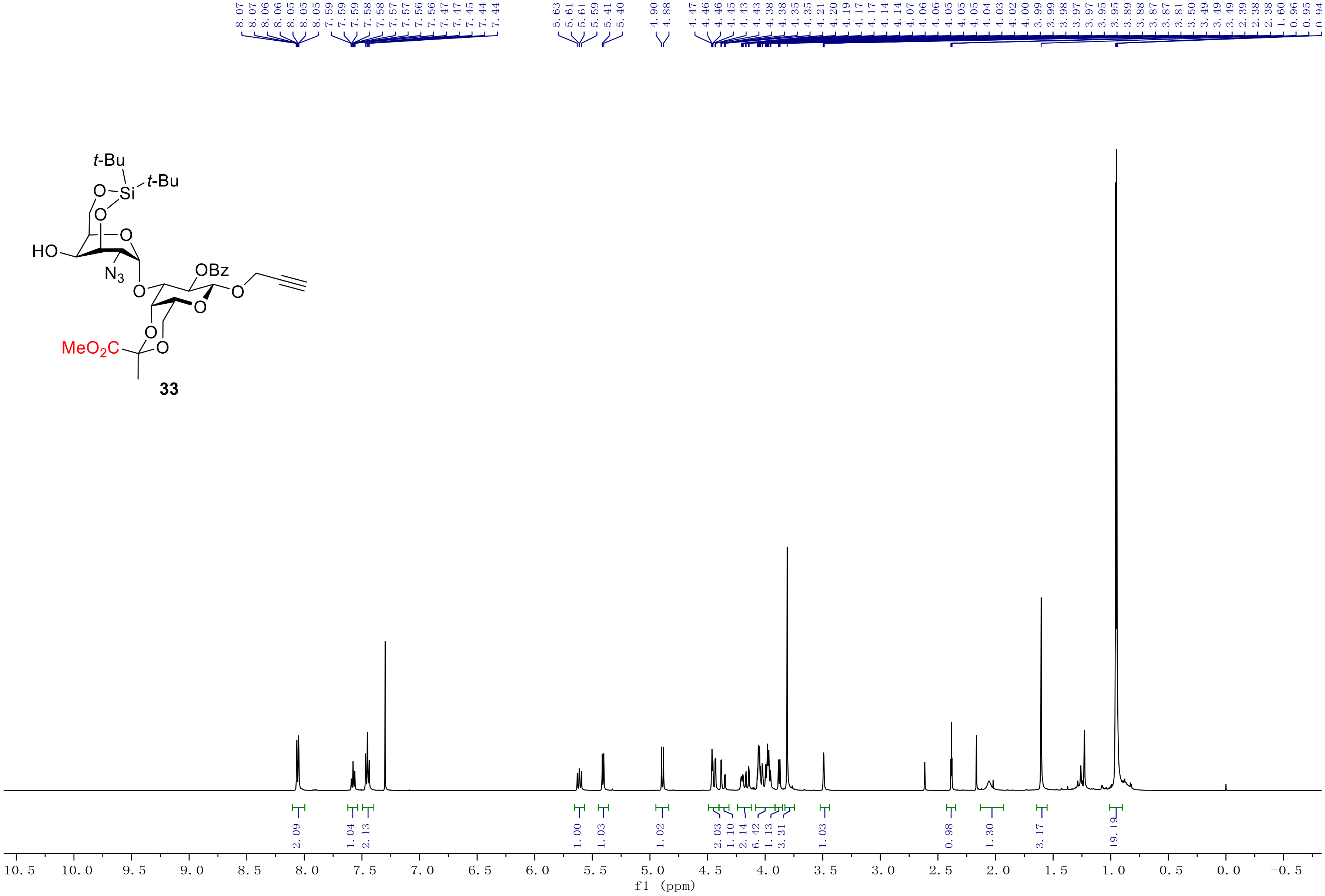

zhen2012biosyn.62.fid — wz739-B — bbo-c13-APT CDCl3 /opt/topspin2.1 nmrafd 11

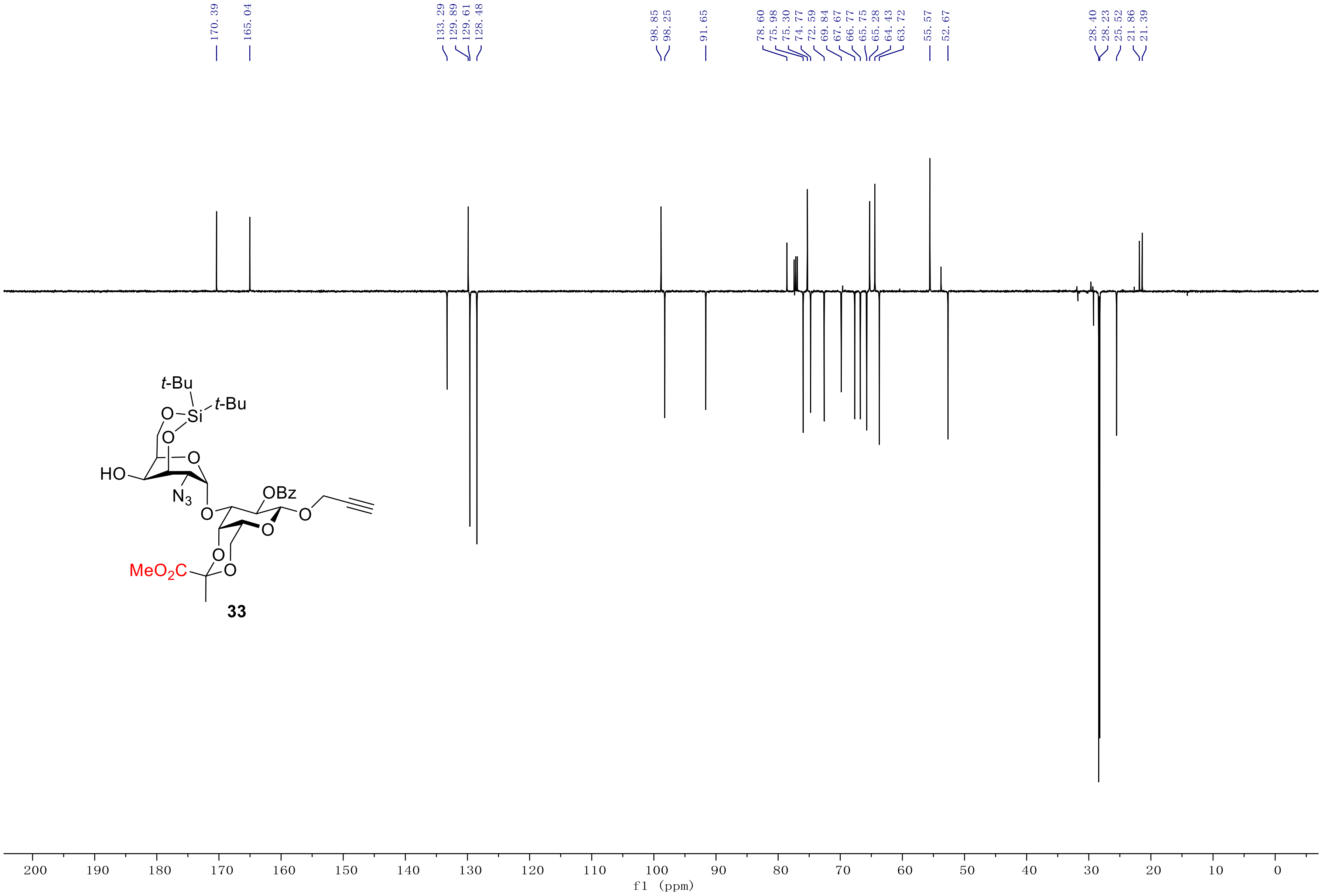

zhen2012biosyn.60.ser — wz739-B — bbo-h1-cosy CDCl3 /opt/topspin2.1 nmrafd 11

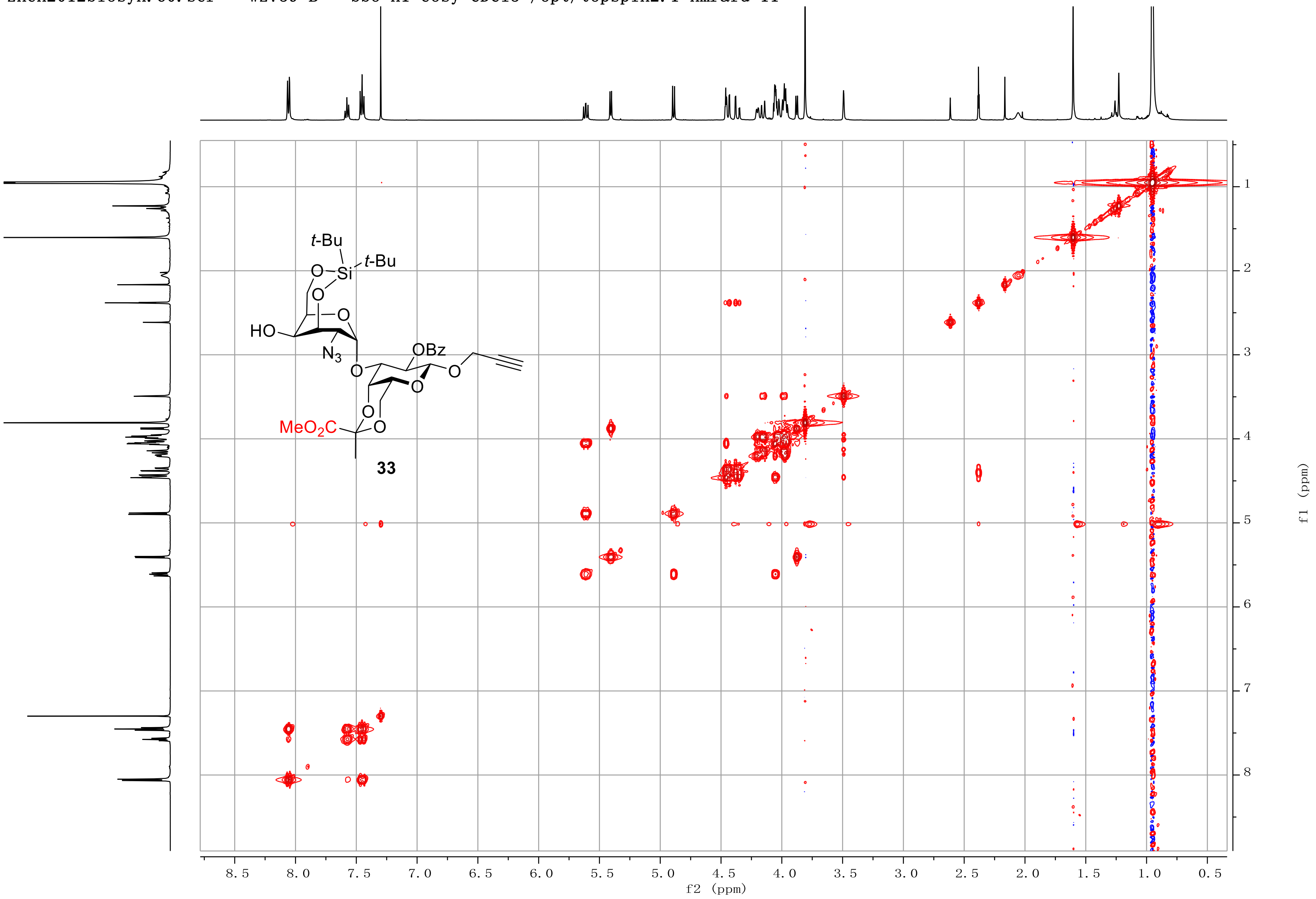

zhen2012biosyn.61.ser — wz739-B — bbo-c13-HSQC CDCl3 /opt/topspin2.1 nmrafd 11

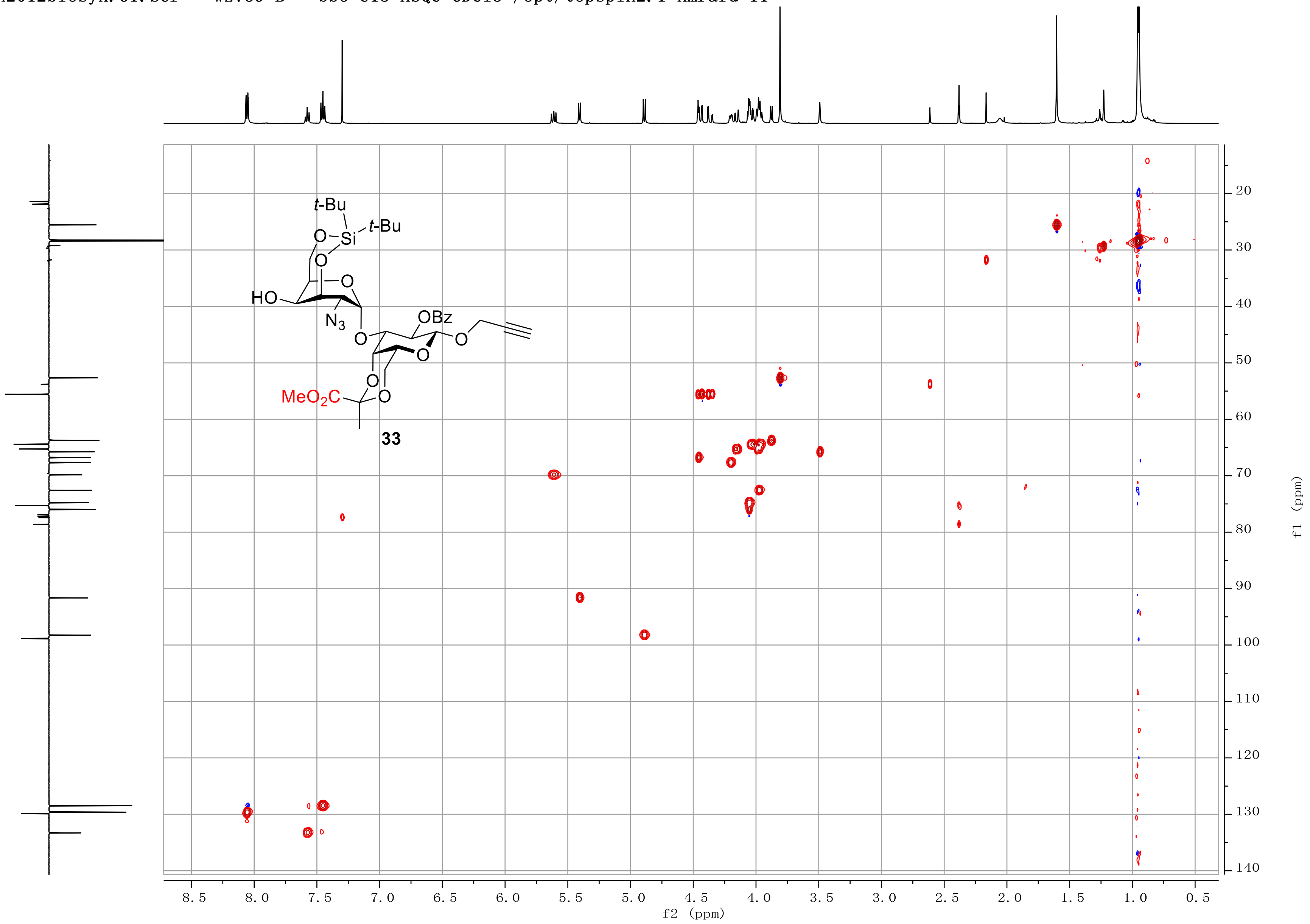

zhen2012biosyn.63.ser — wz739-B — bbo-c13-HMBC CDCl3 /opt/topspin2.1 nmrafd 11

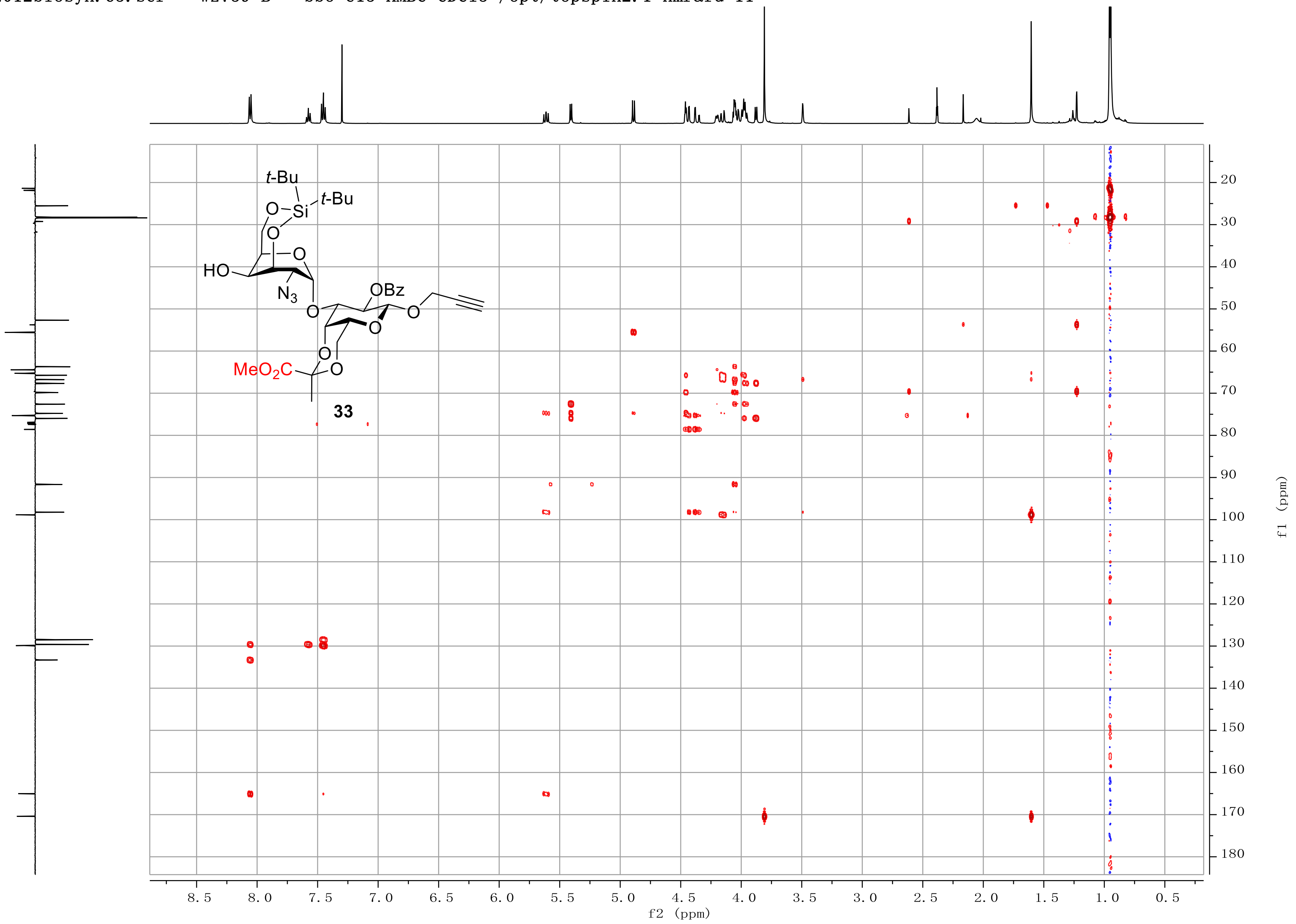

2011zhen.58.fid — wz736-D-2-2 — h1 CDCl3 /opt/DATA nmrafd 19

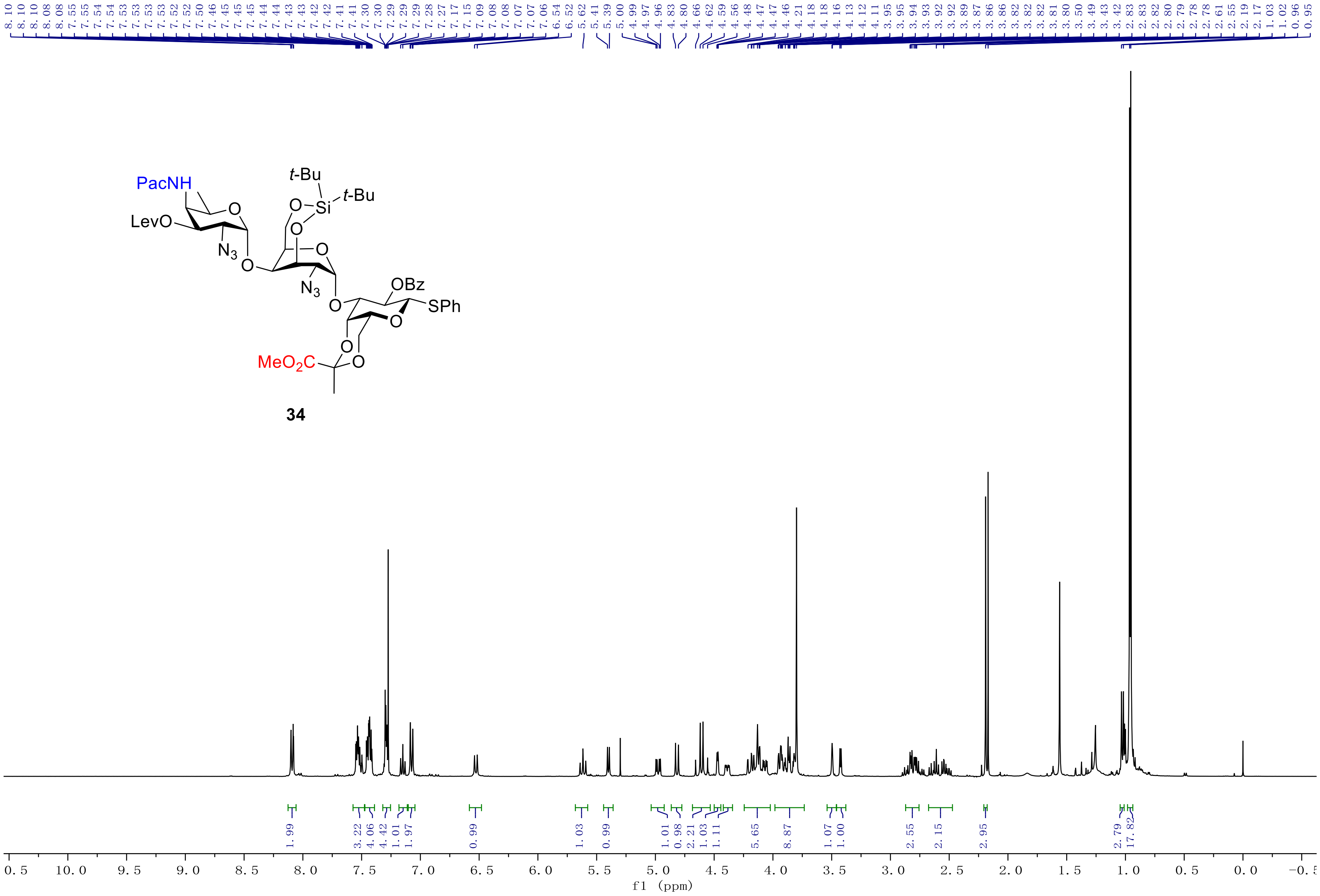

34

2011zhen.59.fid — wz736-D-2-2 — C13APT CDCl3 /opt/DATA nmrafd 19

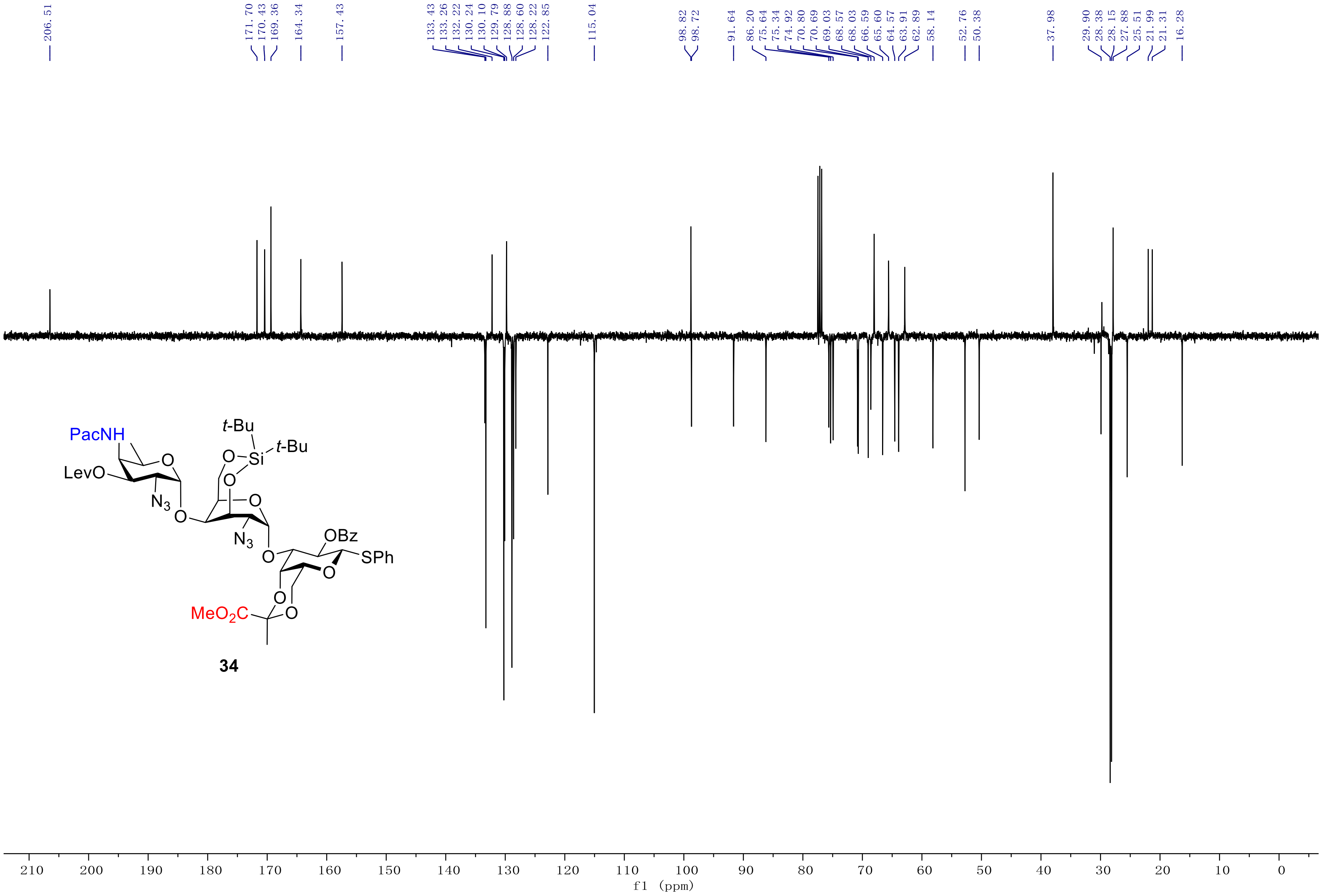

2011zhen.60.ser — wz736-D-2-2 — h1COSY CDCl3 /opt/DATA nmrafd 19

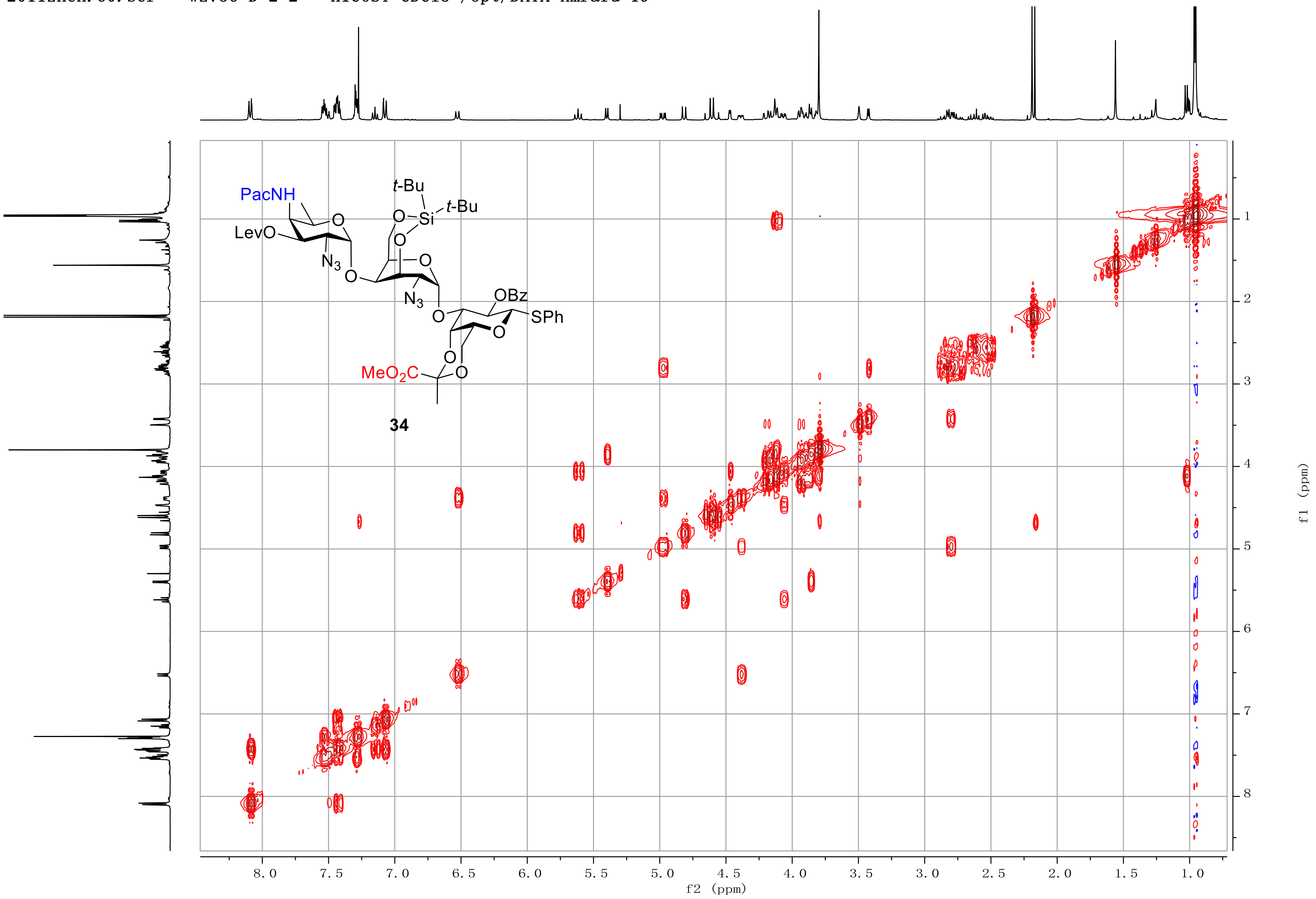

2011zhen.61.ser — wz736-D-2-2 — c13HSQC CDCl3 /opt/DATA nmrafd 19

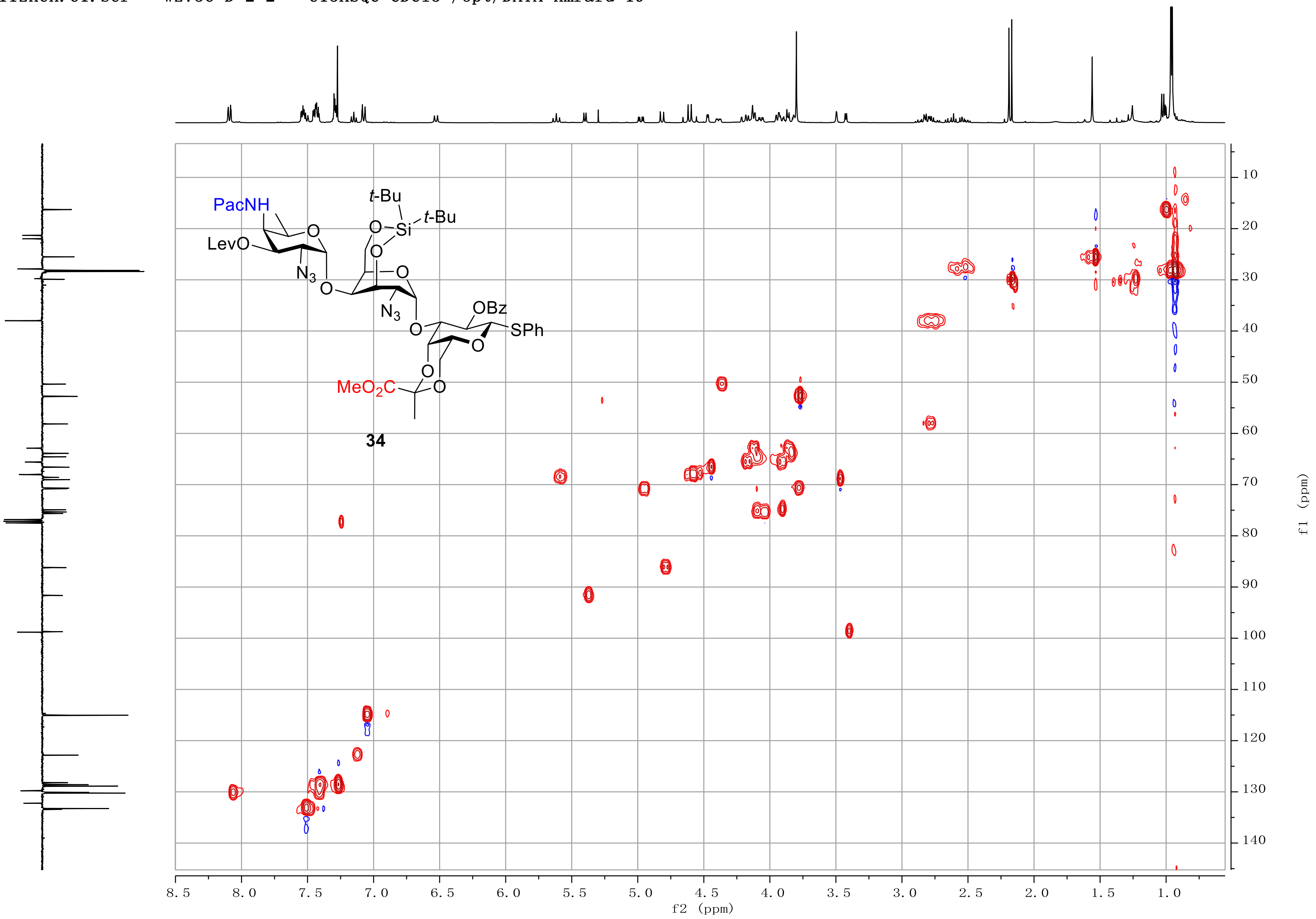

2011zhen.62.ser — wz736-D-2-2 — c13HMBC CDCl3 /opt/DATA nmrafd 19

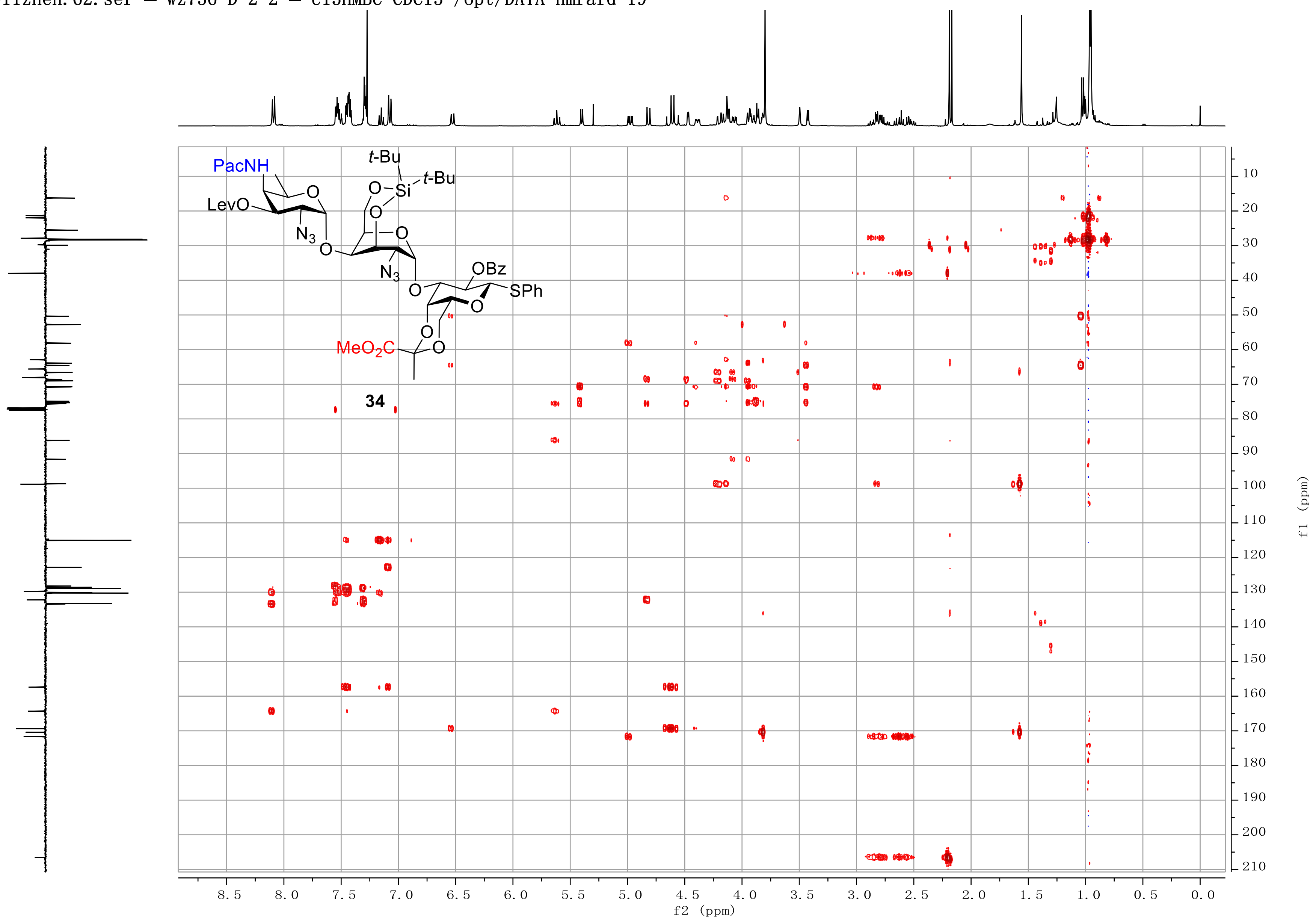

2104zhen.34.fid — wz755-D, s — h1 CDCl3 /opt/DATA nmrafd 15

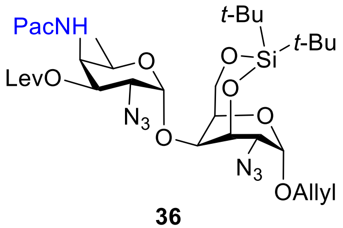

36

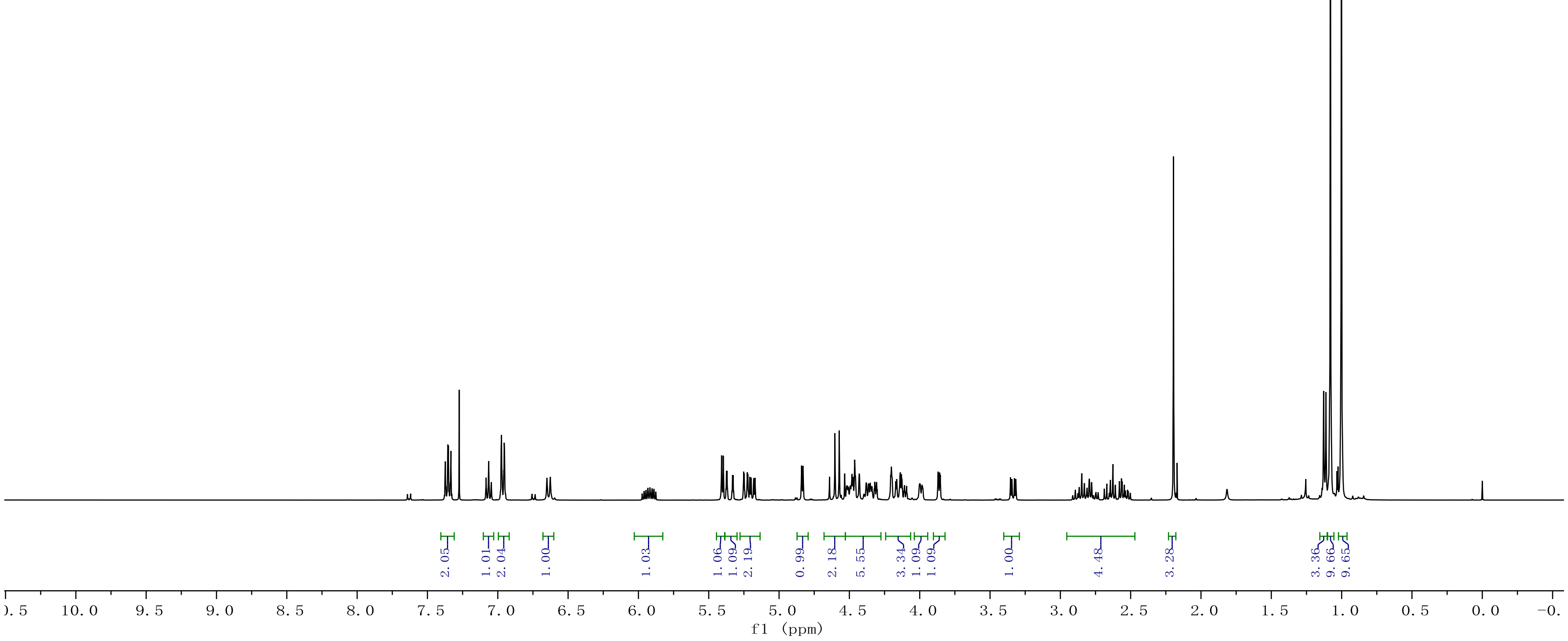

2104zhen.35.fid — wz755-D, s — C13APT CDCl3 /opt/DATA nmrafd 15

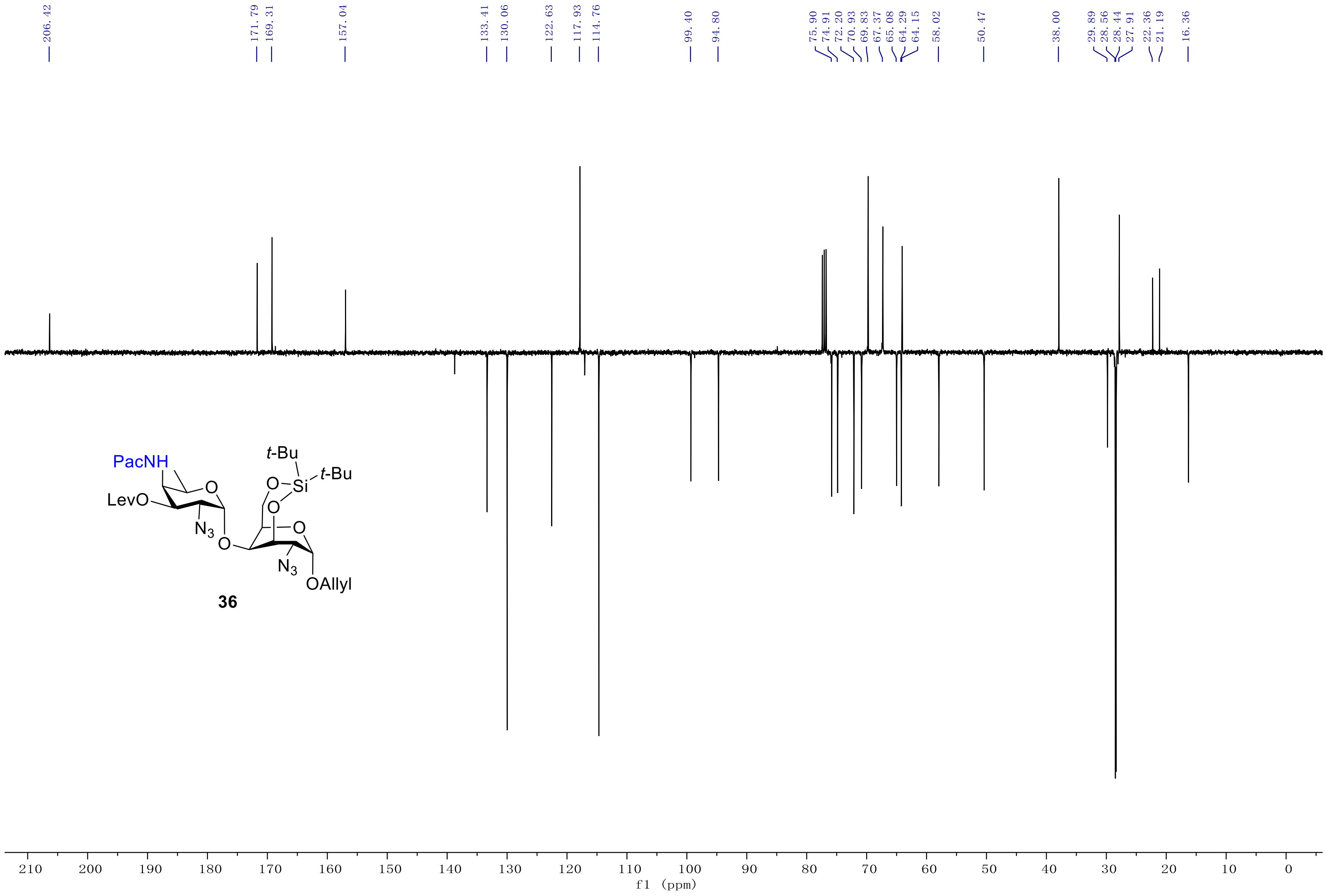

2104zhen.36.ser — wz755-D, s — h1COSY CDCl3 /opt/DATA nmrafd 15

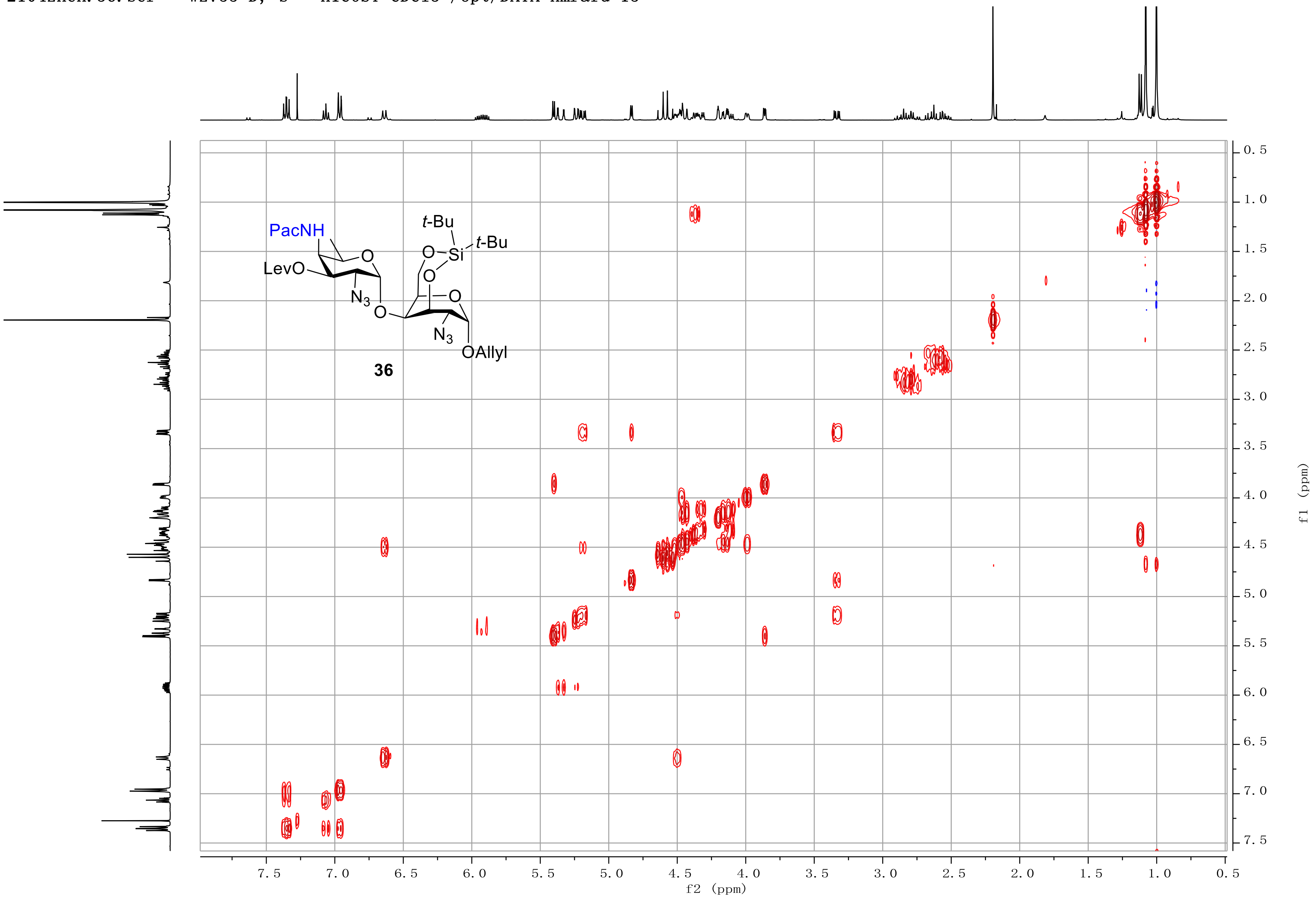

2104zhen.37.ser — wz755-D, s — c13HSQC CDCl3 /opt/DATA nmrafd 15

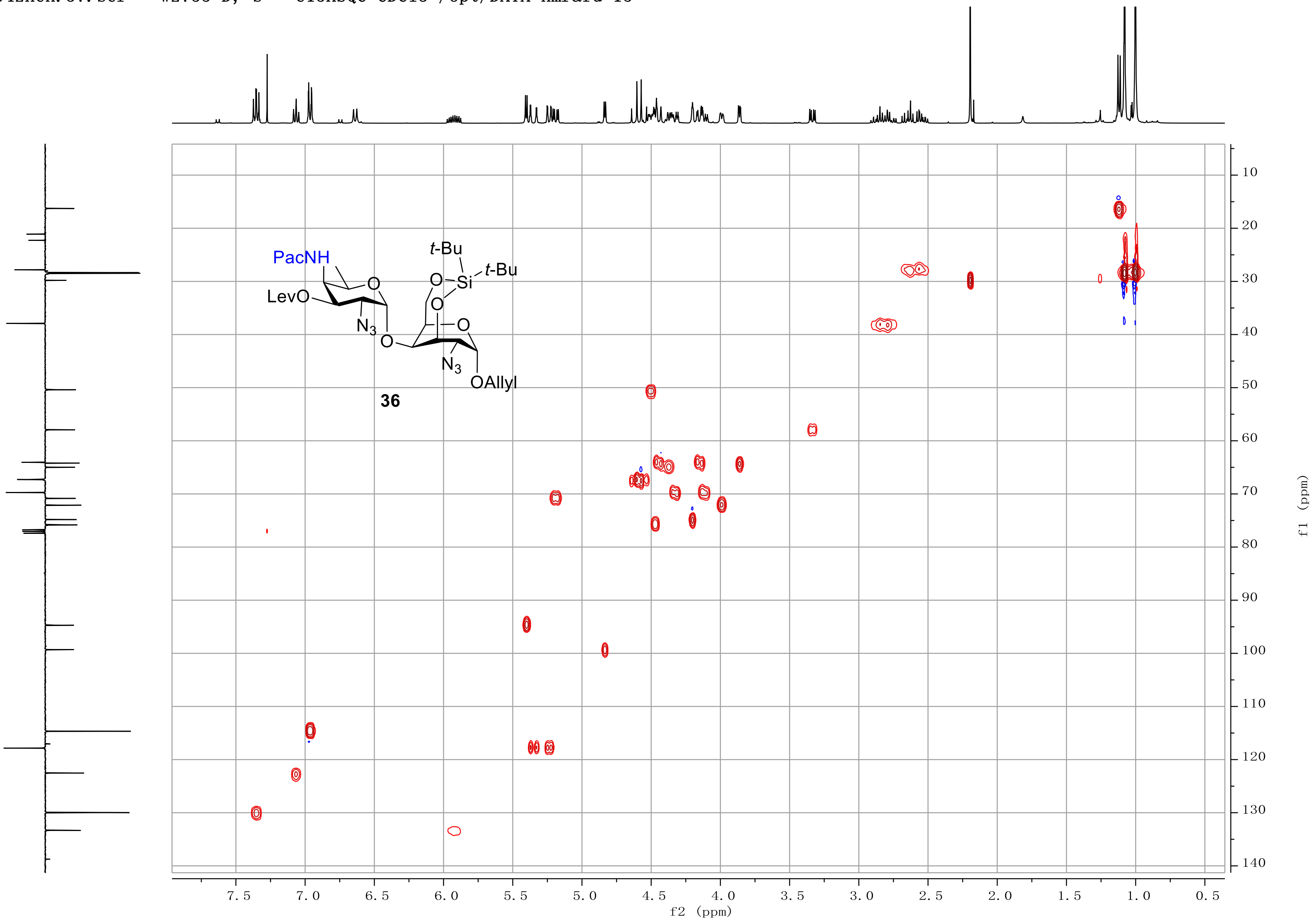

2104zhen.38.ser — wz755-D, s — c13HMBC CDCl3 /opt/DATA nmrafd 15

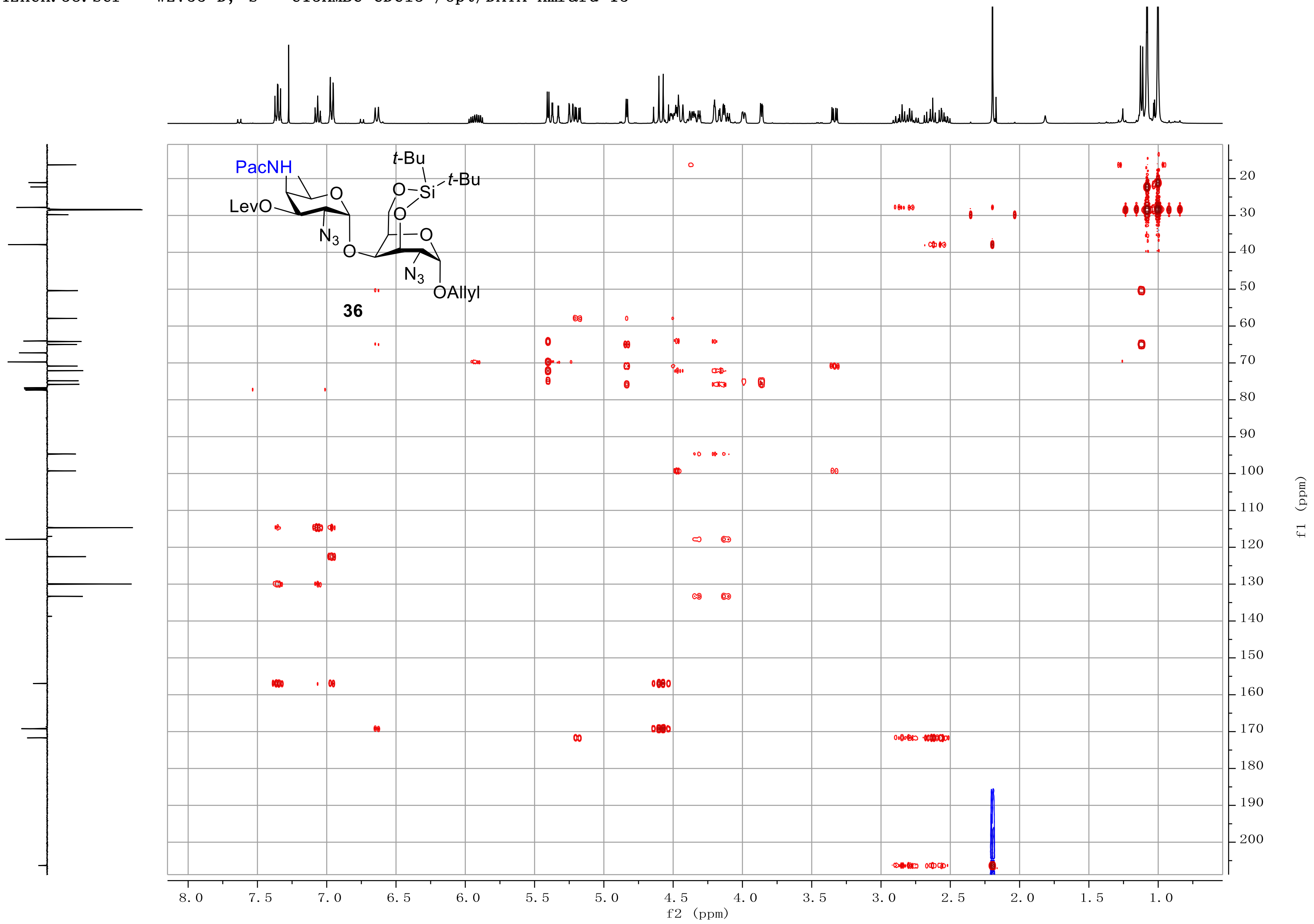

2104zhen.39.ser — wz755-D, s — c13HMBCipvGATED CDCl3 /opt/DATA nmrafd 15

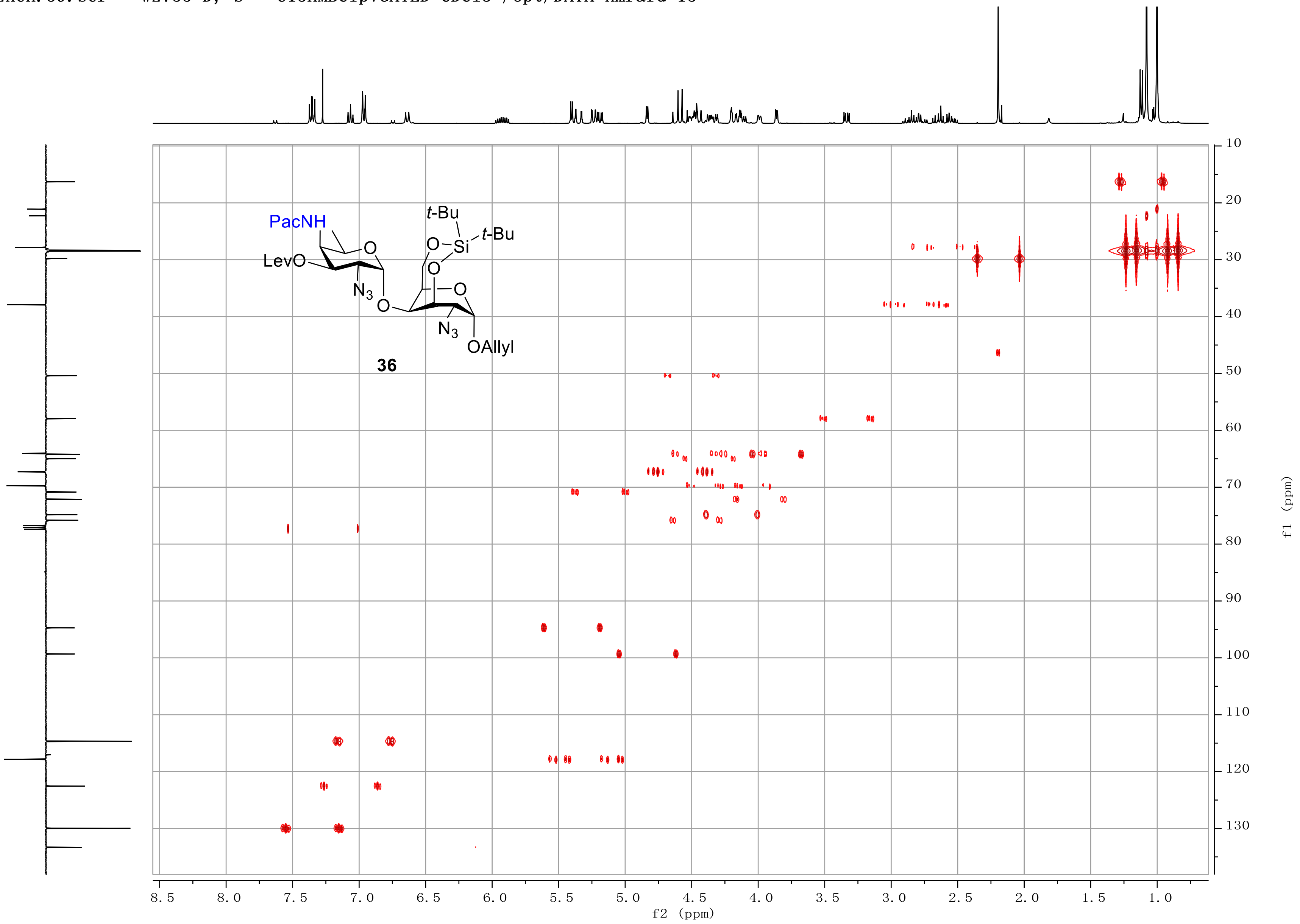

2104zhen.20.fid — wz762-3 — h1 CDCl3 /opt/DATA nmrafd 12

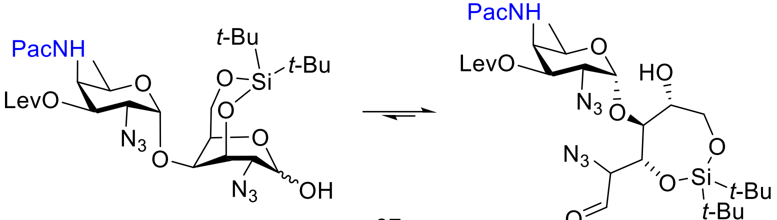

37a

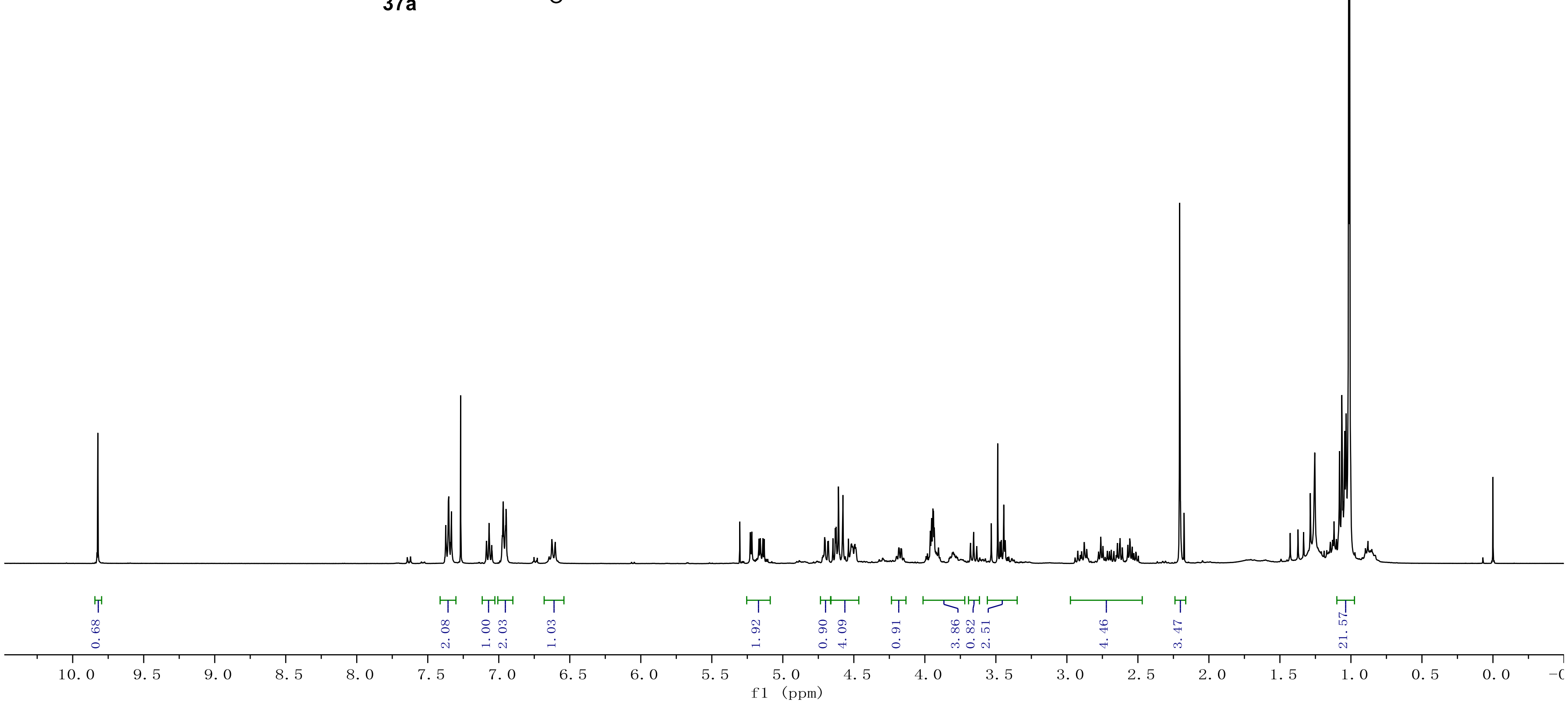

zhen2104biosyn.57.fid — wz762-1 — bbo-c13-APT CDCl3 /opt/topspin2.1 nmrafd 8

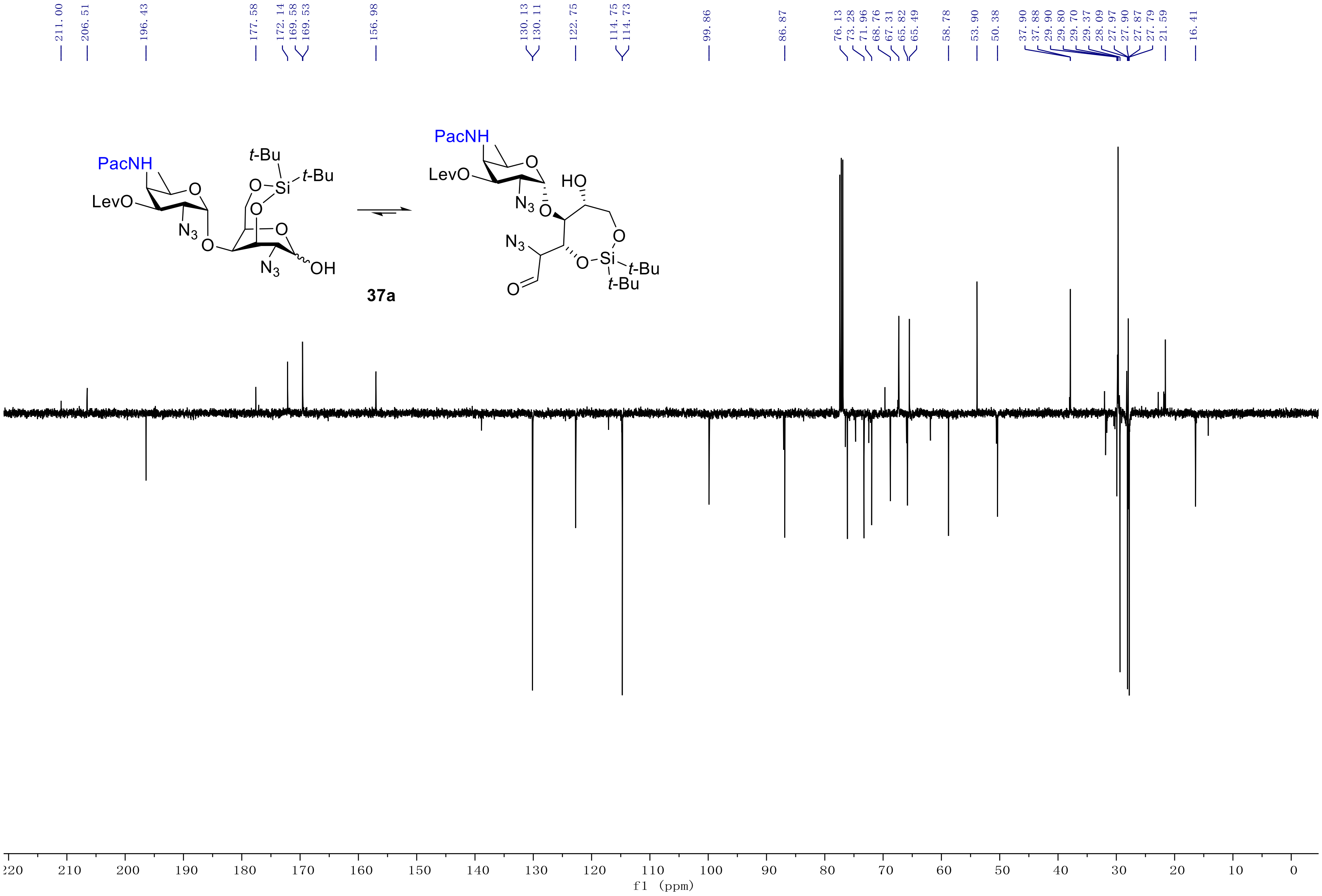

2104zhen.21.ser — wz762-3 — h1COSY CDCl3 /opt/DATA nmrafd 12

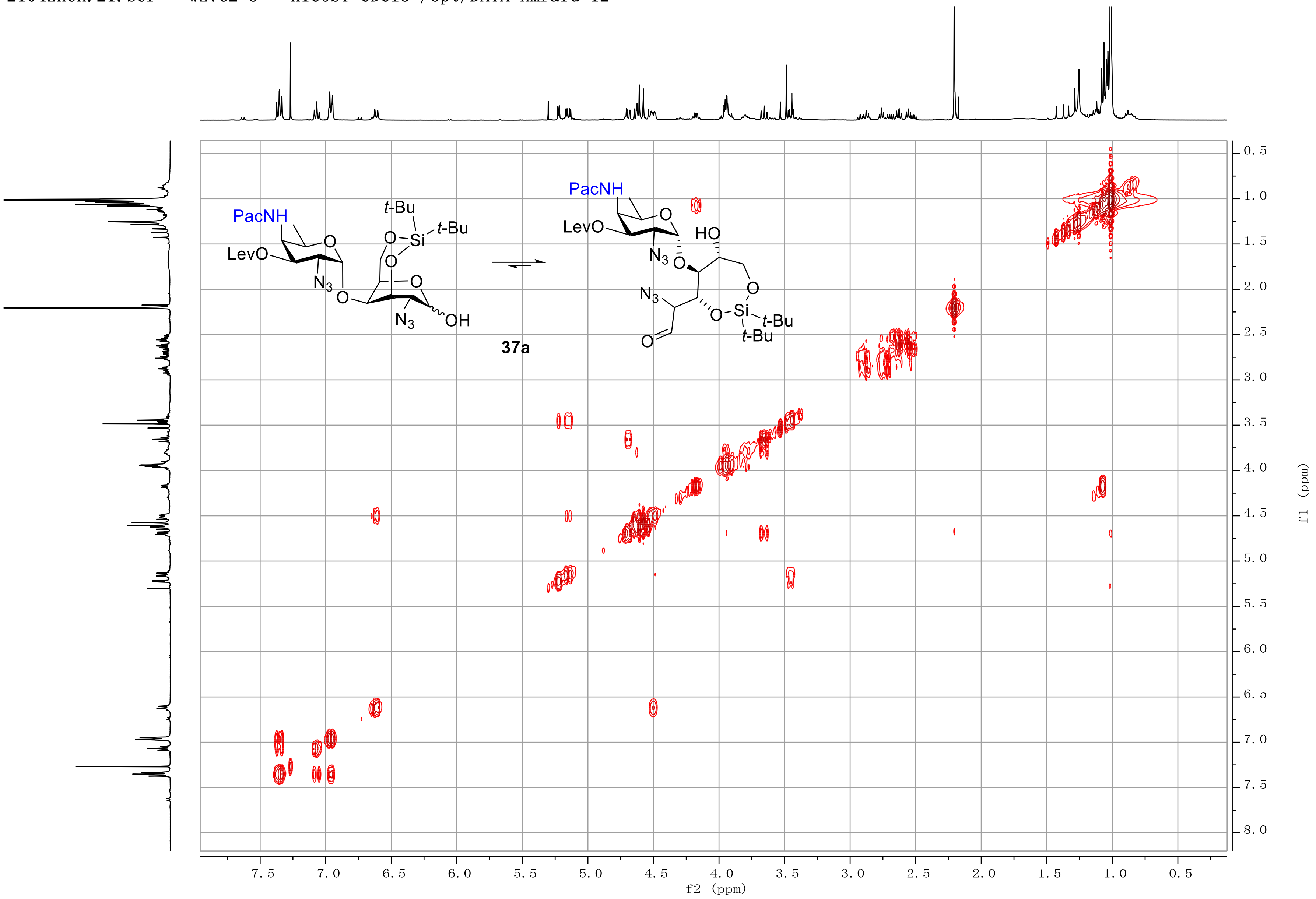

zhen2104biosyn.59.ser — wz762-1 — bbo-c13-HSQC-200ppm CDCl3 /opt/topspin2.1 nmrafd 8

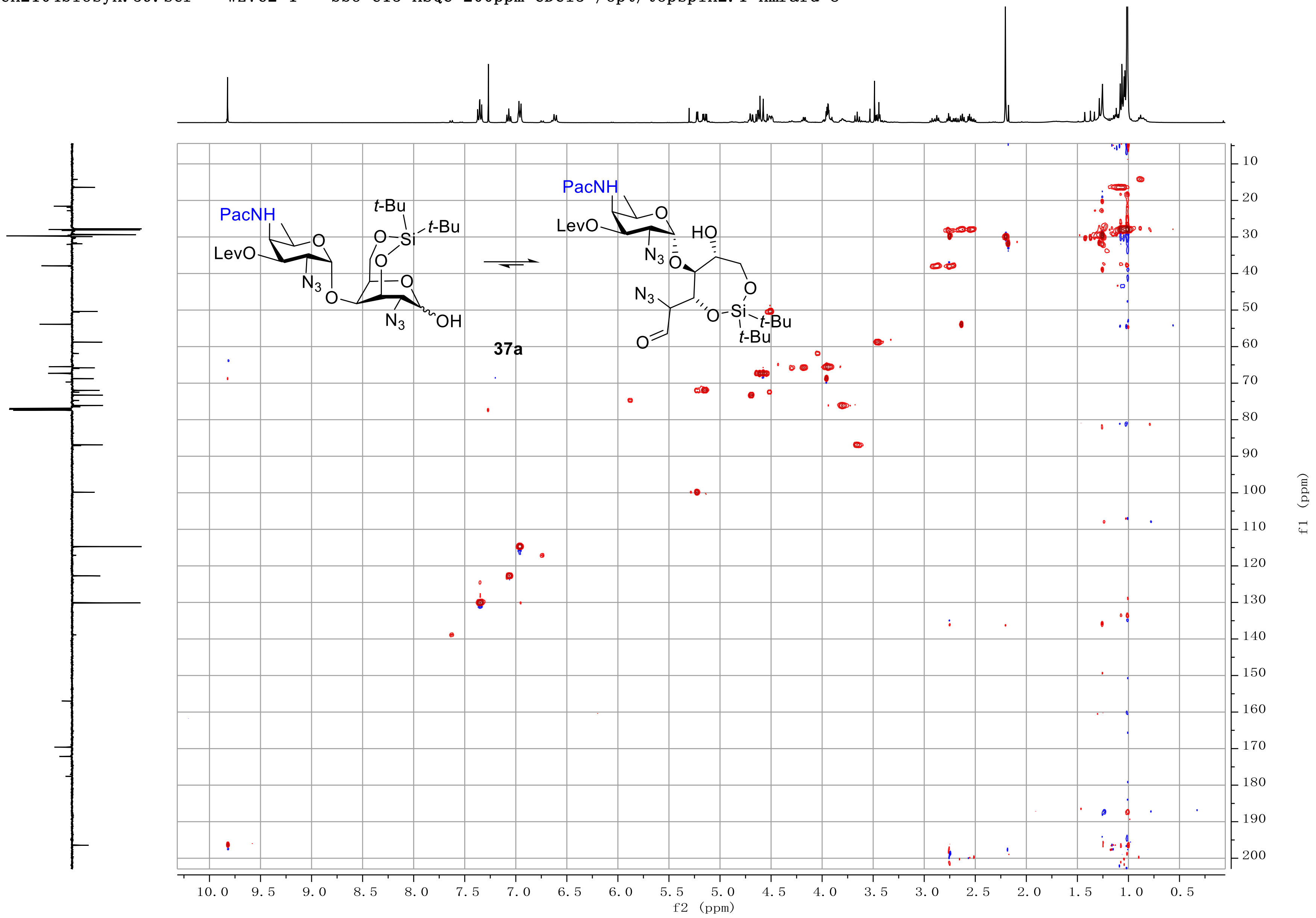

zhen2104biosyn.58.ser — wz762-1 — bbo-c13-HMBC CDCl3 /opt/topspin2.1 nmrafd 8

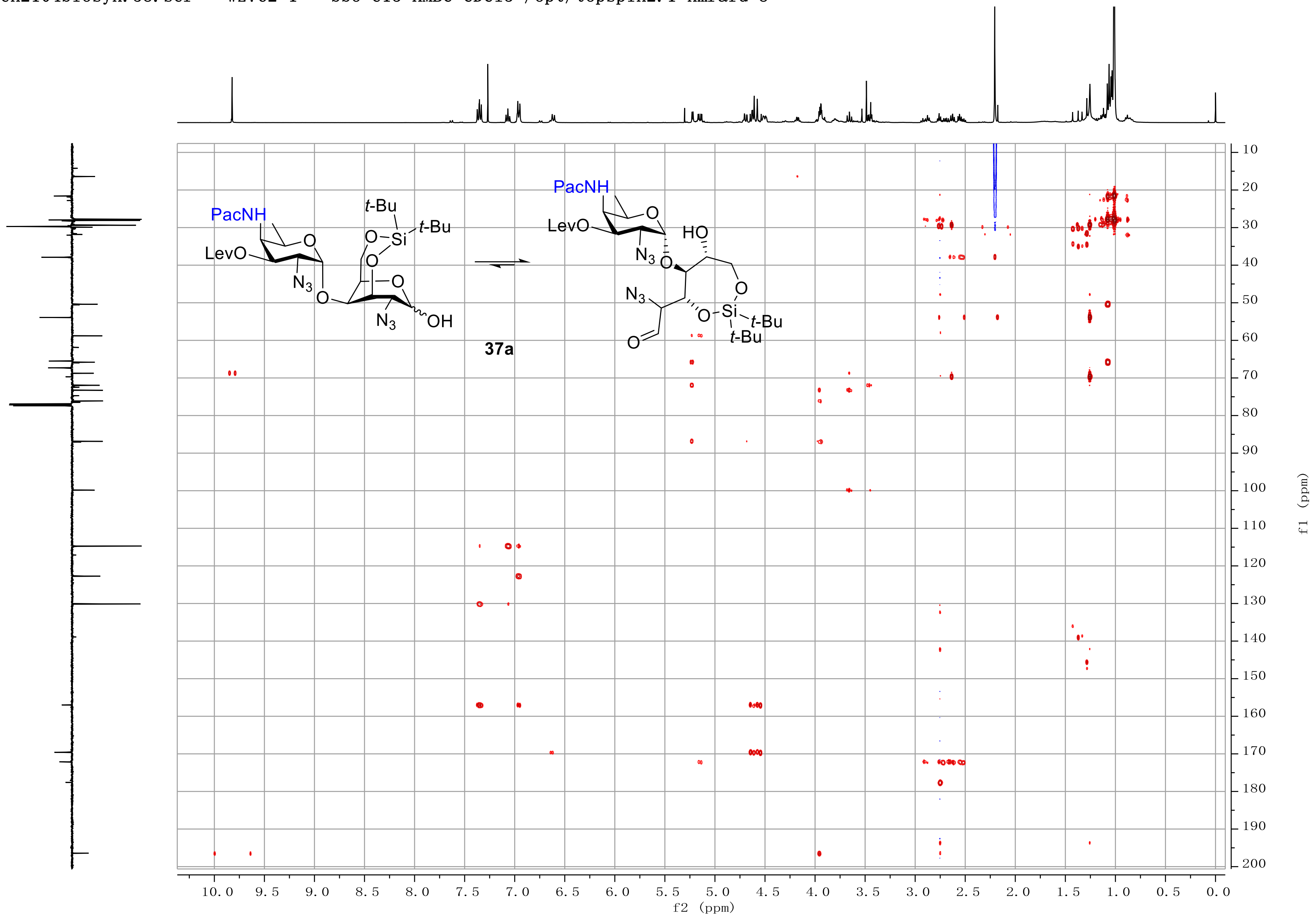

zhen2104biosyn.83.fid — wz765-A — bbo-h1 Acetone /opt/topspin2.1 nmrafd 16

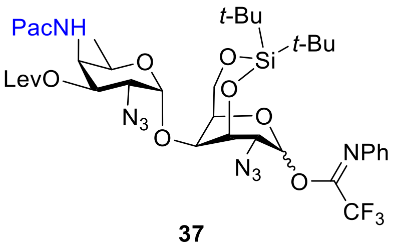

37

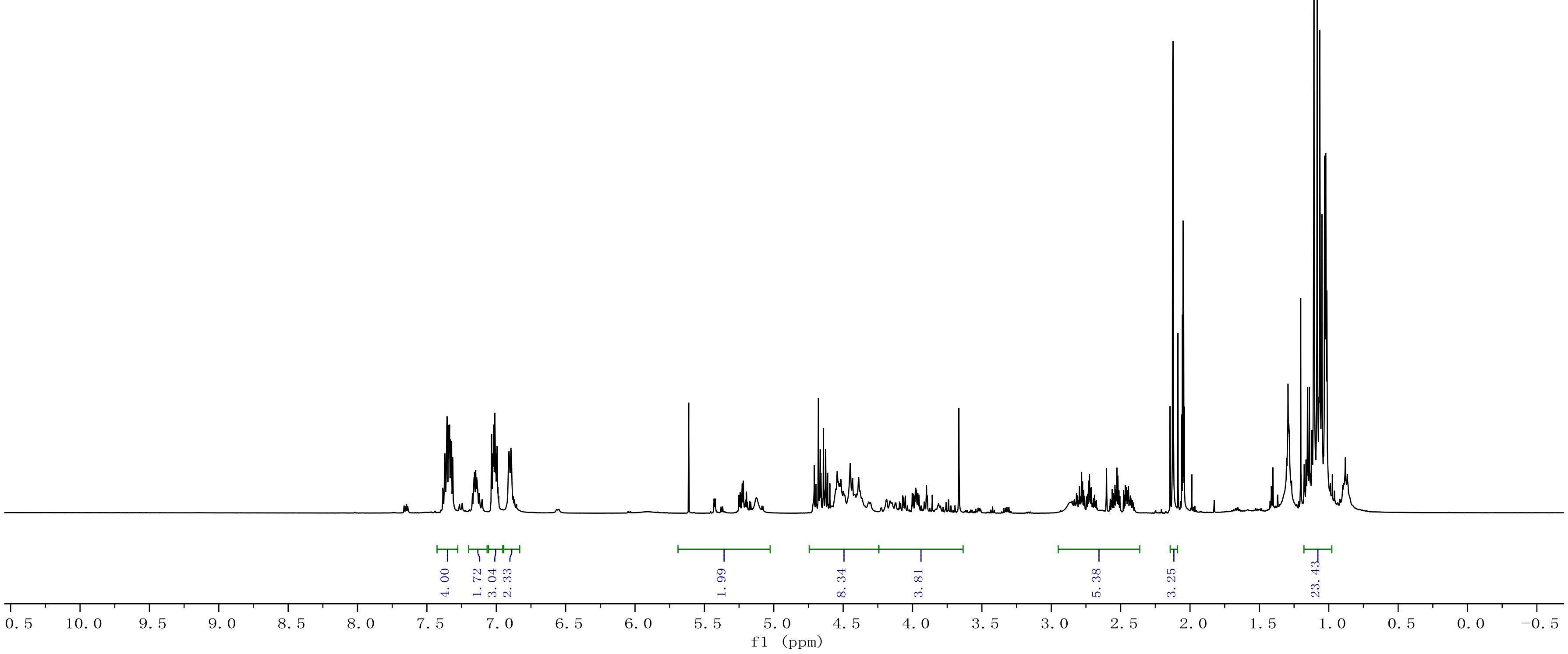

zhen2104biosyn.86.fid — wz765-A — bbo-c13-APT Acetone /opt/topspin2.1 nmrafd 16

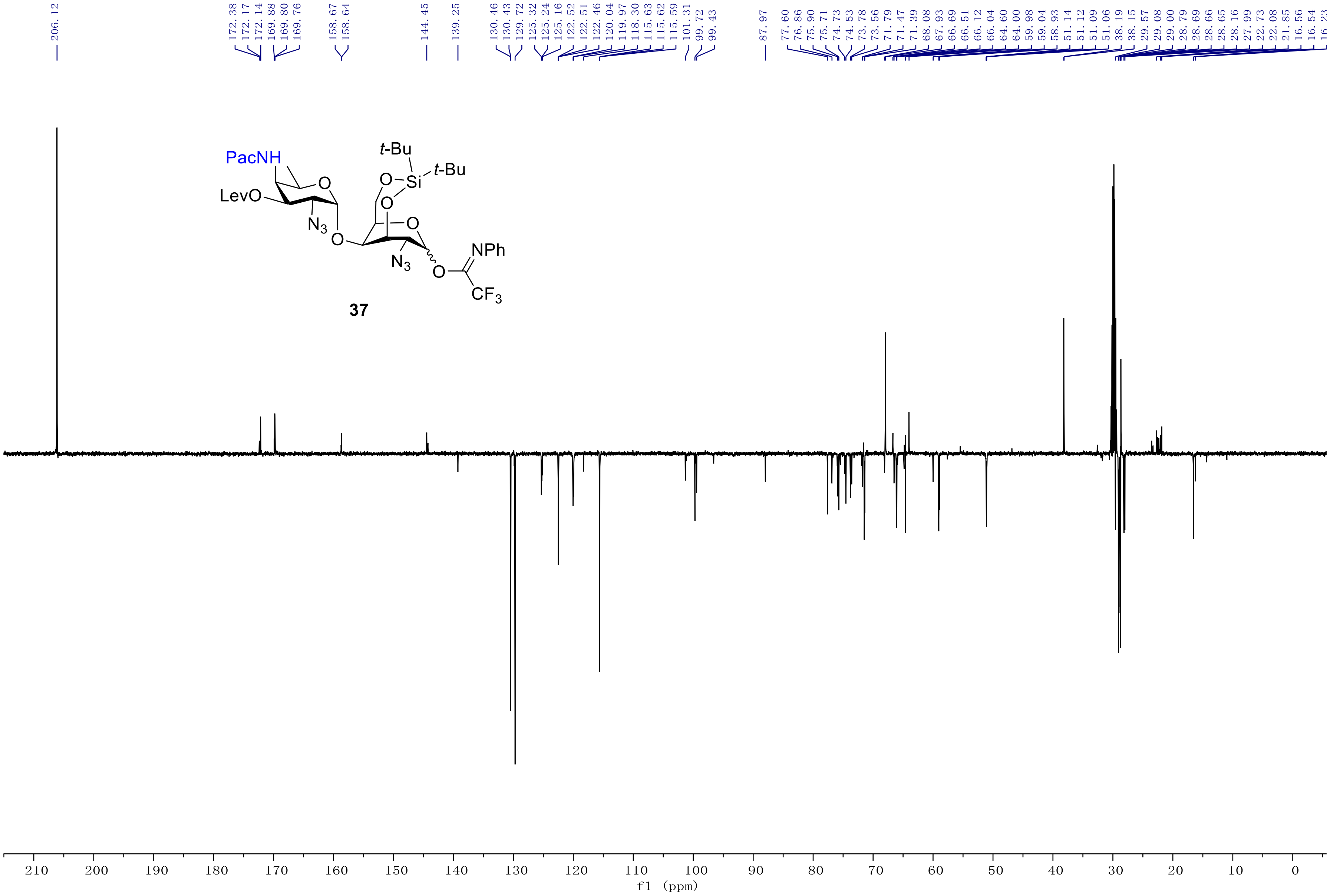

zhen2104biosyn.84.ser — wz765-A — bbo-h1-cosy Acetone /opt/topspin2.1 nmrafd 16

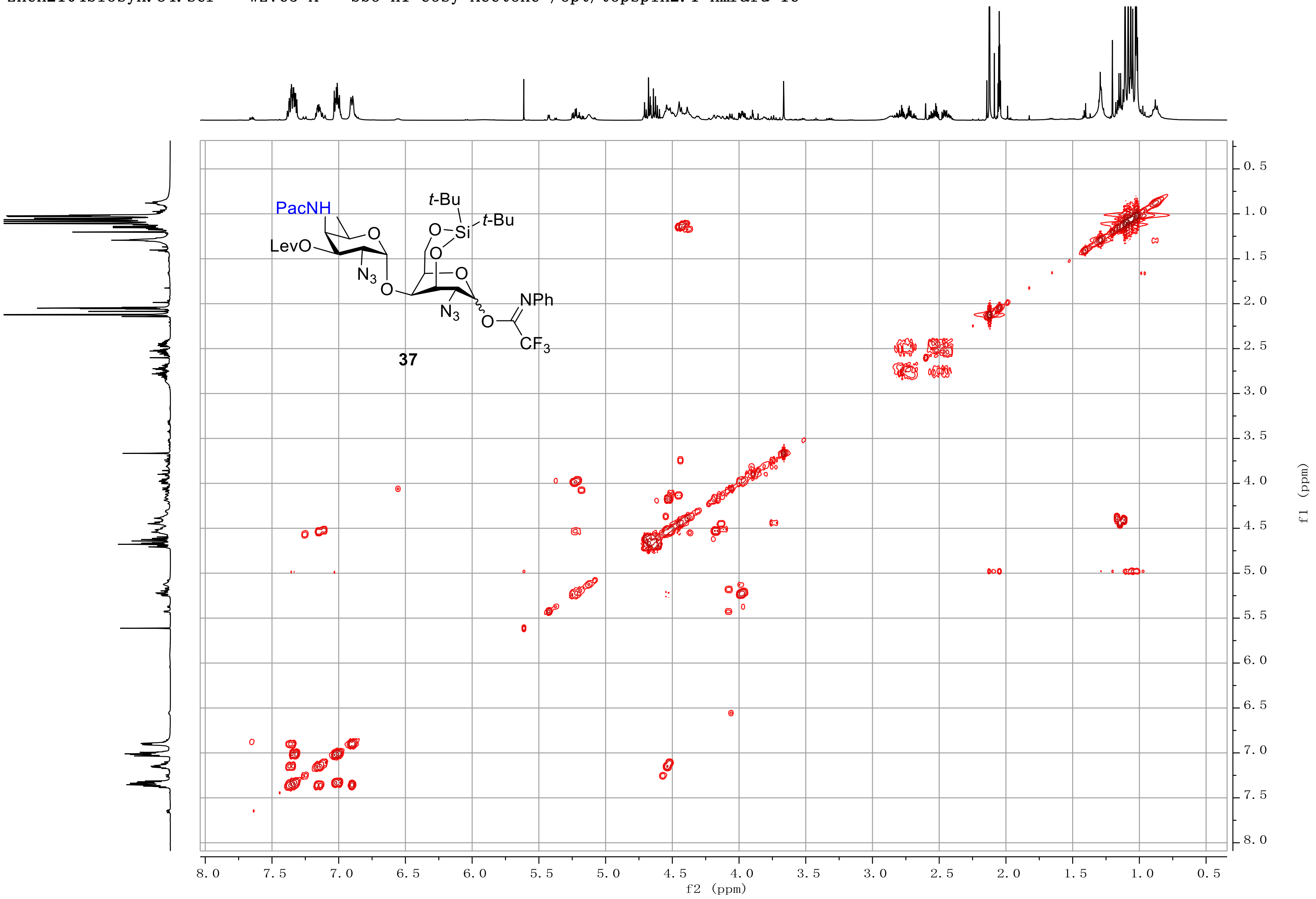

zhen2104biosyn.85.ser — wz765-A — bbo-c13-HSQC Acetone /opt/topspin2.1 nmrafd 16

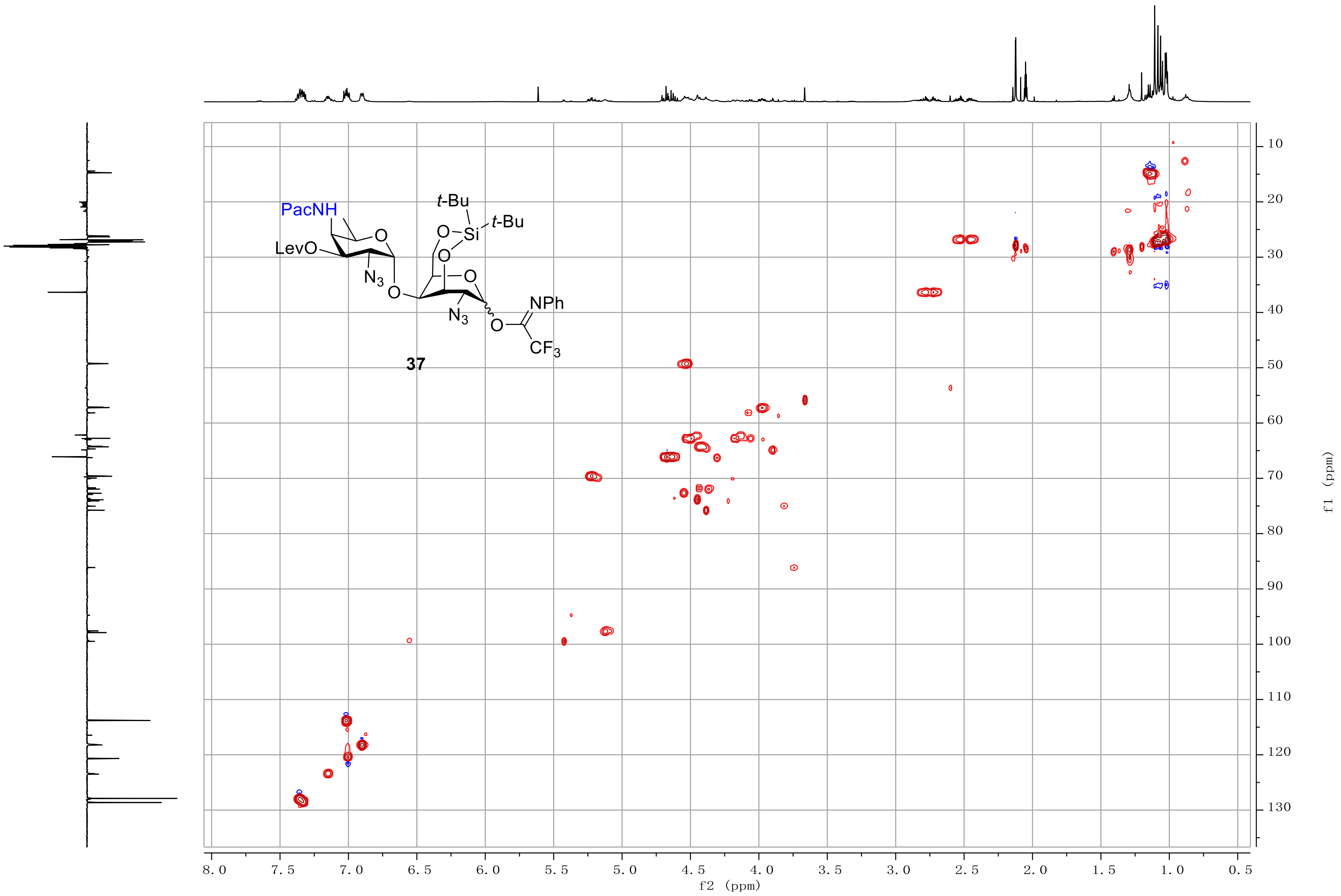

zhen2104biosyn.87.ser — wz765-A — bbo-c13-HMBC Acetone /opt/topspin2.1 nmrafd 16

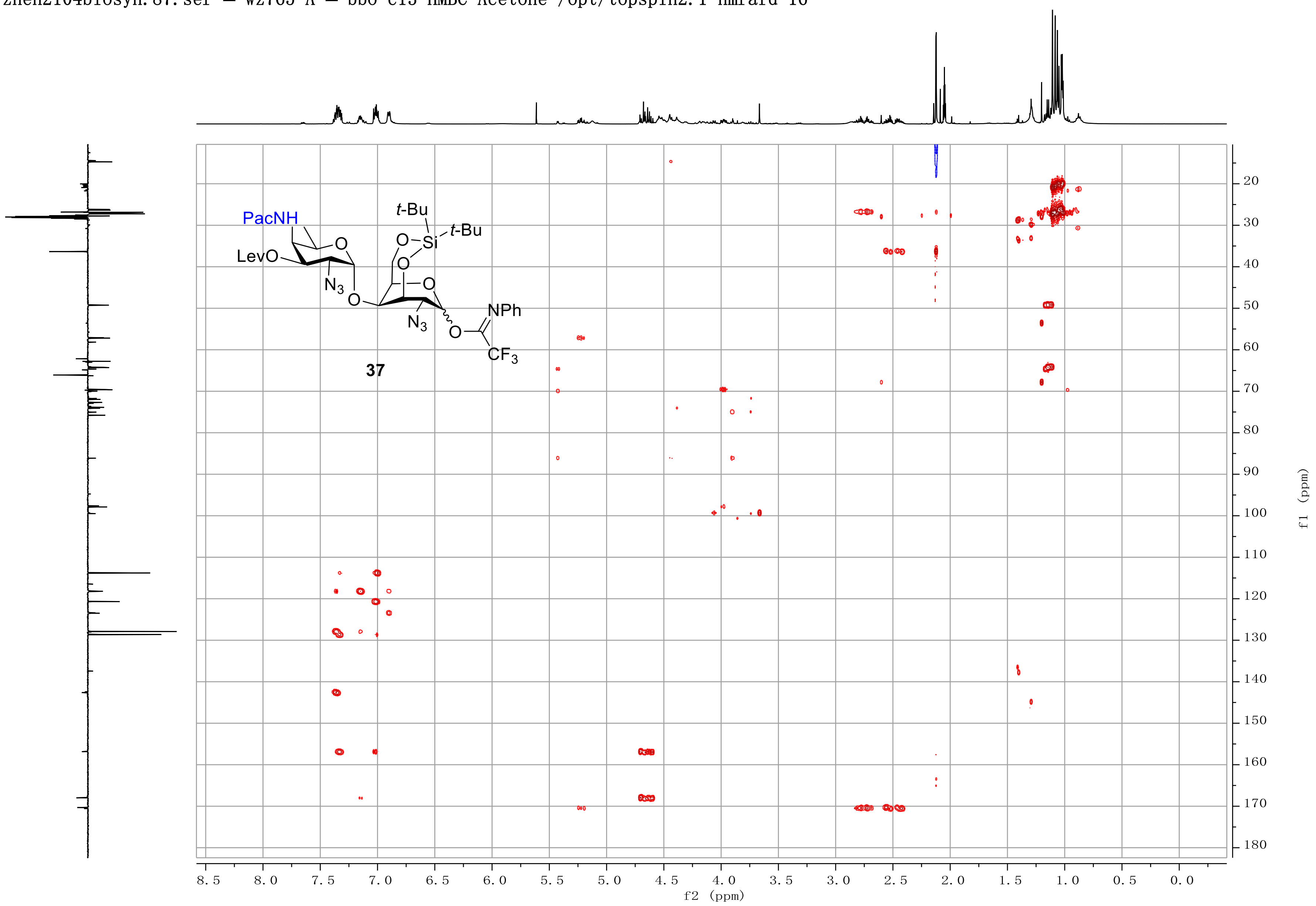

zhen2105biosyn.1.fid — wz766-B, size — bbo-h1 CDCl3 /opt/topspin2.1 nmrafd 14

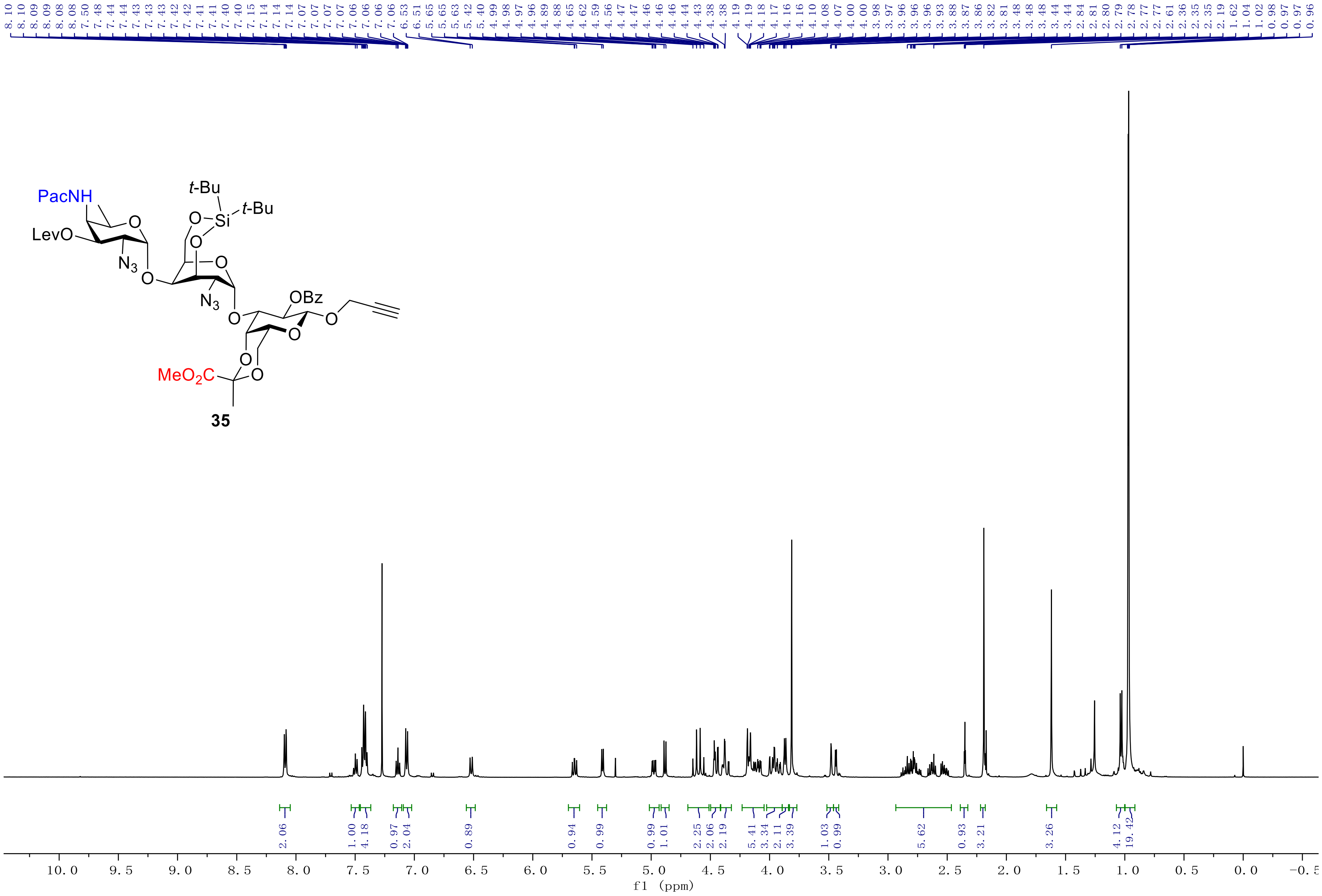

zhen2105biosyn.4.fid — wz766-B, size — bbo-c13-APT CDCl3 /opt/topspin2.1 nmrafd 14

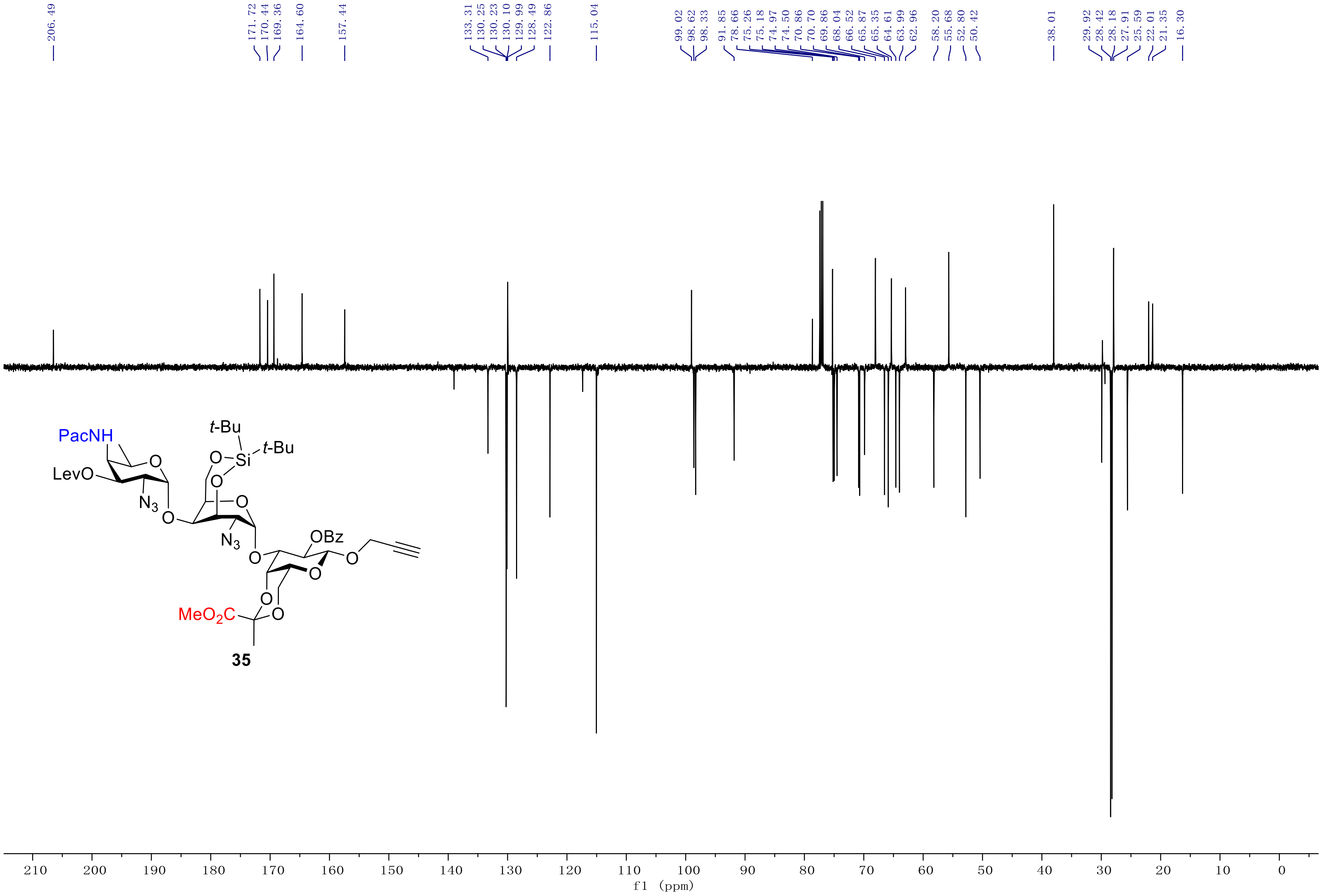

35

zhen2105biosyn.2.ser — wz766-B, size — bbo-h1-cosy CDCl3 /opt/topspin2.1 nmrafd 14

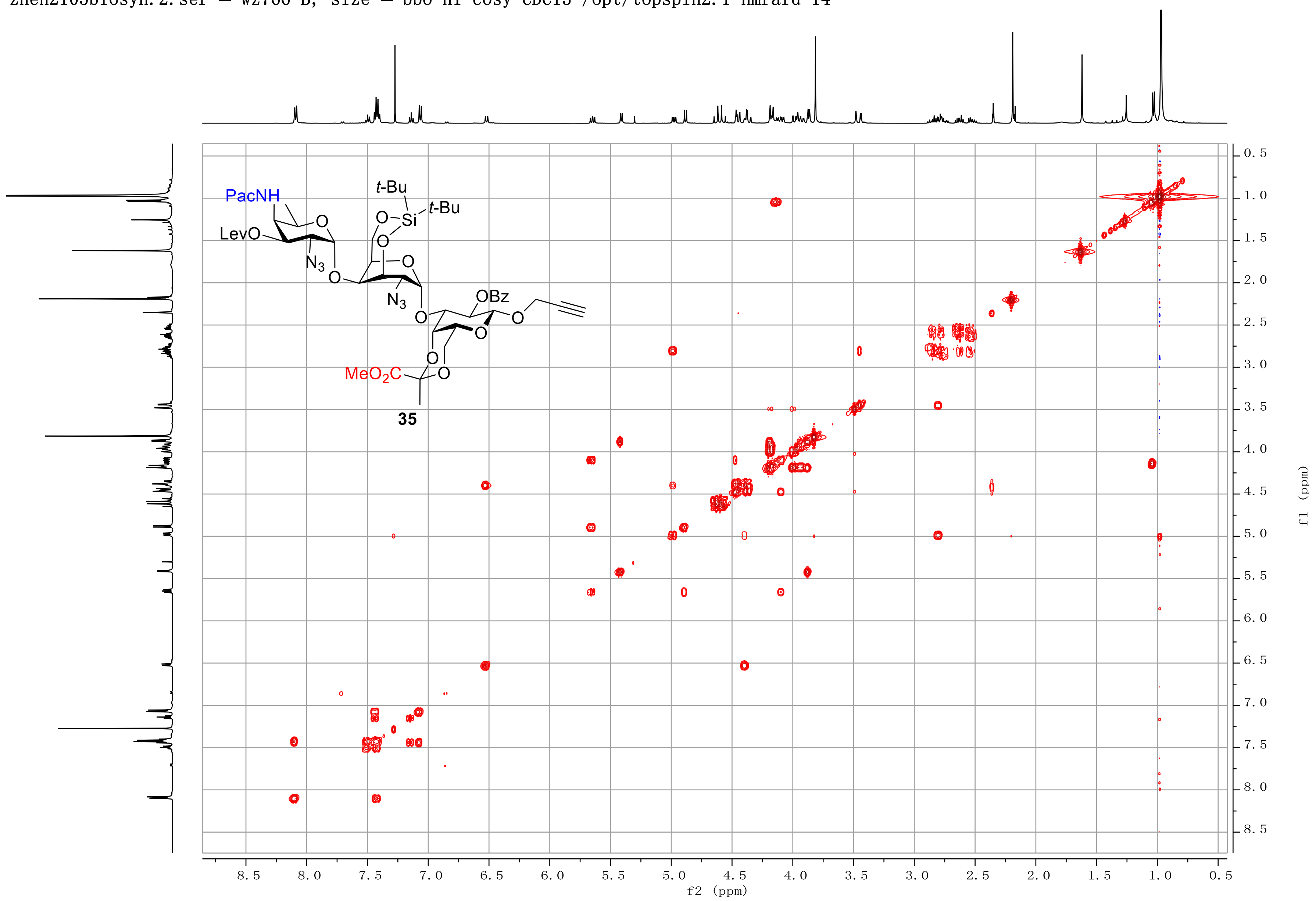

zhen2105biosyn.3.ser — wz766-B, size — bbo-c13-HSQC CDCl3 /opt/topspin2.1 nmrafd 14

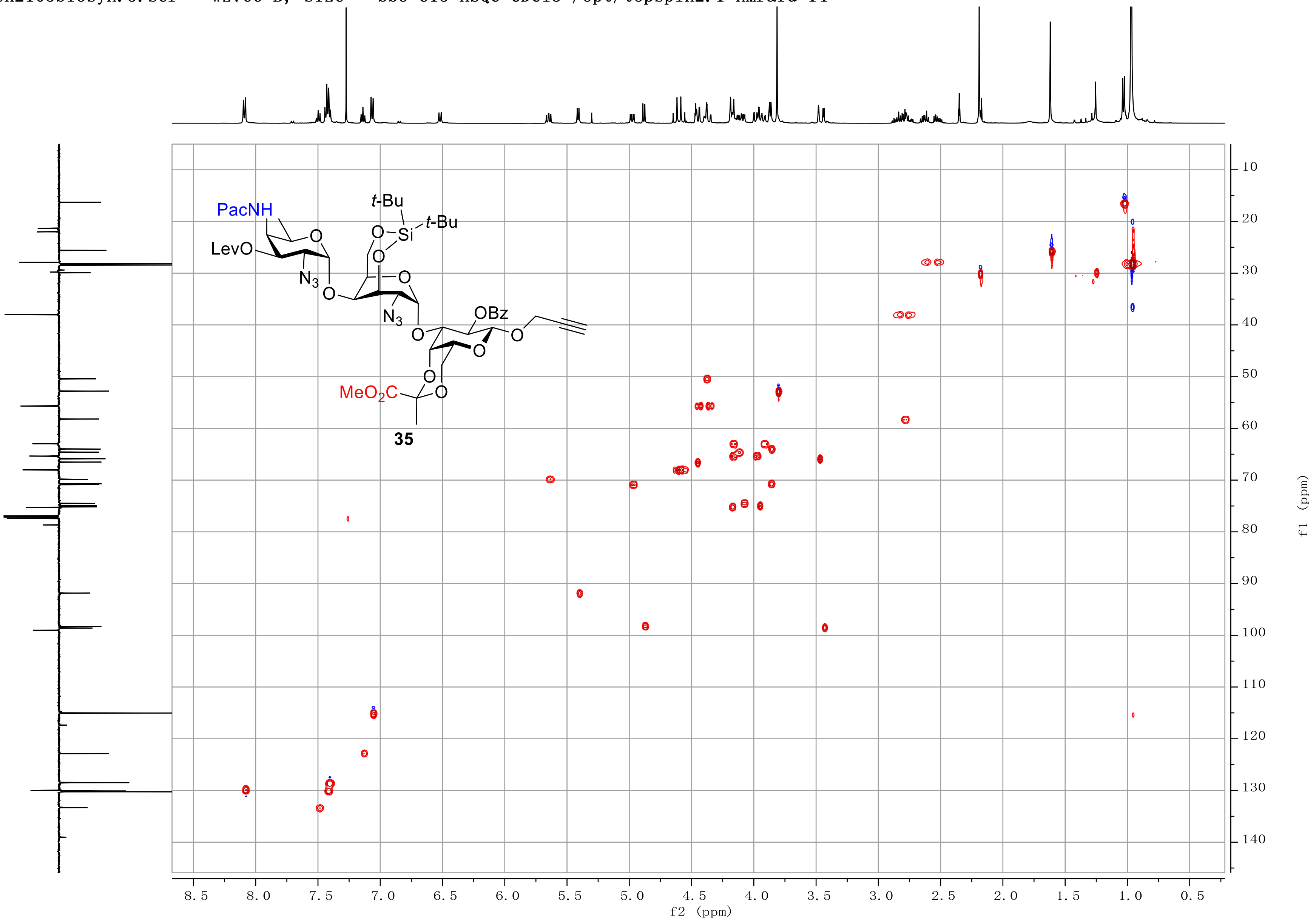

zhen2105biosyn.5.ser — wz766-B, size — bbo-c13-HMBC CDCl3 /opt/topspin2.1 nmrafd 14

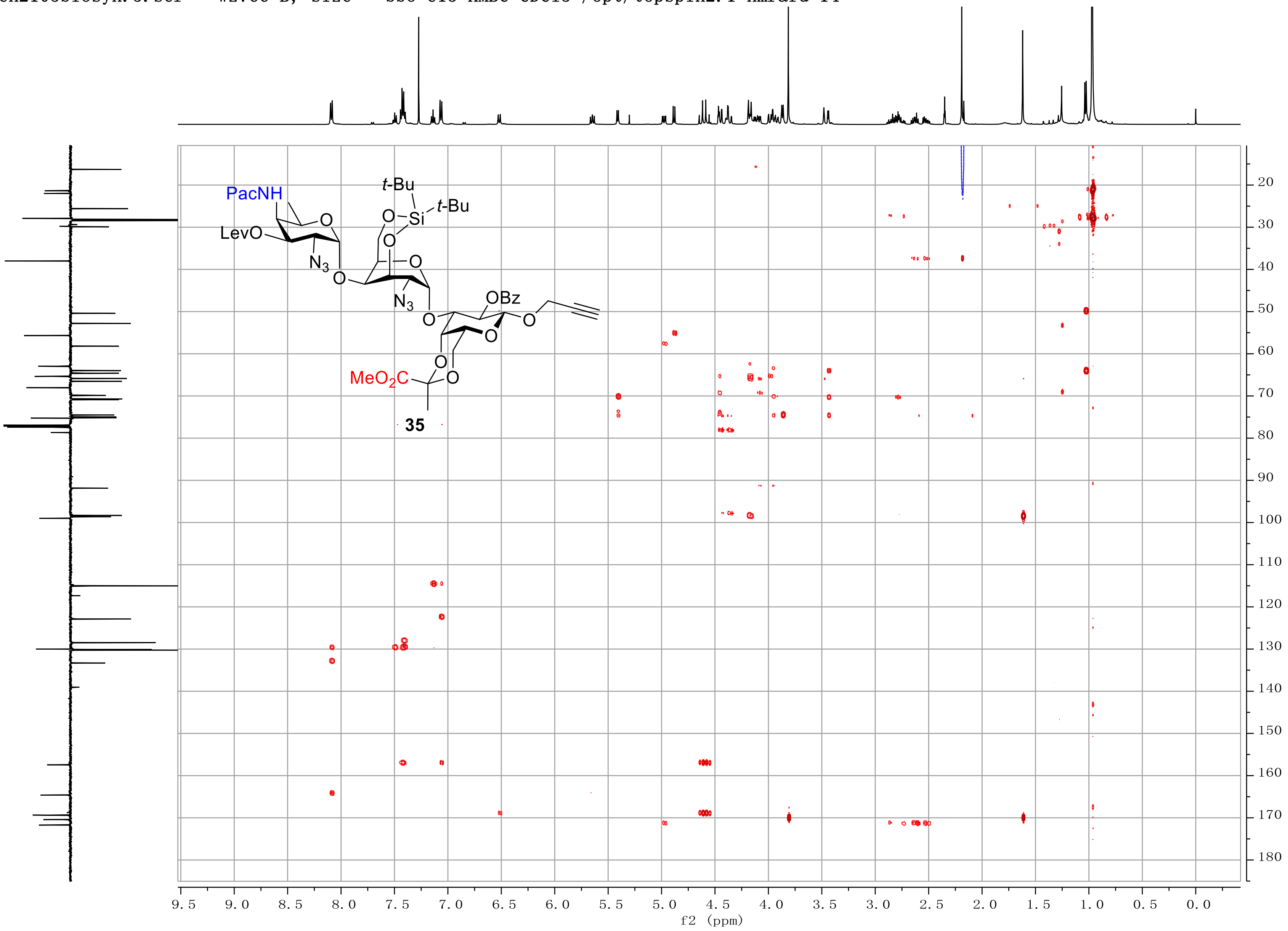

zhen2105biosyn.6.ser — wz766-B, size — bbo-c13-hmbc-ipv-gated CDCl3 /opt/topspin2.1 nmrafd 14

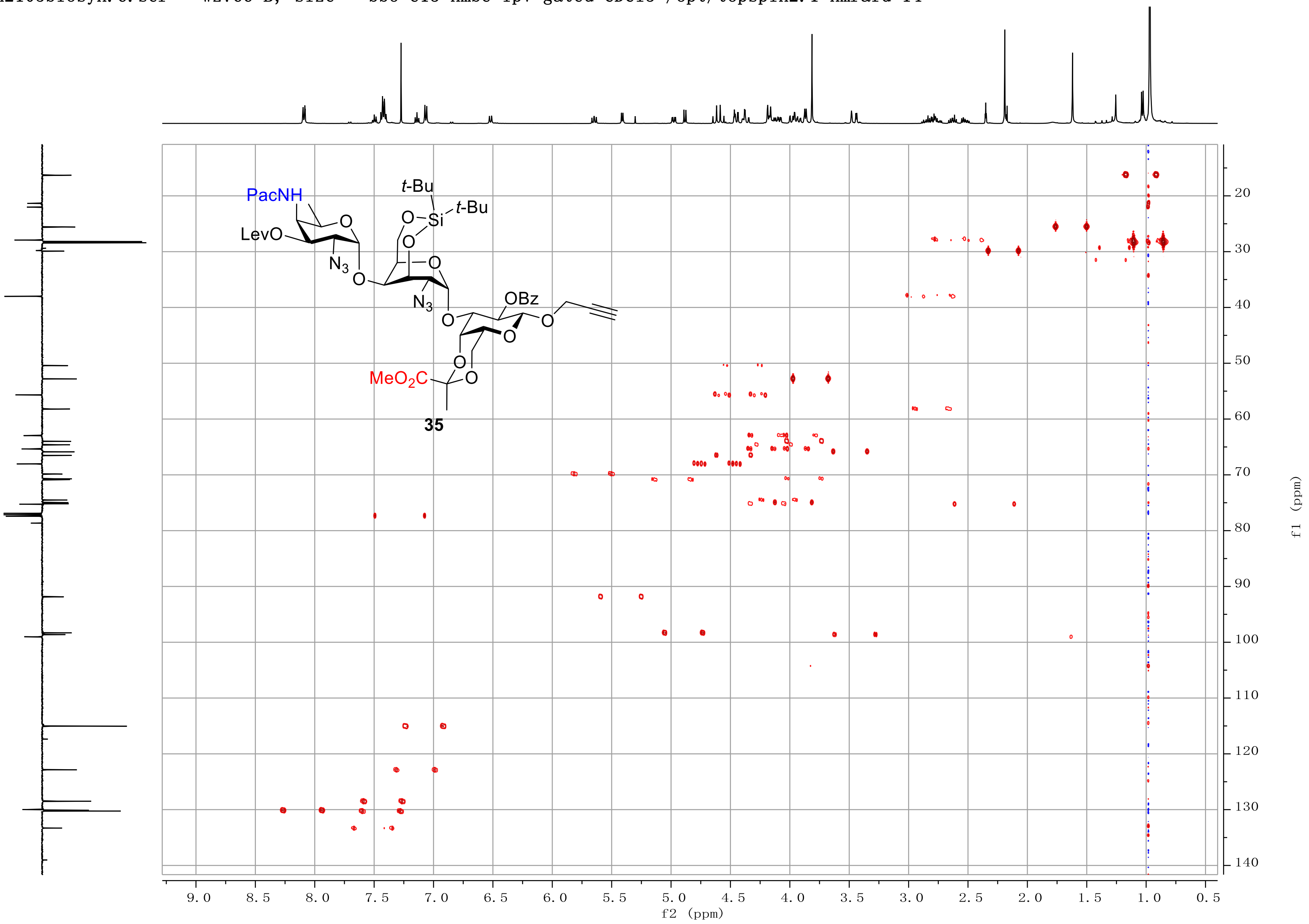

zhen2105biosyn.8.fid — wz767, CC+size — bbo-h1 CDCl3 /opt/topspin2.1 nmrafd 15

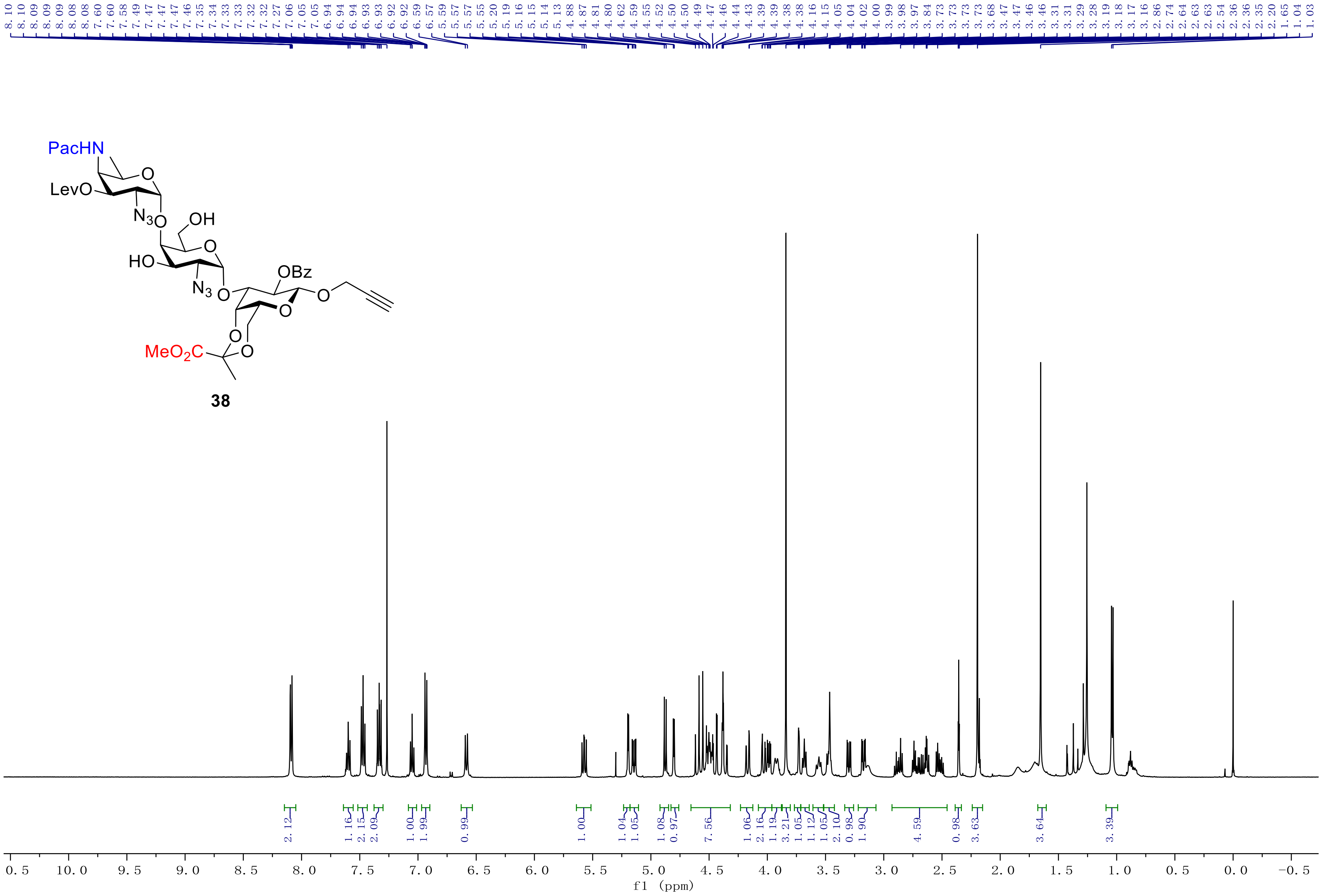

zhen2105biosyn.11.fid — wz767, CC+size — bbo-c13-APT CDCl3 /opt/topspin2.1 nmrafd 15

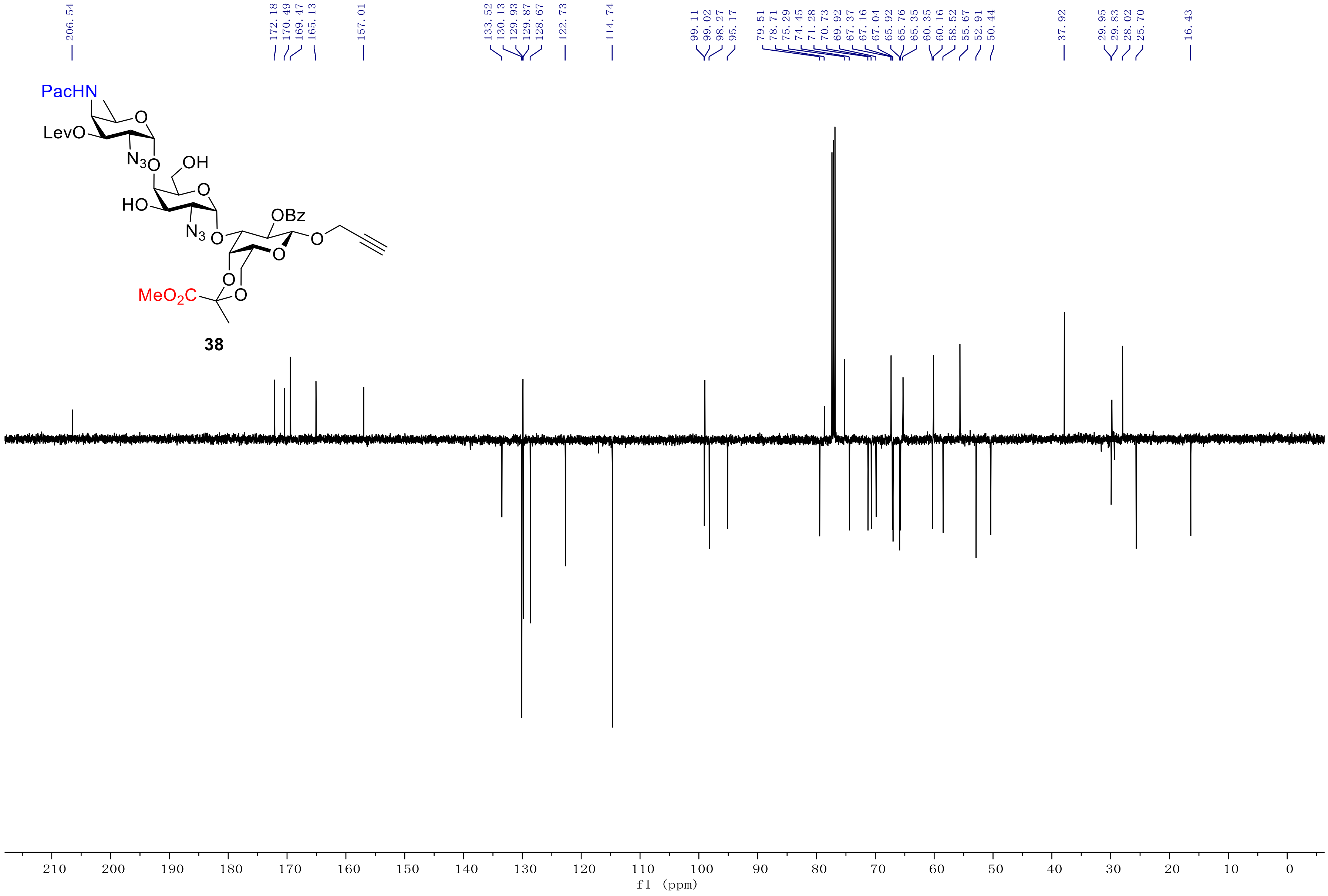

zhen2105biosyn.9.ser — wz767, CC+size — bbo-h1-cosy CDCl3 /opt/topspin2.1 nmrafd 15

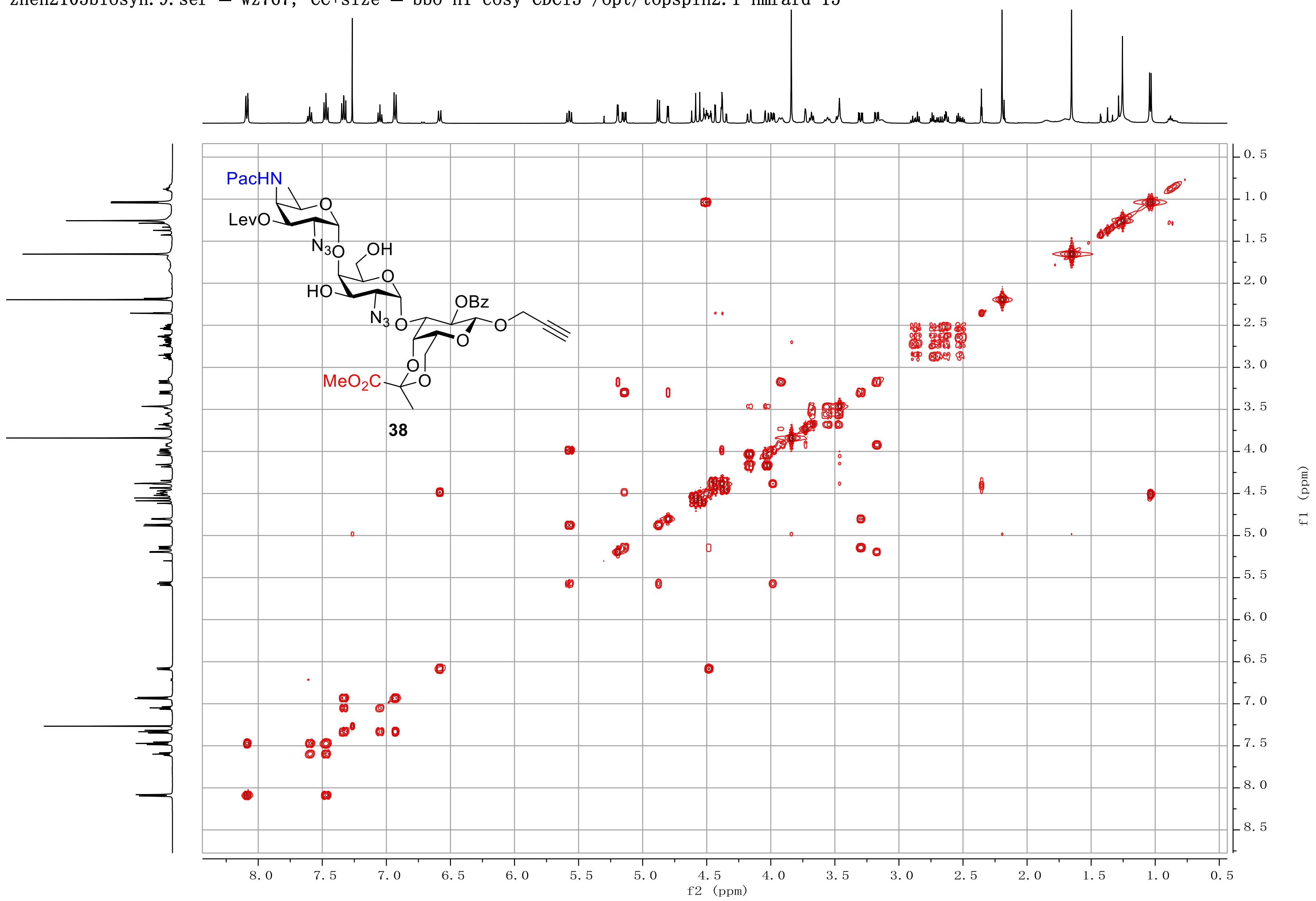

zhen2105biosyn.10.ser — wz767, CC+size — bbo-c13-HSQC CDCl3 /opt/topspin2.1 nmrafd 15

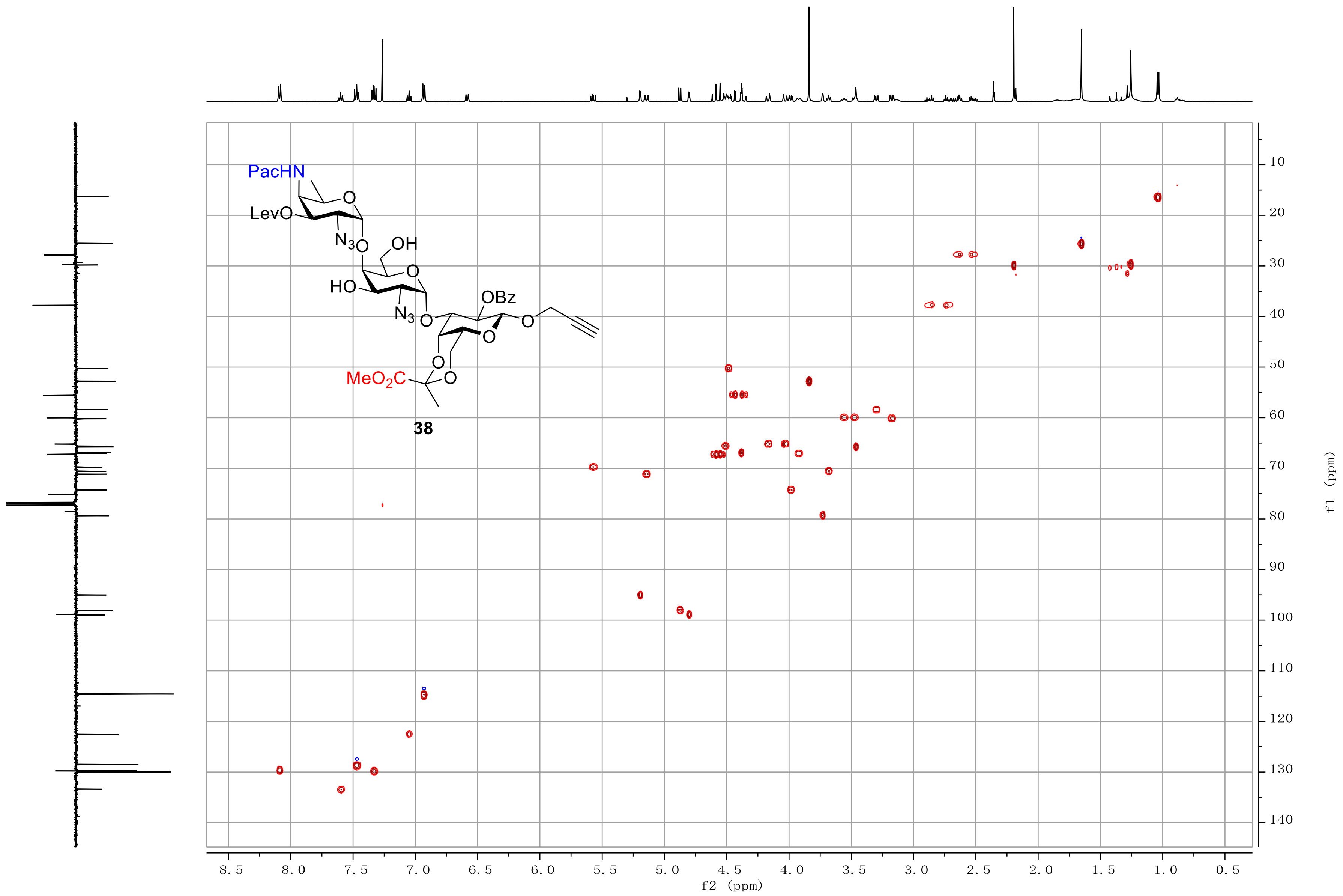

zhen2105biosyn.12.ser — wz767, CC+size — bbo-c13-HMBC CDCl3 /opt/topspin2.1 nmrafd 15

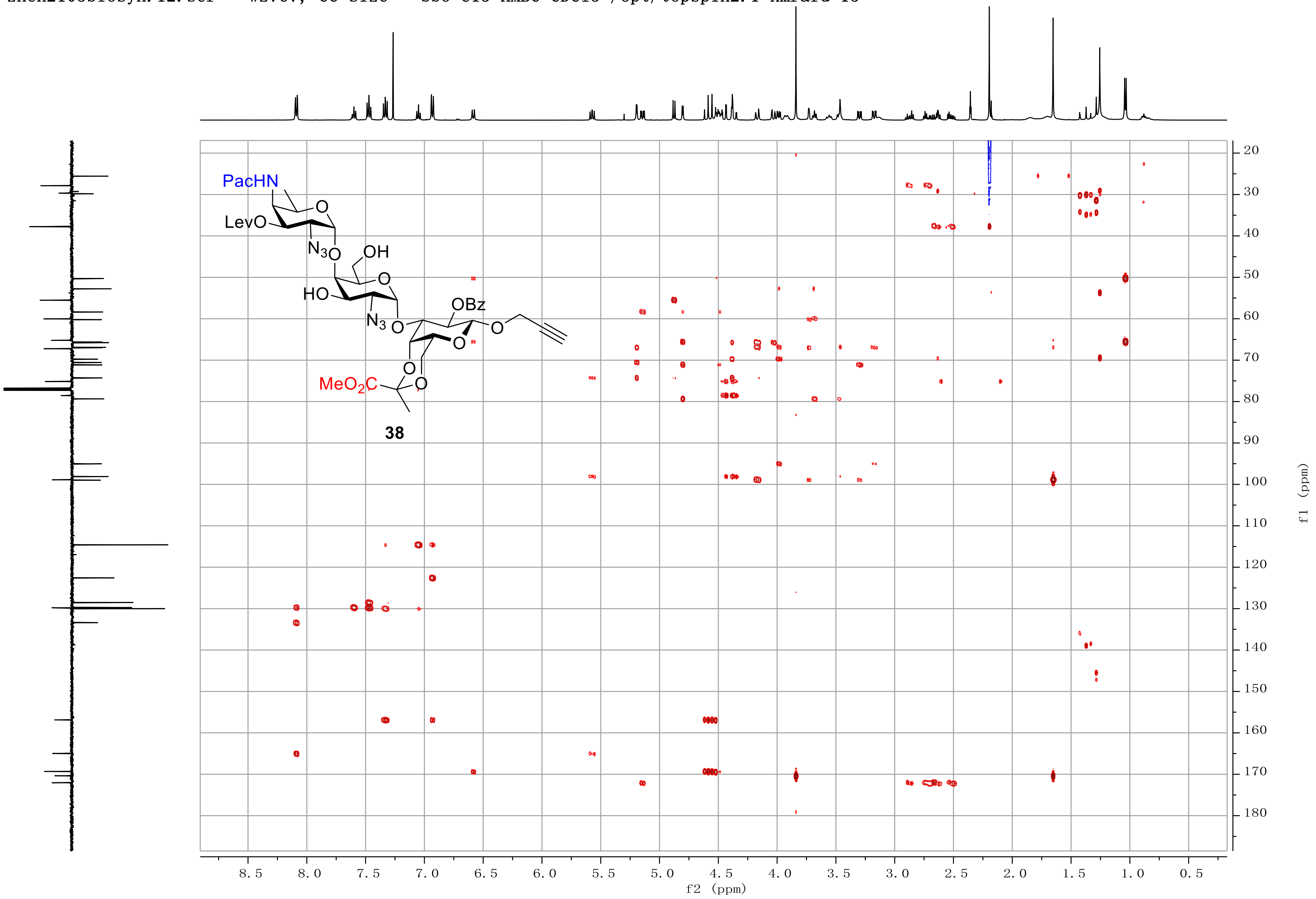

zhen2105biosyn.13.ser — wz767, CC+size — bbo-c13-hmbc-ipv-gated CDCl3 /opt/topspin2.1 nmrafd 15

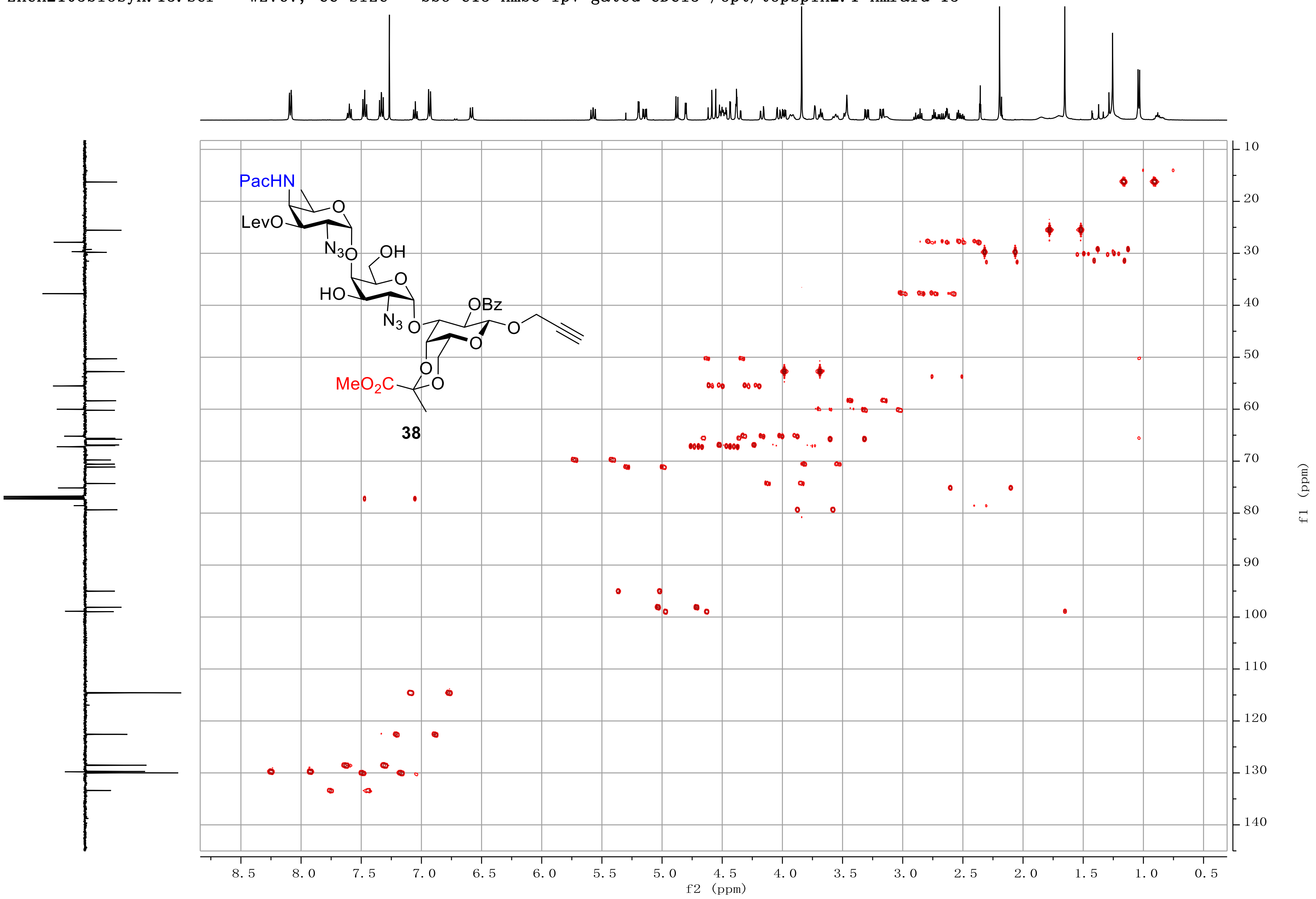

zhen2105biosyn.35.fid — wz769-A, size — bbo-h1 CDCl3 /opt/topspin2.1 nmrafd 2

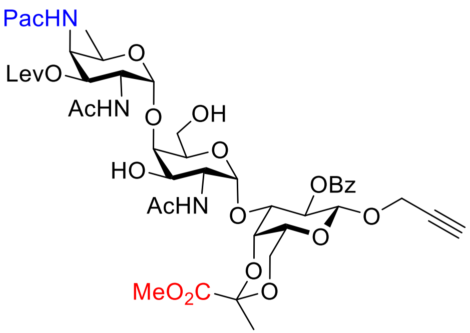

39

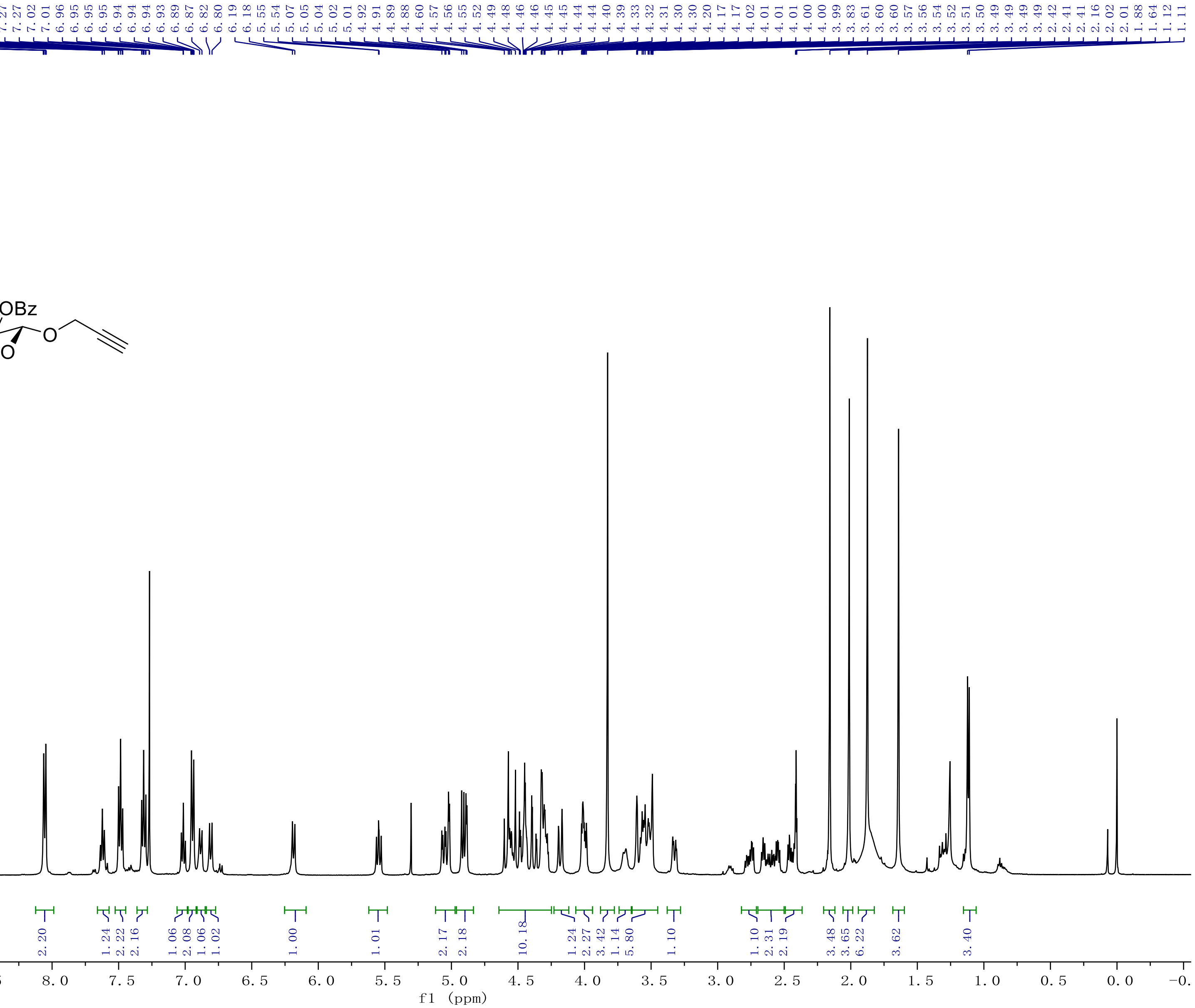

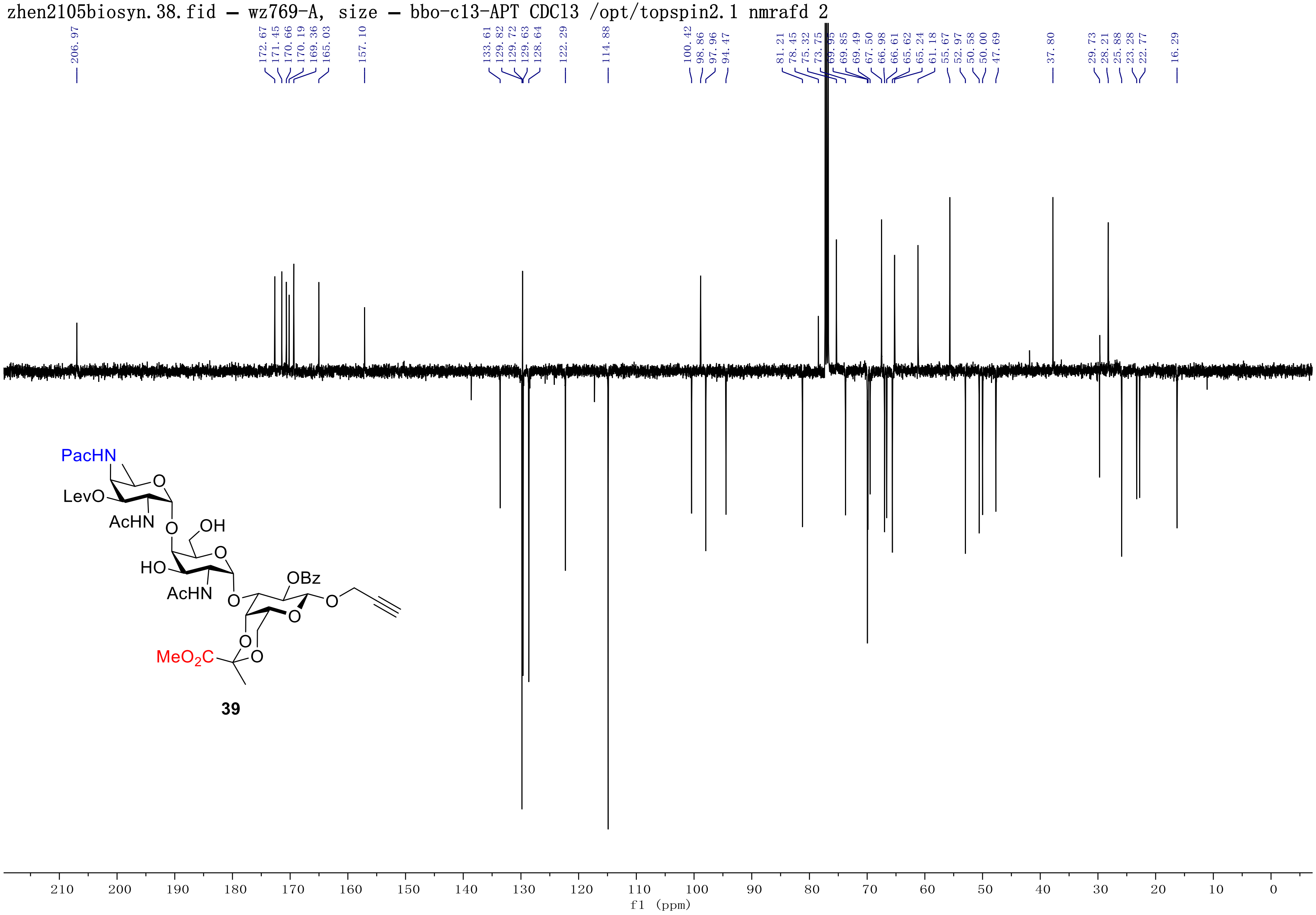
zhen2105biosyn.38.fid — wz769-A, size — bbo-c13-APT CDCl3 /opt/topspin2.1 nmrafd 2
206.97
172.67
171.45
170.66
170.19
169.36
165.03
157.10
133.61
129.82
129.72
129.63
128.64
122.29
114.88
100.42
98.86
97.96
94.47
81.21
78.45
75.32
73.75
69.95
69.85
69.49
67.50
66.98
66.61
65.62
65.24
61.18
55.67
52.97
50.58
50.00
47.69
37.80
29.73
28.21
25.88
23.28
22.77
16.29
210 200 190 180 170 160 150 140 130 120 110 100 90 80 70 60 50 40 30 20 10 0
f1 (ppm)
PacHN
LevO
AcHN
OH
HO
AcHN
OBz
MeO2C

39

zhen2105biosyn.36.ser — wz769-A, size — bbo-h1-cosy CDCl3 /opt/topspin2.1 nmrafd 2

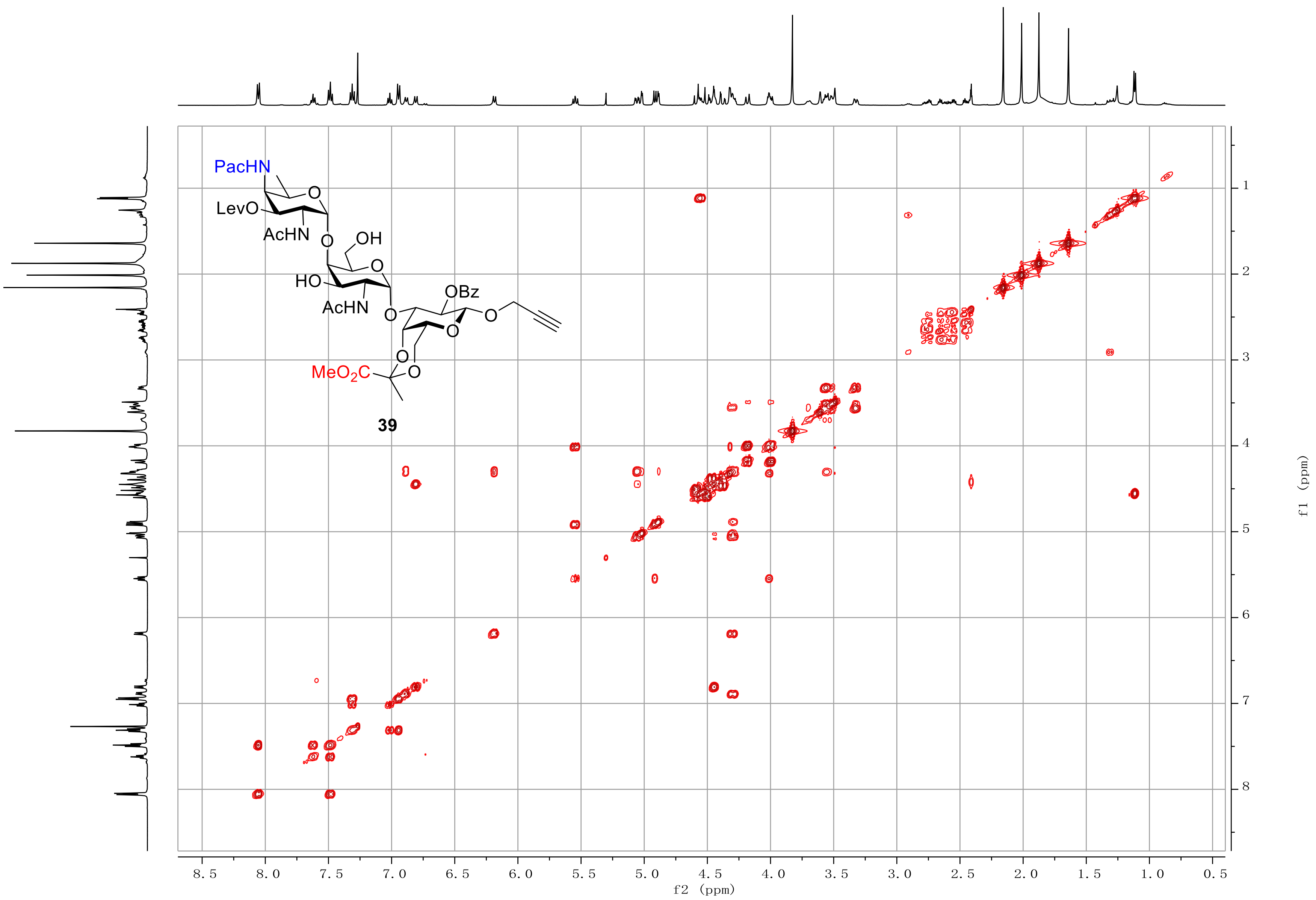

zhen2105biosyn.37.ser — wz769-A, size — bbo-c13-HSQC CDCl3 /opt/topspin2.1 nmrafd 2

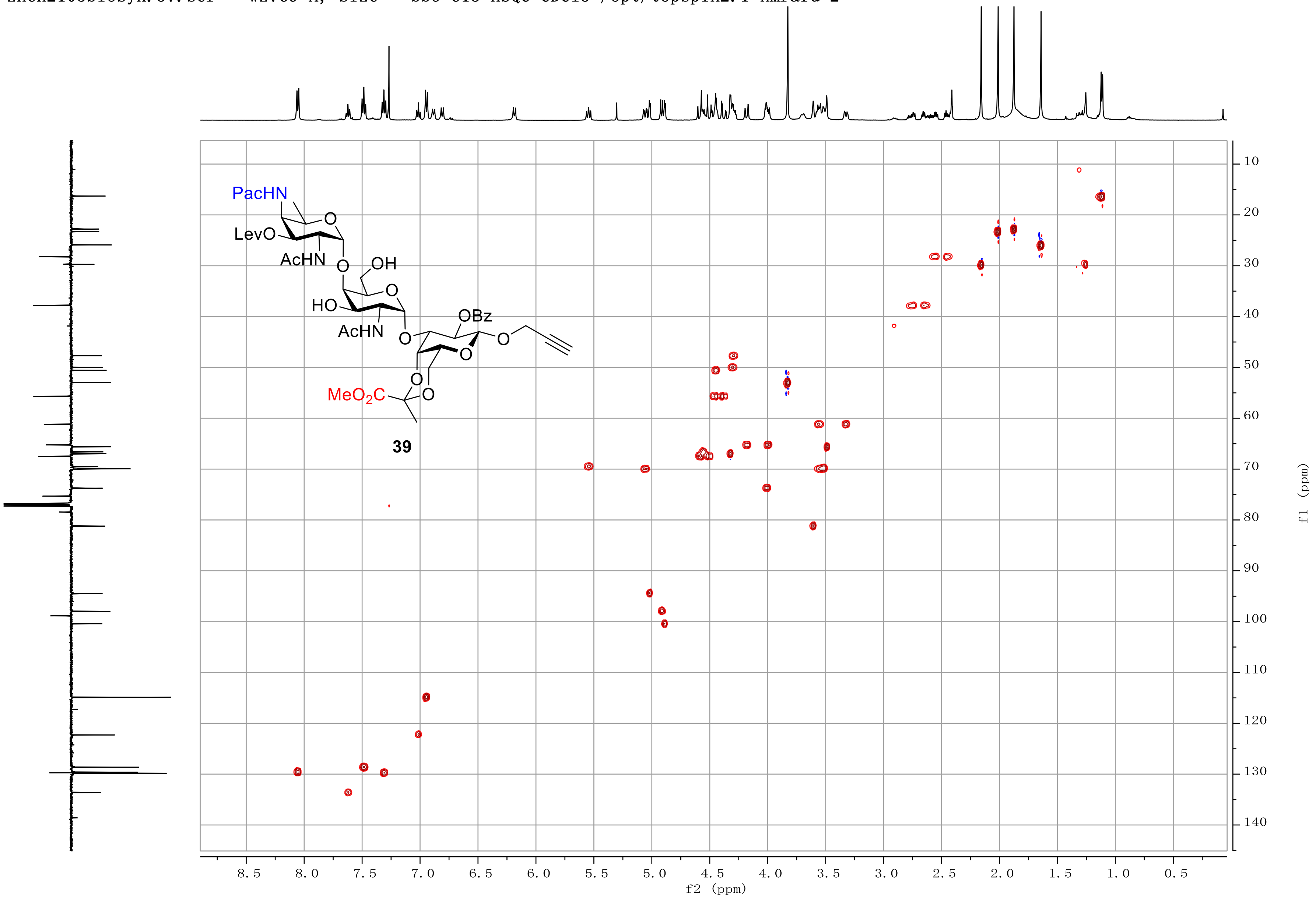

zhen2105biosyn.39.ser — wz769-A, size — bbo-c13-HMBC CDCl3 /opt/topspin2.1 nmrafd 2

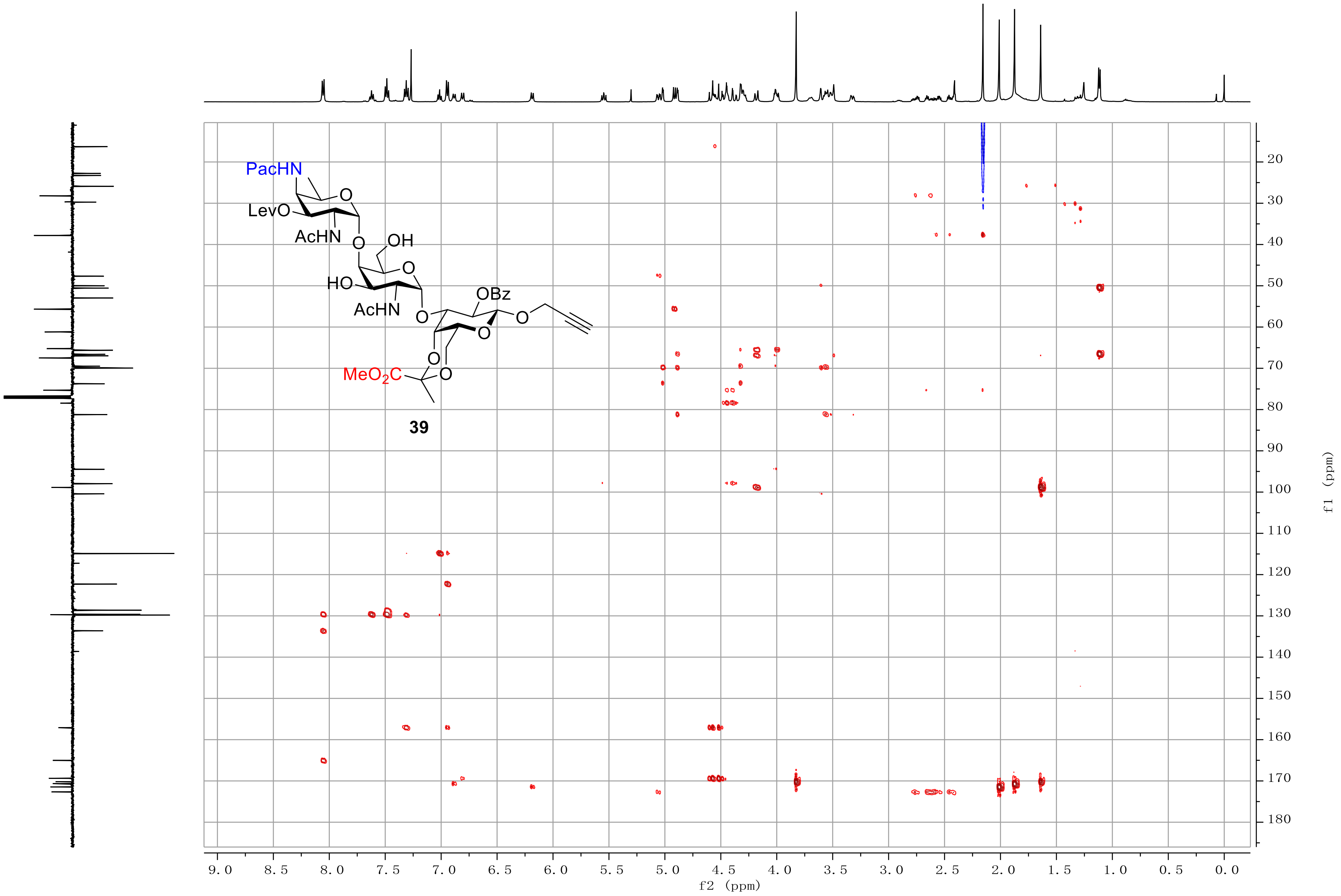

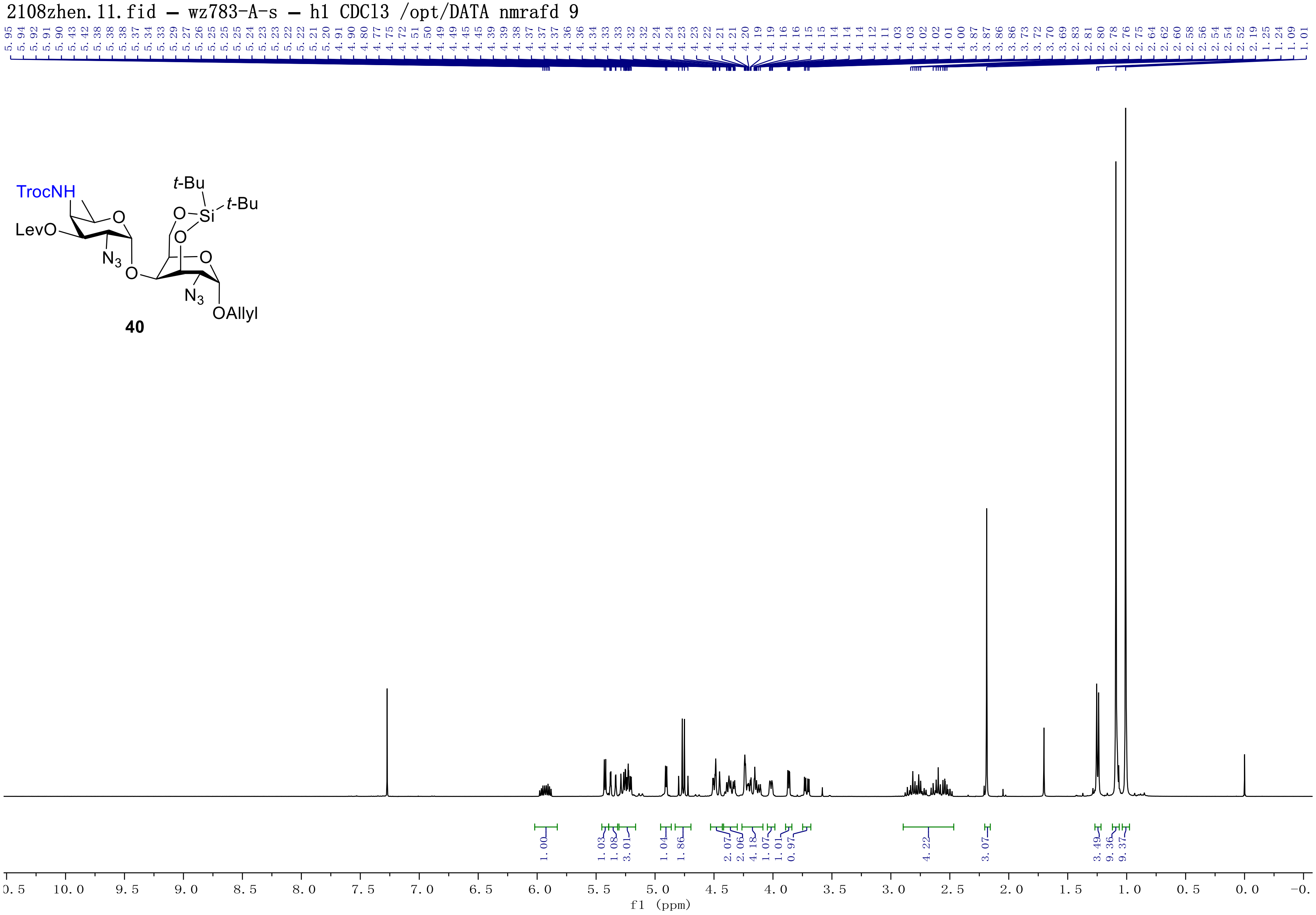
2108zhen.11.fid — wz783-A-s — h1 CDCl3 /opt/DATA nmrafd 9
TrocNH
LevO
t-Bu
t-Bu
Si
OAllyl
40
f1 (ppm)

2108zhen.12.fid — wz783-A-s — C13APT CDCl3 /opt/DATA nmrafd 9

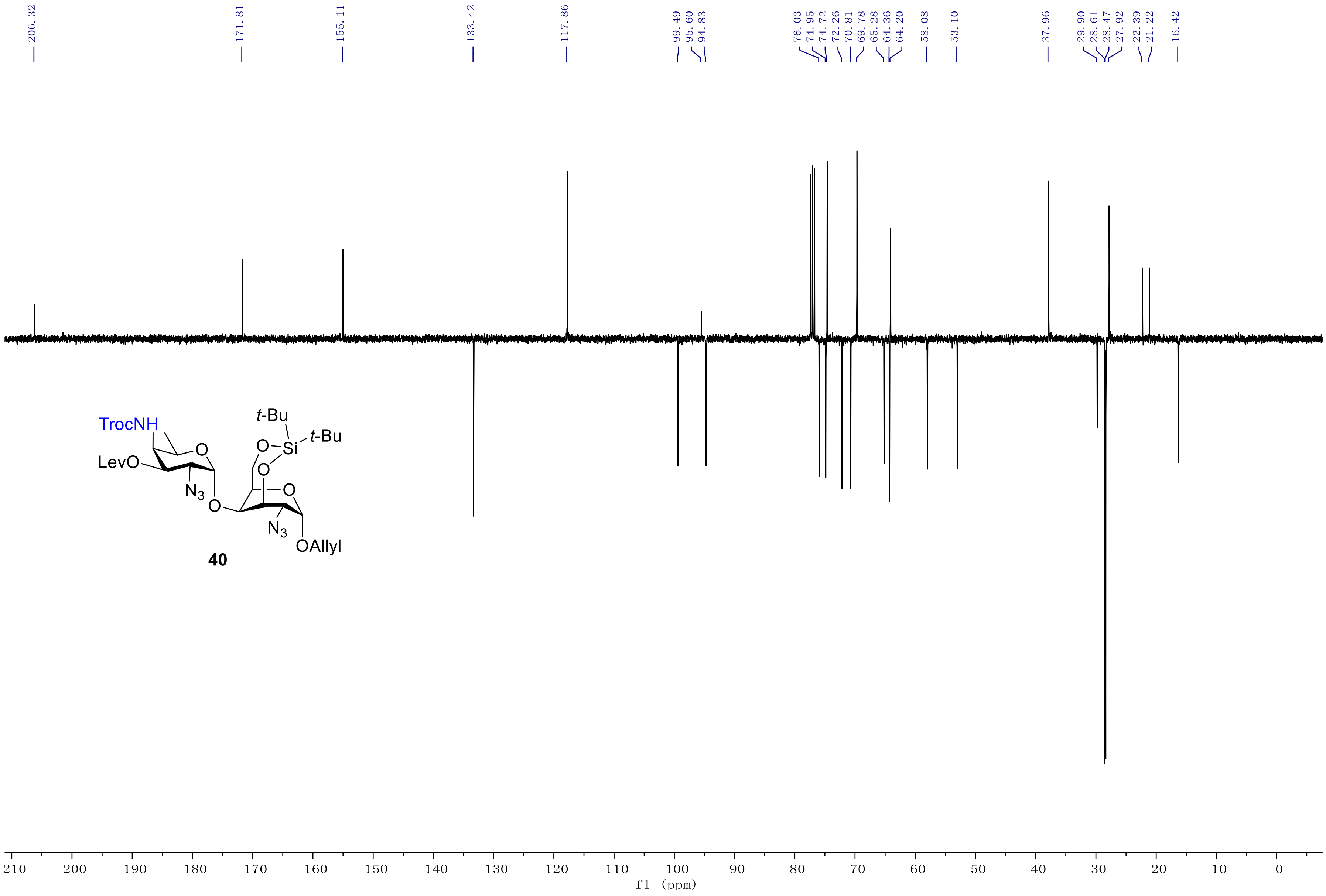

2108zhen.13.ser — wz783-A-s — h1COSY CDCl3 /opt/DATA nmrafd 9

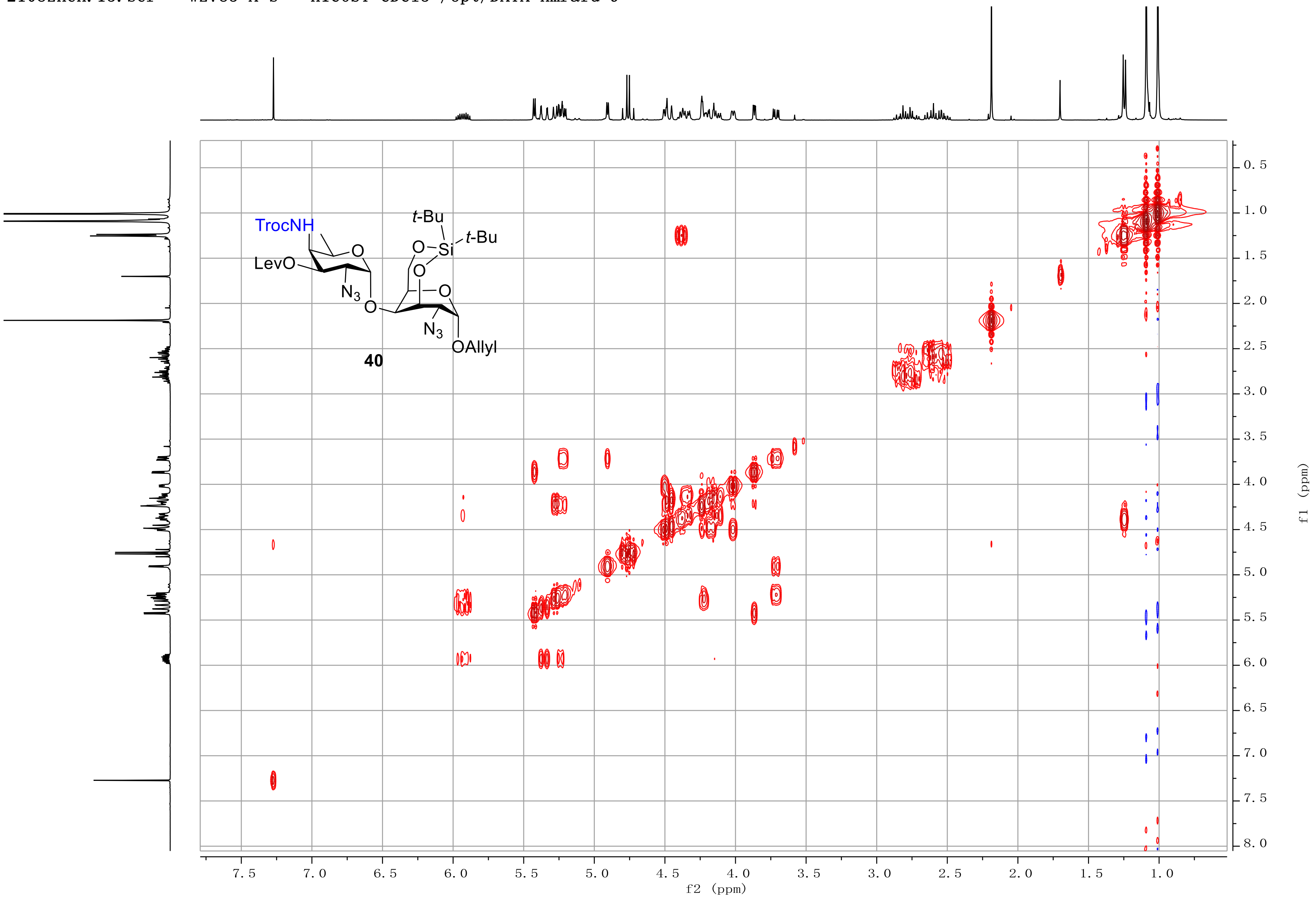

2108zhen.14.ser — wz783-A-s — c13HSQC CDCl3 /opt/DATA nmrafd 9

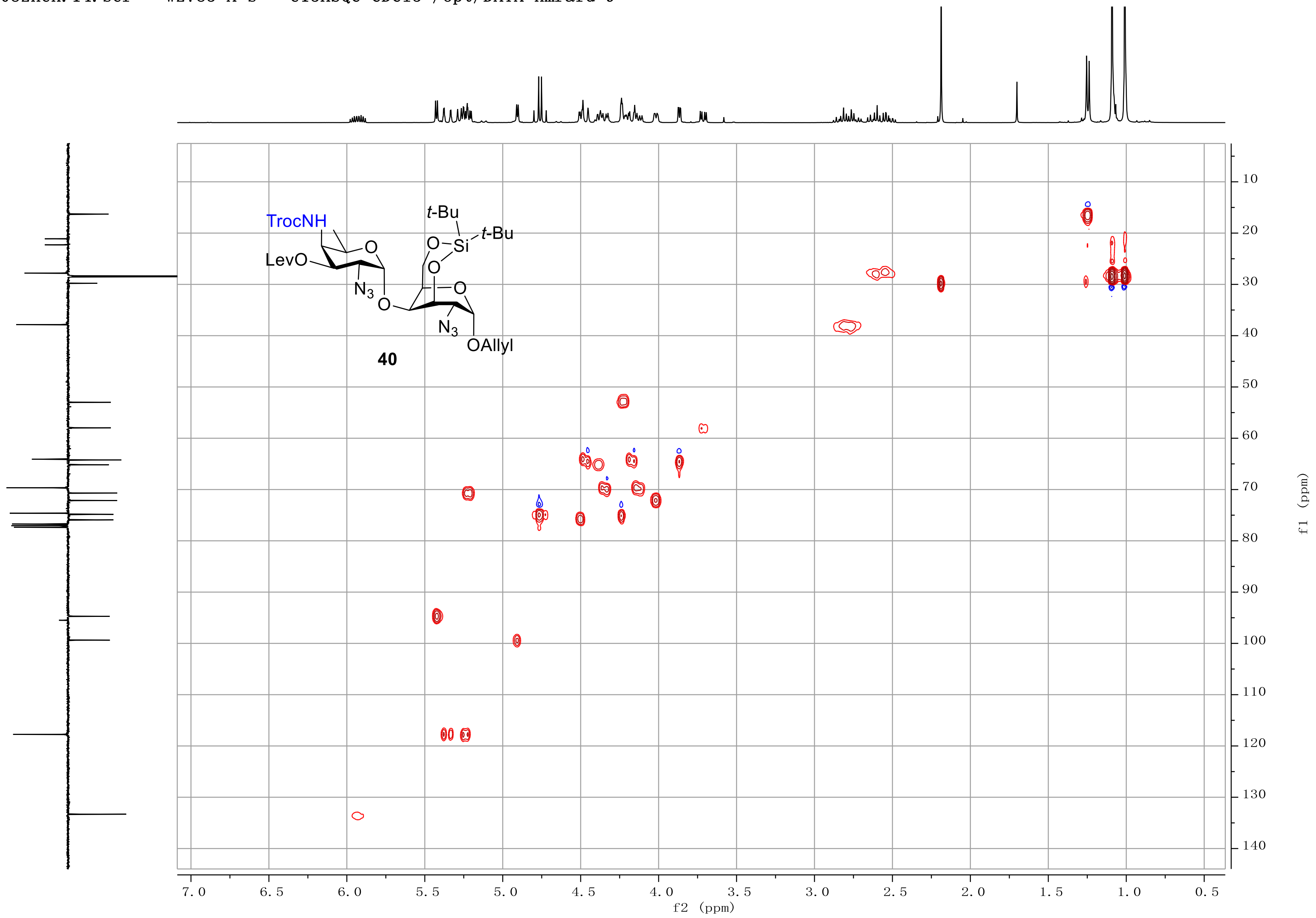

2108zhen.15.ser — wz783-A-s — c13HMBC CDCl3 /opt/DATA nmrafd 9

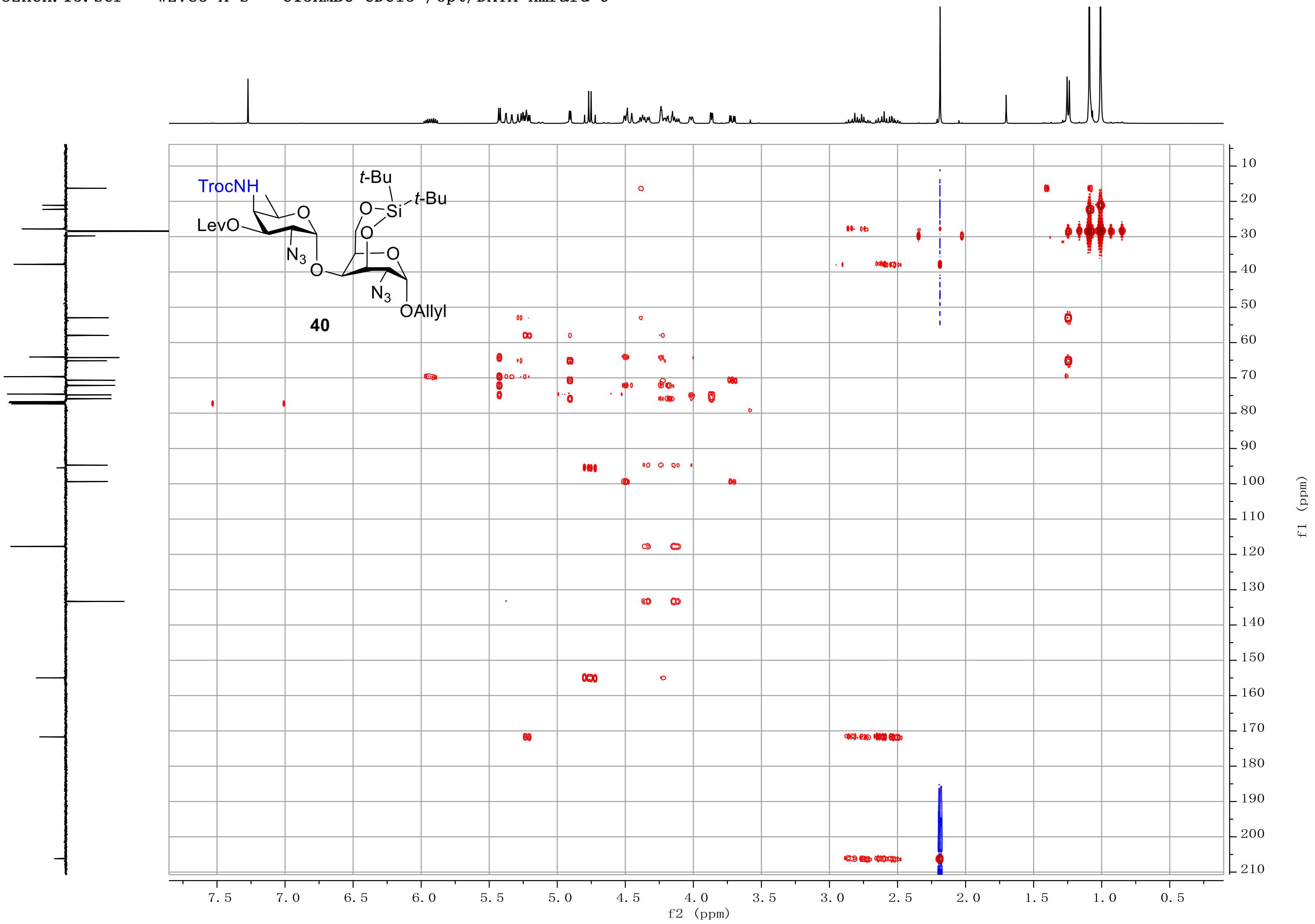

2108zhen.16.ser — wz783-A-s — c13HMBCipvGATED CDCl3 /opt/DATA nmrafd 9

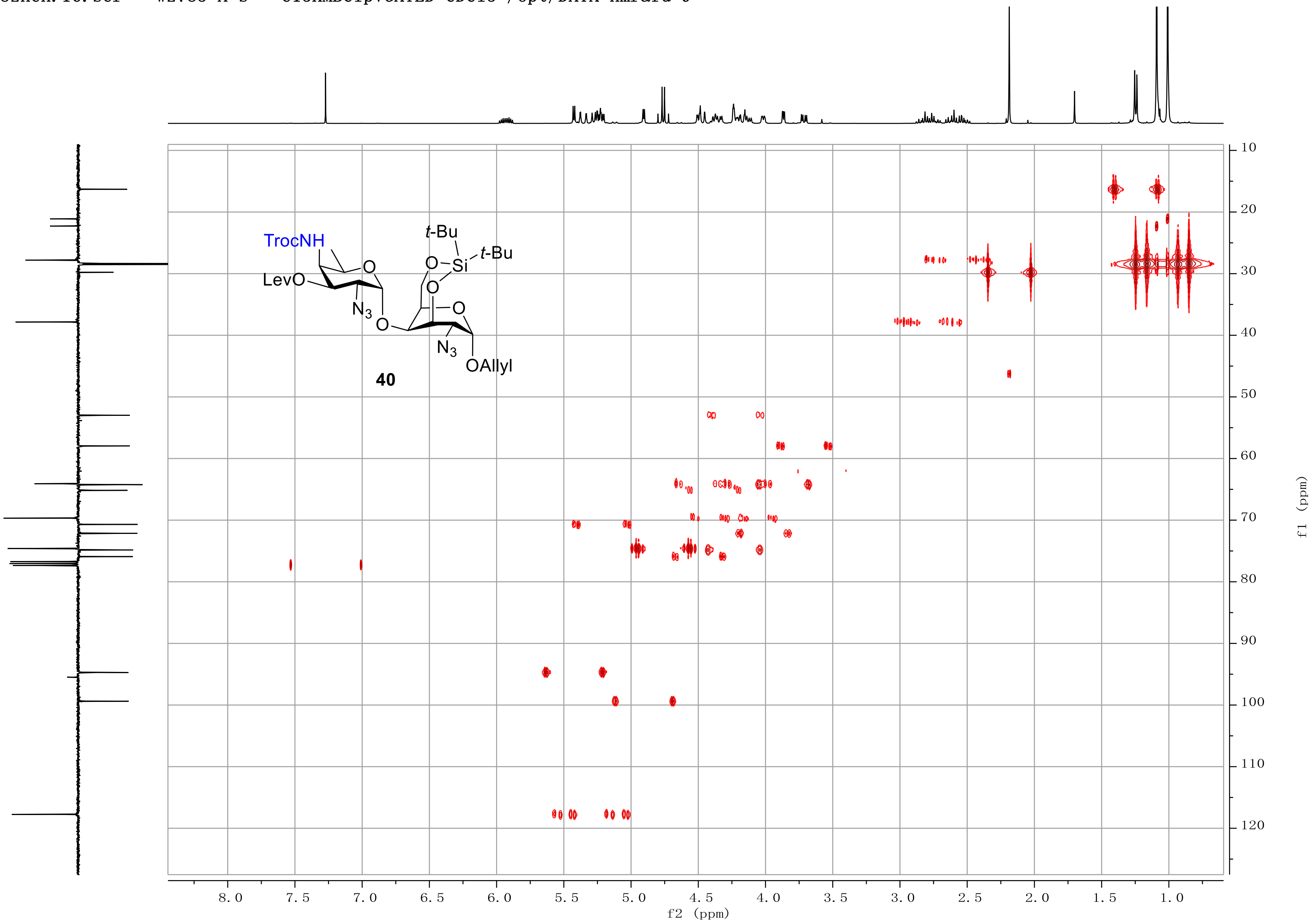

zhen2108biosyn.39.fid — wz784 — bbo-h1 CDCl3 /opt/topspin2.1 nmrafd 2

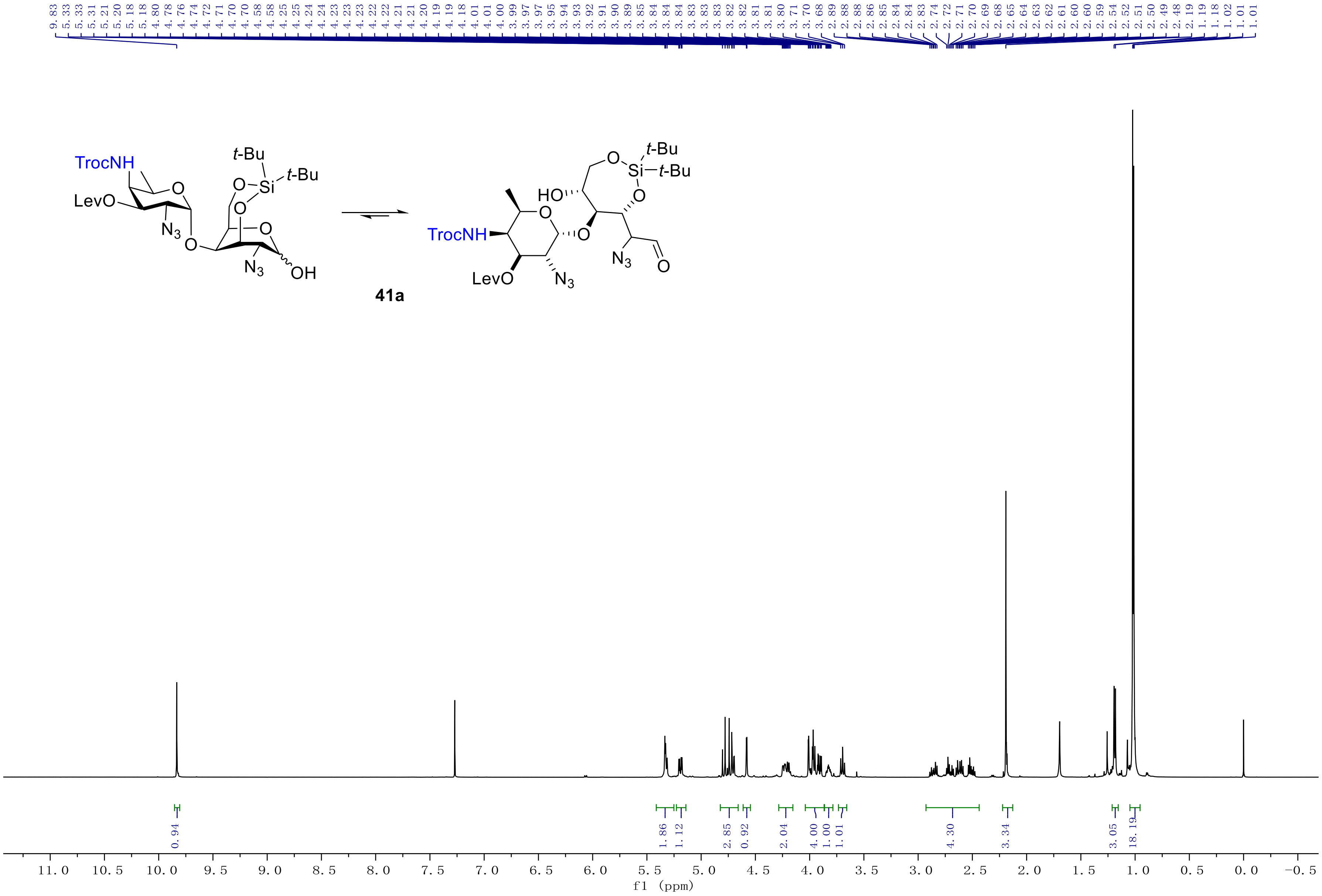

zhen2108biosyn.42.fid — wz784 — bbo-c13-APT CDCl3 /opt/topspin2.1 nmrafd 2

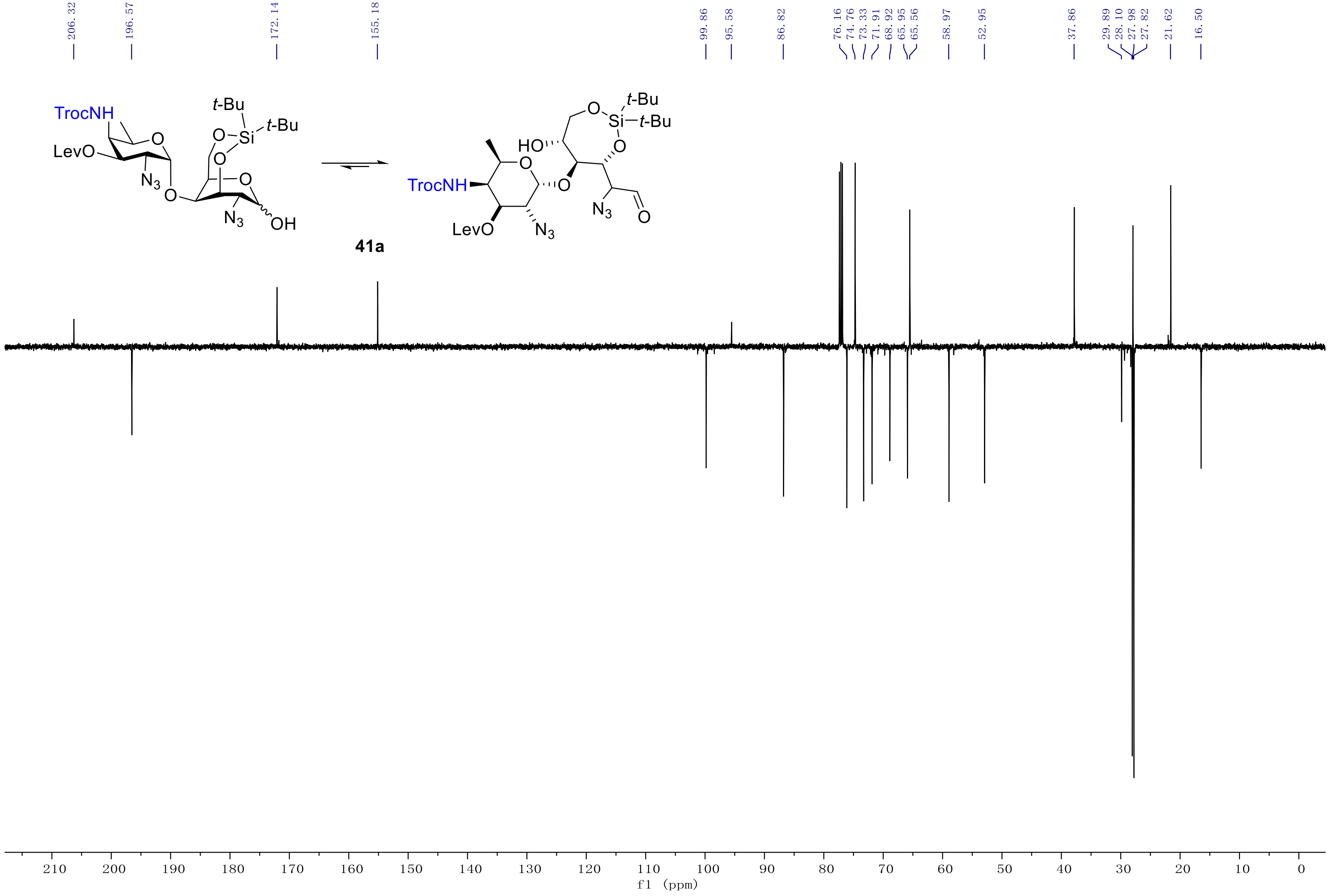

zhen2108biosyn.40.ser — wz784 — bbo-h1-cosy CDCl3 /opt/topspin2.1 nmrafd 2

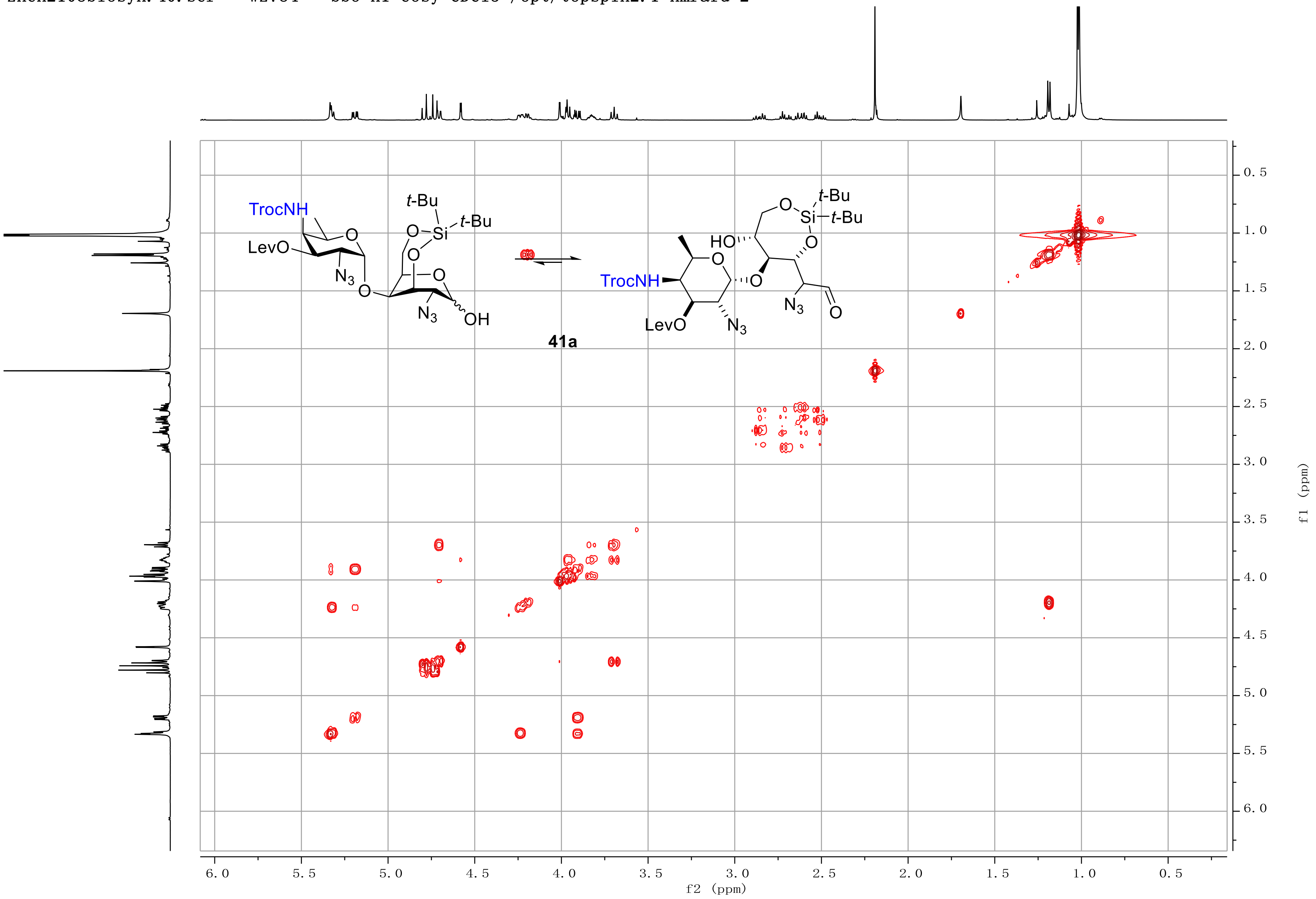

zhen2108biosyn.41.ser — wz784 — bbo-c13-HSQC CDCl3 /opt/topspin2.1 nmrafd 2

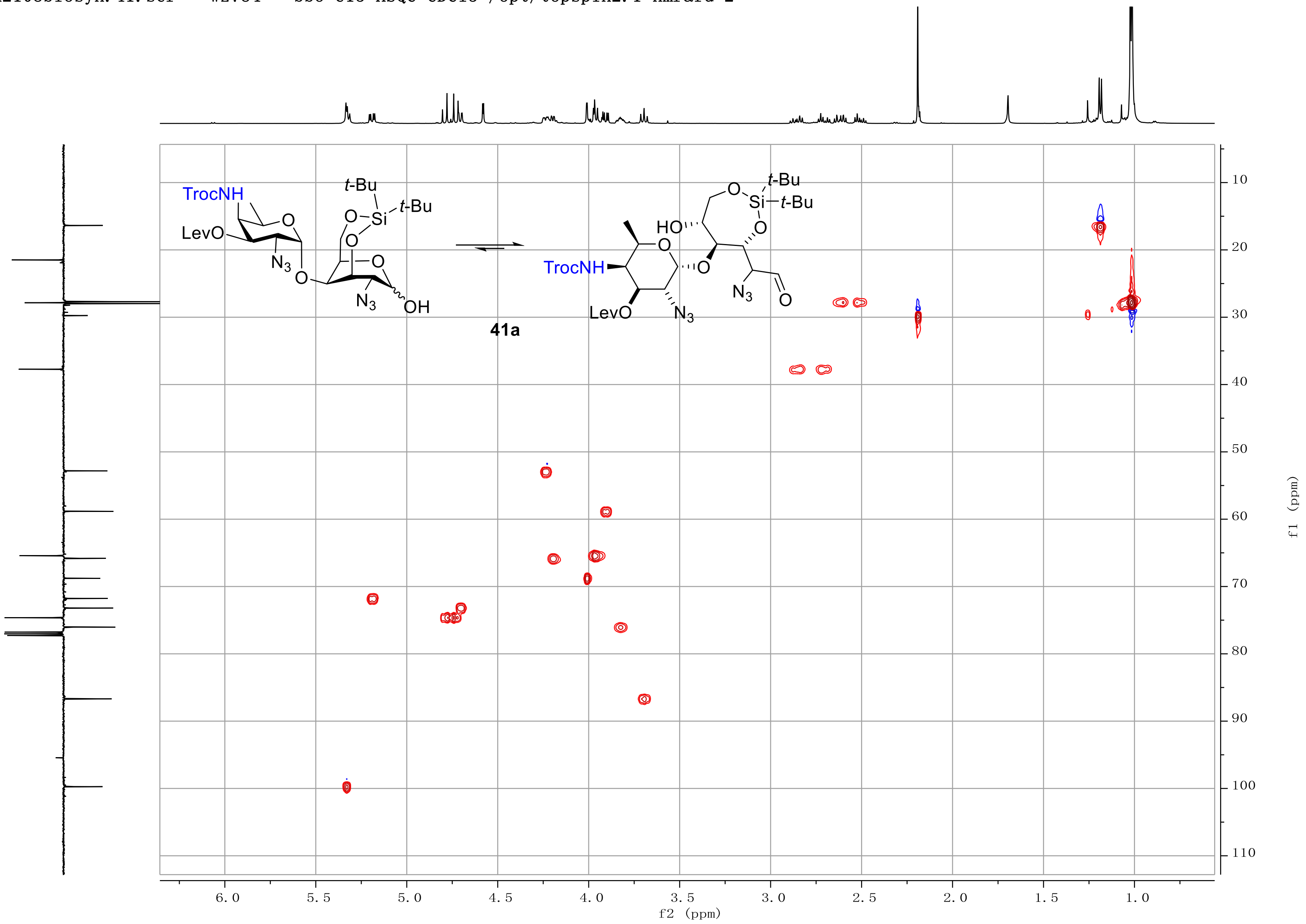

zhen2108biosyn.43.ser — wz784 — bbo-c13-HMBC CDCl3 /opt/topspin2.1 nmrafd 2

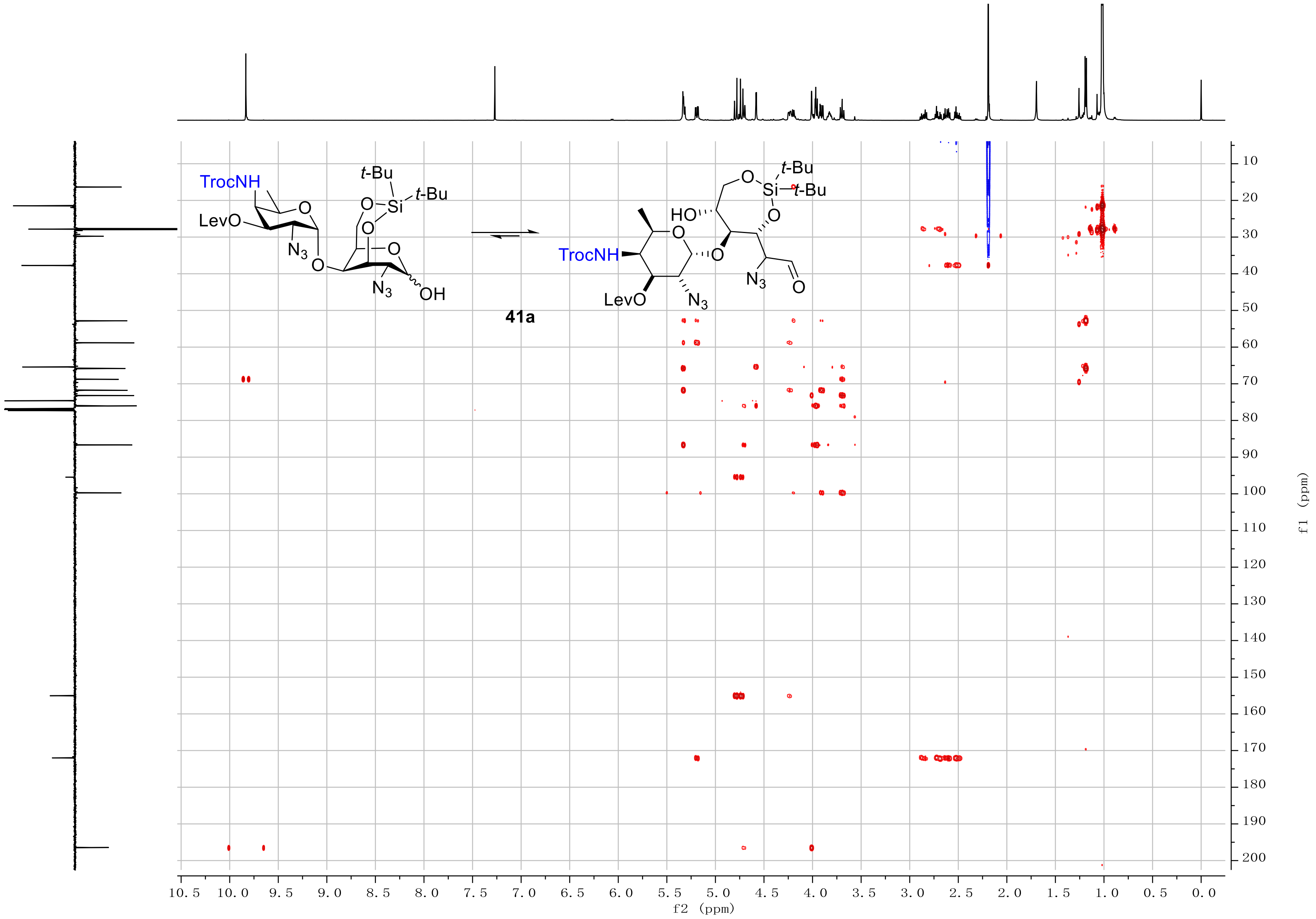

zhen2108biosyn.45.fid — wz785 — bbo-h1 Acetone /opt/topspin2.1 nmrafd 6

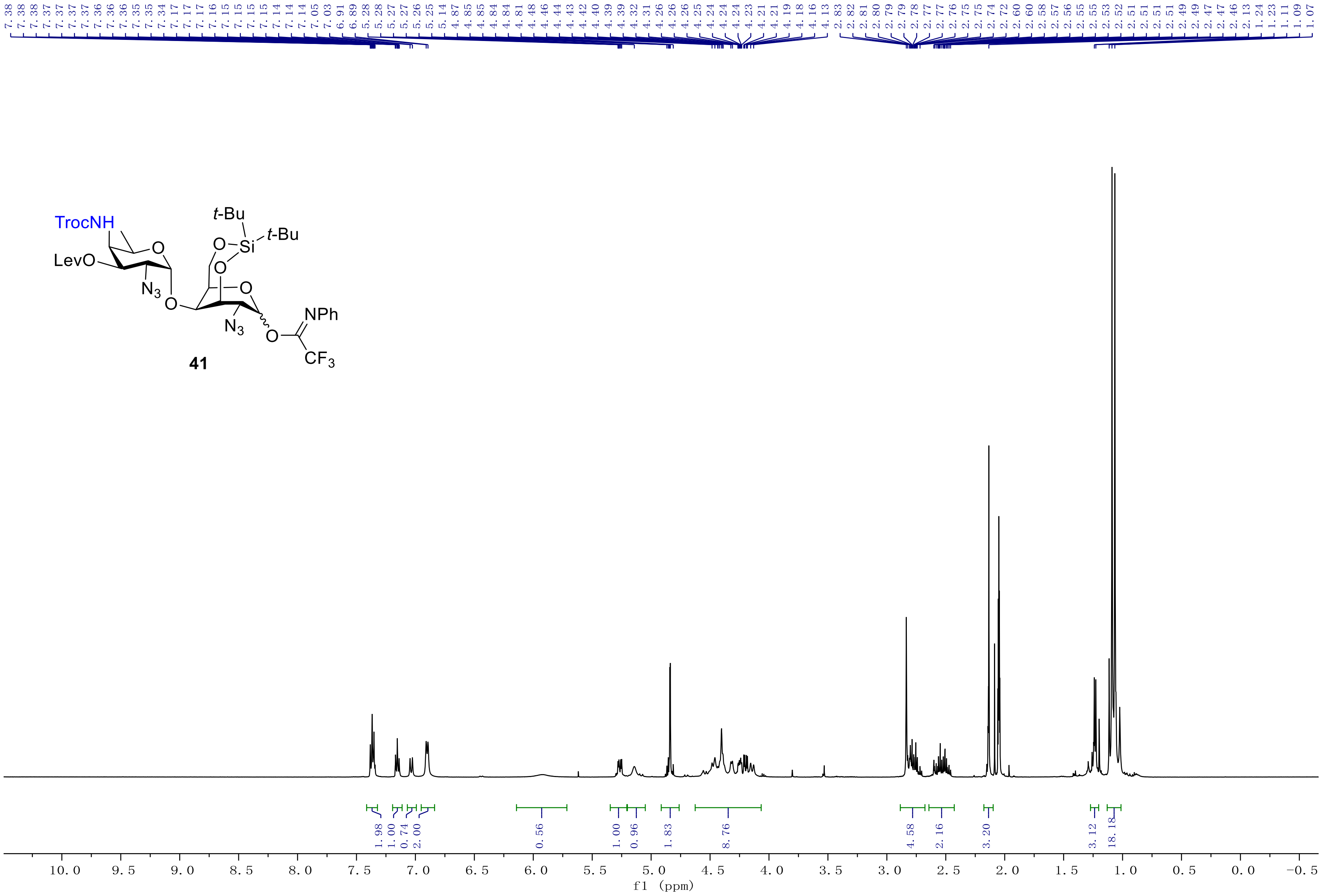

zhen2108biosyn.48.fid — wz785 — bbo-c13-APT Acetone /opt/topspin2.1 nmrafd 6

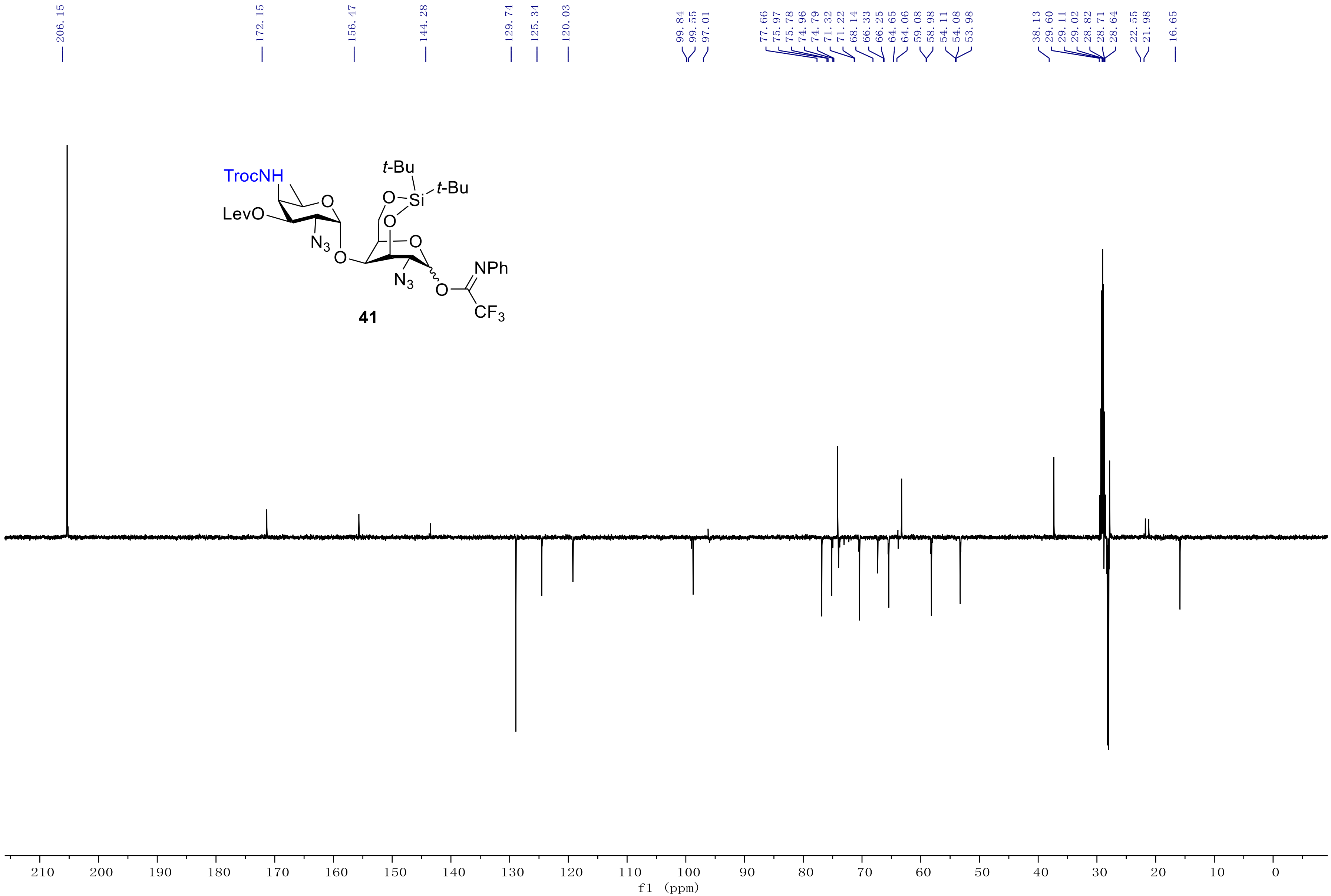

zhen2108biosyn.46.ser — wz785 — bbo-h1-cosy Acetone /opt/topspin2.1 nmrafd 6

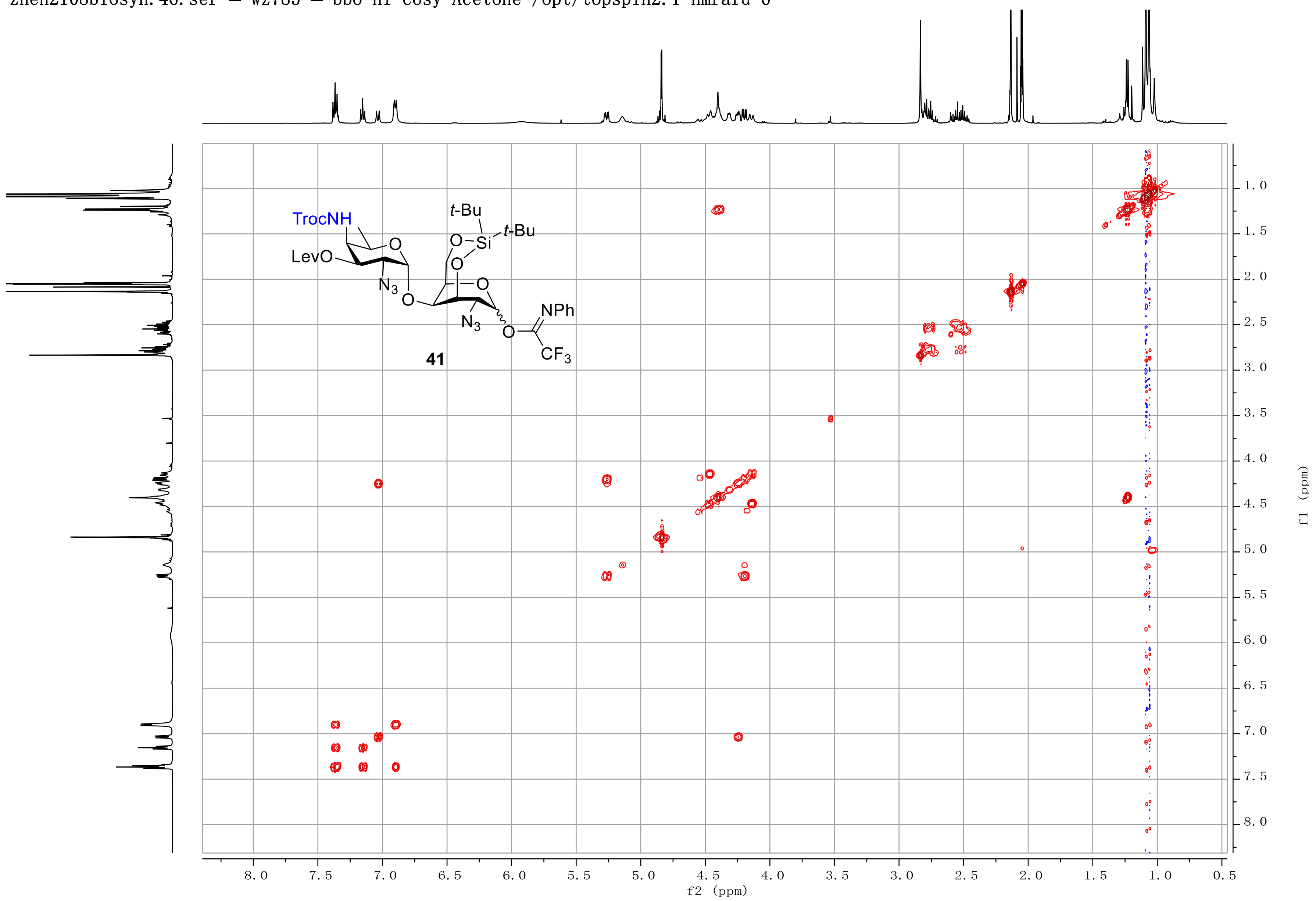

zhen2108biosyn.47.ser — wz785 — bbo-c13-HSQC Acetone /opt/topspin2.1 nmrafd 6

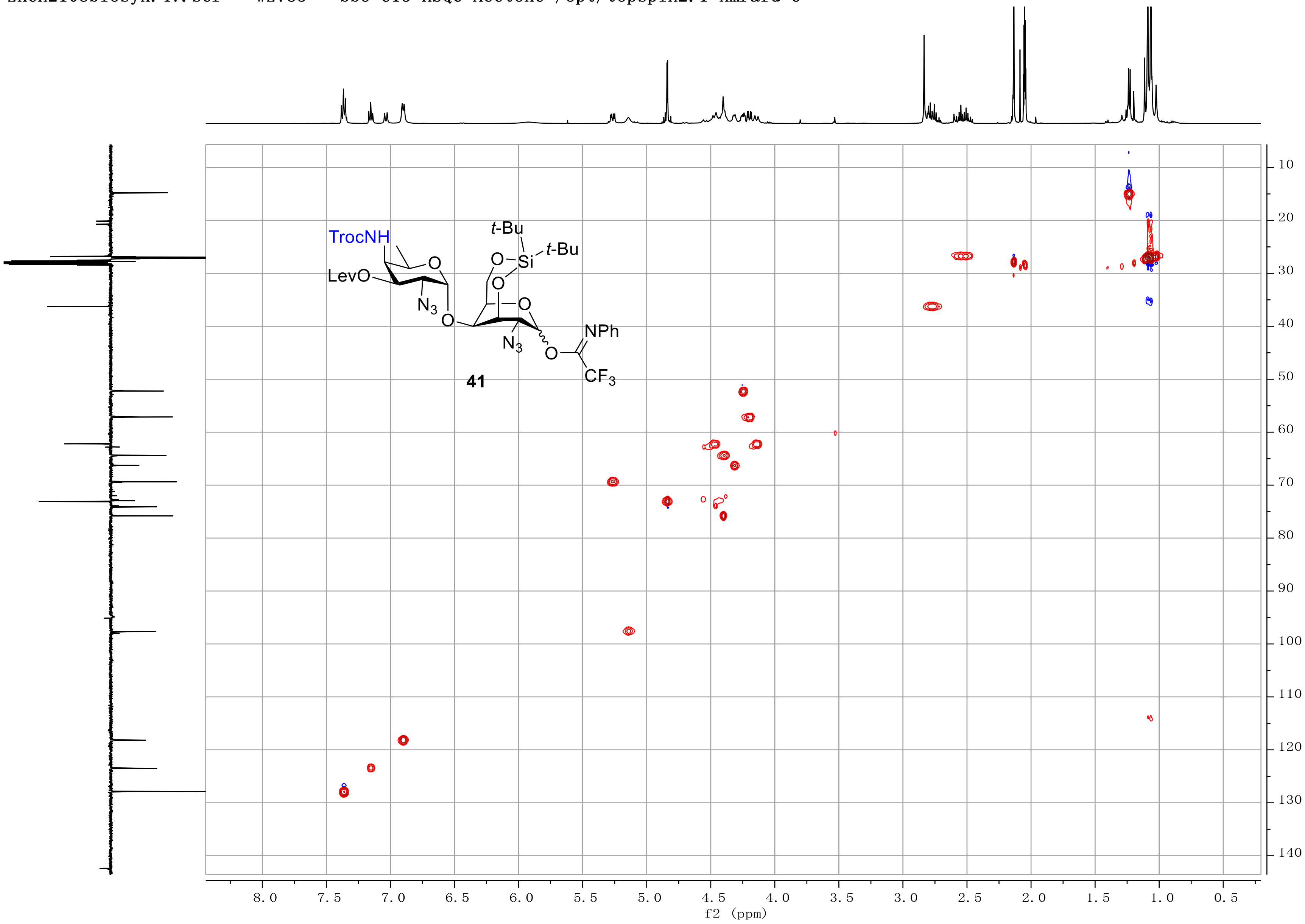

zhen2108biosyn.49.ser — wz785 — bbo-c13-HMBC Acetone /opt/topspin2.1 nmrafd 6

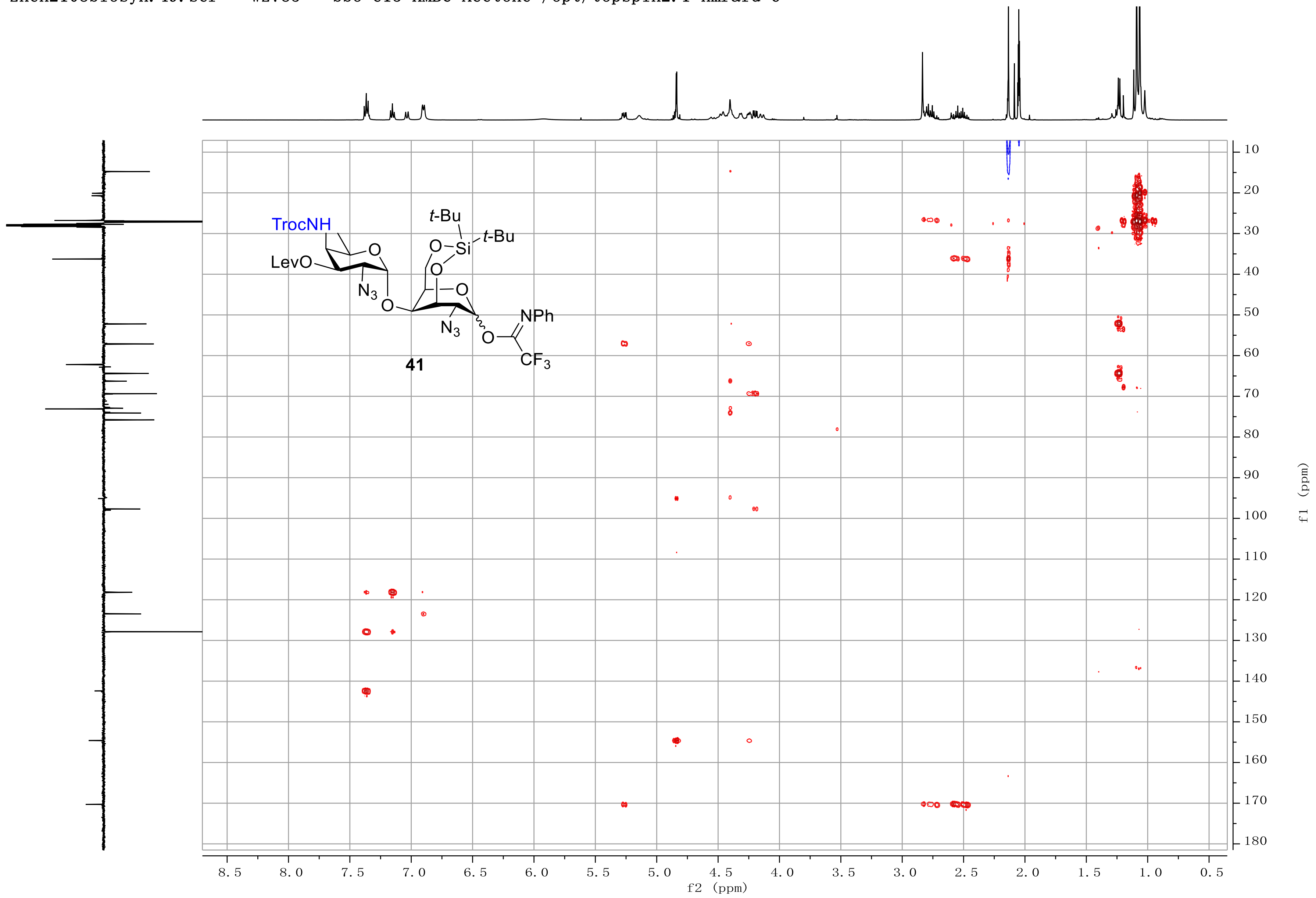

zhen2108biosyn.51.fid — wz786 — bbo-h1 CDCl3 /opt/topspin2.1 nmrafd 4

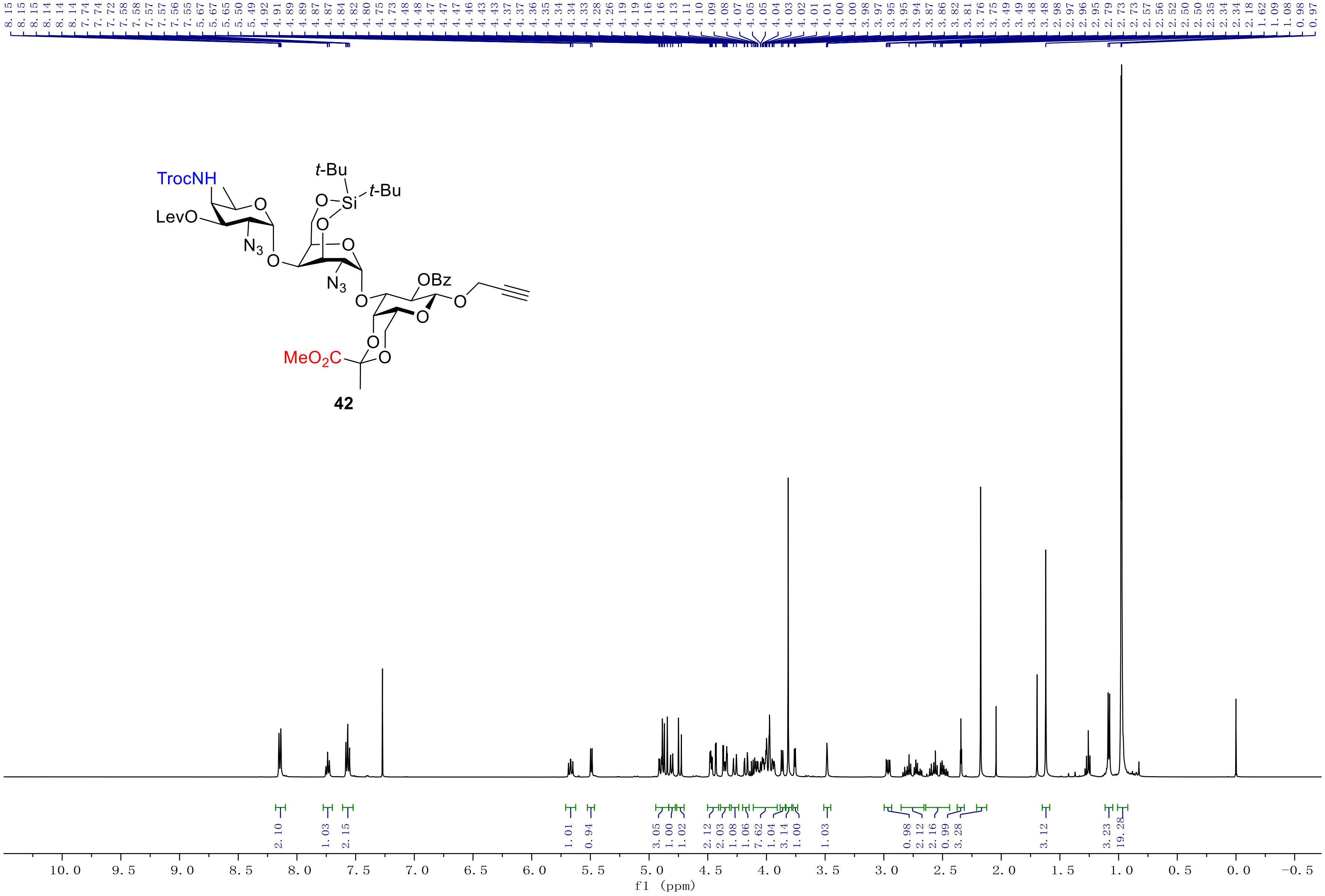

zhen2108biosyn.54.fid — wz786 — bbo-c13-APT CDCl3 /opt/topspin2.1 nmrafd 4

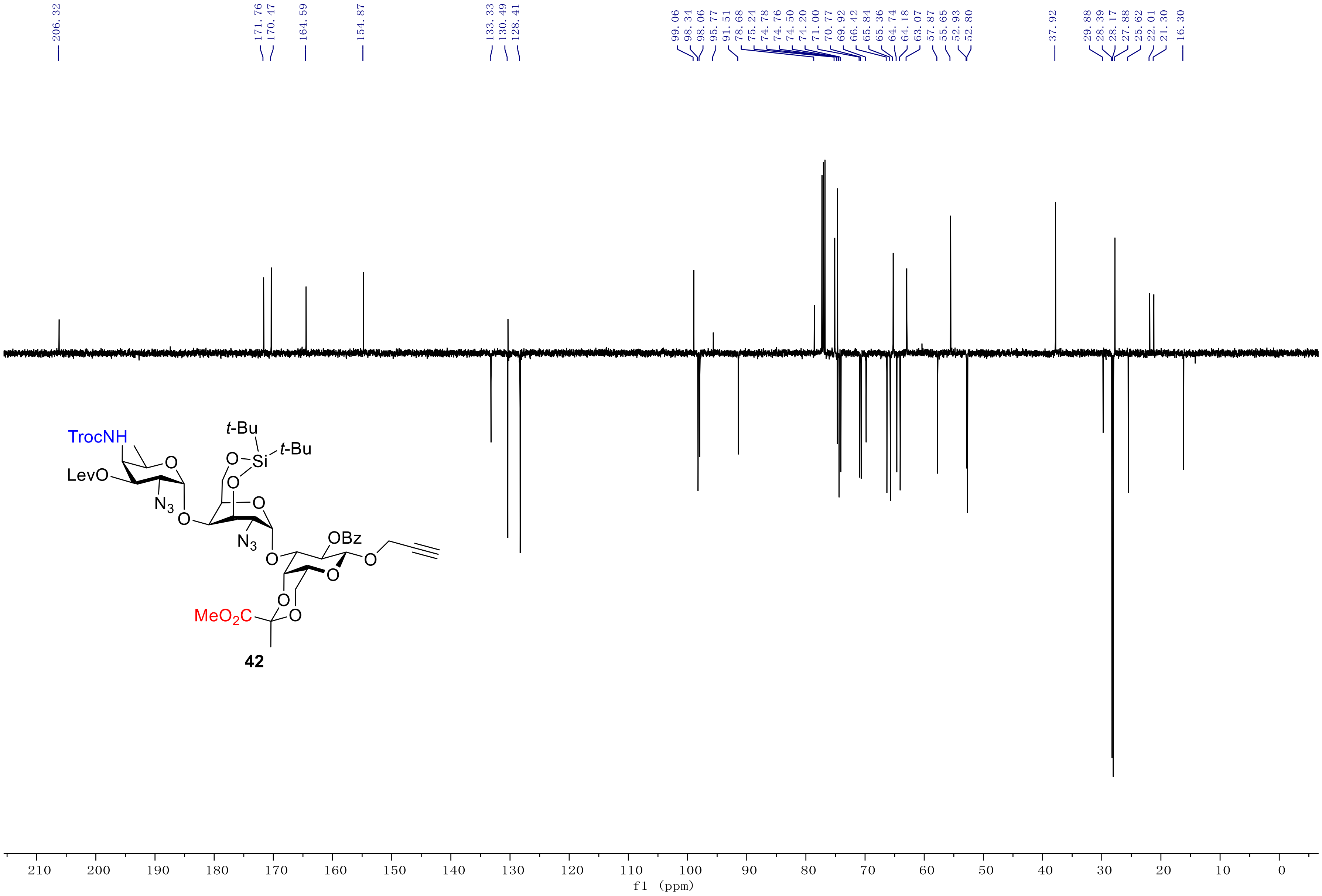

42

zhen2108biosyn.52.ser — wz786 — bbo-h1-cosy CDCl3 /opt/topspin2.1 nmrafd 4

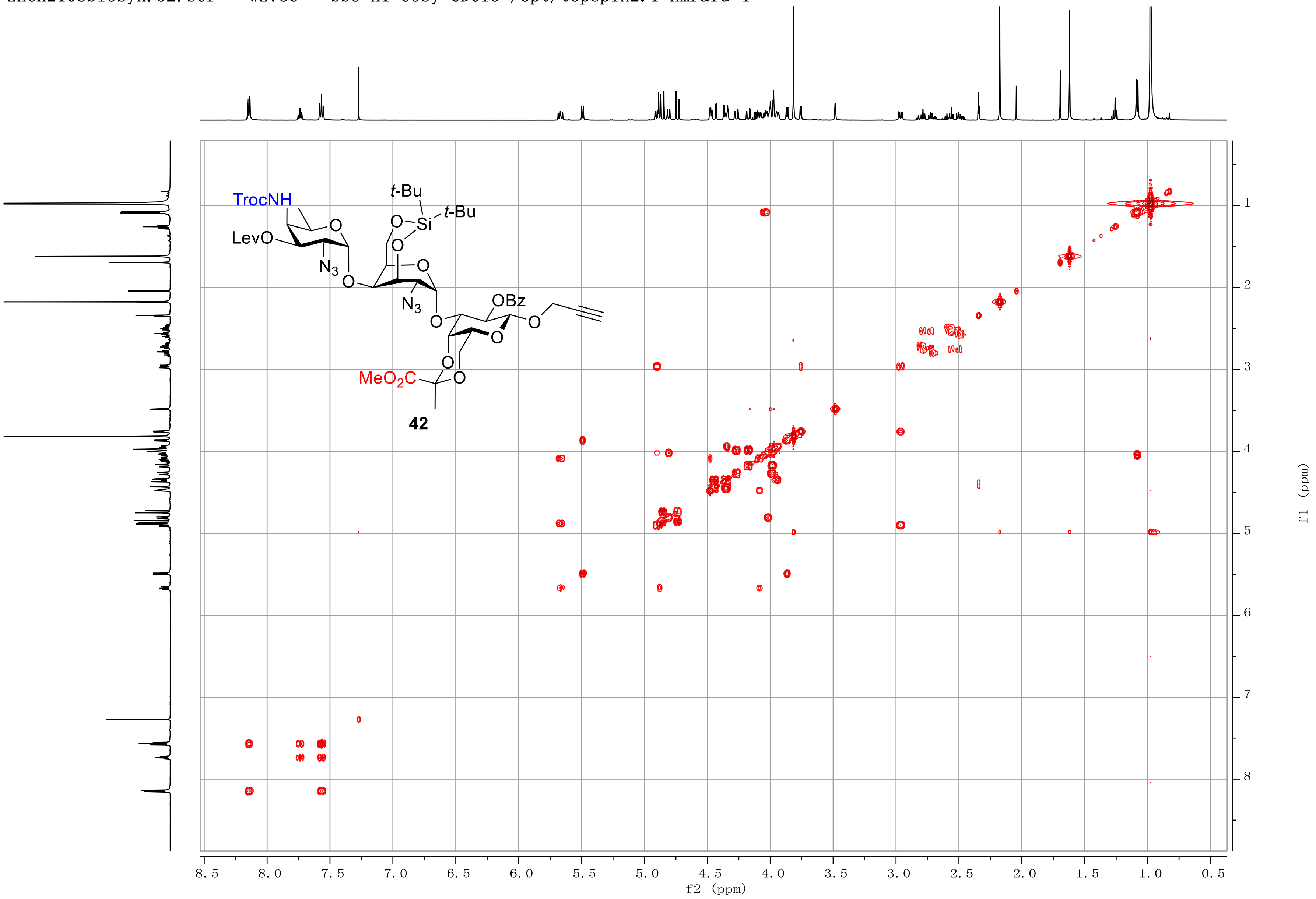

zhen2108biosyn.53.ser — wz786 — bbo-c13-HSQC CDCl3 /opt/topspin2.1 nmrafd 4

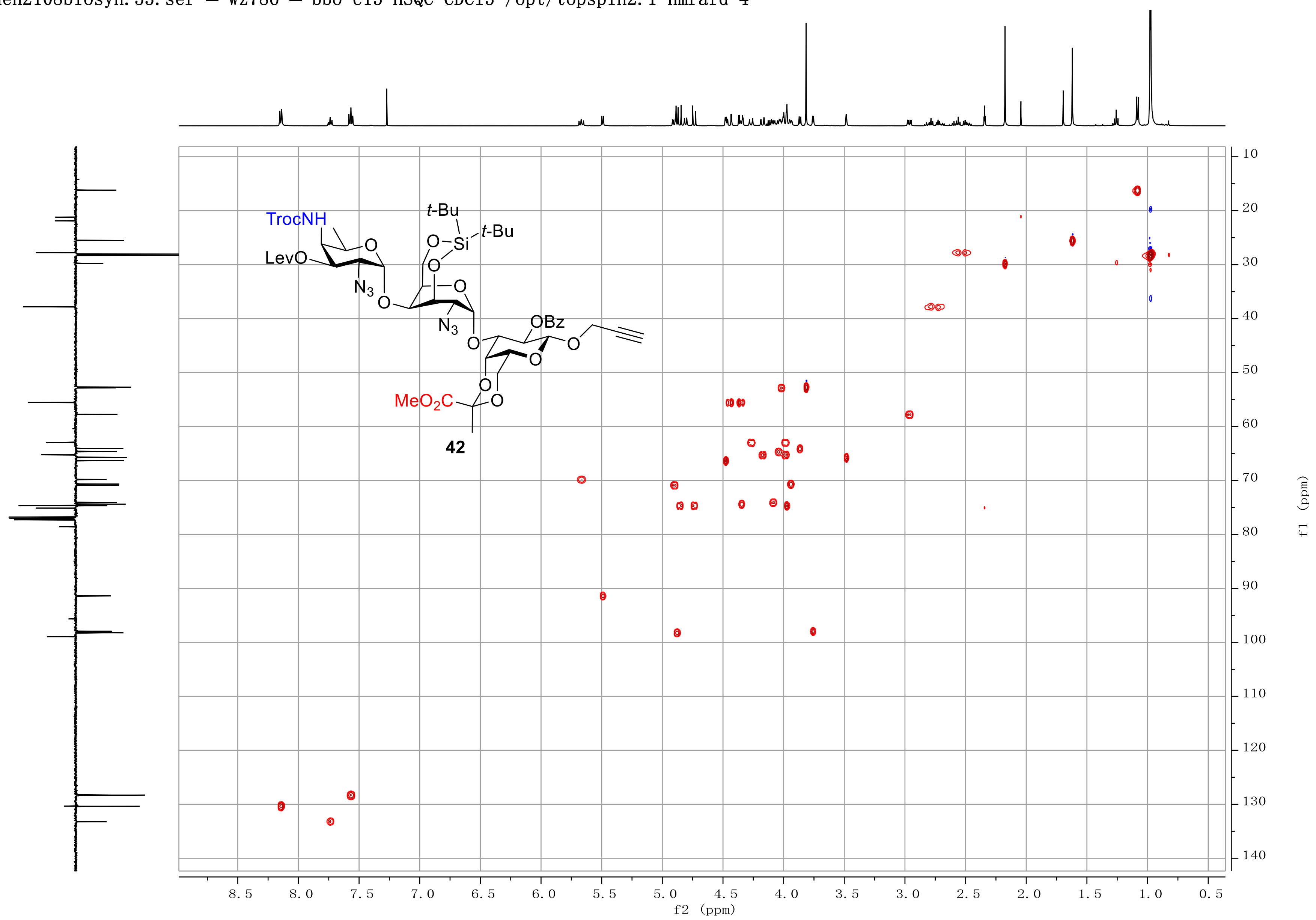

zhen2108biosyn.55.ser — wz786 — bbo-c13-HMBC CDCl3 /opt/topspin2.1 nmrafd 4

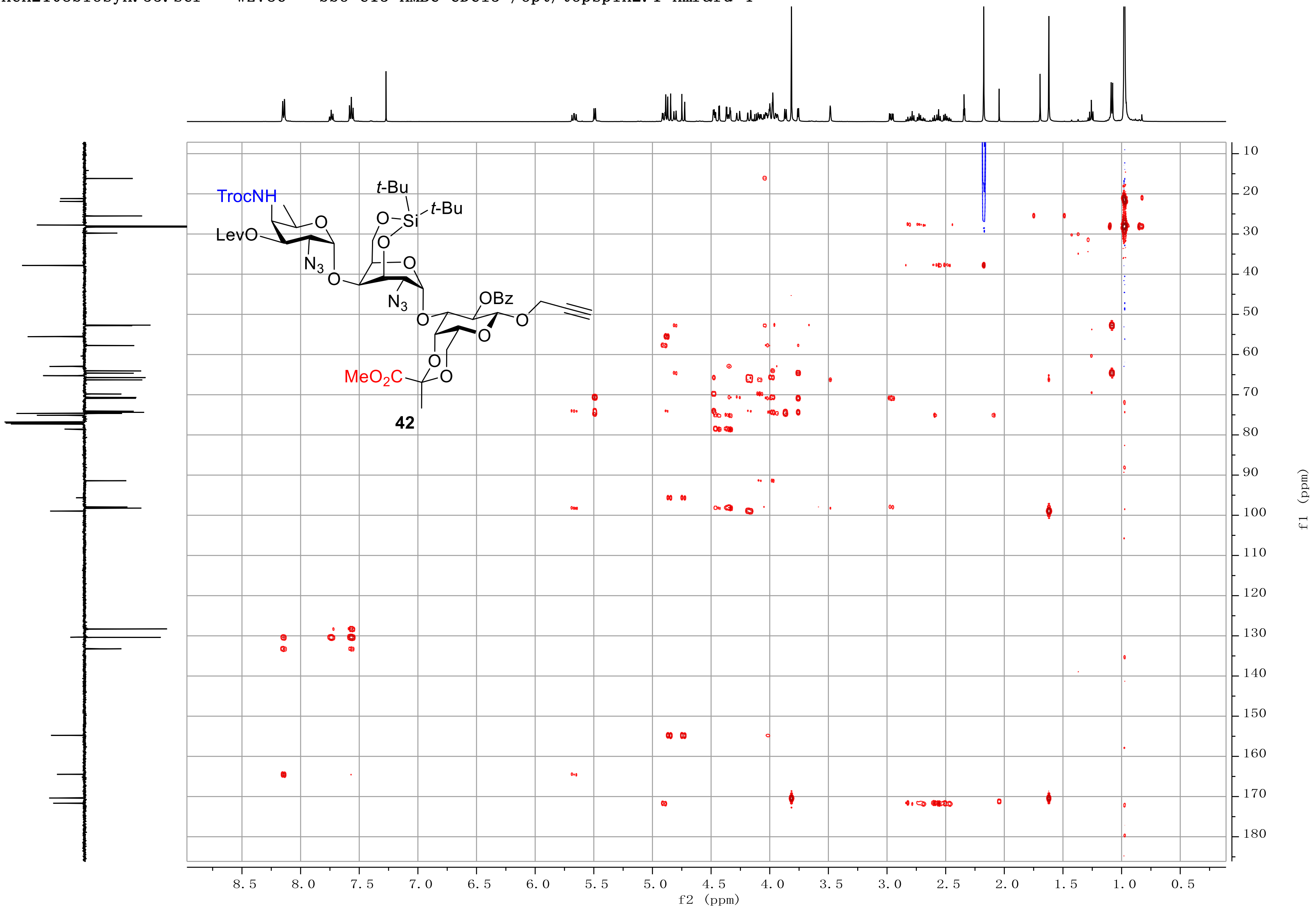

zhen2108biosyn.56.ser — wz786 — bbo-c13-hmbc-ipv-gated CDCl3 /opt/topspin2.1 nmrafd 4

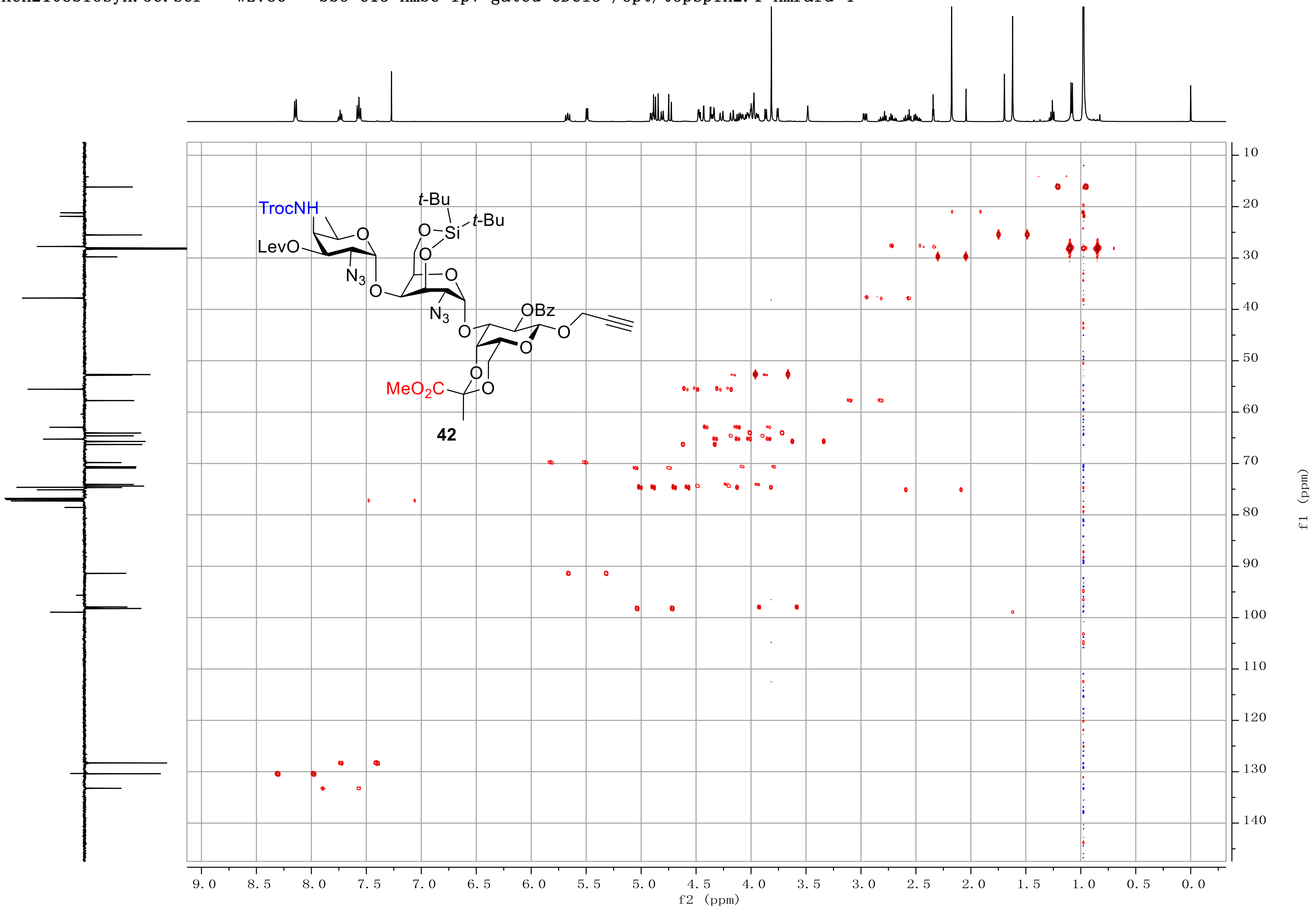

2108zhen.34.fid — wz788-A-s — h1 CDCl3 /opt/DATA nmrafd 9

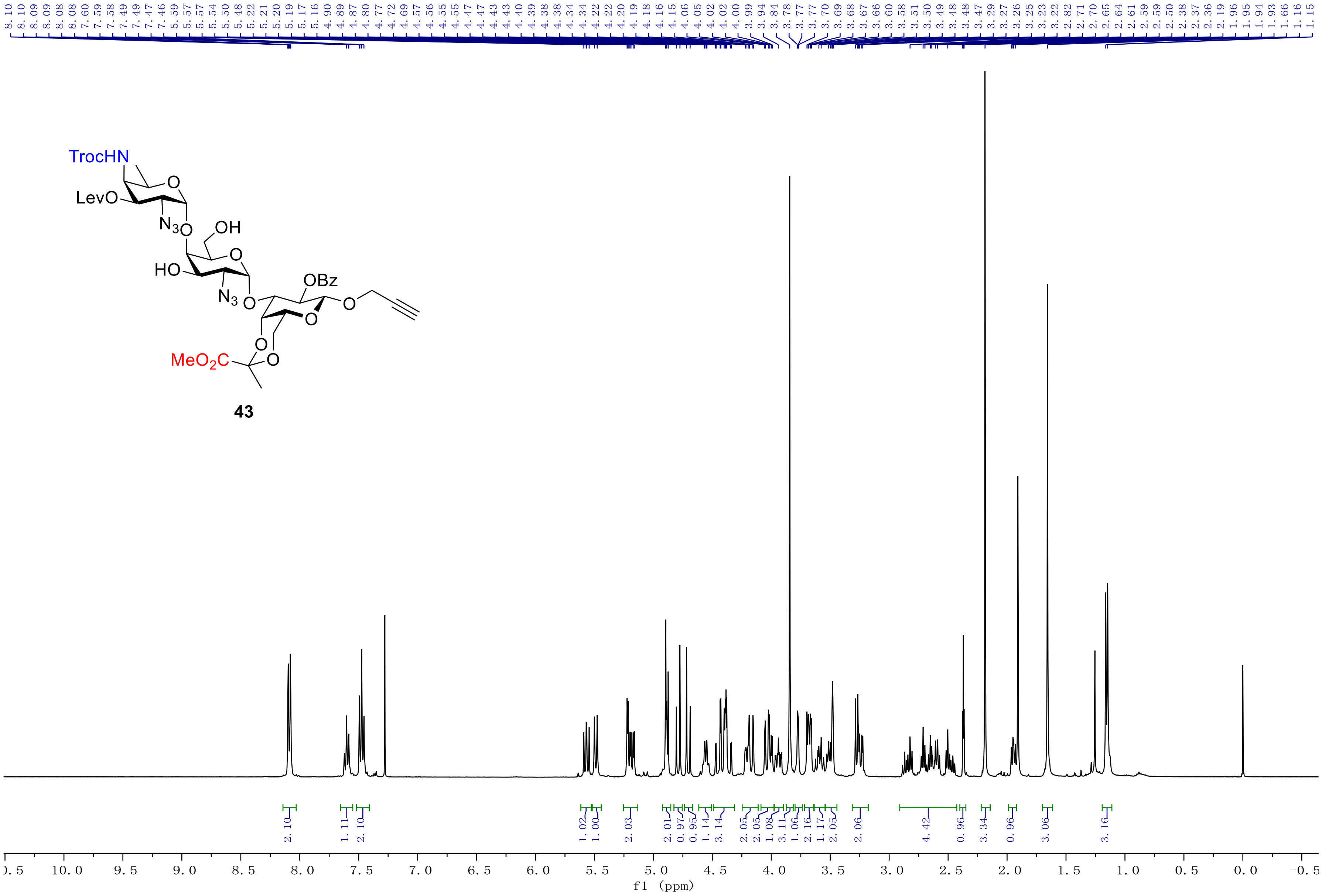

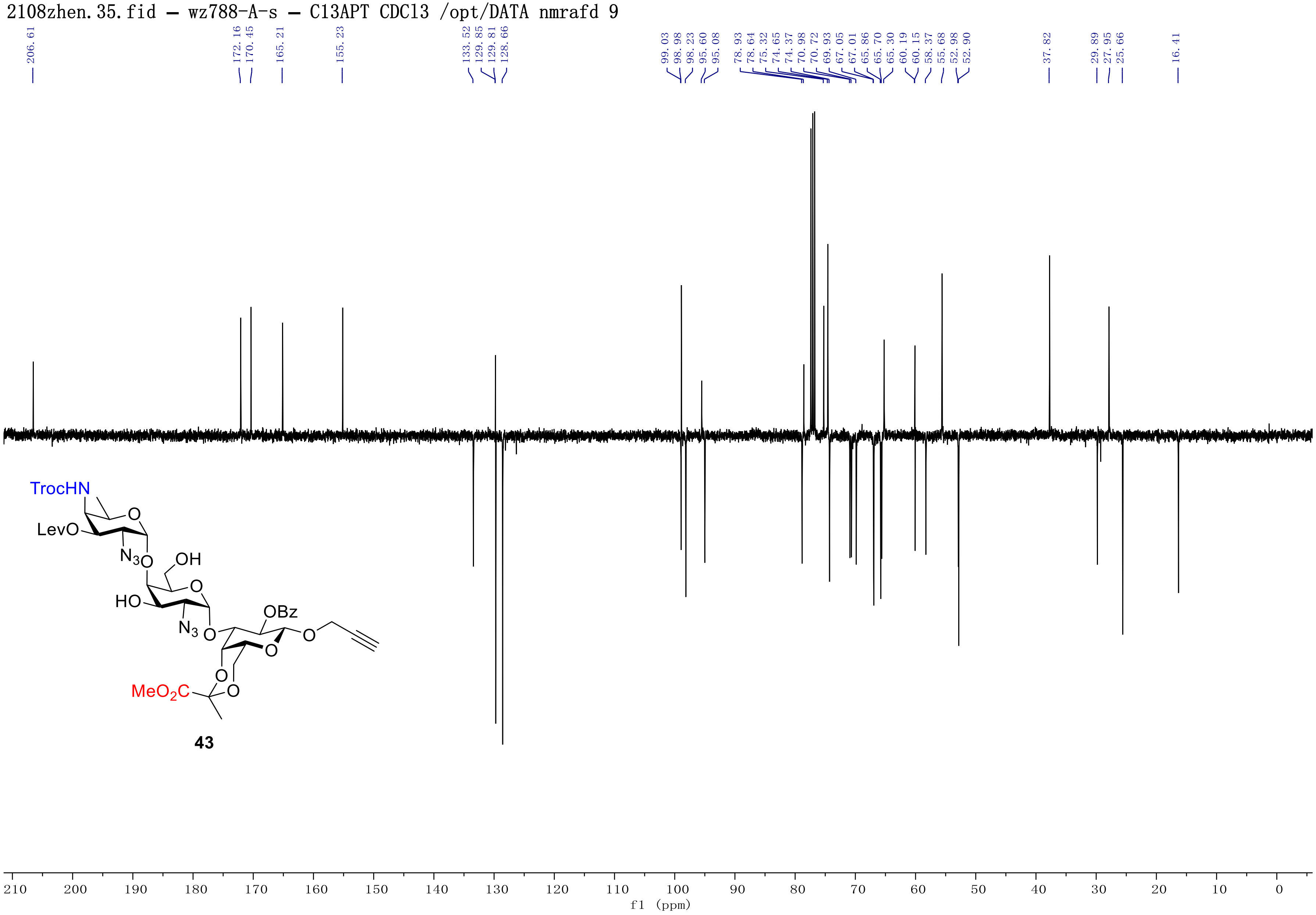
2108zhen.35.fid — wz788-A-s — C13APT CDCl3 /opt/DATA nmrafd 9
206.61
172.16
170.45
165.21
155.23
133.52
129.85
129.81
128.66
99.03
98.98
98.23
95.60
95.08
78.93
78.64
75.32
74.65
74.37
70.98
70.72
69.93
67.05
67.01
65.86
65.70
65.30
60.19
60.15
58.37
55.68
52.98
52.90
37.82
29.89
27.95
25.66
16.41
TrocHN
LevO
N3
O
OH
HO
OBz
MeO2C
43
f1 (ppm)

2108zhen.36.ser — wz788-A-s — h1COSY CDCl3 /opt/DATA nmrafd 9

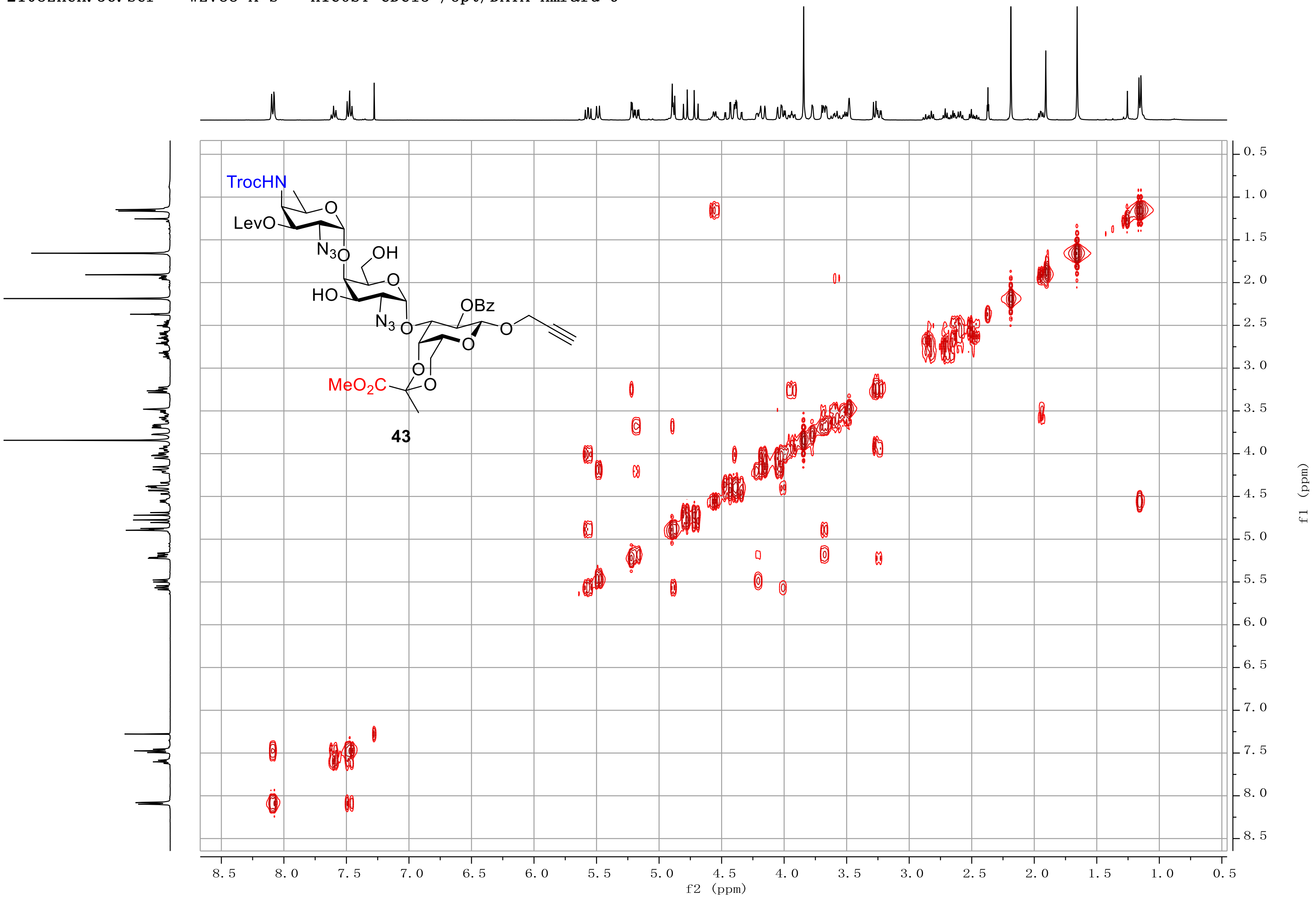

2108zhen.37.ser — wz788-A-s — c13HSQC CDCl3 /opt/DATA nmrafd 9

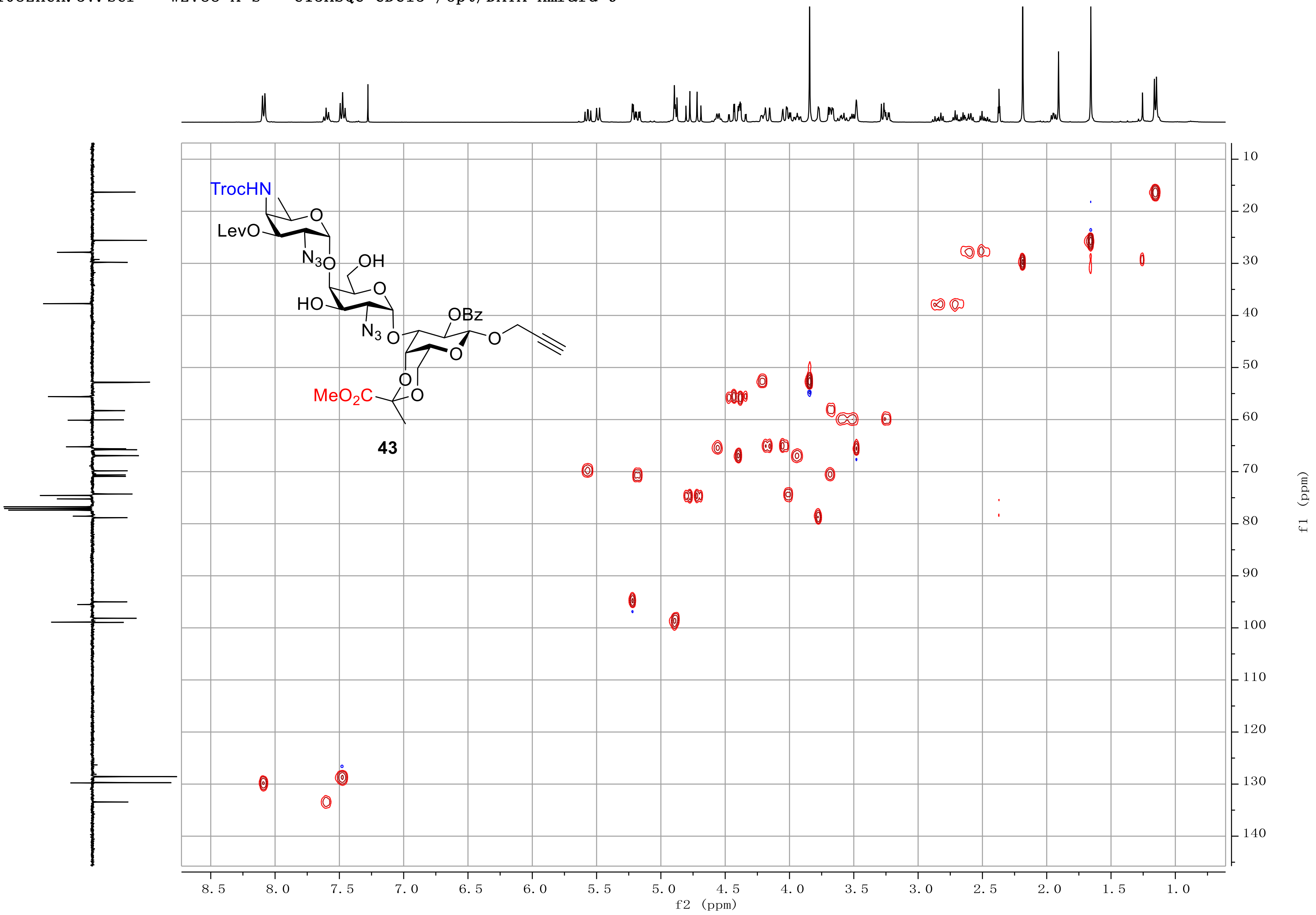

2108zhen.38.ser — wz788-A-s — c13HMBC CDCl3 /opt/DATA nmrafd 9

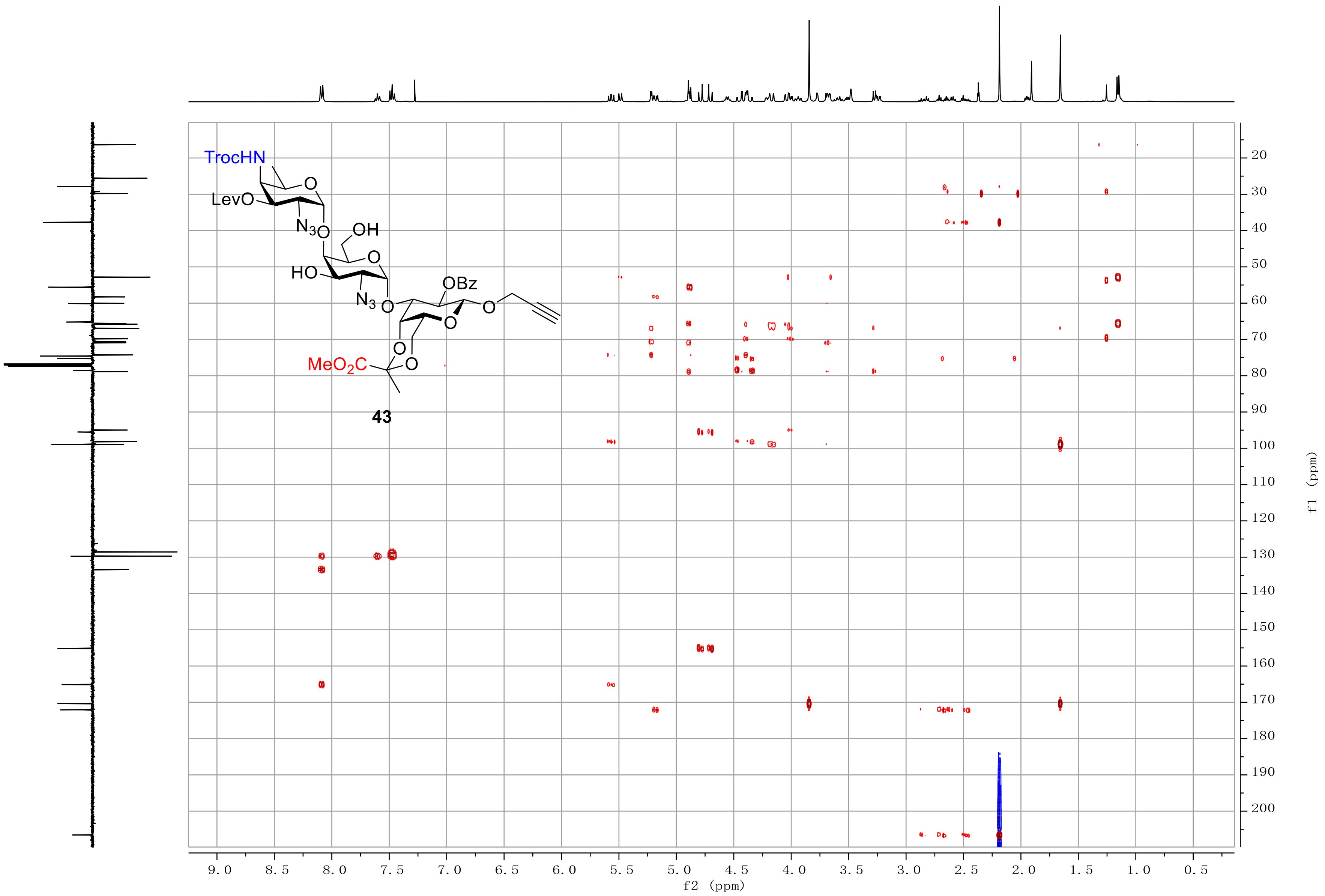

2108zhen.39.ser — wz788-A-s — c13HMBCipvGATED CDCl3 /opt/DATA nmrafd 9

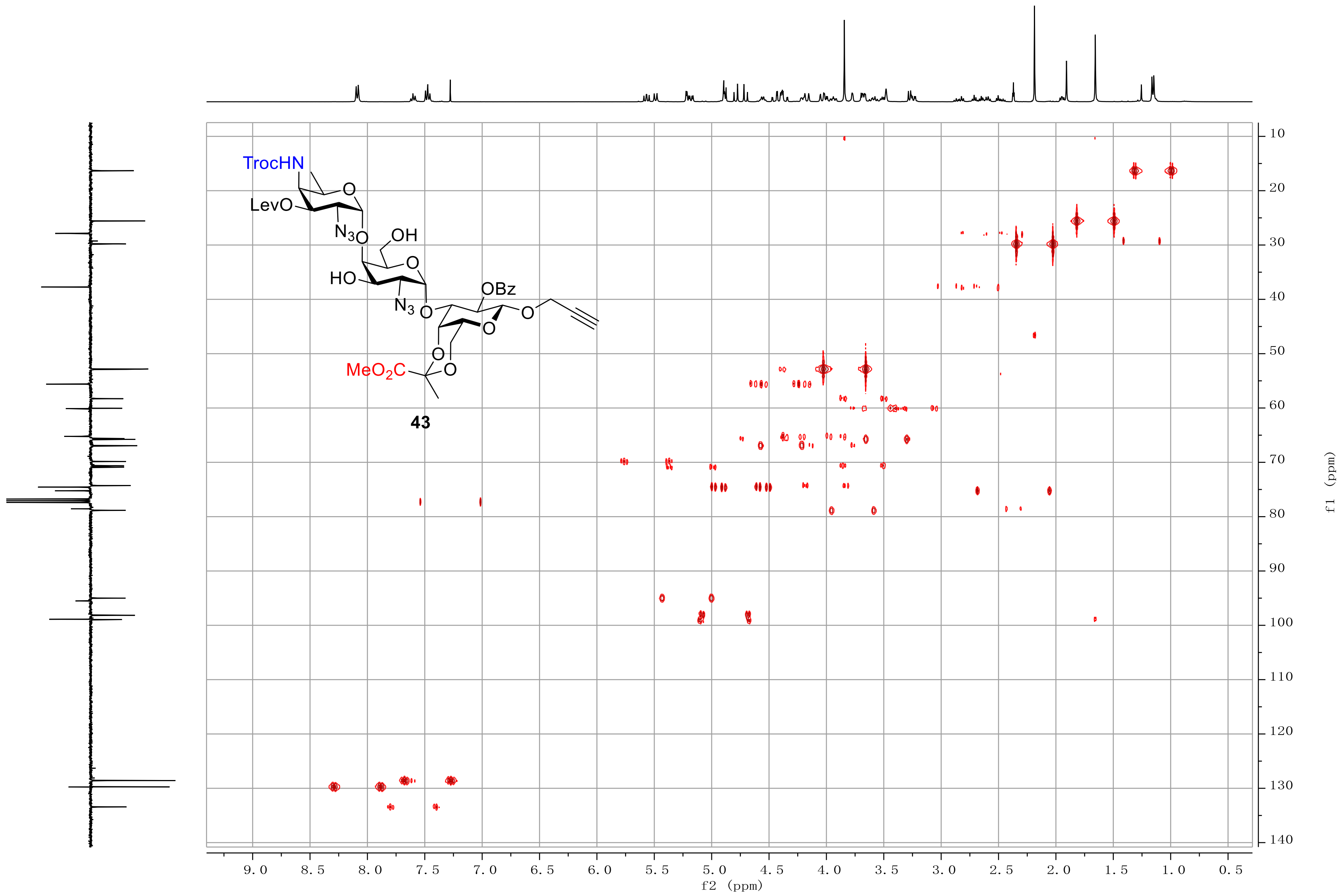

2109zhen.13.fid — wz789-B — h1 CDCl3 /opt/DATA nmrafd 13

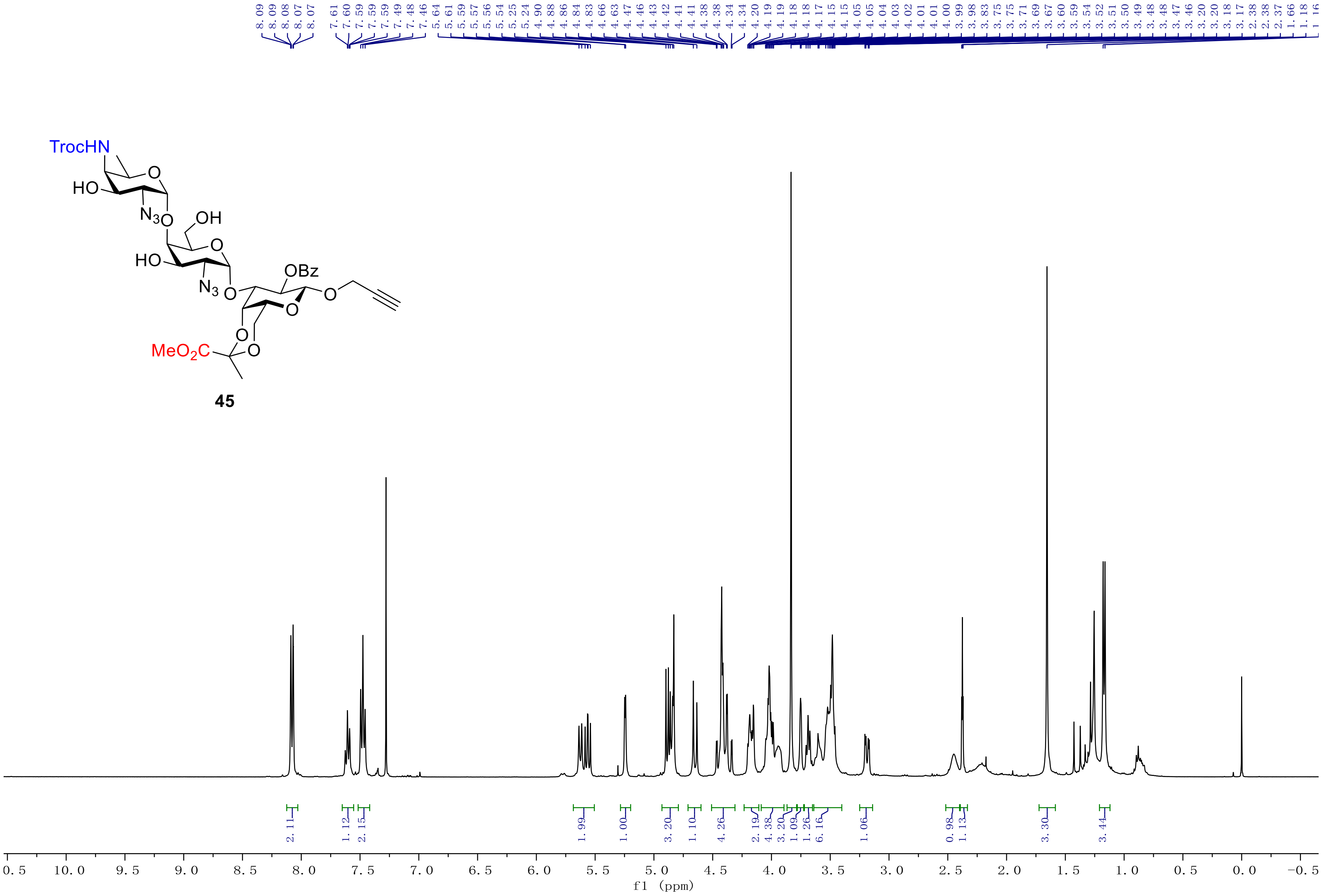

45

2109zhen.14.fid — wz789-B — C13APT CDCl3 /opt/DATA nmrafd 13

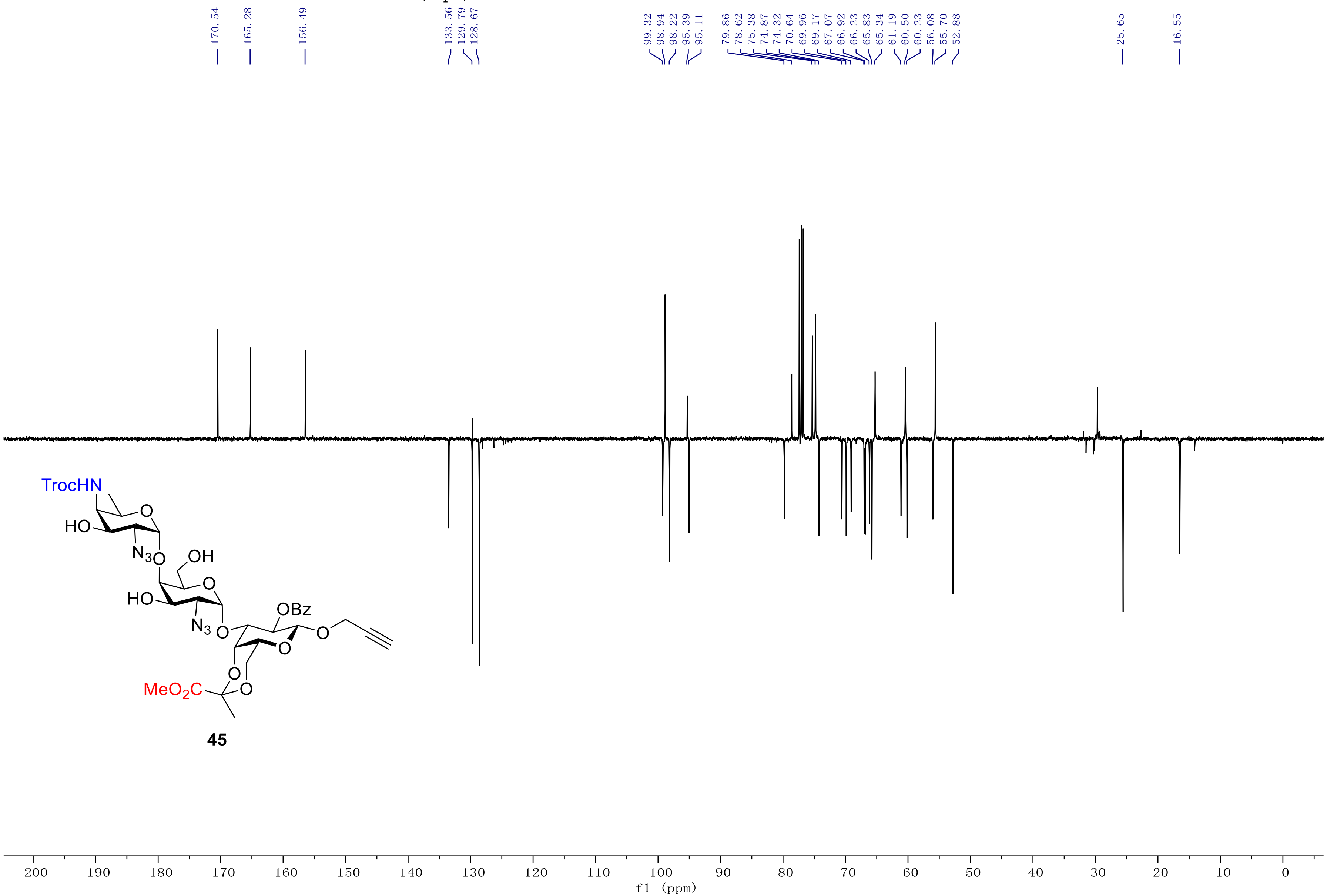

2109zhen.15.ser — wz789-B — h1COSY CDCl3 /opt/DATA nmrafd 13

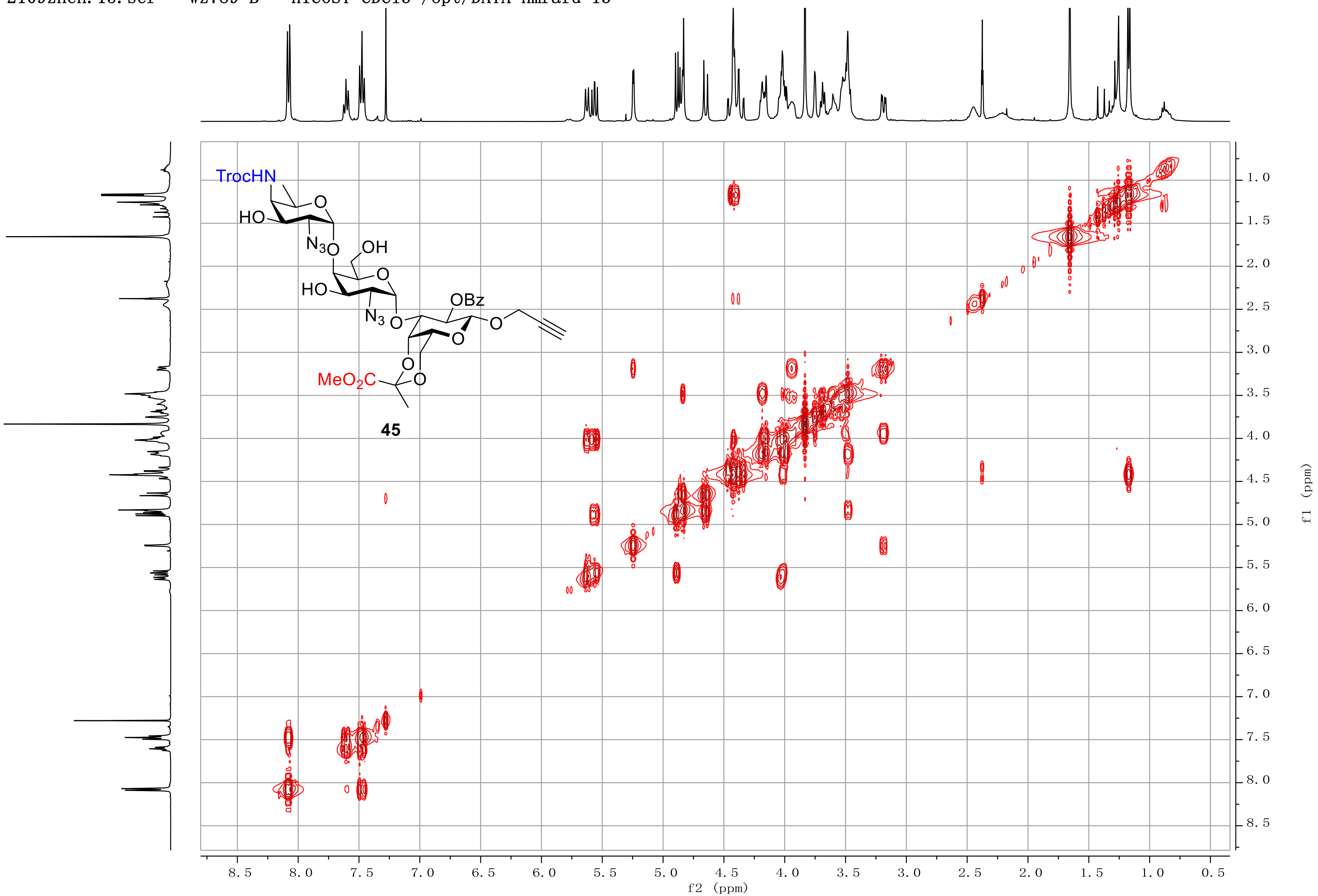

2109zhen.16.ser — wz789-B — c13HSQC CDCl3 /opt/DATA nmrafd 13

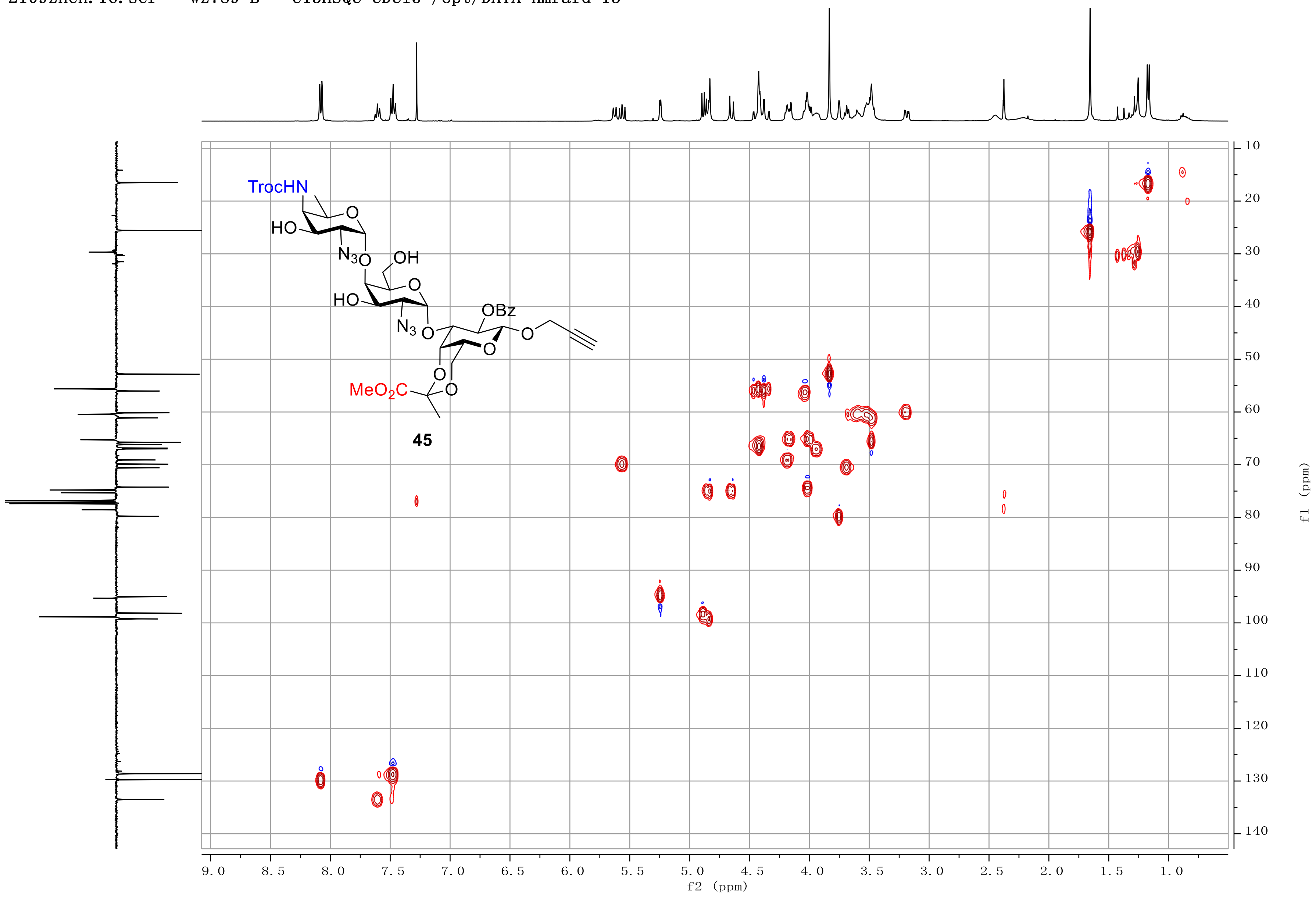

2109zhen.17.ser — wz789-B — c13HMBC CDCl3 /opt/DATA nmrafd 13

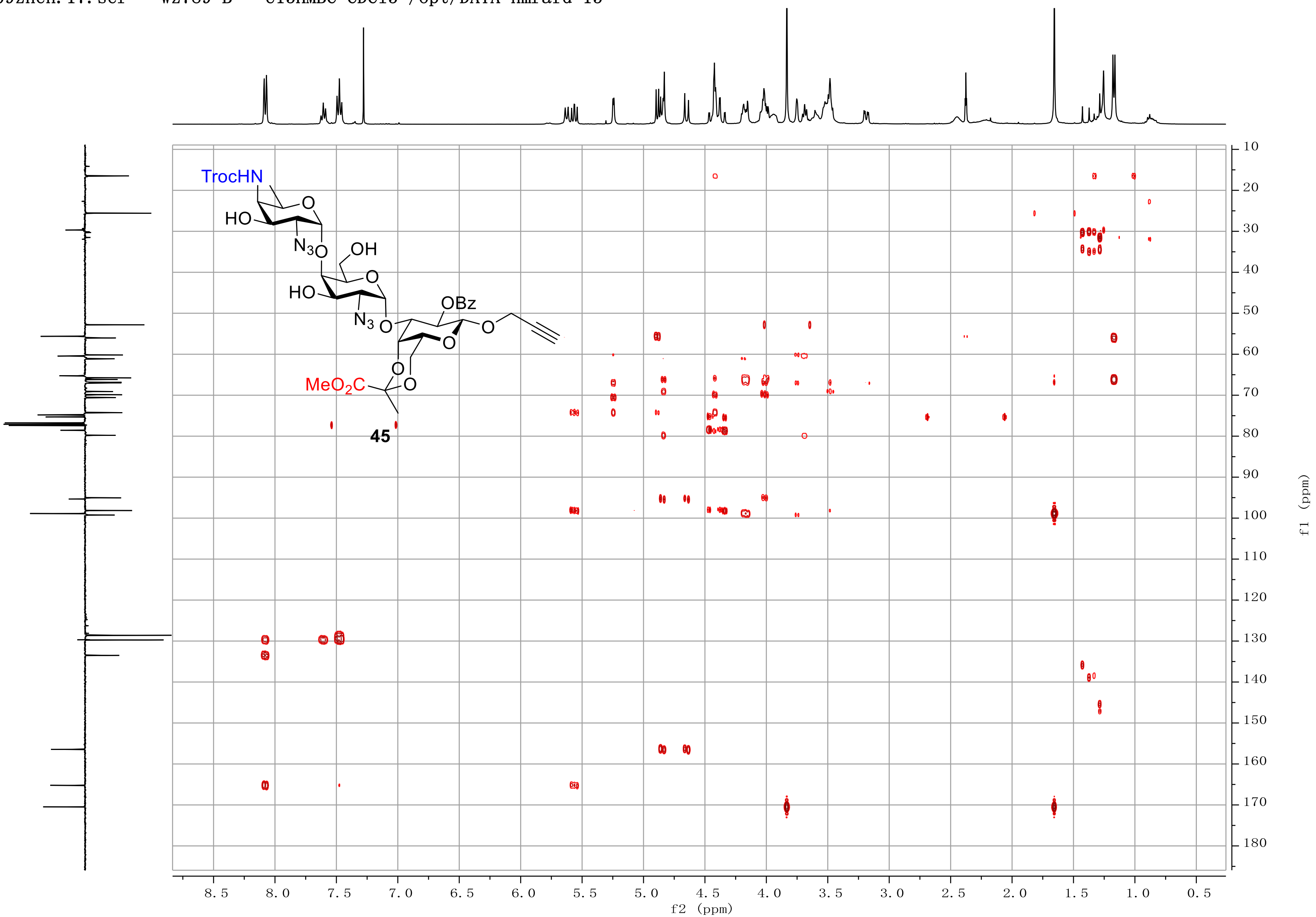

zhen2109biosyn.11.fid — wz790-B — bbo-h1 CDCl3 /opt/topspin2.1 nmrafd 7

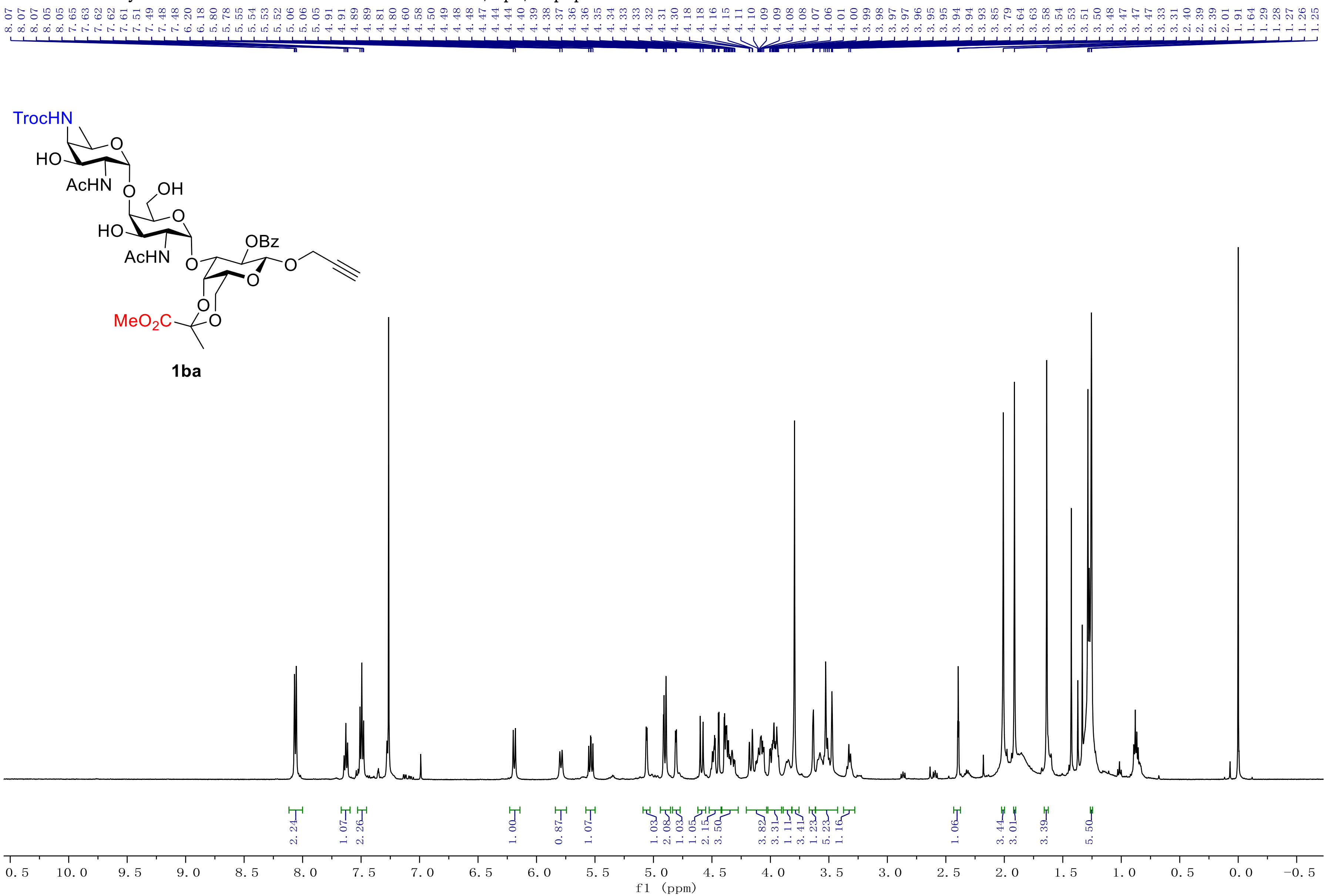

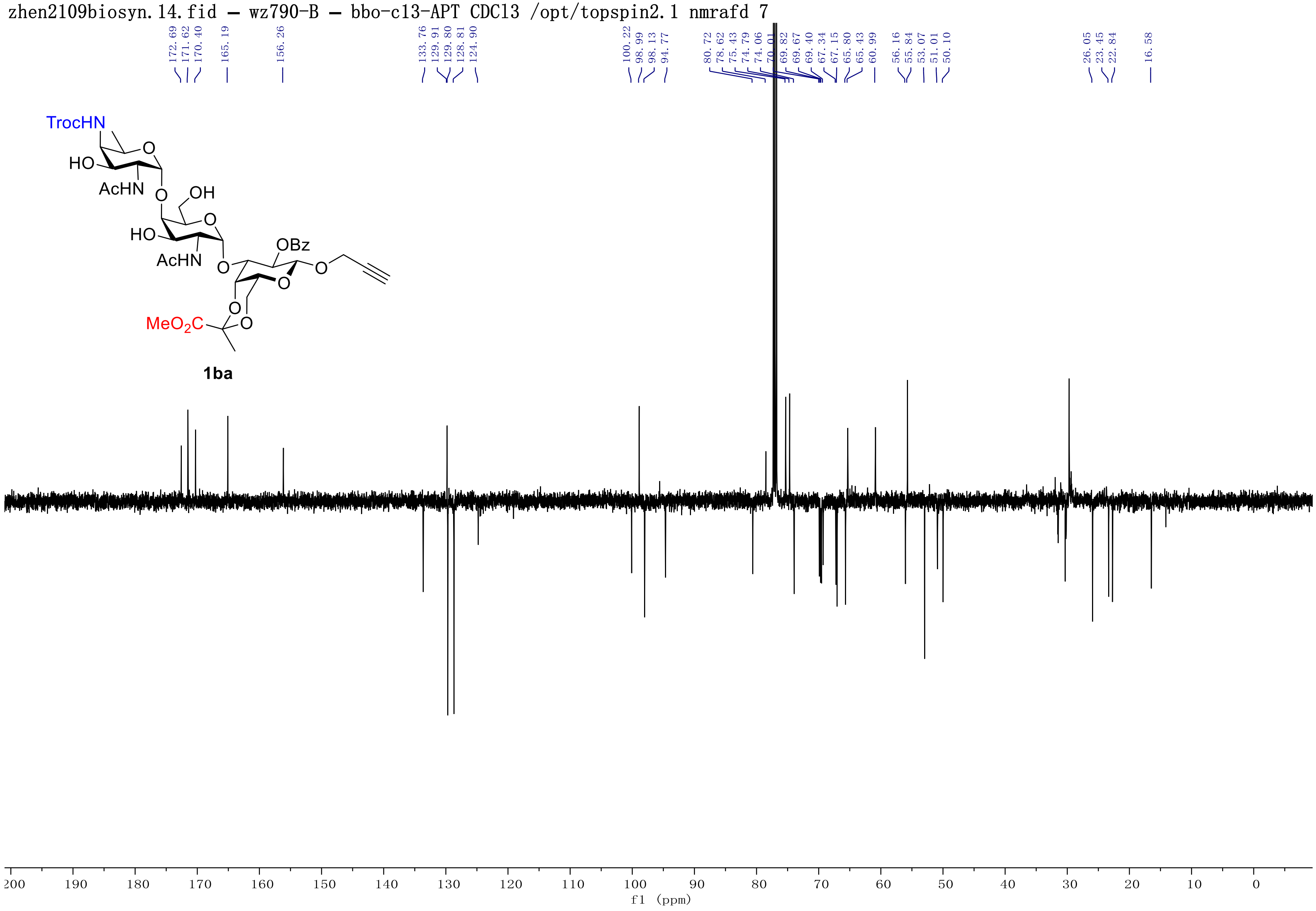
zhen2109biosyn.14.fid — wz790-B — bbo-c13-APT CDCl3 /opt/topspin2.1 nmrafd 7
172.69
171.62
170.40
165.19
156.26
133.76
129.91
129.80
128.81
124.90
100.22
98.99
98.13
94.77
80.72
78.62
75.43
74.79
74.06
70.01
69.82
69.67
69.40
67.34
67.15
65.80
65.43
60.99
56.16
55.84
53.07
51.01
50.10
26.05
23.45
22.84
16.58
TrocHN
HO
AcHN
OH
HO
AcHN
OBz
MeO2C
1ba
f1 (ppm)

zhen2109biosyn.2.ser — wz790-A-1 — bbo-h1-cosy CDCl3 /opt/topspin2.1 nmrafd 5

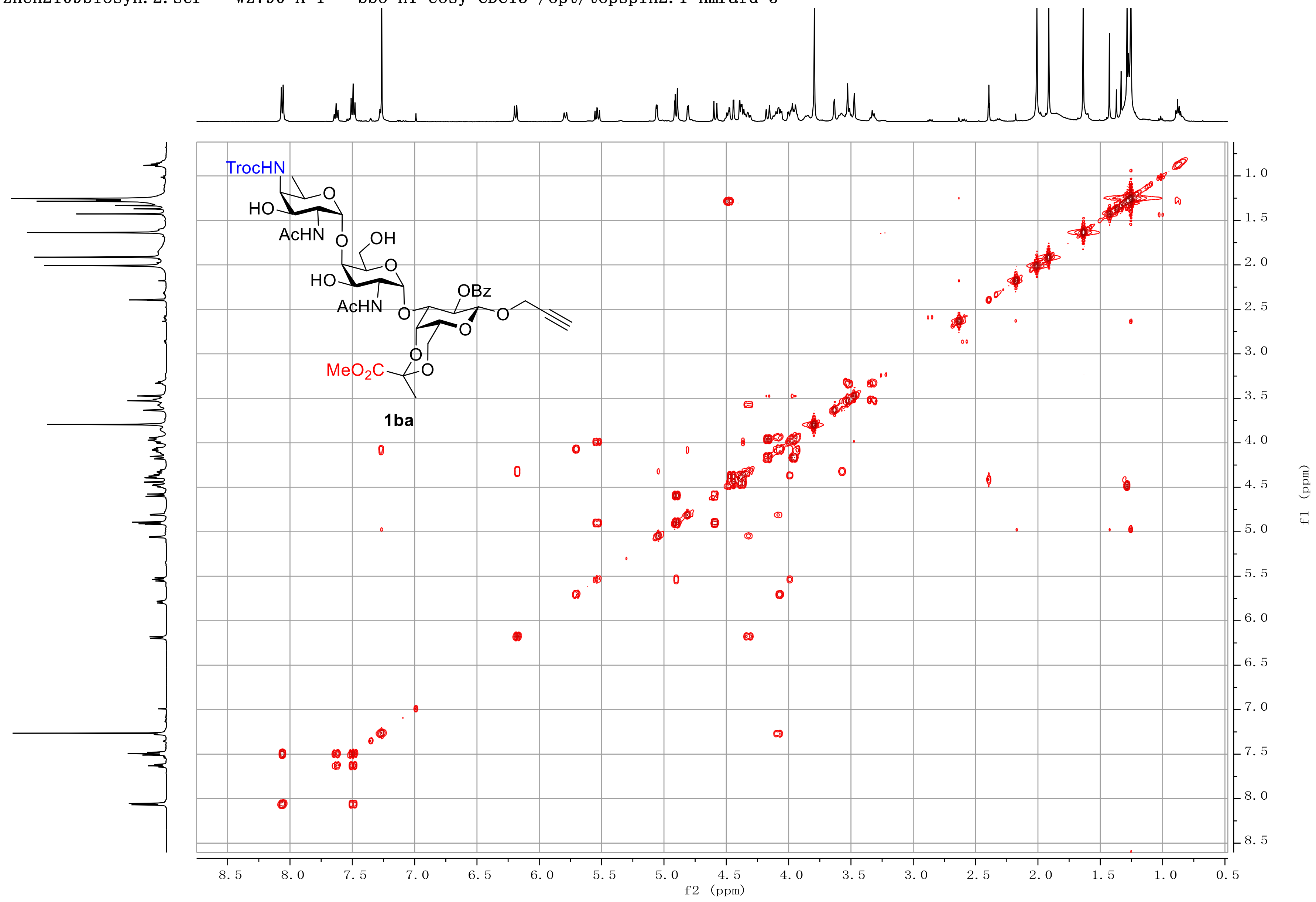

zhen2109biosyn.13.ser — wz790-B — bbo-c13-HSQC CDCl3 /opt/topspin2.1 nmrafd 7

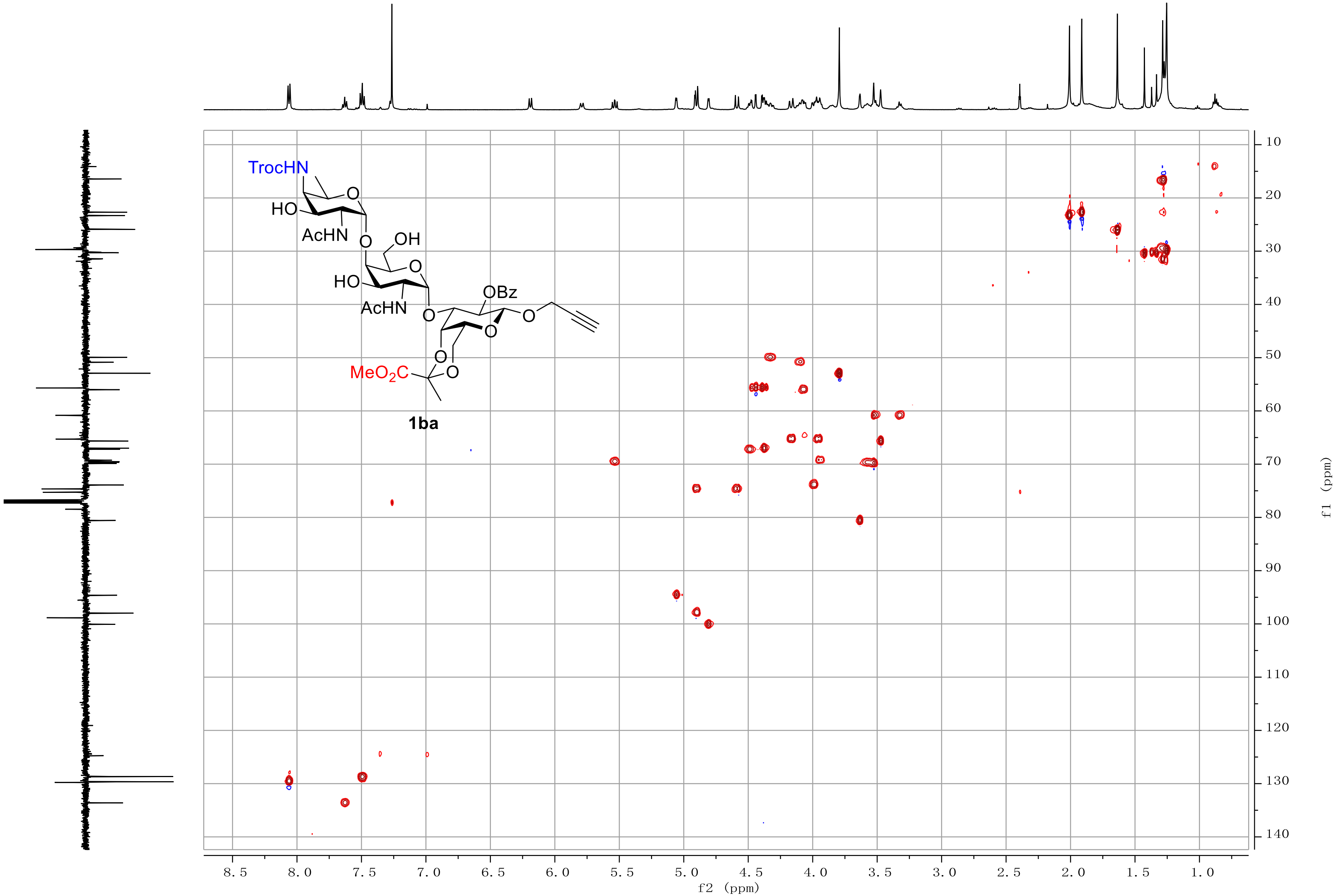

zhen2109biosyn.15.ser — wz790-B — bbo-c13-HMBC CDCl3 /opt/topspin2.1 nmrafd 7

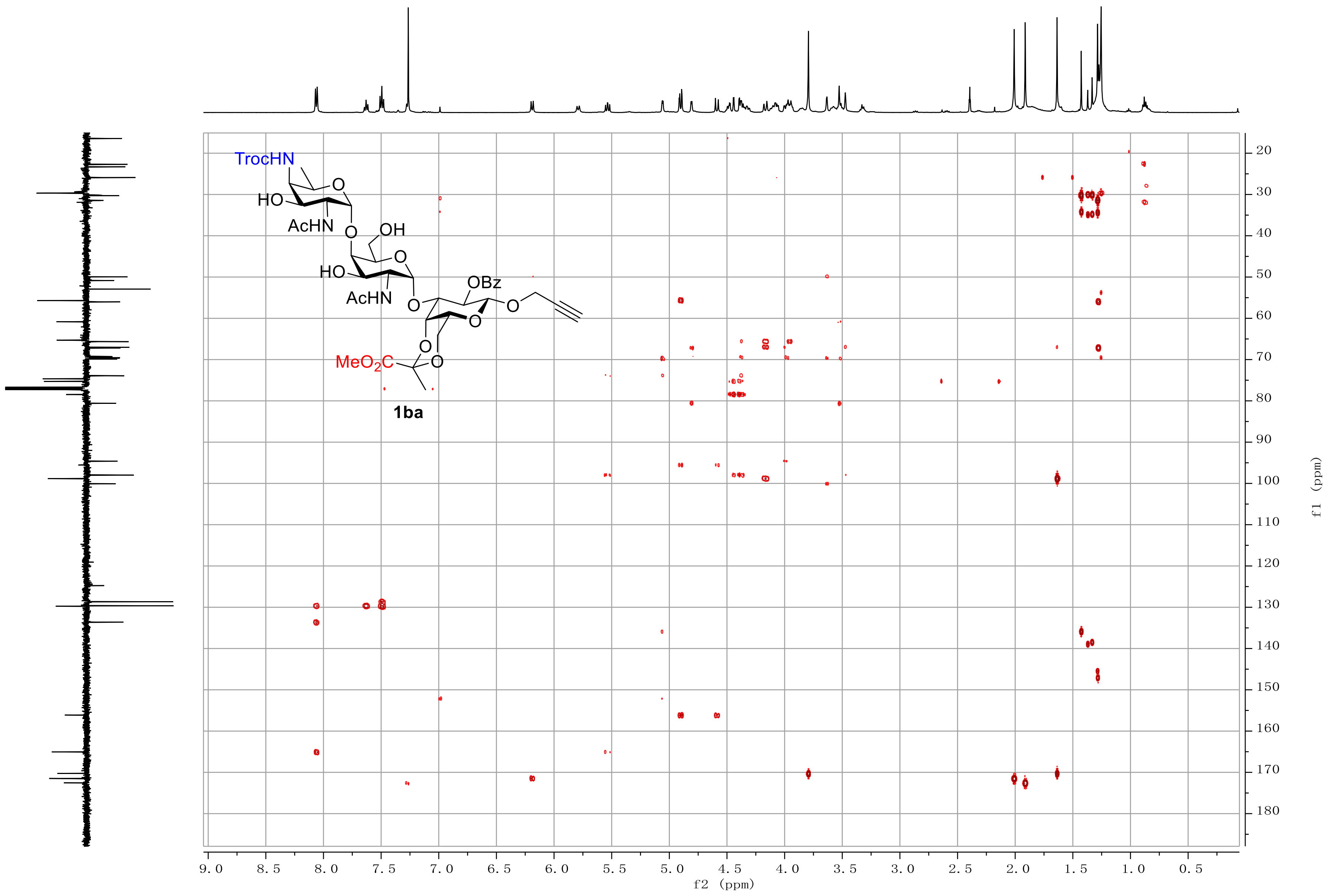

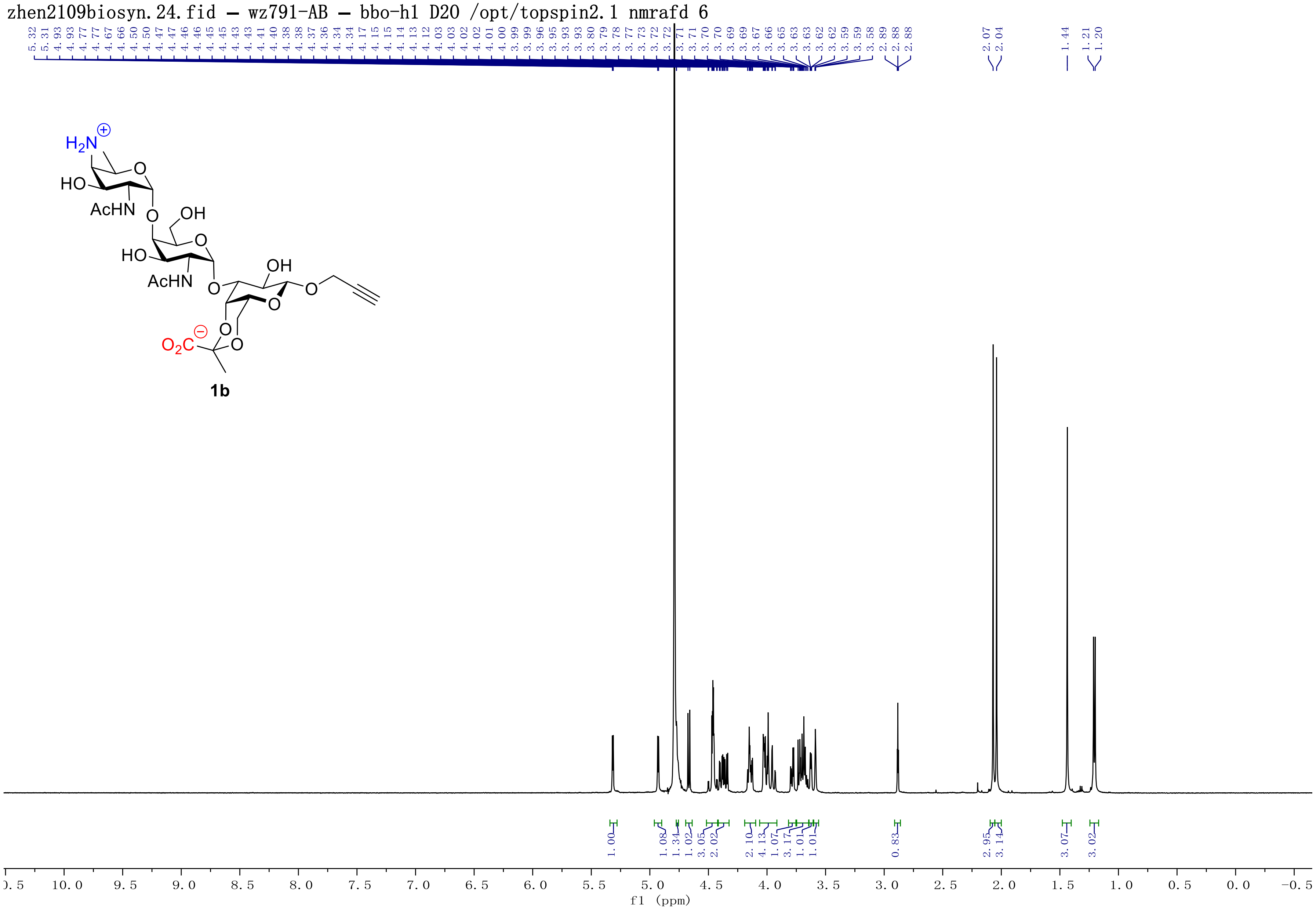
zhen2109biosyn.24.fid — wz791-AB — bbo-h1 D2O /opt/topspin2.1 nmrafd 6
5.32 5.31 4.93 4.93 4.77 4.77 4.67 4.66 4.50 4.50 4.47 4.47 4.46 4.46 4.45 4.45 4.43 4.43 4.41 4.40 4.38 4.38 4.37 4.36 4.34 4.34 4.17 4.15 4.15 4.14 4.13 4.12 4.03 4.03 4.02 4.02 4.01 4.00 3.99 3.96 3.95 3.93 3.93 3.80 3.79 3.78 3.77 3.73 3.72 3.72 3.71 3.71 3.70 3.70 3.69 3.69 3.67 3.66 3.65 3.63 3.63 3.62 3.59 3.59 3.58 2.89 2.88 2.88 2.07 2.04 1.44 1.21 1.20
1.00 1.08 1.34 1.02 3.05 2.02 2.10 4.13 1.07 3.17 1.01 1.01 0.83 2.95 3.14 3.07 3.02
f1 (ppm)
1b

zhen2109biosyn.27.fid — wz791-AB — bbo-c13-APT D2O /opt/topspin2.1 nmrafd 6

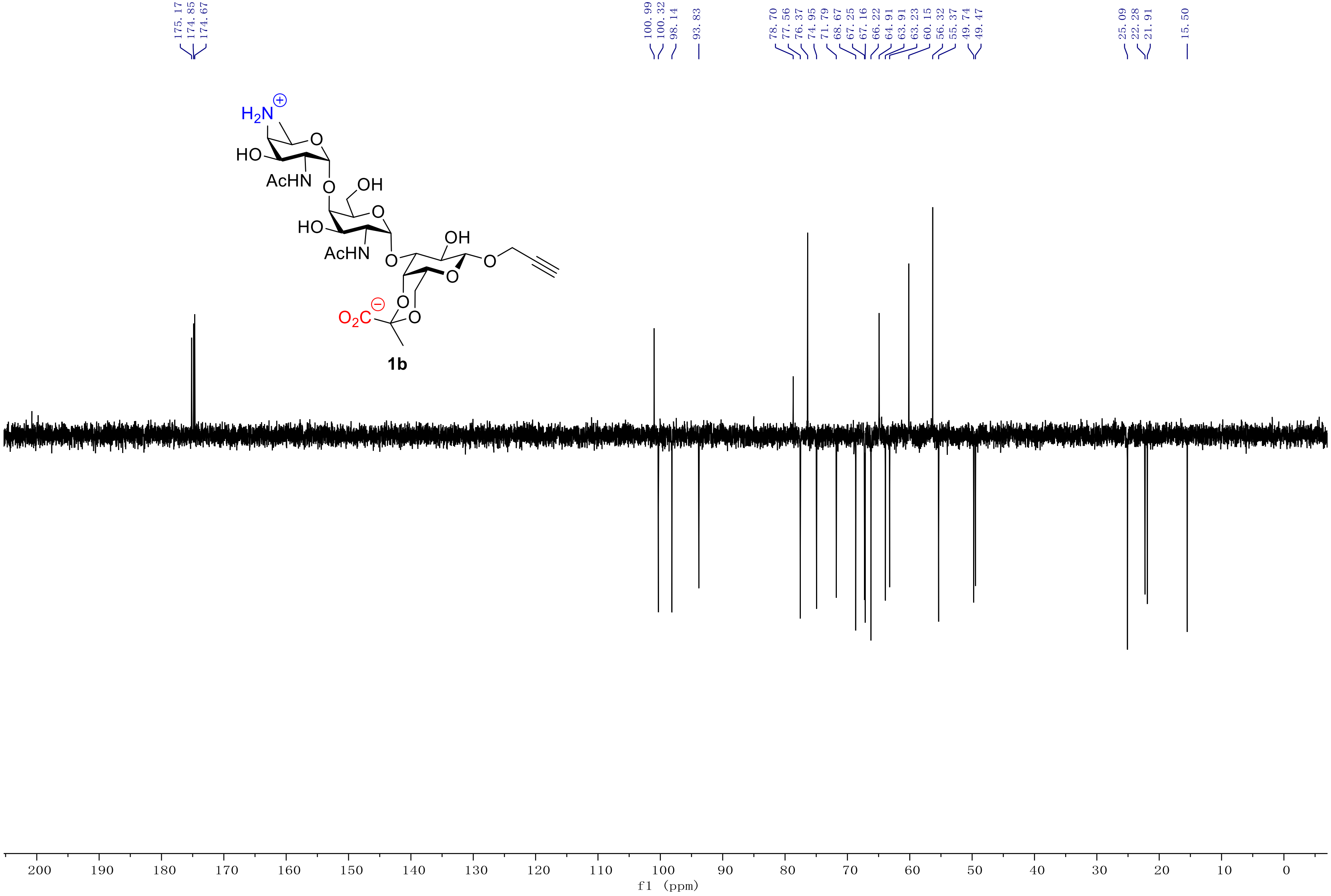

zhen2109biosyn.25.ser — wz791-AB — bbo-h1-cosy D2O /opt/topspin2.1 nmrafd 6

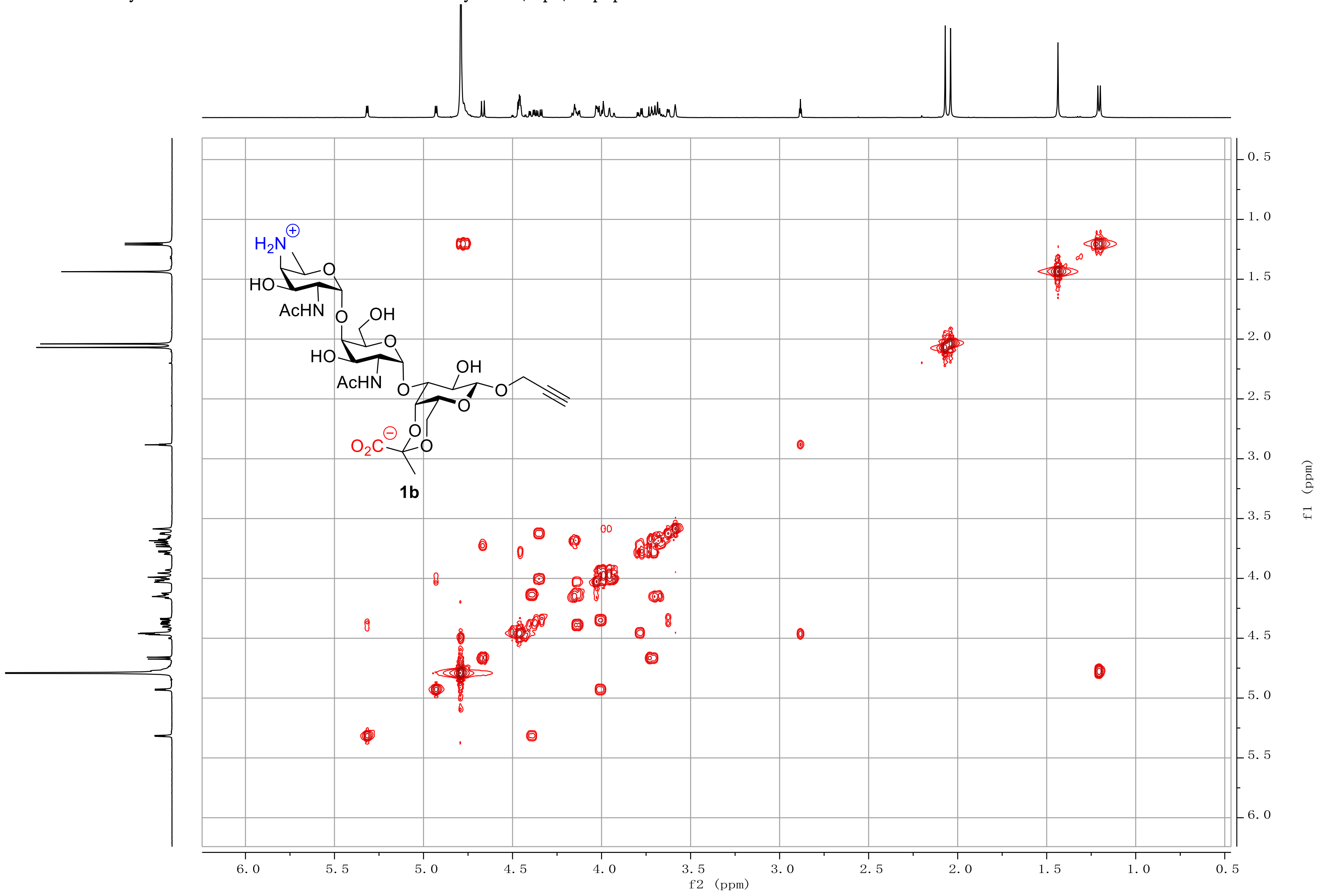

zhen2109biosyn.26.ser — wz791-AB — bbo-c13-HSQC D20 /opt/topspin2.1 nmrafd 6

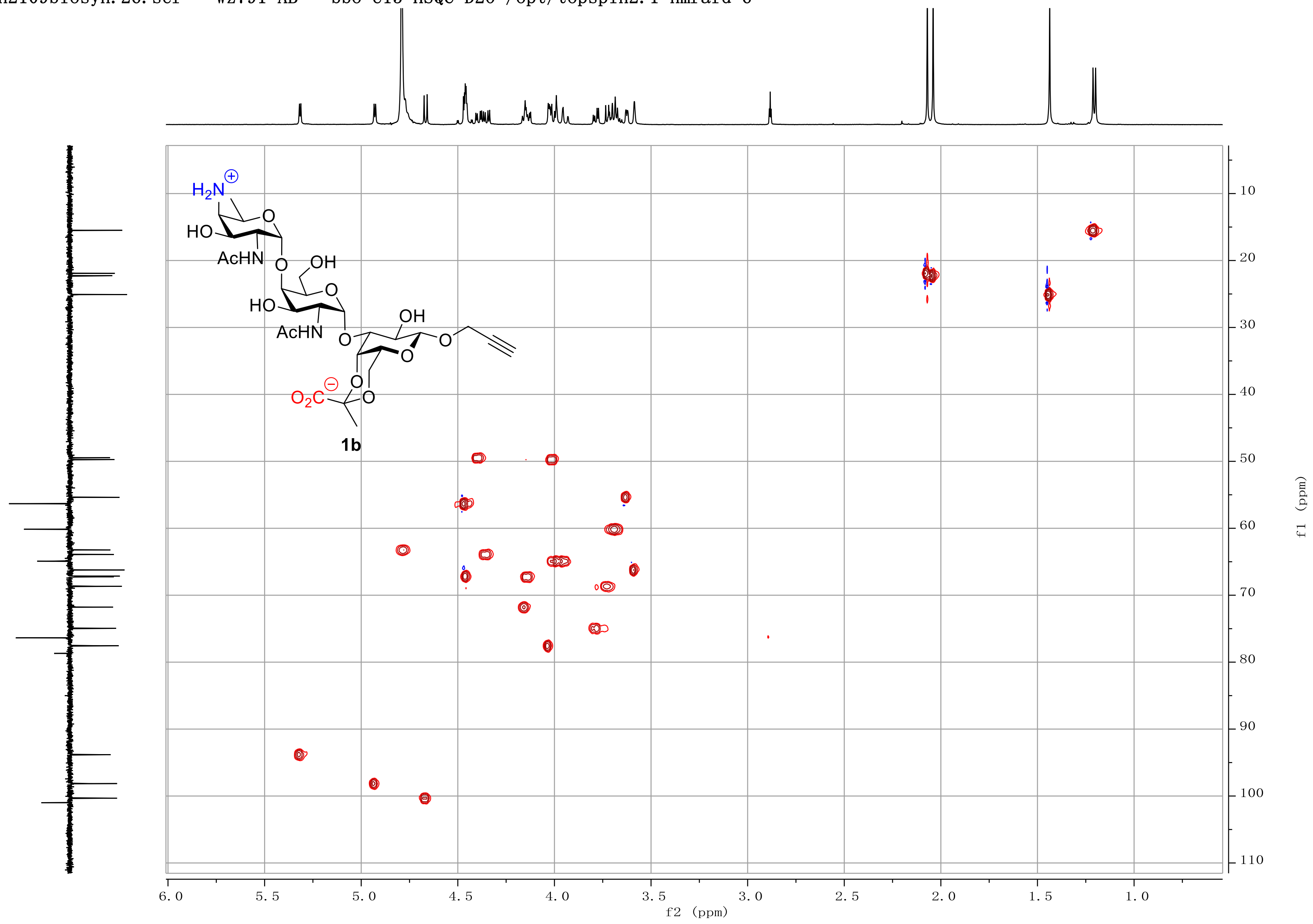

zhen2109biosyn.28.ser — wz791-AB — bbo-c13-HMBC D20 /opt/topspin2.1 nmrafd 6

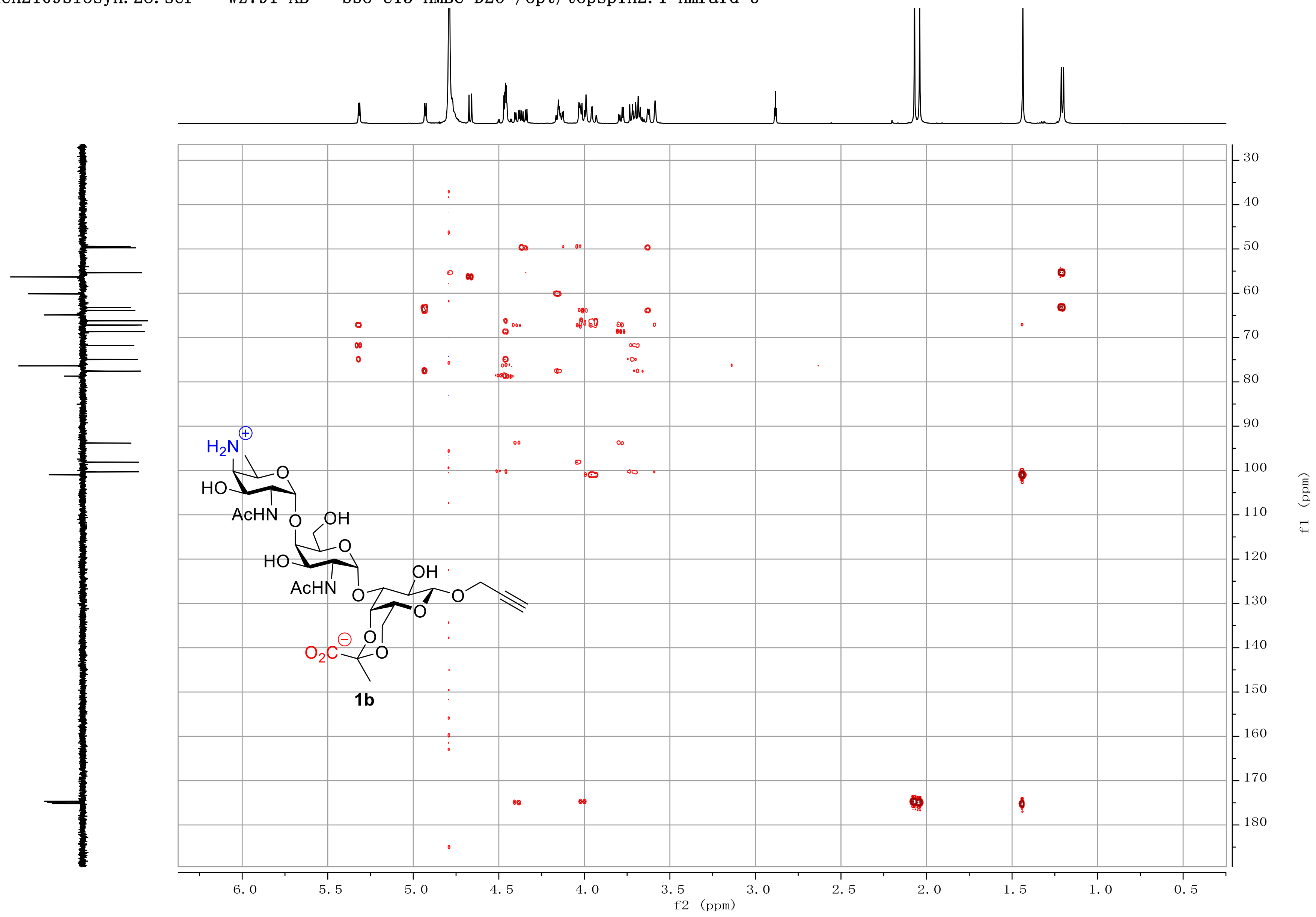

zhen2110biosyn.61.fid — wz812-a-s — bbo-h1 CDCl3 /opt/topspin2.1 nmrafd 2

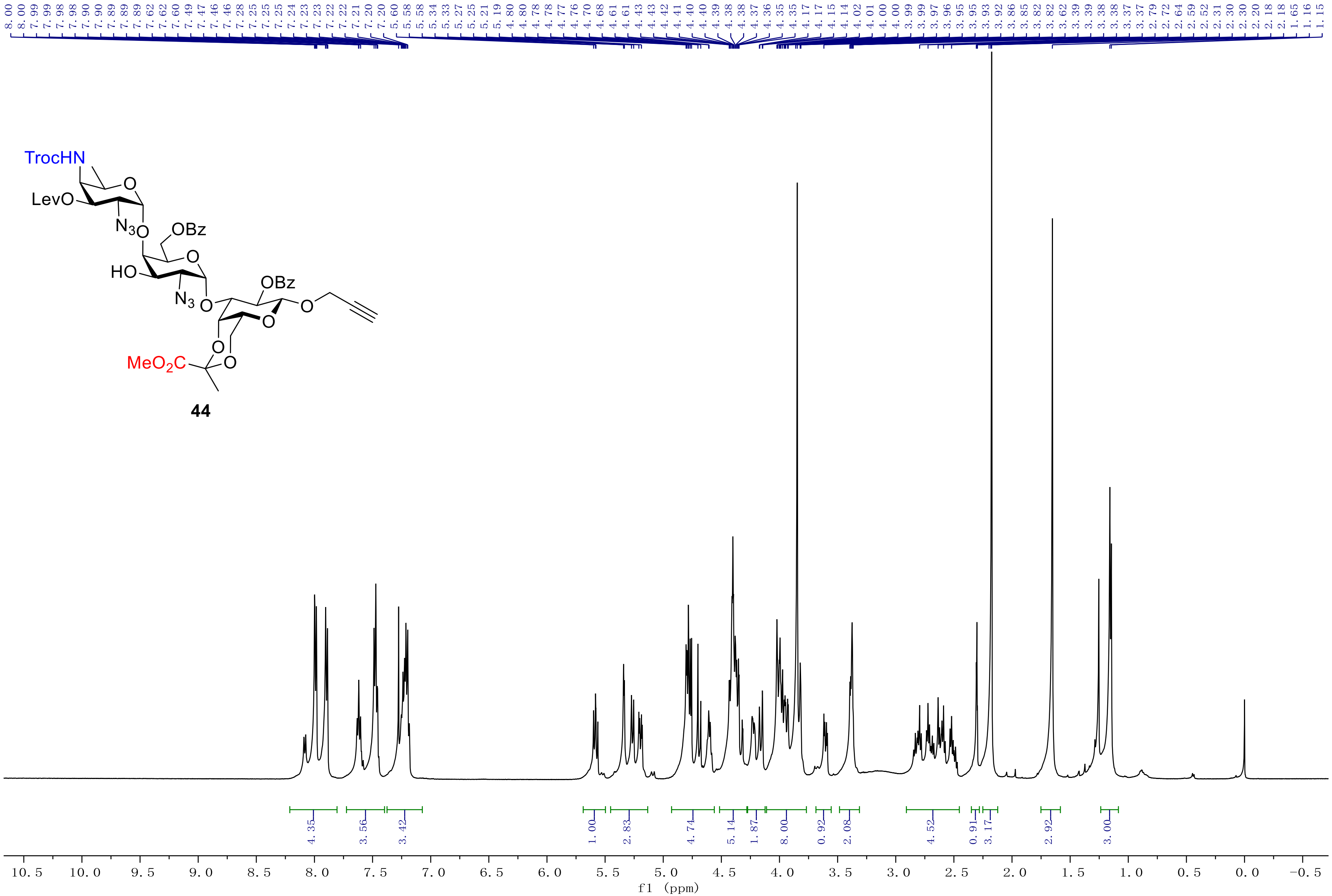

zhen2110biosyn.64.fid — wz812-a-s — bbo-c13-APT CDCl3 /opt/topspin2.1 nmrafd 2

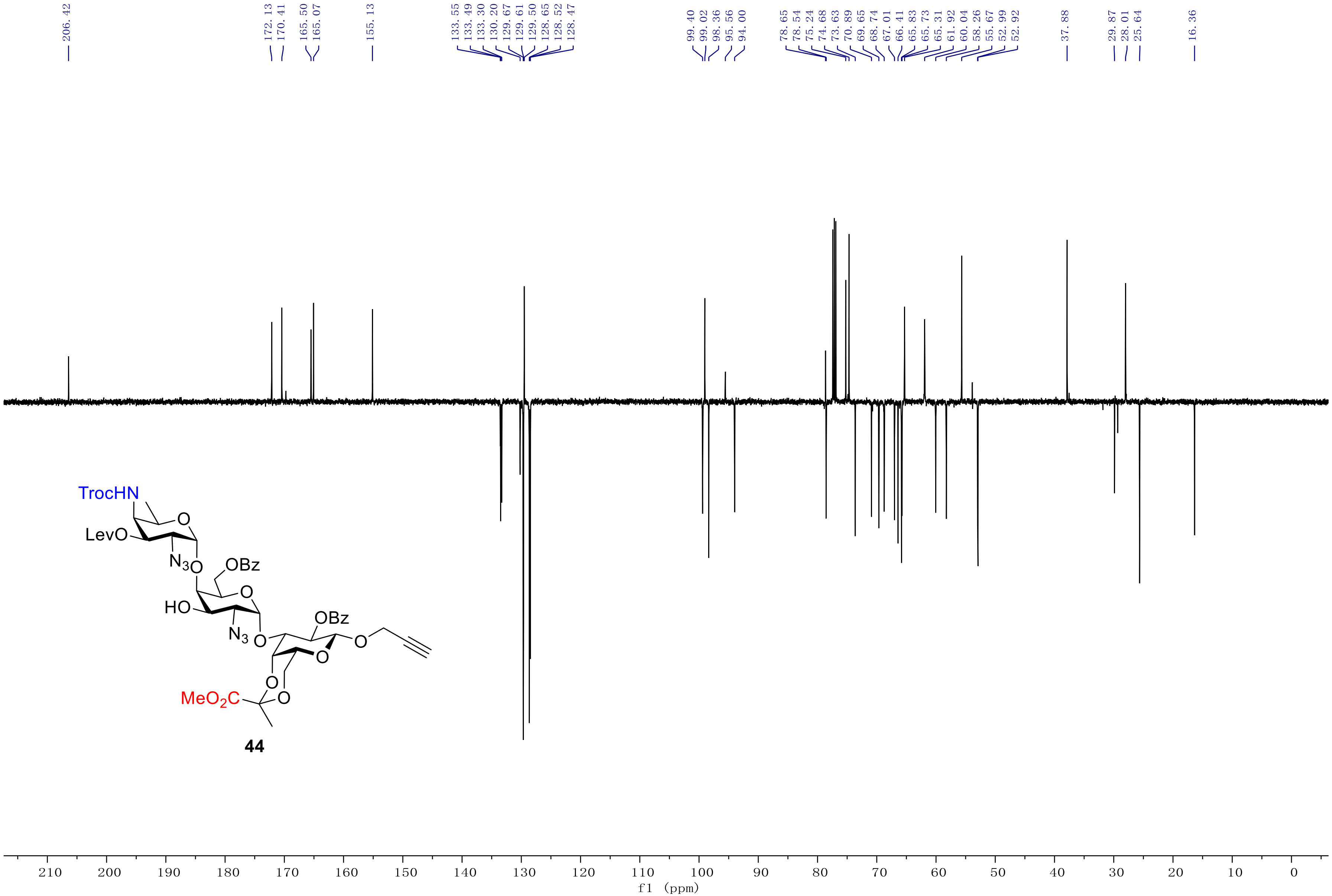

zhen2110biosyn.62.ser — wz812-a-s — bbo-h1-cosy CDCl3 /opt/topspin2.1 nmrafd 2

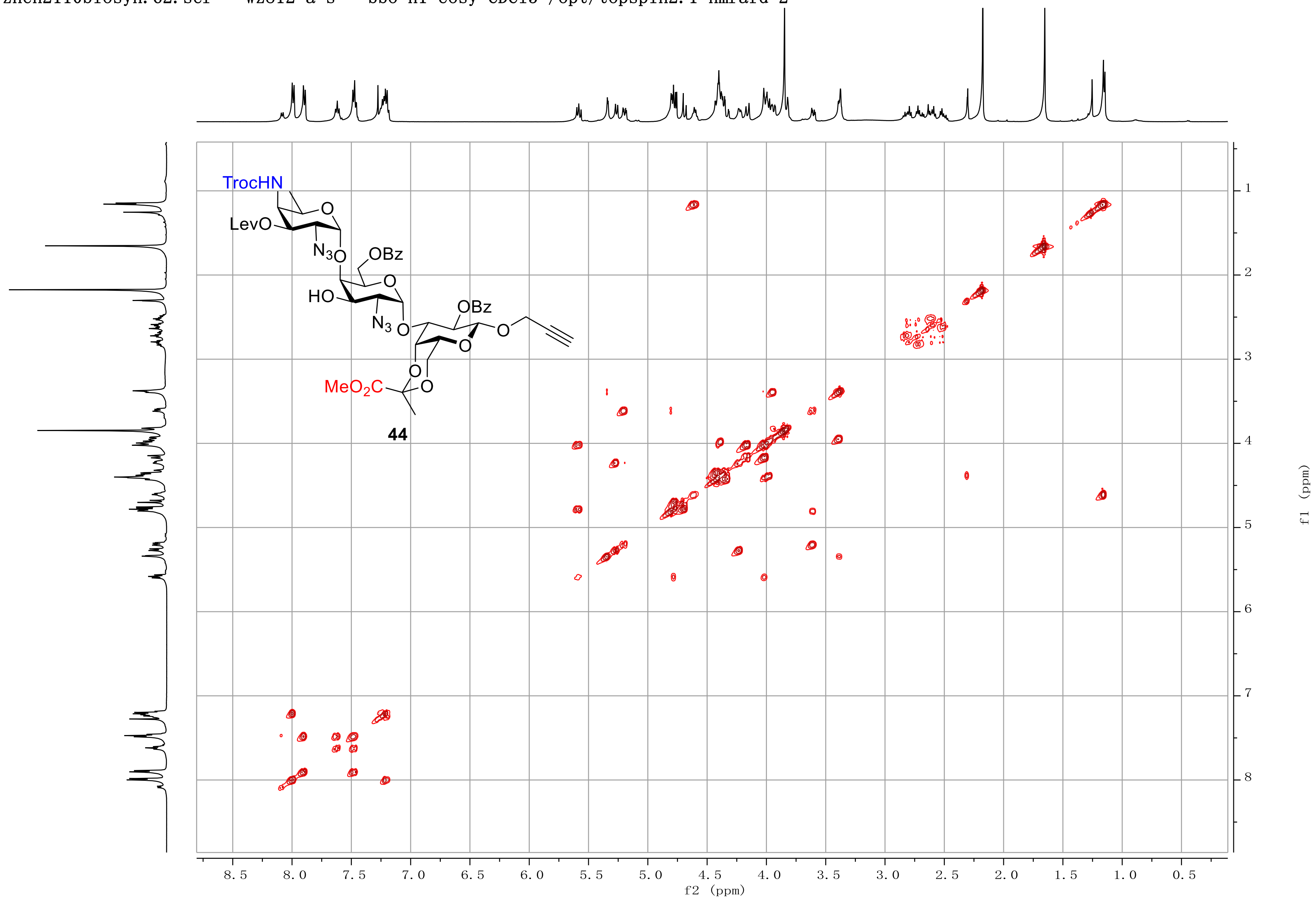

zhen2110biosyn.63.ser — wz812-a-s — bbo-c13-HSQC CDCl3 /opt/topspin2.1 nmrafd 2

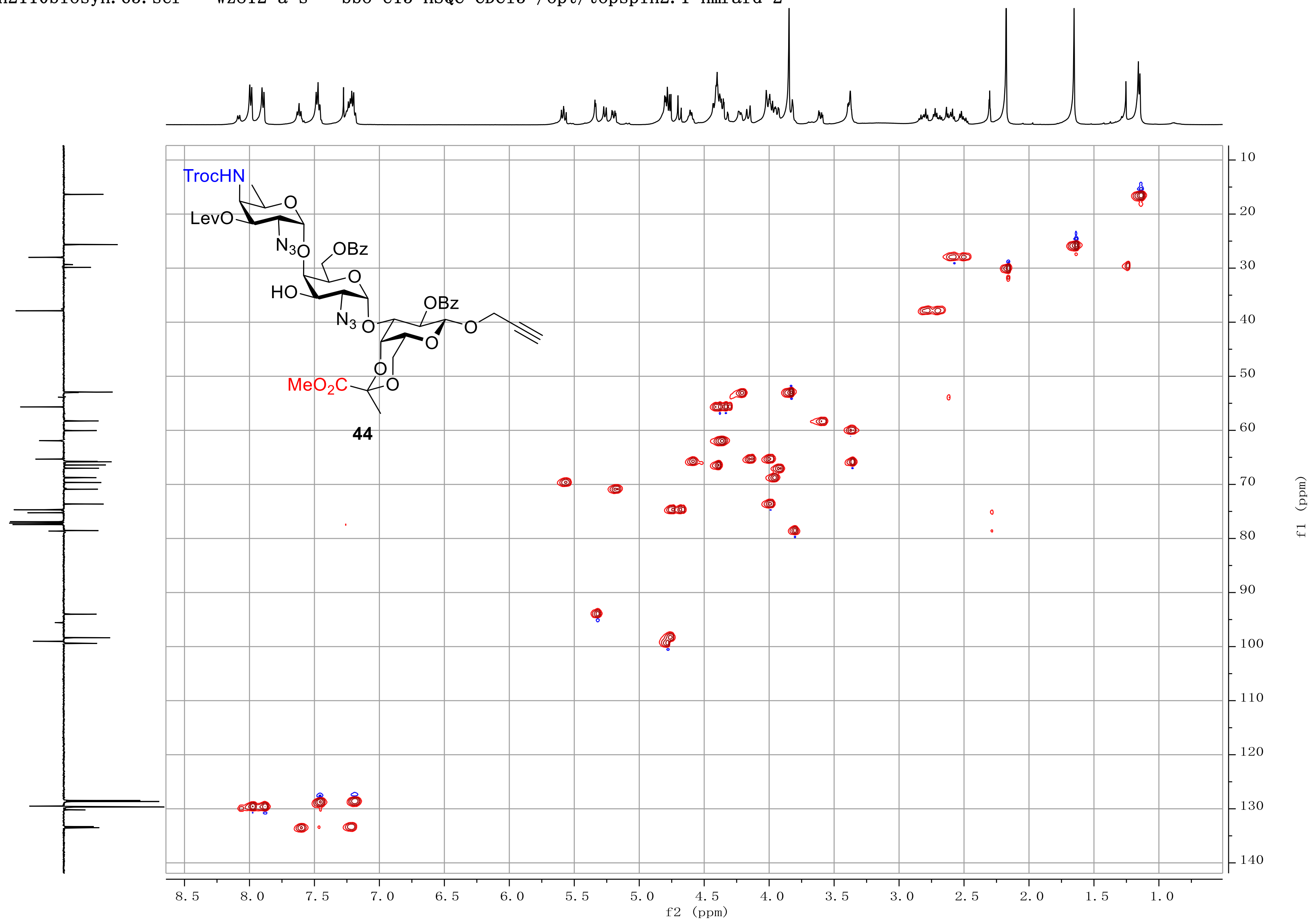

zhen2110biosyn.65.ser — wz812-a-s — bbo-c13-HMBC CDCl3 /opt/topspin2.1 nmrafd 2

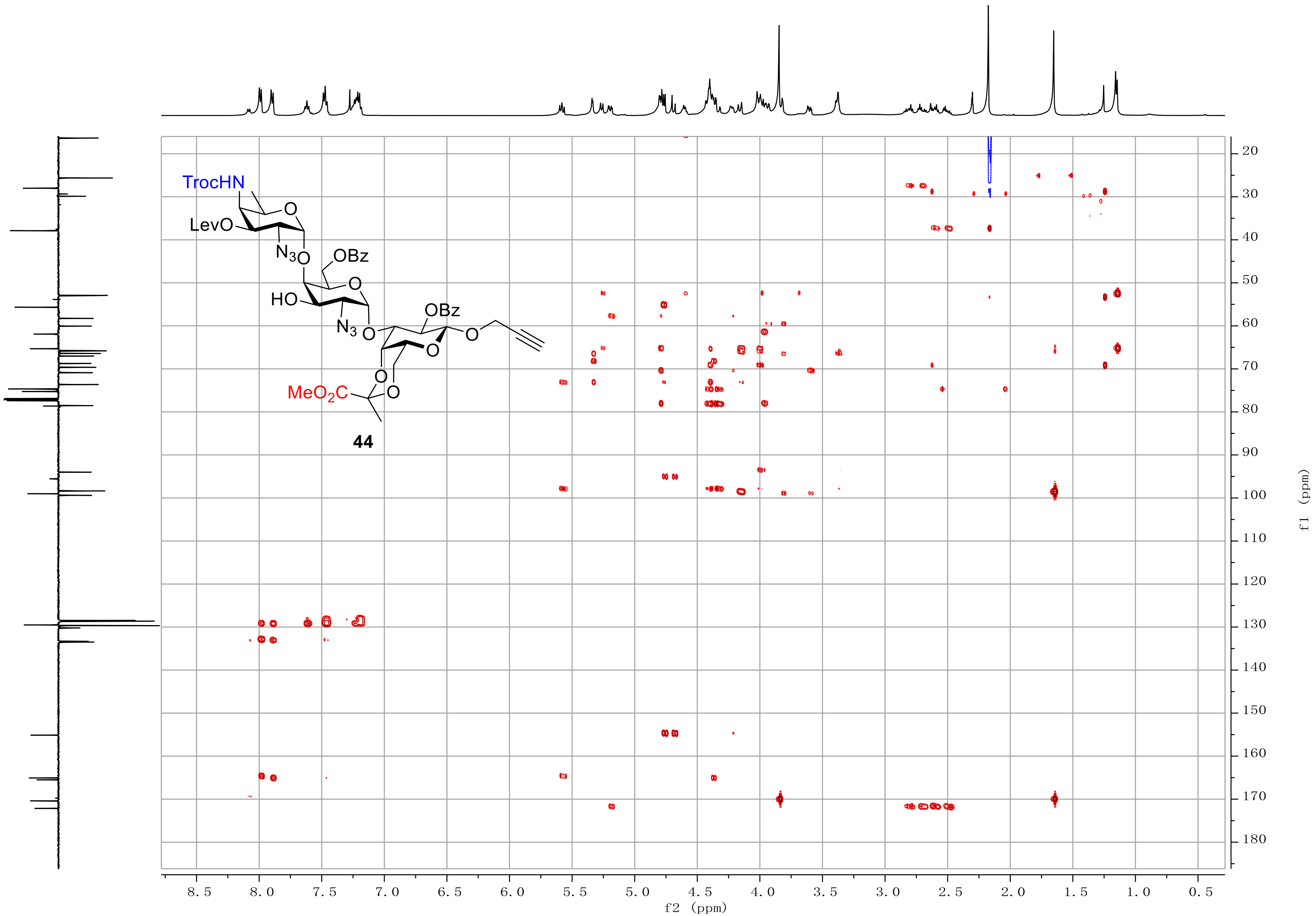

zhen2110biosyn.66.ser — wz812-a-s — bbo-c13-hmbc-ipv-gated CDCl3 /opt/topspin2.1 nmrafd 2

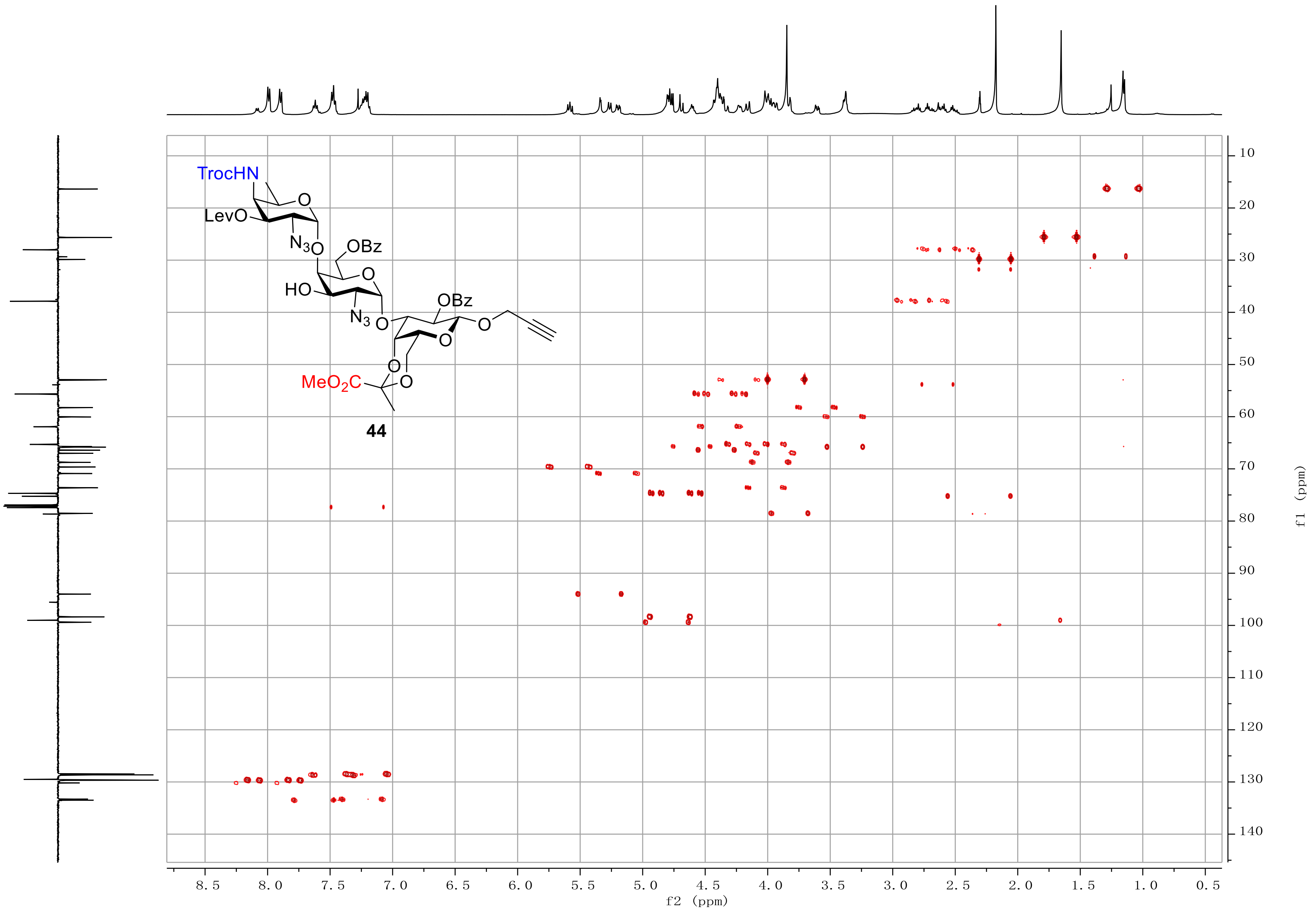

2110zhen.22.fid — wz814-a-s — h1 CDCl3 /opt/DATA nmrafd 18

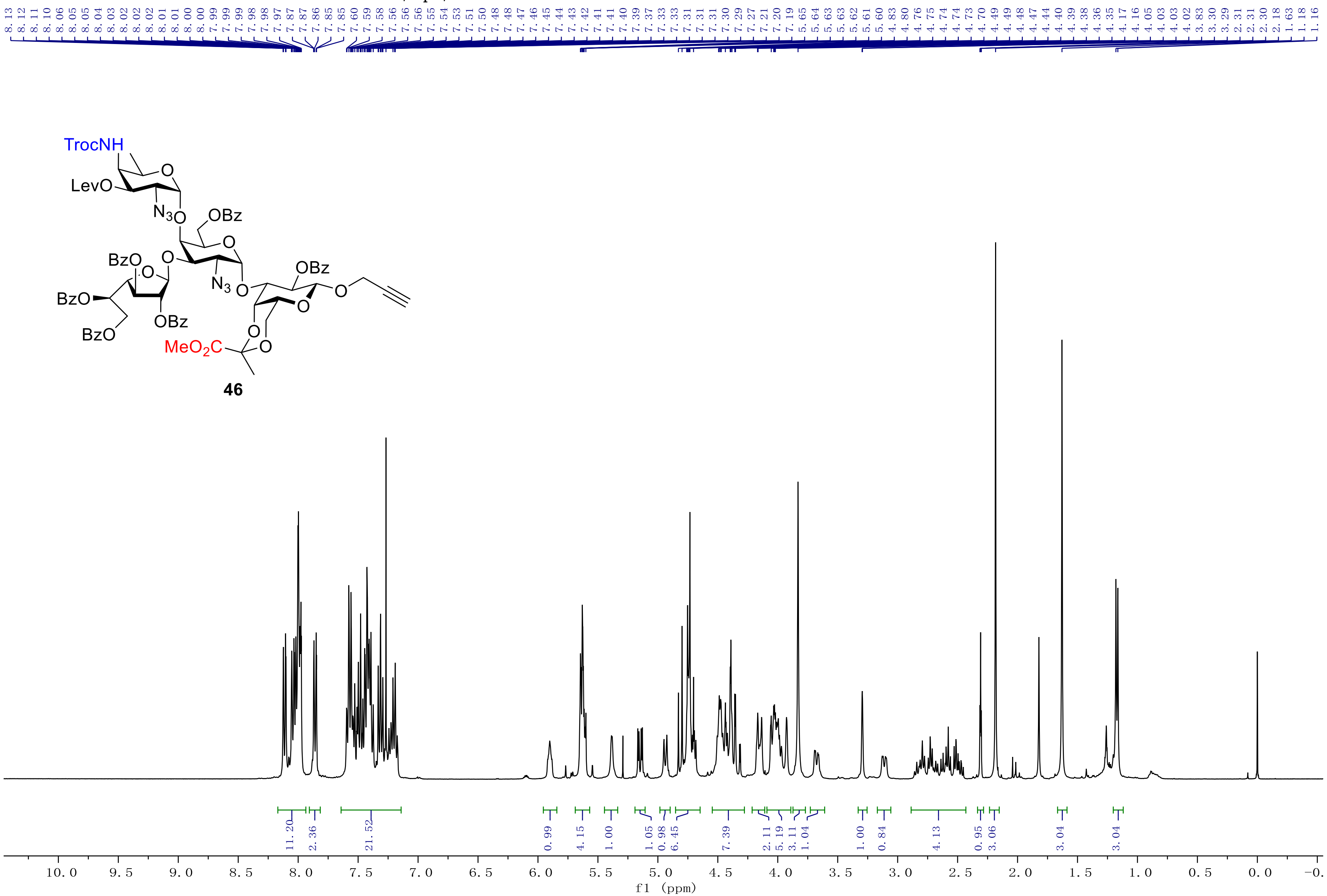

2110zhen.23.fid — wz814-a-s — C13APT CDCl3 /opt/DATA nmrafd 18

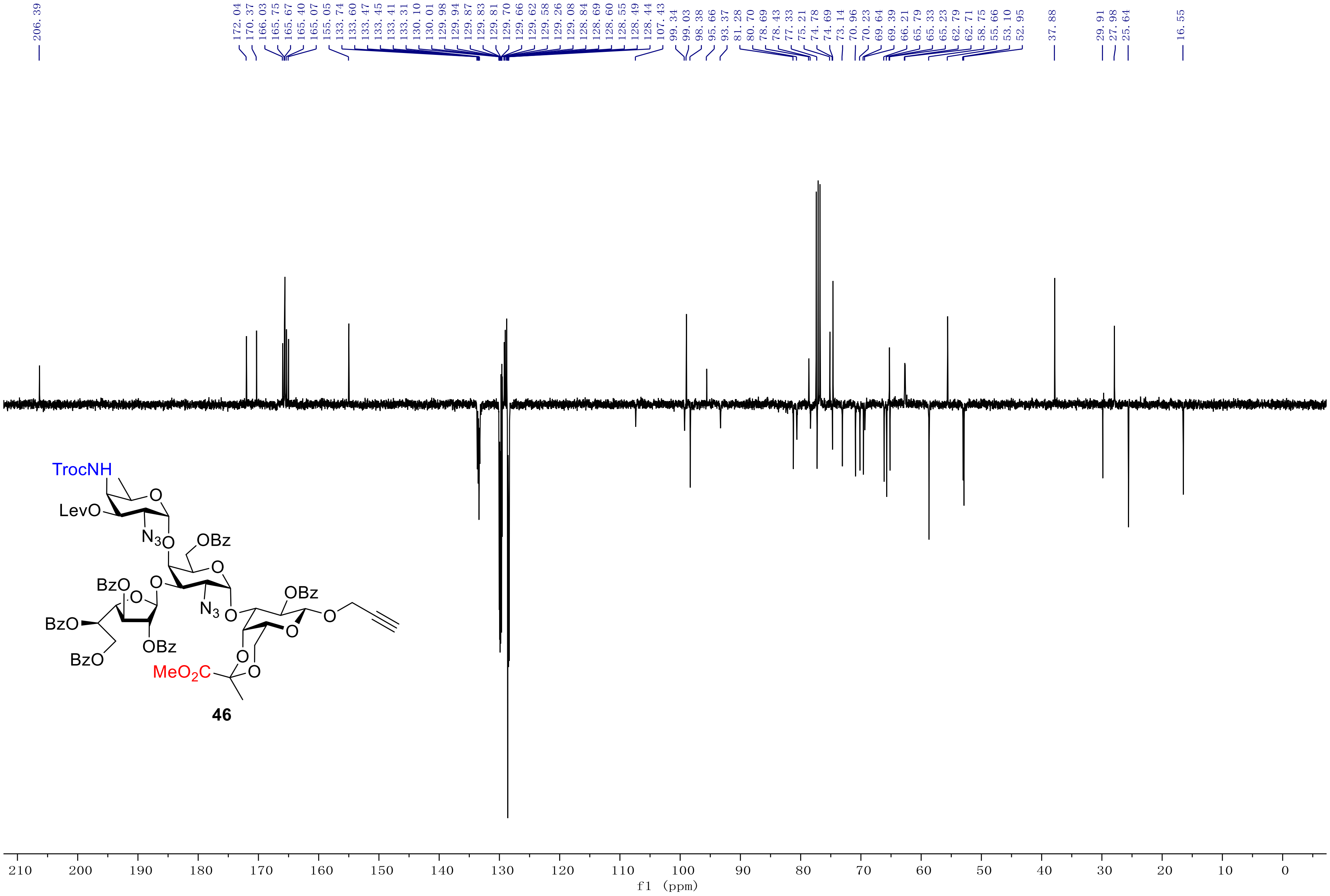

2110zhen.24.ser — wz814-a-s — h1COSY CDCl3 /opt/DATA nmrafd 18

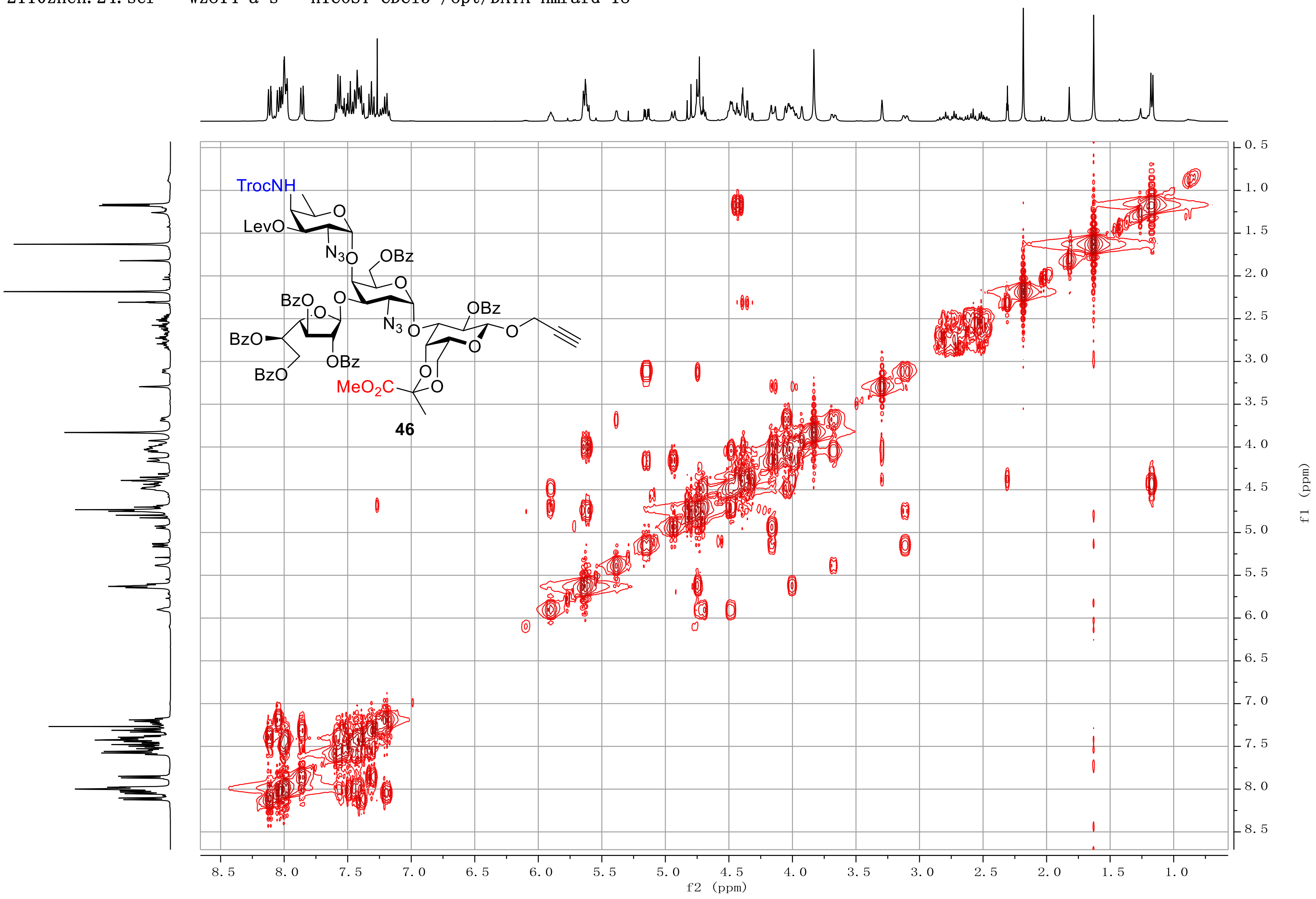

2110zhen.25.ser — wz814-a-s — c13HSQC CDCl3 /opt/DATA nmrafd 18

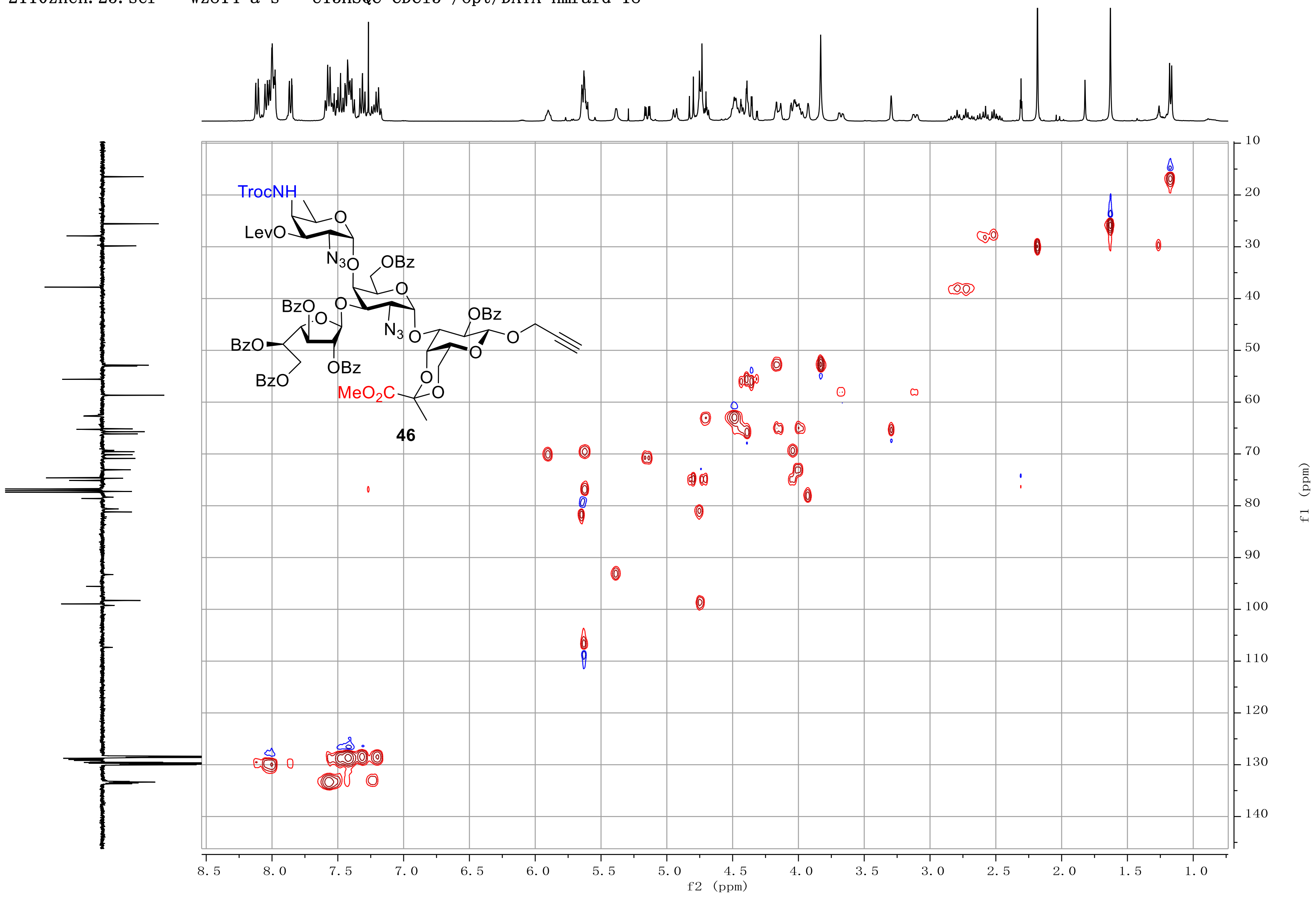

2110zhen.26.ser — wz814-a-s — c13HMBC CDCl3 /opt/DATA nmrafd 18

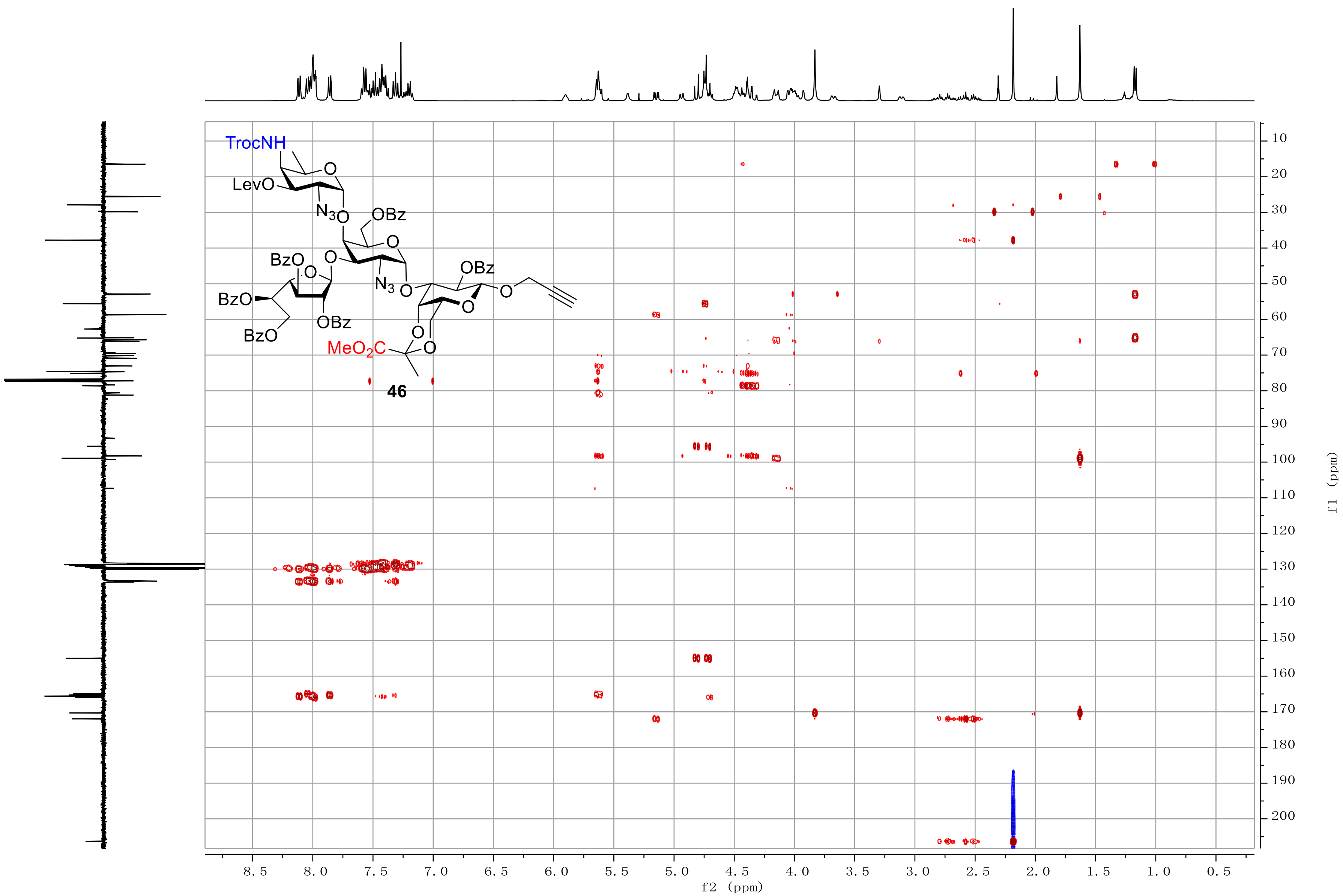

2110zhen.27.ser — wz814-a-s — c13HMBCipvGATED CDCl3 /opt/DATA nmrafd 18

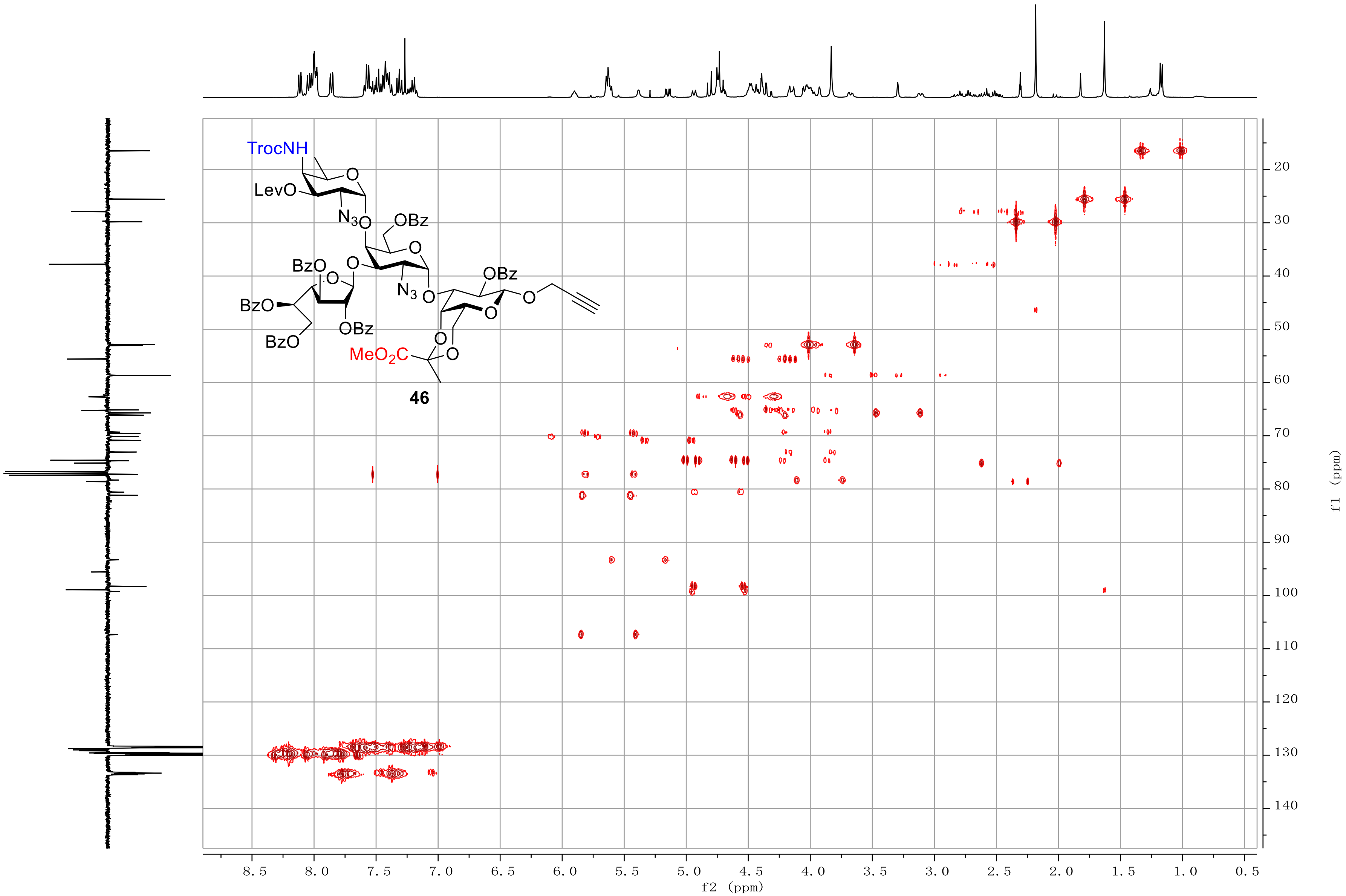

zhen2111biosyn.25.fid — wz815-B-s — bbo-h1 CDCl3 /opt/topspin2.1 nmrafd 12

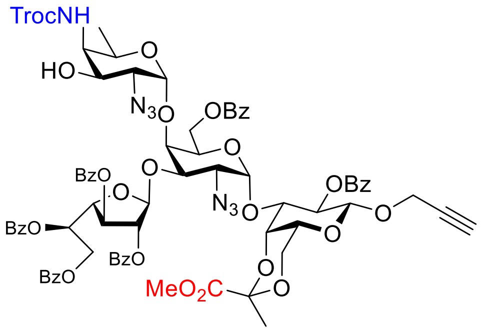

47a

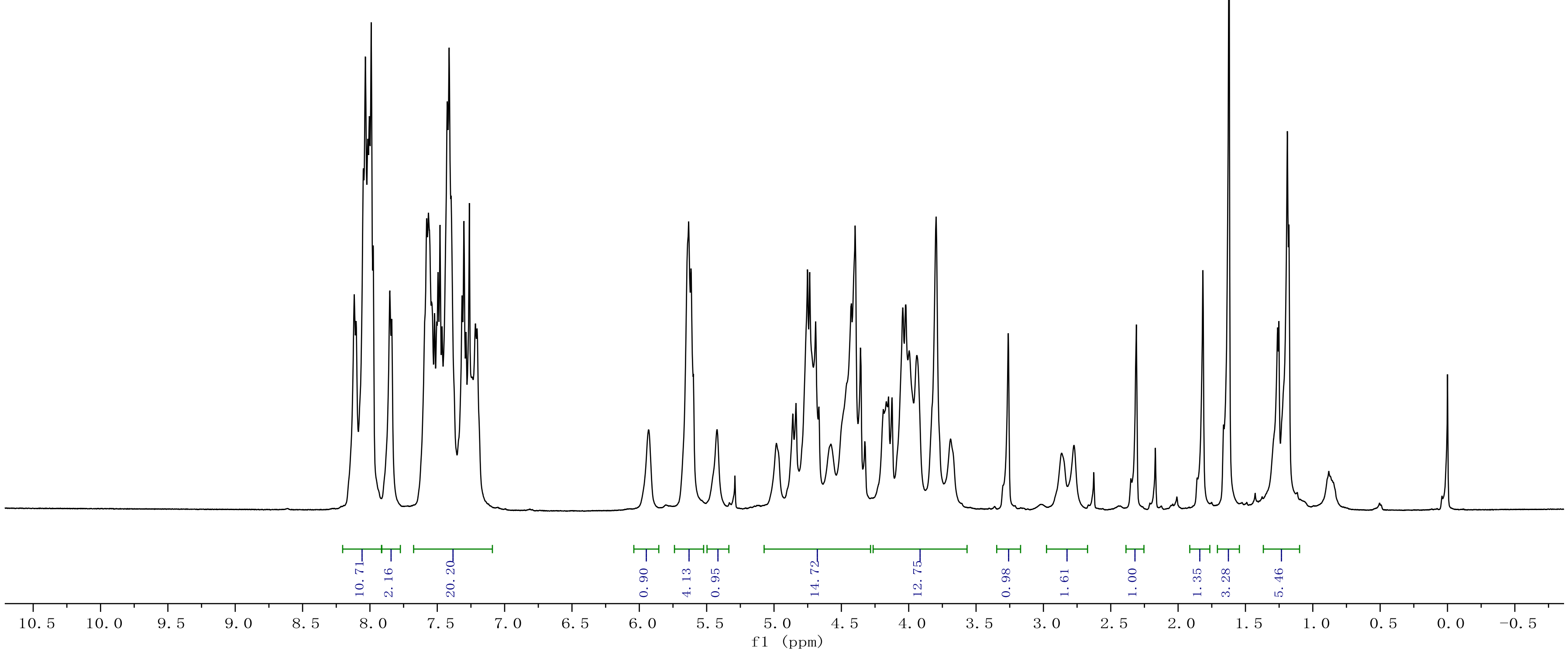

zhen2111biosyn.28.fid — wz815-B-s — bbo-c13-APT CDCl3 /opt/topspin2.1 nmrafd 12

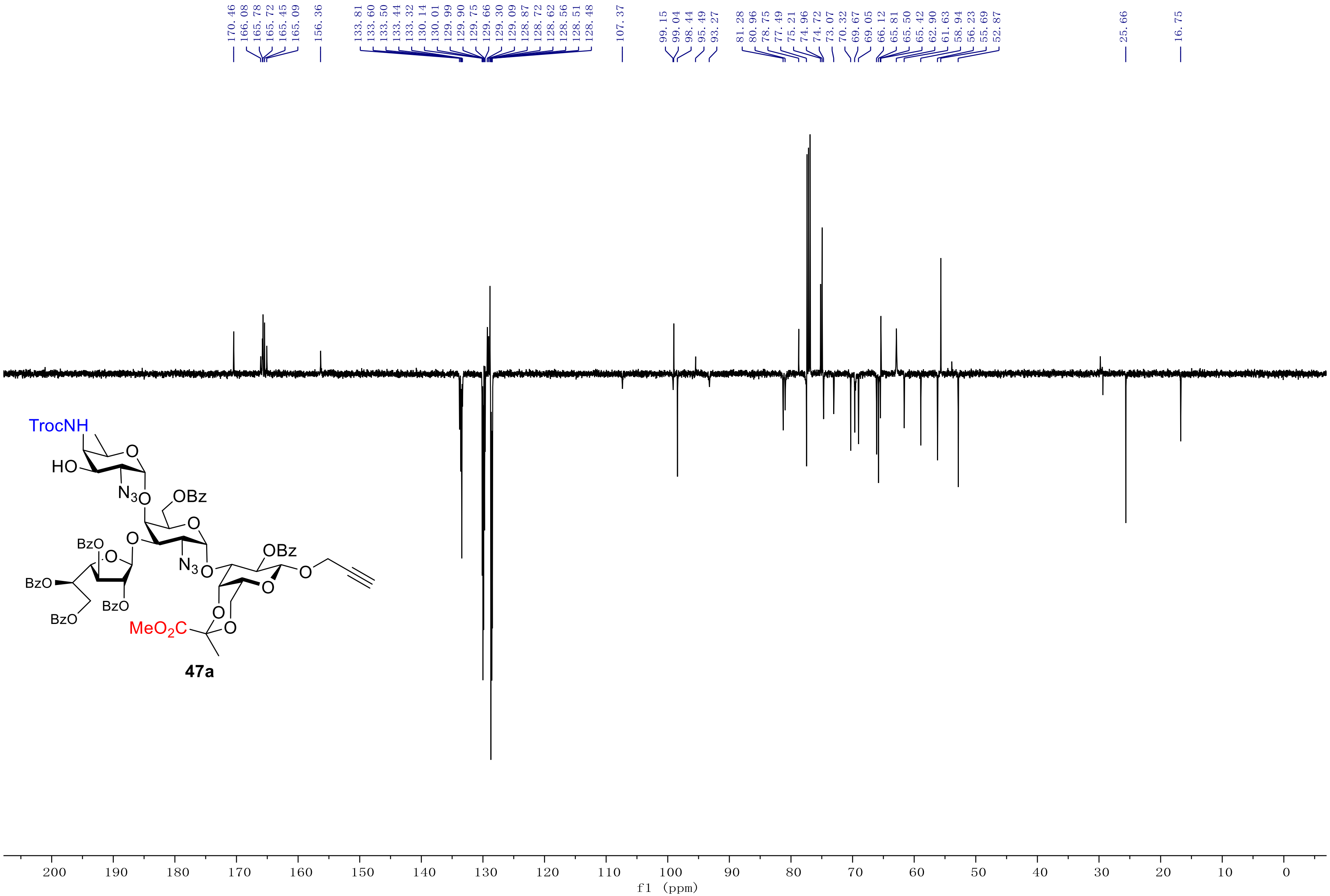

47a

zhen2111biosyn.26.ser — wz815-B-s — bbo-h1-cosy CDCl3 /opt/topspin2.1 nmrafd 12

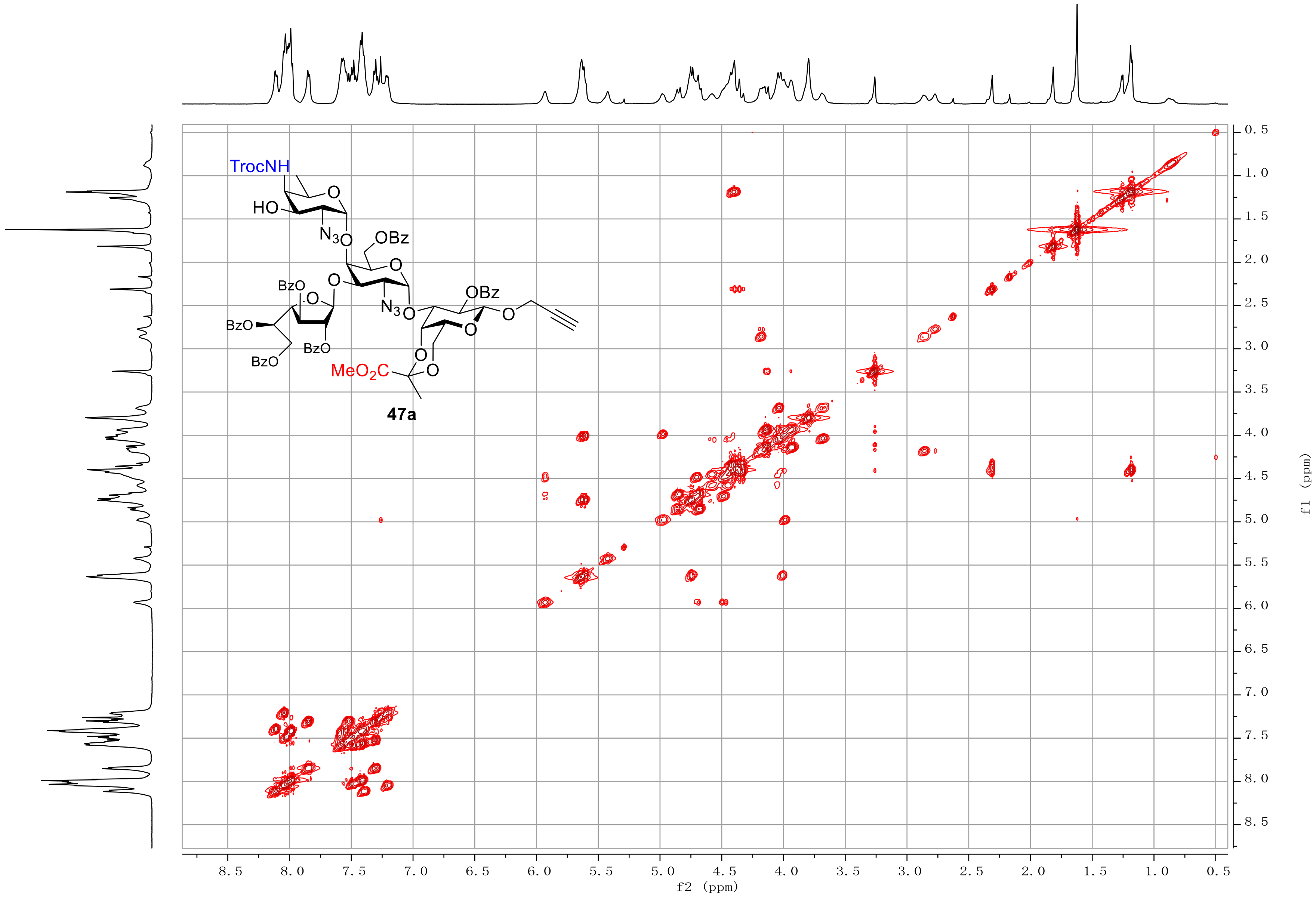

zhen2111biosyn.27.ser — wz815-B-s — bbo-c13-HSQC CDCl3 /opt/topspin2.1 nmrafd 12

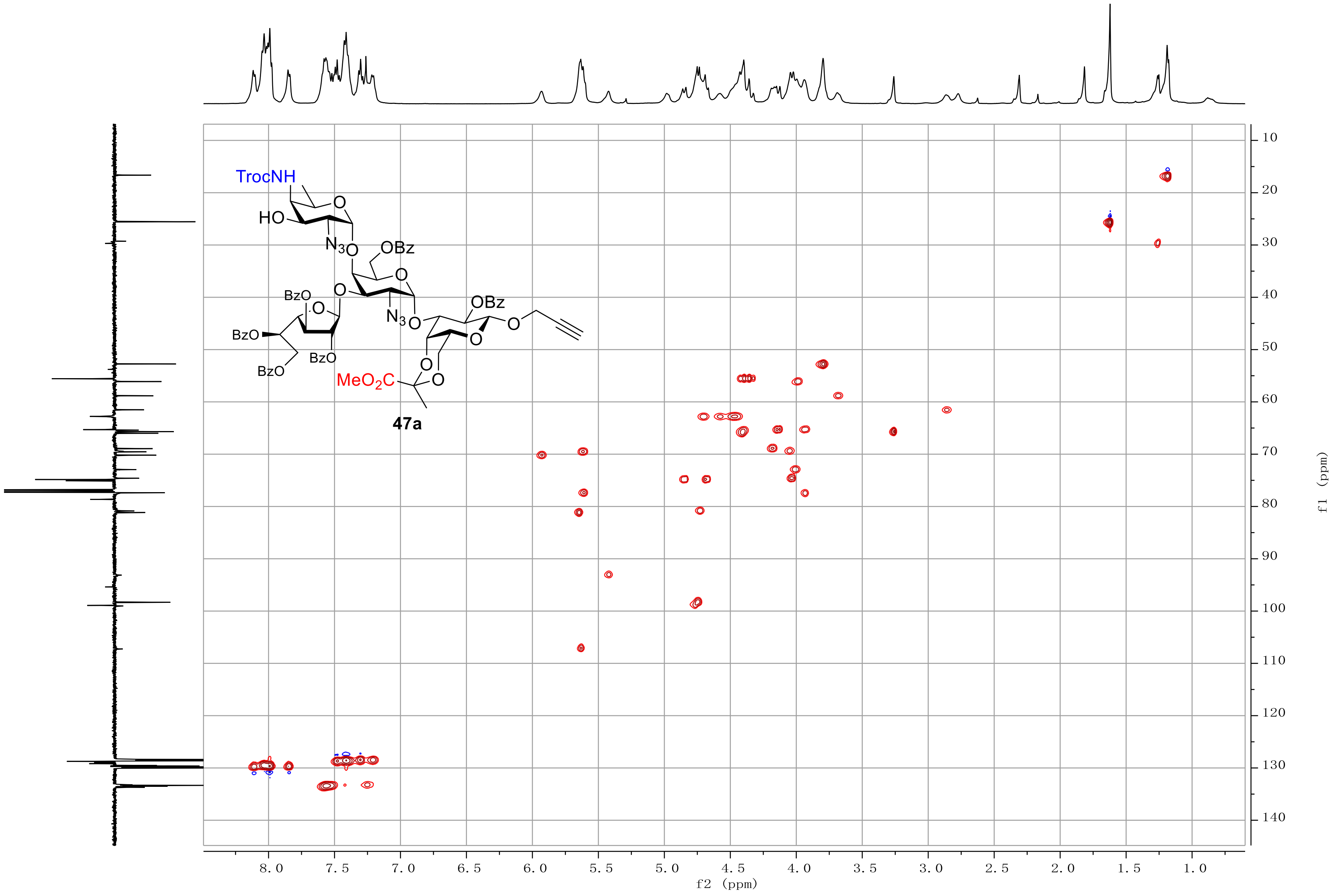

zhen2111biosyn.29.ser — wz815-B-s — bbo-c13-HMBC CDCl3 /opt/topspin2.1 nmrafd 12

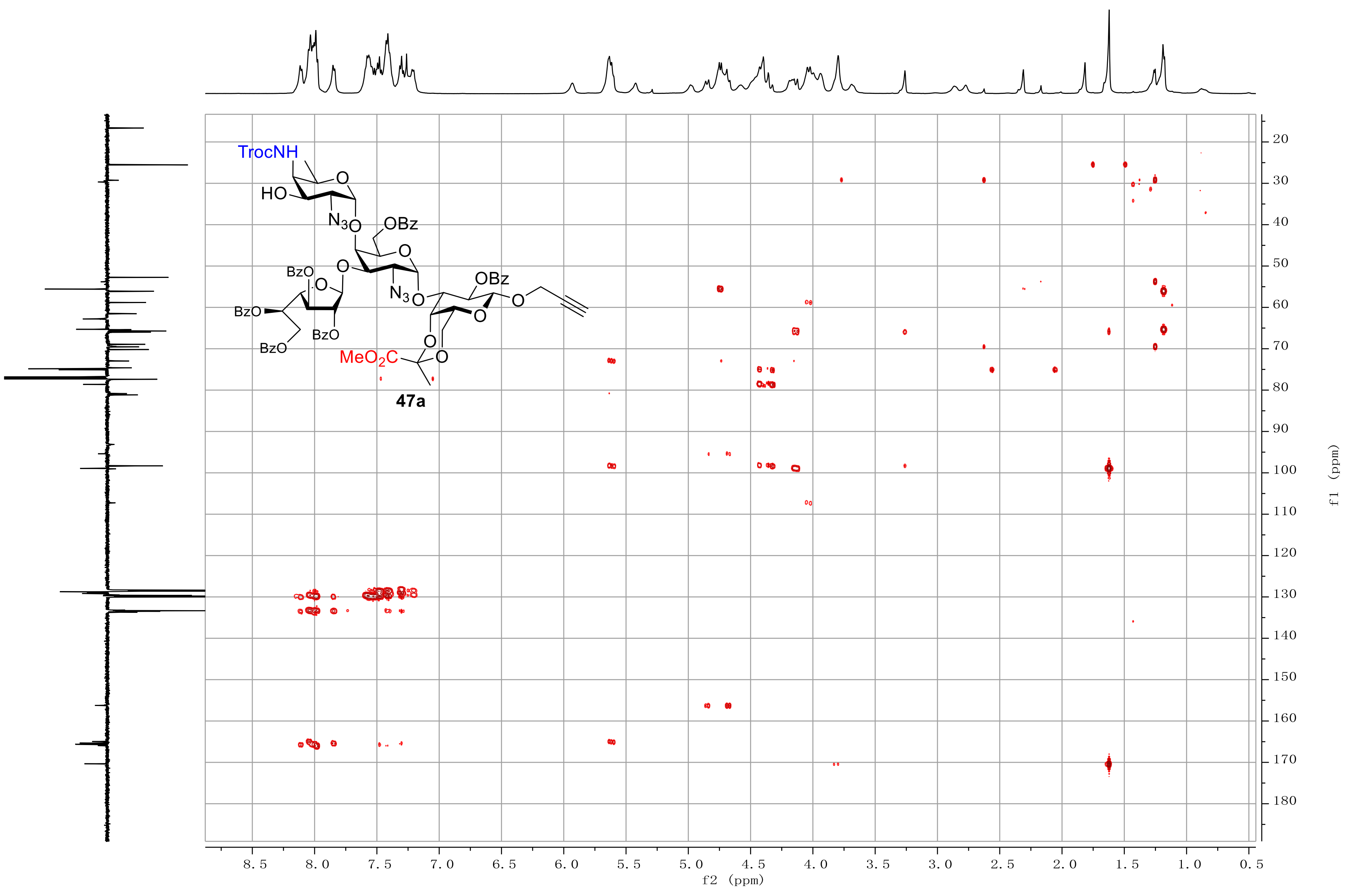

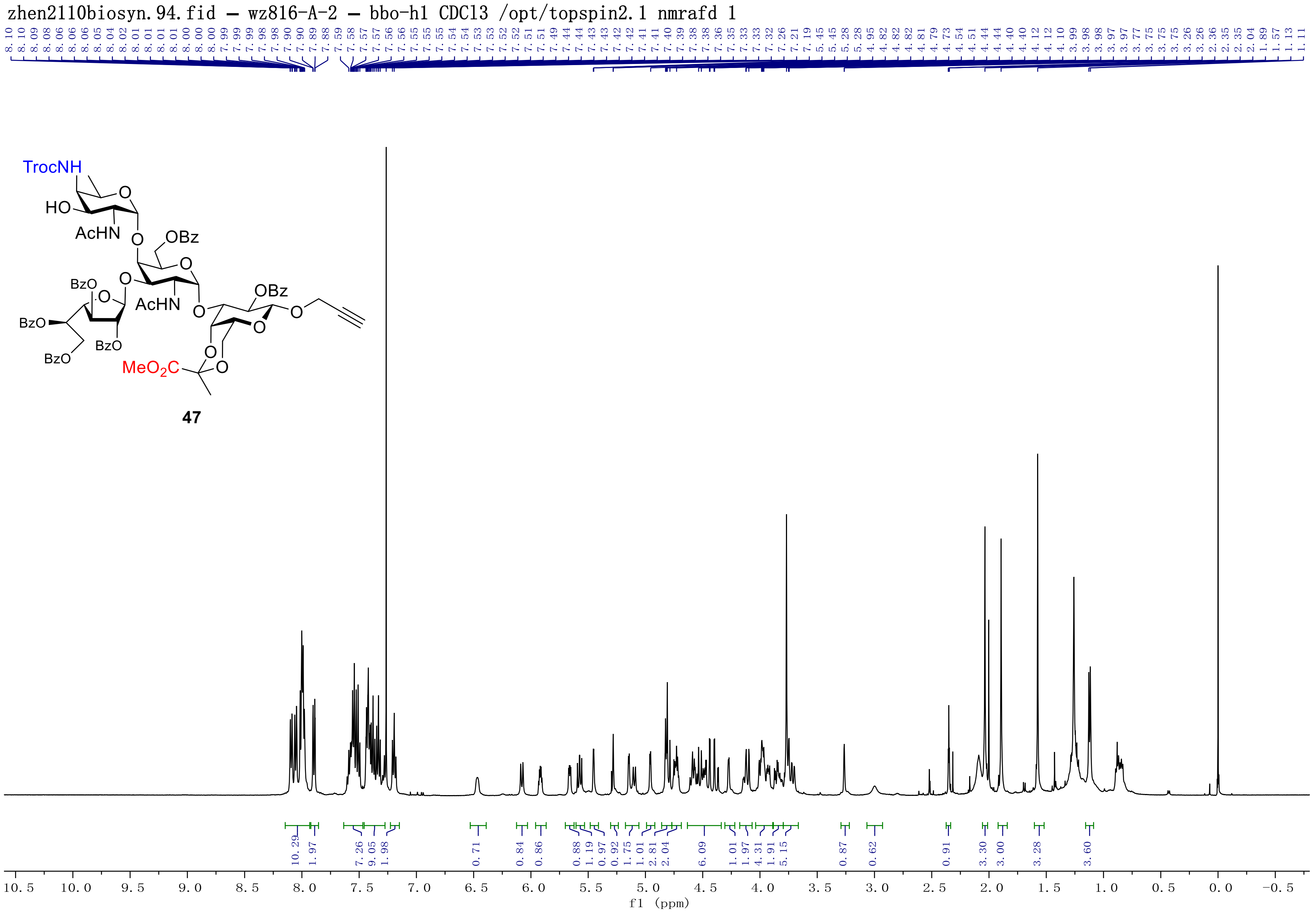

zhen2110biosyn.94.fid — wz816-A-2 — bbo-h1 CDCl3 /opt/topspin2.1 nmrafd 1
TrocNH
HO
AcHN
OBz
BzO
AcHN
OBz
BzO
BzO
BzO
MeO2C
47
f1 (ppm)

zhen2110biosyn.97.fid — wz816-A-2; — 26 mg; MW 1650; NS:1600 — bbo-c13-APT CDCl3 /opt/topspin2.1 nmrafd 1

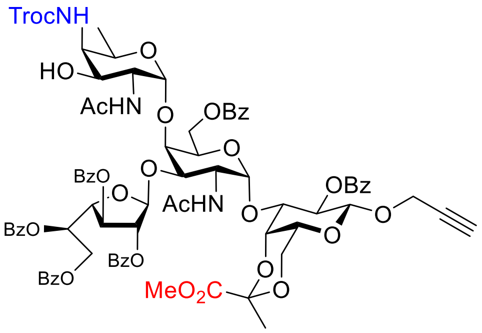

47

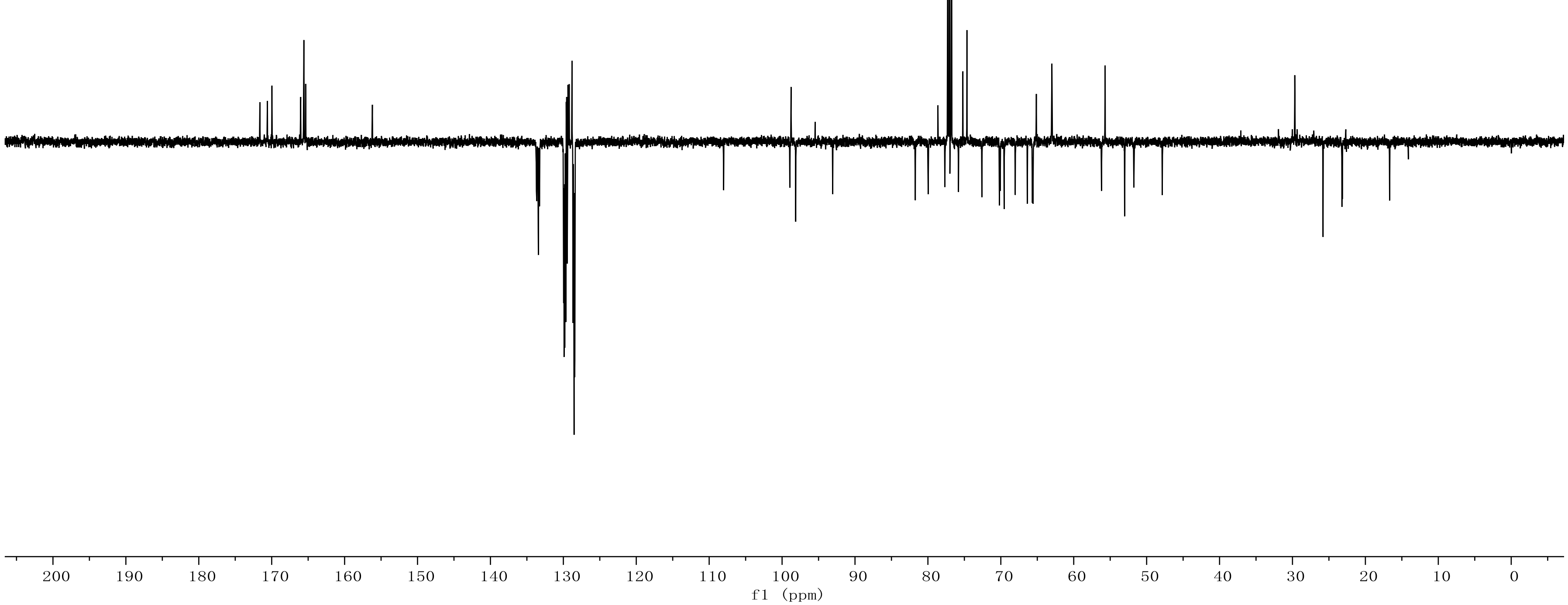

zhen2110biosyn.95.ser — wz816-A-2 — bbo-h1-cosy CDCl3 /opt/topspin2.1 nmrafd 1

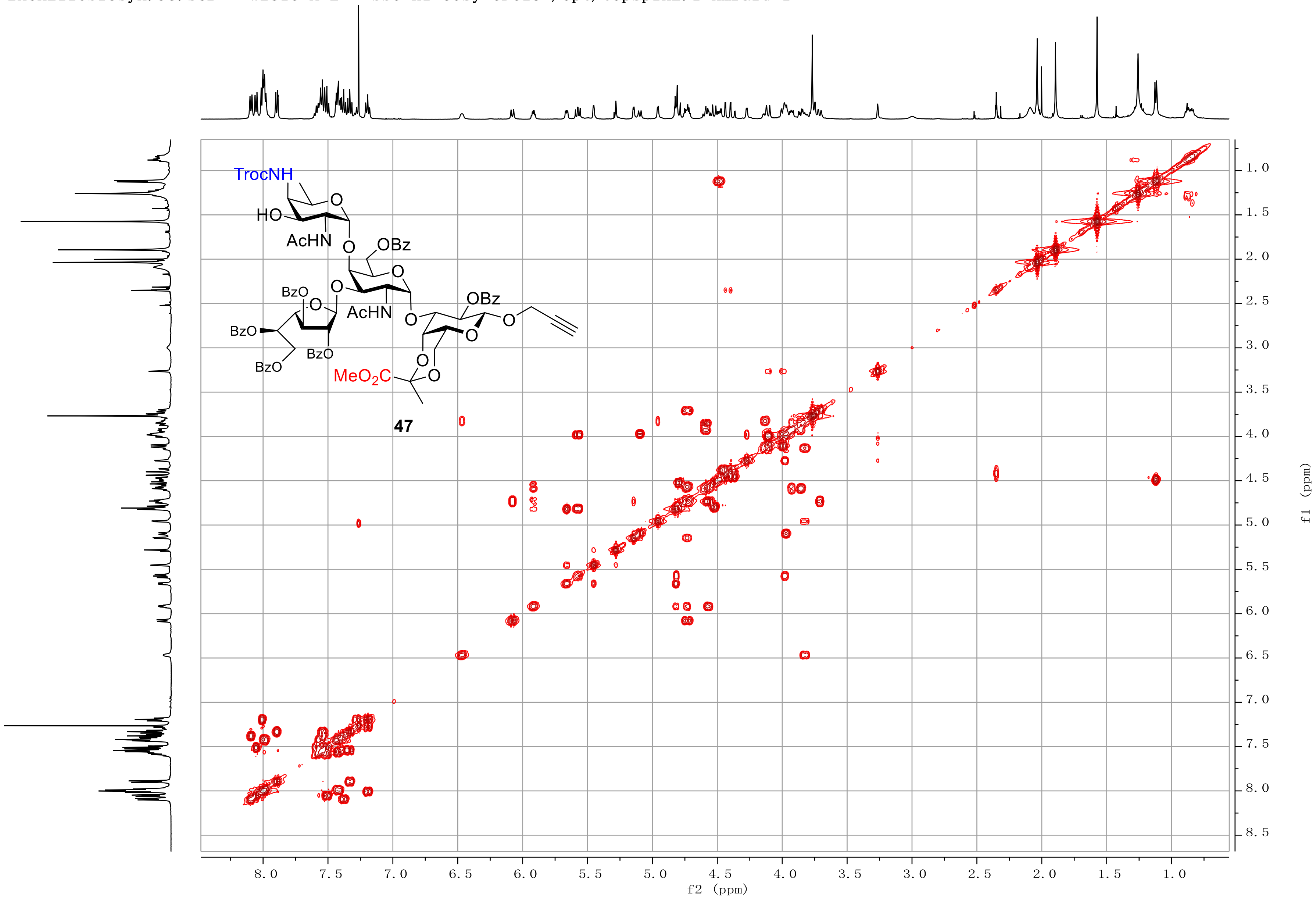

zhen2110biosyn.96.ser — wz816-A-2 — bbo-c13-HSQC CDCl3 /opt/topspin2.1 nmrafd 1

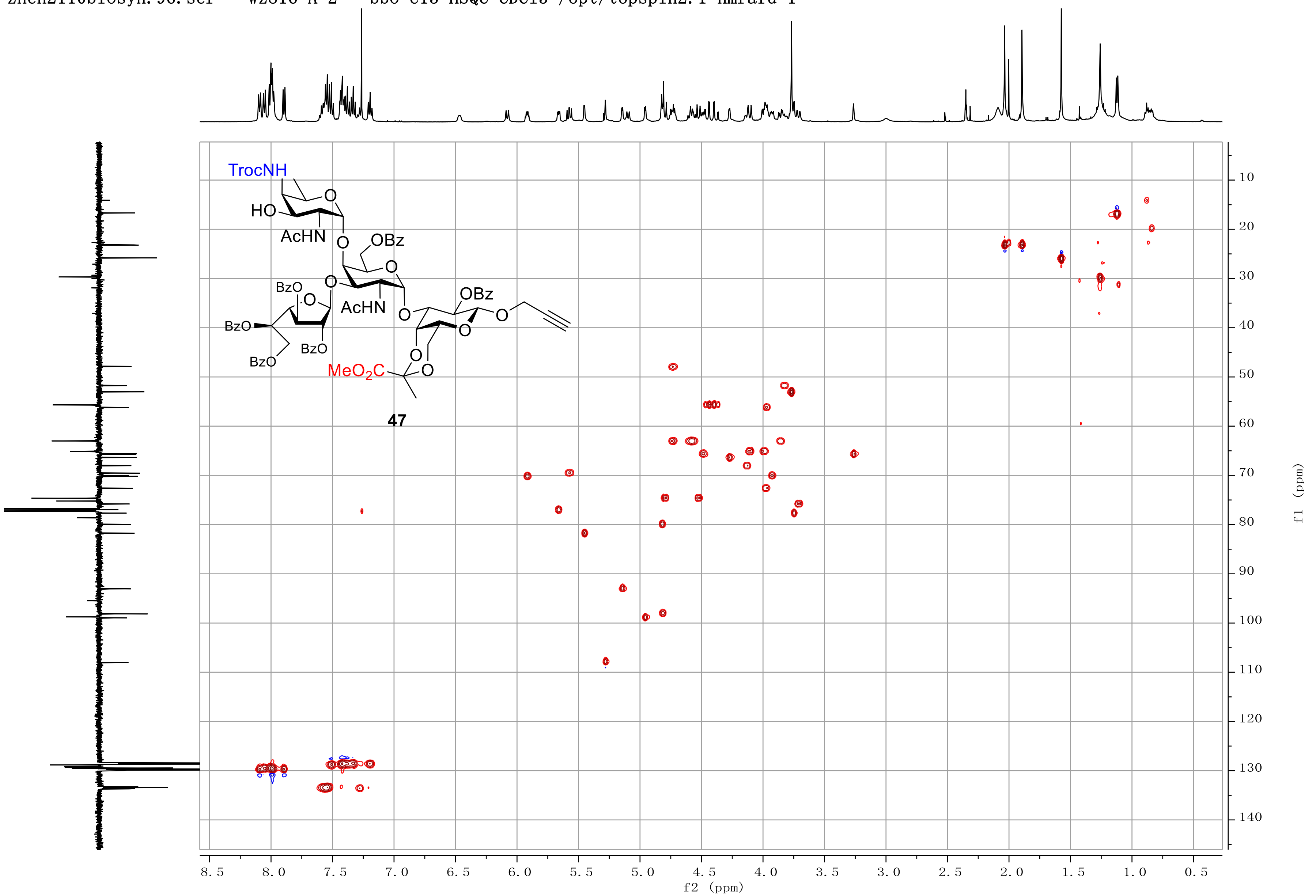

zhen2110biosyn.98.ser — wz816-A-2 — bbo-c13-HMBC CDCl3 /opt/topspin2.1 nmrafd 1

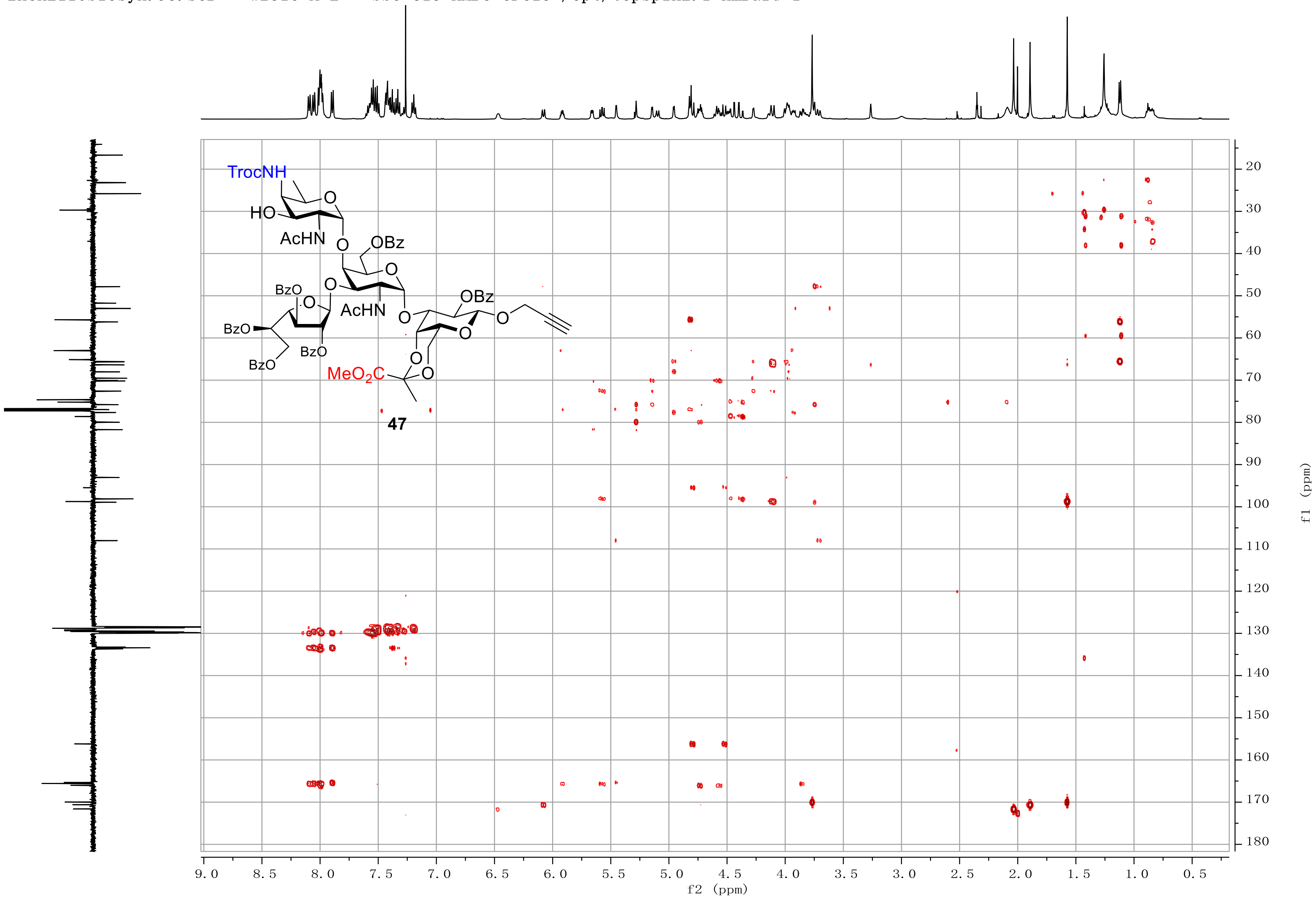

zhen2112biosyn.47.fid — wz837-D-HW40 — bbo-h1 D2O /opt/topspin2.1 nmrafd 8

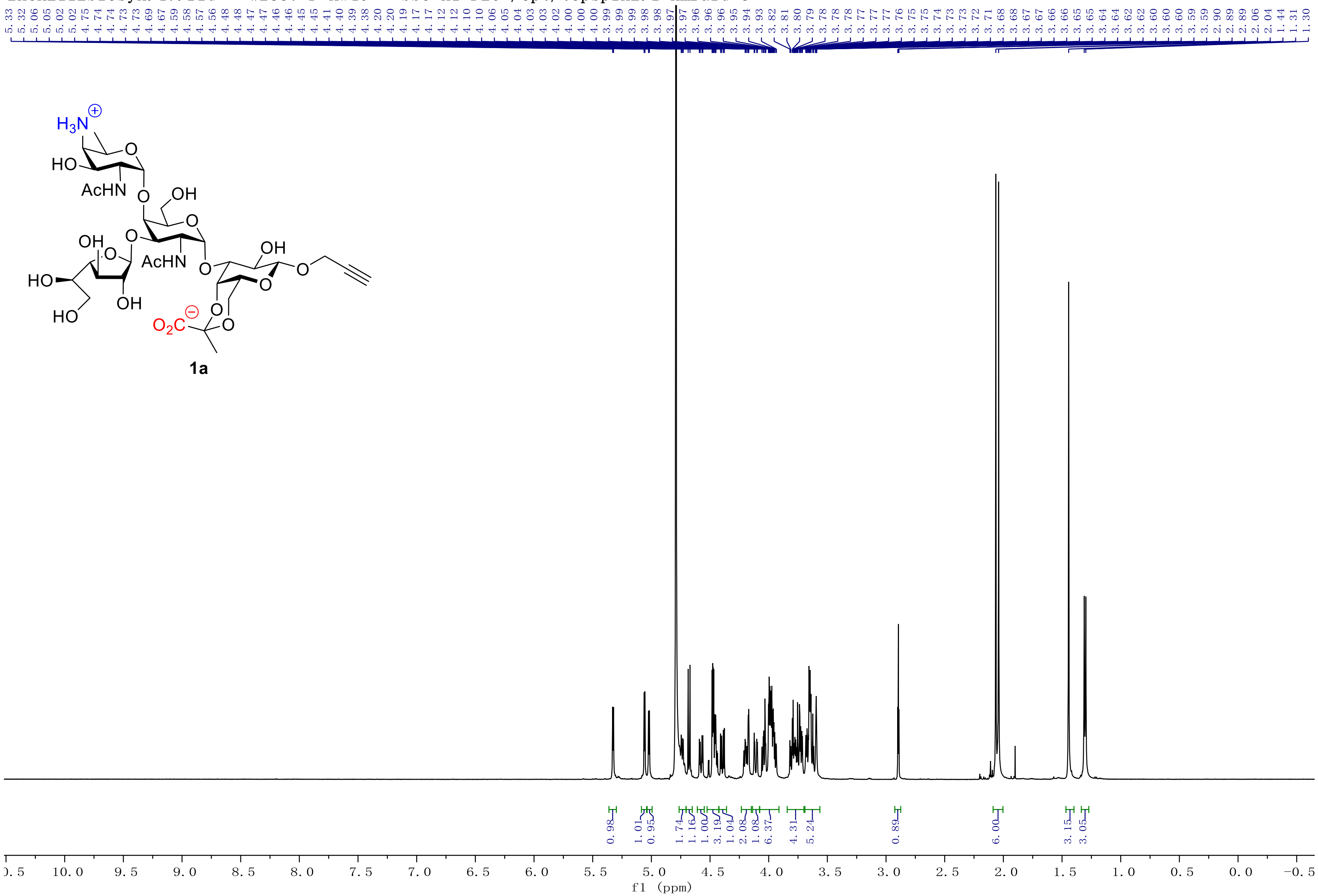

zhen2112biosyn.50.fid — wz837-D-HW40; 7 mg; MW 840 — bbo-c13-APT D2O /opt/topspin2.1 nmrafd 8

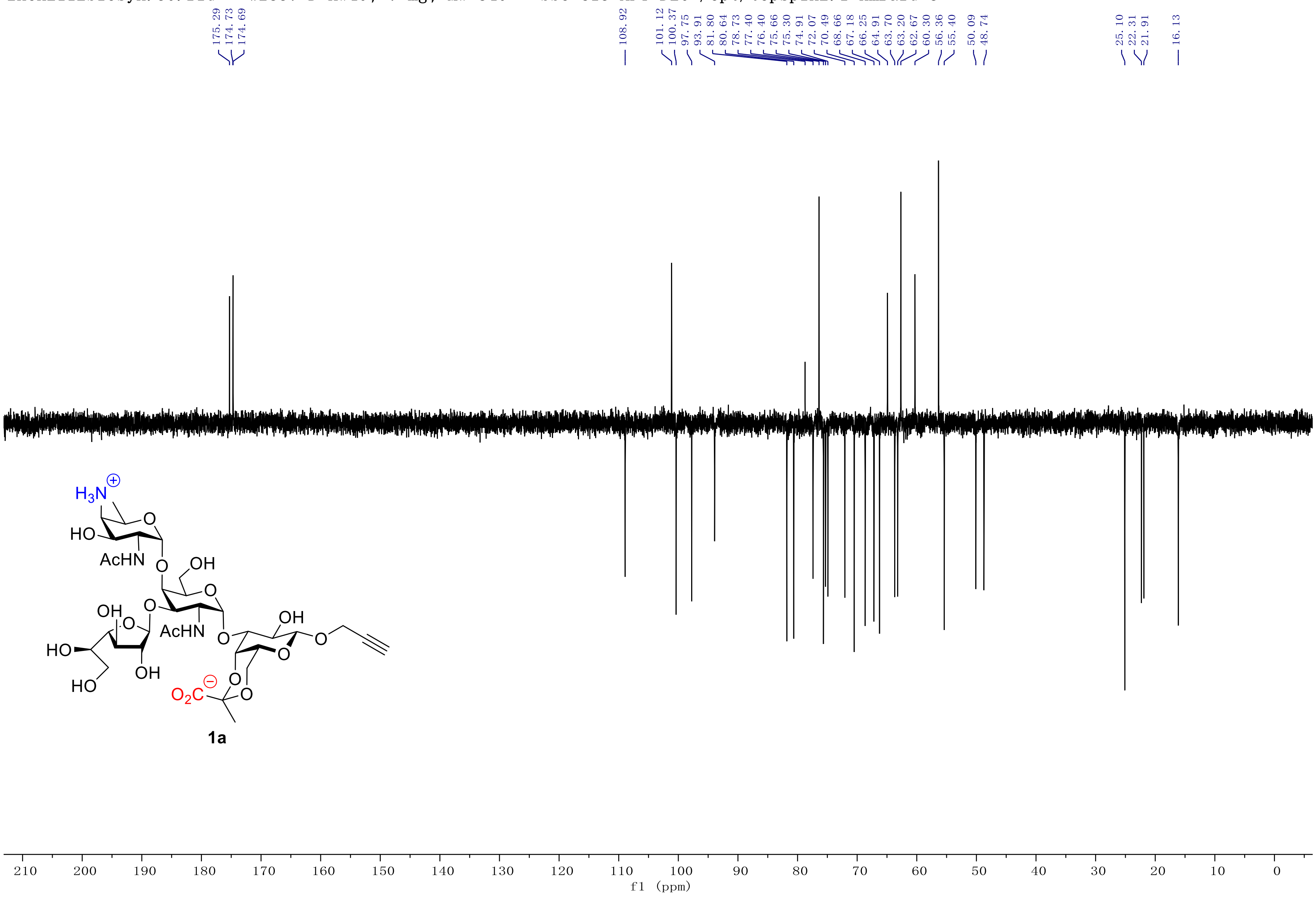

zhen2112biosyn.48.ser — wz837-D-HW40 — bbo-h1-cosy D2O /opt/topspin2.1 nmrafd 8

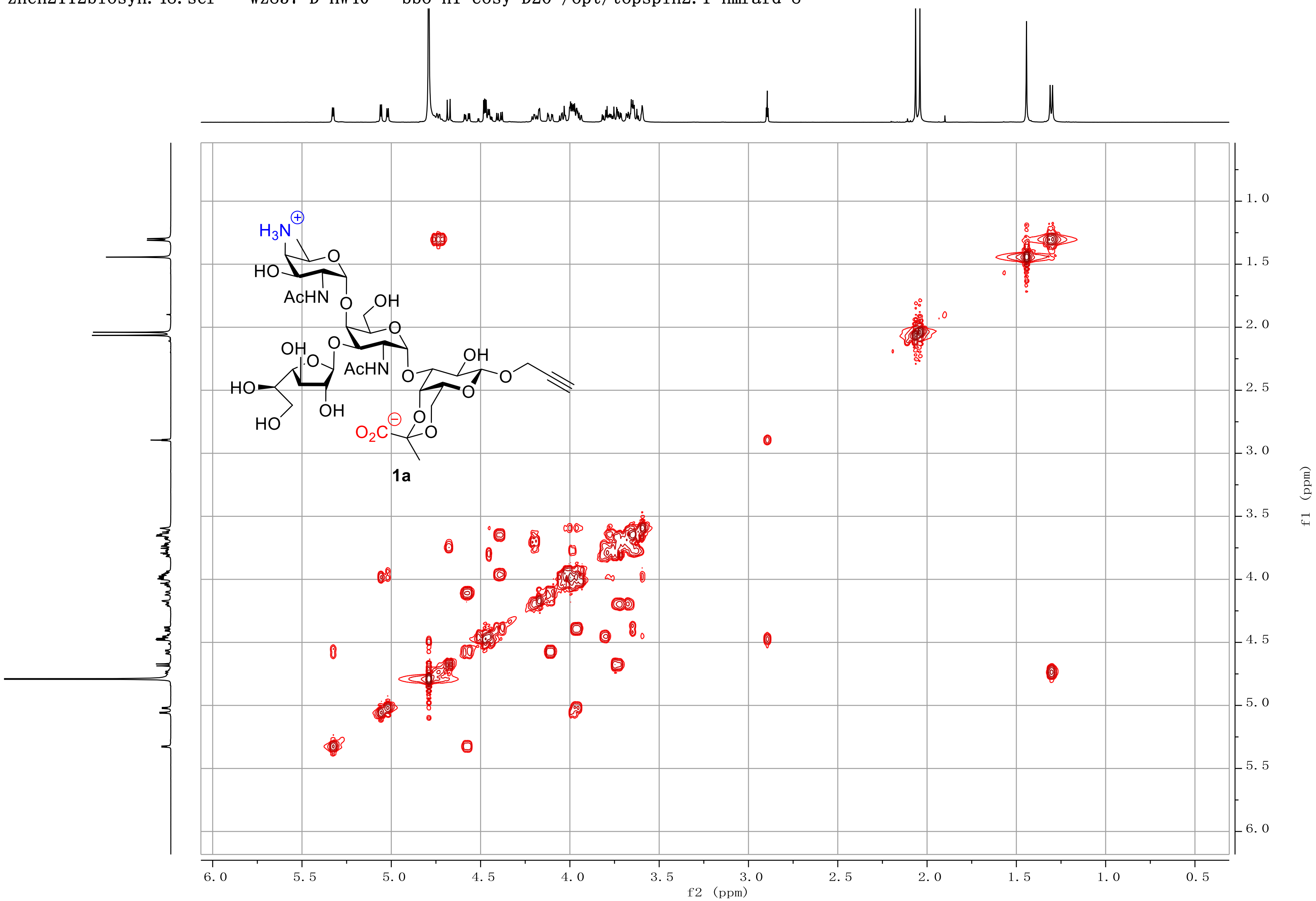

zhen2112biosyn.49.ser — wz837-D-HW40 — bbo-c13-HSQC D2O /opt/topspin2.1 nmrafd 8

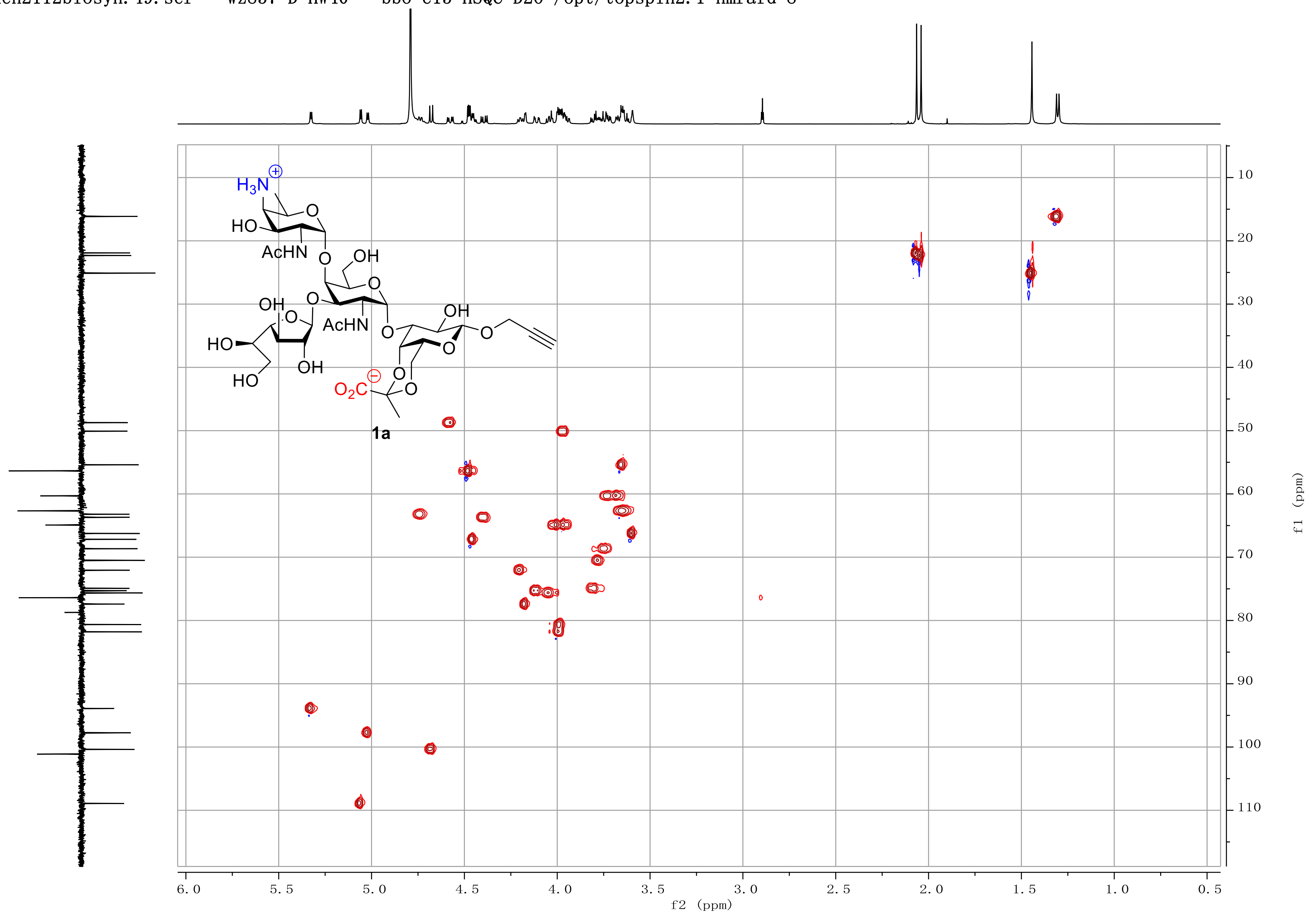

zhen2112biosyn.51.ser — wz837-D-HW40 — bbo-c13-HMBC D2O /opt/topspin2.1 nmrafd 8

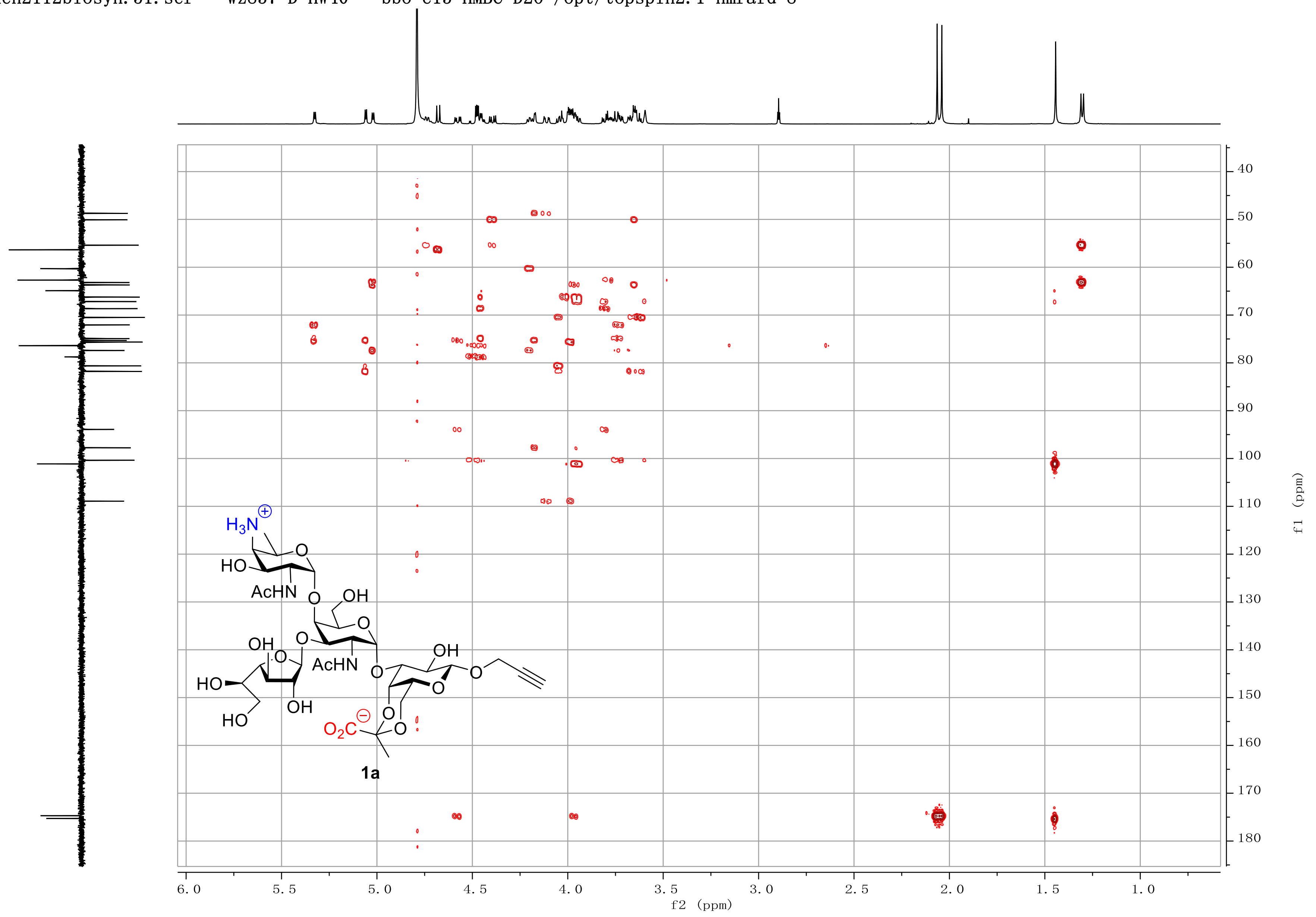

zhen2112biosyn.52.ser — wz837-D-HW40 — bbo-c13-hmbc-ipv-gated D2O /opt/topspin2.1 nmrafd 8

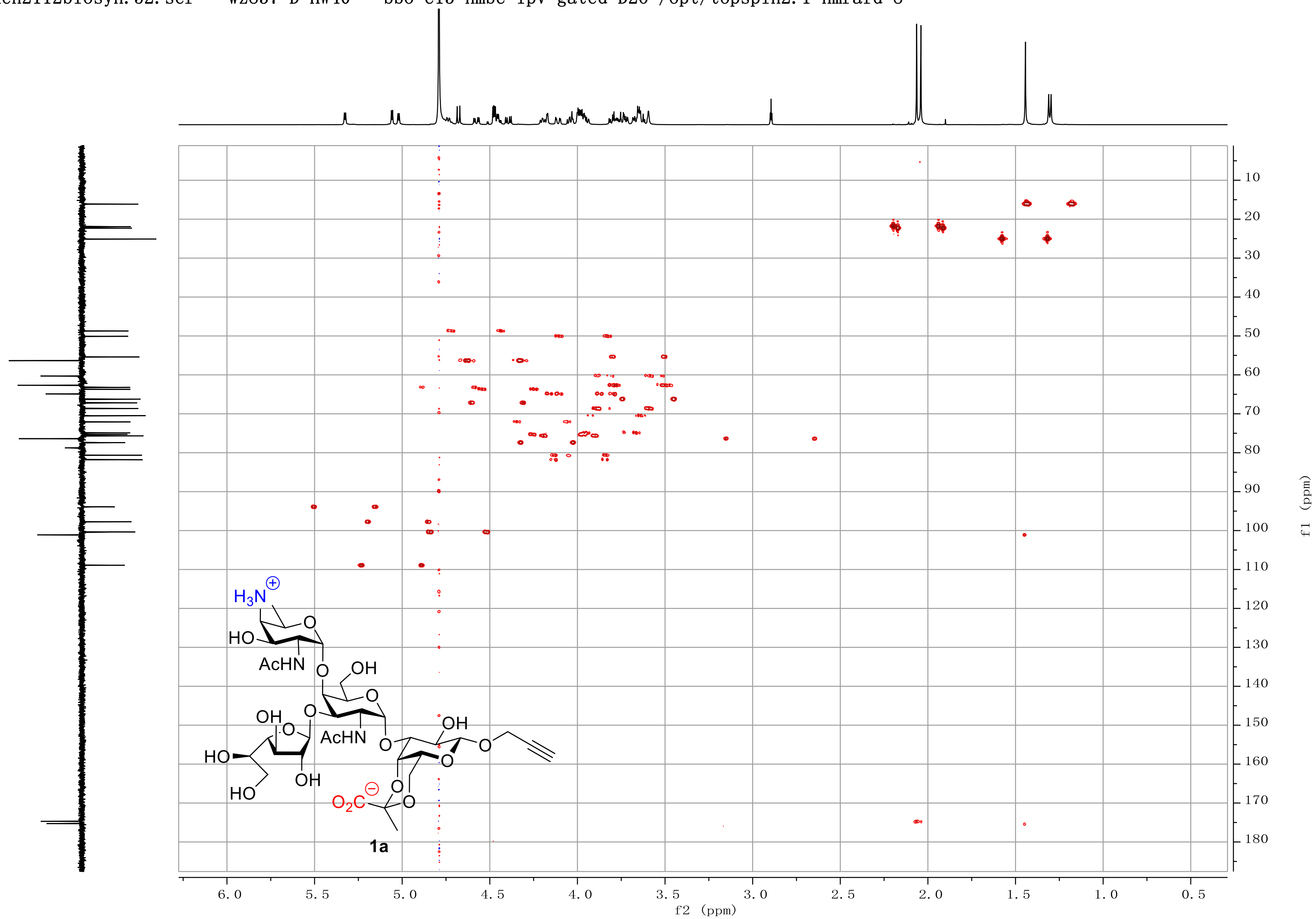

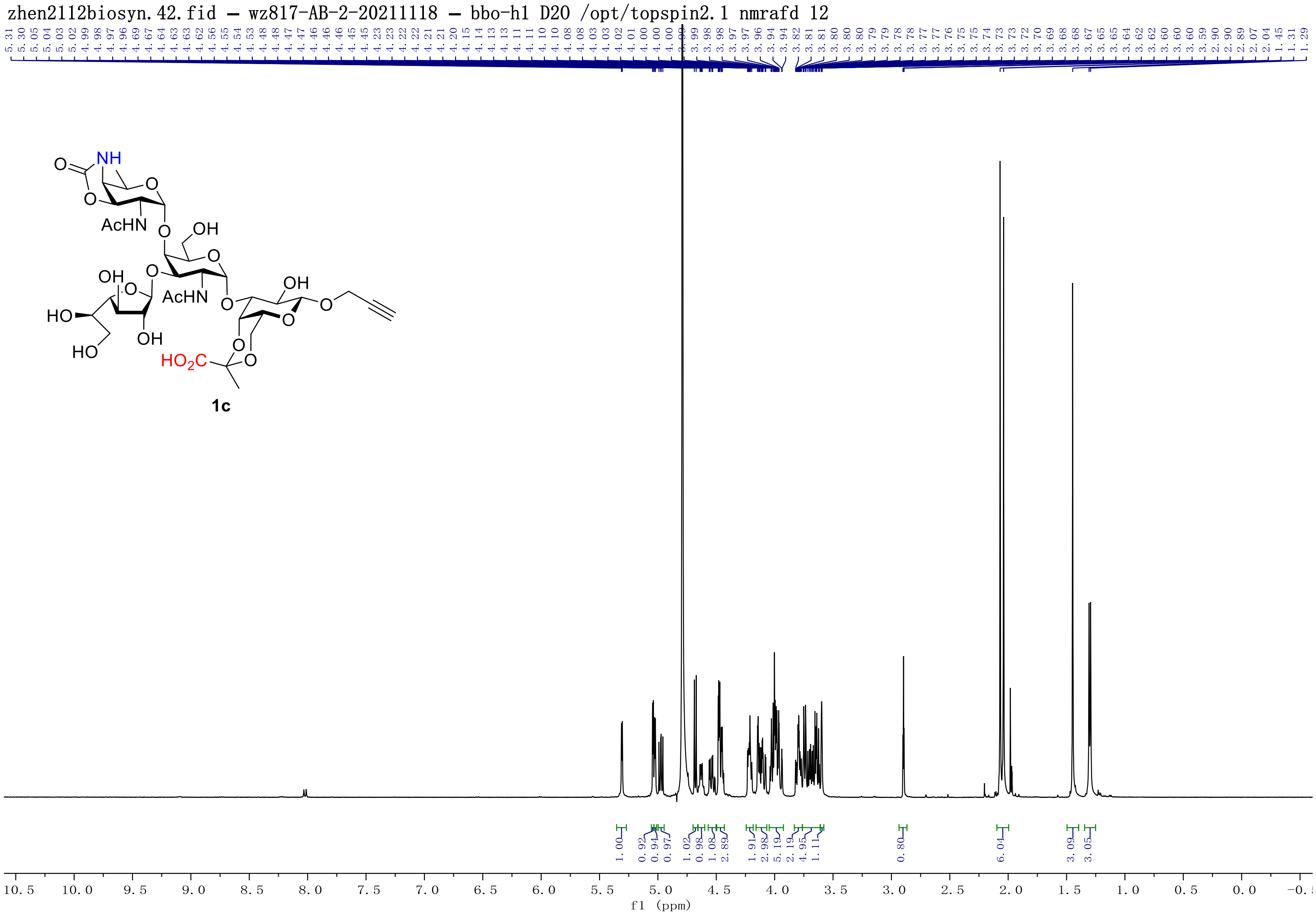
zhen2112biosyn.42.fid — wz817-AB-2-20211118 — bbo-h1 D2O /opt/topspin2.1 nmrafd 12
1c
f1 (ppm)

zhen2111biosyn.23.fid — wz817-AB-2 — bbo-c13-APT D2O /opt/topspin2.1 nmrafd 3

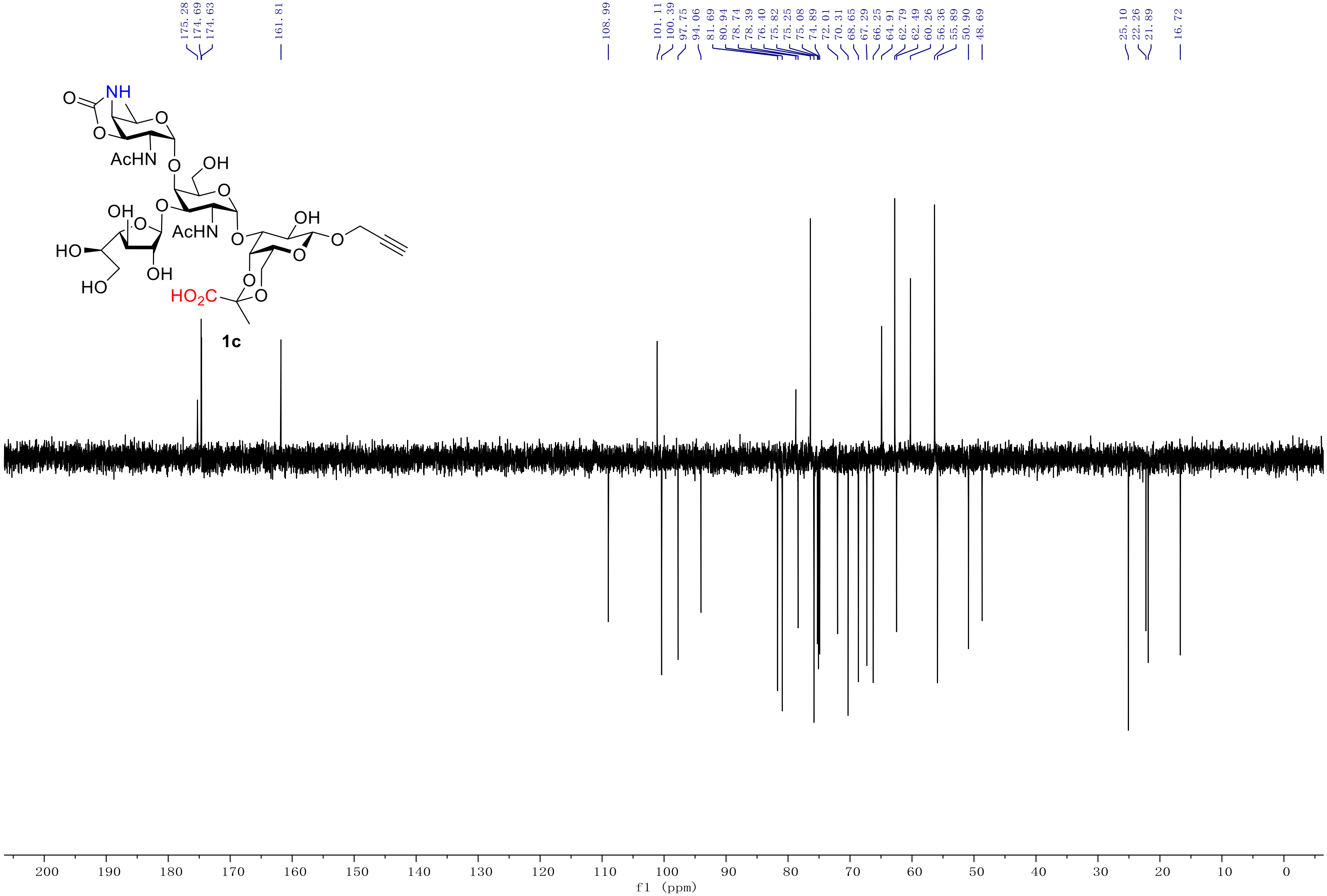

1c

zhen2111biosyn.21.ser — wz817-AB-2 — bbo-h1-cosy D2O /opt/topspin2.1 nmrafd 3

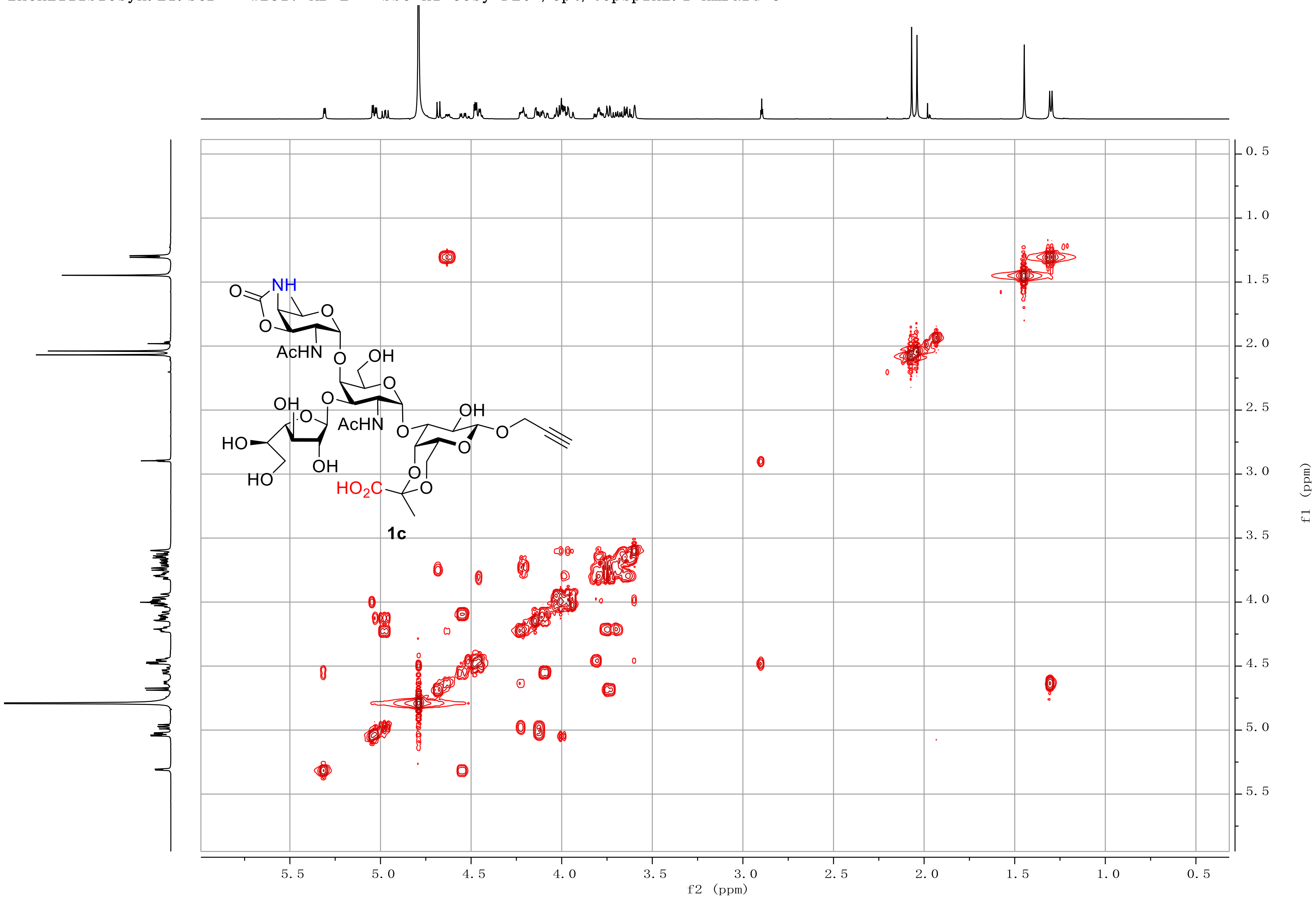

zhen2111biosyn.22.ser — wz817-AB-2 — bbo-c13-HSQC D2O /opt/topspin2.1 nmrafd 3

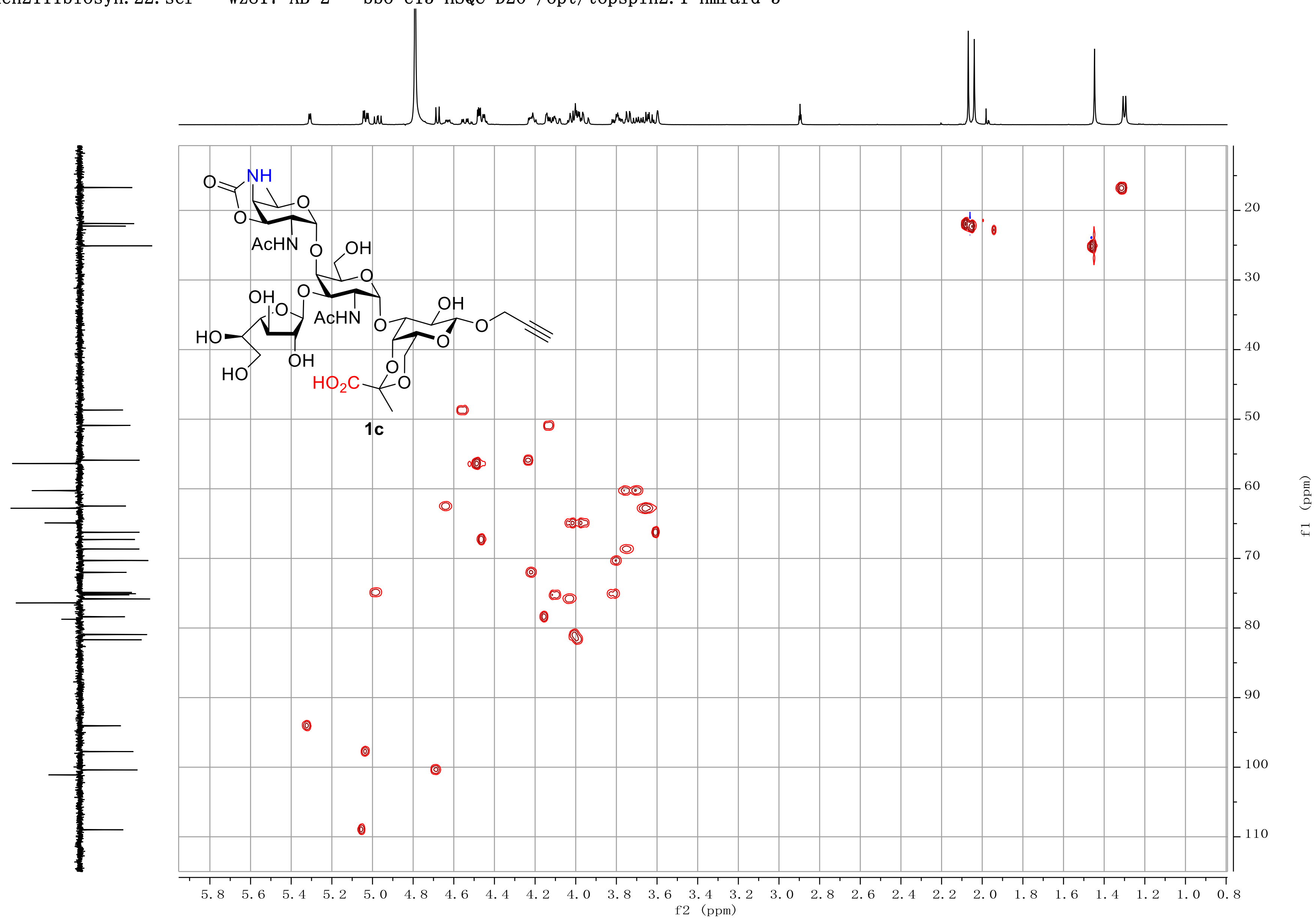

zhen2111biosyn.24.ser — wz817-AB-2 — bbo-c13-HMBC D2O /opt/topspin2.1 nmrafd 3

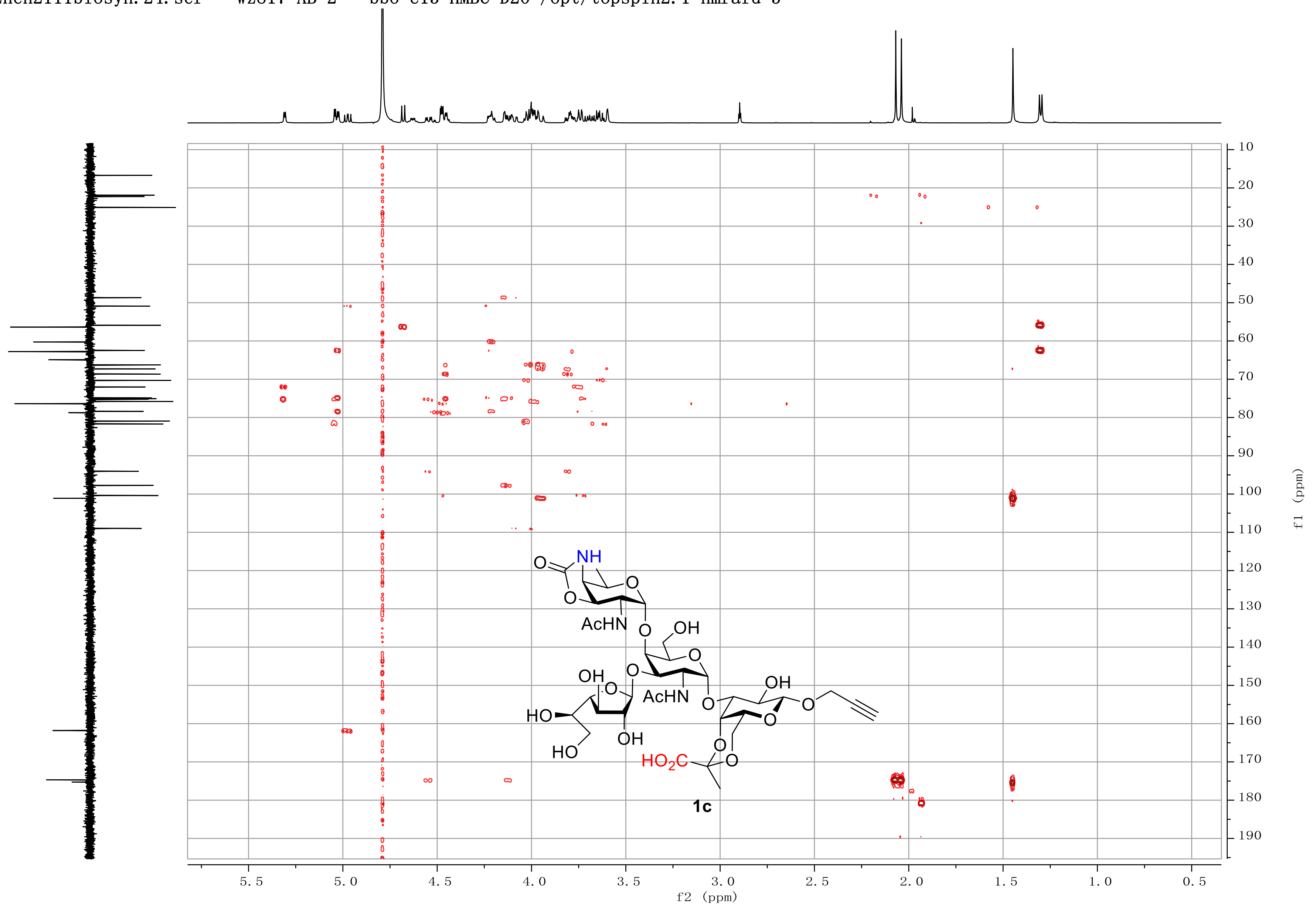

1c

zhen2110biosyn.13.fid — wz794-A-s — bbo-h1 CDCl3 /opt/topspin2.1 nmrafd 5

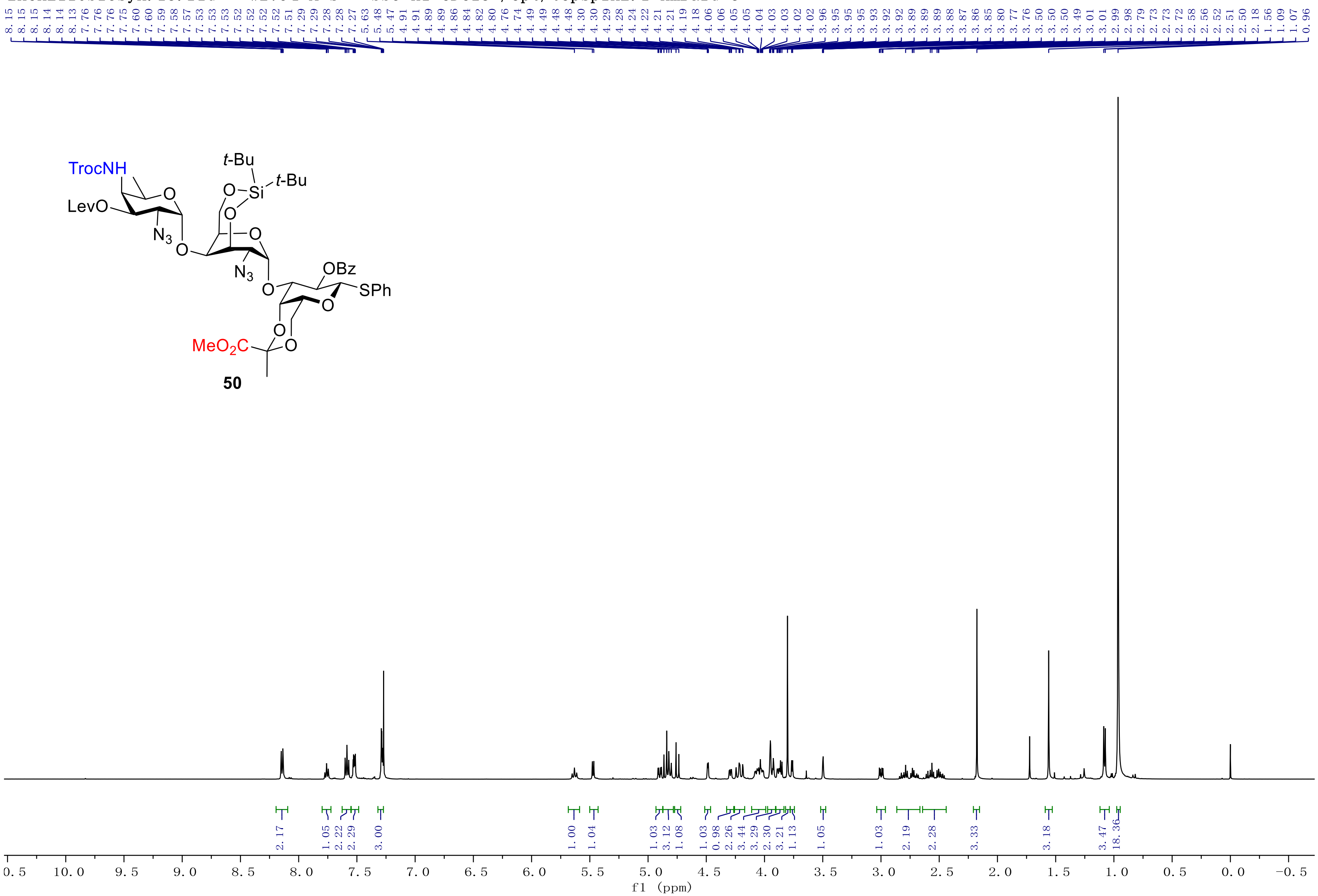

zhen2110biosyn.16.fid — wz794-A-s — bbo-c13-APT CDCl3 /opt/topspin2.1 nmrafd 5

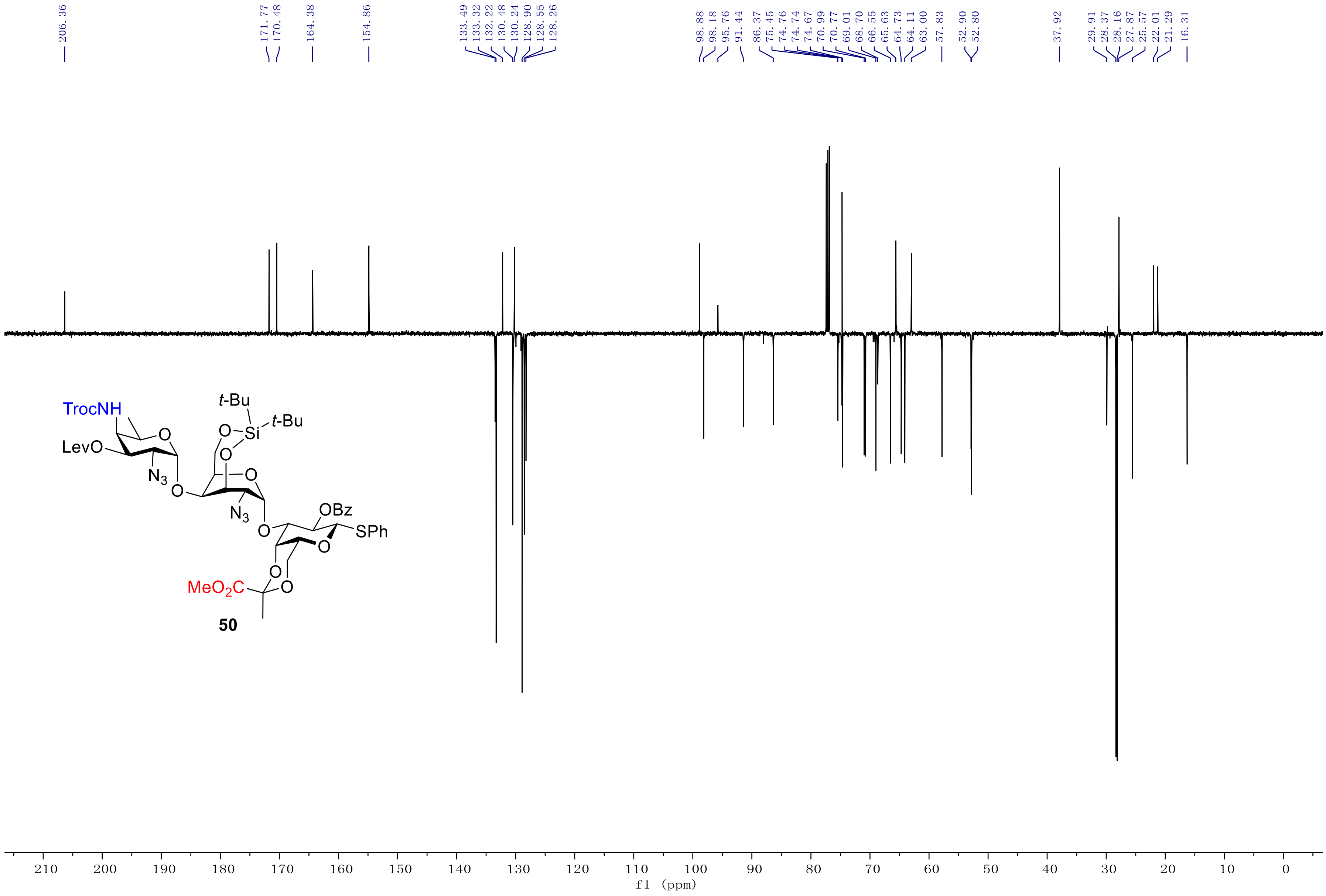

zhen2110biosyn.14.ser — wz794-A-s — bbo-h1-cosy CDCl3 /opt/topspin2.1 nmrafd 5

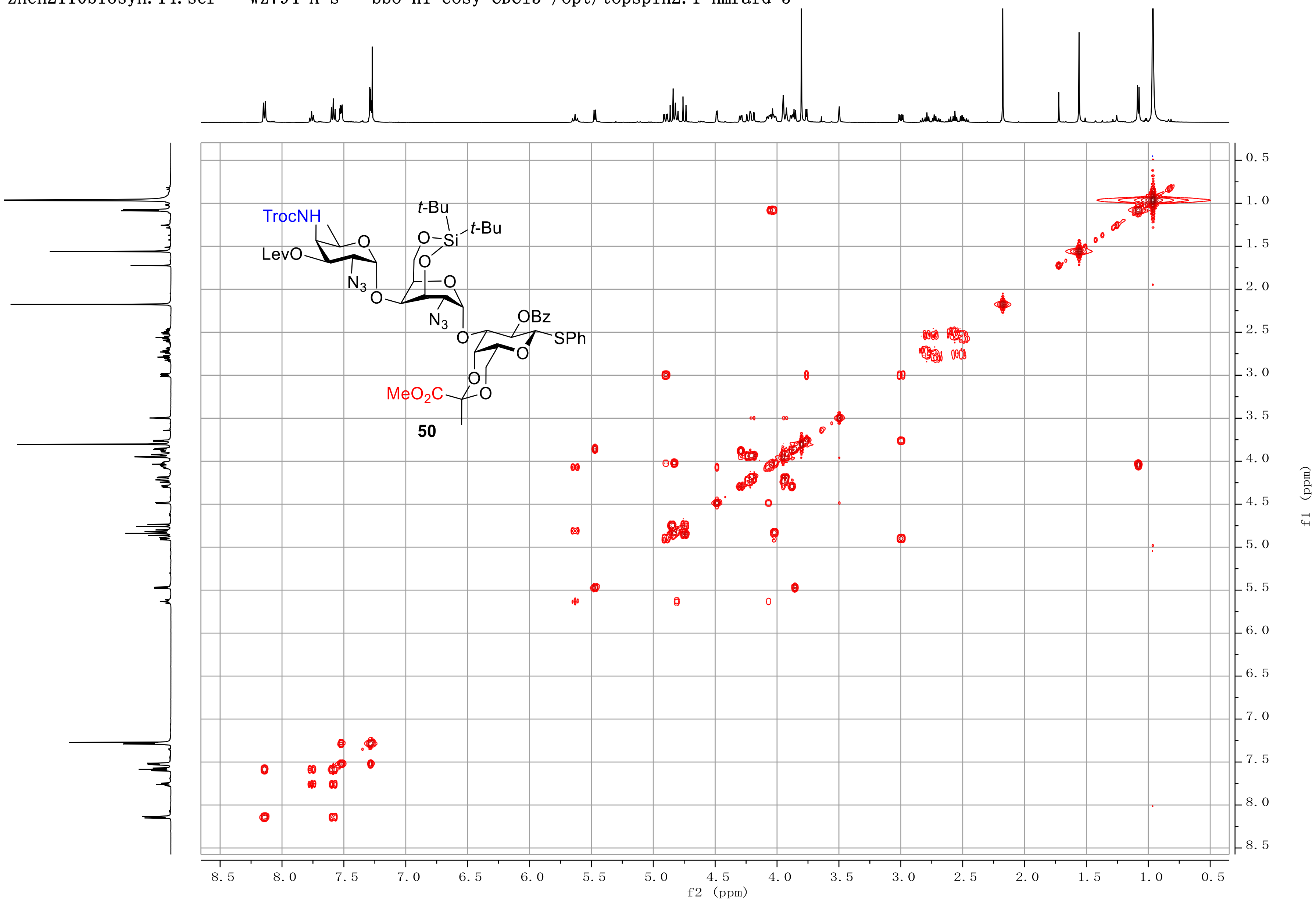

zhen2110biosyn.15.ser — wz794-A-s — bbo-c13-HSQC CDCl3 /opt/topspin2.1 nmrafd 5

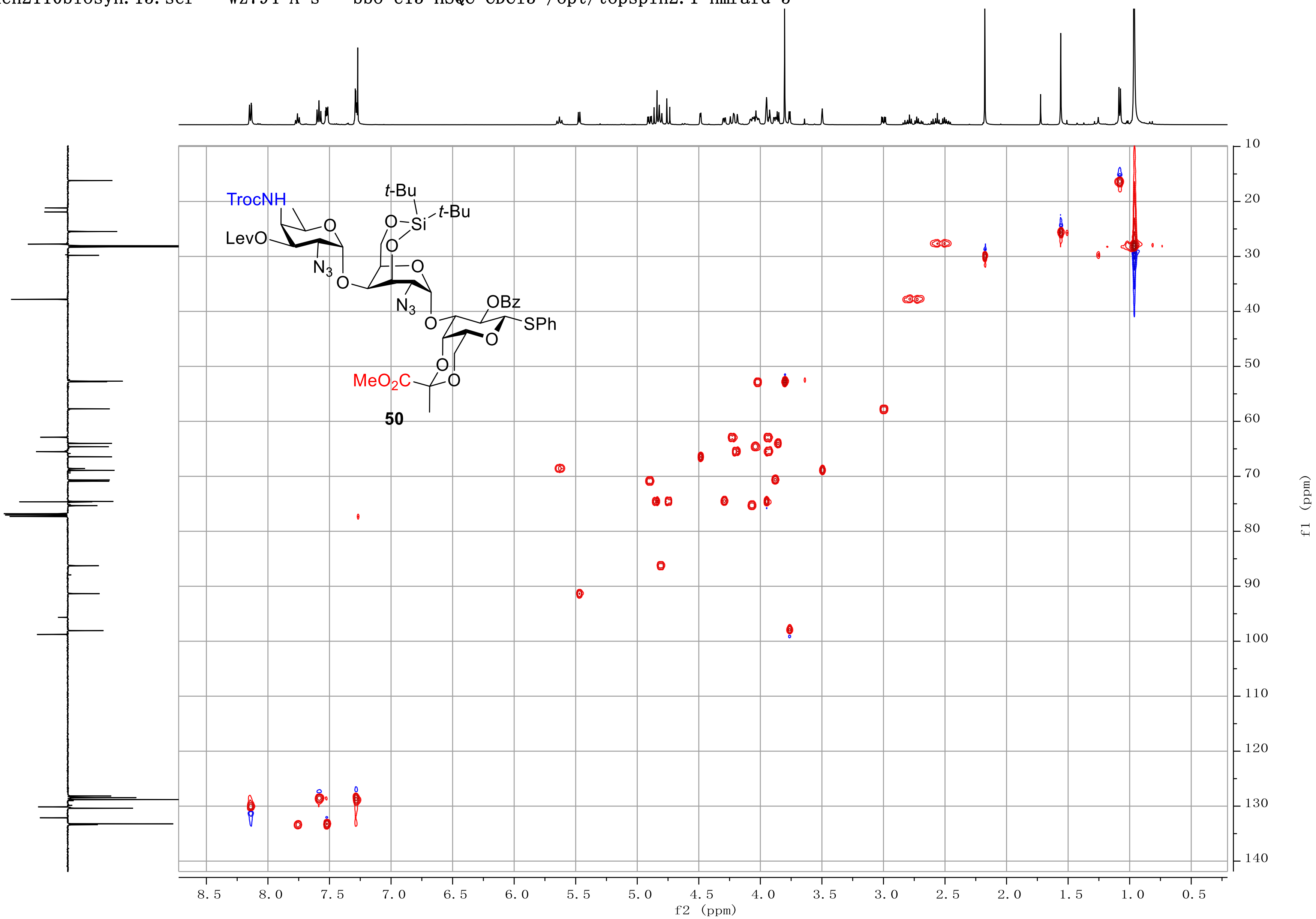

zhen2110biosyn.17.ser — wz794-A-s — bbo-c13-HMBC CDCl3 /opt/topspin2.1 nmrafd 5

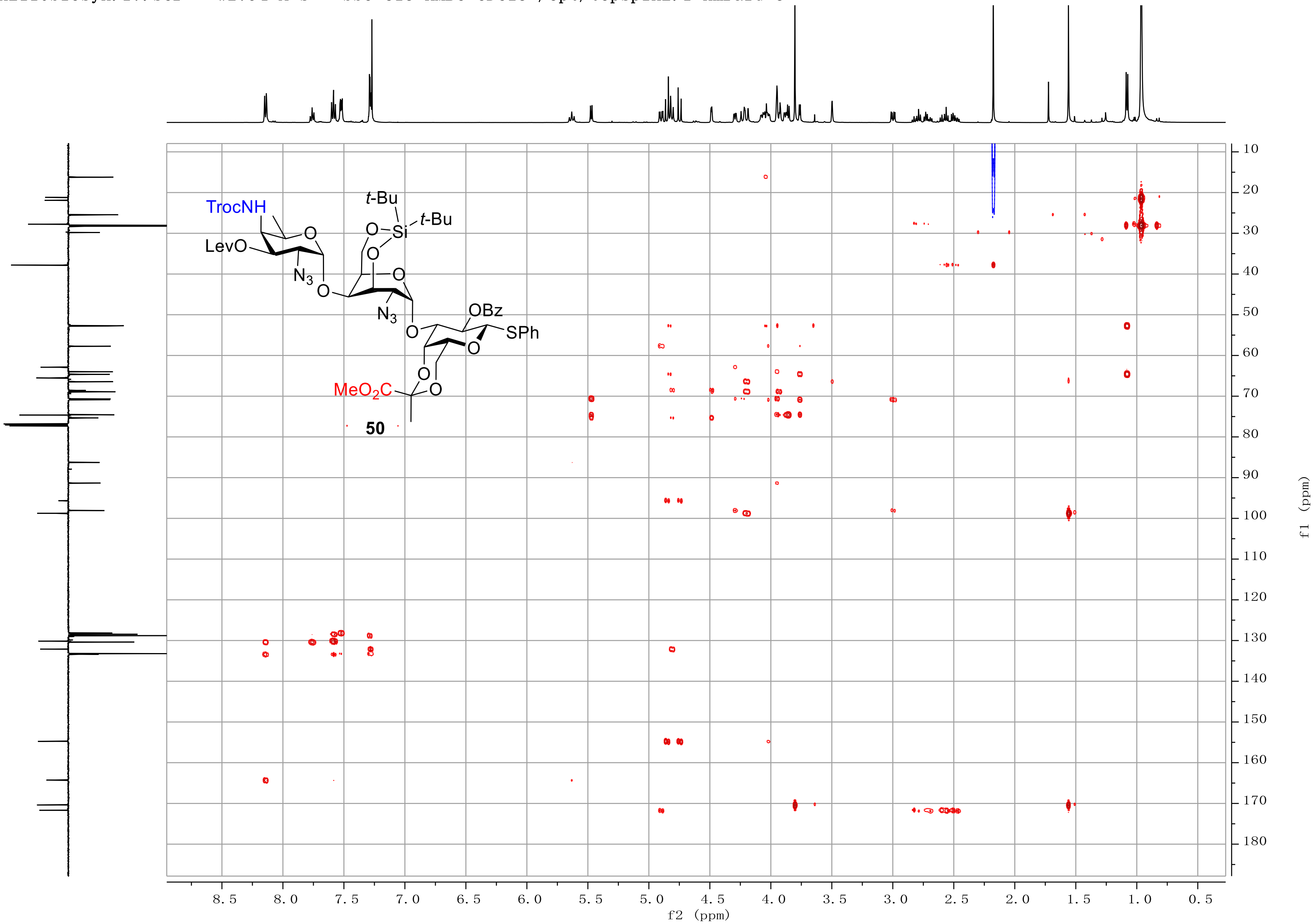

zhen2110biosyn.18.ser — wz794-A-s — bbo-c13-hmbc-ipv-gated CDCl3 /opt/topspin2.1 nmrafd 5

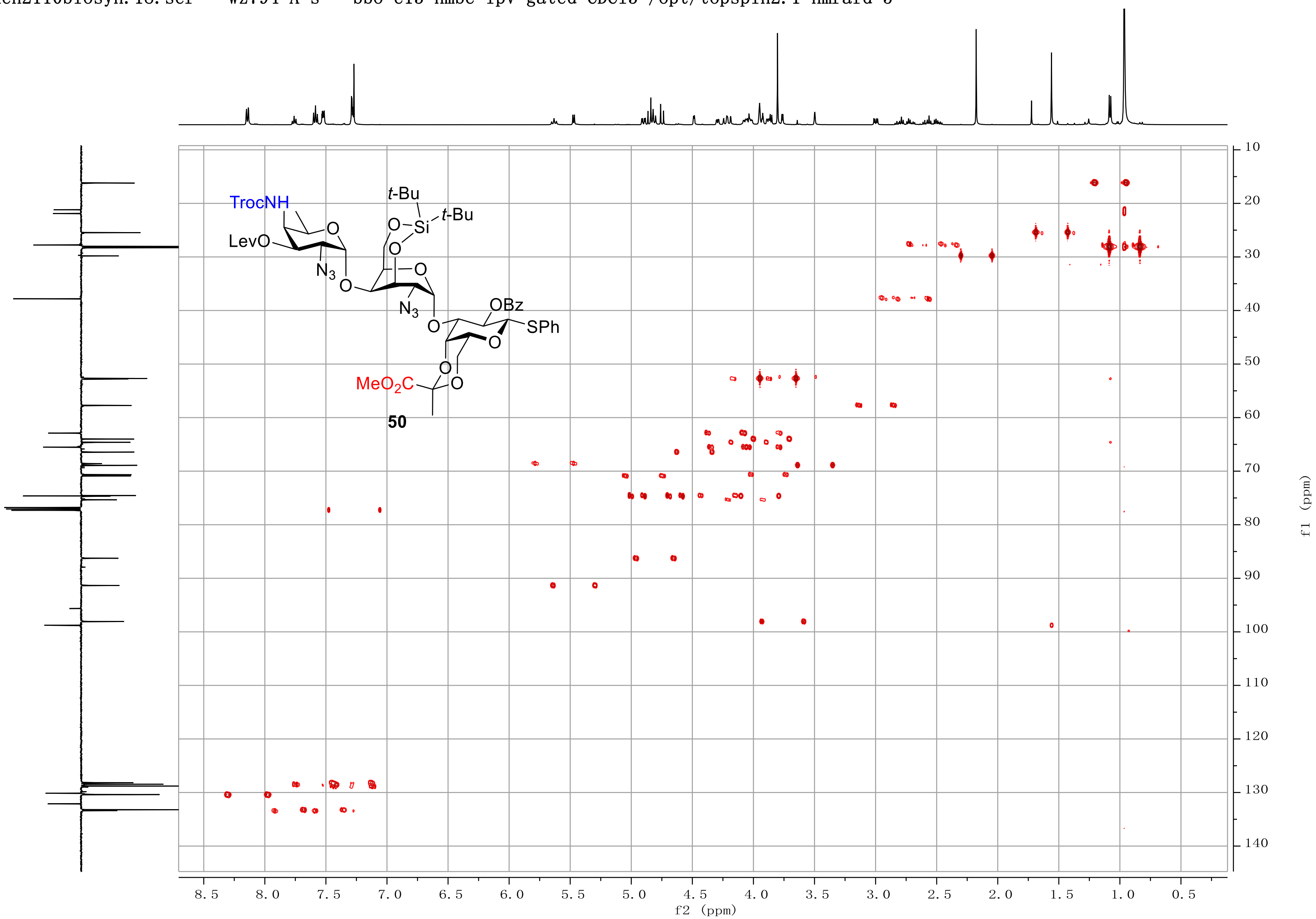

zhen2110biosyn.43.fid — wz795-C-s — bbo-h1 CDCl3 /opt/topspin2.1 nmrafd 15

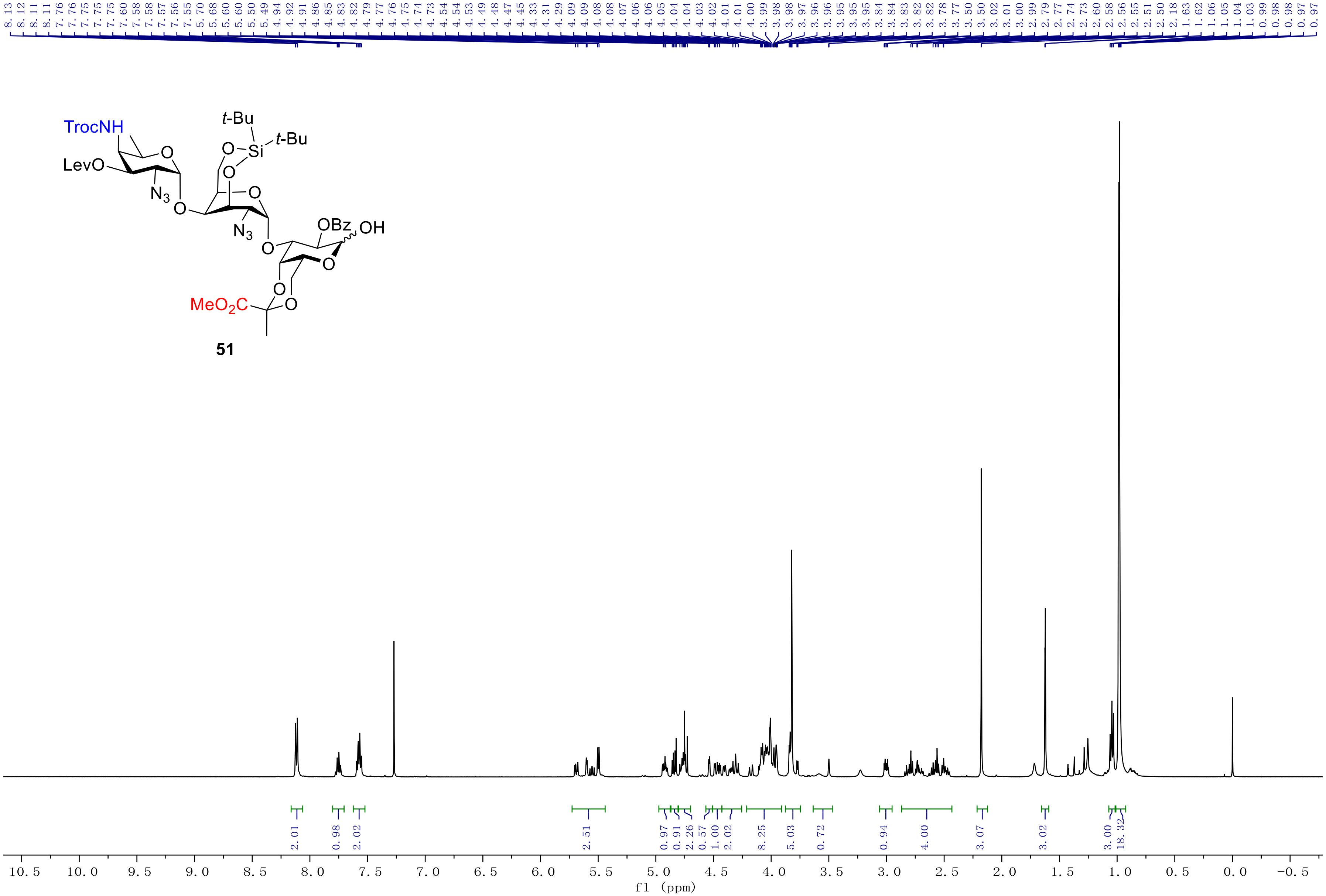

zhen2110biosyn.46.fid — wz795-C-s — bbo-c13-APT CDCl3 /opt/topspin2.1 nmrafd 15

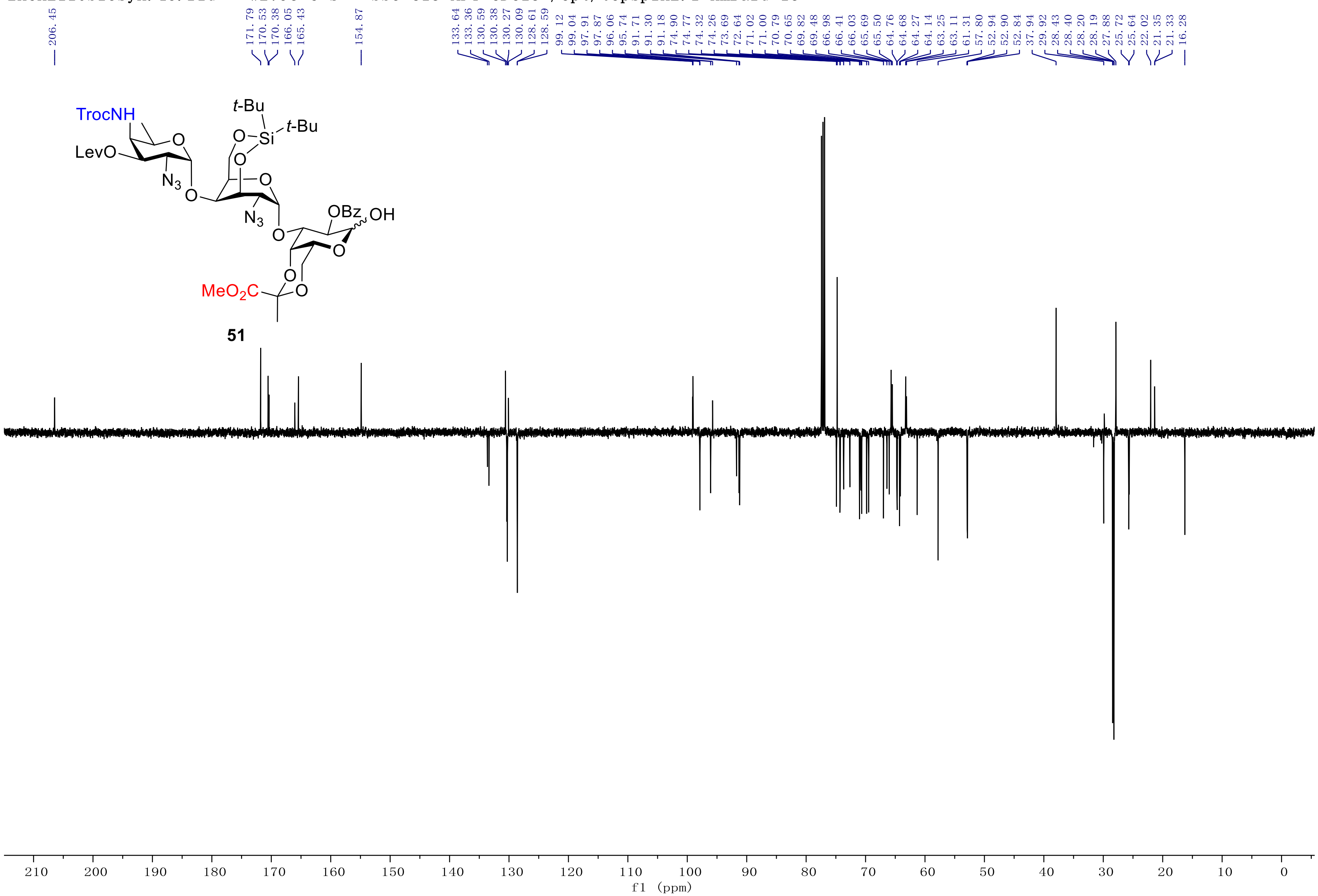

zhen2110biosyn.44.ser — wz795-C-s — bbo-h1-cosy CDCl3 /opt/topspin2.1 nmrafd 15

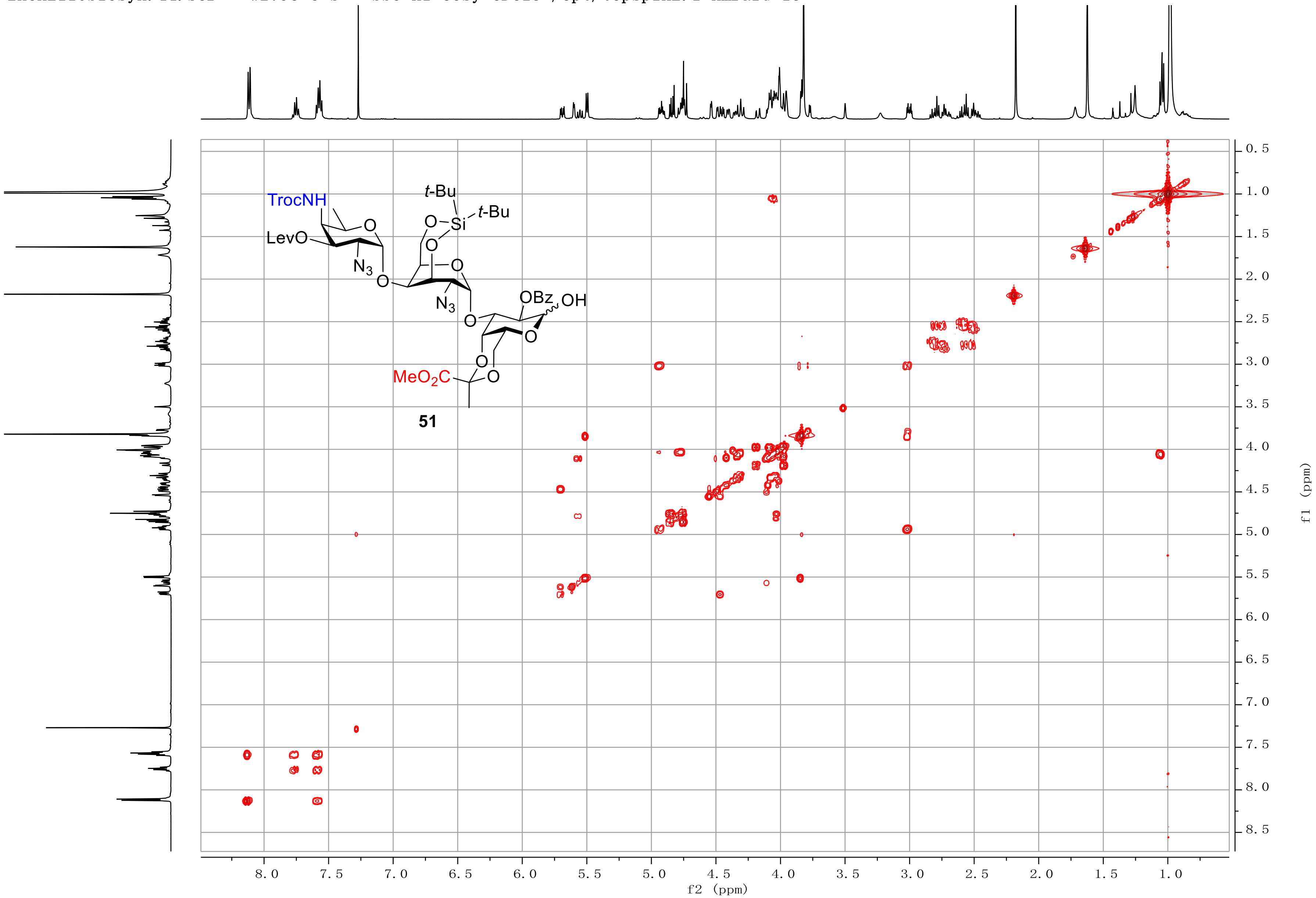

zhen2110biosyn.45.ser — wz795-C-s — bbo-c13-HSQC CDCl3 /opt/topspin2.1 nmrafd 15

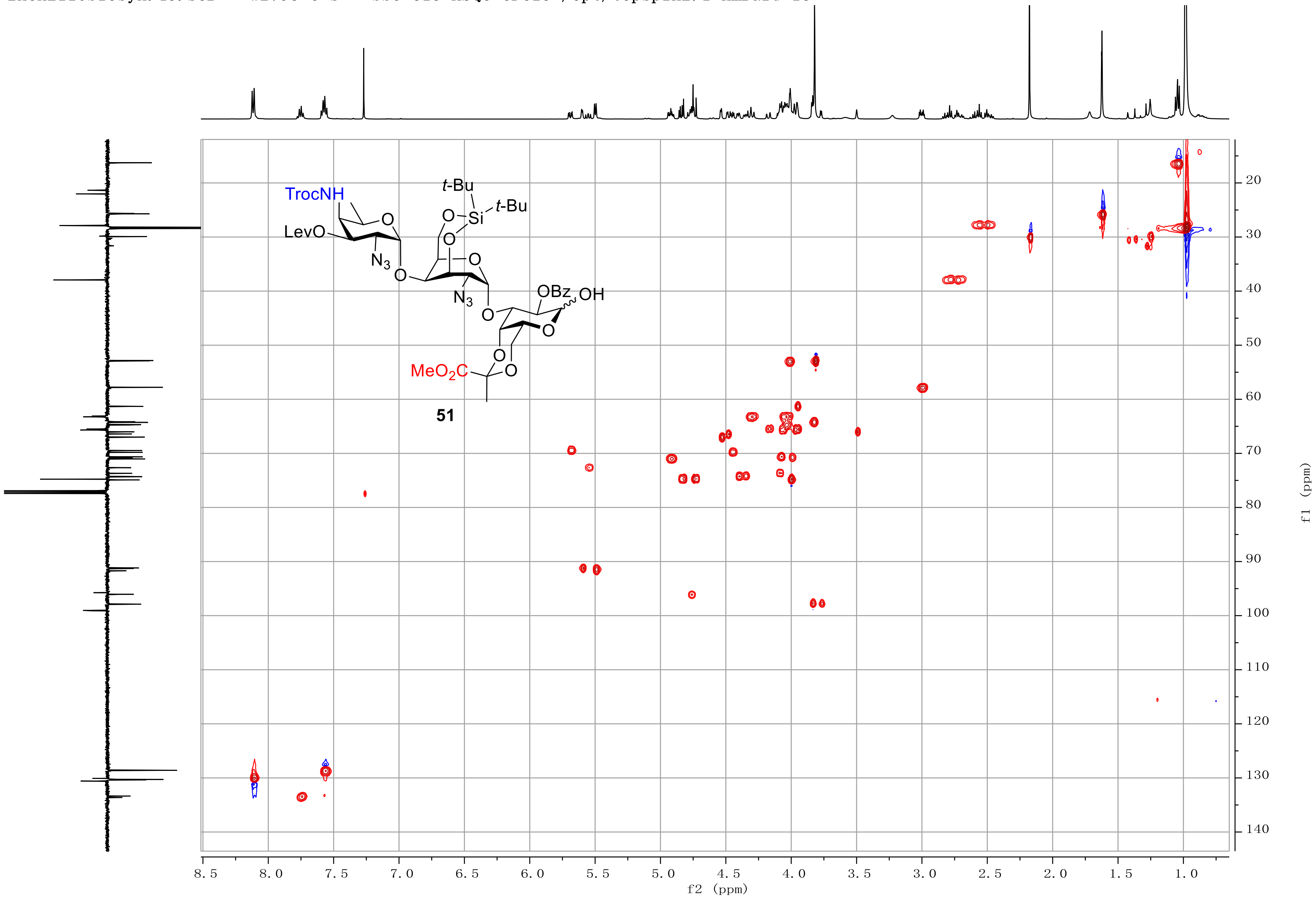

zhen2110biosyn.48.fid — wz796-B-s — bbo-h1 Acetone /opt/topspin2.1 nmrafd 8

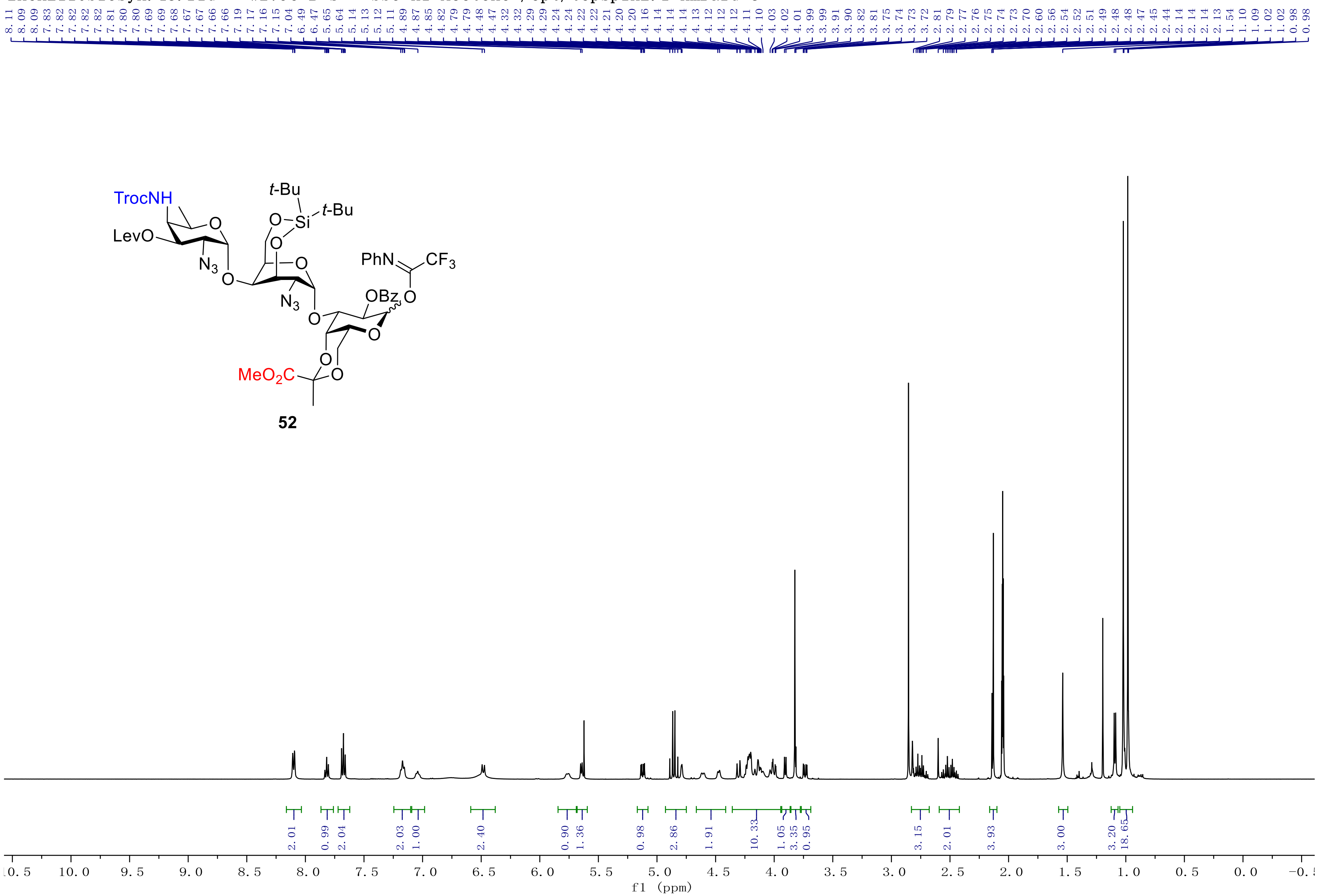

52

zhen2110biosyn.51.fid — wz796-B-s — bbo-c13-APT Acetone /opt/topspin2.1 nmrafd 8

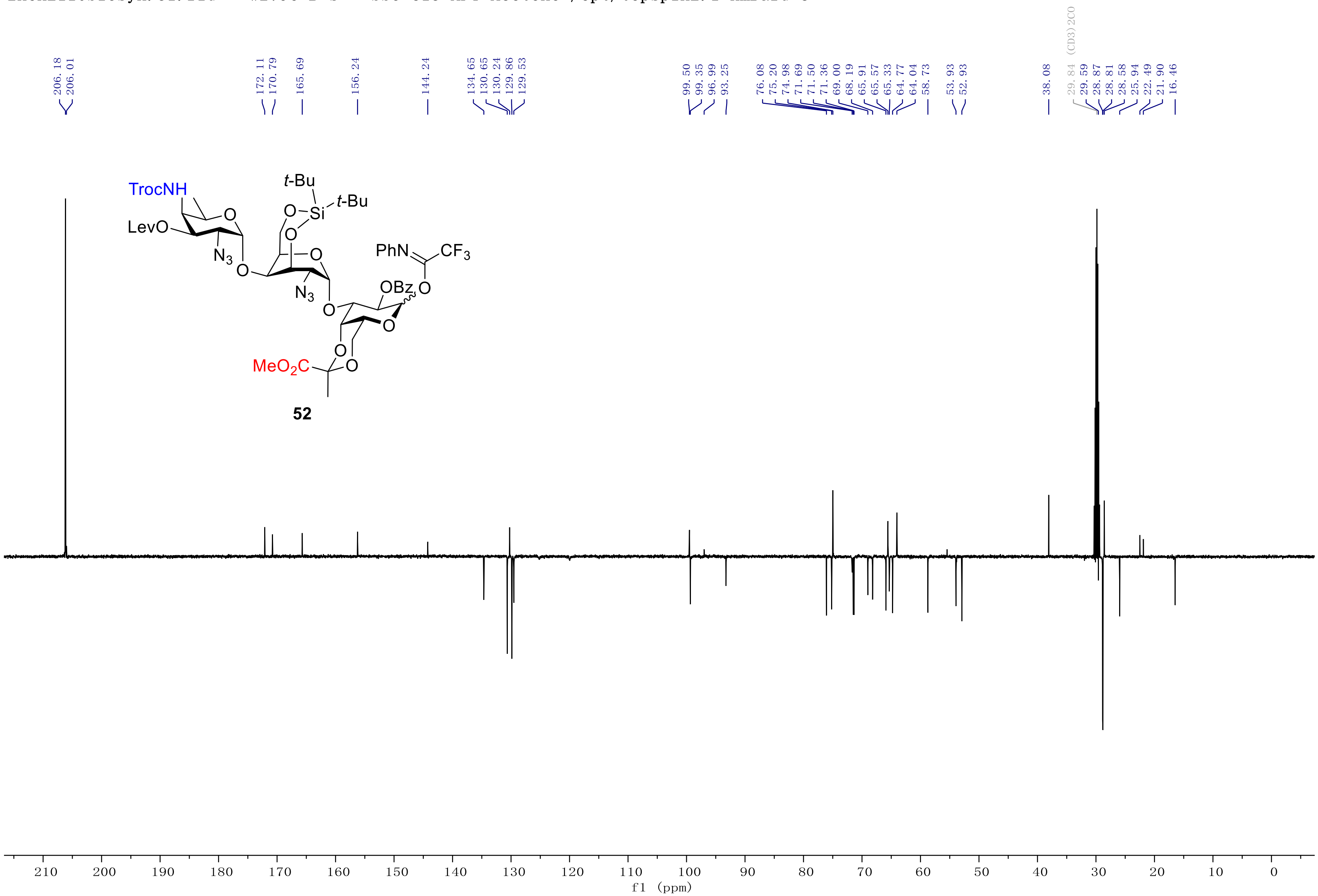

zhen2110biosyn.49.ser — wz796-B-s — bbo-h1-cosy Acetone /opt/topspin2.1 nmrafd 8

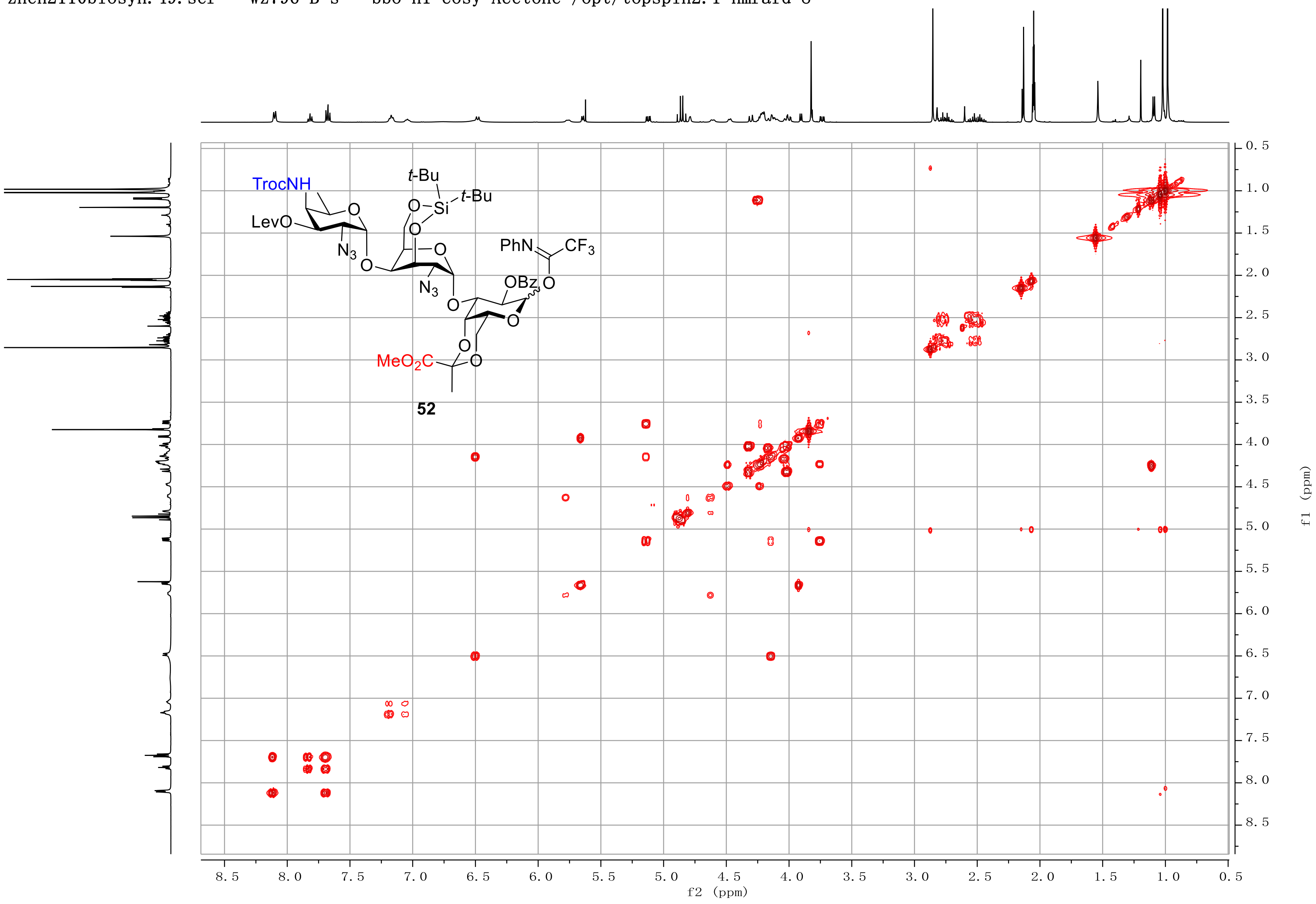

zhen2110biosyn.50.ser — wz796-B-s — bbo-c13-HSQC Acetone /opt/topspin2.1 nmrafd 8

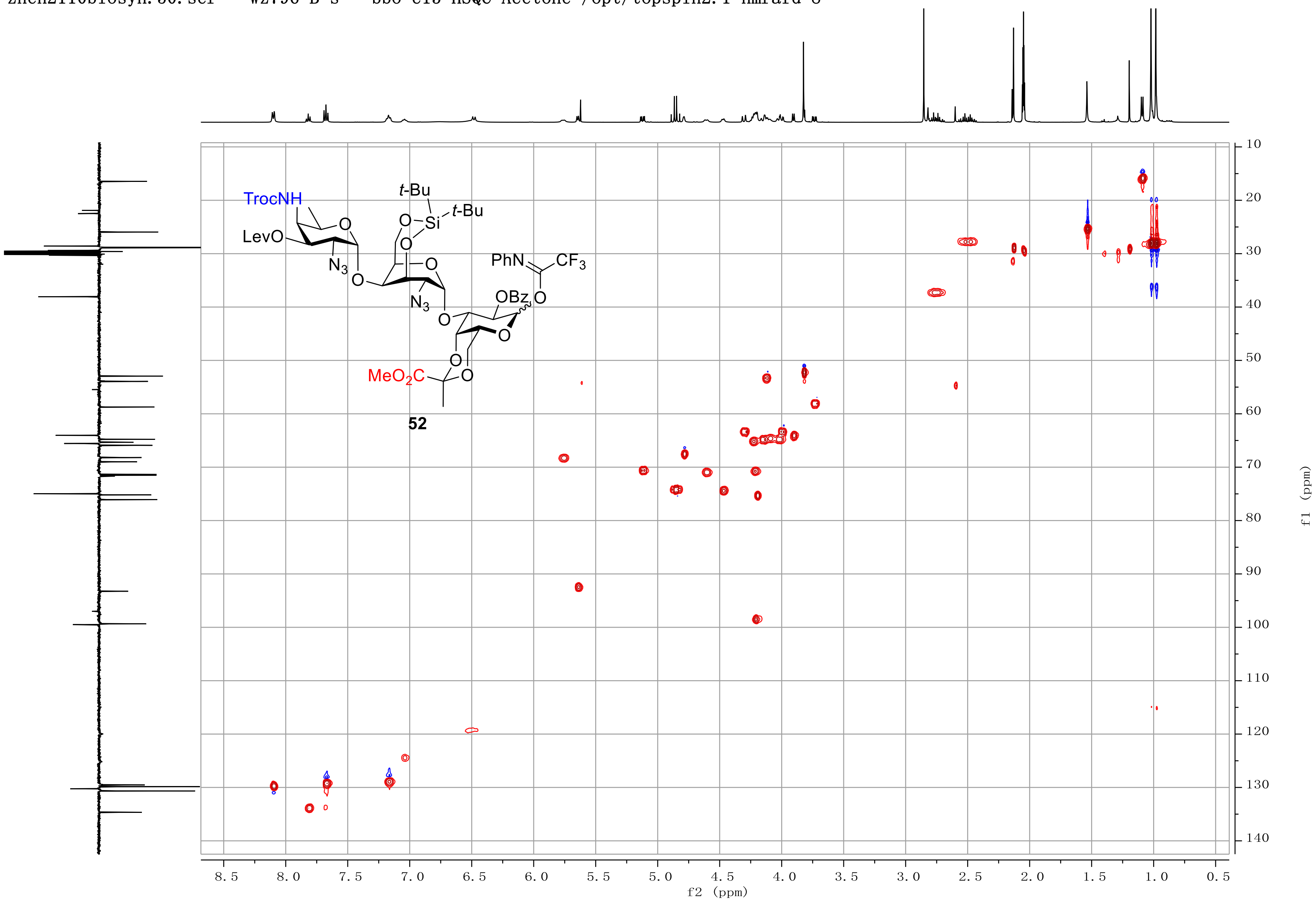

zhen2110biosyn.52.ser — wz796-B-s — bbo-c13-HMBC Acetone /opt/topspin2.1 nmrafd 8

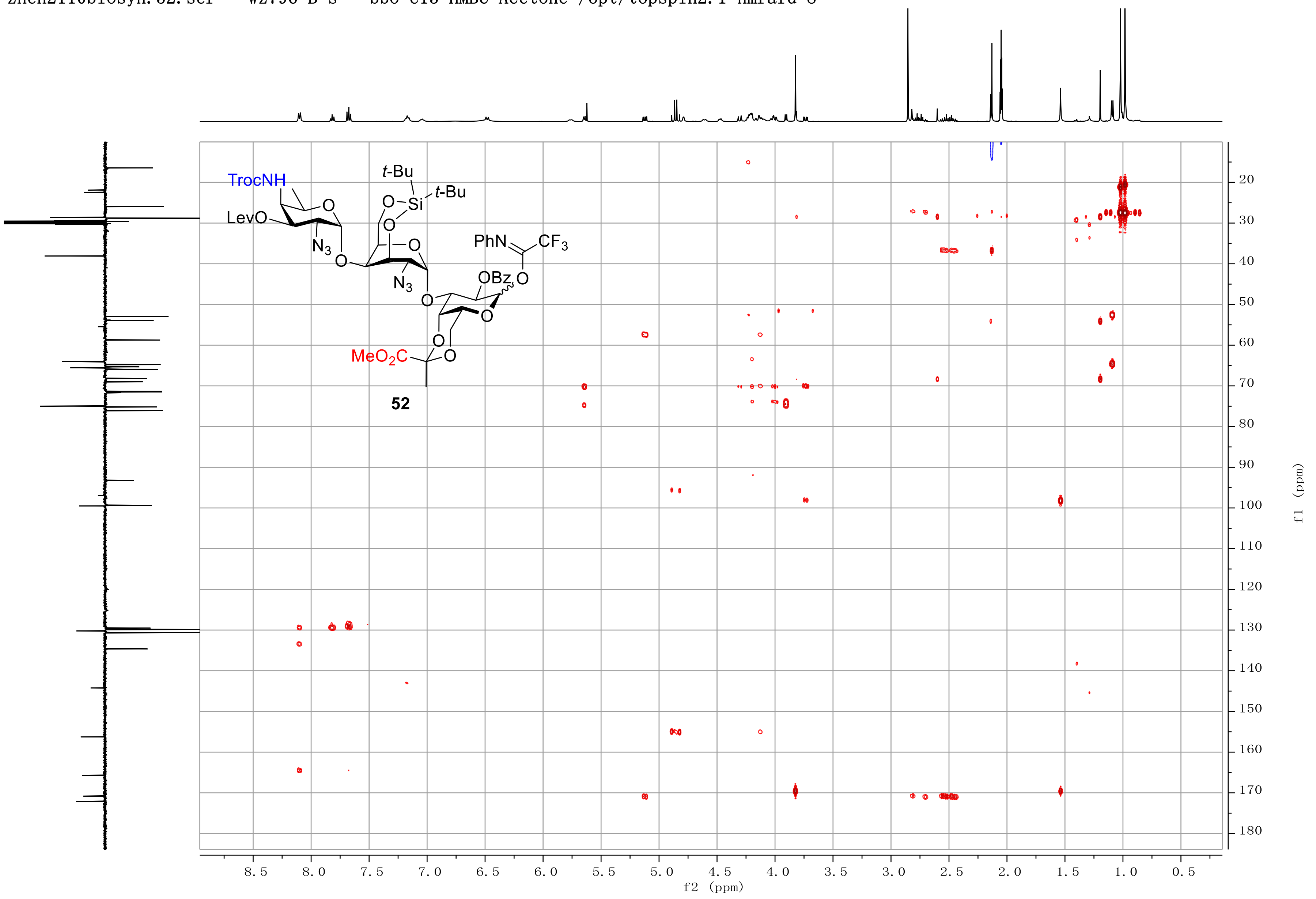

zhen2110biosyn.54.fid — wz811-A-s — bbo-h1 CDCl3 /opt/topspin2.1 nmrafd 14

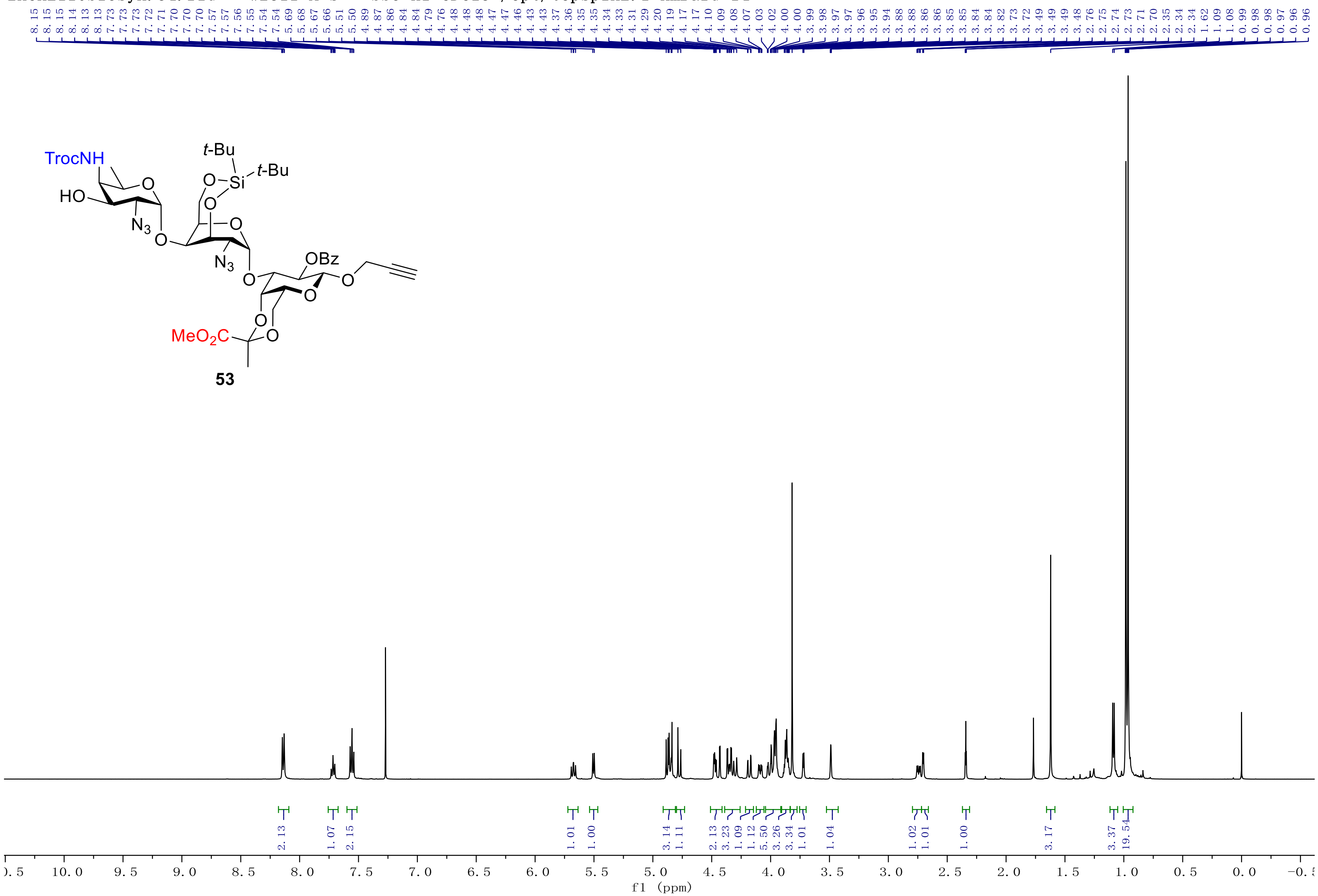

zhen2110biosyn.57.fid — wz811-A-s — bbo-c13-APT CDCl3 /opt/topspin2.1 nmrafd 14

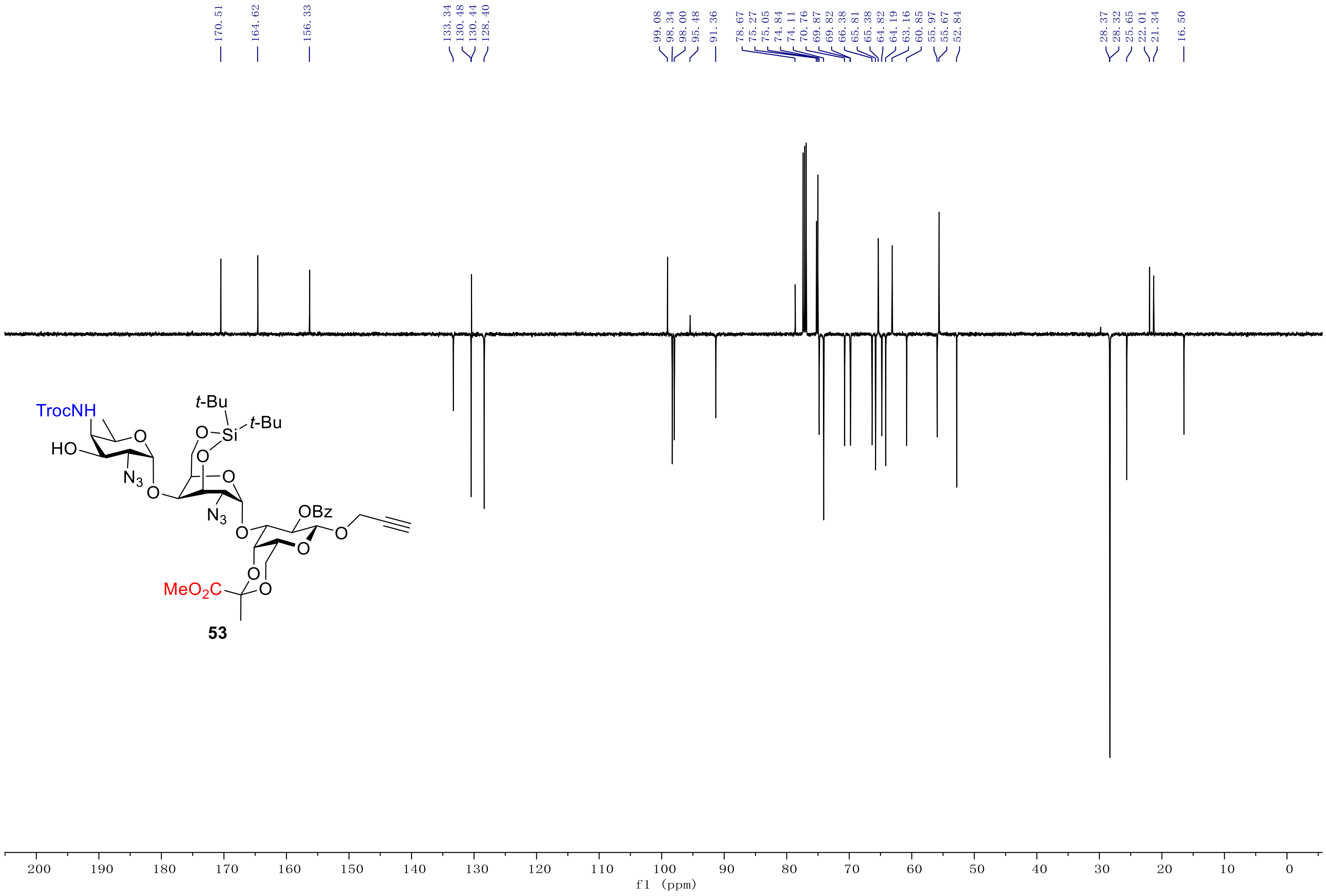

53

zhen2110biosyn.55.ser — wz811-A-s — bbo-h1-cosy CDCl3 /opt/topspin2.1 nmrafd 14

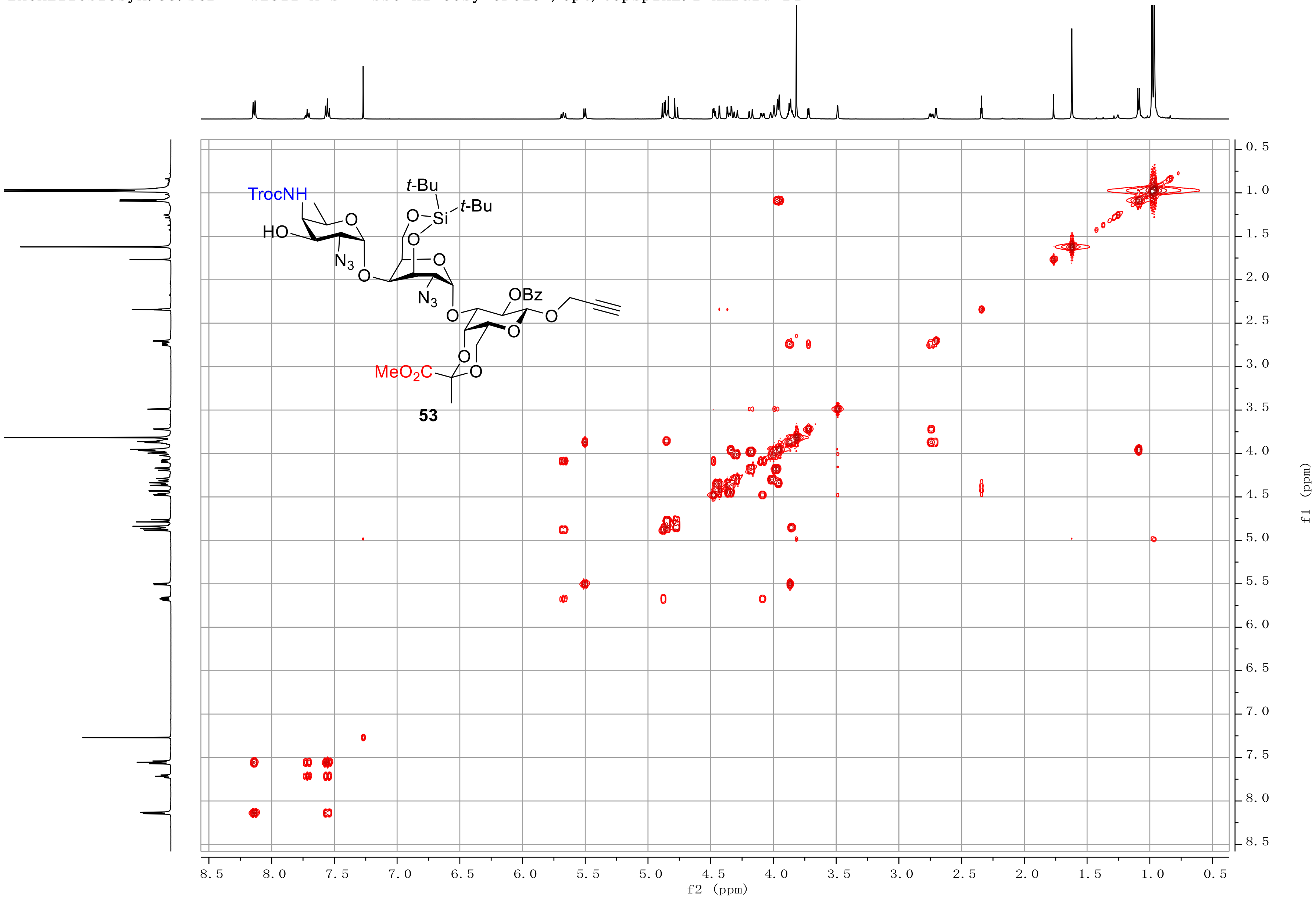

zhen2110biosyn.56.ser — wz811-A-s — bbo-c13-HSQC CDCl3 /opt/topspin2.1 nmrafd 14

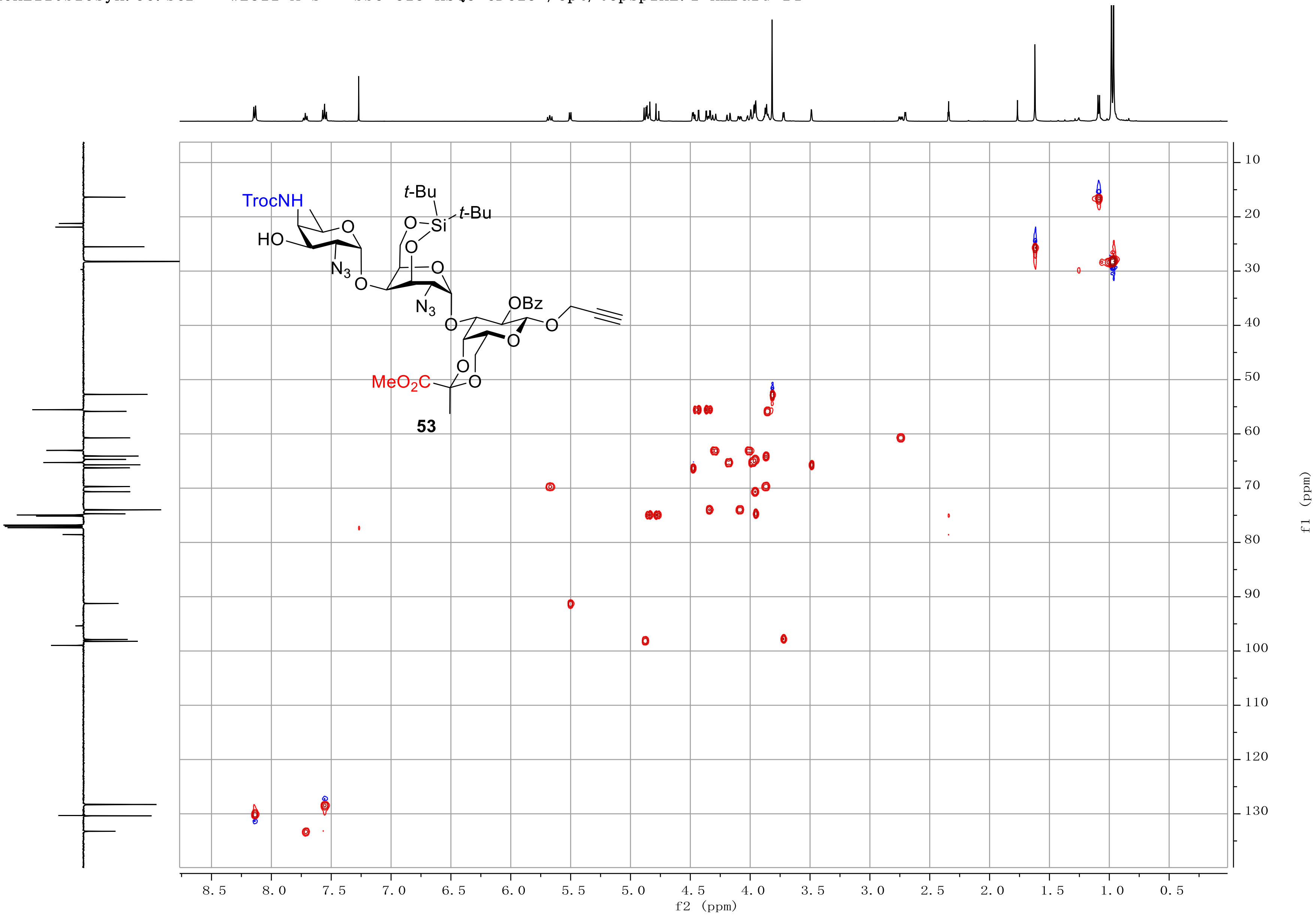

zhen2110biosyn.58.ser — wz811-A-s — bbo-c13-HMBC CDCl3 /opt/topspin2.1 nmrafd 14

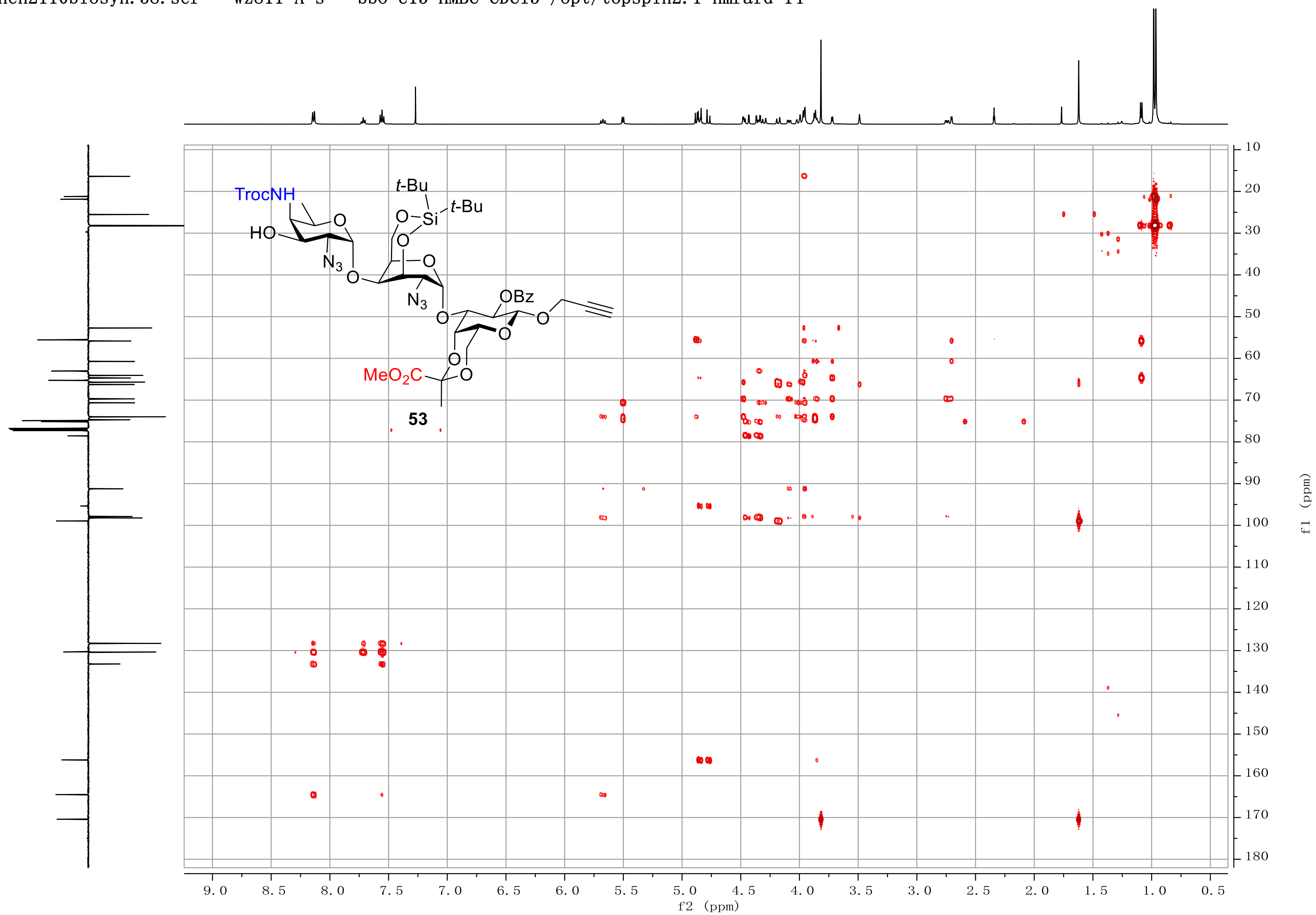

zhen2110biosyn.59.ser — wz811-A-s — bbo-c13-hmbc-ipv-gated CDCl3 /opt/topspin2.1 nmrafd 14

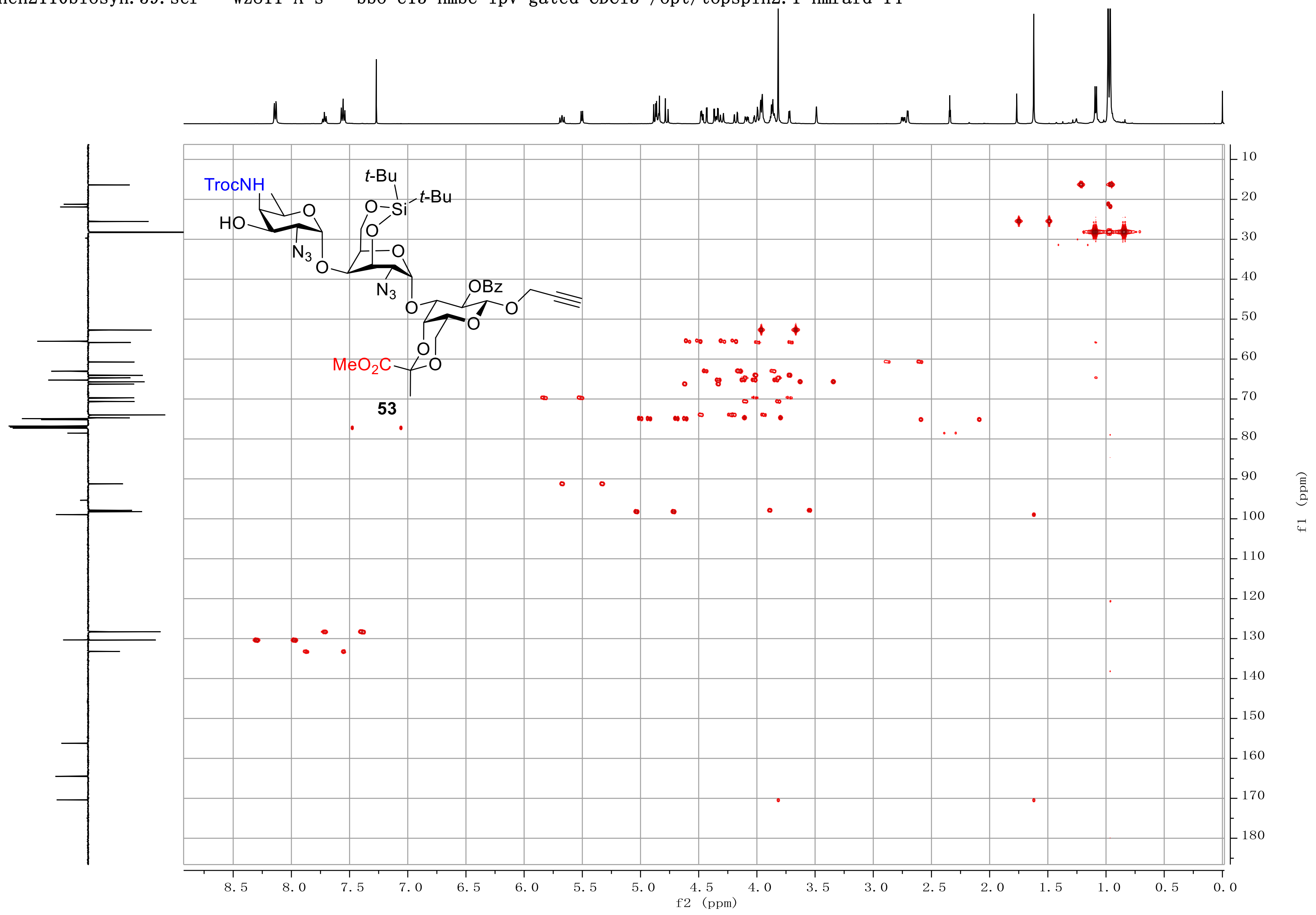

zhen2110biosyn.81.fid — wz813-B-s-again — 1H, bbo, av500

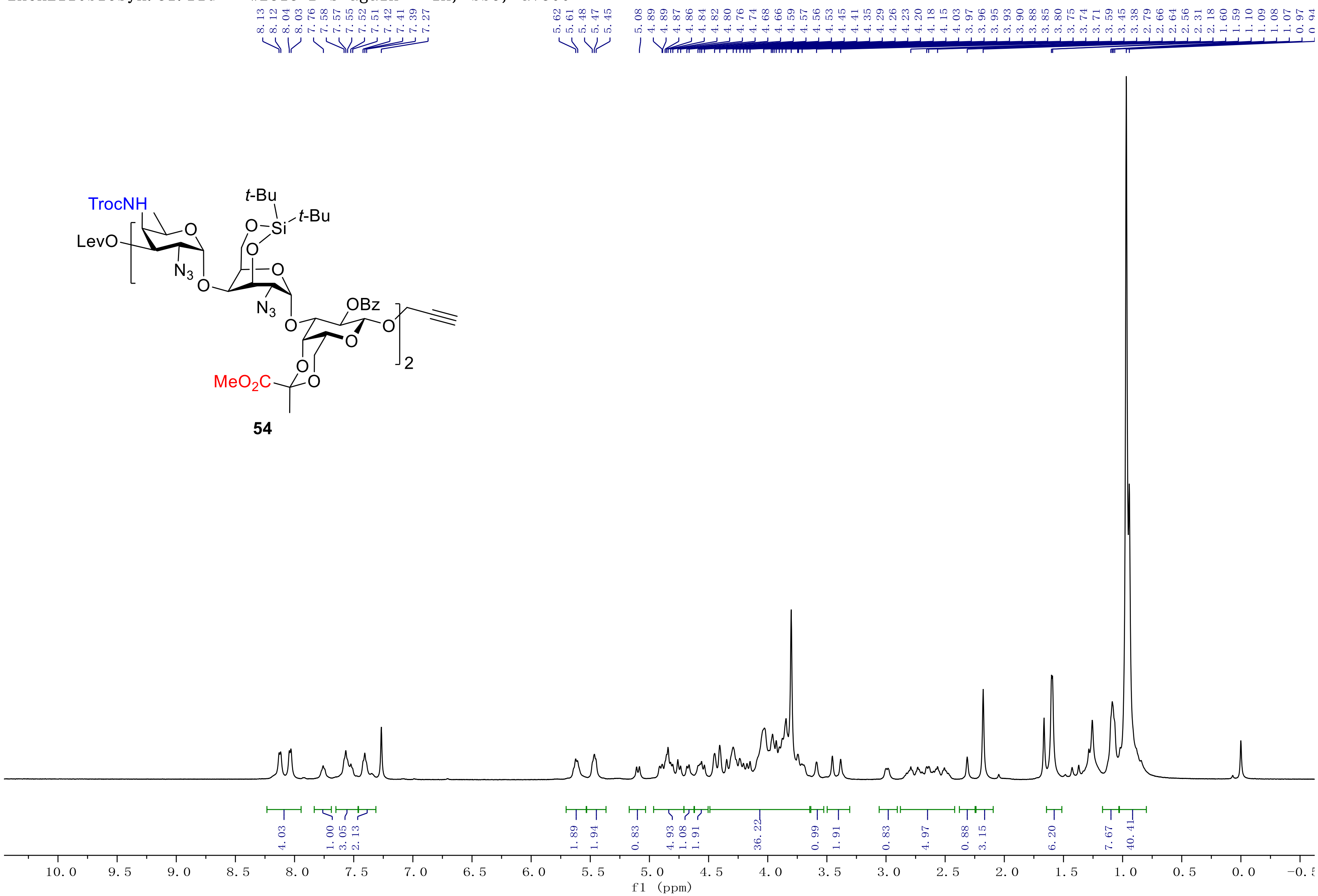

zhen2110biosyn.84.fid — wz813-b-s — bbo-c13-APT CDCl3 /opt/topspin2.1 nmrafd 10

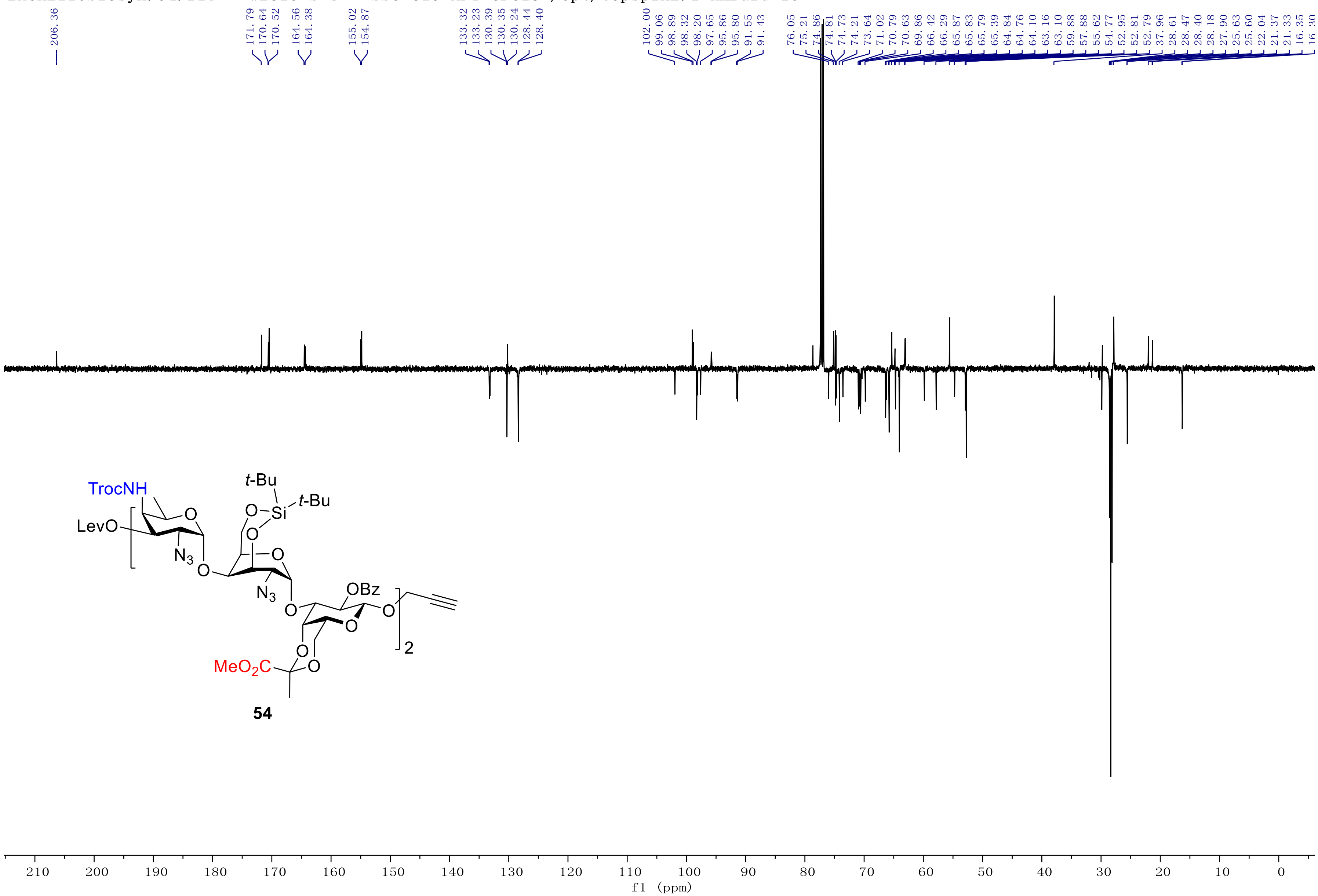

zhen2110biosyn.82.ser — wz813-b-s — bbo-h1-cosy CDCl3 /opt/topspin2.1 nmrafd 10

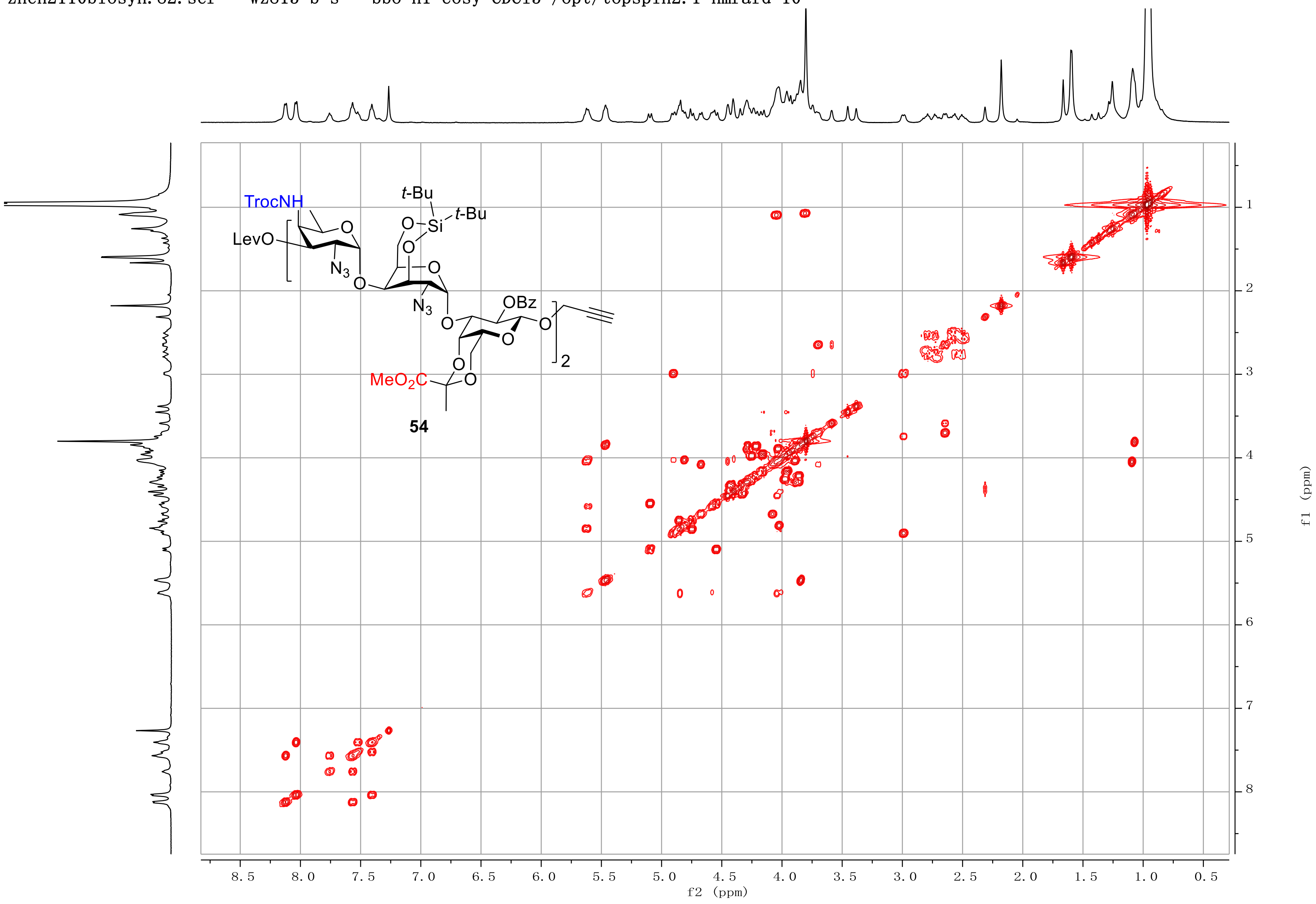

zhen2110biosyn.83.ser — wz813-b-s — bbo-c13-HSQC CDCl3 /opt/topspin2.1 nmrafd 10

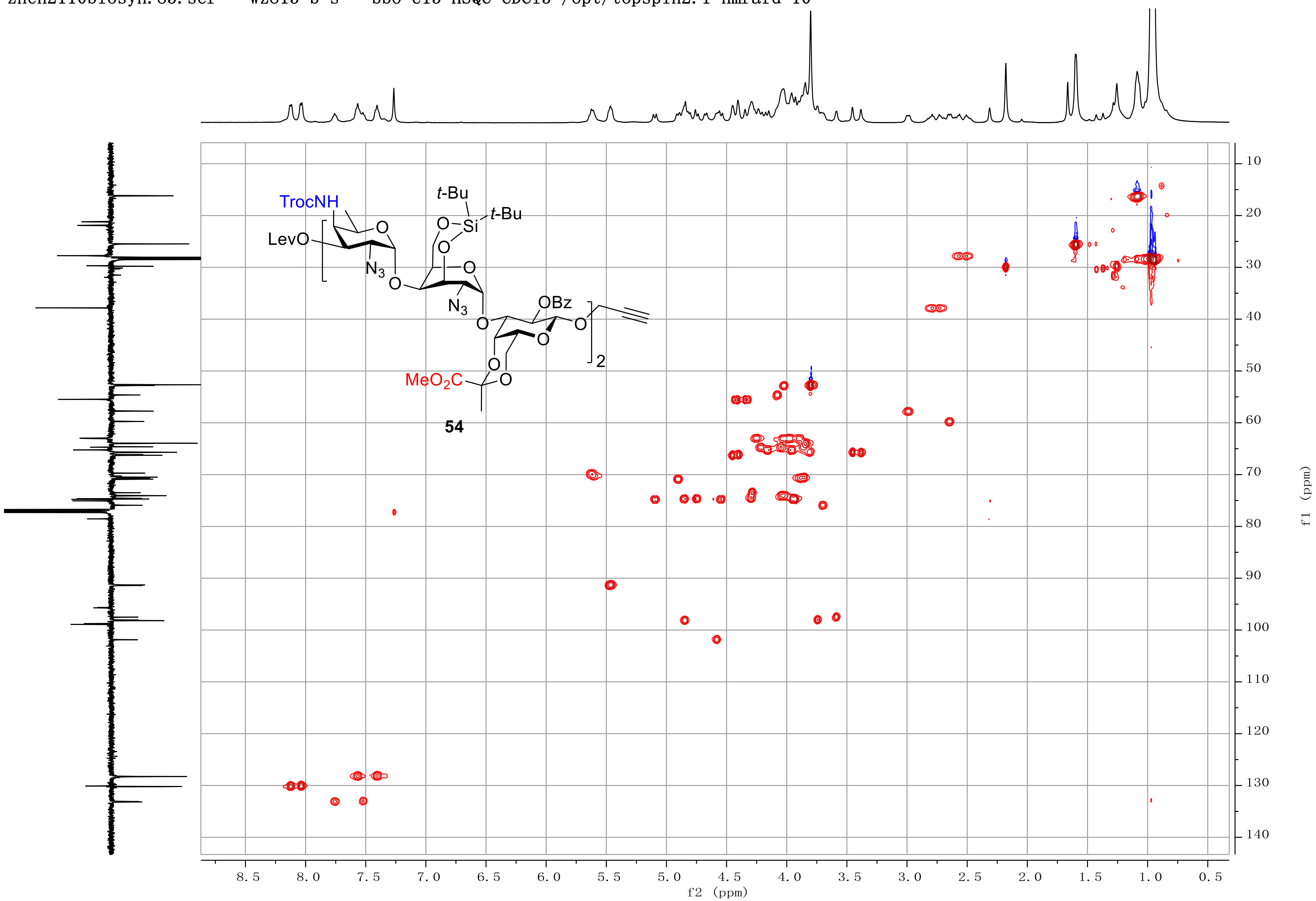

zhen2110biosyn.85.ser — wz813-b-s — bbo-c13-HMBC CDCl3 /opt/topspin2.1 nmrafd 10

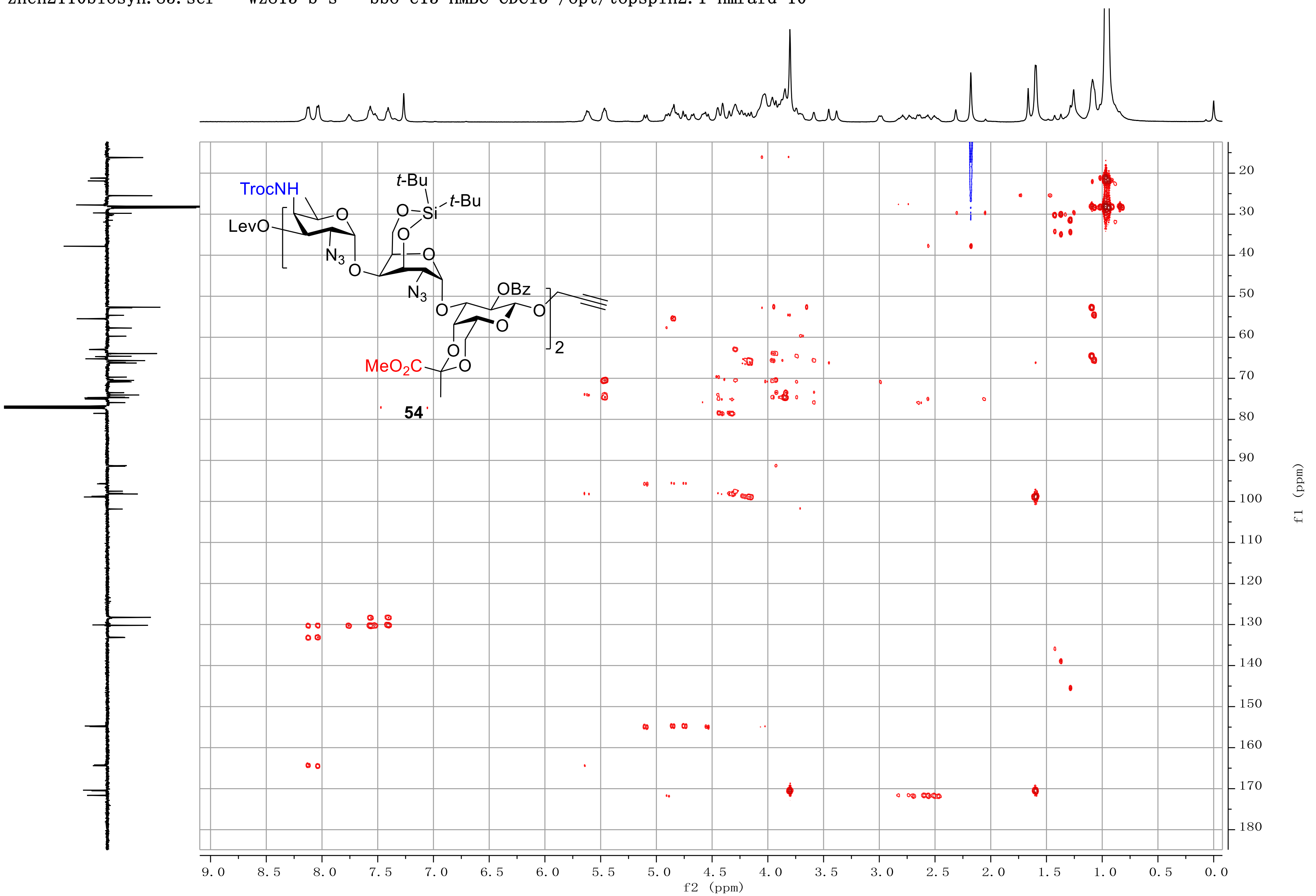

zhen2110biosyn.86.ser — wz813-b-s — bbo-c13-hmbc-ipv-gated CDCl3 /opt/topspin2.1 nmrafd 10

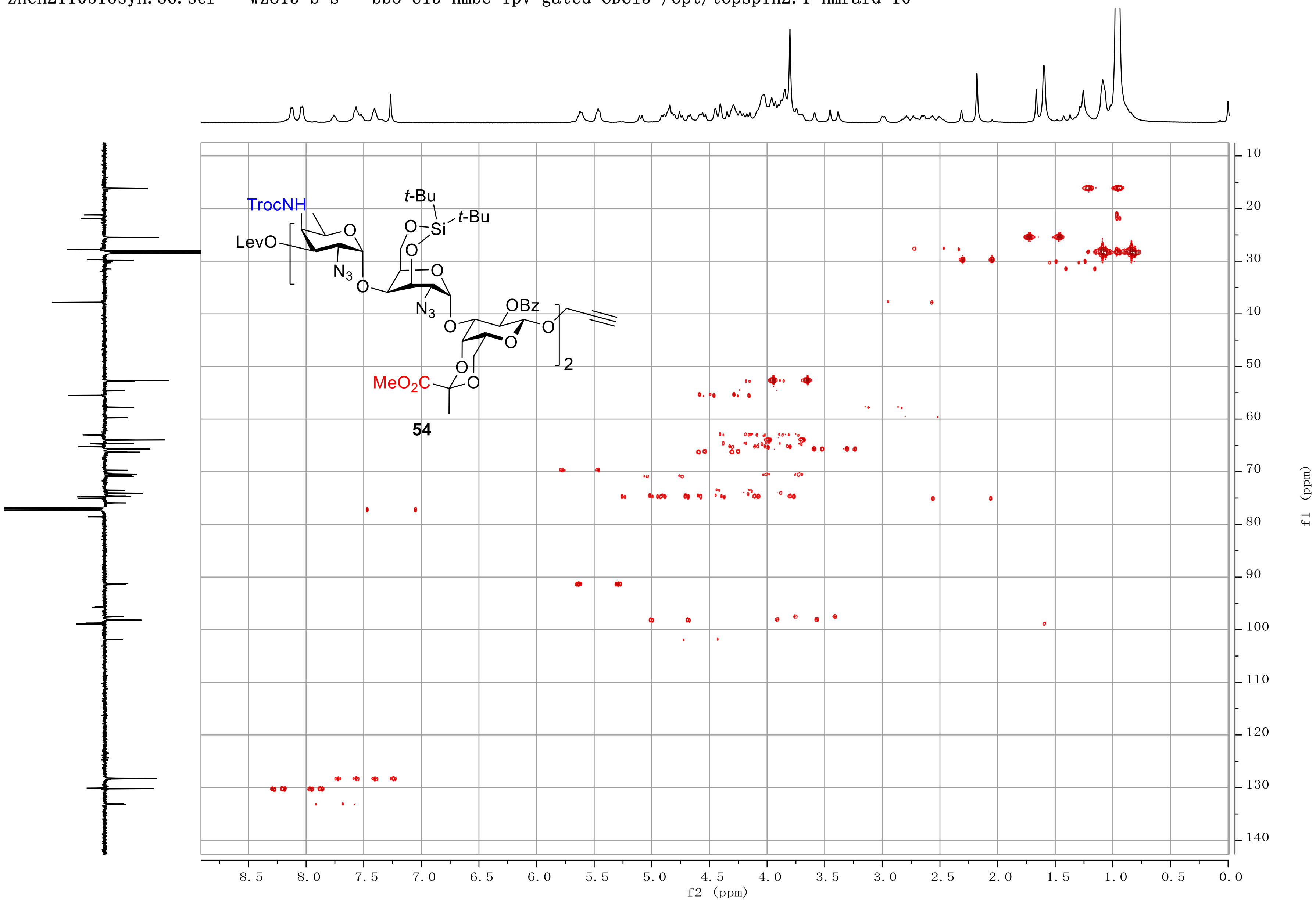

zhen2110biosyn.74.fid — wz818-a-s — bbo-h1 CDCl3 /opt/topspin2.1 nmrafd 10

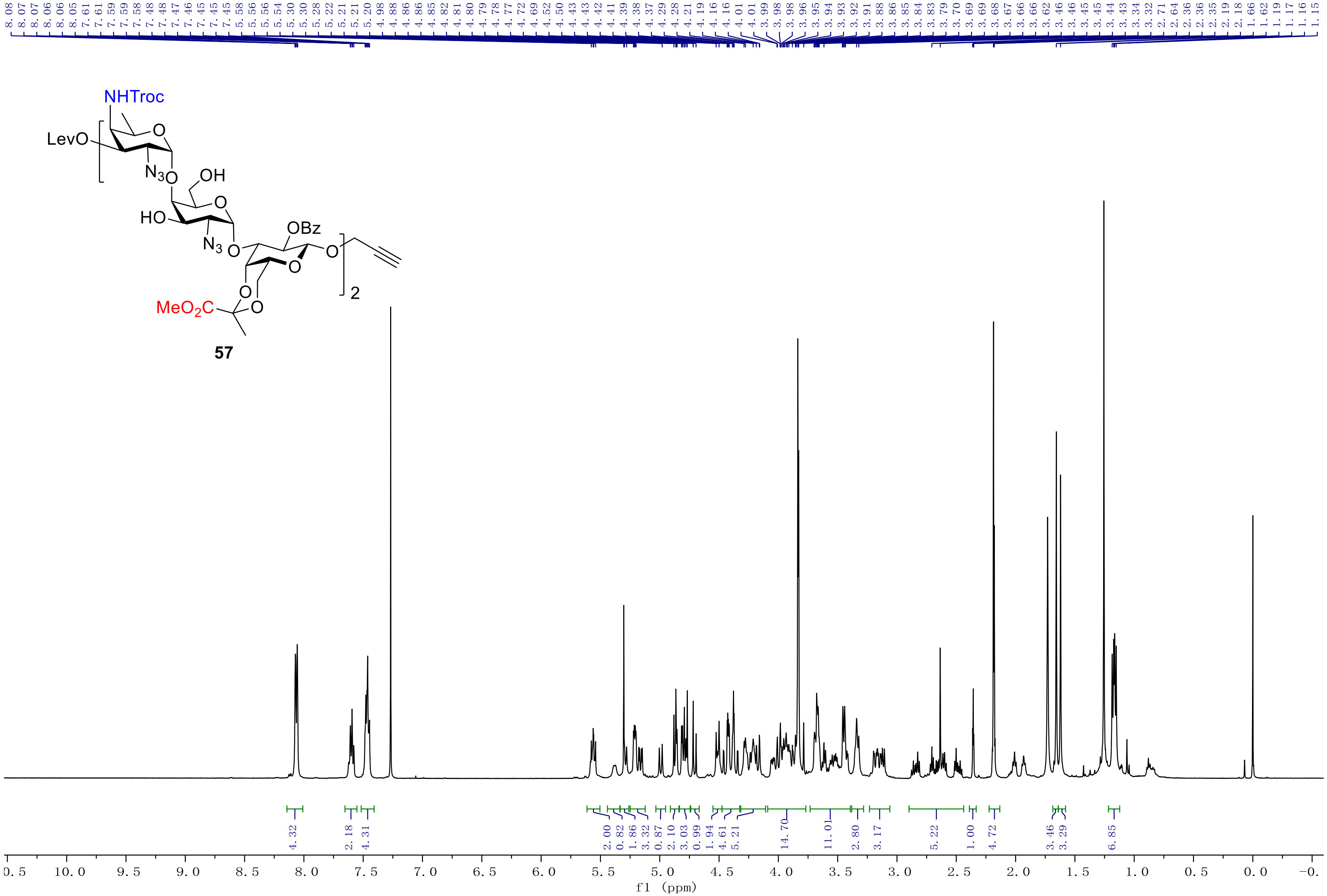

57

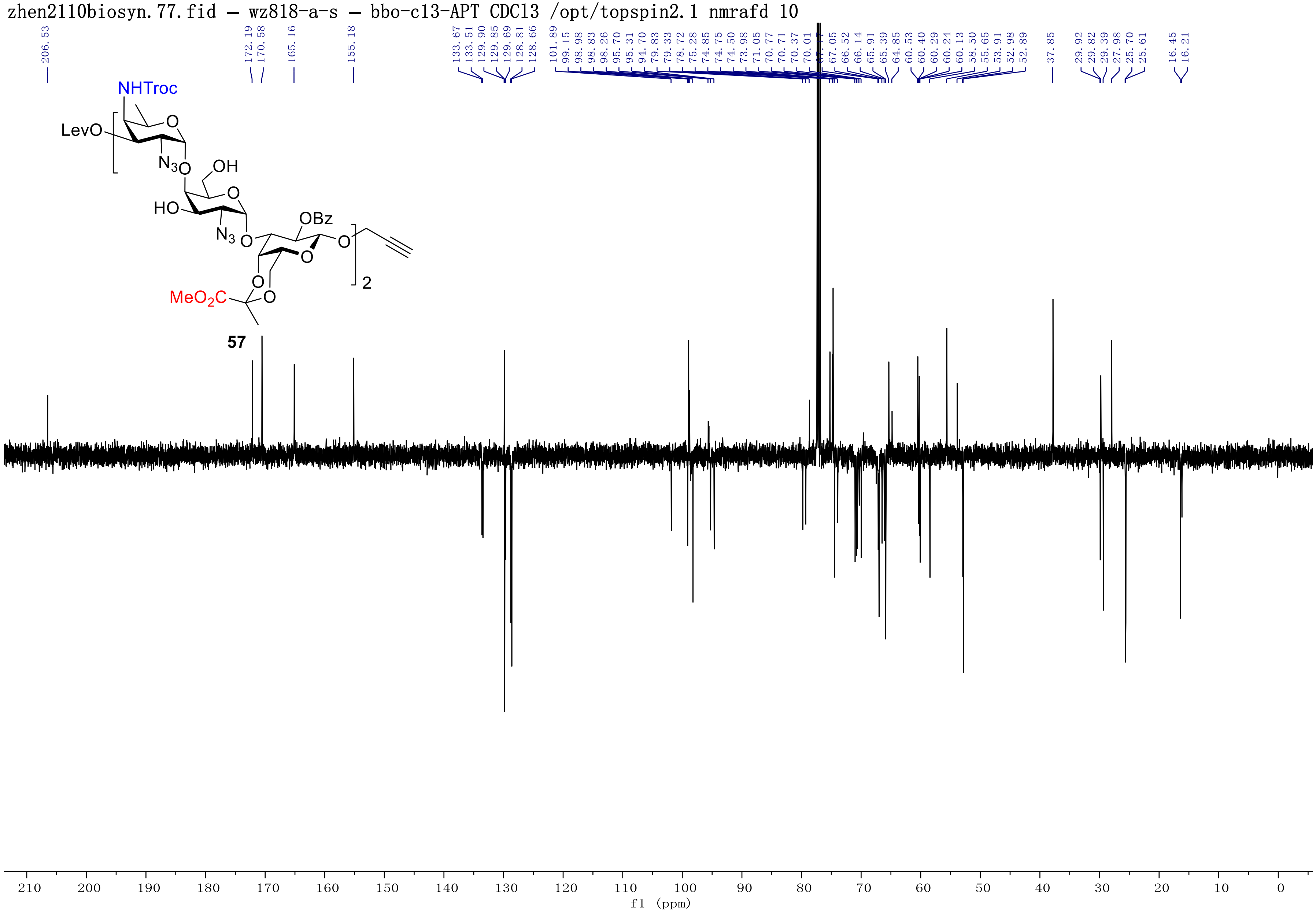

zhen2110biosyn.77.fid — wz818-a-s — bbo-c13-APT CDCl3 /opt/topspin2.1 nmrafd 10
206.53
172.19
170.58
165.16
155.18
133.67
133.51
129.90
129.85
129.69
128.81
128.66
101.89
99.15
98.98
98.83
98.26
95.70
95.31
94.70
79.83
79.33
78.72
75.28
74.85
74.75
74.50
73.98
71.05
70.77
70.71
70.37
70.01
67.05
66.52
66.14
65.91
65.39
64.85
60.53
60.40
60.29
60.24
60.13
58.50
55.65
53.91
52.98
52.89
37.85
29.92
29.82
29.39
27.98
25.70
25.61
16.45
16.21
NHTroc
LevO
O
N3
O
OH
O
HO
N3
O
OBz
O
O
2
O
O
MeO2C
57
210 200 190 180 170 160 150 140 130 120 110 100 90 80 70 60 50 40 30 20 10 0
f1 (ppm)

zhen2110biosyn.75.ser — wz818-a-s — bbo-h1-cosy CDCl3 /opt/topspin2.1 nmrafd 10

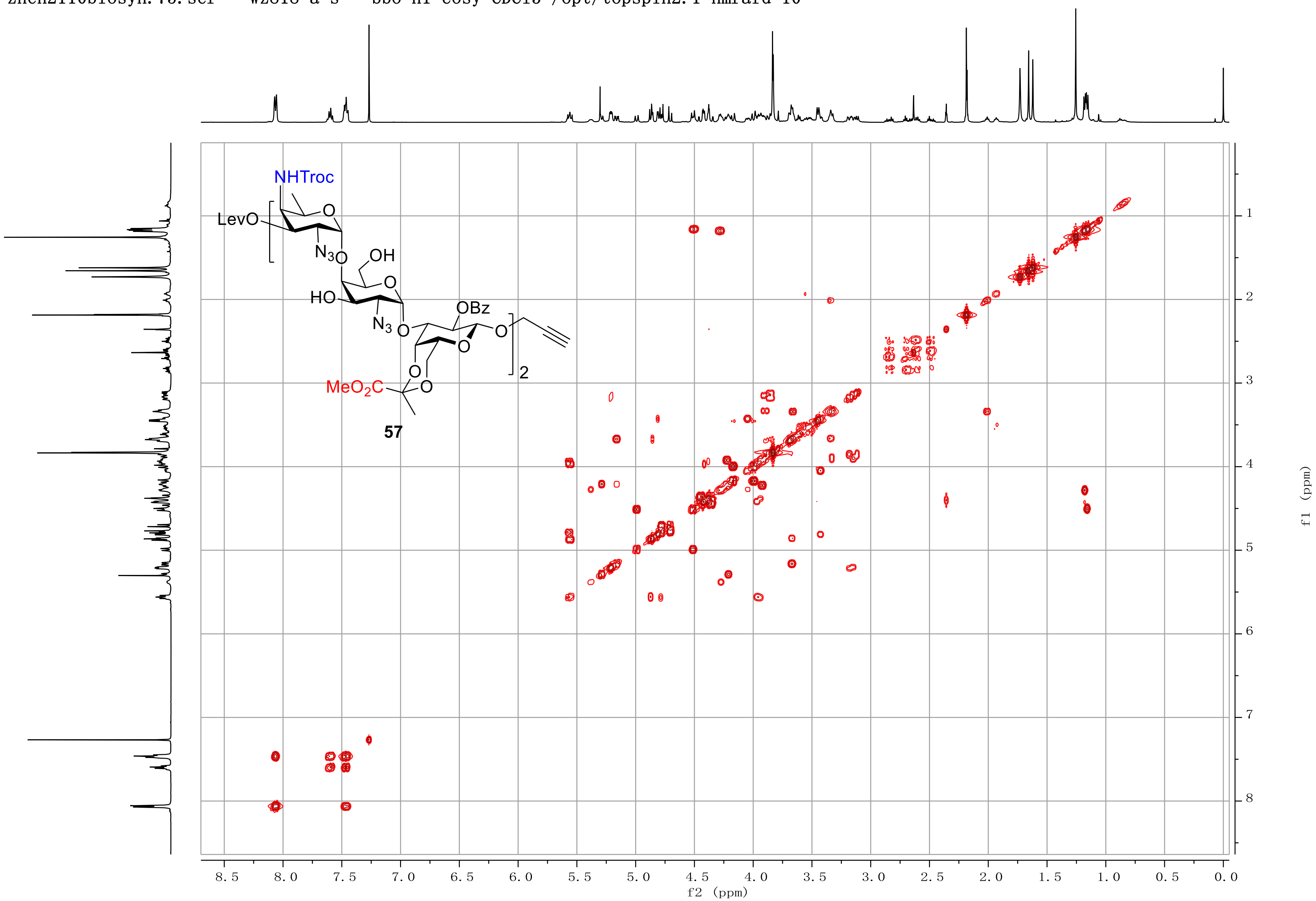

zhen2110biosyn.76.ser — wz818-a-s — bbo-c13-HSQC CDCl3 /opt/topspin2.1 nmrafd 10

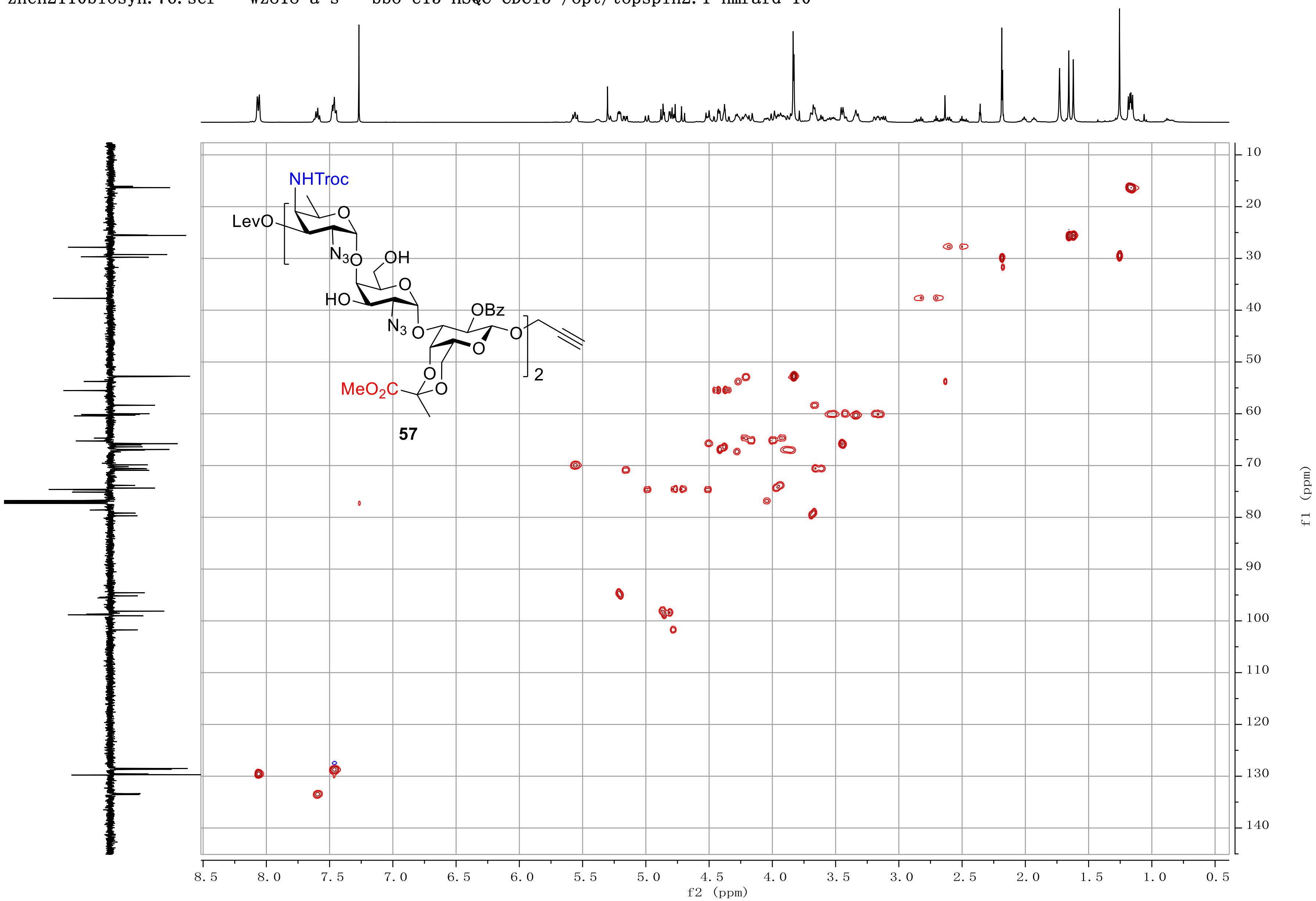

zhen2110biosyn.78.ser — wz818-a-s — bbo-c13-HMBC CDCl3 /opt/topspin2.1 nmrafd 10

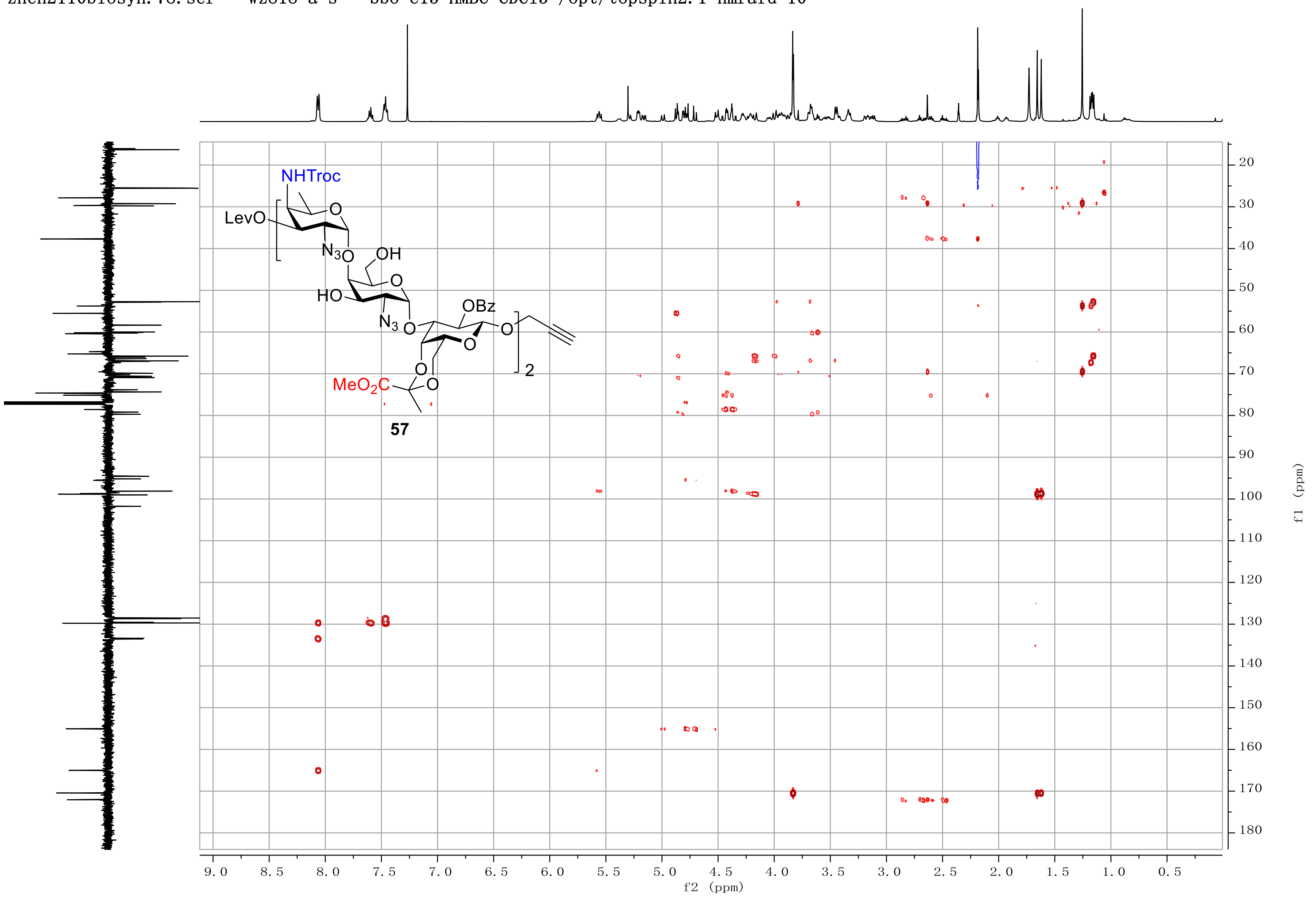

zhen2201biosyn.5.fid — wz839-b-s — bbo-h1 CDCl3 /opt/topspin2.1 nmrafd 3

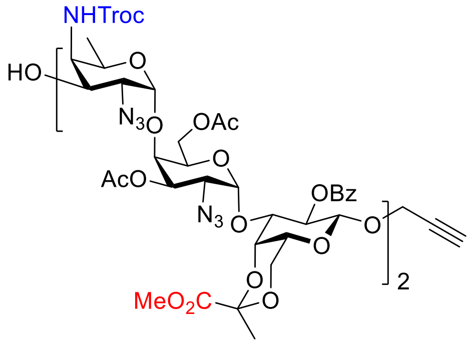

58

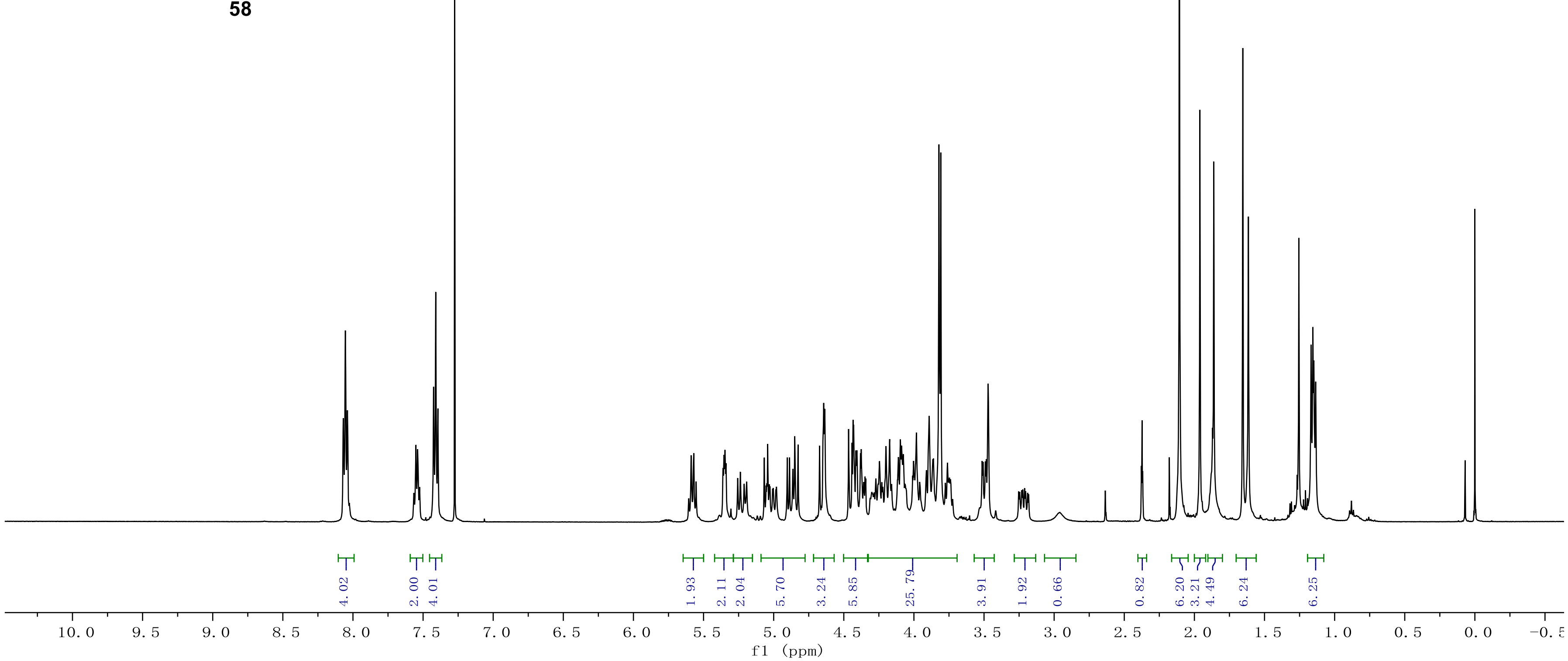

zhen2201biosyn.8.fid — wz839-b-s — bbo-c13-APT CDCl3 /opt/topspin2.1 nmrafd 3

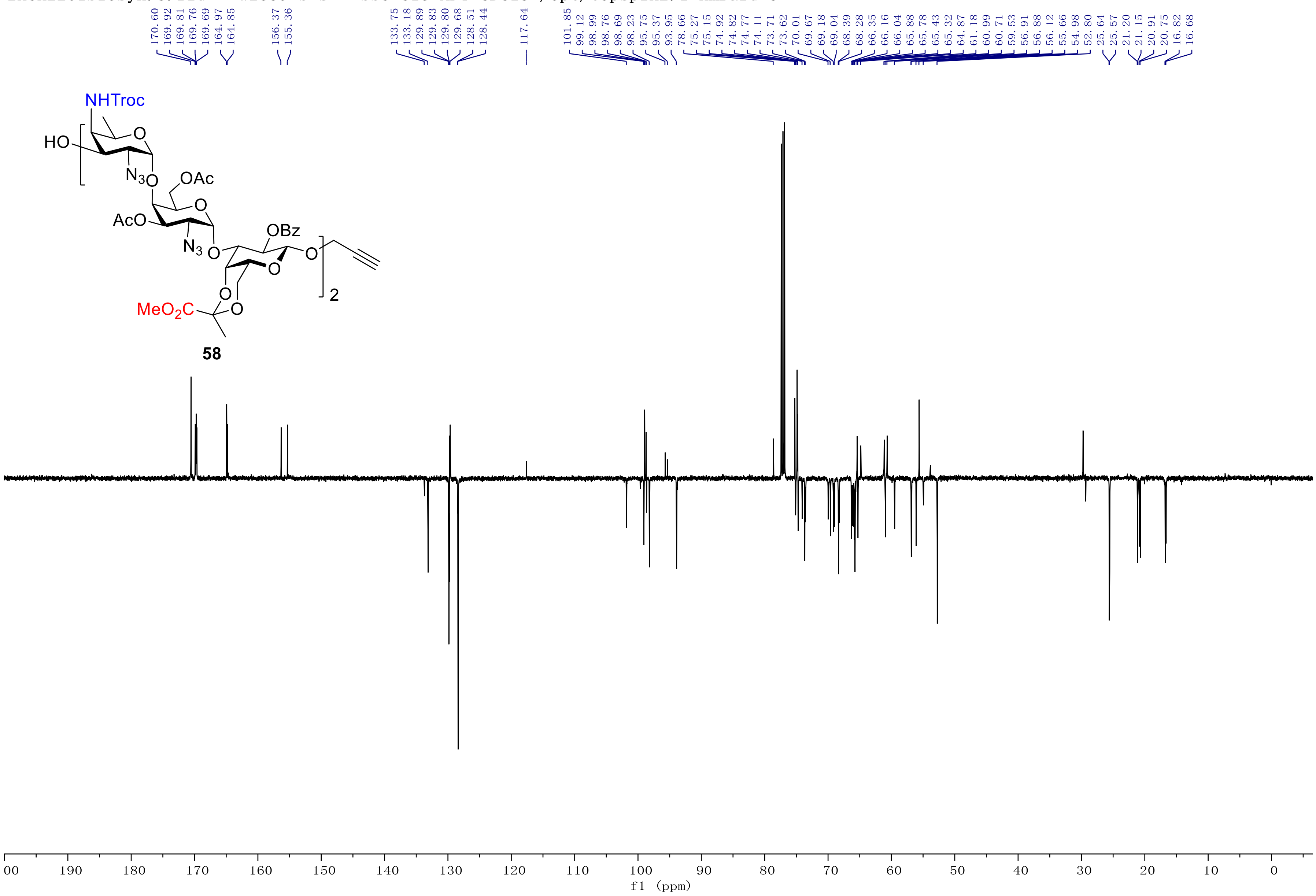

zhen2201biosyn.6.ser — wz839-b-s — bbo-h1-cosy CDCl3 /opt/topspin2.1 nmrafd 3

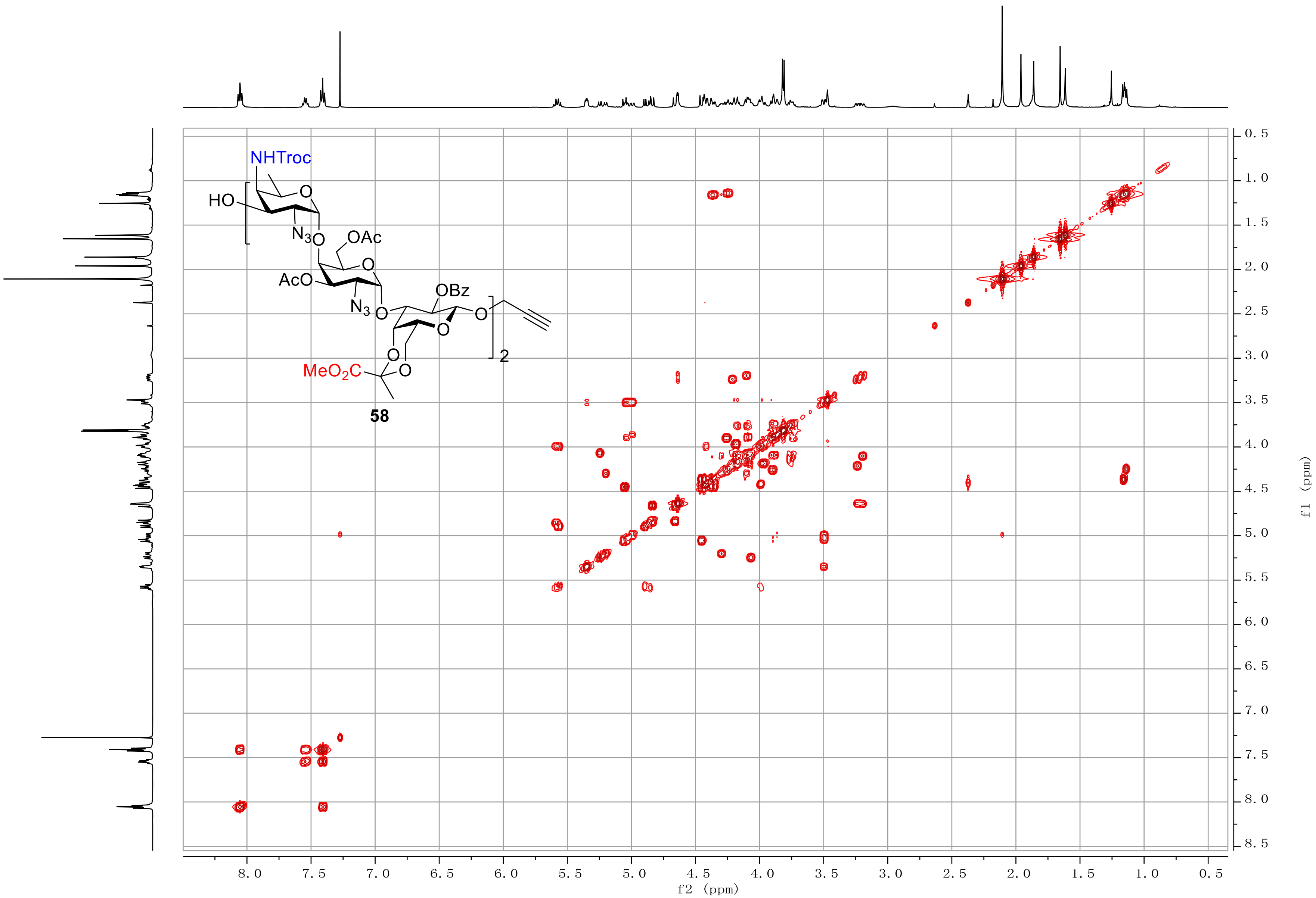

zhen2201biosyn.7.ser — wz839-b-s — bbo-c13-HSQC CDCl3 /opt/topspin2.1 nmrafd 3

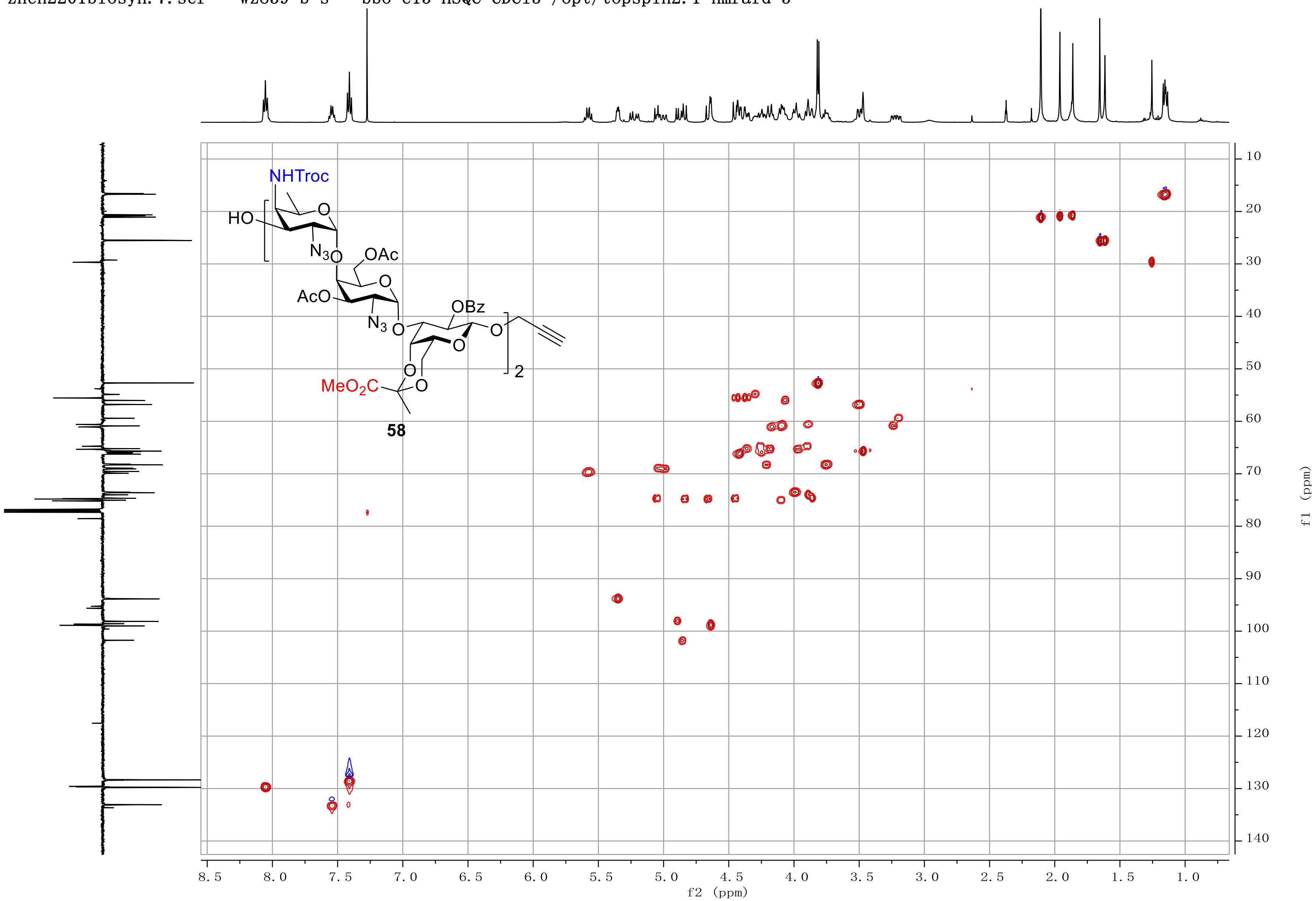

zhen2201biosyn.9.ser — wz839-b-s — bbo-c13-HMBC CDCl3 /opt/topspin2.1 nmrafd 3

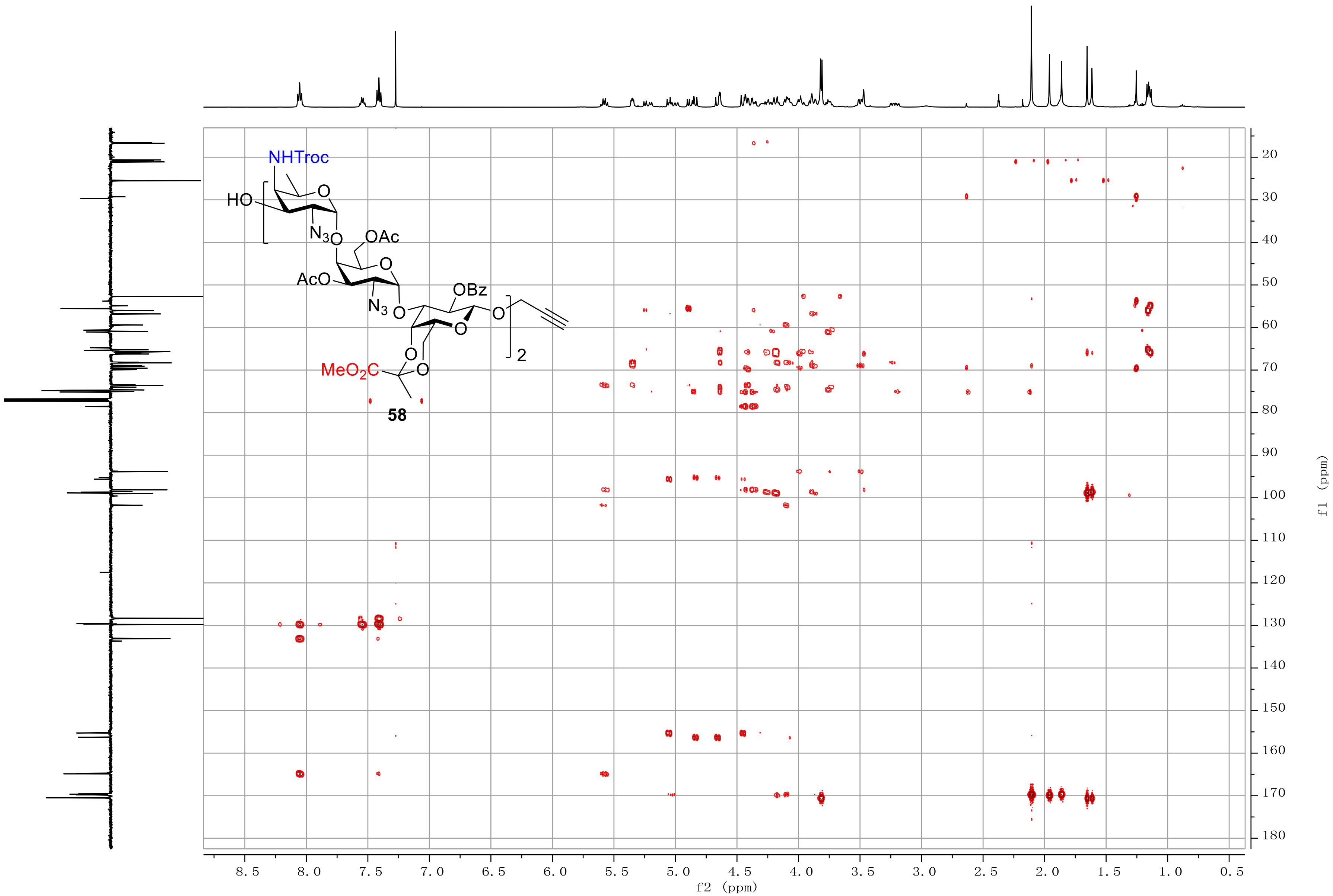

zhen2201biosyn.10.ser — wz839-b-s — bbo-c13-hmbc-ipv-gated CDCl3 /opt/topspin2.1 nmrafd 3

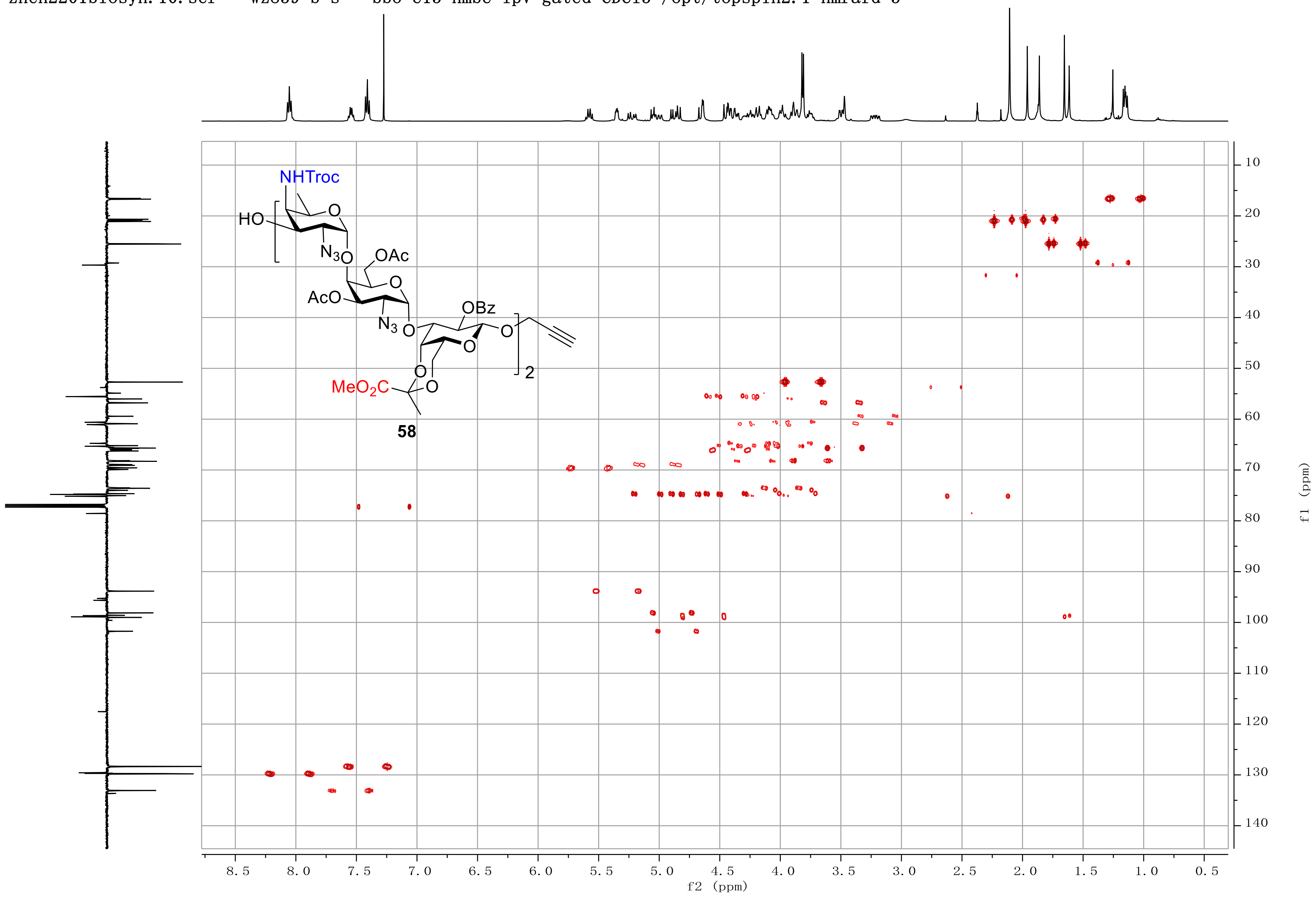

zhen2112biosyn.55.fid — wz841-A-HW40; — 1H, bbo, av500

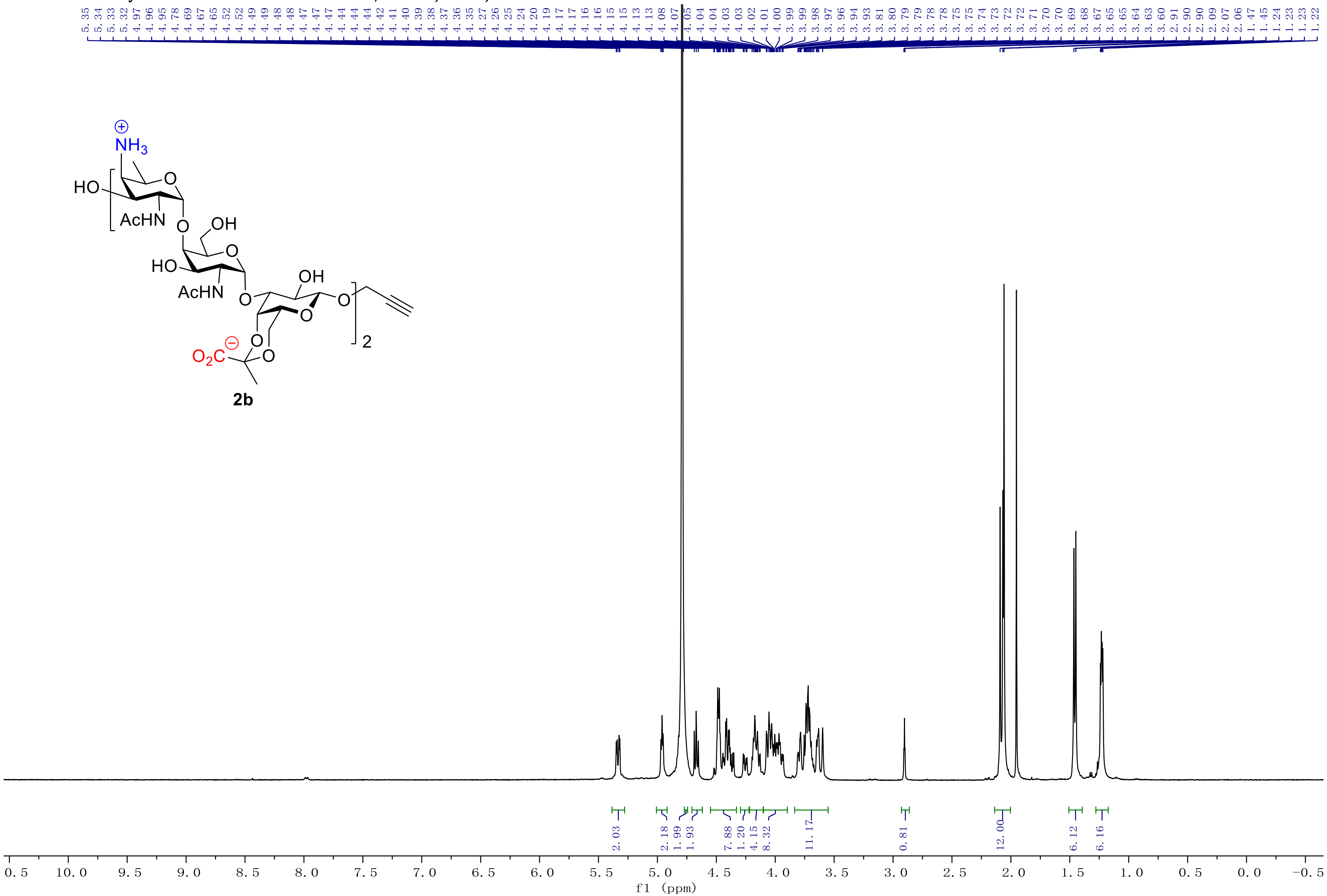

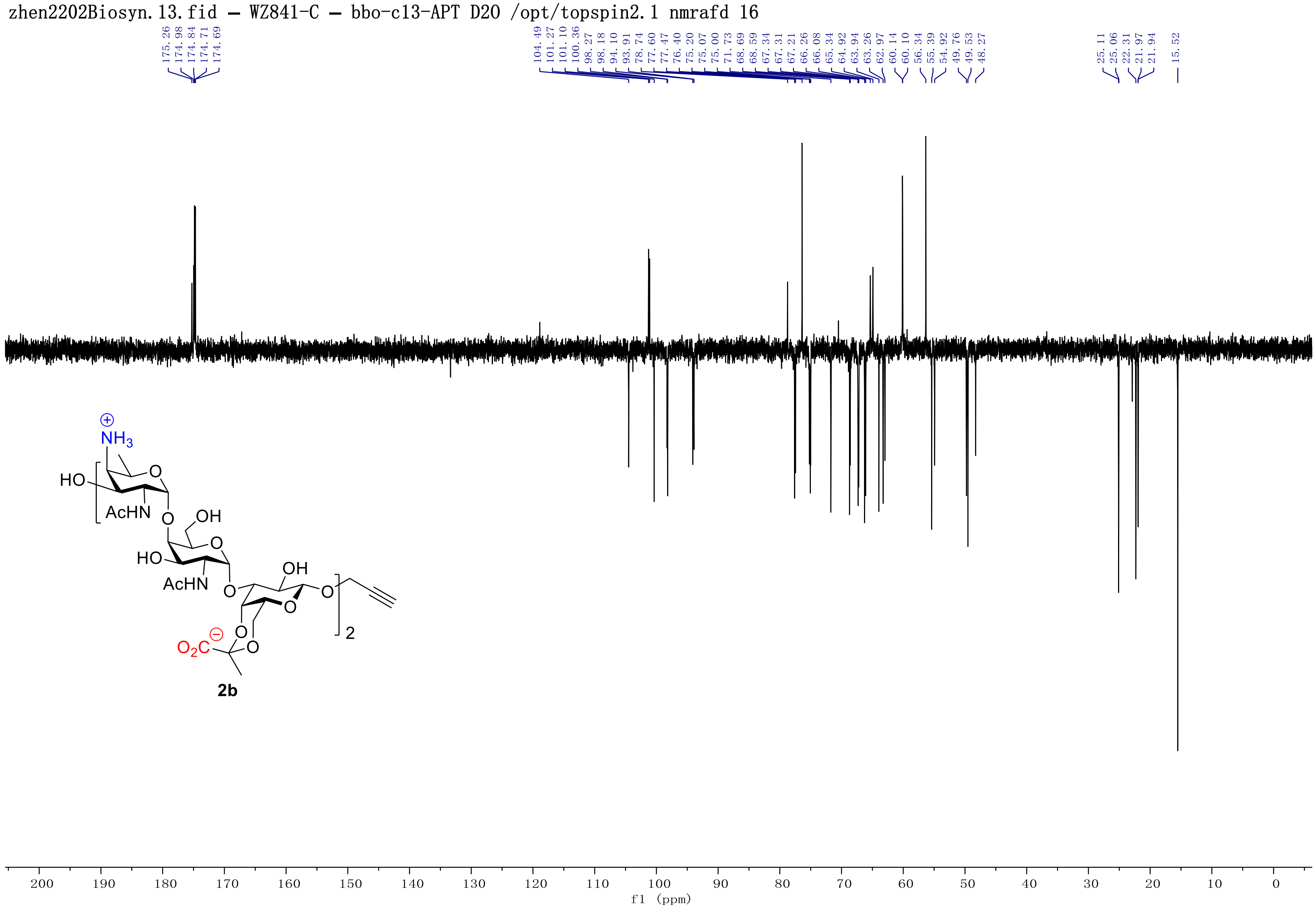
zhen2202Biosyn.13.fid — WZ841-C — bbo-c13-APT D2O /opt/topspin2.1 nmrafd 16
175.26
174.98
174.84
174.71
174.69
104.49
101.27
101.10
100.36
98.27
98.18
94.10
93.91
78.74
77.60
77.47
76.40
75.20
75.07
75.00
71.73
68.69
68.59
67.34
67.31
67.21
66.26
66.08
65.34
64.92
63.94
63.26
62.97
60.14
60.10
56.34
55.39
54.92
49.76
49.53
48.27
25.11
25.06
22.31
21.97
21.94
15.52
NH3
HO
AcHN
O
OH
HO
AcHN
O
OH
O
O
O2C
O
O
2
2b
200
190
180
170
160
150
140
130
120
110
100
90
80
70
60
50
40
30
20
10
0
f1 (ppm)

zhen2202Biosyn.11.ser — WZ841-C — bbo-h1-cosy D2O /opt/topspin2.1 nmrafd 16

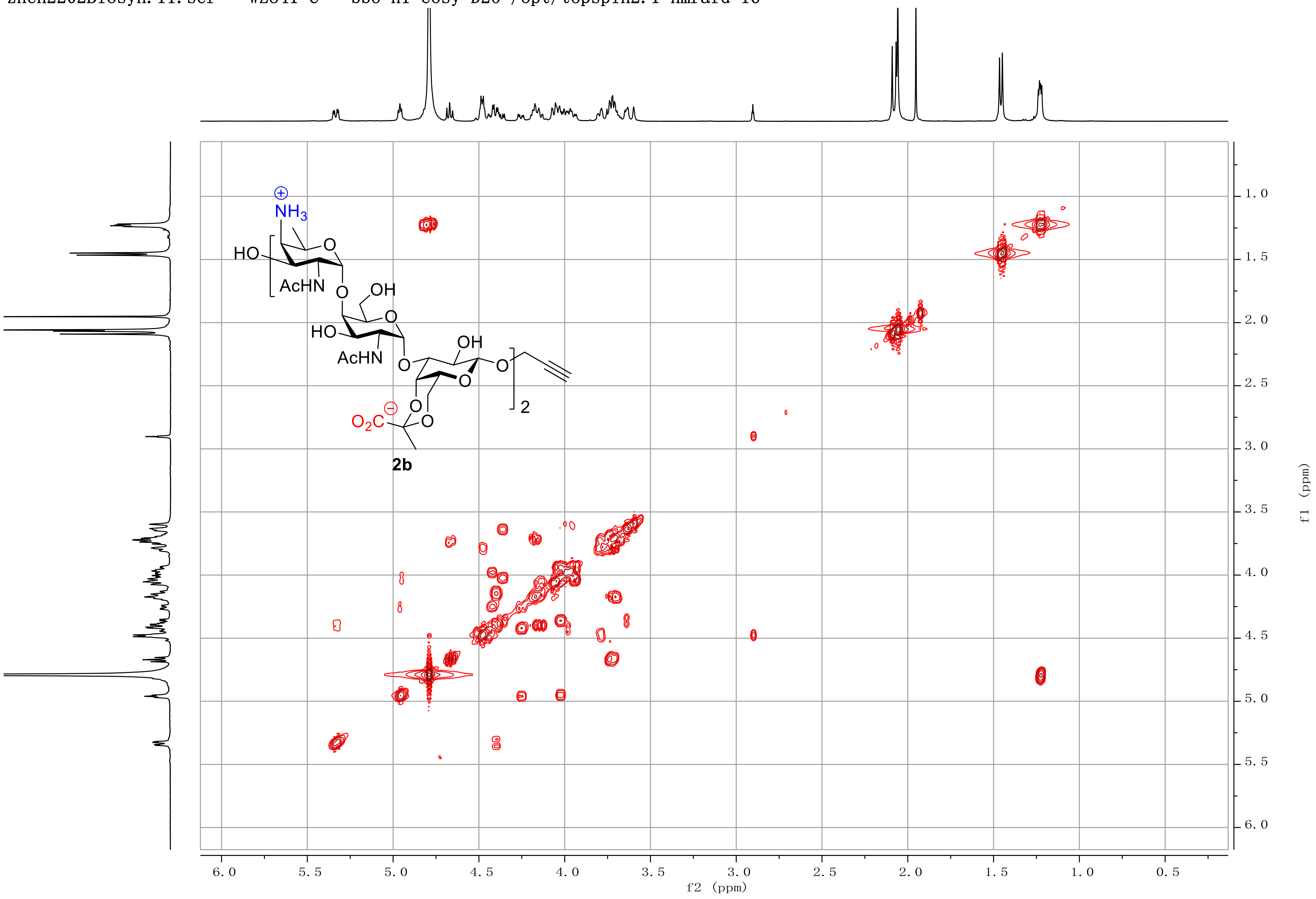

zhen2202Biosyn.12.ser — WZ841-C — bbo-c13-HSQC D2O /opt/topspin2.1 nmrafd 16

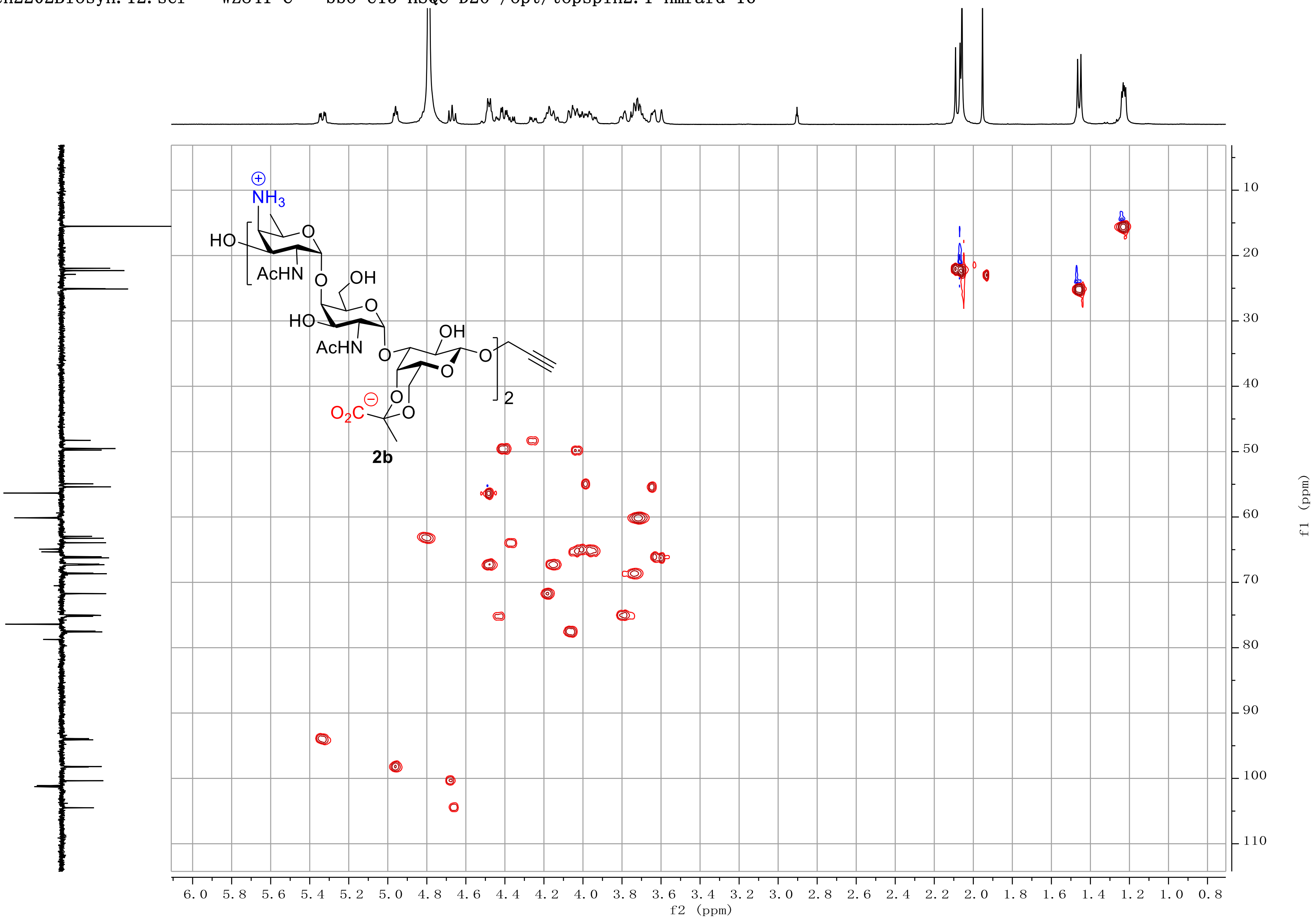

zhen2202Biosyn.14.ser — WZ841-C — bbo-c13-HMBC D2O /opt/topspin2.1 nmrafd 16

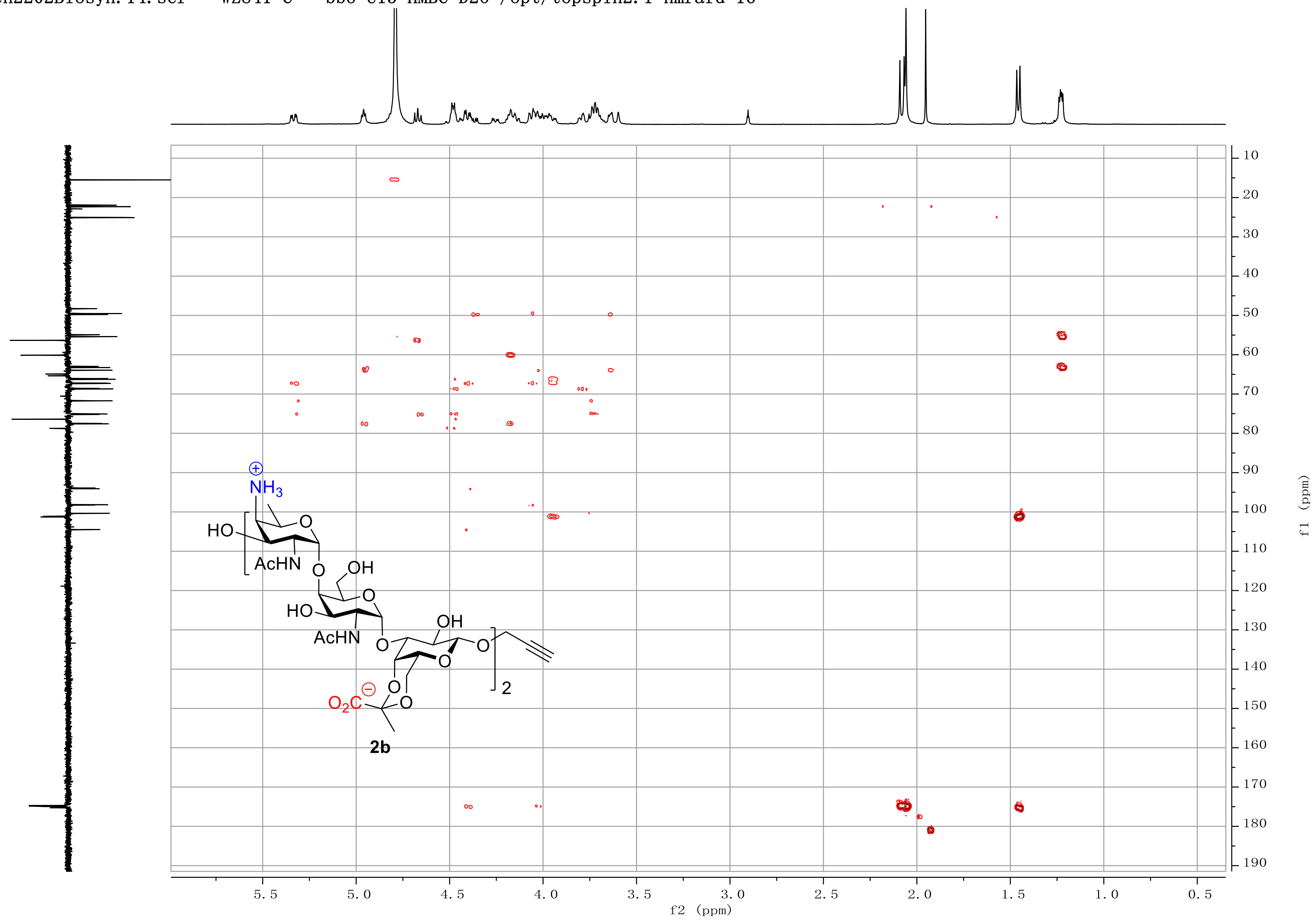

zhen2112biosyn.1.fid — wz822-A-2-s — bbo-h1 CDCl3 /opt/topspin2.1 nmrafd 13

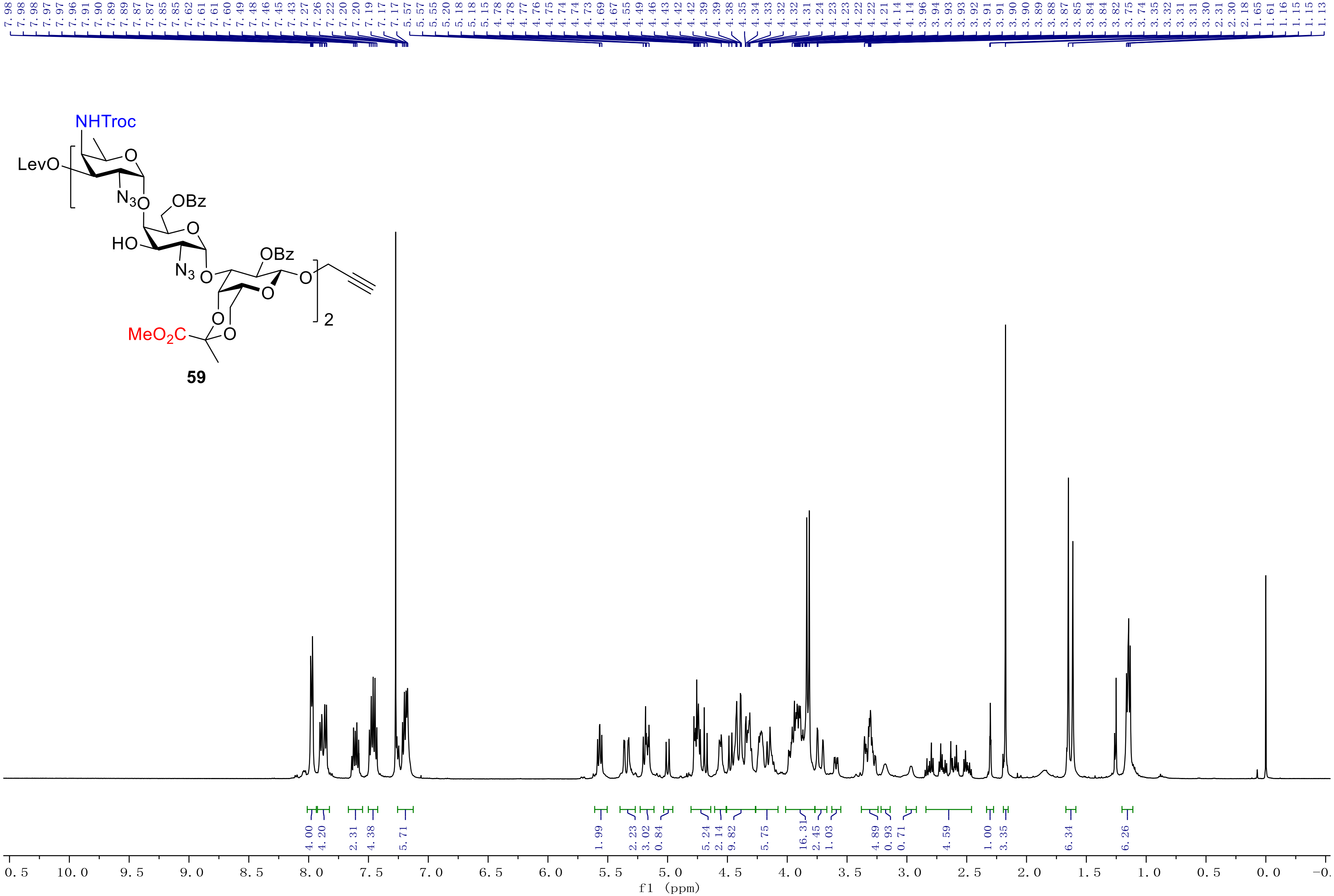

59

zhen2112biosyn.4.fid — wz822-A-2-s — bbo-c13-APT CDCl3 /opt/topspin2.1 nmrafd 13

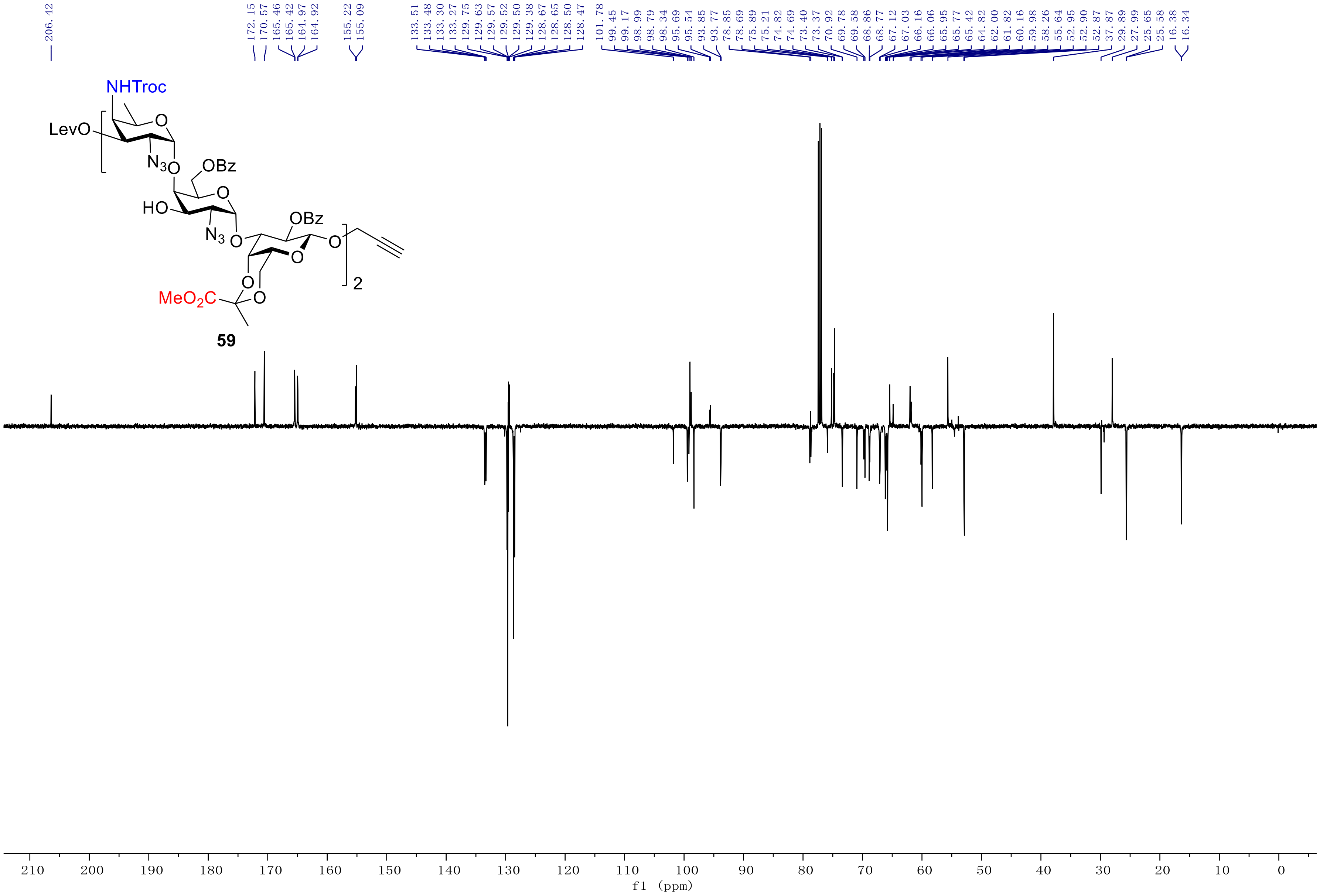

zhen2112biosyn.2.ser — wz822-A-2-s — bbo-h1-cosy CDCl3 /opt/topspin2.1 nmrafd 13

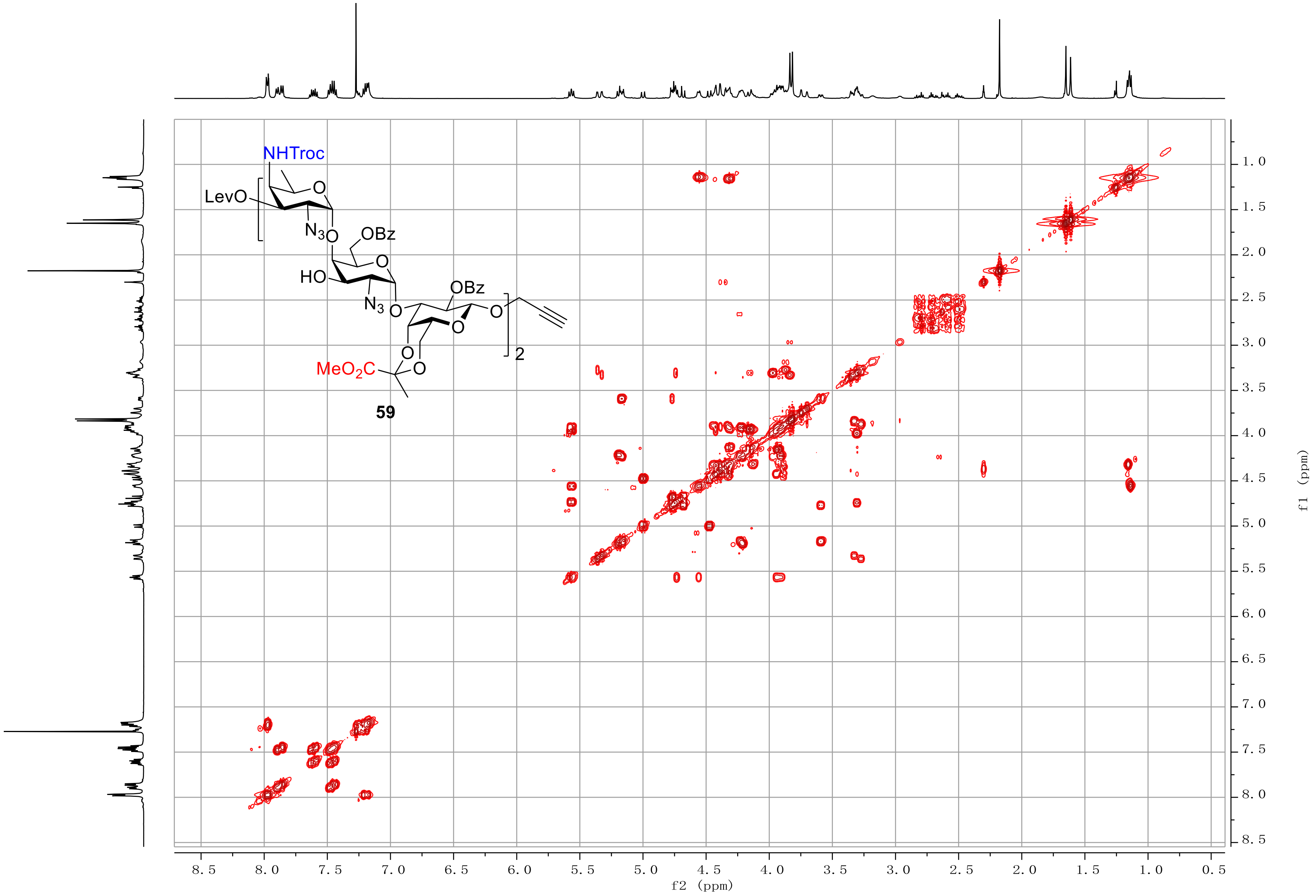

zhen2112biosyn.3.ser — wz822-A-2-s — bbo-c13-HSQC CDCl3 /opt/topspin2.1 nmrafd 13

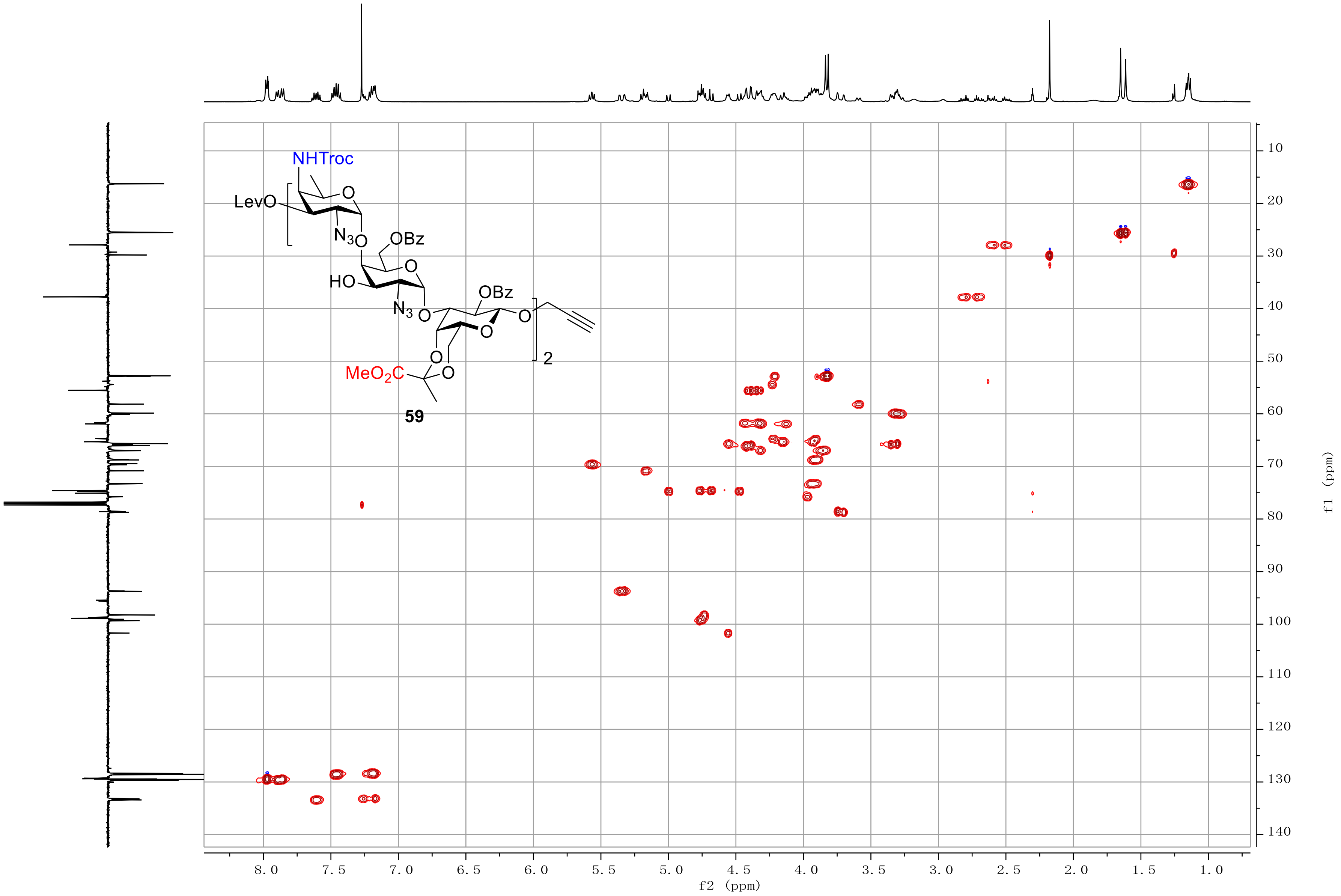

zhen2112biosyn.5.ser — wz822-A-2-s — bbo-c13-HMBC CDCl3 /opt/topspin2.1 nmrafd 13

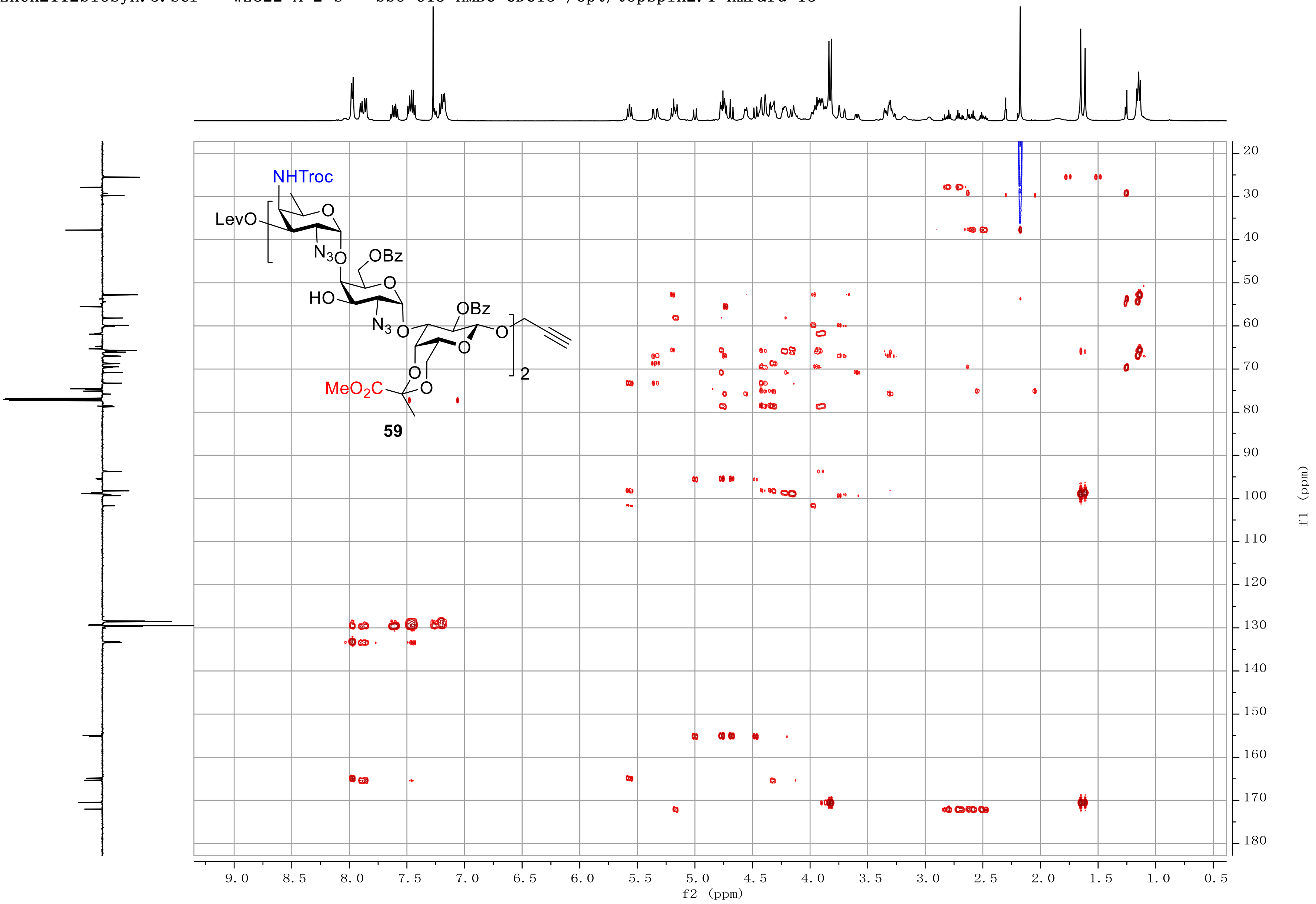

zhen2112biosyn.6.ser — wz822-A-2-s — bbo-c13-hmbc-ipv-gated CDCl3 /opt/topspin2.1 nmrafd 13

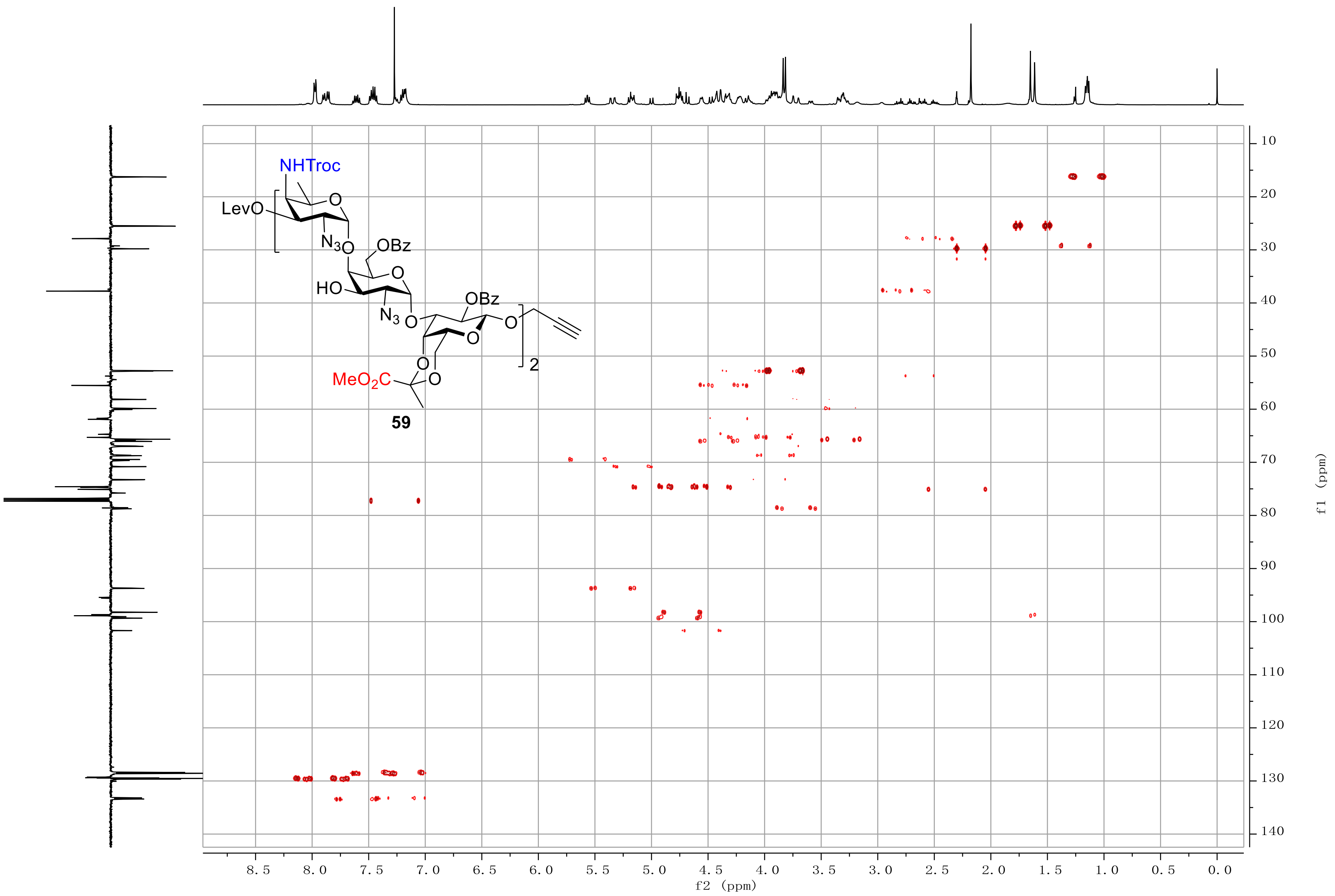

zhen2112biosyn.28.fid — wz823-A-size — bbo-h1 CDCl3 /opt/topspin2.1 nmrafd 14

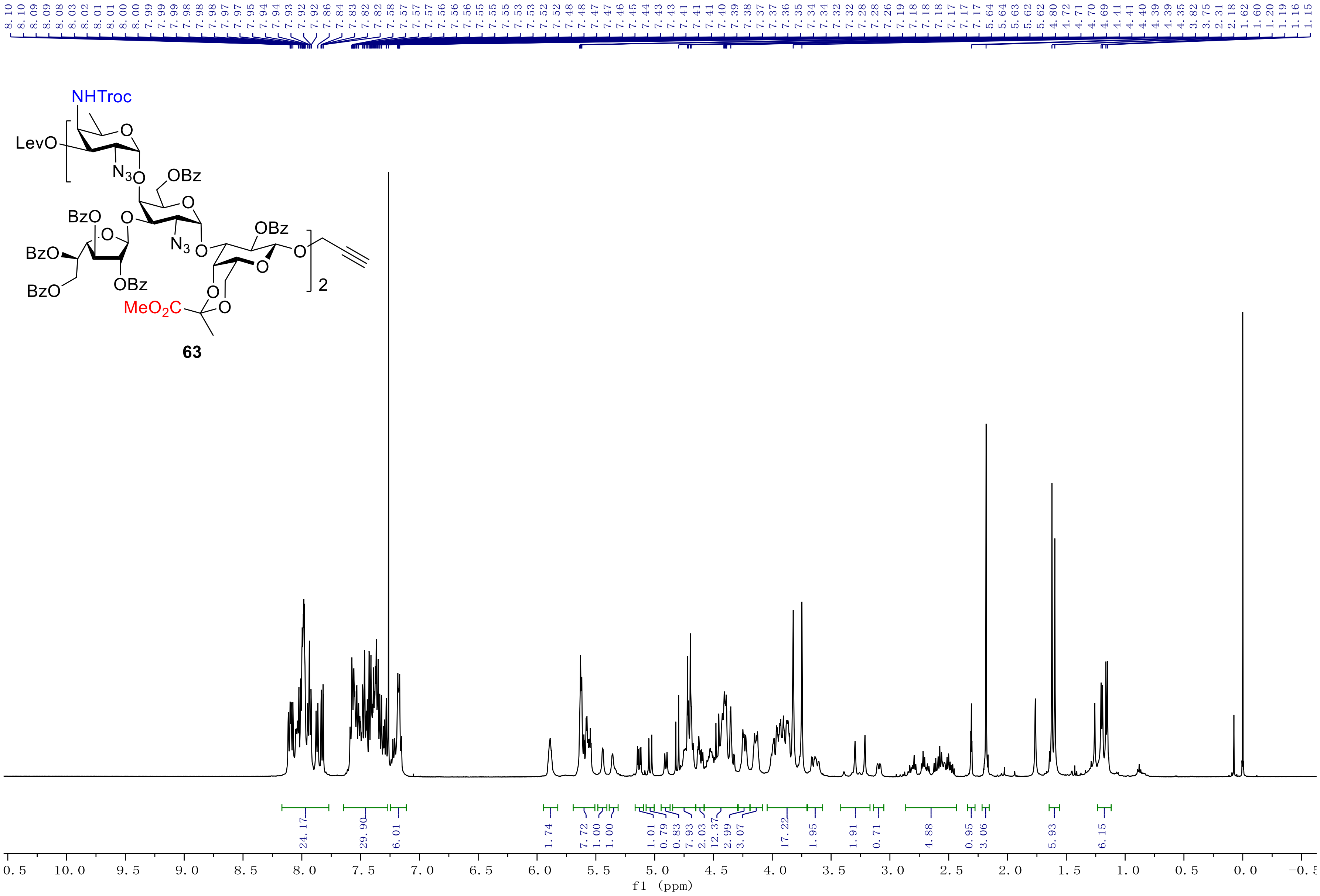

zhen2112biosyn.31.fid — wz823-A-size — bbo-c13-APT CDCl3 /opt/topspin2.1 nmrafd 14

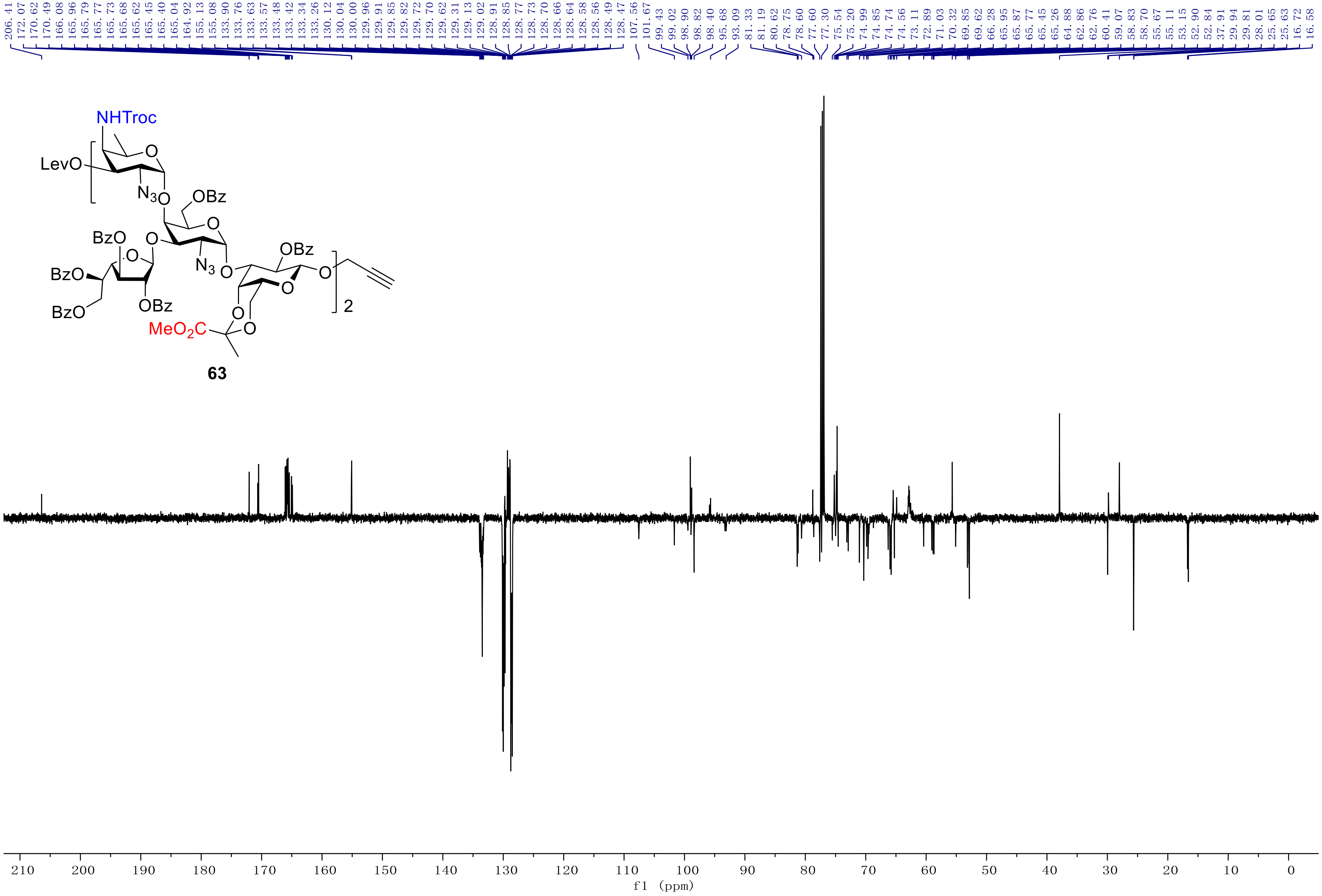

zhen2112biosyn.29.ser — wz823-A-size — bbo-h1-cosy CDCl3 /opt/topspin2.1 nmrafd 14

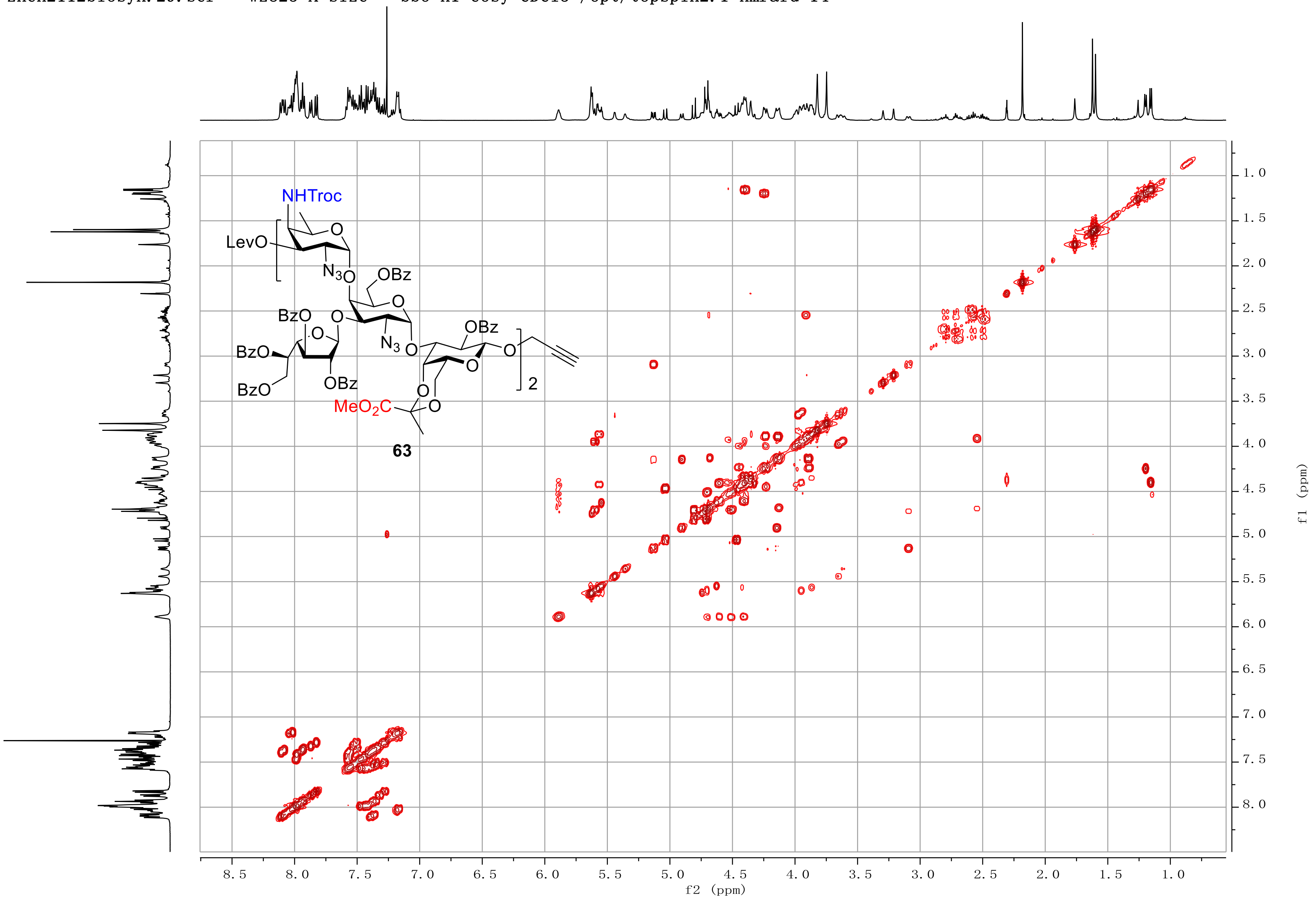

zhen2112biosyn.30.ser — wz823-A-size — bbo-c13-HSQC CDCl3 /opt/topspin2.1 nmrafd 14

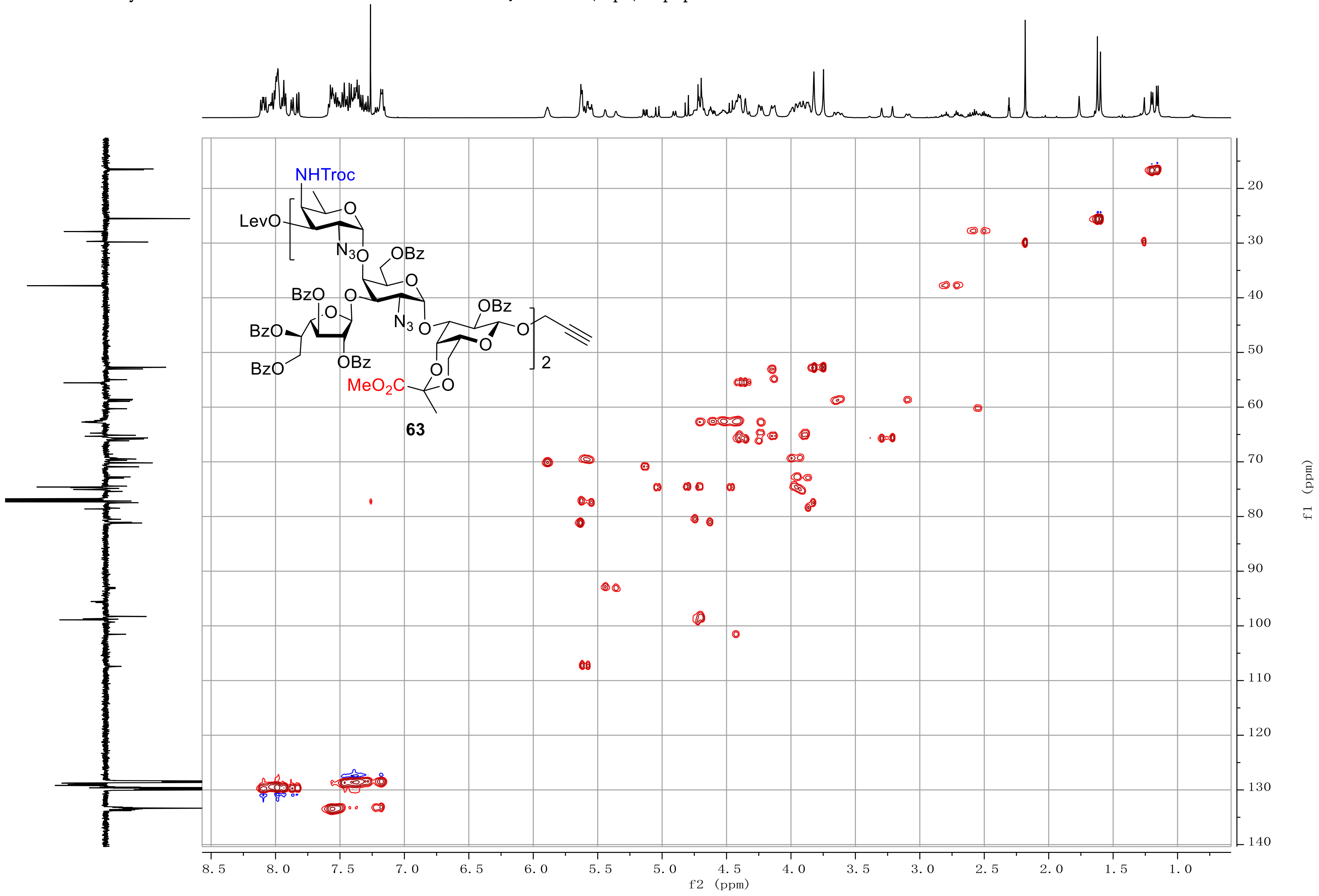

zhen2112biosyn.32.ser — wz823-A-size — bbo-c13-HMBC CDCl3 /opt/topspin2.1 nmrafd 14

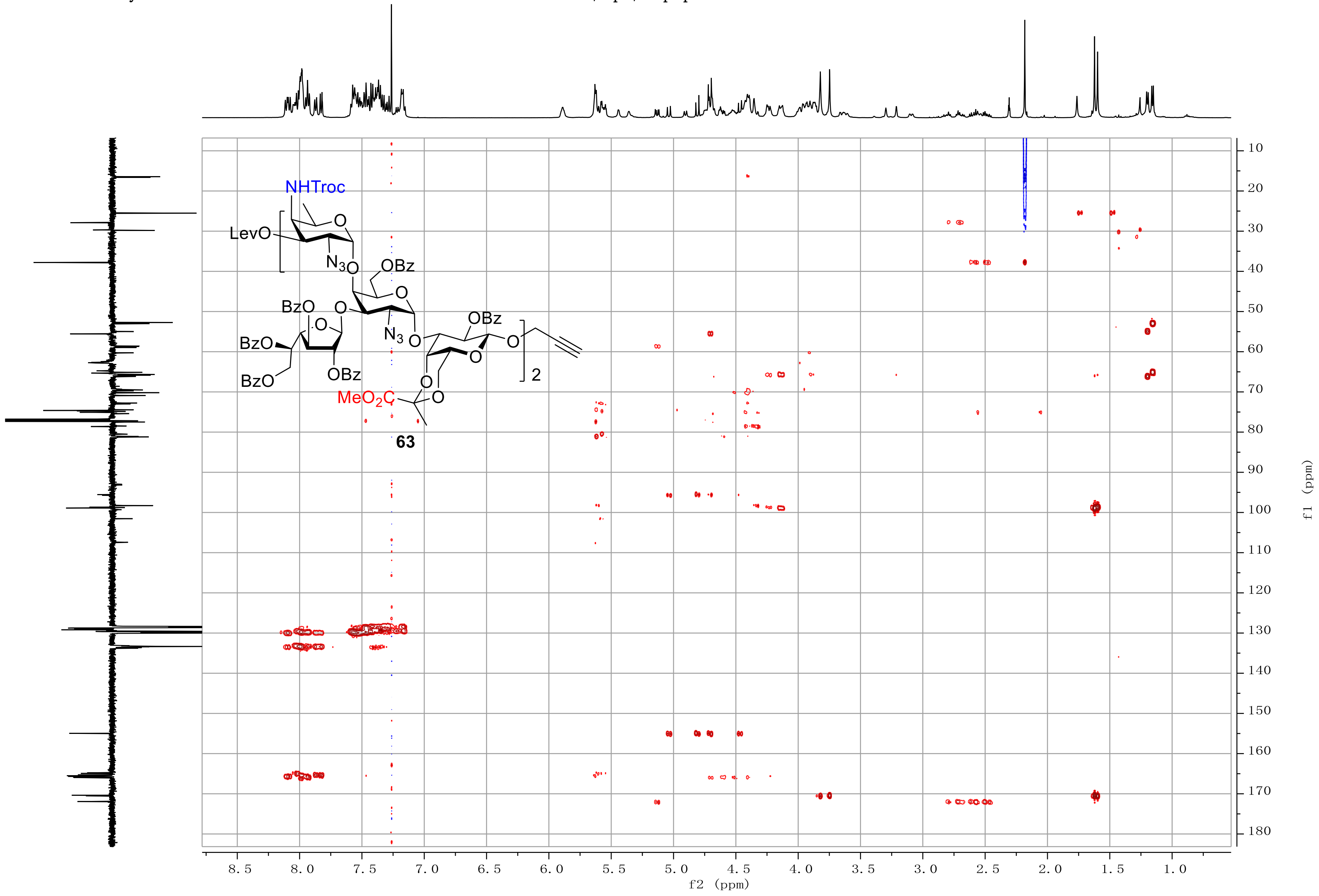

zhen2112biosyn.33.ser — wz823-A-size — bbo-c13-hmbc-ipv-gated CDCl3 /opt/topspin2.1 nmrafd 14

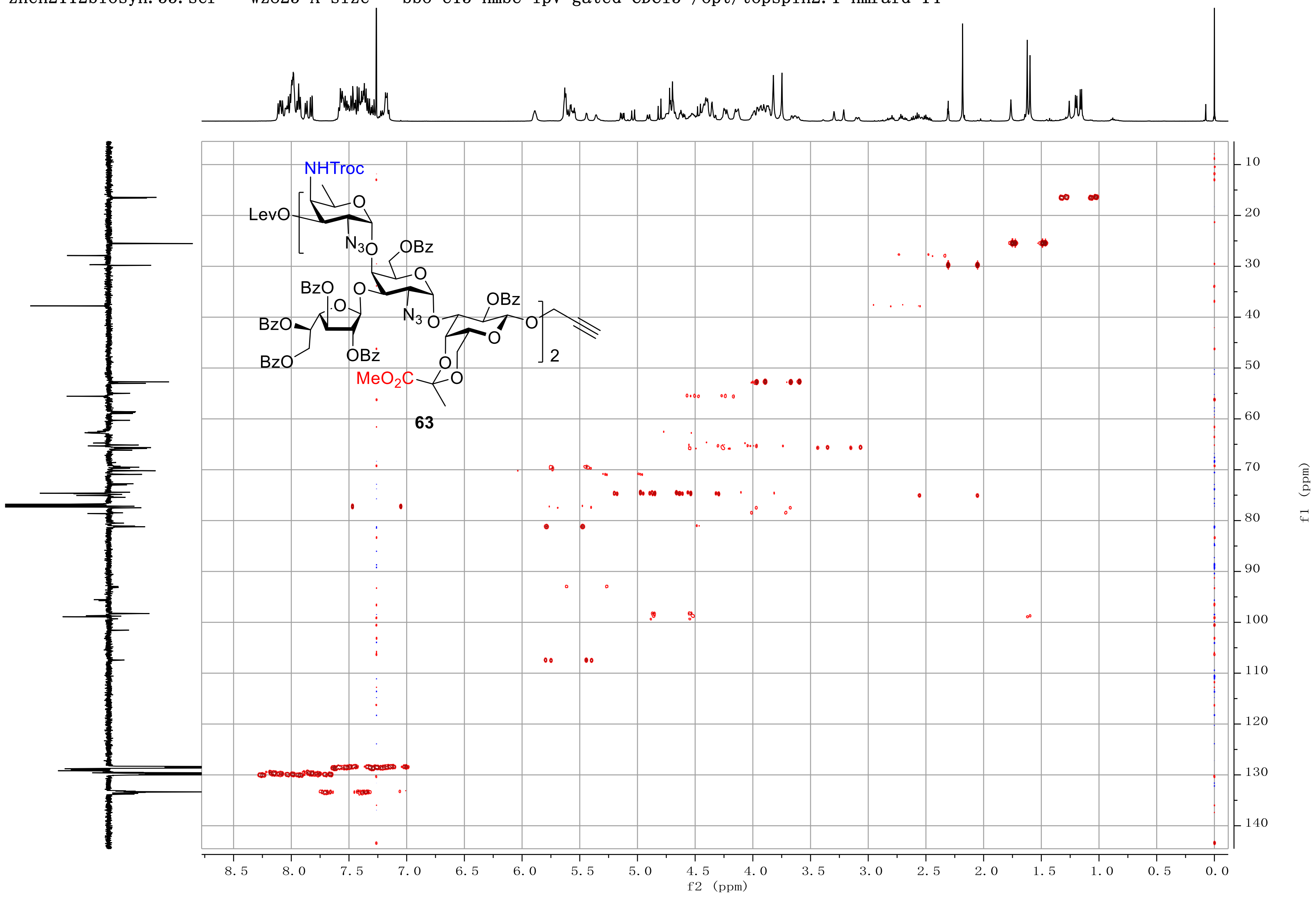

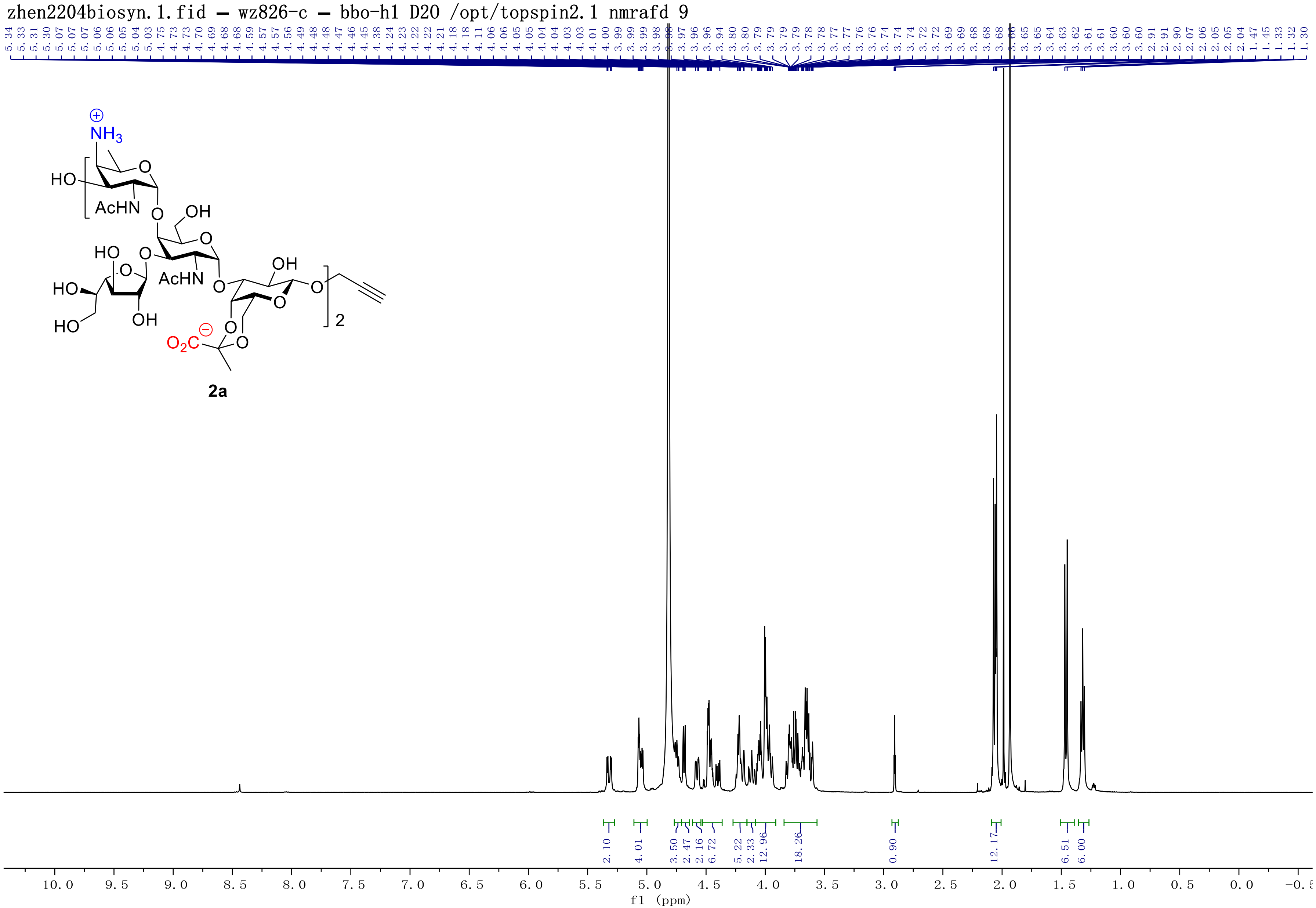
zhen2204biosyn.1.fid — wz826-c — bbo-h1 D2O /opt/topspin2.1 nmrafd 9
NH3
HO
AcHN
OH
HO
AcHN
OH
HO
HO
OH
O2C
2
2a
2.10
4.01
3.50
2.47
2.16
6.72
5.22
2.33
12.96
18.26
0.90
12.17
6.51
6.00
10.0 9.5 9.0 8.5 8.0 7.5 7.0 6.5 6.0 5.5 5.0 4.5 4.0 3.5 3.0 2.5 2.0 1.5 1.0 0.5 0.0
f1 (ppm)

zhen2204biosyn.4.fid — wz826-c — bbo-c13-APT D2O /opt/topspin2.1 nmrafd 9

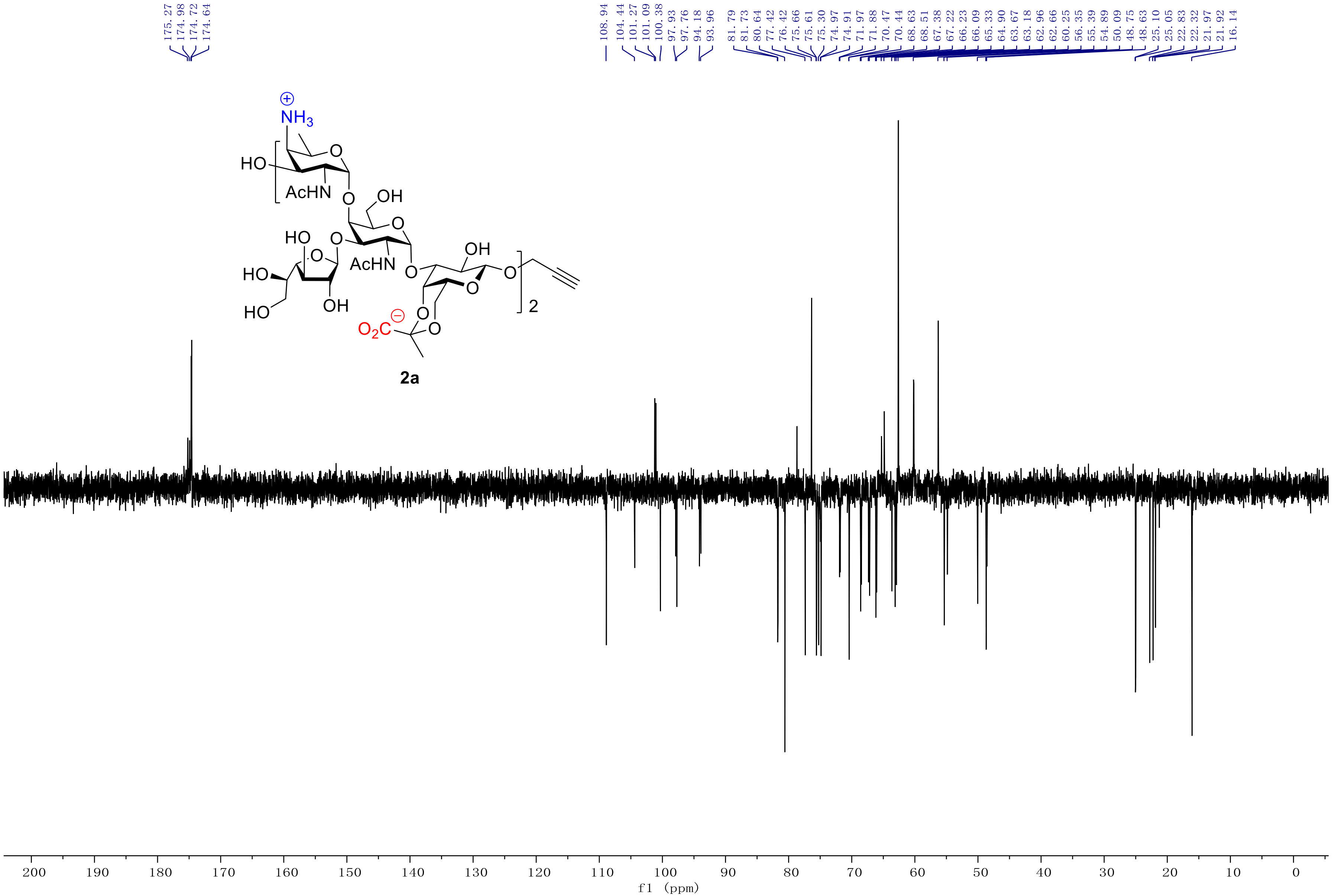

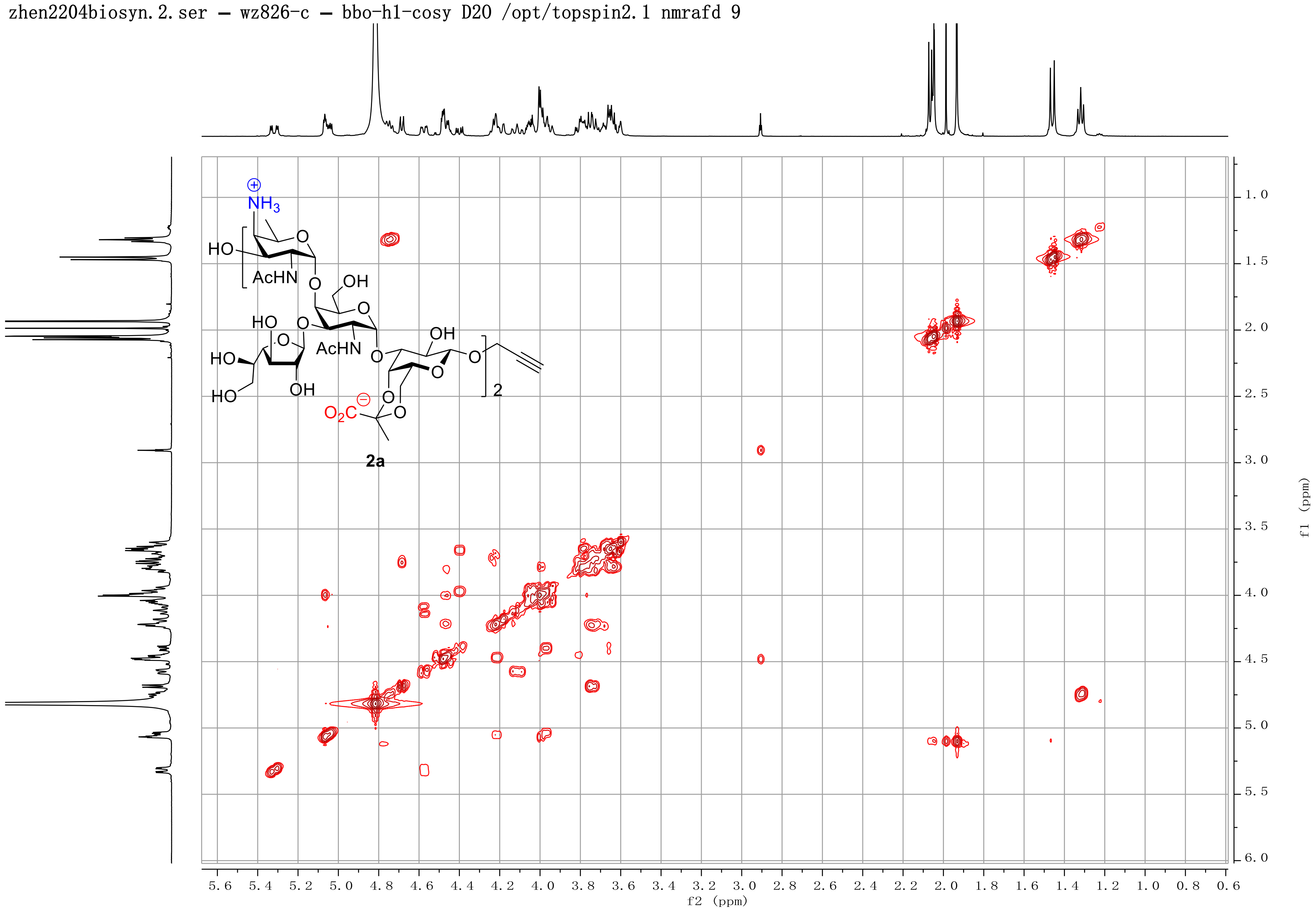

zhen2204biosyn.2.ser — wz826-c — bbo-h1-cosy D2O /opt/topspin2.1 nmrafd 9
2a
f2 (ppm)
f1 (ppm)

zhen2204biosyn.3.ser — wz826-c — bbo-c13-HSQC D2O /opt/topspin2.1 nmrafd 9

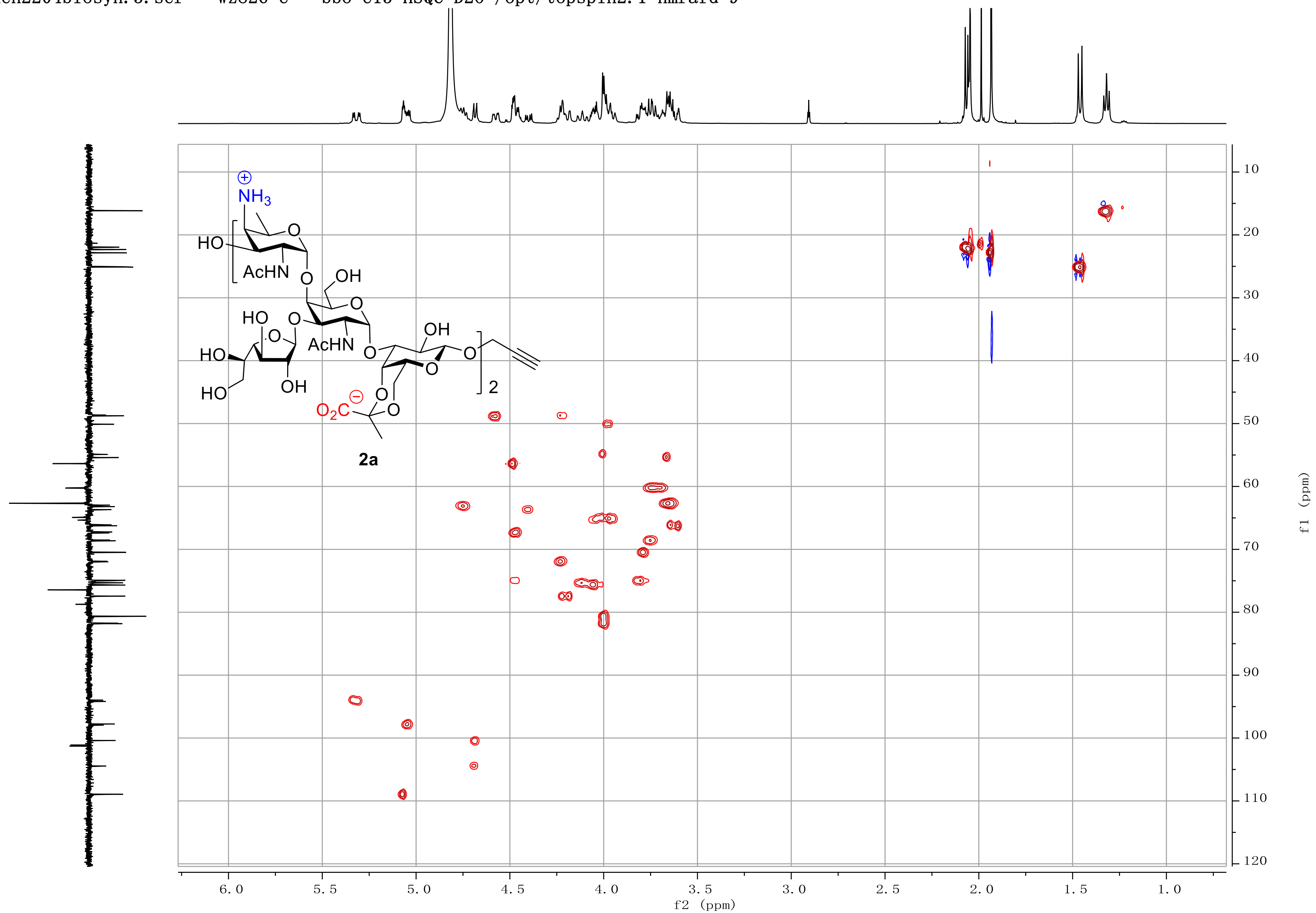

zhen2204biosyn.5.ser — wz826-c — bbo-c13-HMBC D2O /opt/topspin2.1 nmrafd 9

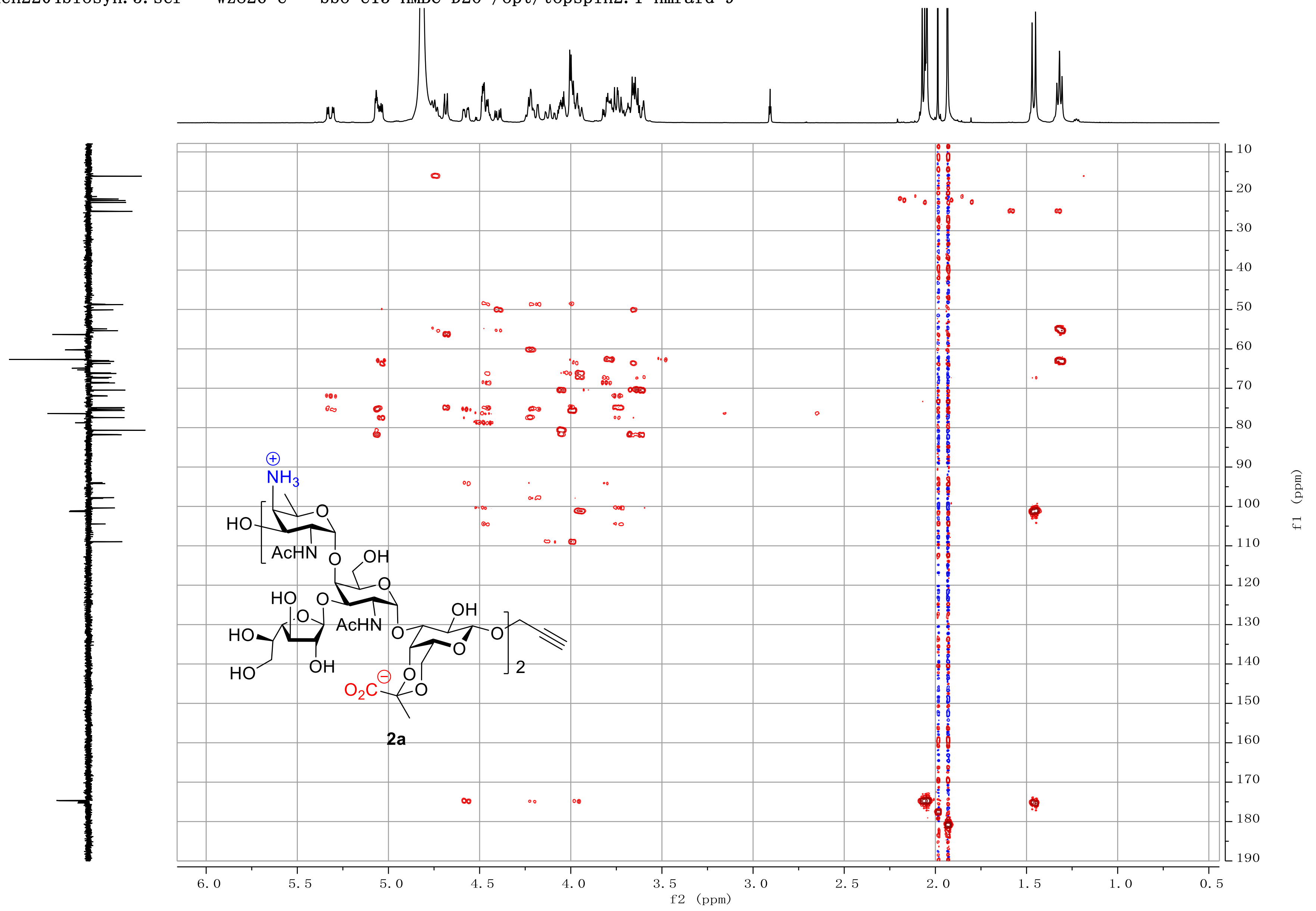

zhen2110biosyn.88.fid — wz827-a — bbo-h1 CDCl3 /opt/topspin2.1 nmrafd 1

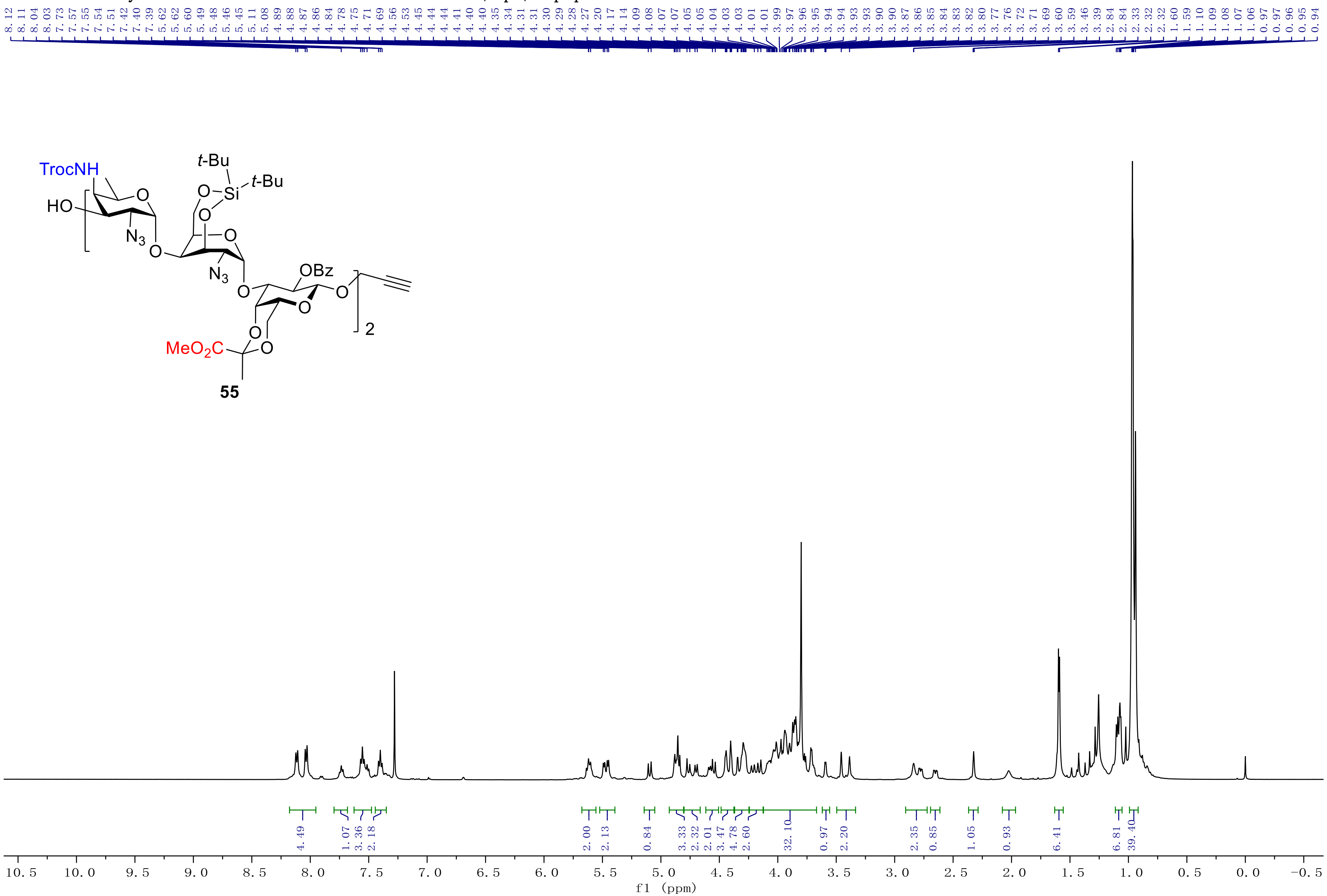

zhen2110biosyn.91.fid — wz827-a — bbo-c13-APT CDCl3 /opt/topspin2.1 nmrafd 1

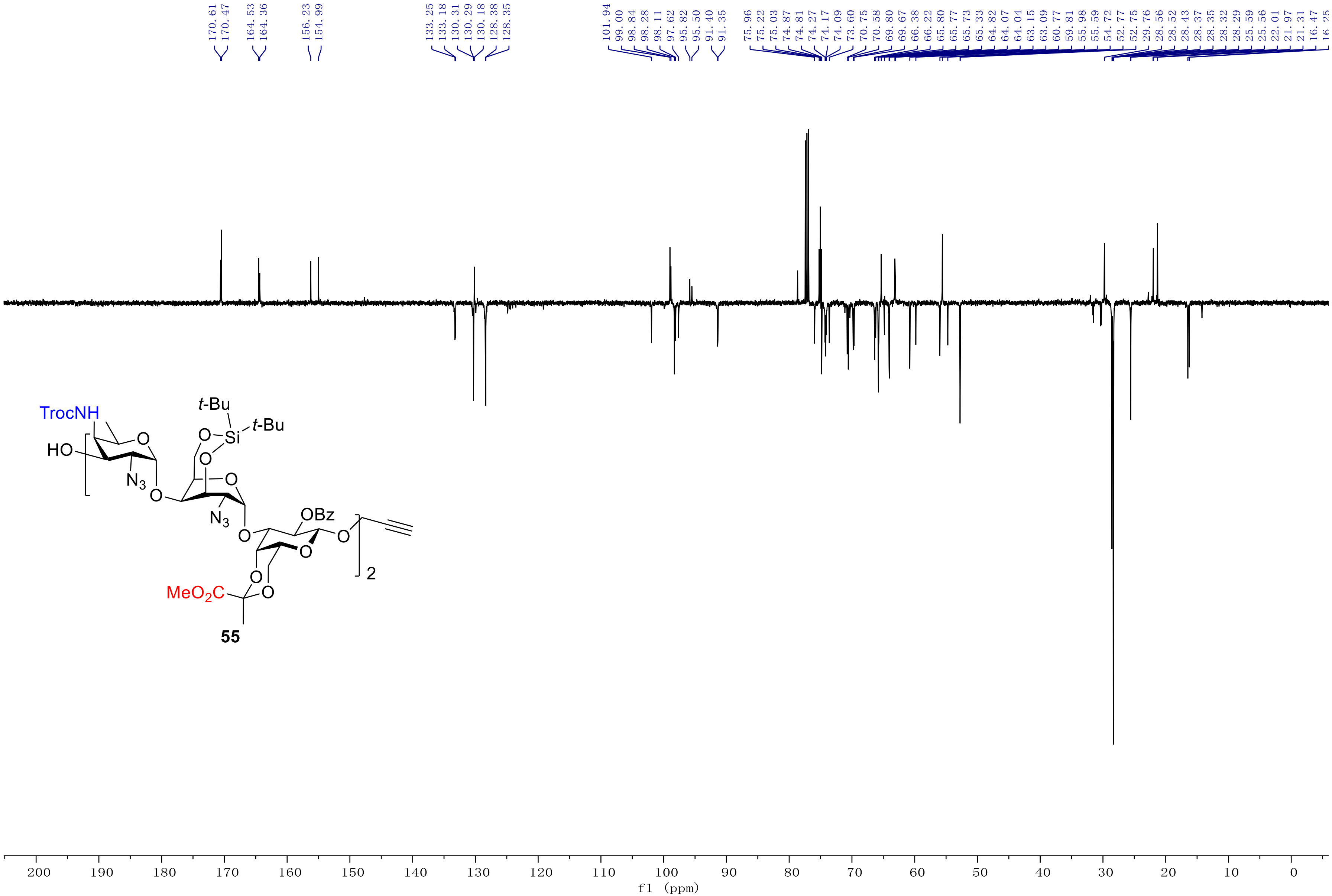

55

zhen2110biosyn.89.ser — wz827-a — bbo-h1-cosy CDCl3 /opt/topspin2.1 nmrafd 1

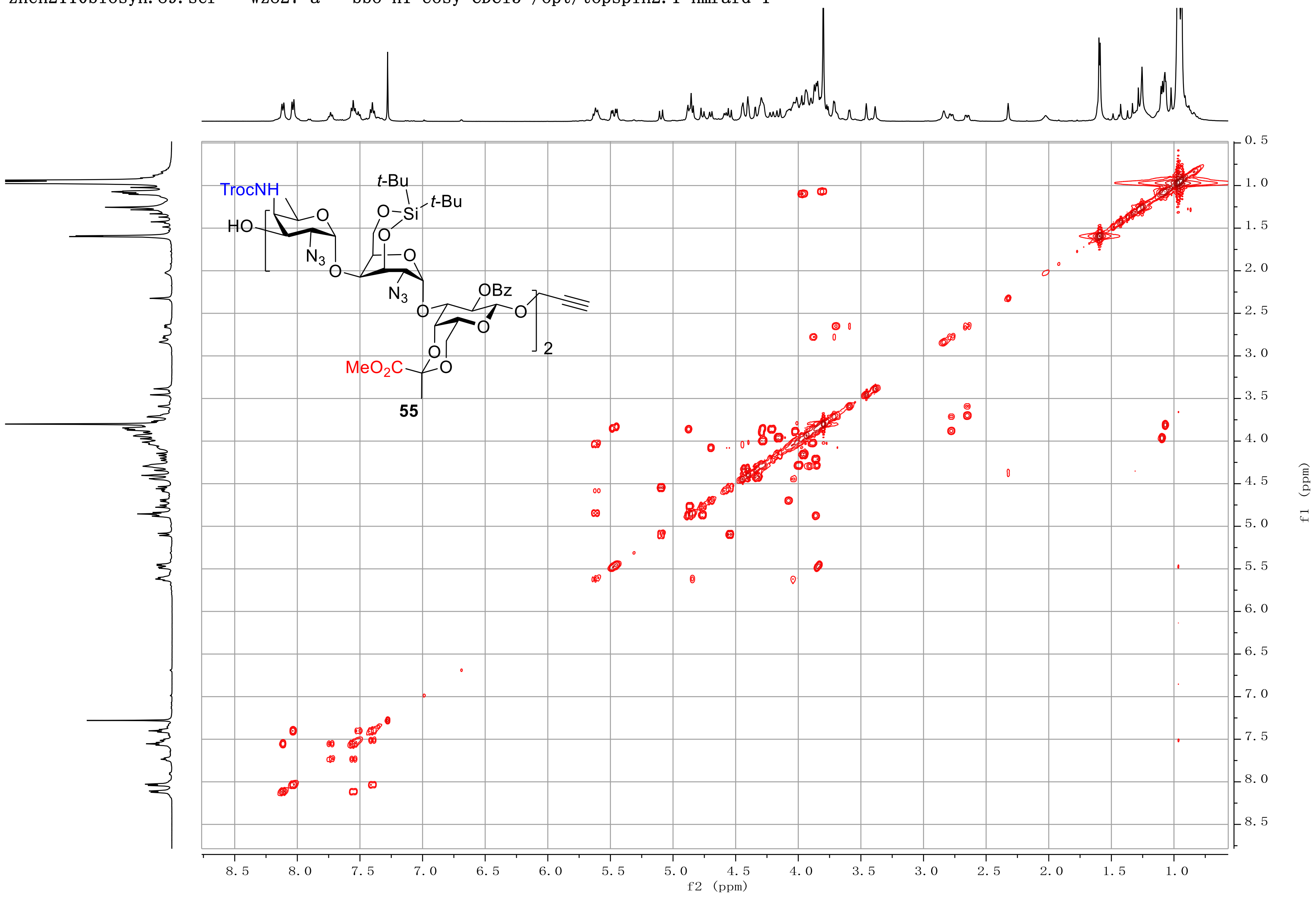

zhen2110biosyn.90.ser — wz827-a — bbo-c13-HSQC CDCl3 /opt/topspin2.1 nmrafd 1

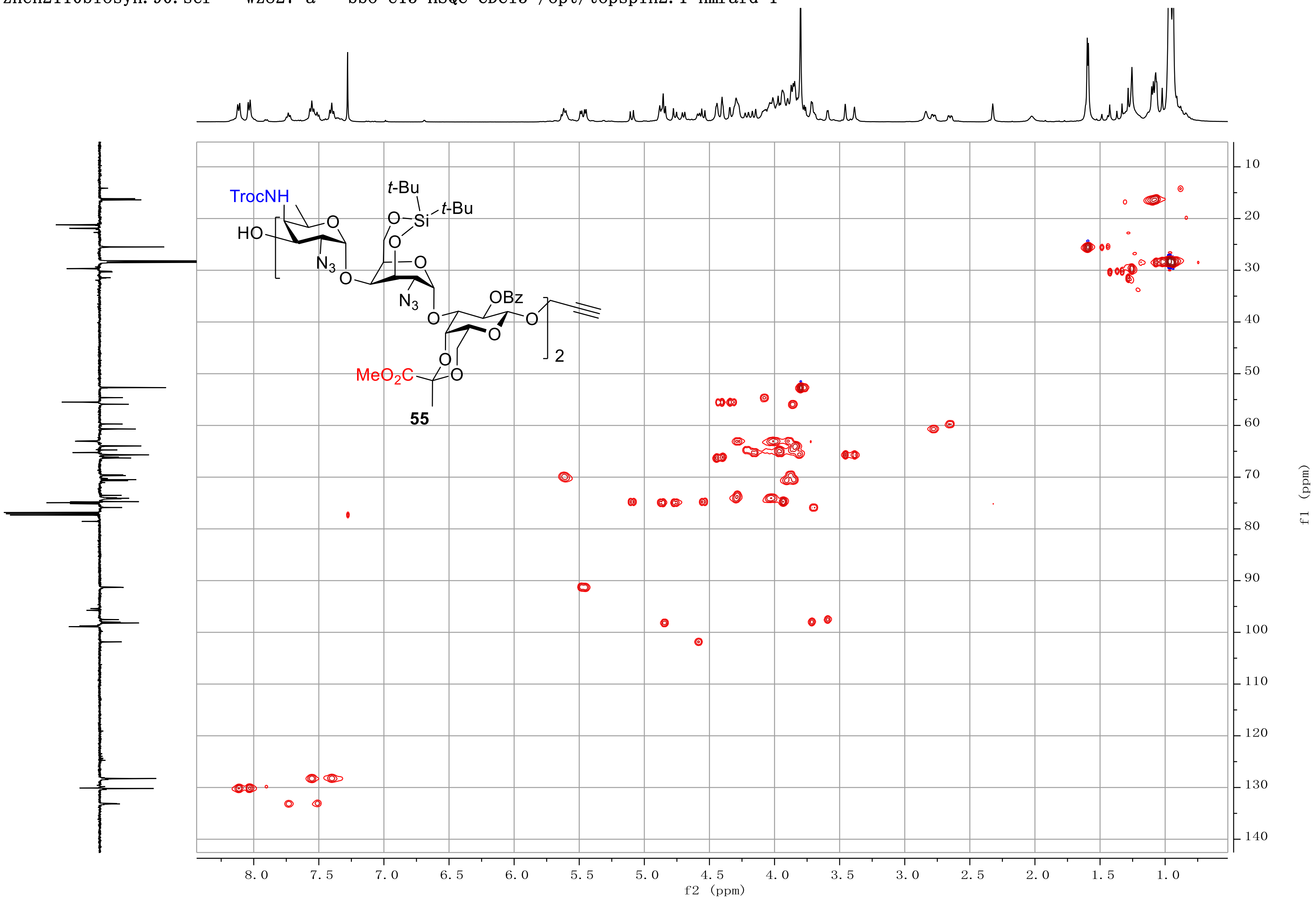

zhen2110biosyn.92.ser — wz827-a — bbo-c13-HMBC CDCl3 /opt/topspin2.1 nmrafd 1

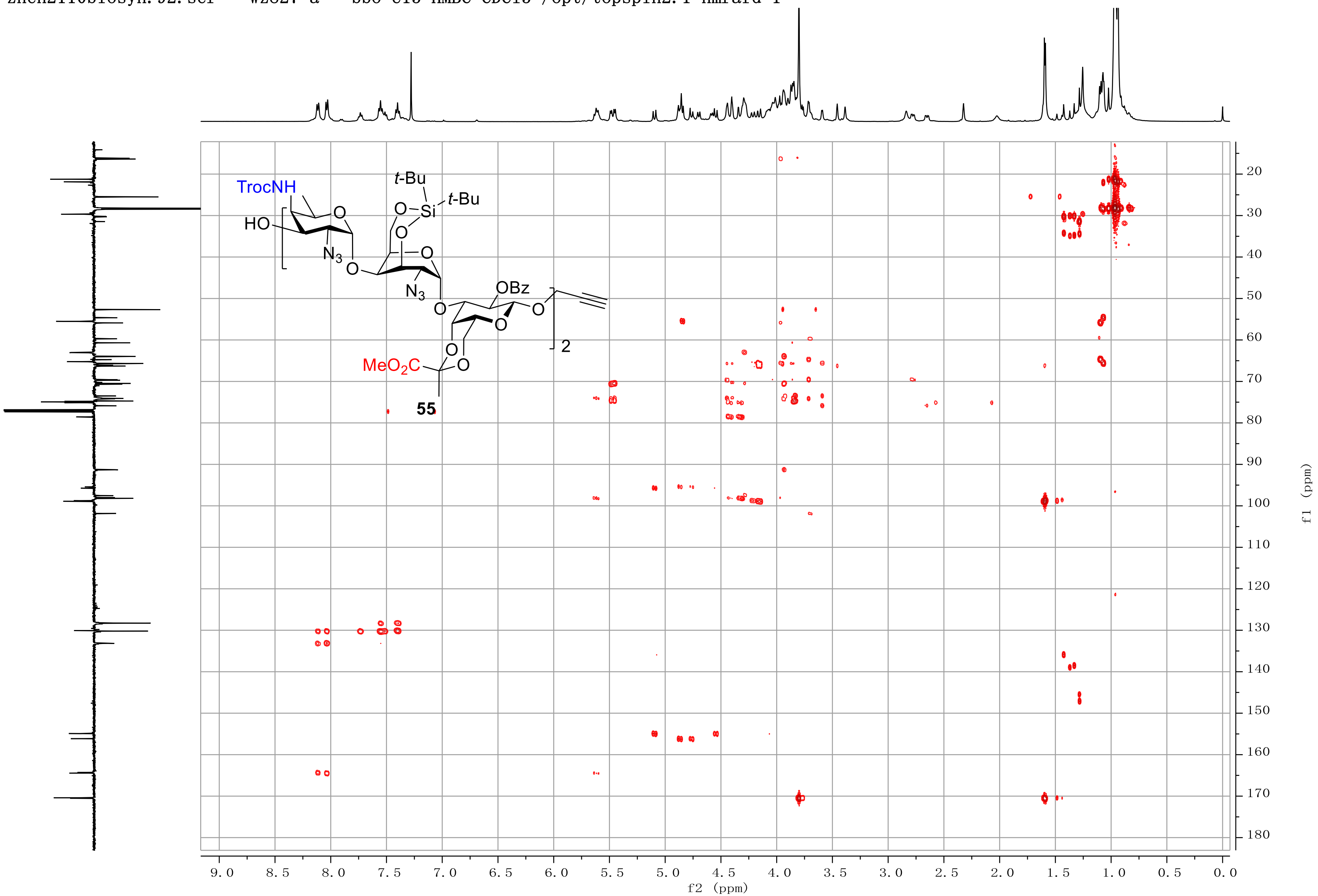

zhen2110biosyn.93.ser — wz827-a — bbo-c13-hmbc-ipv-gated CDCl3 /opt/topspin2.1 nmrafd 1

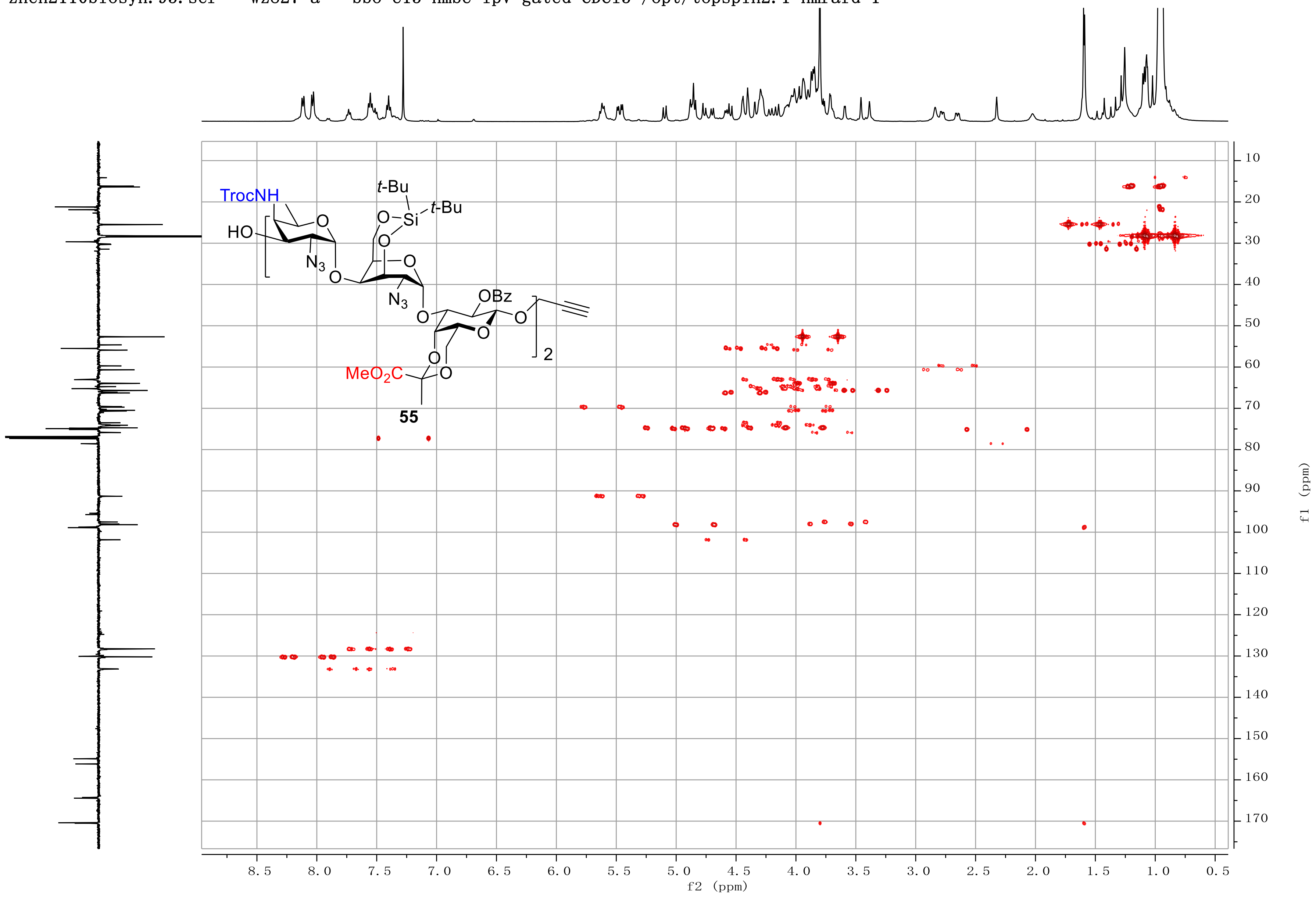

2110zhen.30.fid — wz828-A-s — h1 CDCl3 /opt/DATA nmrafd 19

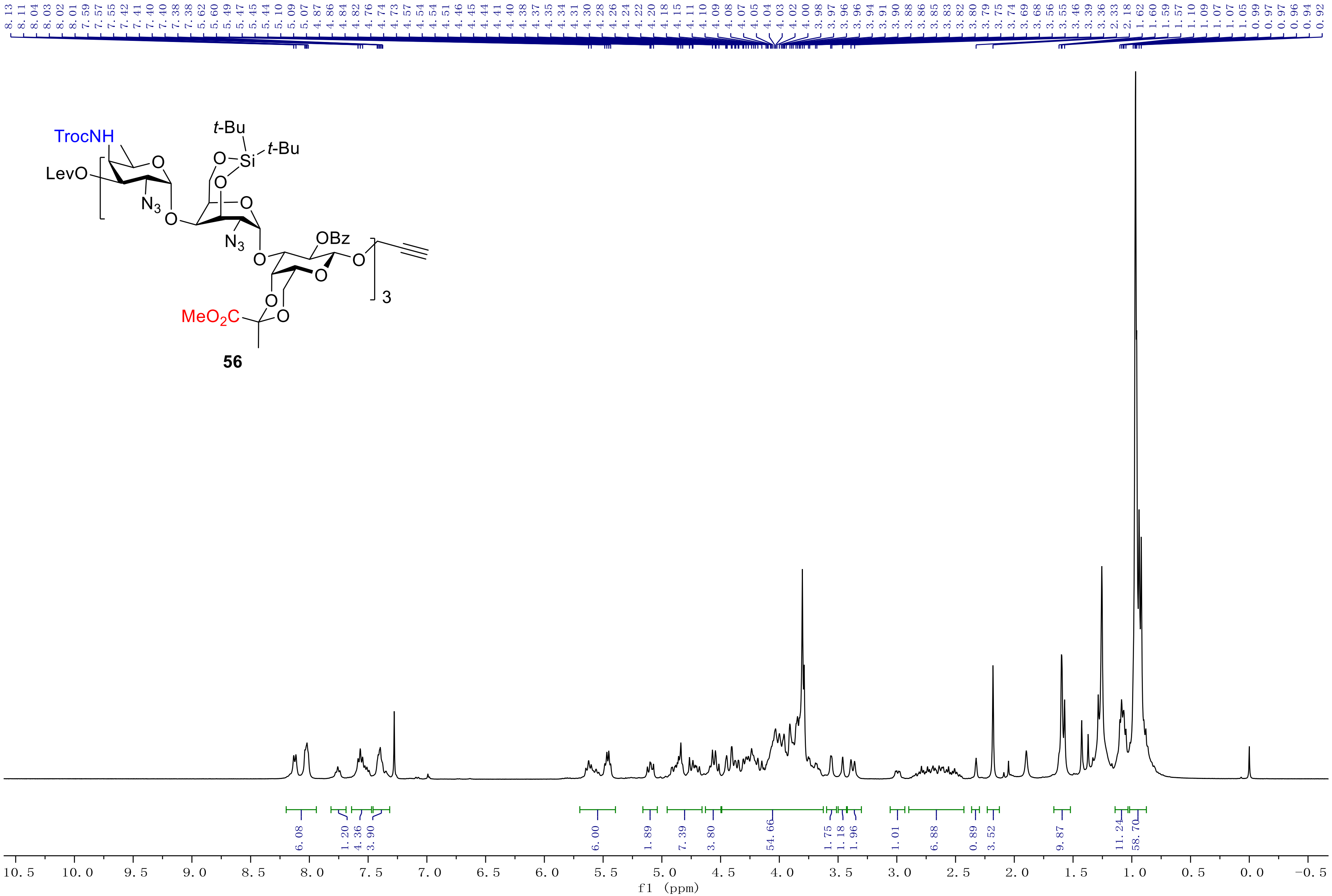

56

2110zhen.31.fid — wz828-A-s — C13APT CDCl3 /opt/DATA nmrafd 19

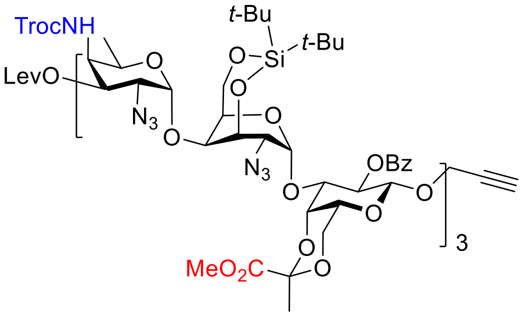

56

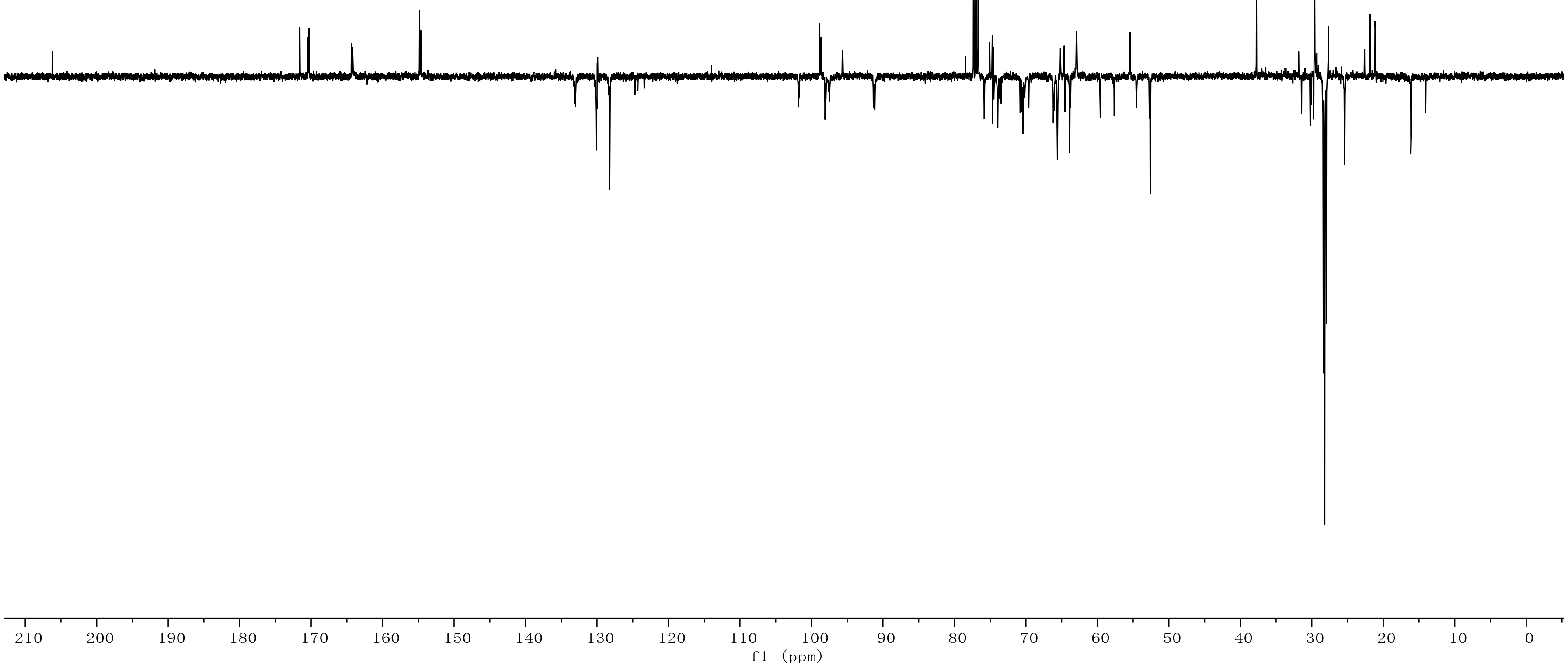

2110zhen.32.ser — wz828-A-s — h1COSY CDCl3 /opt/DATA nmrafd 19

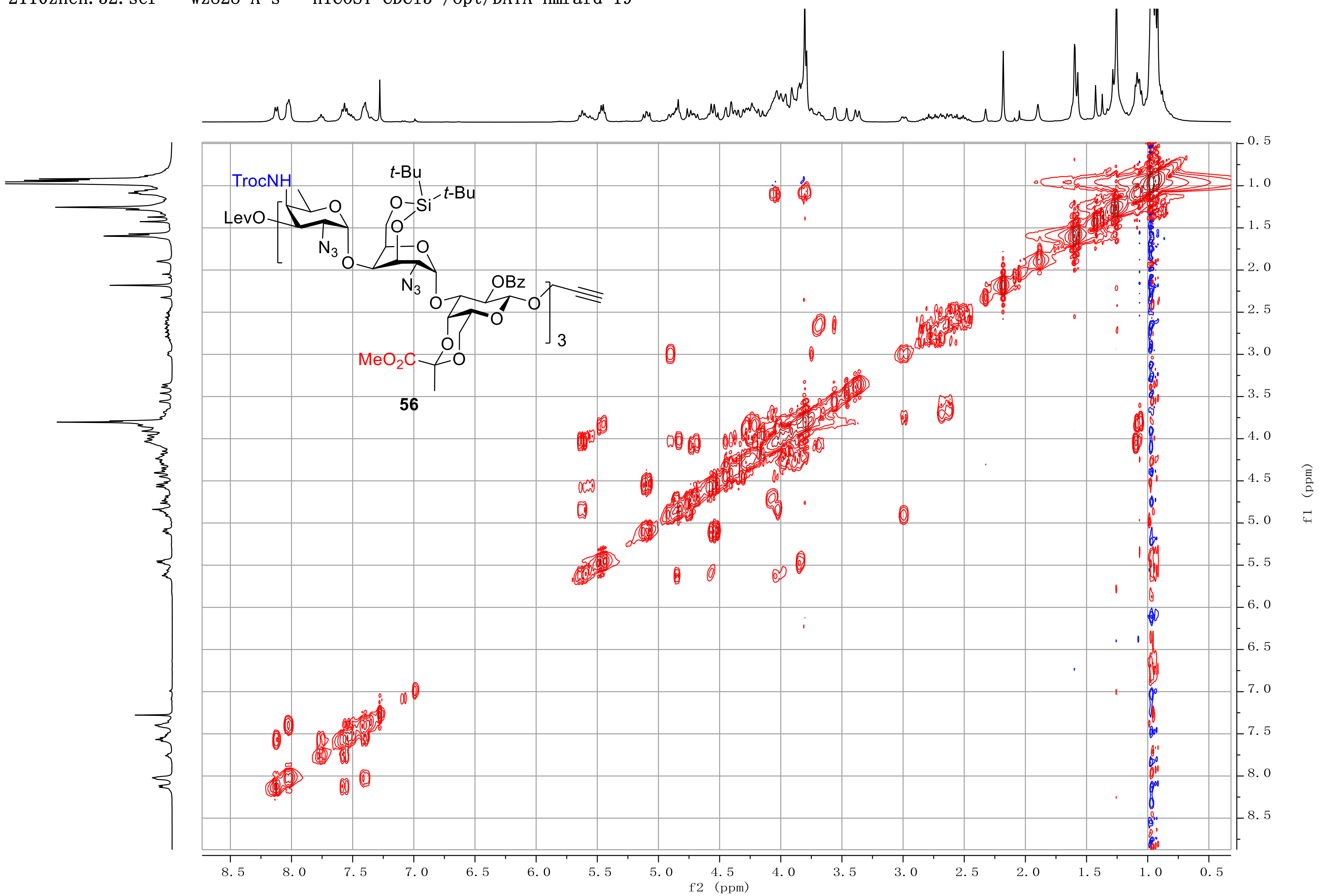

2110zhen.33.ser — wz828-A-s — c13HSQC CDCl3 /opt/DATA nmrafd 19

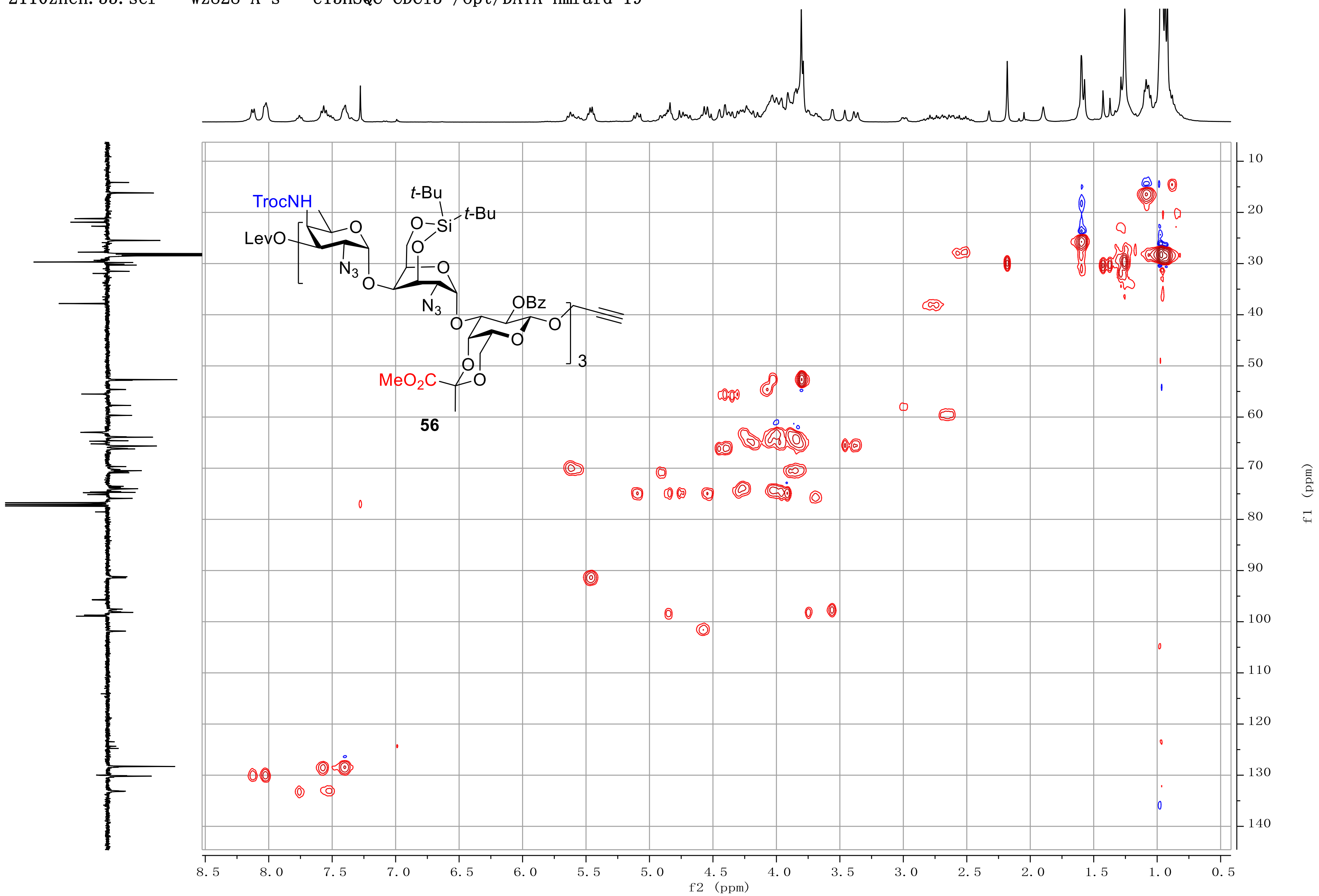

2202zhen.1.fid — wz842-B — h1 CDCl3 /opt/DATA nmrafd 18

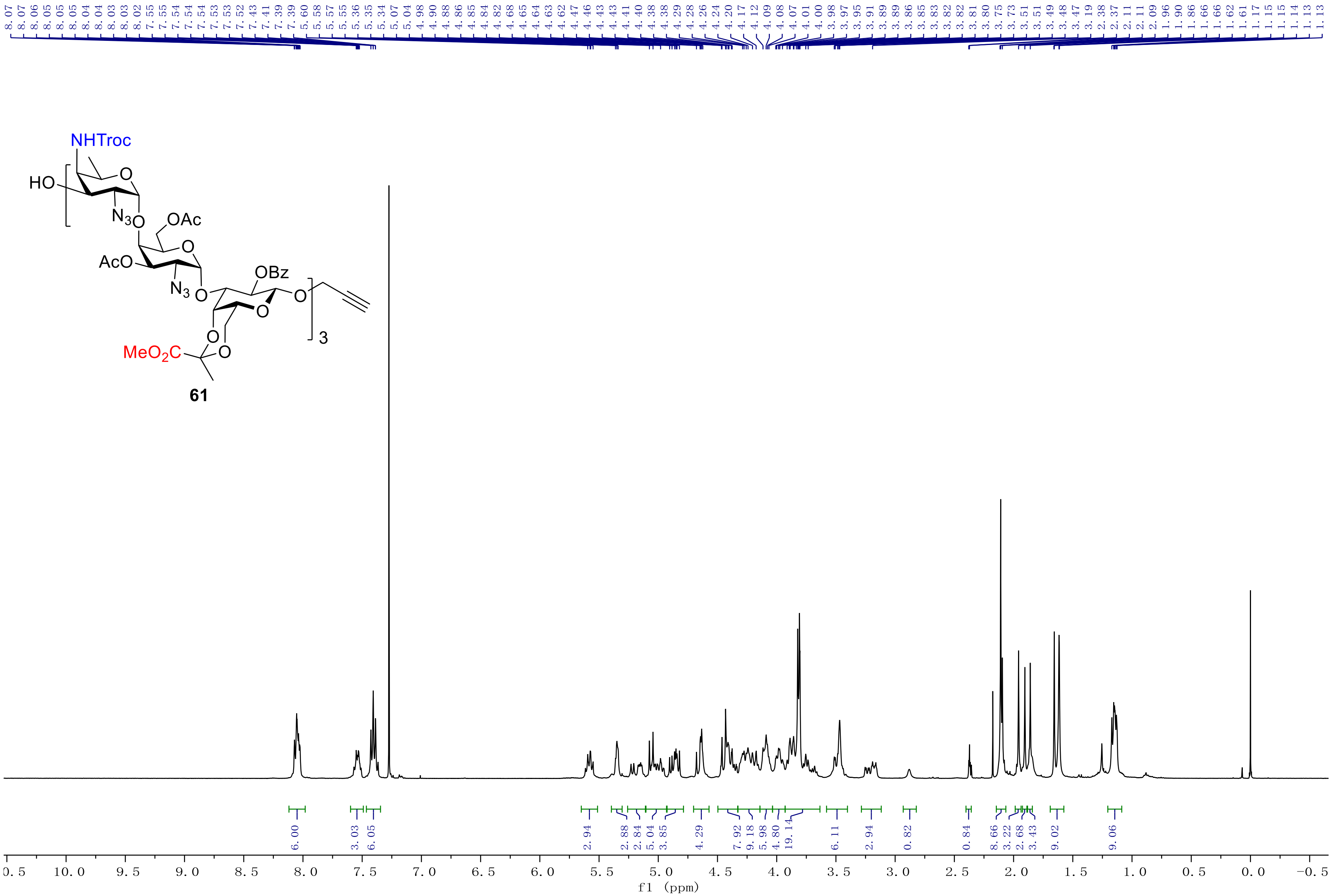

2202zhen.2.fid — wz842-B — C13APT CDCl3 /opt/DATA nmrafd 18

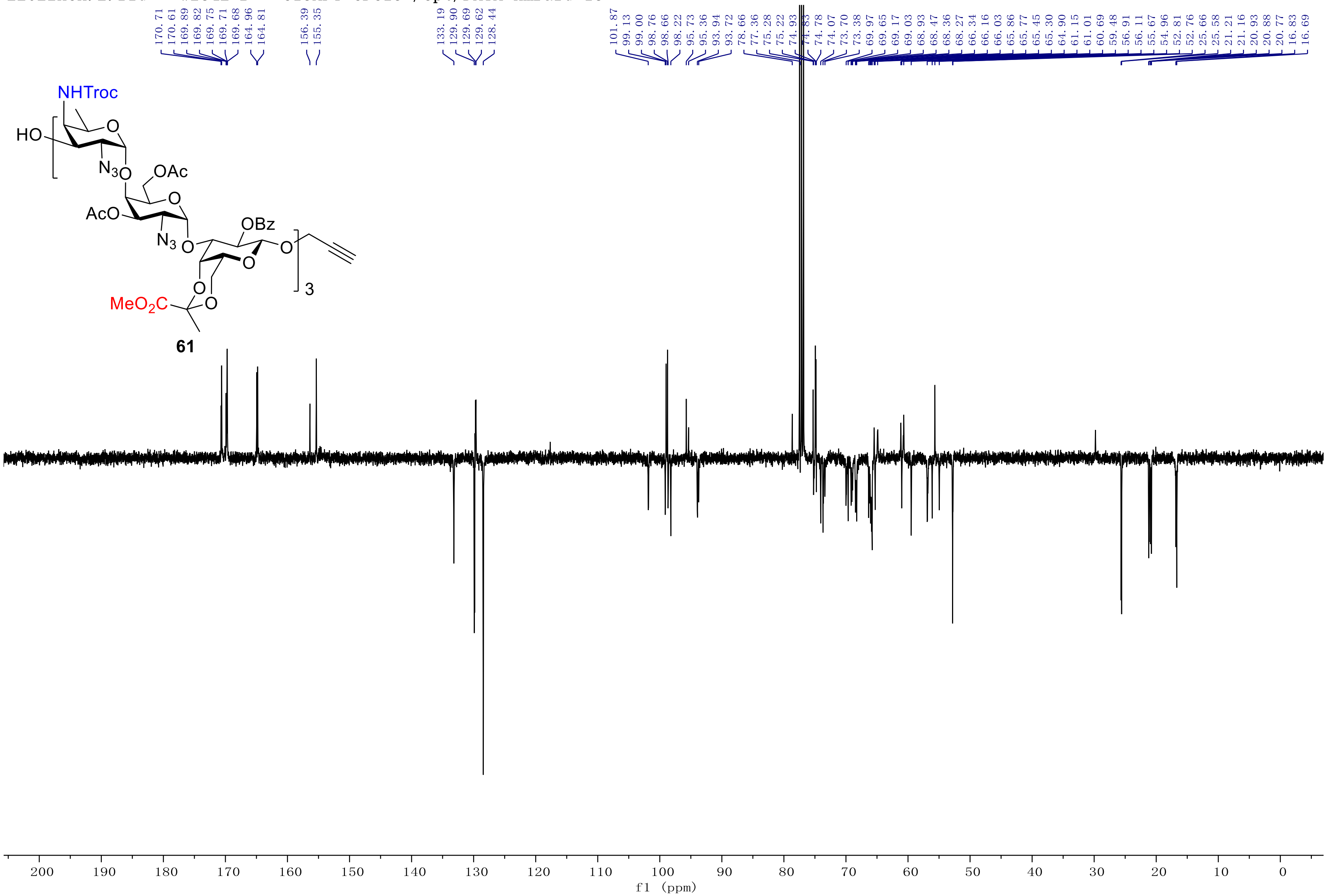

2202zhen.3.ser — wz842-B — h1COSY CDCl3 /opt/DATA nmrafd 18

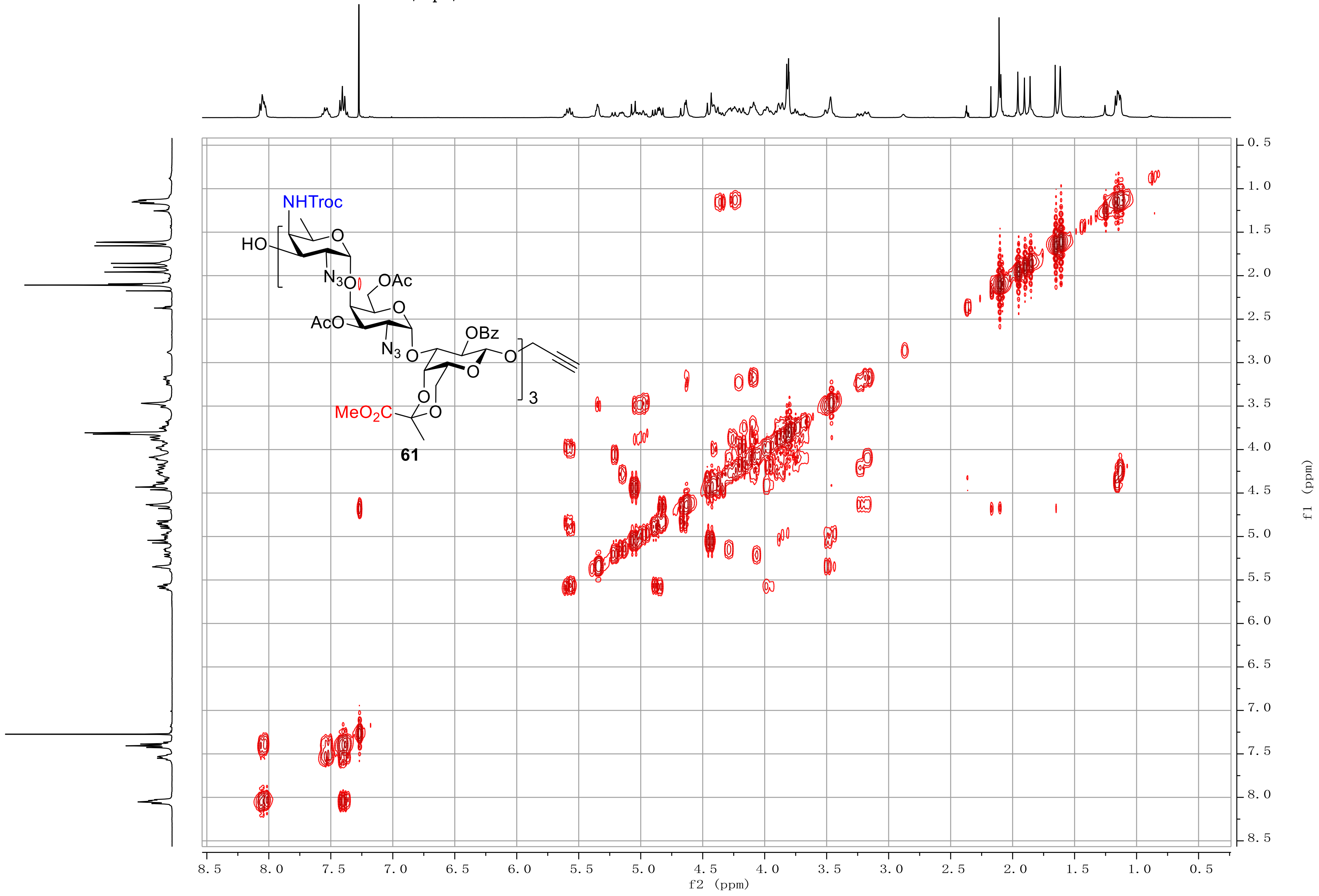

2202zhen.4.ser — wz842-B — c13HSQC CDCl3 /opt/DATA nmrafd 18

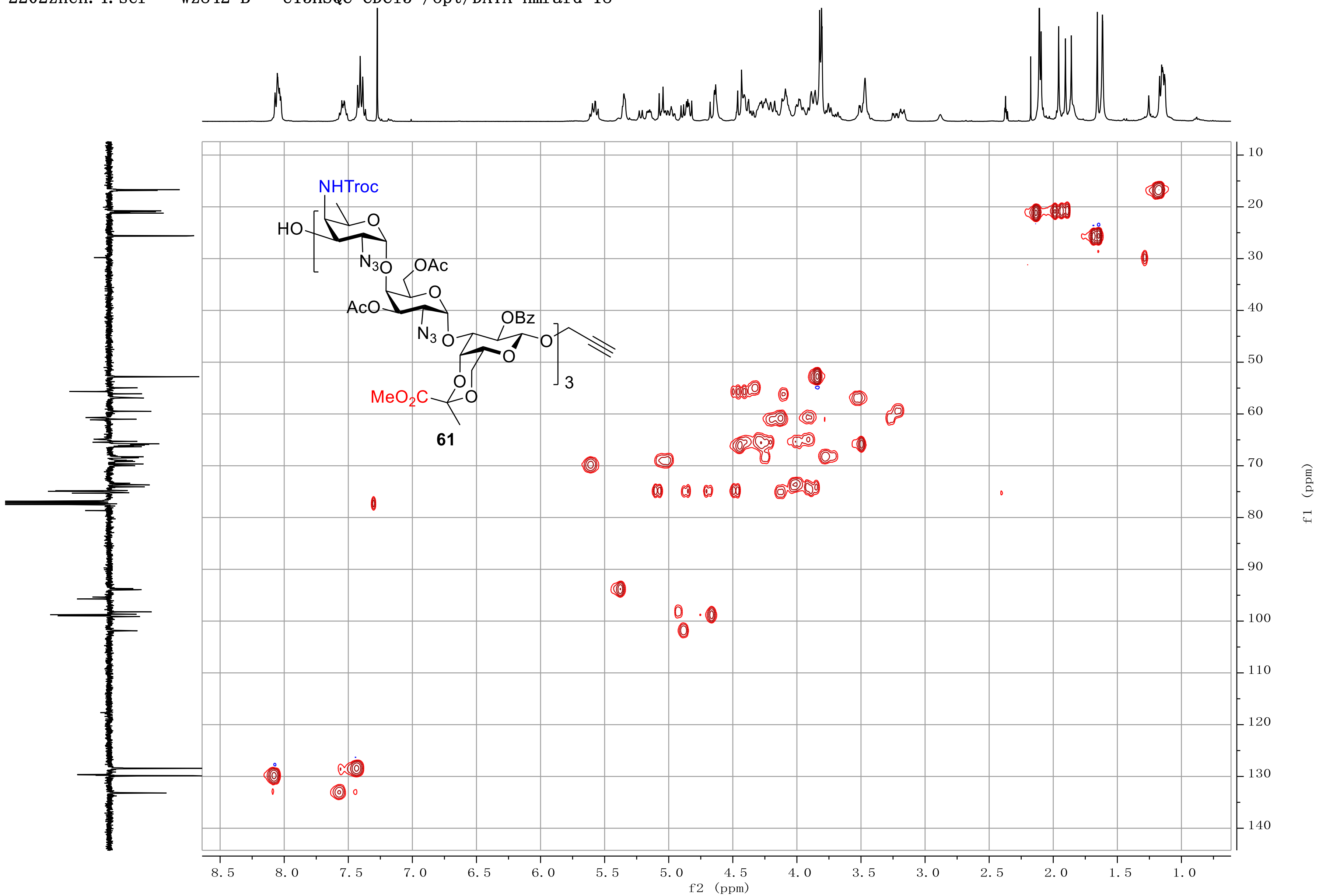

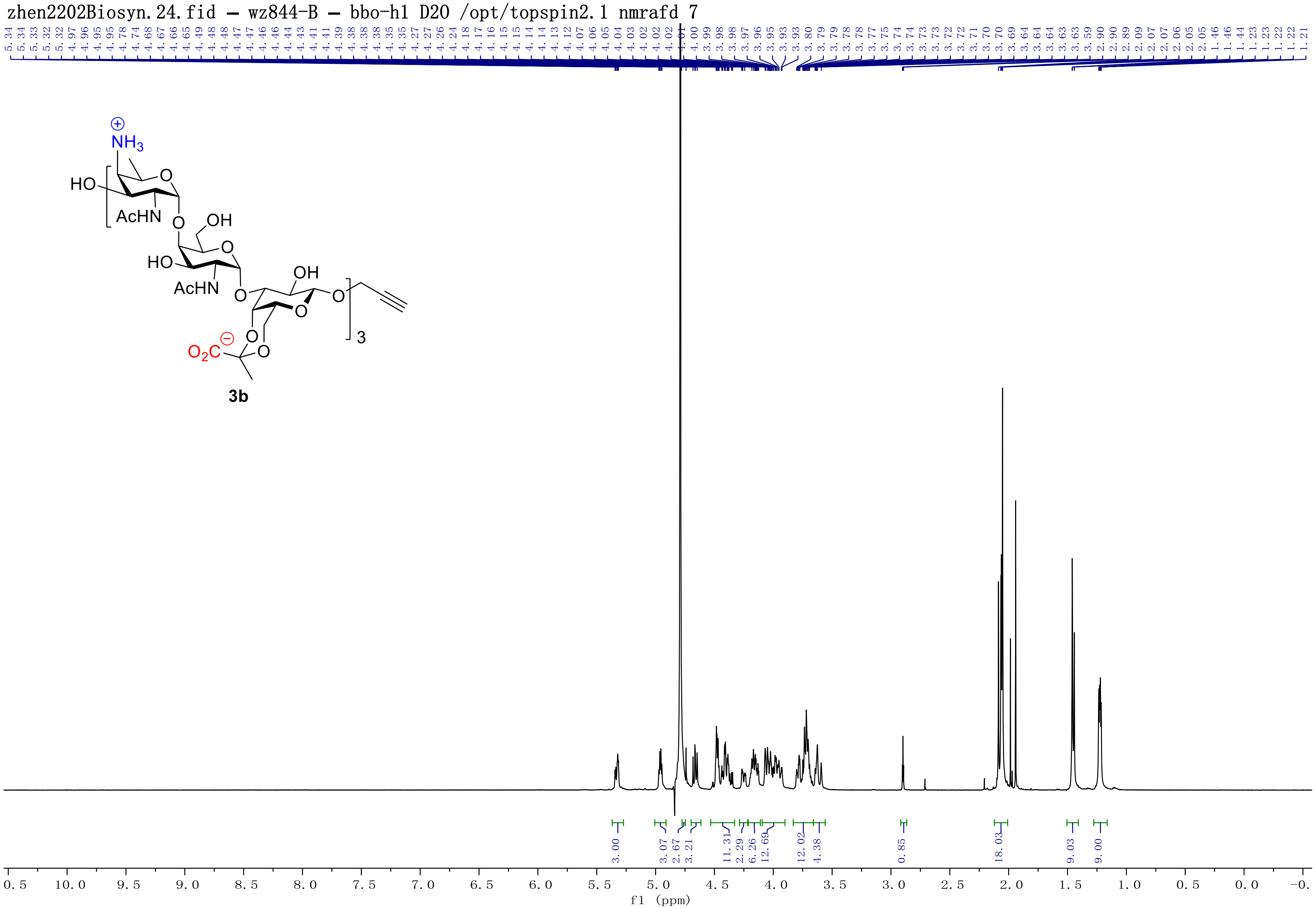
zhen2202Biosyn.24.fid — wz844-B — bbo-h1 D2O /opt/topspin2.1 nmrafd 7
3b
f1 (ppm)

zhen2202Biosyn.27.fid — wz844-B — bbo-c13-APT D20 /opt/topspin2.1 nmrafd 7

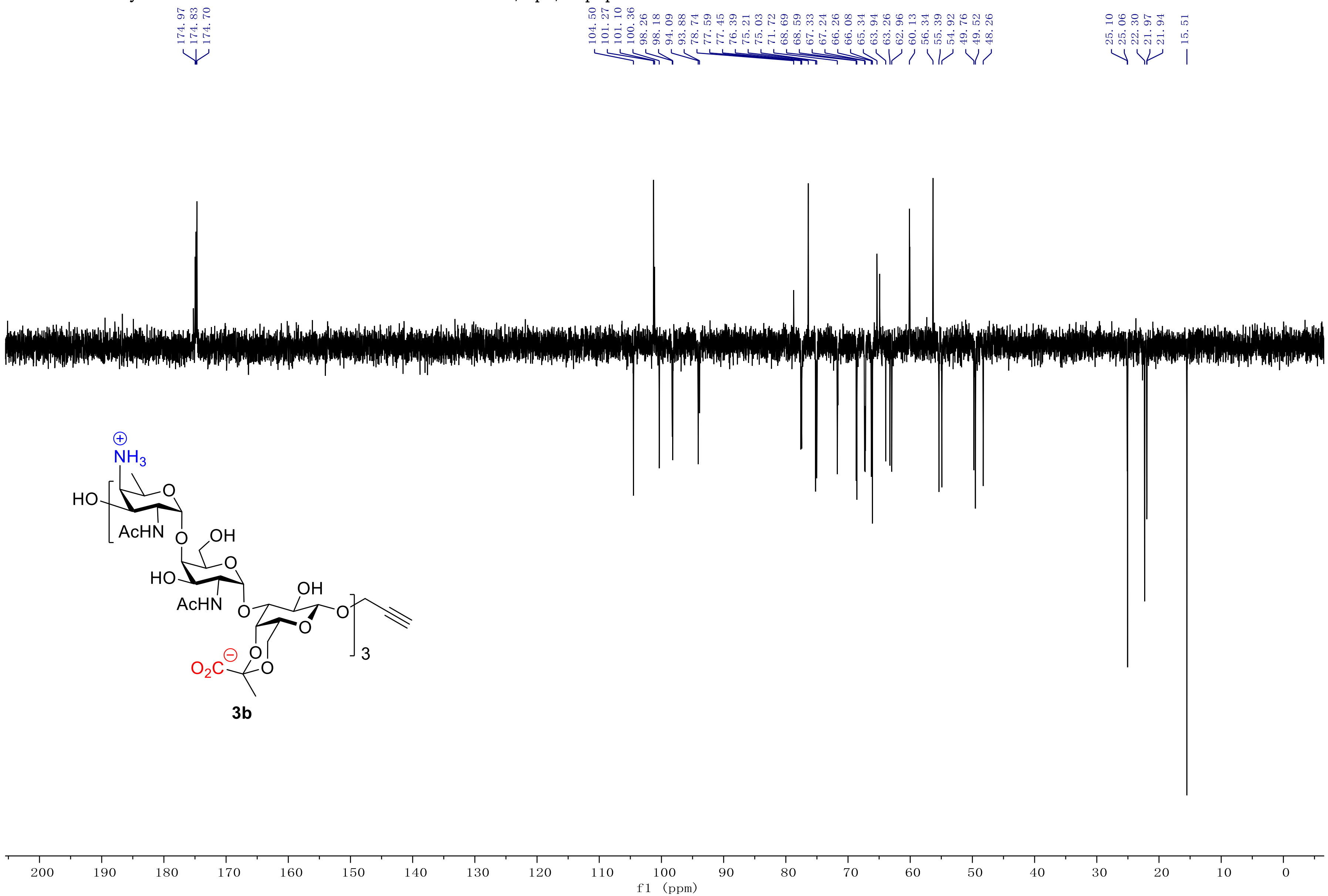

3b

zhen2202Biosyn.25.ser — wz844-B — bbo-h1-cosy D20 /opt/topspin2.1 nmrafd 7

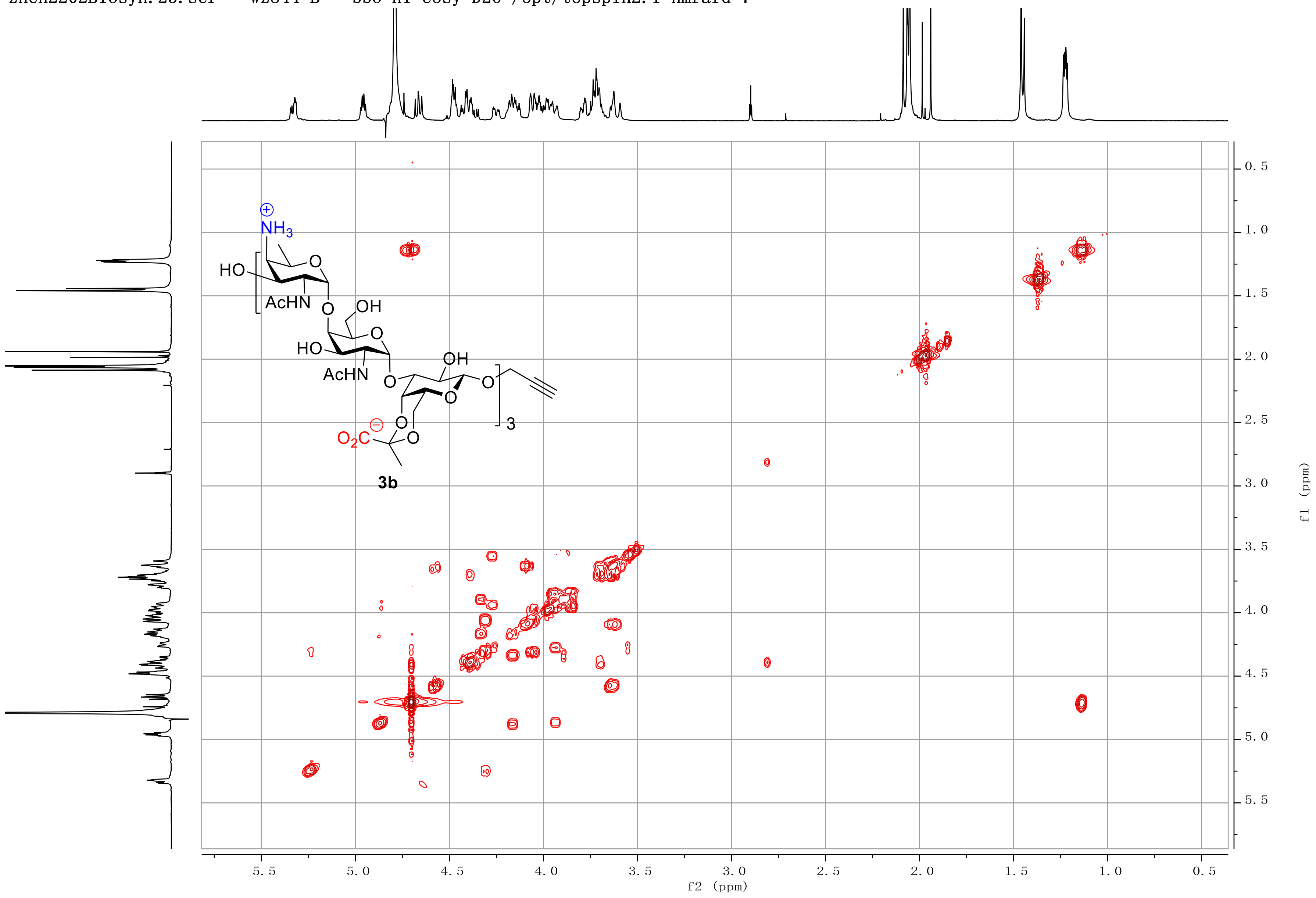

zhen2202Biosyn.26.ser — wz844-B — bbo-c13-HSQC D2O /opt/topspin2.1 nmrafd 7

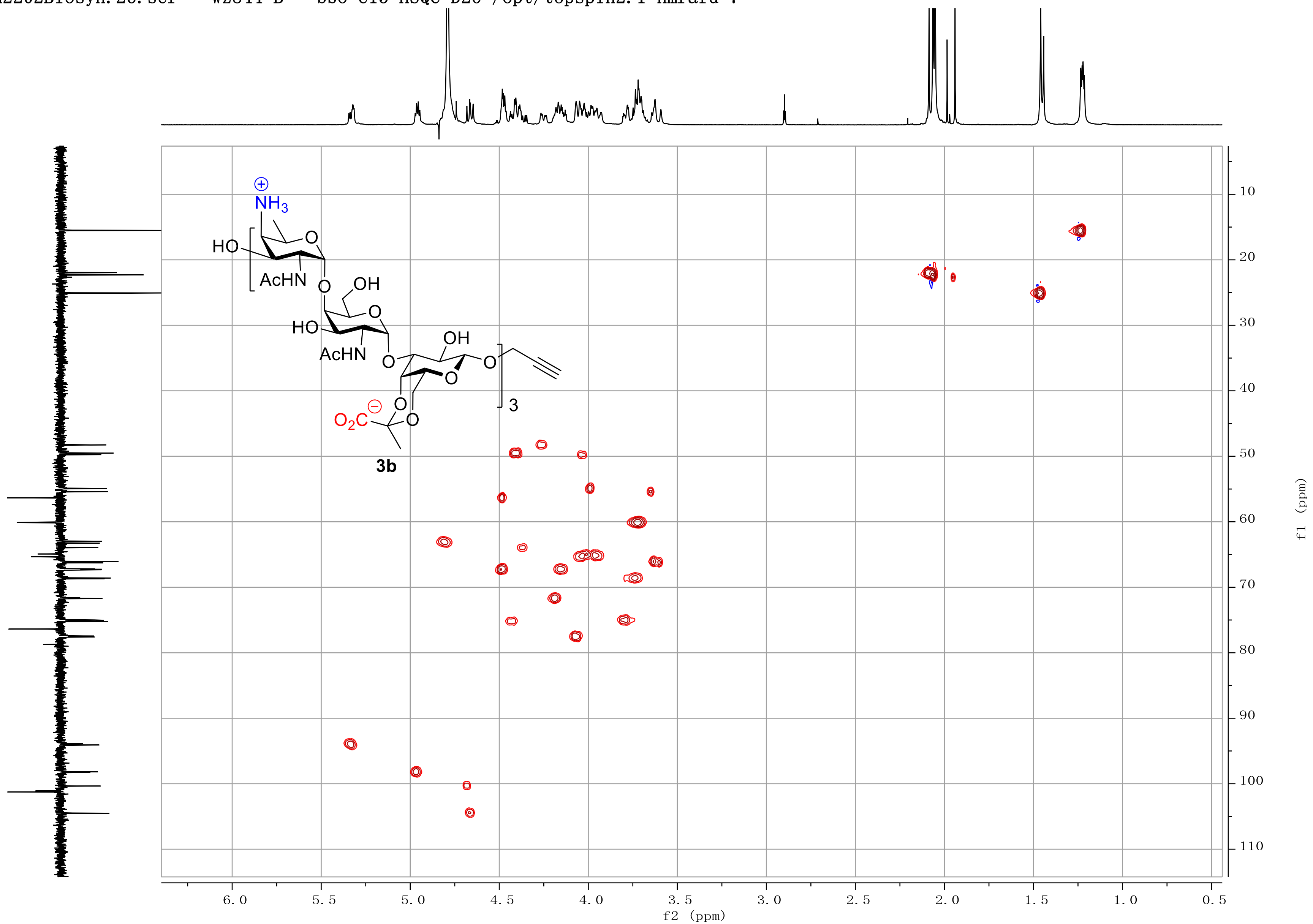

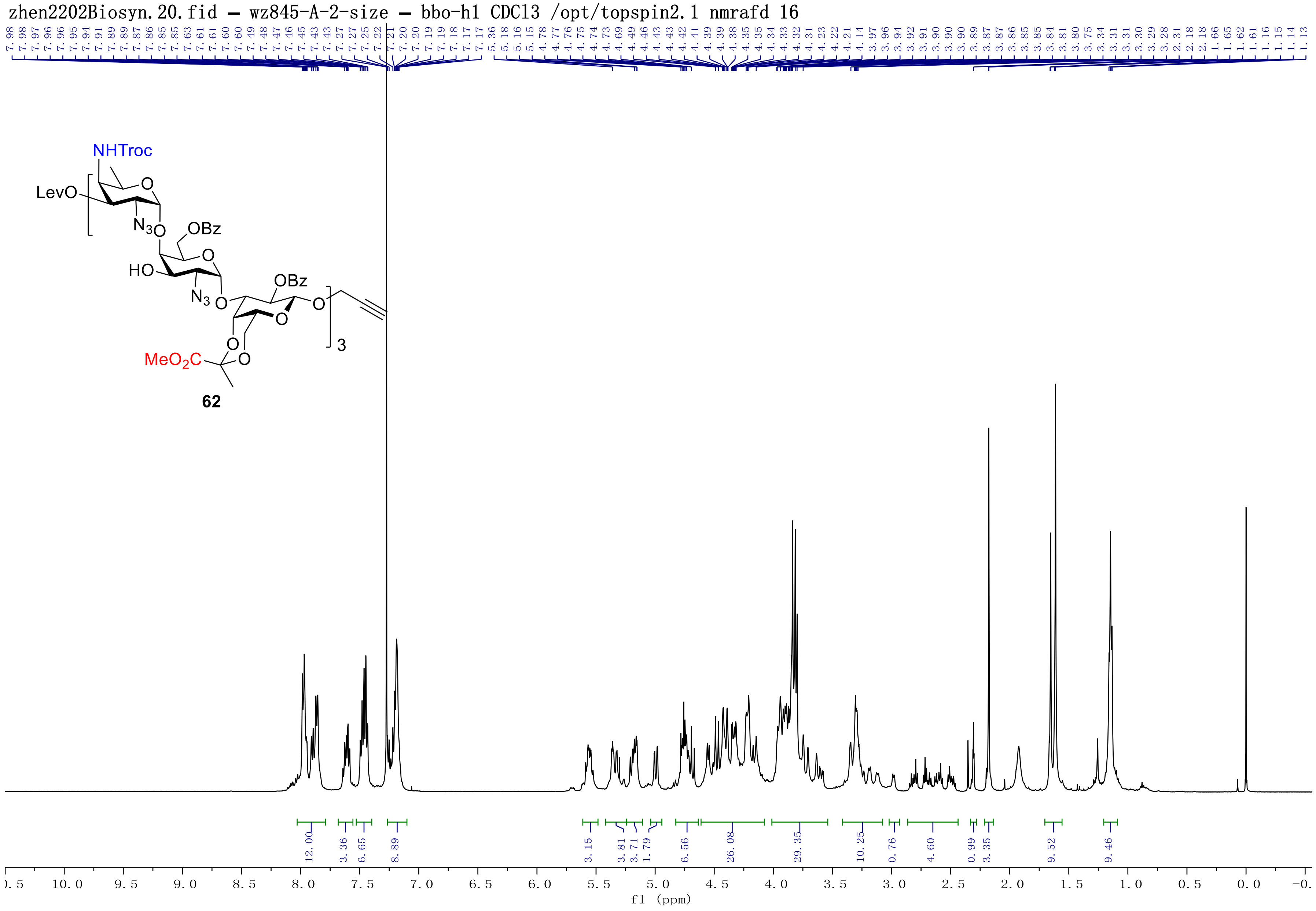

zhen2202Biosyn.20.fid — wz845-A-2-size — bbo-h1 CDCl3 /opt/topspin2.1 nmrafd 16
NHTroc
LevO
O
N3
OBz
HO
N3
OBz
MeO2C
3
62
f1 (ppm)

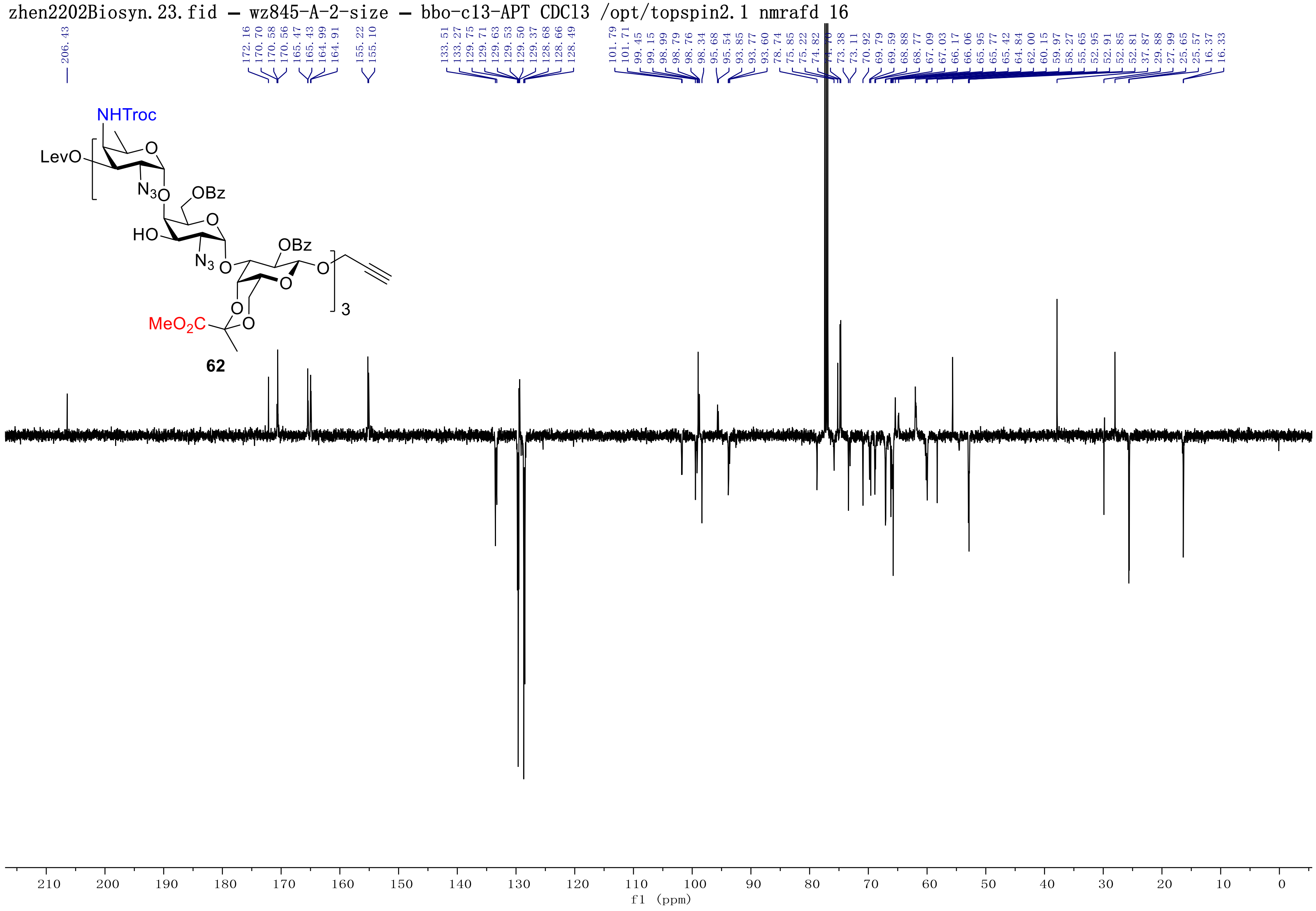
zhen2202Biosyn.23.fid — wz845-A-2-size — bbo-c13-APT CDCl3 /opt/topspin2.1 nmrafd 16
206.43
172.16 170.70 170.58 170.56 165.47 165.43 164.99 164.91
155.22 155.10
133.51 133.27 129.75 129.71 129.63 129.53 129.50 129.37 128.68 128.66 128.49
101.79 101.71 99.45 99.15 98.99 98.79 98.76 98.34 95.68 95.54 93.85 93.77 93.60
78.74 75.85 75.22 74.82 74.70 73.38 73.11 70.92 69.79 69.59 68.88 68.77 67.09 67.03 66.17 66.06 65.95 65.77 65.42 64.84 62.00 60.15 59.97 58.27 55.65 52.95 52.91 52.85 52.81 37.87 29.88 27.99 25.65 25.57 16.37 16.33
NHTroc
LevO
O
N3
O
OBz
HO
N3
O
OBz
O
3
MeO2C
O
O
62
210 200 190 180 170 160 150 140 130 120 110 100 90 80 70 60 50 40 30 20 10 0
f1 (ppm)

zhen2202Biosyn.21.ser — wz845-A-2-size — bbo-h1-cosy CDCl3 /opt/topspin2.1 nmrafd 16

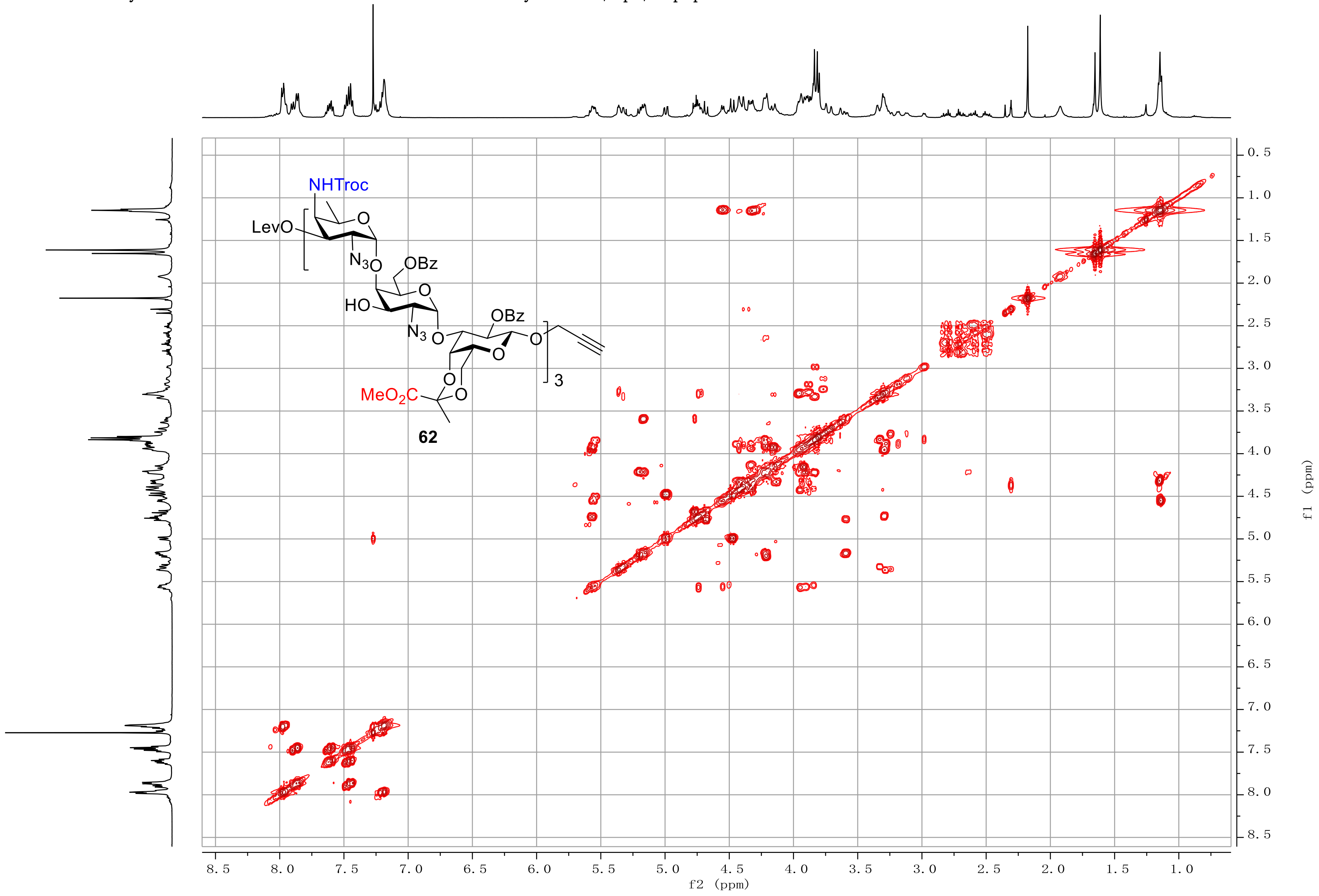

zhen2202Biosyn.22.ser — wz845-A-2-size — bbo-c13-HSQC CDCl3 /opt/topspin2.1 nmrafd 16

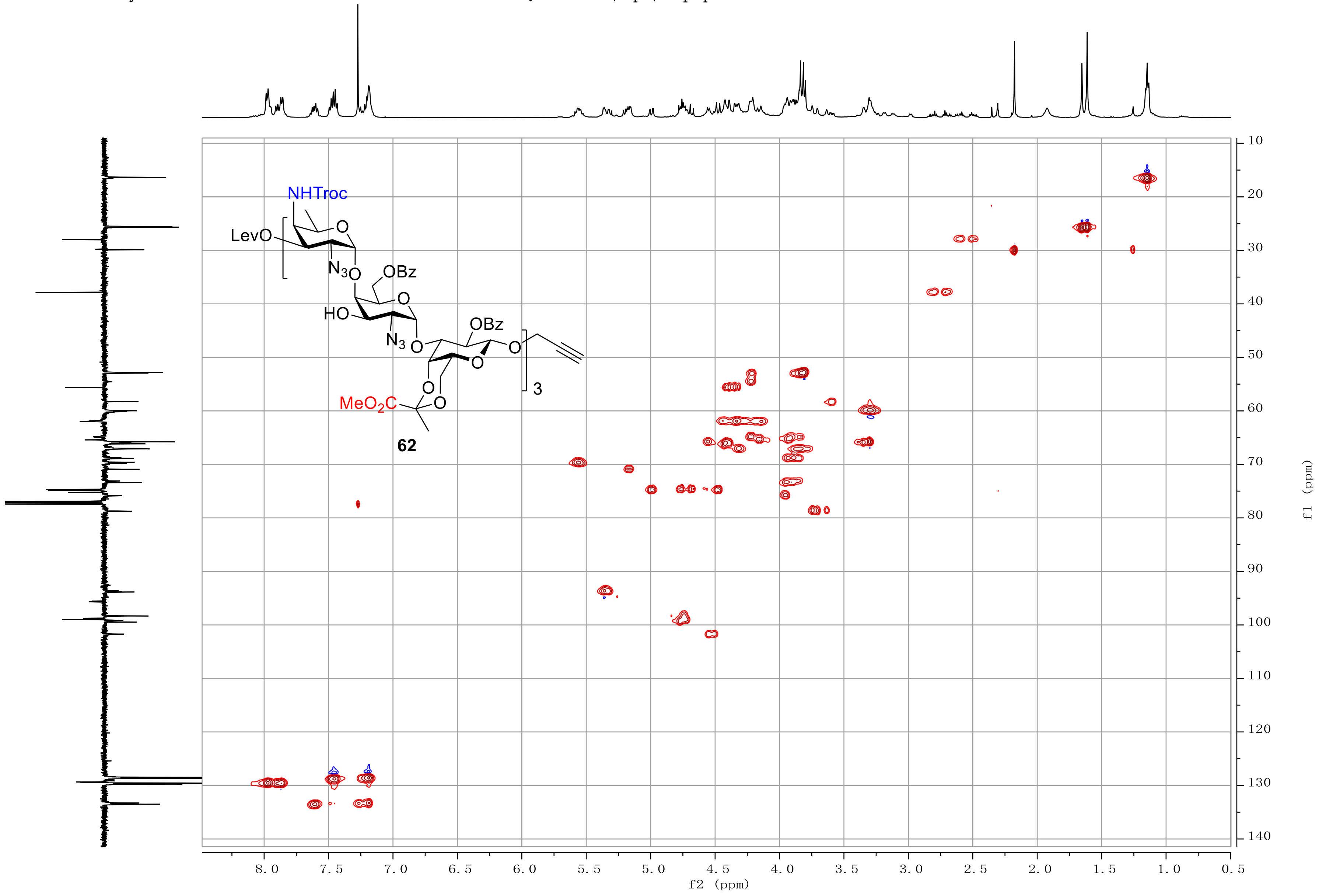

2202zhen.11.fid — wz846 — h1 CDCl3 /opt/DATA nmrafd 28

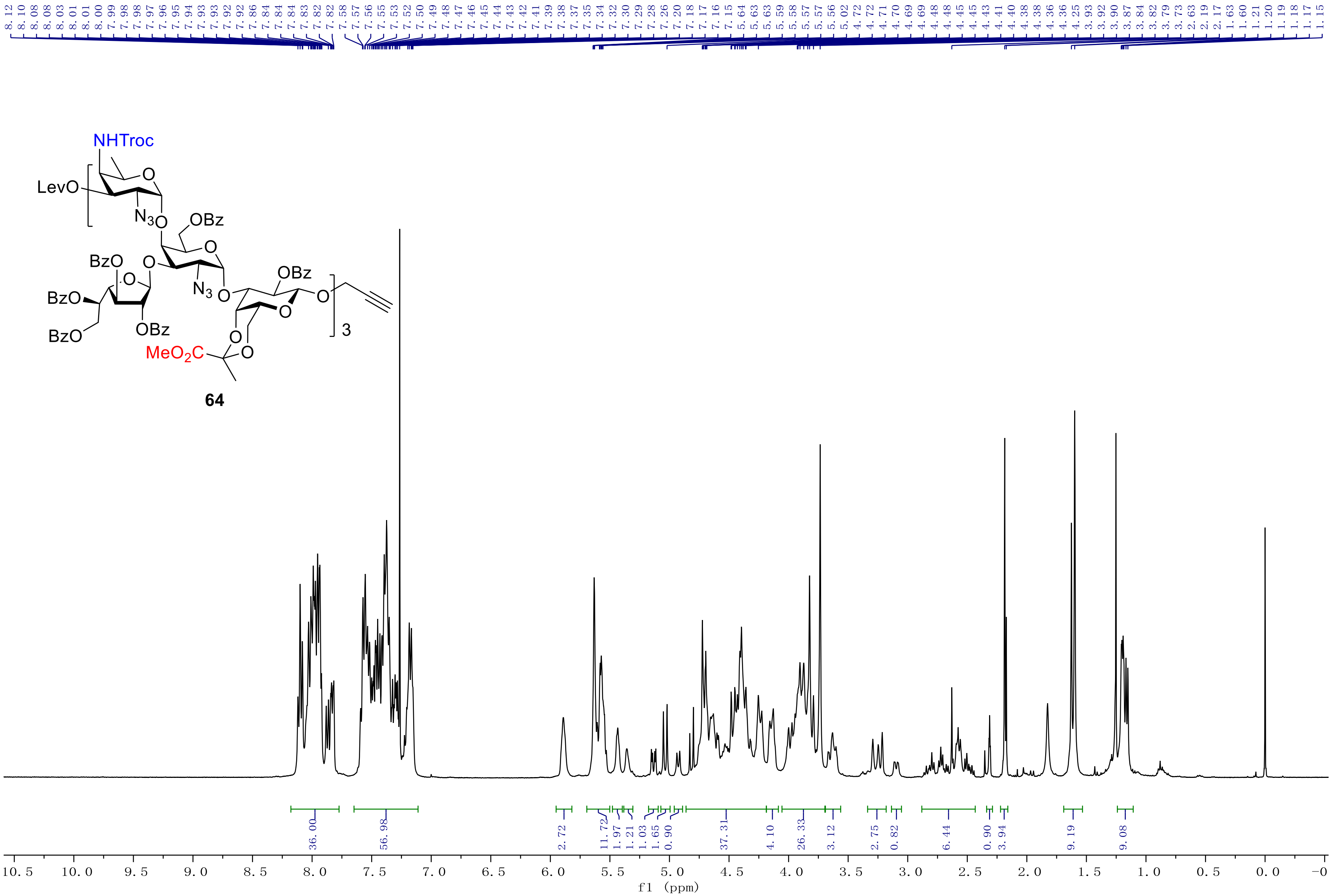

2202zhen.15.fid — wz846 — c13APT CDCl3 /opt/DATA nmrafd 28

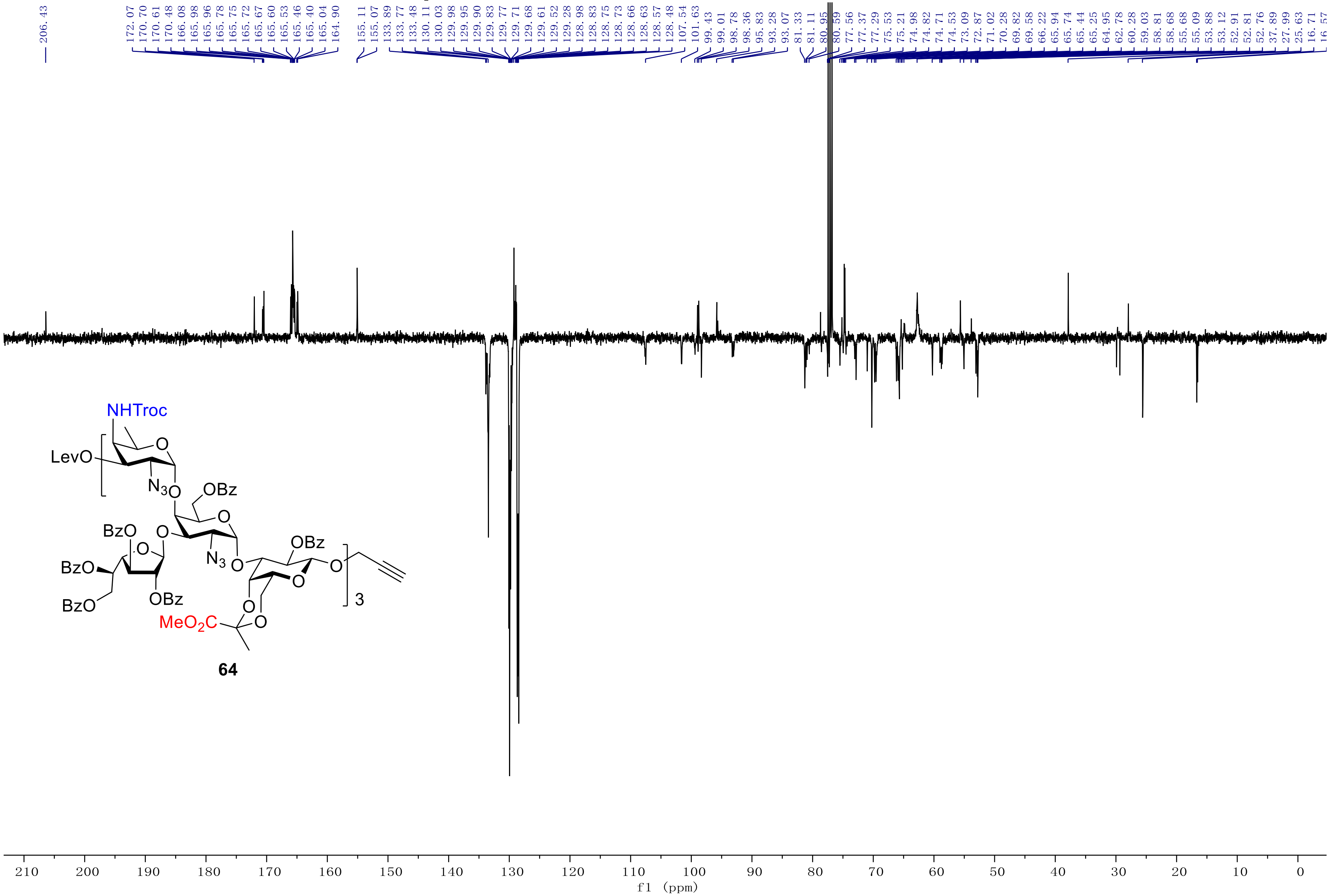

2202zhen.13.ser — wz846 — h1COSY CDCl3 /opt/DATA nmrafd 28

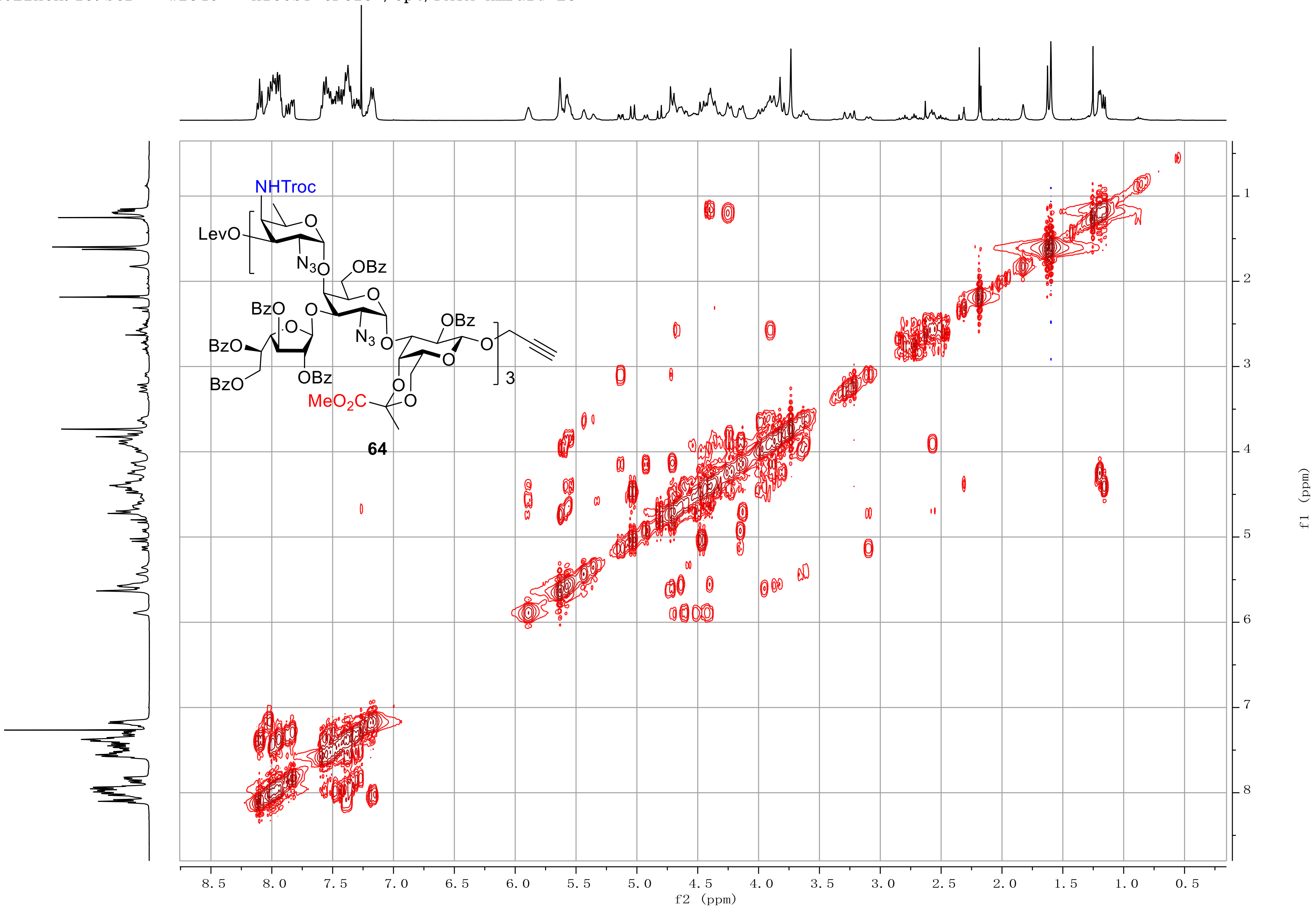

2202zhen.14.ser — wz846 — c13HSQC CDCl3 /opt/DATA nmrafd 28

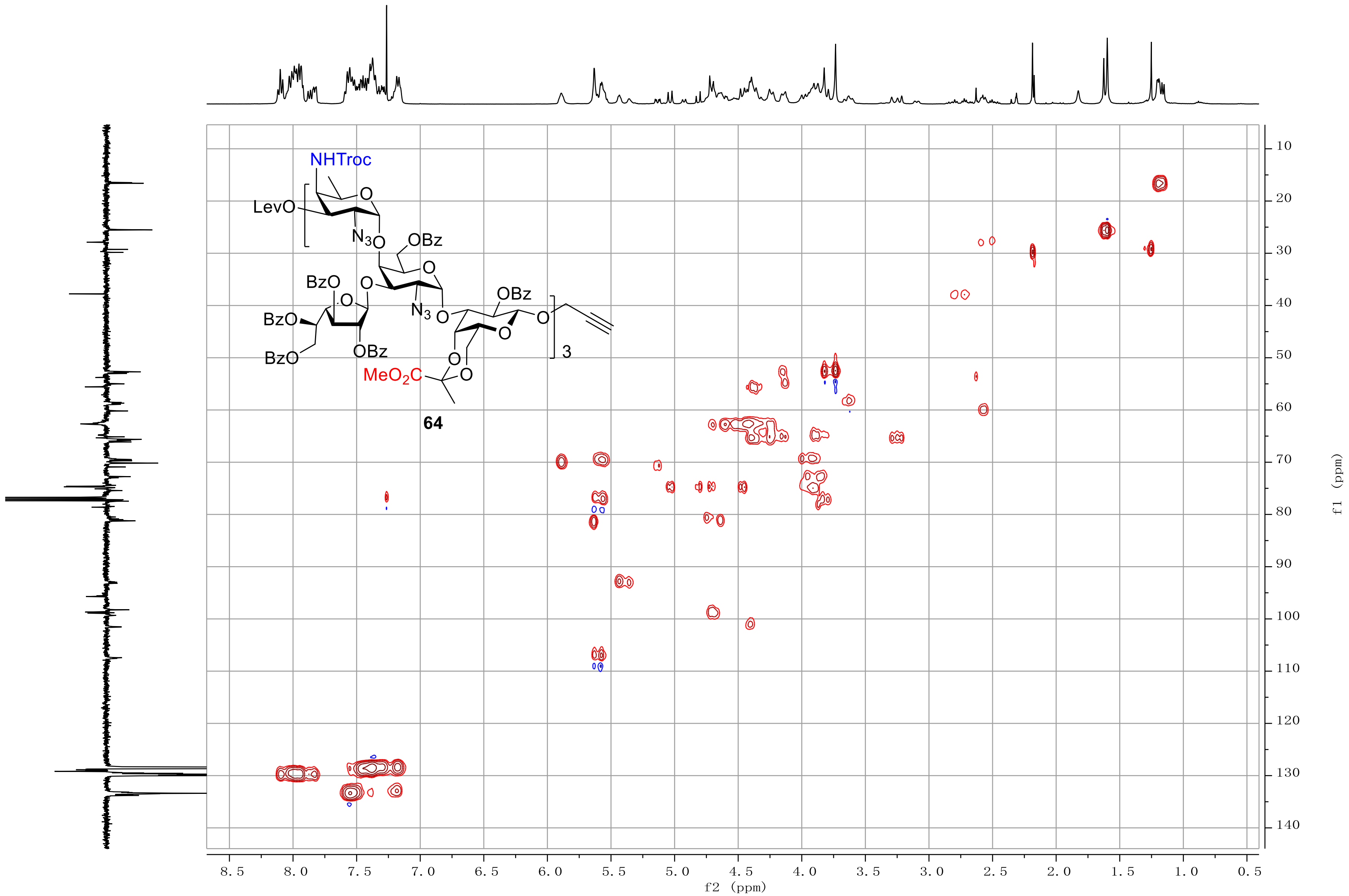

zhen2203biosyn.4.fid — wz848-B; 5.5 mg — bbo-h1 D2O /opt/topspin2.1 nmrafd 12

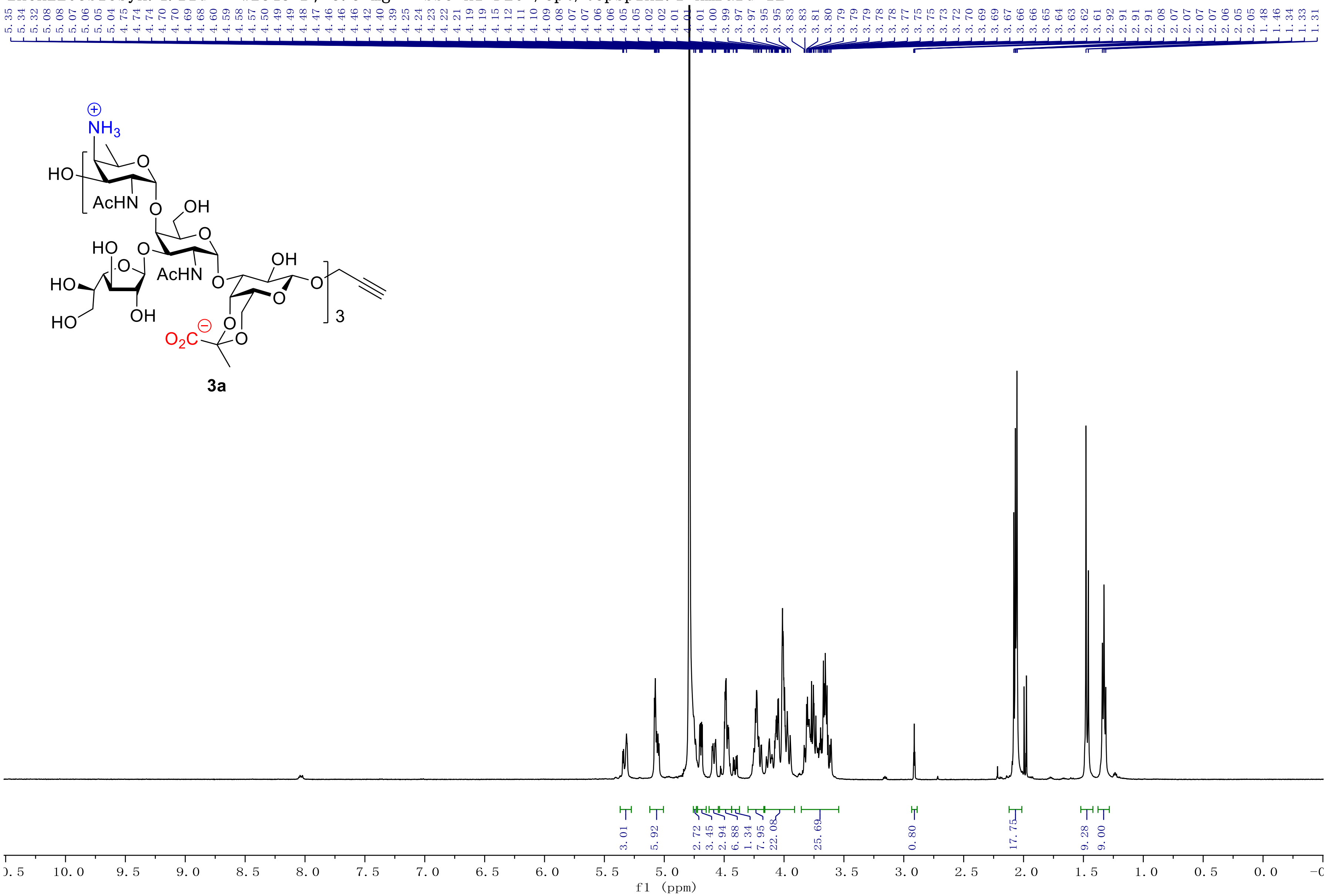

3a

zhen2203biosyn.8.fid — wz848-b, 5.5 mg — bbo-c13-APT D2O /opt/topspin2.1 nmrafd 16

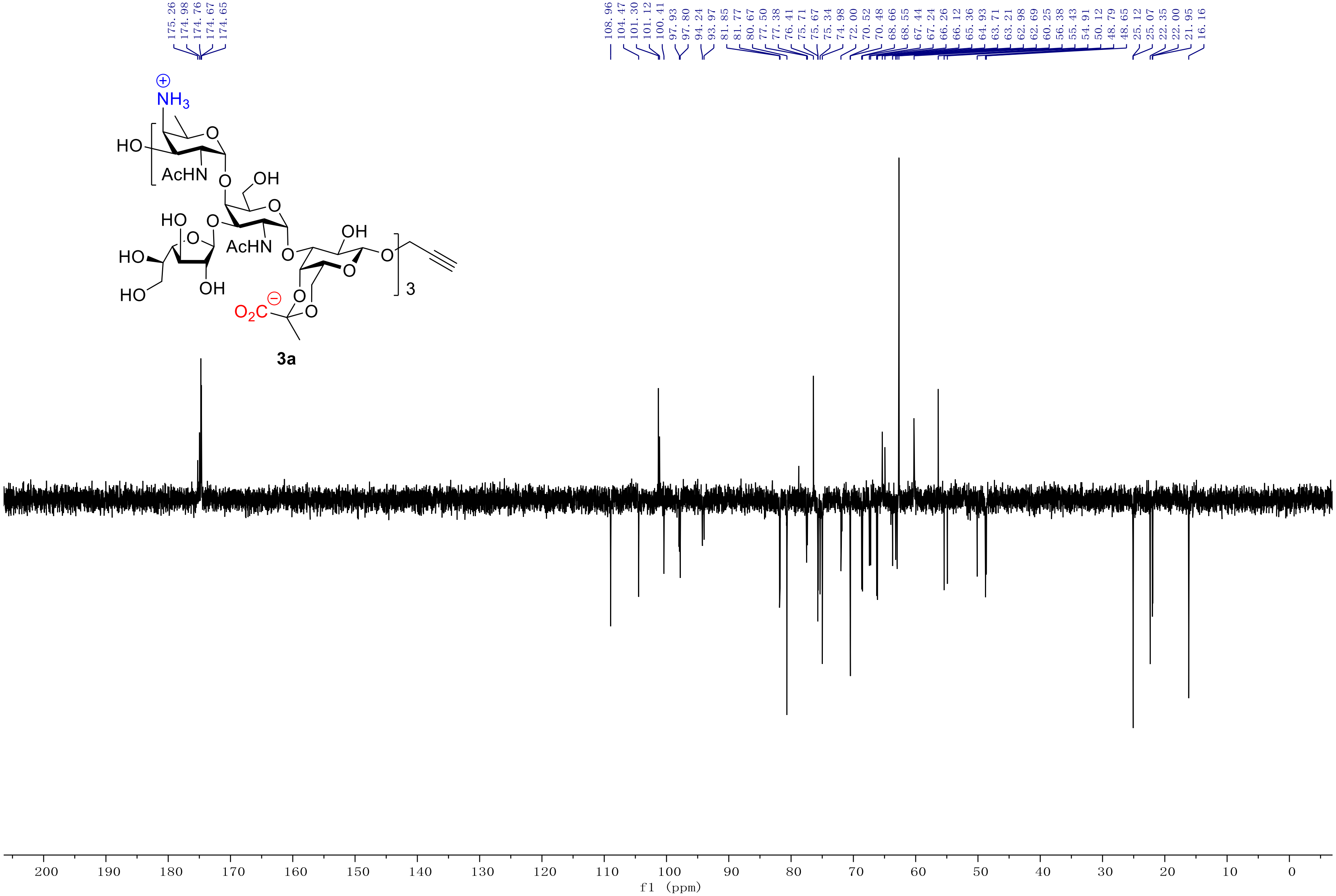

zhen2203biosyn.5.ser — wz848-B; 5.5 mg — bbo-h1-cosy D2O /opt/topspin2.1 nmrafd 12

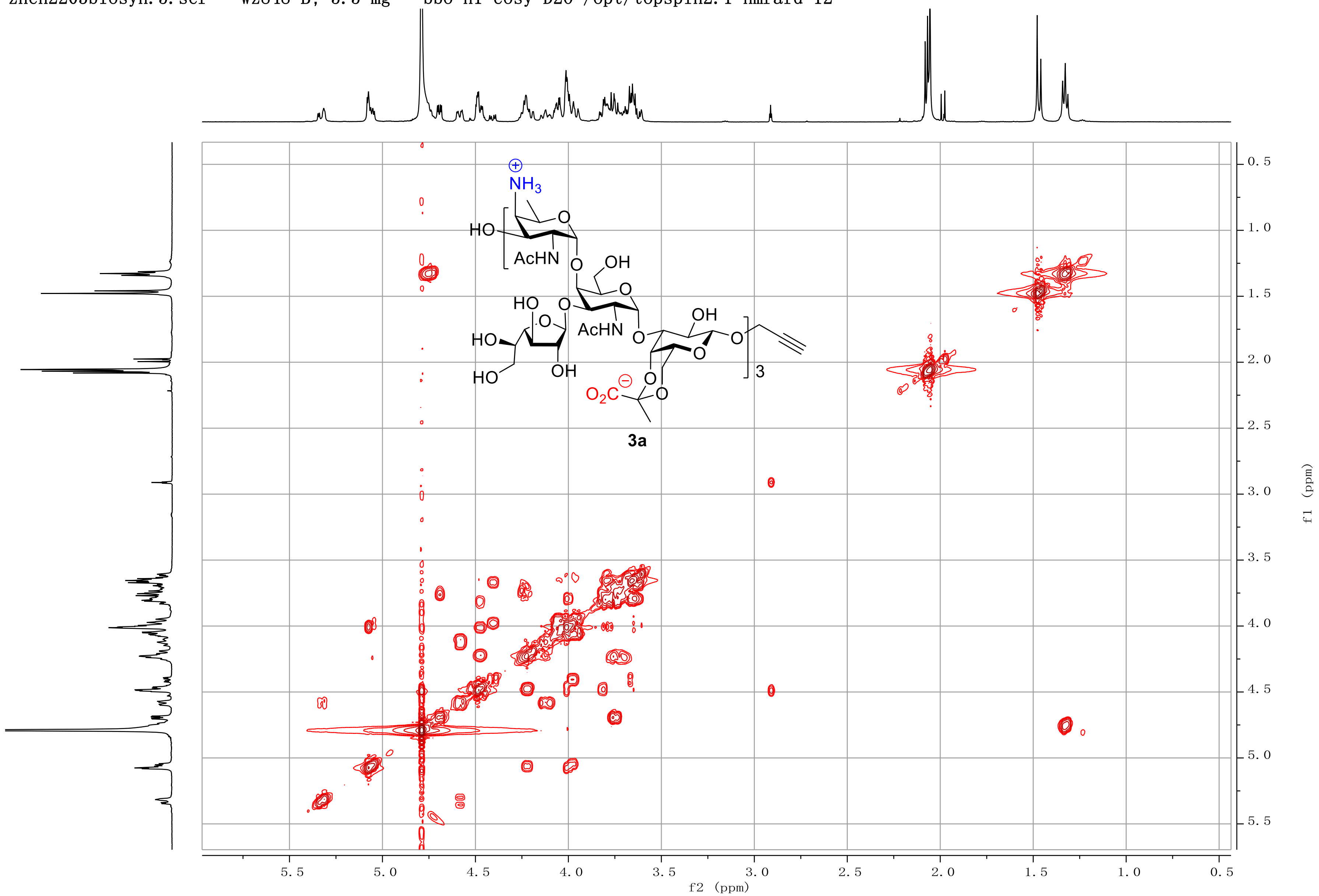

zhen2203biosyn.6.ser — wz848-B; 5.5 mg — bbo-c13-HSQC D2O /opt/topspin2.1 nmrafd 12

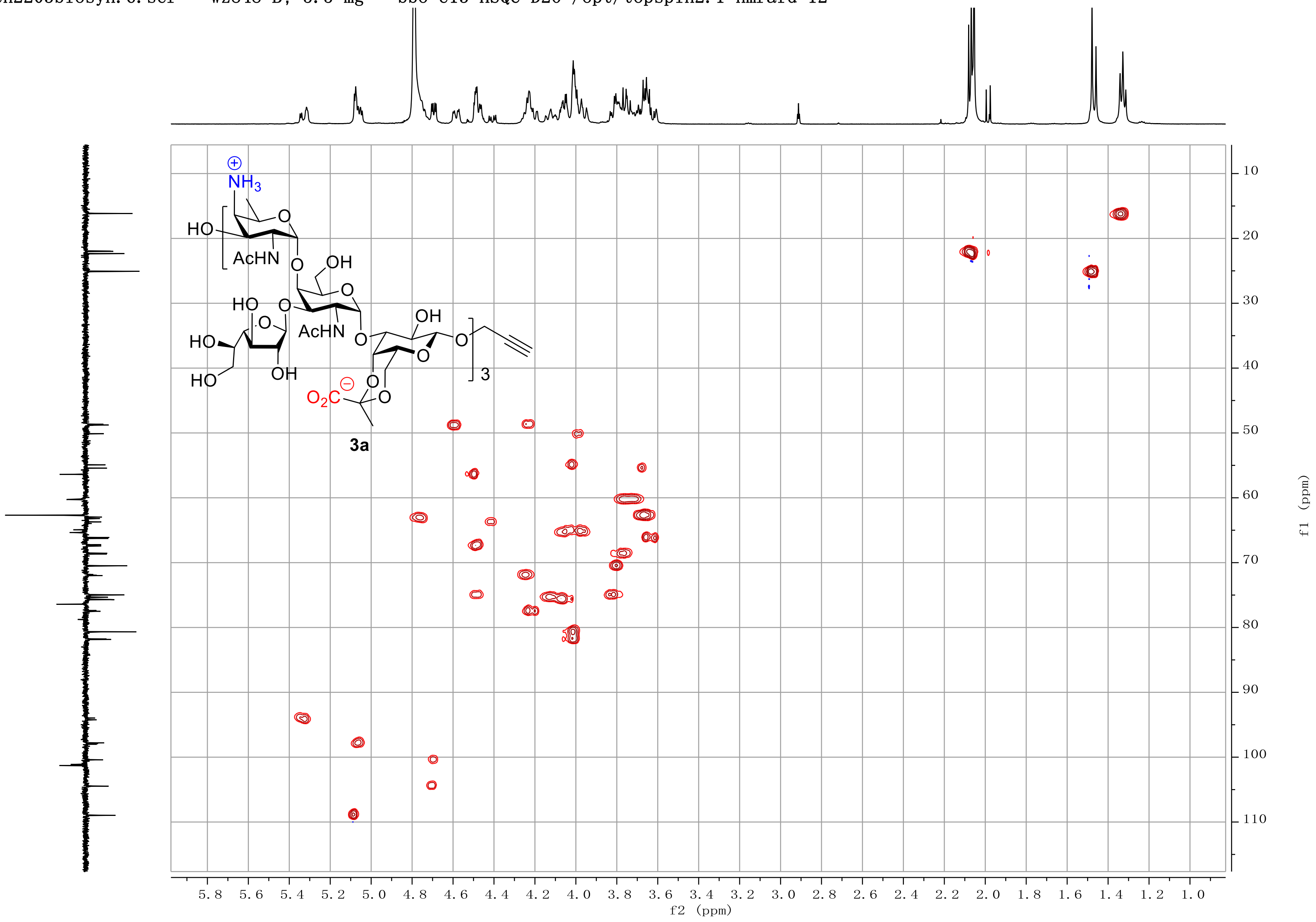

Supplement: Supplementary file 1 — ja3c03976_si_001.pdf [file ja3c03976_si_001.pdf]
